# Visible Light-Mediated Heterodifunctionalization of Alkynylazobenzenes for 2*H*-Indazole Synthesis

**DOI:** 10.1021/acs.orglett.4c00097

**Published:** 2024-02-22

**Authors:** Clara Mañas, Estíbaliz Merino

**Affiliations:** † Universidad de Alcalá, Departamento de Química Orgánica y Química Inorgánica, Instituto de Investigación Andrés del Río (IQAR), Facultad de Farmacia, 28805 Alcalá de Henares, Madrid, Spain; ‡ Instituto Ramón y Cajal de Investigación Sanitaria (IRYCIS), Ctra. de Colmenar Viejo, Km. 9.100, 28034 Madrid, Spain

## Abstract

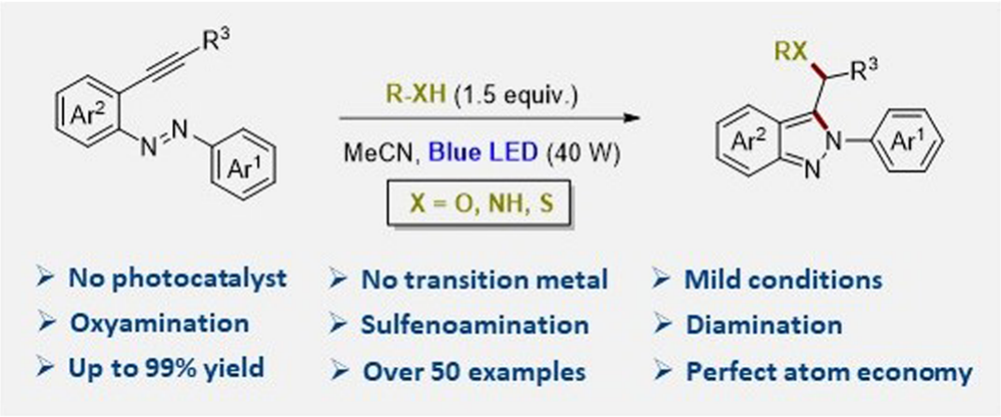

We disclose the heterodifunctionalization of alkynylazobenzenes promoted exclusively by visible light in the absence of any transition metal and/or photocatalyst. This reaction features excellent regioselectivity on a broad variety of substrates with perfect atom economy. Alcohols, carboxylic acids, thiols, amides, heterocycles, and even water are suitable substrates for the promotion of the oxyamination, sulfenoamination, and diamination reactions. In this manner, biologically active indazole scaffolds can be rapidly assembled from alkyne feedstocks.

Alkynes are some of the most common fundamental and sustainable chemical raw materials for the pharmaceutical, agrochemical, and materials industries, and their use in chemical reactions forms the basis of modern catalysis and synthesis.^1,2^ The most prevalent mode of reaction of alkynes is the formation of alkenes via a metal-mediated π-insertion process of the triple bond.^3,4^ Difunctionalization of alkynes is an efficient and straightforward strategy for the synthesis of sophisticated scaffolds. This methodology represents one of the most efficient methods for the formation of C–C and C–heteroatom bonds, as there is a simultaneous installation of two functional groups at vicinal positions in a single synthetic step leading to the straightforward construction of complex molecules from simple alkyne feedstocks. Many methods have been developed for C–C and C–X bond formation with photoredox catalysis^5−7^ or metal-mediated cross couplings.^8−10^ The regioselective construction of the C–N bond under mild conditions exemplifies a class of reactions with significant synthetic potential due to the ubiquitous presence of amines in both naturally occurring and synthetic compounds, which manifest high levels of biological activity.^11,12^ The direct amination reaction of simple alkynes, which results in the formation of a C–N bond along with a C–O, C–S, or C–N bond, has scarcely been explored. Some examples such as oxynitration,^13^ thiocyanation–amination,^14^ or sulfonylamination^15^ have been reported to occur via radical mechanisms. The reported examples from Yamame and Lin using alkynylazobenzenes as substrates showed the viability for enabling the formation of C–N and C–C^16^ and C–P^17^ bonds using a Pd/Cu system (Figure 1a). However, the difunctionalization of alkynylazobenzenes by the formation of C–N and C–N, C–O, and C–S bonds is still an uncharted transformation. Despite progress in addressing this issue, regioselective heterodifunctionalization of alkynes under mild conditions in the absence of a transition metal or organophotocatalyst via a polar mechanism remains an exciting synthetic challenge.

**Figure 1:**
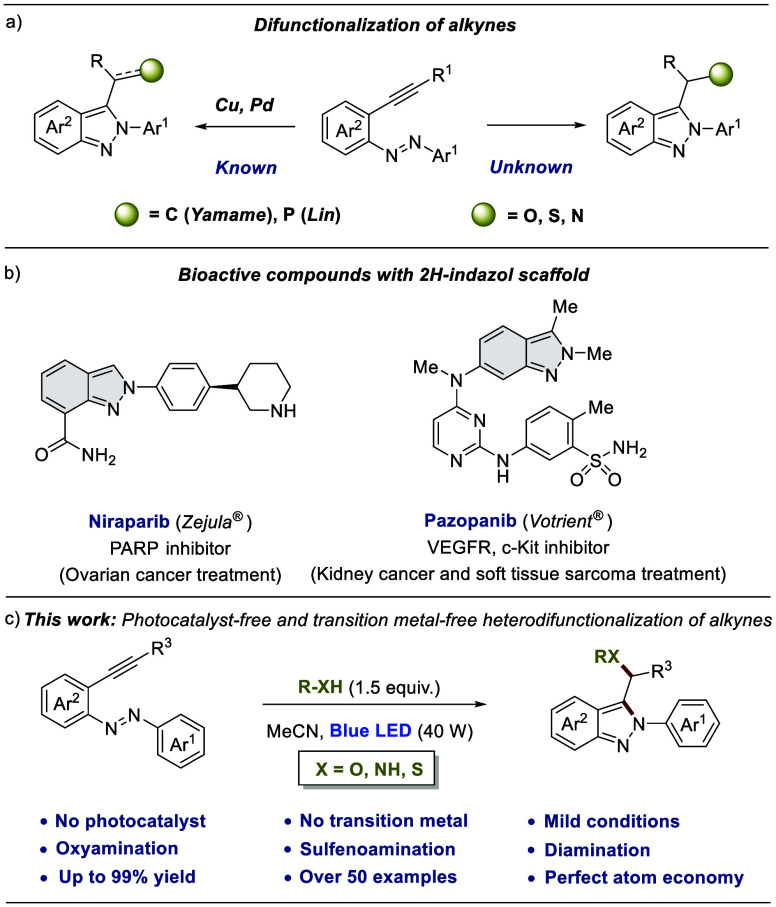
Strategies for the heterodifunctionalization of alkynylazobenzenes.

2*H*-Indazoles constitute prevalent structural motifs in bioactive natural products,^18,19^ and drugs such as niraparib^20^ and pazopanib^21^ (Figure 1b) and 2-aryl indazoles exhibit interesting spectrophotometric properties.^22^ Their preparation from alkynylazobenzenes has been reported using transition metals such as Pd/Cu^16,17^ and Rh^23^ or sensitive reagents such as TBAF.^24,25^ No examples reporting the synthesis of 2*H*-indazoles in the absence of a catalyst or excess of additives have been published to date. Considering their applications, it is of paramount importance to have new and general environmentally sustainable procedures in organic synthesis that reduce the cost of energy. These methods aim to reduce energy costs, minimize the generation of waste, and either reduce or eliminate the use of transition metals.

We decided to investigate the reactivity of alkynylazobenzenes under visible light irradiation, given that these compounds present an alkynyl moiety, which adds an additional point of reactivity to their azobenzene backbone that can be activated by visible light.^26−28^

Herein, we describe the regioselective heterodifunctionalization of terminal and internal alkynylazobenzenes exclusively promoted by irradiation with blue light in the absence of a catalyst or an additive (Figure 1c). In a single reaction step and in the presence of a variety of nucleophiles, two new C–heteroatom bonds are formed with perfect atom economy. The excellent regioselectivity, combined with broad functional group tolerance and mild reaction conditions, showcases the synthetic utility and generality of this transformation. This makes it a valuable tool for assembling relevant blueprints such as 2*H*-indazoles used in the production of pharmaceuticals, bioactive natural products, and ligands for transition metal catalysis.

We started our investigation with **1a**, which was dissolved in methanol (0.1 M) and upon irradiation with blue light (40 W) furnished 2*H*-indazol **2a** in 95% yield (Scheme 1). When the scale was increased 10 times (1 mmol), product **2a** was isolated in 90% yield after filtration over silica gel. X-ray crystallographic analysis of compound **2a** confirmed the structure of the product (details in the Supporting Information).

**Scheme 1:**
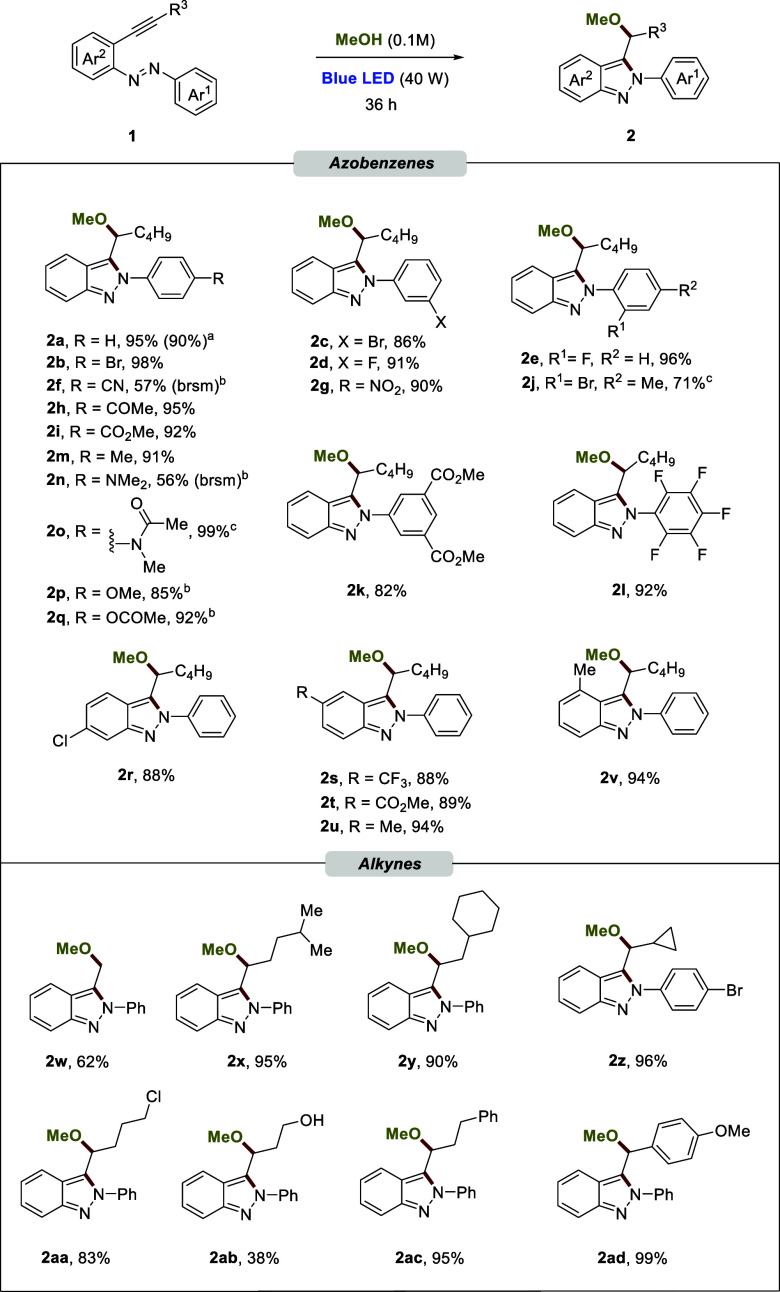
Azobenzene and Alkyne Scope of the Oxyamination Reaction

Next, we turned our focus to explore the generality of this oxyamination reaction through the simple procedure of dissolving a variety of alkynylazobenzenes **1** in methanol and irradiating them with a blue light-emitting diode (40 W) (Scheme 1). Alkynylazobenzenes that bear a halogen atom in the *ortho*, *meta*, or *para* position on Ar^1^ furnished the corresponding products (**2b**–**2e**) in excellent yields. Electron-withdrawing groups [CN, NO_2_, C(O)Me, and CO_2_Me] were evaluated, and the corresponding 2*H*-indazoles **2** were also obtained in excellent yields. Rings with two substituents successfully gave the oxyamination reaction (**2j** and **2k**). The fluorinated compounds are of great interest in medicinal chemistry because they have a beneficial effect in modulating the physicochemical properties of active pharmaceutical ingredients.^29^ Under the standard conditions, **1l** and **1s** afforded 2*H*-indazoles **2l** and **2s**, respectively, in excellent yields. Methyl (**2m**), dimethylamine (**2n**), and methoxy (**2p**) derivatives confirmed the compatibility of electron-donating substituents under the reaction conditions.

Amides and esters were also revealed as suitable substituents for obtaining **2o** and **2q** by simple irradiation of a solution of the corresponding alkynylazobenzene **1** in methanol. Modification of Ar^2^ of the substrates with electron-withdrawing and electron-donating groups in different positions of the aromatic rings afforded in excellent yields products **2r**–**2v**. Attempts to synthesize azo compounds with an alkyl moiety instead of Ar^1^ were unsuccessful (details in the Supporting Information).^30,31^ Modifications of the alkynyl moiety offered an additional reason for structural diversification. The substrate with a terminal alkyne provided product **2w** in 62% yield probably due to its low stability. Branched chains and cycloalkyls such as cyclohexyl and cyclopropyl provided the products (**2x**–**2z**) in excellent yields. Halogen chains are also compatible with this transformation (**2aa**). Remarkably, **2ab** with a primary free alcohol was synthesized in moderate yield. An aromatic ring on the alkyne also led to the corresponding product (**2ac**) in almost quantitative yield. Substrates with a phenyl ring directly attached to the alkyne group were also revealed as compatible substrates under the reaction conditions, furnishing **2ad** in 99% yield.

Encouraged by the general application of a wide variety of alkynylazobenzenes with methanol under irradiation with blue light in the oxyamination reaction, we then systematically evaluated the transformation with respect to the scope of different alcohols (Scheme 2). In this case, a solution of **1a** in acetonitrile (0.1 M) was prepared and 1.5 equiv of the alcohol were used. Ethanol was successfully incorporated into **2ae** in 75% yield. Bulkier alcohols such as *tert*-butanol (**2af**) or *iso*-propanol (**2ag**) confirmed that the oxyamination reaction under standard conditions is not sensitive to the steric constraints. X-ray crystallographic analysis of the latter compound confirmed the structure (details in the Supporting Information). Terminal alkenyl and alkynyl alcohols are also compatible with the reaction conditions (**2ah** and **2ai**, respectively). Alcohols with terminal halogens such as fluorine (**2aj**) or iodine (**2ak**) were successfully incorporated into the corresponding alkynylazobenzene **1a**. Less nucleophilic alcohols such as phenol furnished the product (**2al**) in high yield. Interestingly, norbornanyl and adamantyl alcohols were also incorporated under the standard conditions (**2am** and **2an**, respectively). We next sought to explore the possibility of applying this transformation in the context of a structural diversification natural product synthesis setting.^32,33^ To this end, we were pleased to observe the successful conversion of the tocopherol-derived alcohol into product **2ao** in 40% yield. Product **2ap** was produced when the oxyamination protocol was applied with an estrone-derived alcohol in a similar yield. Hydroxyamination was suitable when water was used, allowing the isolation of free alcohol **2aq** in very good yield. These transformations highlight the potential of this methodology to broaden the structural diversity of complex biologically active molecules and influence structure–activity relationship (SAR) optimization in medicinal chemistry studies. Next, we focused our attention on exploring other types of nucleophiles as carboxylic acids. As shown in Scheme 2, with aliphatic and aromatic carboxylic acids, oxyamination took place with moderate to good yields (**2ar** and **2as**, respectively). Cyclic tertiary carboxylic acids could be utilized as counterparts, giving the transformation in an efficient manner (**2at** and **2au**). Similarly, this practical protocol was applied in the sulfenoamination reaction, exhibiting excellent functional compatibility and efficiency with aliphatic and aromatic thiols (**2av** and **2aw**, respectively). Interestingly, when the reaction was performed with 2-mercaptoethanol, a mixture of compounds was obtained (**2ax** and **2ax′**). The major product, isolated in 65% yield, is the outcome of the attack by the most nucleophilic sulfur atom, and the product resulting from the attack of the alcohol was isolated in 23% yield. Attempts to react with primary and secondary amines, such as isopropylamine and diethylamine, as well as with aromatic amines, like aniline, proved to be unsuccessful (Figure S9). Amides have been shown to be powerful nucleophiles in the presence of strong bases.^34^ We were able to achieve the incorporation of acetamide into the 2*H*-indazole scaffold through the nitrogen atom with a moderate yield, even in the absence of a base (**2ay**). This result highlights the reactivity of the excited species generated by the irradiation of alkynylazobenzene **1**. Finally, indole was tested to extend the synthetic potential of this transformation to heterocycles, leading to **2az** in 62% yield.

**Scheme 2:**
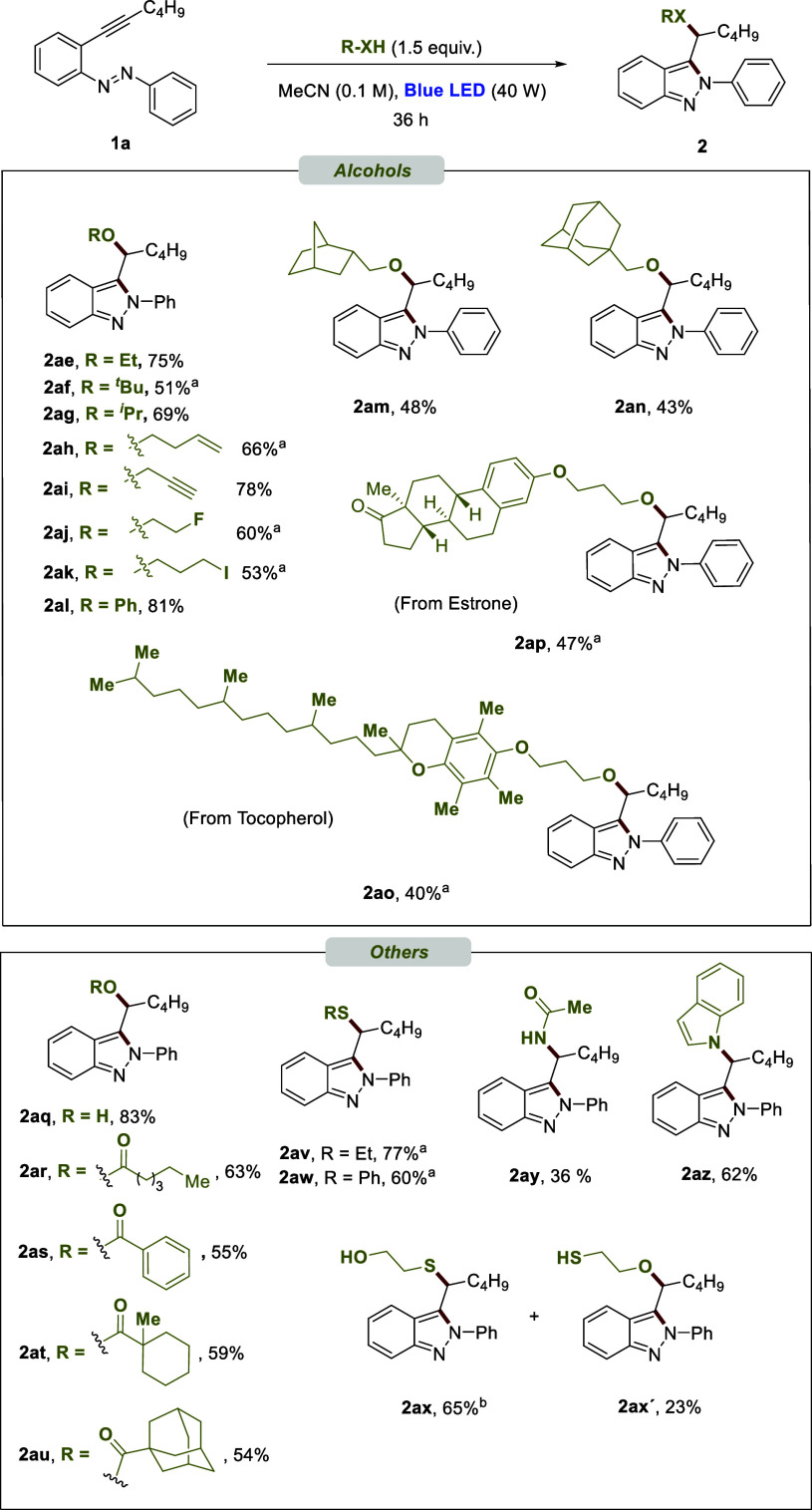
Scope of the Oxyamination, Sulfenoamination, and Diamination Reactions

To gain more insight into the mechanism of this heterodifunctionalization of alkynylazobenzenes, several mechanistic experiments were performed. *Cis*- and *trans*-**1a** were separately subjected to the standard reaction conditions, and the isomerization was monitored by ^1^H nuclear magnetic resonance (NMR). In both cases, **2a** was obtained in comparable yields. A plot of the temporal concentration over time revealed that, after only 10 min, both isomers converge to an ∼1:1.9 *cis*:*trans* ratio (Figures S3 and S4). Such a photostationary state, reached at a regime much faster than the product reaction itself, suggests that both isomers will be present at the outset of the reaction, regardless of the initial azo group geometry. A deuteration experiment with CD_3_OD was carried out to observe the formation of deuterated **2a** in 92% yield, with >99% deuteration. Analysis of the progress of the reaction by ^1^H NMR revealed that (*Z*)-**1a** is generated during the reaction (Figure S5).

Light proved to be crucial for successfully performing the reaction, pointing to the formation of the S_1_ excited state of the alkynylazobenzene molecule (Figure S10). Furthermore, when the reaction mixture was heated to 70 °C, **2a** was isolated in only 16% yield. This assumption was supported by an additional experiment with (*E*)-1-(hex-1-yn-1-yl)-2-styrylbenzene, which showed a *trans*:*cis* isomerization ratio of 1:2.3 under the given reaction conditions (Figure S8). This experiment seems to indicate that the reaction begins with the absorption of light by alkynylazobenzene **1** and excitation to the S_1_ state. The reaction of **1a** to **2a** was performed under standard conditions in the presence of 5 equiv of piperylene, which is an established triplet quencher (Figure S6).^35^ The fact that there was no reduction in the rate or yield of **2a** suggests that the reaction does not involve a triplet intermediate. The yield of the oxyamination reaction was not adversely affected by the presence of radical inhibitors such as TEMPO or 2,2-dimethyl-3,4-dihydro-2*H*-pyrrole 1-oxide (Figure S7). This result could rule out the formation of radical intermediates, supporting the hypothesis of a polar mechanism. On the basis of the results we achieved, a proposed mechanism is outlined in Figure 2. The first step of the reaction would be the excitation of **1a** under visible light irradiation. The nitrogen atom of the azo group of this generated excited species can attack the alkynyl moiety, generating intermediate **I** by capturing a hydrogen of methanol. The addition of methanol leads to **2a** through transition state **TS**_**I-2a**_ (Δ*G*^⧧^ = 17.7 kcal mol^–1^).

**Figure 2:**
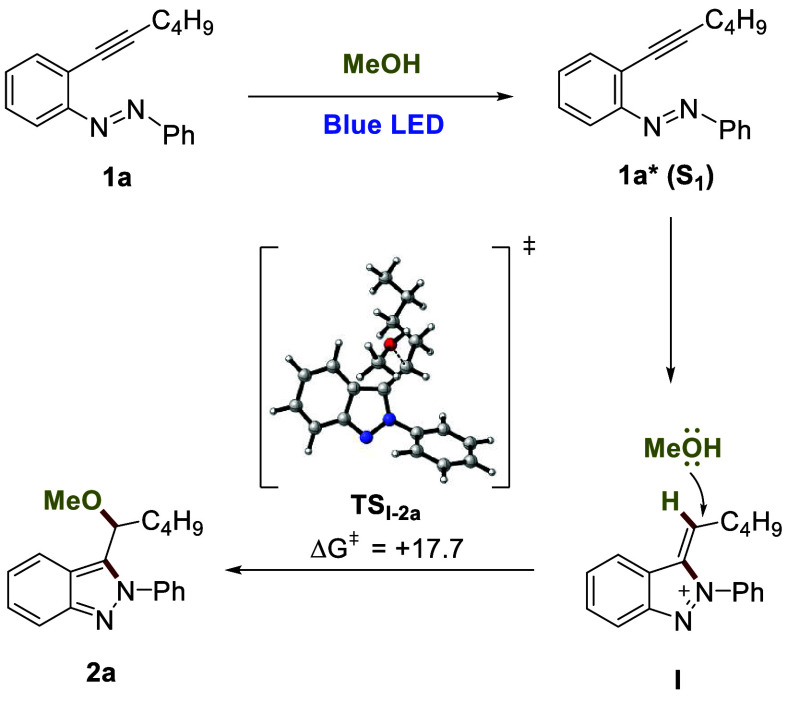
Mechanistic proposal.

To expand the utility of indazoles, we focused our attention on exploring the derivatization of 2*H*-indazoles **2** (Figure 3). Arylation of **2b** by the Suzuki coupling reaction gave access to indazole **2ba** with a biphenyl moiety. Amino indazole **2bb** could be achieved by reduction of the nitro group of **2g** in excellent yield. The hydrolysis of ester groups led to **2bc** in almost quantitative yield. Sulfonyl group-containing compounds constitute an important class of therapeutic agents in medicinal chemistry. Oxidation with *m*-CPBA allowed us to obtain sulfone indazole **2bd** in 49% yield.

**Figure 3:**
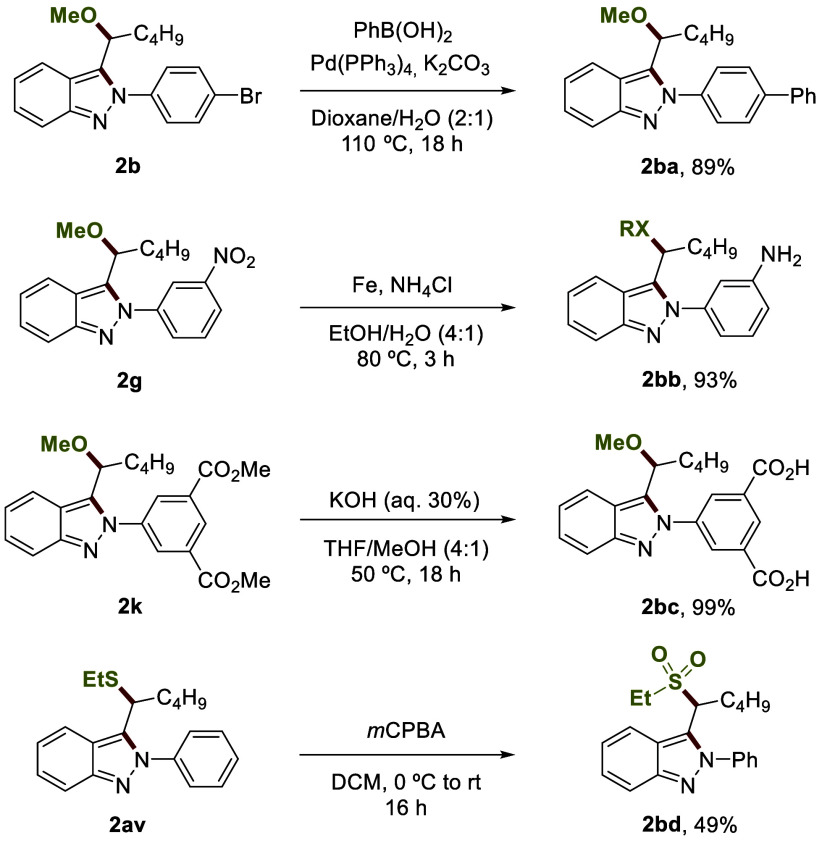
Transformations of 2*H*-indazoles.

In conclusion, we have overcome a formidable challenge in the heterodifunctionalization of alkynes by carrying out this transformation in the absence of a catalyst and/or additive simply by irradiation of an alkynylazobenzene with visible light. Oxyamination, sulfenoamination, and diamination reactions can be carried out in a single reaction step under mild reaction conditions with a wide range of substrates. The process is regioselective with perfect atom economy and provides access to 2*H*-indazole skeletons, which are in great demand in medicinal chemistry. We postulate that this method harnesses the great potential of visible light to promote chemical transformations and advances the broader field of alkyne difunctionalization to a new dimension.

## Data Availability

The data underlying this study are available in the published article and its Supporting Information.

## Supporting Information Available

Experimental procedures, characterization and crystal data, and additional experimental details (PDF)
