# Supplementary material for: Visible Light-Mediated Heterodifunctionalization of Alkynylazobenzenes for 2H-Indazole Synthesis
Source: Org Lett. 2024 Feb 22;26(9):1868–73. doi: 10.1021/acs.orglett.4c00097 (PMC10928707; doi:10.1021/acs.orglett.4c00097)
Supplement: Supplementary file 1 — ol4c00097_si_001.pdf [file ol4c00097_si_001.pdf]

Supplementary Materials for

Visible light-mediated heterodifunctionalization of alkynylazobenzenes for 2*H*-indazole synthesis

Clara Mañas[1,2], Estíbaliz Merino[1,2]*

[1]Universidad de Alcalá, Departamento de Química Orgánica y Química Inorgánica, Instituto de Investigación Química "Andrés M. del Río" (IQAR). Facultad de Farmacia, Alcalá de Henares, 28805 Madrid, Spain.

[2]Instituto Ramón y Cajal de Investigación Sanitaria (IRYCIS), Ctra. de Colmenar Viejo, Km. 9.100, 28034, Madrid, Spain.

*E-mail: estibaliz.merino@uah.es

Table of contents

**General information**	**3**
**Control experiments**	**5**
**Procedures for the synthesis of alkynylazobenzenes (1a-1ac) and characterization**	**16**
**General procedure for the synthesis of 2*H*-indazoles (2a-2ay) and characterization**	**42**
**X-Ray crystallographic data**	**69**
**DFT calculations**	**71**
**Copies of NMR spectra**	**76**
**References**	**201**

General information

All manipulations of air and moisture sensitive species were performed under argon atmosphere unless otherwise stated. Dry solvents, where necessary, were dried by a MBRAUN MB-SPS-800 apparatus. Starting materials were acquired from commercial sources and used without further purification. Reactions were monitored by thin-layer chromatography (TLC) carried out on 0.25 mm E. Merck silica gel plates (60FS-254) using UV light for visualization. Silica gel grade 60 (230-400 mesh, Silicycle Inc.) was used for column chromatography. Melting points were measured in open capillary tubes on a Stuart Scientific SMP3 melting point apparatus and are uncorrected. ^{1}H, ^{13}C and ^{19}F NMR spectra were recorded in $CDCl_3$ (except otherwise indicated) on either a Varian Mercury VX-300, Varian Unity 300, Bruker Avance Neo 400 or Varian Unity 500 MHz spectrometers at room temperature. Chemical shifts are given in ppm (δ) downfield from TMS. Coupling constants (J) are in Hertz (Hz) and signals are described as follows: s, singlet; d, doublet; dd, doublet of doublets; dq, double of quadruplets; ddd, doublet of doublet of doublets; ddt, doublet of doublets of triplets; t, triplet; td, triplet of doublets, tt, triplets of triplets; q, quadruplet; m, multiplet. Absorption spectra were recorded in a UV-VIS FLS980 Spectrophotometer (Edinburgh Instruments) equipped with a detector (200-1000 nm) that is allowed for absorbance measurements. High-resolution analysis (HRMS) was performed on an Agilent 6545 Q-TOF. The irradiations were carried out with Kessil LED light A160WE Tuna Blue. Crystals for X-ray diffraction were grown through vapor diffusion crystallization, and the D8 VENTURE Dual-source configuration X-ray diffractometer was used for the measurements.

Reaction setup

The reaction was exposed to a Kessil 40 W blue LED lamp (model: A160WE Tuna Blue) positioned at approximately 3 cm from the Schlenk tube. A fan was placed on top of the Schlenk tube to maintain the temperature at 30°C.

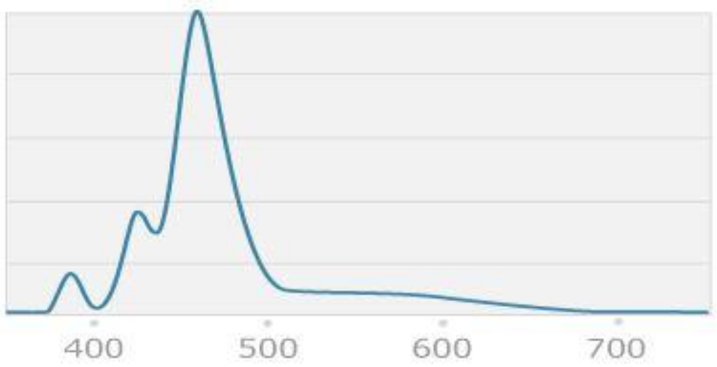

Supplementary Figure 1. Emission spectrum of the Kessil blue LED lamp.

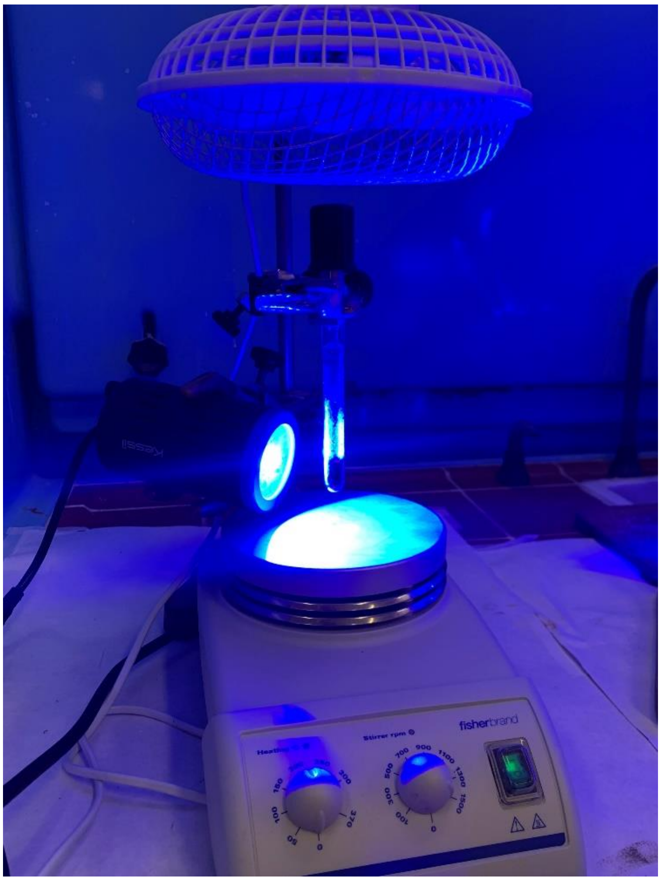

Supplementary Figure 2. Photochemical experimental setup.

Optimization

Supplementary Table 1. Optimization of the reaction conditions.

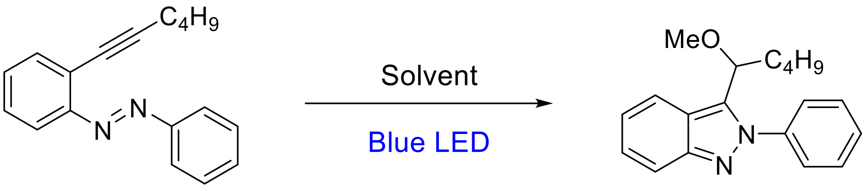

Entry	Blue LED (W)	Solvent	Time	Photocatalyst	Yield (%)
1	40	MeCN	24	-	-
2	40	DCE	24	-	-
3	**40**	**MeOH**	**24**	**-**	**95**
4[a]	15	MeOH	48	-	20
5[b]	40	MeOH	24	-	90

[a] 40% conversion. [b] Reaction conducted with 1mmol of **2a**.

Control experiments

Profile for (*E*)-1-[2-(Hex-1-yn-1-yl)phenyl]-2-phenyldiazene isomerization under the reaction conditions

To a flame dried J-Young cap NMR tube, (*E*)-1-(2-(hex-1-yn-1-yl)phenyl)-2-phenyldiazene (0.07 mmol) was added under a flow of argon. The NMR tube was evacuated and then filled back with argon (three times). Then, deuterated acetonitrile (0.1 M) was added. The NMR tube was placed in the photoreactor under blue light irradiation at room temperature. After the allotted time, the light was turned off and reaction solution was analyzed by ^{1}H-NMR. Temporal concentrations (%) of both isomers were calculated based on the integration of the corresponding signals and they were plotted against time in minutes. After 5 min a photostationary state was reached featuring a 65:35 ratio of *E* vs *Z* 1-[2-(hex-1-yn-1-yl)phenyl]-2-phenyldiazene.

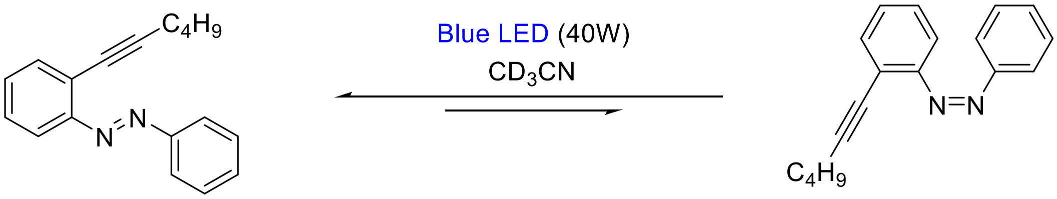

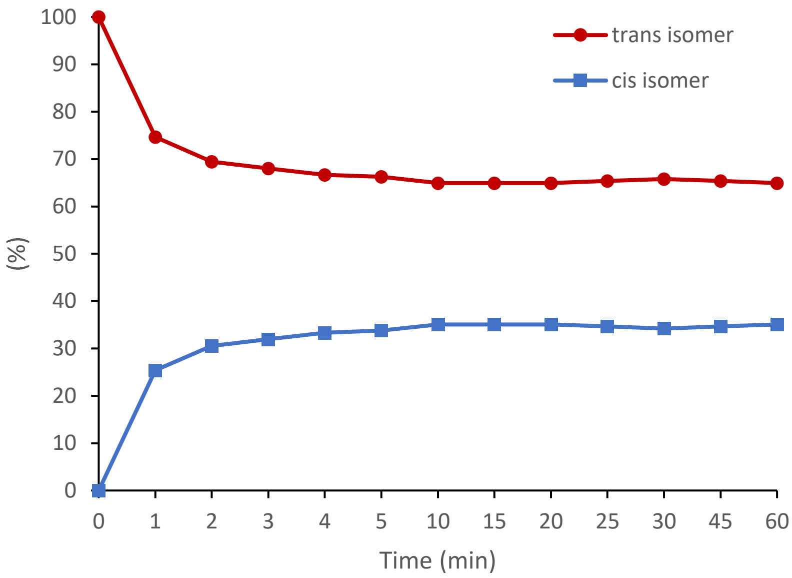

Supplementary Figure 3. Plotted isomerization profile of (*E*)-1-[2-(Hex-1-yn-1-yl)phenyl]-2-phenyldiazene).

Profile for (*Z*)-1-[2-(Hex-1-yn-1-yl)phenyl]-2-phenyldiazene isomerization under the reaction conditions

To a flame dried J-Young cap NMR tube, (*Z*)-1-(2-(hex-1-yn-1-yl)phenyl)-2-phenyldiazene (0.07 mmol) was added under a flow of argon. The NMR tube was evacuated and then filled back with argon (three times). Then, deuterated acetonitrile (0.1 M) was added. The NMR tube was placed in the photoreactor under blue light irradiation at room temperature. After the allotted time, the light was turned off and reaction solution was analyzed by ^{1}H-NMR. Temporal concentrations (%) of both isomers were calculated based on the integration of the corresponding signals and they were plotted against time in minutes. After 5 min a photostationary state was reached featuring a 65:35 ratio of *E vs Z* 1-[2-(hex-1-yn-1-yl)phenyl]-2-phenyldiazene.

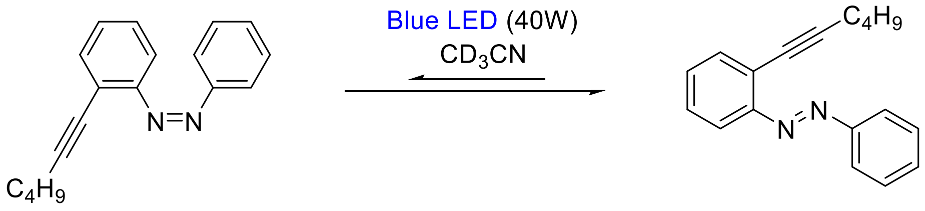

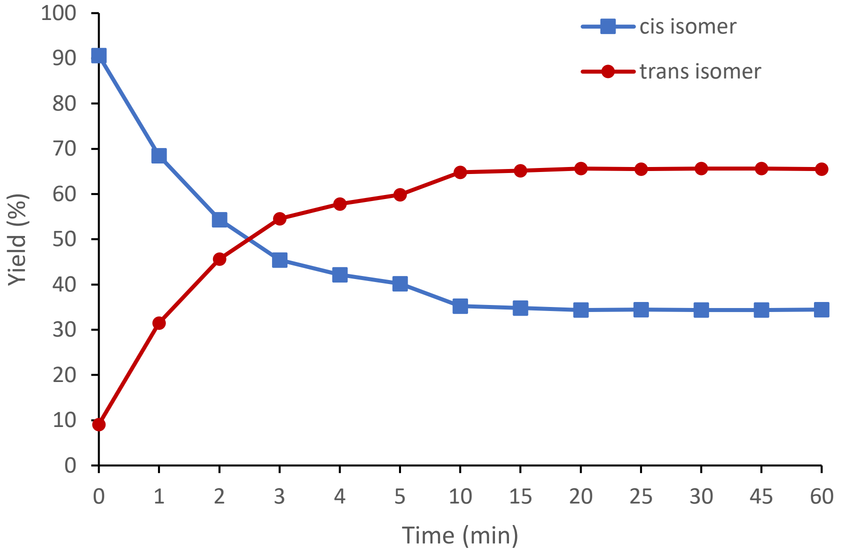

Supplementary Figure 4. Plotted isomerization profile of (*Z*)-1-[2-(Hex-1-yn-1-yl)phenyl]-2-phenyldiazene).

Deuterium labelling experiment

To a flame dried J-Young cap NMR tube, (*E*)-1-(2-(hex-1-yn-1-yl)phenyl)-2-phenyldiazene (0.07 mmol) was added under a flow of argon. The NMR tube was evacuated and then filled back with argon (three times). Then, deuterated methanol (0.1 M) was added. Time zero (t = 0) ^{1}H-NMR spectrum was recorded prior irradiation. Then the NMR tube was placed in the photoreactor under blue light irradiation (40 W) at room temperature. After the allotted time, the light was turned off and reaction solution was analyzed by ^{1}H-NMR. Temporal concentrations (%) of both isomers were calculated based on the integration of the corresponding signals and they were plotted against time in minutes.

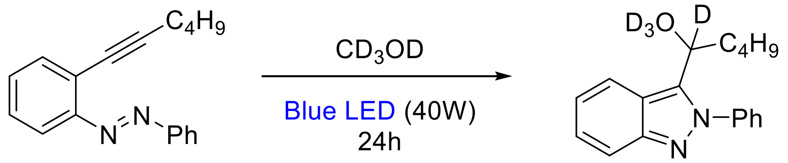

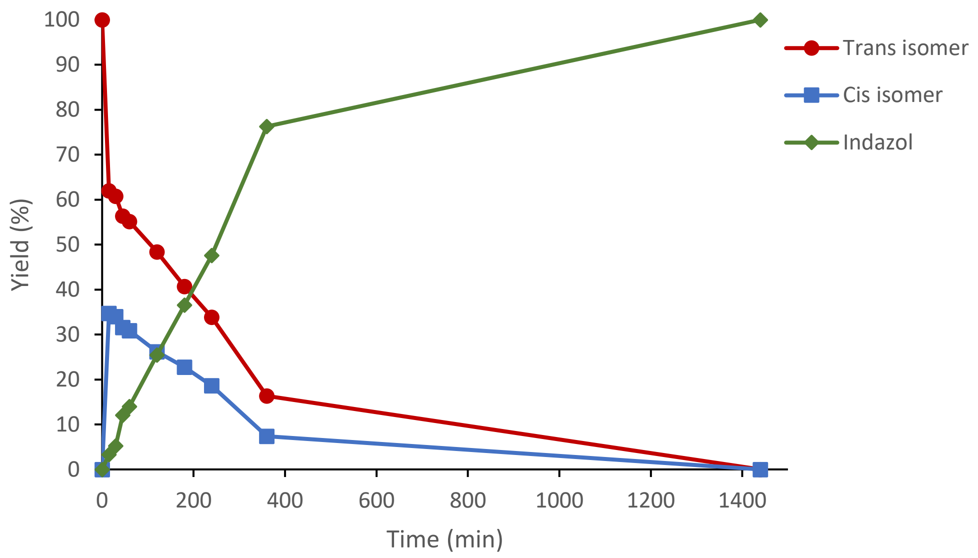

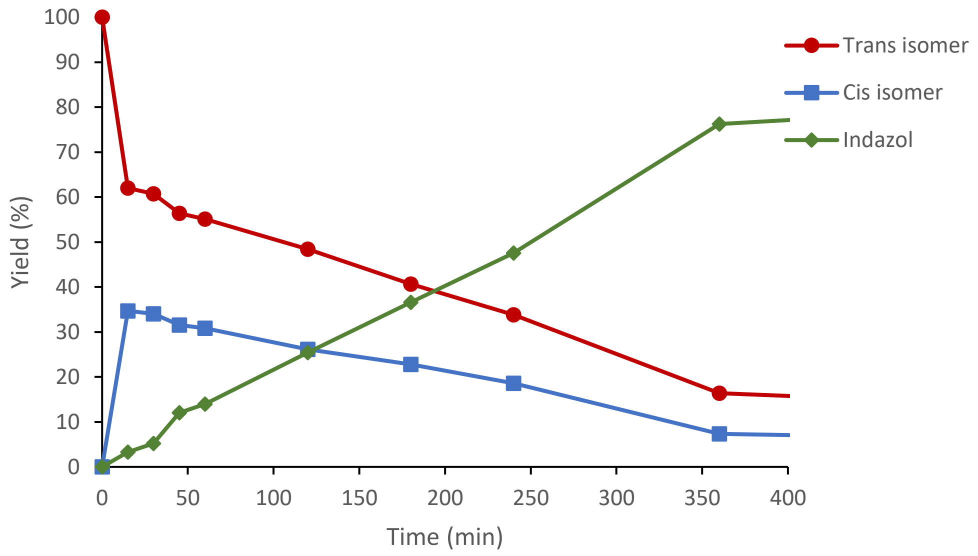

Supplementary Figure 5. Kinetics for the reaction of (*E*)-1-[2-(hex-1-yn-1-yl)phenyl]-2-phenyldiazene with deuterated methanol.

When deuterated methanol was used as the solvent in the reaction with (*E*)-1-(2-(hex-1-yn-1-yl)phenyl)-2-phenyldiazene, incorporation of a molecule of methanol was observed. **[*D*]-2a** was obtained in 92% yield with > 99% deuteration after 24 h.

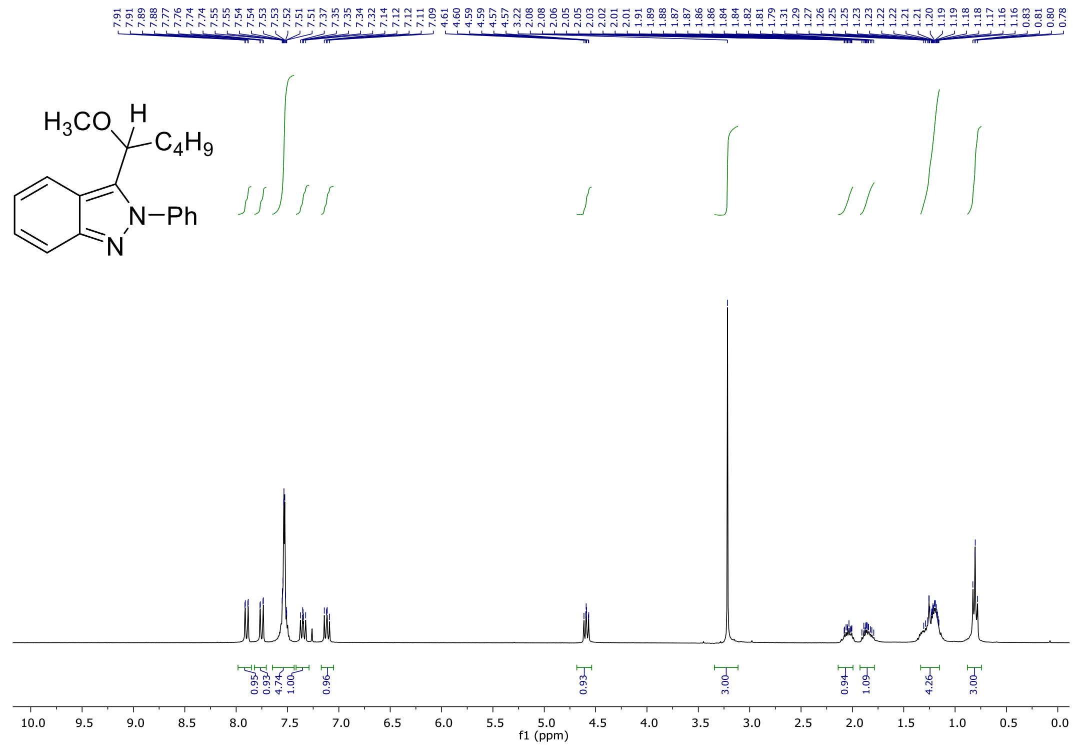

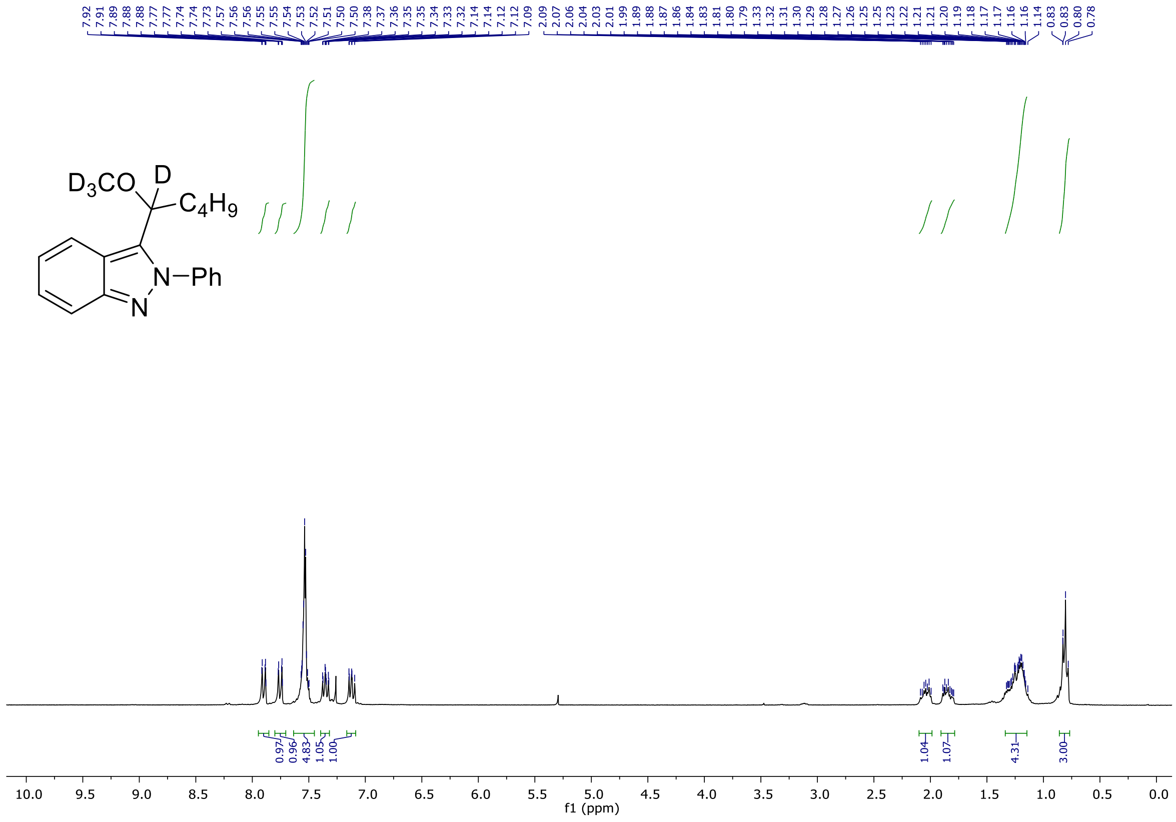

Experiment without irradiation

(*E*)-1-(2-(Hex-1-yn-1-yl)phenyl)-2-phenyldiazene (0.07 mmol) was added into a flame dried J-Young valved NMR tube under argon. Subsequently, deuterated methanol (0.1 M) and CH_2Cl_2 (1µL) as internal standard were added. The NMR tube was irradiated with blue light at room temperature for 10 minutes, until it reached the photostationary state. Afterward, the light source was turned off, and the reaction was allowed to proceed in darkness for an additional 24 hours. The reaction mixture was analyzed by ^{1}H-NMR, and the temporal concentrations (%) of both isomers and the 2*H*-indazol were calculated based on the integration of their respective signals. These data were then plotted against time in minutes. A similar profile, as shown in **Supplementary Figure 3** was obtained. During the initial 10 minutes of reaction, a partial isomerization of *E*-**1a** to *Z*-**1a** occurred, resulting in a 60% yield of *E*-**1a** and a 30% yield of *Z* -**1a**. Additionally, at this point, a 5% yield of **2a** was observed. However, once the blue light irradiation ceased, the reaction did not progress further, and no additional conversion to the 2*H*-indazole was observed.

Triplet Quenching Experiments

To a flame dried J-Young cap NMR tube, (*E*)-1-(2-(hex-1-yn-1-yl)phenyl)-2-phenyldiazene (0.06 mmol) was added under a flow of argon. The NMR tube was evacuated and then filled back with argon (three times). Then, piperylene (0.3 mmol) and deuterated methanol (0.1 M) were added. Time zero (t = 0) ^{1}H-NMR spectrum was recorded prior irradiation. Then the NMR tube was placed in the photoreactor under blue light irradiation (40 W) at room temperature. After the allotted time, the light was turned off and reaction solution was analyzed by ^{1}H-NMR. Temporal concentrations (%) of both isomers were calculated based on the integration of the corresponding signals and they were plotted against time in minutes.

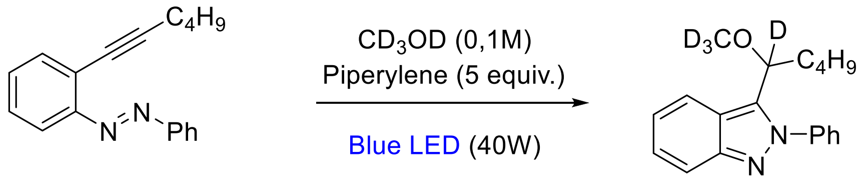

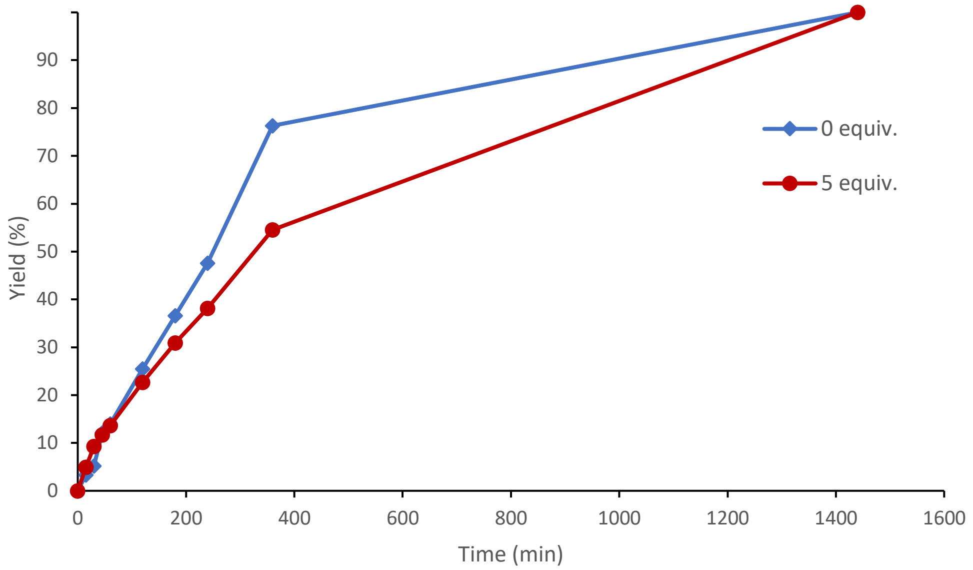

Supplementary Figure 6. Kinetics for the formation of 3-[1-(methoxy-d_3)pentyl-1-*d*]-2-phenyl-2*H*-indazole with and without piperylene.

The general procedure was followed adding the corresponding additive (1.0 mmol, 5 equiv.) in each case. After evaporation of the solvent and purification by flash chromatography yields were calculated. No significant effect in the yield was observed in the presence of radical inhibitor.

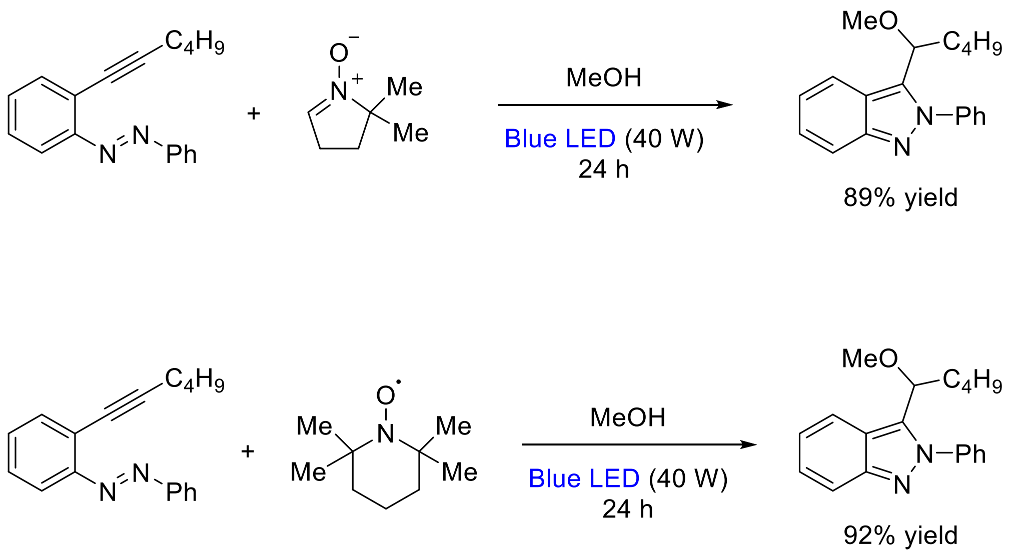

Supplementary Figure 7. Radical inhibitor experiments

No reaction was observed without irradiation with blue LED. When the reaction was carried out with (*E*)-**1a** only isomerization of double bond was observed. These results point out to that the reaction could start with the excitation of azo group. An additional experiment was carried out

by solely heating the reaction of **1a** in methanol at 70 ºC, without blue light irradiation. After 36 hours, only a 16% yield of **2a** was obtained by column chromatography.

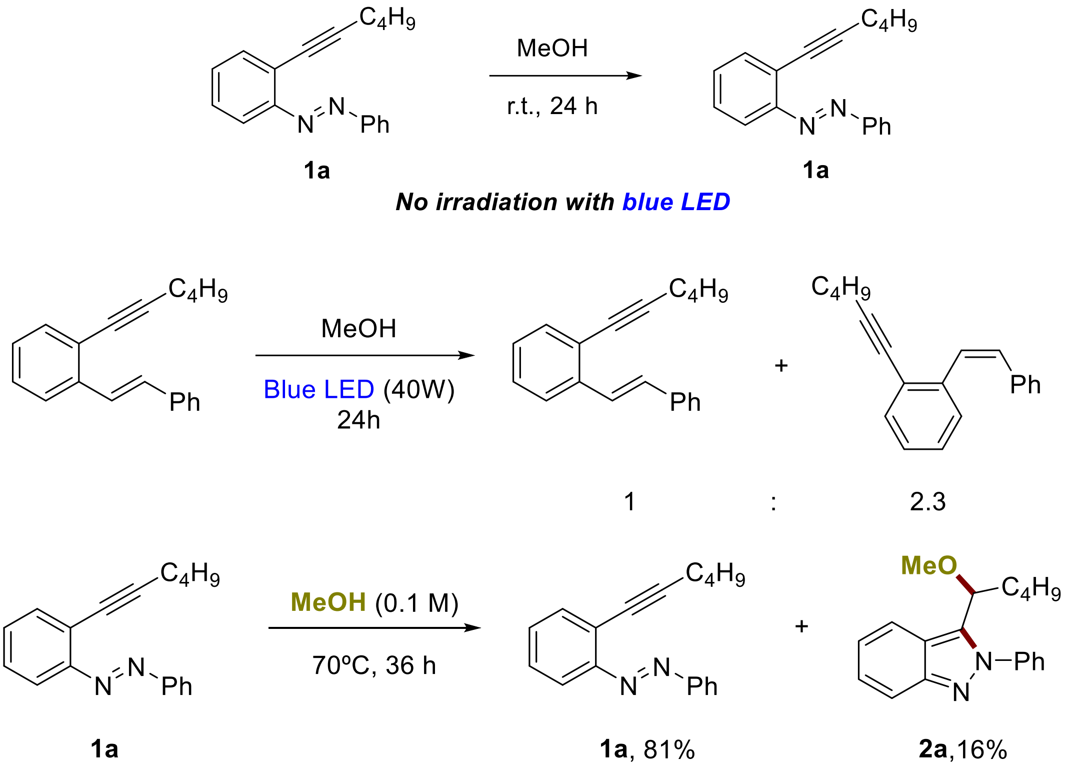

Supplementary Figure 8. Additional control experiments

The synthesis of the following substrates was attempted by Mills reaction or formation of diazonium salt but in no case was the formation of the corresponding substrate detected:

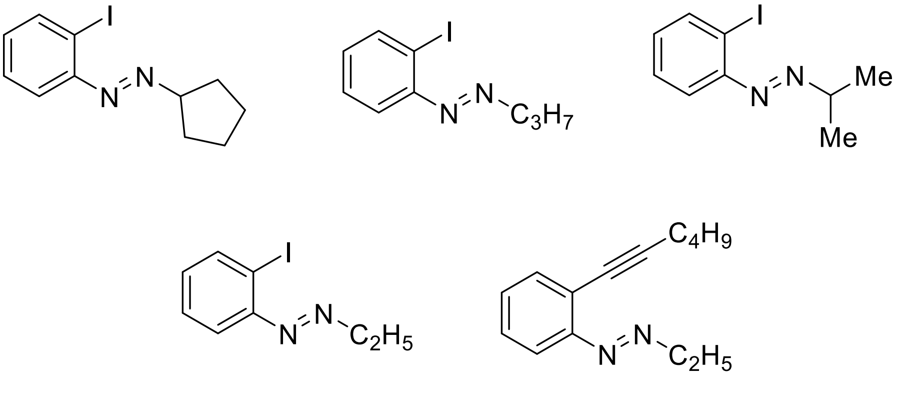

With the substrates depicted in Figure S9, we were unable to obtain the desired products using either the standard reaction conditions or with extended reaction times or increased quantities of the corresponding nucleophile. In general, a complex mixture of compounds was obtained, with (*E*)-3-(pent-1-en-1-yl)-2-phenyl-2*H*-indazole identified as the major product. As demonstrated in the cases where the reaction was carried out with tocopherol and estrone, it is likely that better results could be achieved by derivatizing the alcohols with 3-chloropropan-1-ol.

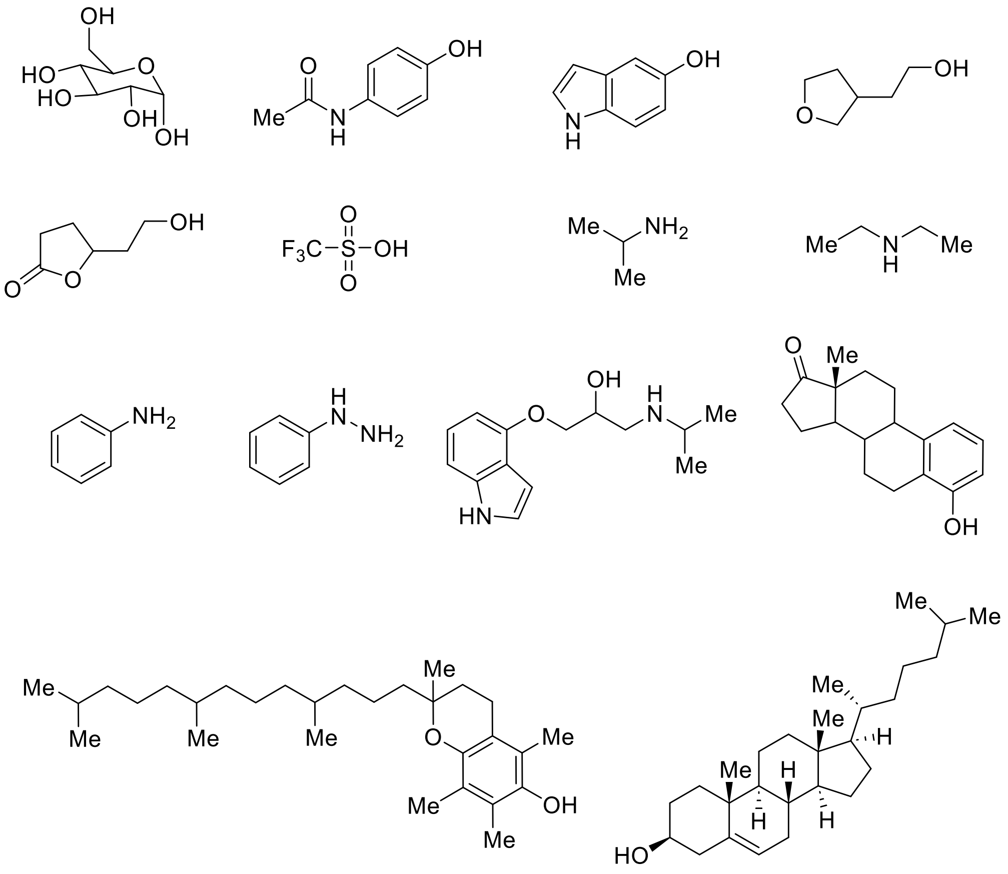

Supplementary Figure 9. Substrate limitations

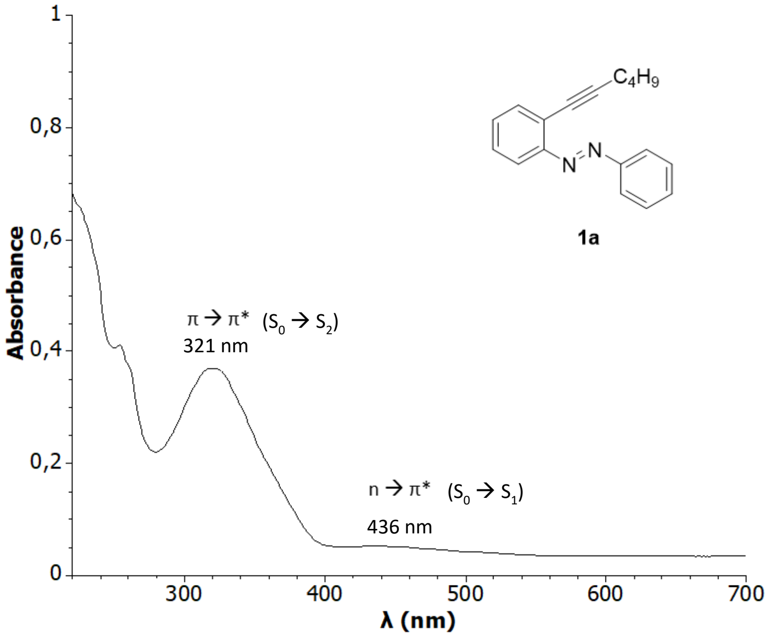

Supplementary Figure 10. UV-vis spectra of **1a**

The photoisomerization of azobenzenes has been extensively studied. The absorption spectrum of **1a** comprises two main bands in the UV-visible region (Figure S10). UV light (321 nm) induces E $\rightarrow$ Z isomerization via $\pi \rightarrow \pi^*$ excitation and corresponds with the transition $S_0 \rightarrow S_2$. The reverse photoisomerization to Z $\rightarrow$ E occurs by irradiation in the visible region (436 nm). This band correspond with the transition n $\rightarrow \pi^*$ excitation and correlates with the transition $S_0 \rightarrow S_1$.

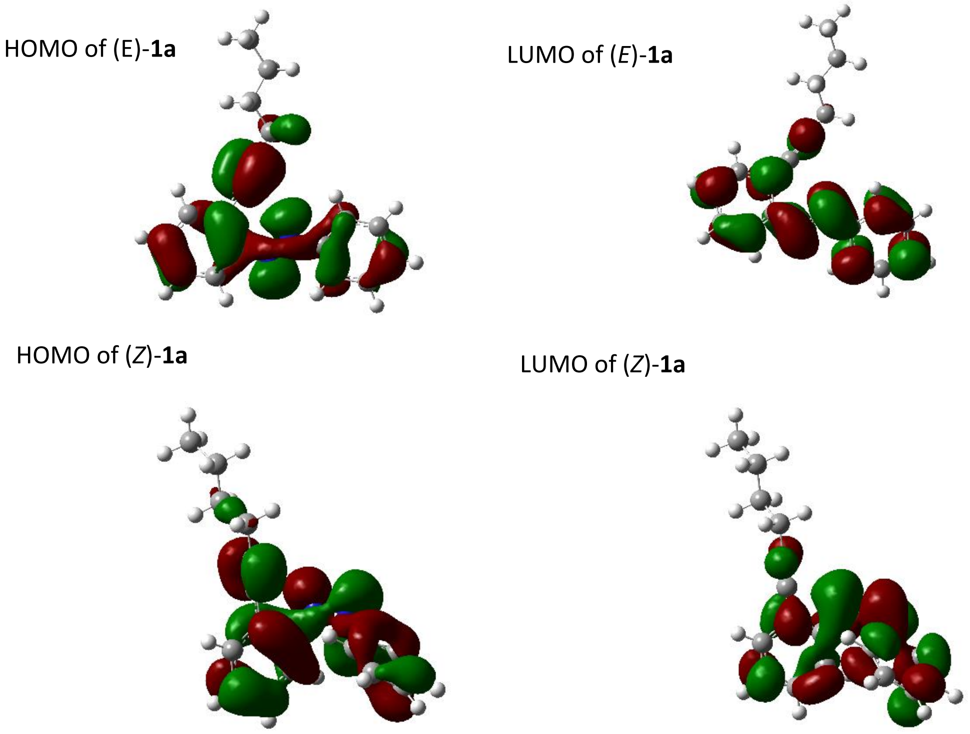

Supplementary Figure 11. HOMO and LUMO orbital of (*E*)- and (*Z*)-**1a**

The plots of HOMO and LUMO molecular orbitals for the isomers (*E*)-**1a** and (*Z*)-**1a** are shown in Figure S11. For both isomers, the HOMO orbitals are spread mainly over aromatic rings, azo group and alkyne moiety. In contrast, the LUMO orbitals are almost uniformly distributed over aromatic rings and azo group.

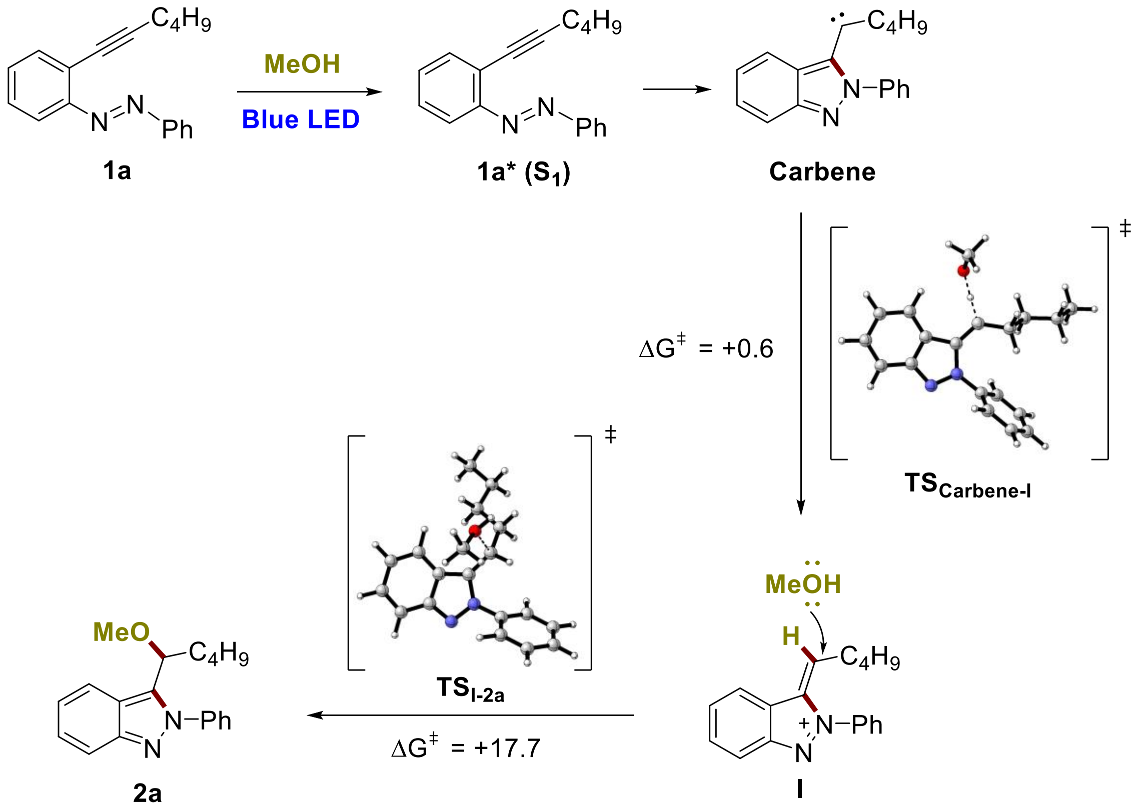

Supplementary Figure 12.

Intermediate **I** could potentially be generated from the corresponding carbene. DFT calculations were conducted, revealing that the transition state for the conversion from the carbene intermediate to **I** is +0.64 kcal/mol.

Procedures for the synthesis of alkynylazobenzenes (1a-1ac)

Preparation of anilines

Methyl 4-aminobenzoate[1]

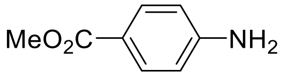

Eluent: hexane/EtOAc (50:1). Colorless oil, 42% yield.

^{1}H-NMR (300 MHz, $CDCl_3$) δ (ppm) δ 7.93–7.78 (m, 2H), 6.72–6.56 (m, 2H), 4.05 (s, 2H), 3.85 (s, 3H).

^{13}C-NMR (126 MHz, $CDCl_3$) δ (ppm) 167.3, 151.0, 131.7, 119.8, 113.9, 51.7.

2-(Hex-1-yn-1-yl)aniline[2]

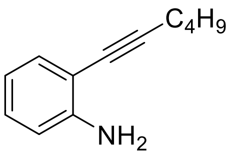

Eluent: hexane/EtOAc (50:1). Colorless oil, 77% yield.

^{1}H-NMR (300 MHz, $CDCl_3$) δ (ppm) 7.29–7.20 (m, 1H), 7.14–7.02 (m, 1H), 6.73–6.61 (m, 2H), 4.16 (s, 2H), 2.48 (td, *J* = 7.0, 1.2 Hz, 2H), 1.67–1.43 (m, 4H), 0.96 (t, *J* = 7.2 Hz, 3H).

^{13}C-NMR (126 MHz, $CDCl_3$) δ (ppm) 147.7, 132.2, 128.9, 118.0, 114.3, 109.1, 95.9, 77.1, 31.2, 22.2, 19.5, 13.8.

Preparation of alcohols

In a 5 mL round bottom flask equipped with a magnetic stirring bar, K_2CO_3 (1.28 mmol, 1.1 equiv.), DMF (2.3 mL, 0.5 M) and tocopherol or estrone (1.16 mmol, 1.0 equiv.) were sequentially added. The mixture was stirred in an oil bath at 60 ^oC for 30 min and then 3-chloro-1-propanol was added. After 16 h, the reaction was cooled down to room temperature, diluted with DCM (10 mL) and washed with HCl 1M (2 x 10 mL). The organic layer was dried over Mg_2SO_4, filtered and concentrated *in vacuo* to afford the desired product, which was further purified by flash chromatography using a mixture hexane: EtOAc.

3-[(2,5,7,8-Tetramethyl-2-(4,8,12-trimethyltridecyl)chroman-6-yl)oxy]propan-1-ol[3]

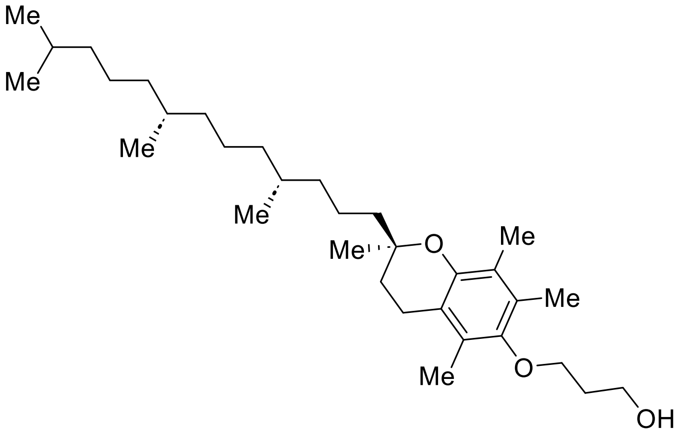

Eluent: hexane/EtOAc, 5:1. Yellow oil, 35% yield.

^{1}H-NMR (400 MHz, $CDCl_3$) δ (ppm) 3.97 (t, *J* = 5.7 Hz, 2H), 3.85 (t, *J* = 5.8 Hz, 2H), 2.59 (t, *J* = 6.8 Hz, 2H), 2.24–2.10 (m, 9H), 2.06 (t, *J* = 5.8 Hz, 3H), 1.90–1.71 (m, 2H), 1.59–1.08 (m, 24H), 0.94–0.82 (m, 12H).

^{13}C-NMR (101 MHz, $CDCl_3$) δ (ppm) 148.1, 127.8, 125.8, 123.1, 117.7, 74.9, 71.9, 61.9, 40.2, 40.1, 39.5, 37.7, 37.7, 37.6, 37.6, 37.5, 37.5, 37.5, 37.5, 37.4, 32.9, 32.9, 32.8, 32.8, 32.8, 31.4, 31.4, 28.1, 24.9, 24.9, 24.6, 23.9, 22.9, 22.8, 22.8, 21.2, 21.1, 20.8, 19.9, 19.8, 19.8, 19.8, 19.7, 12.8, 11.9, 11.9.

HRMS (ESI+) m/z calculated for $C_{32}H_{57}O_3$ $[M+H]^+$: 489.4302, found $[M+H]^+$: 489.4310.

(8*R*,9*S*,13*S*,14*S*)-4-(3-Hydroxypropoxy)-13-methyl-6,7,8,9,11,12,13,14,15,16-decahydro-17H-cyclopenta[a]phenanthren-17-one[3]

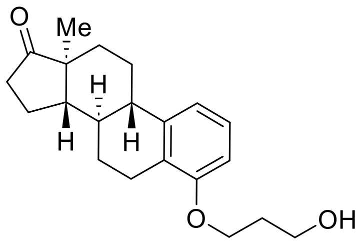

Eluent: hexane/EtOAc, 5:1. White solid, 59% yield, m.p. = 76-77 ^{o}C.

^{1}H-NMR (300 MHz, $CDCl_3$) δ (ppm) δ 7.11 (dd, *J* = 8.6, 1.1 Hz, 1H), 6.64 (dd, *J* = 8.6, 2.8 Hz, 1H), 6.58 (d, *J* = 2.7 Hz, 1H), 4.02 (t, *J* = 6.0 Hz, 2H), 3.76 (t, *J* = 5.9 Hz, 2H), 2.88–2.75 (m, 2H), 2.48–2.36 (m, 1H), 2.32–2.28 (m, 1H), 2.18–1.93 (m, 6H), 1.89–1.83 (m, 1H), 1.62–1.27 (m, 6H), 1.18 (t, *J* = 7.2 Hz, 1H), 0.82 (s, 3H).

^{13}C-NMR (101 MHz, $CDCl_3$) δ (ppm) 156.9, 137.8, 132.3, 126.4, 114.6, 112.2, 65.8, 60.5, 50.5, 48.1, 44.0, 38.4, 35.9, 32.1, 31.6, 29.7, 26.6, 26.0 21.6, 13.9.

HRMS (ESI+) m/z calculated for $C_{21}H_{29}O_3$ $[M+H]^+$: 329.2111, found $[M+H]^+$: 329.2114.

General procedure for the synthesis of 1-(2-iodophenyl)-2-phenyldiazenes

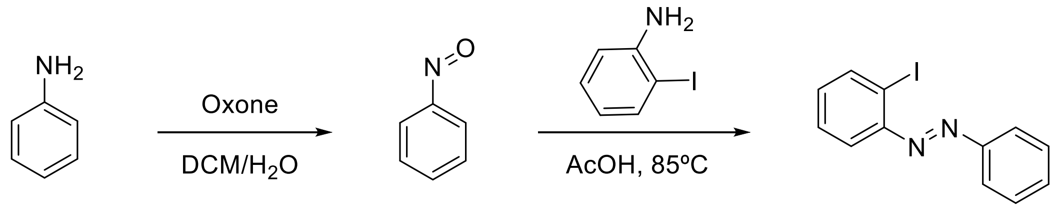

Method A

In a round bottom flask the corresponding aniline (20.0 mmol) was dissolved in DCM (0.4 M). A solution of Oxone (24.0 mmol, 1.2 equiv.) in H_2O (0.4 M) was added slowly to the aniline, and the mixture was stirred at room temperature until completion. The mixture was quenched with H_2O and extracted with DCM (3 x 60 mL). The organic phases were combined, dried over Mg_2SO_4, filtered, and concentrated *in vacuo* to afford the corresponding nitrosobenzene that was used directly in the next step without further purification.

The appropriate 2-iodoaniline was dissolved in AcOH (0.1 M) and the corresponding nitrosobenzene (30.0 mmol, 1.5 equiv.) was added. The reaction was stirred in an oil bath at 85 ^{0}C until completion. The crude was cooled down to room temperature, diluted with EtOAc and washed with brine (3 x 100 mL) and H_2O (2 x 100 mL). The organic layer was dried over Mg_2SO_4 and filtered. The solvents were removed under reduced pressure and the crude was purified by column chromatography using a mixture of hexane and ethyl acetate as eluent to yield the corresponding 1-(2-iodophenyl)-2-phenyldiazene.

(*E*)-1-(2-Iodophenyl)-2-phenyldiazene[4]

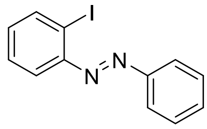

It was prepared following the general method A from nitrosobenzene (69% yield) and 2-iodoaniline. Eluent: hexane. Red solid, 65% yield, m.p. = 59-60 ^{0}C.

^{1}H-NMR (300 MHz, $CDCl_3$) δ (ppm) 8.11–7.94 (m, 3H), 7.65 (dd, *J* = 8.0, 1.6 Hz, 1H), 7.59–7.48 (m, 3H), 7.43 (ddd, *J* = 8.1, 7.2, 1.3 Hz, 1H), 7.17 (ddd, *J* = 7.9, 7.2, 1.7 Hz, 1H).

^{13}C-NMR (75 MHz, $CDCl_3$) δ (ppm) 152.4, 151.4, 134.0 132.3, 131.7, 129.3, 129.1, 123.7, 117.5, 102.7.

HRMS (ESI+) m/z calculated for $C_{12}H_9IN_2Na$ $[M+Na]^+$: 330.9703, found $[M+Na]^+$: 330.9705.

(*E*)-1-(4-Bromophenyl)-2-(2-iodophenyl)diazene

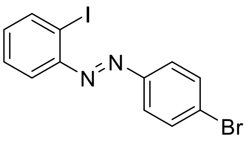

It was prepared following the general method A from 1-bromo-4-nitrosobenzene (80% yield) and 2-iodoaniline. Eluent: hexane. Red solid, 65% yield, m.p. = 94-95 ^oC.

^{1}H-NMR (300 MHz, $CDCl_3$) δ (ppm) 8.04 (dt, *J* = 7.9, 1.0 Hz, 1H), 7.94–7.80 (m, 2H), 7.70–7.59 (m, 3H), 7.40–7.46 (m, 1H), 7.21–7.18 (m, 1H).

^{13}C-NMR (75 MHz, $CDCl_3$) δ (ppm) 151.0, 140.0, 132.6, 132.5, 132.5, 129.0, 126.2, 125.0, 117.3, 102.9.

HRMS (ESI+) m/z calculated for $C_{12}H_9BrIN_2$ $[M+H]^+$: 386.8988, found $[M+H]^+$: 386.8989.

(*E*)-1-(3-Bromophenyl)-2-(2-iodophenyl)diazene

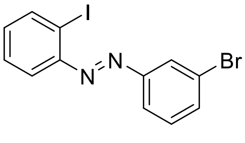

It was prepared following the general method A from 1-bromo-3-nitrosobenzene (54% yield) and 2-iodoaniline. Eluent: hexane. Red solid, 57% yield, m.p. = 115-116 ^oC.

^{1}H-NMR (300 MHz, $CDCl_3$) δ (ppm) 8.12 (t, *J* = 1.9 Hz, 1H), 8.04 (dd, *J* = 7.9, 1.3 Hz, 1H), 7.94 (ddd, *J* = 7.9, 1.8, 1.0 Hz, 1H), 7.63 (dd, *J* = 8.0, 1.3 Hz, 2H), 7.49–7.36 (m, 2H), 7.19 (ddd, *J* = 7.9, 7.2, 1.6 Hz, 1H).

^{13}C-NMR (75 MHz, $CDCl_3$) δ (ppm) 153.2, 151.1, 140.1, 134.3, 132.9, 130.7, 129.1, 125.9, 123.3, 123.3, 117.5, 103.2.

HRMS (ESI+) m/z calculated for $C_{12}H_9BrIN_2$ $[M+H]^+$: 386.8988, found $[M+H]^+$: 386.8988.

(*E*)-1-(3-Fluorophenyl)-2-(2-iodophenyl)diazene

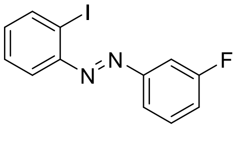

It was prepared following the general method A from 1-fluoro-3-nitrosobenzene (25% yield) and 2-iodoaniline. Eluent: hexane. Red solid, (51% yield), m.p. = 76-77 ^oC.

^{1}H-NMR (300 MHz, $CDCl_3$) δ (ppm) 8.03 (dt, *J* = 7.9, 0.9 Hz, 1H), 7.84–7.80 (m, 1H), 7.74–7.57 (m, 2H), 7.55–7.38 (m, 2H), 7.27–7.13 (m, 2H).

^{19}F-NMR (282 MHz, $CDCl_3$) δ (ppm) -111.89 – -112.10 (m).

^{13}C-NMR (75 MHz, CDCl$_3$) δ (ppm) 164.9, 161.6, 150.9, 140.0, 132.7, 130.4 (d, *J* = 8.3 Hz), 129.0, 121.3 (d, *J* = 2.9 Hz), 118.4 (d, *J* = 22.0 Hz), 117.4, 108.7 (d, *J* = 23.0 Hz), 103.1.

HRMS (ESI+) m/z calculated for $C_{12}H_9FIN_2$ [M+H]$^+$: 326.9789, found [M+H]$^+$: 326.9799.

(*E*)-1-(2-Fluorophenyl)-2-(2-iodophenyl)diazene

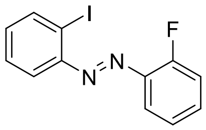

It was prepared following the general method A from 1-fluoro-2-nitrosobenzene (31% yield) and 2-iodoaniline. Eluent: hexane. Red solid, 50% yield, m.p. = 81-82 ^{o}C.

^{1}H-NMR (300 MHz, CDCl$_3$) δ (ppm) 8.02 (dt, *J* = 7.9, 1.0 Hz, 1H), 7.90–7.83 (m, 1H), 7.67 (dt, *J* = 8.0, 1.2 Hz, 1H), 7.52–7.38 (m, 2H), 7.31–7.13 (m, 3H).

^{19}F-NMR (282 MHz, CDCl$_3$) δ (ppm)-124.04 (dd, *J* = 11.3, 5.7 Hz).

^{13}C-NMR (75 MHz, CDCl$_3$) δ (ppm) 162.2, 158.8, 151.4, 140.0, 133.3 (d, *J* = 8.3 Hz), 132.7, 129.0, 124.5 (d, *J* = 3.8 Hz), 118.4, 117.9, 117.2 (d, *J* = 19.8 Hz), 103.1.

HRMS (ESI+) m/z calculated for $C_{12}H_9FIN_2$ [M+H]$^+$: 326.9789, found [M+H]$^+$: 326.9795.

(*E*)-4-[(2-iodophenyl)diazenyl]benzonitrile

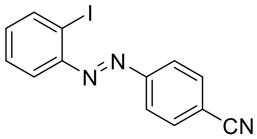

It was prepared following the general method A from 4-nitrosobenzonitrile (90% yield) and 2-iodoaniline. Eluent: hexane/EtOAc, 100:1. Orange solid, 83% yield, m.p. = 134-135 ^{o}C.

^{1}H-NMR (300 MHz, CDCl$_3$) δ (ppm) 8.15–7.98 (m, 3H), 7.89–7.78 (m, 2H), 7.66 (dd, *J* = 8.0, 1.6 Hz, 1H), 7.44 (td, *J* = 7.8, 1.3 Hz, 1H), 7.26–7.17 (m, 1H).

^{13}C-NMR (75 MHz, CDCl$_3$) δ (ppm) 153.9, 150.9, 140.2, 133.4, 133.3, 129.0, 124.0, 118.4, 117.3, 114.5, 104.0.

HRMS (ESI+) m/z calculated for $C_{13}H_9IN_3O$ [M+H]$^+$:333.9836, found [M+H]$^+$:333.9837.

(*E*)-1-(2-Iodophenyl)-2-(3-nitrophenyl)diazene

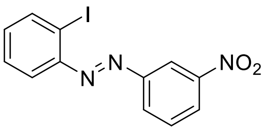

It was prepared following the general method A from 1-nitro-3-nitrosobenzene (92% yield) and 2-iodoaniline. Eluent: hexane/EtOAc, 100:1. Orange solid, 62% yield, m.p. = 125-126 ^{o}C.

^{1}H-NMR (400 MHz, CDCl$_3$) δ (ppm) 8.80 (t, *J* = 2.1 Hz, 1H), 8.36 (ddd, *J* = 8.1, 2.3, 1.1 Hz, 1H), 8.31 (ddd, *J* = 7.9, 1.9, 1.1 Hz, 1H), 8.07 (dd, *J* = 7.9, 1.3 Hz, 1H), 7.74 (d, *J* = 8.1 Hz, 1H), 7.68 (dd, *J* = 8.0, 1.6 Hz, 1H), 7.46 (ddd, *J* = 8.1, 7.2, 1.3 Hz, 1H), 7.26–7.20 (m, 1H).

^{13}C-NMR (101 MHz, CDCl$_3$) δ (ppm) 152.7, 151.0, 149.2, 140.3, 133.5, 130.2, 129.2, 128.9, 125.6, 118.8, 117.5, 103.8.

HRMS (ESI+) m/z calculated for $C_{12}H_9IN_3O_3$ [M+H]$^+$: 353.9734, found [M+H]$^+$: 353.9734.

(*E*)-1-[4-((2-Iodophenyl)diazenyl)phenyl]ethan-1-one

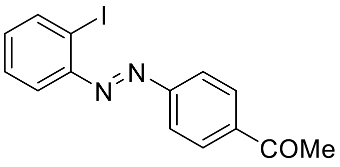

It was prepared following the general method A from 1-(4-nitrosophenyl)-1-ethanone (87% yield) and 2-iodoaniline. Eluent: hexane. Red solid, 20% yield, m.p. = 112-113 ^{o}C.

^{1}H-NMR (300 MHz, CDCl$_3$) δ (ppm) 8.16–7.98 (m, 5H), 7.65 (ddd, *J* = 8.1, 1.6, 0.7 Hz, 1H), 7.43 (ddt, *J* = 8.6, 7.2, 1.0 Hz, 1H), 7.24–7.13 (m, 1H), 2.66 (s, 3H).

^{13}C-NMR (75 MHz, CDCl$_3$) δ (ppm) 197.5, 154.6, 151.2, 140.1, 138.1, 133.1, 129.5, 129.1, 123.7, 117.4, 103.6, 27.0.

HRMS (ESI+) m/z calculated for $C_{14}H_{12}IN_2O$ [M+H]$^+$: 350.9989, found [M+H]$^+$: 350.9999.

Methyl (*E*)-4-[(2-iodophenyl)diazenyl]benzoate

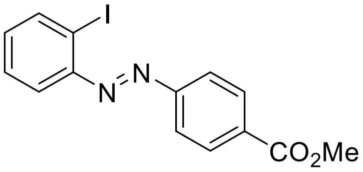

It was prepared following the general method A from methyl-4-nitrosobenzoate (91% yield) and 2-iodoaniline. Eluent: hexane. Red solid, 41% yield, m.p. = 157-158 ^{o}C.

^{1}H-NMR (500 MHz, CDCl$_3$) δ (ppm) 8.23–8.17 (m, 2H), 8.07–7.99 (m, 3H), 7.65 (dd, *J* = 8.0, 1.6 Hz, 1H), 7.43 (ddd, *J* = 8.1, 7.2, 1.3 Hz, 1H), 7.19 (ddd, *J* = 7.9, 7.2, 1.6 Hz, 1H), 3.96 (s, 3H).

^{13}C-NMR (75 MHz, CDCl$_3$) δ (ppm) 166.4, 154.6, 151.2, 140.1, 132.9, 132.3, 130.7, 129.0, 123.4, 117.3, 103.4, 52.6.

HRMS (ESI+) m/z calculated for $C_{14}H_{12}IN_2O_2$ [M+H]$^+$: 366.9938, found [M+H]$^+$:366.9945.

(*E*)-1-(2-Bromo-4-methylphenyl)-2-(2-iodophenyl)diazene

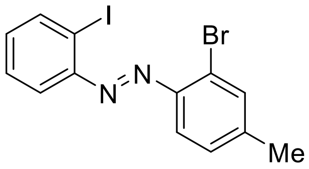

It was prepared following the general method A from 3-bromo-1-methyl-4-nitrosobenzene (79% yield) and 2-iodoaniline. Eluent: hexane. Red solid, 88% yield, m.p. = 106-107 ^oC.

^{1}H-NMR (300 MHz, $CDCl_3$) δ (ppm) 8.06–7.97 (m, 1H), 7.78–7.67 (m, 2H), 7.58 (dt, *J* = 1.7, 0.8 Hz, 1H), 7.46–7.40 (m, 1H), 7.24–7.11 (m, 2H), 2.42 (s, 3H).

^{13}C-NMR (75 MHz, $CDCl_3$) δ (ppm) 151.2, 147.1, 143.7, 139.8, 134.1, 132.5, 129.0, 129.0, 126.7, 118.3, 118.1, 103.0, 21.5.

HRMS (ESI+) m/z calculated for $C_{13}H_{11}BrIN_2$ $[M+H]^+$: 400.9145, found $[M+H]^+$: 400.9151.

Dimethyl (*E*)-5-[(2-iodophenyl)diazenyl]isophthalate

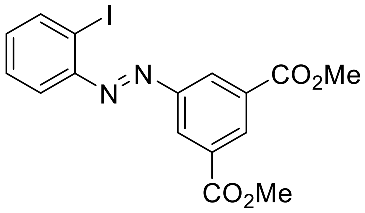

It was prepared following the general method A from dimethyl 5-nitrosoisophthalate (93% yield) and 2-iodoaniline. Eluent: hexane. Red solid, 45% yield, m.p. = 132-133 ^oC.

^{1}H-NMR (400 MHz, $CDCl_3$) δ (ppm) 8.84–8.78 (m, 3H), 8.07 (dd, *J* = 7.9, 1.3 Hz, 1H), 7.67 (dd, *J* = 8.0, 1.6 Hz, 1H), 7.45 (ddd, *J* = 8.0, 7.2, 1.3 Hz, 1H), 7.22 (ddd, *J* = 7.9, 7.2, 1.6 Hz, 1H), 4.01 (s, 6H).

^{13}C-NMR (101 MHz, $CDCl_3$) δ (ppm) 165.8, 152.5, 151.2, 140.3, 133.2, 132.9, 132.0, 129.1, 128.5, 117.5, 103.4, 52.8.

HRMS (ESI+) m/z calculated for $C_{16}H_{14}IN_2O_4$ $[M+H]^+$: 424.9993, found $[M+H]^+$: 424.9985.

(*E*)-1-(2-Iodophenyl)-2-(*p*-tolyl)diazene

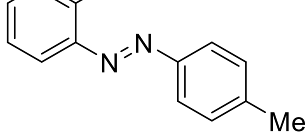

It was prepared following the general method A from 1-methyl-4-nitrosobenzene (45% yield) and 2-iodoaniline. Eluent: hexane. Red solid, 30% yield, m.p. = 81-82 ^oC.

^{1}H-NMR (400 MHz, $CDCl_3$) δ (ppm) 8.02 (dd, *J* = 7.9, 1.3 Hz, 1H), 7.94–7.87 (m, 2H), 7.62 (dd, *J* = 8.0, 1.6 Hz, 1H), 7.42 (ddd, *J* = 8.1, 7.2, 1.3 Hz, 1H), 7.37–7.30 (m, 2H), 7.15 (ddd, *J* = 7.9, 7.2, 1.6 Hz, 1H), 2.45 (s, 3H).

^{13}C-NMR (101 MHz, $CDCl_3$) δ (ppm) 151.6, 150.7, 142.4, 139.9, 132.0, 130.0, 129.0, 123.7, 117.5, 102.4, 21.7.

HRMS (ESI+) m/z calculated for $C_{13}H_{12}IN_2$ $[M+H]^+$: 323.0040, found $[M+H]^+$: 323.0046.

Method B

(*E*)-*N*-[4-((2-iodophenyl)diazenyl)phenyl]-*N*-methylacetamide

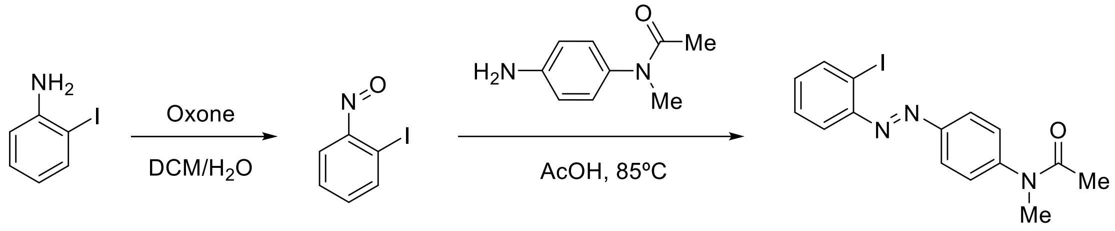

In a 250 mL round bottom flask, 2-iodoaniline (10.0 mmol, 1.0 equiv.) was dissolved in DCM (0.4 M). A solution of Oxone (15.0 mmol, 1.5 equiv.) in H_2O (0.4 M) was added slowly, and the mixture was stirred at room temperature. After 1 h, the mixture was quenched with H_2O and extracted with DCM (3 x 100 mL). The organic phases were combined, dried over Mg_2SO_4, filtered and concentrated *in vacuo* to afford 1-iodo-2-nitrosobenzene which was used in the next step without further purification.

N-(4-aminophenyl)-*N*-methylacetamide was dissolved in AcOH (0.1 M) and 1-iodo-2-nitrosobenzene (1.5 equiv.) was added. The reaction was stirred in an oil bath at 85 ^{o}C overnight. The crude was cooled down to room temperature, diluted with EtOAc and washed with brine (3 x 30 mL) and H_2O (2 x 30 mL). The combined organic phases were dried over Mg_2SO_4, filtered and concentrated in vacuo to afford the desired product, which was further purified by flash chromatography using a mixture hexane/EtOAc (5:1) as eluent in 52% yield.

^{1}H-NMR (300 MHz, $CDCl_3$) δ (ppm) 8.05 (dd, *J* = 8.2, 1.5 Hz, 3H), 7.65 (dd, *J* = 8.1, 1.6 Hz, 1H), 7.50–7.40 (m, 1H), 7.40–7.32 (m, 2H), 7.25–7.14 (m, 1H), 3.34 (s, 3H), 1.99 (s, 3H).

^{13}C-NMR (75 MHz, $CDCl_3$) δ (ppm) 170.5, 151.3, 147.3, 140.1, 132.7, 129.1, 127.9, 124.9, 117.4, 103.1, 37.4, 22.8.

HRMS (ESI+) m/z calculated for $C_{15}H_{15}IN_3O$ $[M+H]^+$: 380.0254, found $[M+H]^+$: 380.0245.

Method C

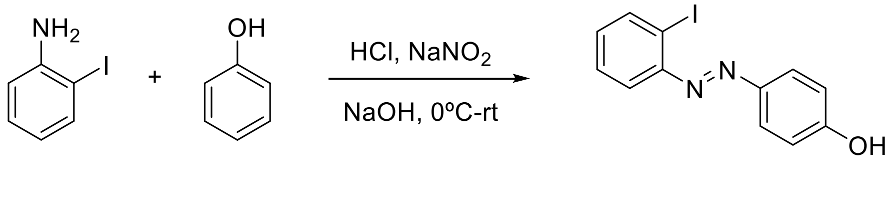

2-iodoaniline (18.3 mmol, 1.0 equiv.) was dissolved in H_2O (0.1 M) and a solution of $NaNO_2$ (20.1 mmol, 1.1 equiv.) was added dropwise at 0 ^oC. The mixture was stirred for 15 min, and it was added over a solution of phenol (20.1 mmol, 1.1 equiv.). The reaction was stirred at room temperature until completion. HCl was added until pH = 7, and the mixture was filtered over a pad of Celite and extracted with $CHCl_3$ (3 x 100 mL). The combined organic layers were dried over $MgSO_4$, filtered and concentrated *in vacuo*. The crude was purified by flash chromatography using a mixture hexane:EtOAc to afford the corresponding 1-(2-iodophenyl)-2-phenyldiazene.

(*E*)-4-[(2-Iodophenyl)diazenyl]phenol

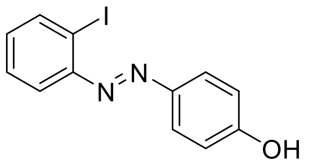

Eluent hexane:EtOAc (5:1). It was prepared following the general method C. Red oil, 51% yield.

^{1}H-NMR (300 MHz, $CDCl_3$) δ (ppm) 8.12–7.86 (m, 3H), 7.60 (dd, *J* = 8.0, 1.6 Hz, 1H), 7.44–7.38 (m, 1H), 7.16–7.10 (m, 1H), 7.03–6.87 (m, 2H).

^{13}C-NMR (75 MHz, $CDCl_3$) δ (ppm) 158.9, 151.4, 147.0, 139.8, 131.8, 129.0, 125.9, 117.4, 116.1, 101.9.

HRMS (ESI+) m/z calculated for $C_{12}H_{10}IN_2O$ $[M+H]^+$: 324.9832, found $[M+H]^+$: 324.9837.

(*E*)-1-(2-Iodophenyl)-2-(4-methoxyphenyl)diazene

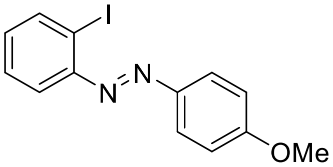

Powder KOH (21.5 mmol, 5 equiv.) was dissolved in DMSO (0.4 M), then (*E*)-4-[(2-iodophenyl)diazenyl]phenol and iodomethane (11.0 mmol, 2.4 equiv.) were added and the mixture was stirred at room temperature. After 3 h, the crude was poured into water (10 mL), extracted with DCM (2 x 20 mL) and washed with water (3 x 20 mL). The combined organic phases were dried over Mg_2SO_4, filtered and concentrated in vacuo to afford the desired product, which was further purified by flash chromatography using a mixture hexane/EtOAc (50:1) a red solid with 63% yield.

^{1}H-NMR (300 MHz, $CDCl_3$) δ (ppm) 8.04–8.00 (m, 3H), 7.63 (dd, *J* = 8.1, 1.6 Hz, 1H), 7.41 (ddd, *J* = 8.2, 7.2, 1.2 Hz, 1H), 7.12 (ddd, *J* = 7.9, 7.1, 1.6 Hz, 1H), 7.08–6.98 (m, 2H), 3.88 (s, 3H).

^{13}C-NMR (75 MHz, $CDCl_3$) δ (ppm) 162.6, 151.4, 146.9, 139.8, 131.7, 128.9, 125.6, 117.3, 114.4, 102.3, 55.7.

HRMS (ESI+) m/z calculated for $C_{13}H_{11}IN_2NaO$ $[M+Na]^+$: 360.9808, found $[M+Na]^+$: 360.9804.

(*E*)-4-[(2-Iodophenyl)diazenyl]phenyl acetate

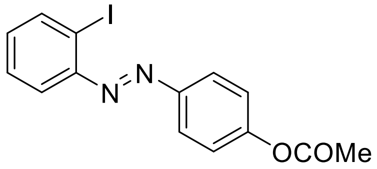

Et_3N (1.1 mmol, 1.1 equiv.) was added over a solution of (*E*)-4-[(2-iodophenyl)diazenyl]phenol in anhydrous THF (0.1 M), acetyl chloride (2.0 mmol, 2.0 equiv.) was then added and the reaction was refluxed overnight. The solvent was evaporated under reduced pressure and (*E*)-4-[(2-iodophenyl)diazenyl]phenyl acetate was isolated by flash chromatography using hexane/EtOAc (10:1) as red solid, 42% yield, m.p. = 88-89°C.

^{1}H-NMR (300 MHz, $CDCl_3$) δ (ppm) 8.09–7.98 (m, 3H), 7.62 (dd, *J* = 8.0, 1.6 Hz, 1H), 7.43 (ddd, *J* = 8.1, 7.2, 1.3 Hz, 1H), 7.30–7.24 (m, 2H), 7.17 (ddd, *J* = 7.9, 7.2, 1.6 Hz, 1H), 2.35 (s, 3H).

^{13}C-NMR (101 MHz, $CDCl_3$) δ (ppm) 169.2, 153.2, 151.4, 150.1, 140.0, 132.4, 129.1, 124.9, 122.5, 117.5, 102.7, 21.3.

HRMS (ESI+) m/z calculated for $C_{14}H_{11}IN_2NaO_2$ $[M+Na]^+$: 388,9757, found $[M+Na]^+$: 388.9755.

(*E*)-1-(5-Chloro-2-iodophenyl)-2-phenyldiazene

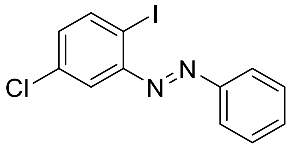

It was prepared following the general method A from nitrosobenzene (69% yield) and 5-chloro-2-iodoaniline. Eluent: hexane. Red solid, 24% yield, m.p. = 107-108 °C.

^{1}H-NMR (300 MHz, $CDCl_3$) δ (ppm) 8.05–7.97 (m, 2H), 7.93 (d, *J* = 8.4 Hz, 1H), 7.65 (d, *J* = 2.5 Hz, 1H), 7.60–7.49 (m, 3H), 7.16 (dd, *J* = 8.4, 2.5 Hz, 1H).

^{13}C-NMR (75 MHz, $CDCl_3$) δ (ppm) 152.1, 151.8, 140.5, 135.6, 132.1, 131.9, 129.3, 123.8, 117.8, 99.7.

HRMS (ESI+) m/z calculated for $C_{12}H_9ClIN_2$ $[M+H]^+$: 342.9493, found $[M+H]^+$: 342.9500.

(*E*)-1-(2-Iodo-4-(trifluoromethyl)phenyl)-2-phenyldiazene

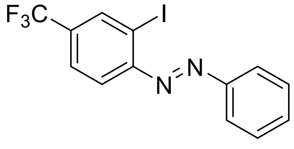

It was prepared following the general method A from nitrosobenzene (69% yield) and 2-iodo-4-(trifluoromethyl)aniline. Eluent: hexane. Red solid, 15% yield, m.p. = 77-78 ^{o}C.

^{1}H-NMR (300 MHz, $CDCl_3$) δ (ppm) 8.26–8.25 (m, 1H), 8.06–7.94 (m, 2H), 7.70–7.60 (m, 2H), 7.60–7.47 (m, 3H).

^{19}F-NMR (282 MHz, $CDCl_3$) δ (ppm) -63.39 (s).

^{13}C-NMR (75 MHz, $CDCl_3$) δ (ppm) 153.4, 152.2, 136.9, 136.8 (q, *J* = 4.0 Hz), 132.4, 129.4, 129.0, 126.1 (q, *J* = 3.6 Hz), 123.9, 120.6, 117.6, 101.3.

HRMS (ESI+) m/z calculated for $C_{13}H_9F_3IN_2$ $[M+H]^+$: 376.9757, found $[M+H]^+$: 376.9764.

Methyl (*E*)-3-iodo-4-(phenyldiazenyl)benzoate

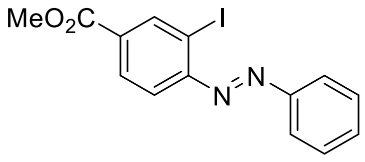

It was prepared following the general method A from nitrosobenzene (69% yield) and methyl 4-amino-3-iodobenzoate. Eluent: hexane. Red solid, 11% yield. m.p. = 68-69 ^{o}C.

^{1}H-NMR (300 MHz, $CDCl_3$) δ (ppm) 8.67 (d, *J* = 1.7 Hz, 1H), 8.07 (dd, *J* = 8.3, 1.8 Hz, 1H), 8.04–7.98 (m, 2H), 7.61 (d, *J* = 8.3 Hz, 1H), 7.58–7.51 (m, 3H), 3.95 (s, 3H).

^{13}C-NMR (126 MHz, $CDCl_3$) δ (ppm) 165.3, 154.1, 152.4, 141.2, 133.0, 132.4, 130.3, 129.4, 123.9, 117.2, 101.2, 52.7.

HRMS (ESI+) m/z calculated for $C_{14}H_{12}IN_2O_2$ $[M+H]^+$: 366.9938, found $[M+H]^+$: 366.9943.

(*E*)-1-(2-Iodo-4-methylphenyl)-2-phenyldiazene

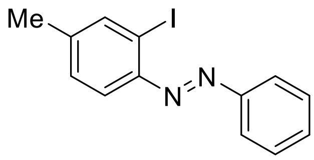

It was prepared following the general method A from nitrosobenzene (69% yield) and 2-iodo-4-methylaniline. Eluent: hexane. Red solid, 69% yield, m.p. = 136-137 ^{o}C.

^{1}H-NMR (300 MHz, $CDCl_3$) δ (ppm) 7.99 (ddd, *J* = 8.1, 1.6, 0.8 Hz, 2H), 7.87 (dt, *J* = 1.7, 0.8 Hz, 1H), 7.67–7.43 (m, 5H), 7.23 (ddd, *J* = 8.2, 1.7, 0.8 Hz, 1H), 2.40 (s, 3H).

^{13}C-NMR (75 MHz, $CDCl_3$) δ (ppm) 152.3, 149.2, 143.1, 140.2, 131.3, 129.8, 129.2, 123.5, 116.9, 103.4, 21.1.

HRMS (ESI+) m/z calculated for $C_{13}H_{12}IN_2$ $[M+H]^+$: 323.0040, found $[M+H]^+$: 323.0047.

(*E*)-1-(2-Iodo-3-methylphenyl)-2-phenyldiazene

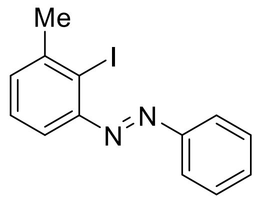

It was prepared following the general method A from nitrosobenzene (69% yield) and 3-iodo-2-methylaniline. Eluent: hexane. Red solid, 43% yield. m.p. = 38-39 ^{o}C.

^{1}H-NMR (300 MHz, $CDCl_3$) δ (ppm) 8.15–7.94 (m, 2H), 7.61–7.47 (m, 3H), 7.44–7.23 (m, 3H), 2.61 (s, 3H).

^{13}C-NMR (75 MHz, $CDCl_3$) δ (ppm) 153.8, 153.3, 144.8, 133.0, 132.9, 130.6, 129.7, 125.0, 115.9, 110.6, 29.9.

HRMS (ESI+) m/z calculated for $C_{14}H_{12}IN_2O_2$ $[M+H]^+$: 366.9938, found $[M+H]^+$: 366.9943.

General procedure for the synthesis of (*E*)-1-(2-alkynylphenyl)-2-phenyldiazenes

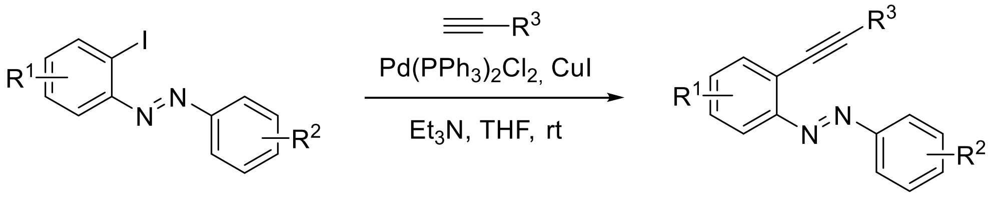

In a 50 mL Schlenk tube under argon flow, the corresponding 1-(2-iodophenyl)-2-phenyldiazene (0.97 mmol, 1.0 equiv.), $Pd(PPh_3)_2Cl_2$ (0.08 mmol, 0.08 equiv.), and CuI (0.1 mol, 0.1 equiv.) were dissolved in THF (0.05 M) at room temperature. Then, Et_3N (5.8 mmol, 6 equiv.) was added dropwise. After 10 min, the appropriate alkyne (1.2 mmol, 1.2 equiv.) was added. The reaction was stirred at room temperature until completion. The mixture was diluted with EtOAc (20 mL), washed with NH_4Cl (2 x 20 mL), dried over Mg_2SO_4 and filtered. The solvent was concentrated *in vacuo* and the corresponding (*E*)-1-(2-alkynylphenyl)-2-phenyldiazene was purified by flash chromatography using a mixture hexane:EtOAc.

(*E*)-1-[2-(Hex-1-yn-1-yl)phenyl]-2-phenyldiazene (1a)

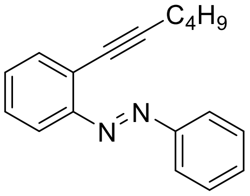

Eluent: hexane/EtOAc, 100:1. Red oil, 65% yield.

^{1}H-NMR (300 MHz, CDCl$_3$) δ (ppm) 8.04–7.91 (m, 2H), 7.67 (ddd, *J* = 6.4, 2.3, 0.6 Hz, 1H), 7.62–7.44 (m, 4H), 7.41–7.31 (m, 2H), 2.53 (t, *J* = 6.8 Hz, 2H), 1.67–1.50 (m, 4H), 0.96 (t, *J* = 7.2 Hz, 3H).

^{13}C-NMR (126 MHz, CDCl$_3$) δ (ppm) 153.4, 153.0, 133.6, 131.3, 130.6, 129.2, 128.2, 124.7, 123.4, 116.0, 97.4, 78.0, 31.0, 22.1, 19.7, 13.8.

HRMS (ESI+) m/z calculated for $C_{18}H_{18}N_2Na$ [M+Na]$^+$: 285.1362, found [M+Na]$^+$: 285.1365.

UV-Vis (MeOH) [λ (nm), ε (l/mol·cm)]: (220, 61085), (253, 36687), (321, 33002), (436, 4650).

(*Z*)-1-[2-(Hex-1-yn-1-yl)phenyl]-2-phenyldiazene (1a´)

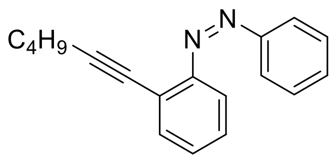

In a 5 mL Schlenk flask (*E*)-1-[2-(hex-1-yn-1-yl)phenyl]-2-phenyldiazene (0.15 mmol, 1 equiv) was dissolved in MeCN (0.1 M) and irradiated with blue LED (40 W) for 15 minutes. A ratio 66:34 of *Z:E* isomers was measured by ^{1}H-NMR. Afterwards the solvent was concentrated in vacuo and the *Z* isomer was isolated by flash chromatography using hexane as eluent (13 mg, 0.05 mmol, 34% yield).

^{1}H-NMR (500 MHz, CDCl$_3$) δ (ppm) 7.38 – 7.33 (m, 1H), 7.23 (dd, *J* = 8.5, 7.1 Hz, 2H), 7.17 – 7.10 (m, 1H), 7.08 – 7.01 (m, 2H), 6.95 – 6.88 (m, 2H), 6.40 – 6.35 (m, 1H), 2.42 (t, *J* = 7.1 Hz, 2H), 1.62 – 1.53 (m, 3H), 1.46 (dq, *J* = 9.4, 7.3 Hz, 2H), 0.93 (t, *J* = 7.3 Hz, 3H).

^{13}C-NMR (126 MHz, CDCl$_3$) δ (ppm) 156.5, 153.8, 132.8, 128.6, 127.8, 126.5, 120.7, 117.8, 116.3, 97.4, 76.8, 30.8, 22.1, 19.5, 13.8.

(*E*)-1-(4-Bromophenyl)-2-[2-(hex-1-yn-1-yl)phenyl]diazene (1b)

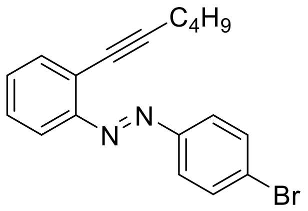

Eluent: hexane/EtOAc, 100:1. Red oil, 49% yield.

^{1}H-NMR (300 MHz, CDCl$_3$) δ (ppm) 7.90–7.79 (m, 2H), 7.70–7.55 (m, 4H), 7.45–7.28 (m, 2H), 2.52 (t, *J* = 6.8 Hz, 2H), 1.66–1.51 (m, 4H), 0.96 (t, *J* = 7.2 Hz, 3H).

^{13}C-NMR (126 MHz, CDCl$_3$) δ (ppm) 153.1, 151.7, 133.7, 132.4, 131.0, 128.3, 125.7, 125.1, 124.8, 115.9, 97.6, 77.9, 30.9, 22.1, 19.7, 13.8.

HRMS (ESI+) m/z calculated for $C_{18}H_{17}BrN_2Na$ $[M+Na]^+$: 363.0467, found $[M+Na]^+$: 363.0473.

UV-Vis (MeOH) [λ (nm), ε (l/mol·cm)]: (220, 3460), (331, 2260), (440, 266).

(*E*)-1-(3-Bromophenyl)-2-[2-(hex-1-yn-1-yl)phenyl]diazene (1c)

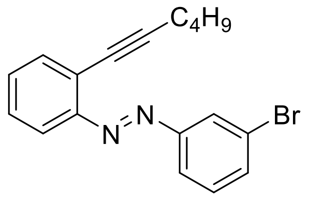

Eluent: hexane/EtOAc, 100:1. Red oil, 33% yield.

^{1}H-NMR (300 MHz, $CDCl_3$) δ (ppm) 8.10 (td, *J* = 1.9, 0.6 Hz, 1H), 7.95–7.91 (m, 1H), 7.73–7.54 (m, 3H), 7.44 – 7.32 (m, 3H), 2.57 (t, *J* = 6.9 Hz, 2H), 1.73–1.53 (m, 4H), 0.98 (t, *J* = 6.9 Hz, 3H).

^{13}C-NMR (75 MHz, $CDCl_3$) δ (ppm) 153.7, 142.1, 133.8, 133.6, 131.1, 130.4, 128.1, 125.2, 124.7, 123.7, 123.2, 115.8, 97.8, 77.8, 31.0, 22.3, 19.8, 14.0.

HRMS (ESI+) m/z calculated for $C_{18}H_{17}BrN_2Na$ $[M+Na]^+$: 363.0467, found $[M+Na]^+$: 363.0467.

UV-Vis (MeOH) [λ (nm), ε (l/mol·cm)]: (220,39567), (254, 23979), (321, 19182), (437, 2753).

(*E*)-1-(3-Fluorophenyl)-2-[2-(hex-1-yn-1-yl)phenyl]diazene (1d)

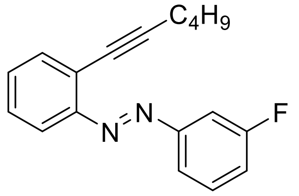

Eluent: hexane/EtOAc, 100:1. Red solid, 47% yield, m.p. = 33-34 ^oC.

^{1}H-NMR (300 MHz, $CDCl_3$) δ (ppm) 7.83–7.79 (m, 1H), 7.73–7.30 (m, 6H), 7.25–7.13 (m, 1H), 2.55 (t, *J* = 6.8 Hz, 2H), 1.73–1.49 (m, 4H), 0.98 (t, *J* = 6.7 Hz, 3H).

^{19}F-NMR (282 MHz, $CDCl_3$) δ (ppm) -112.36 – -112.48 (m).

^{13}C-NMR (75 MHz, $CDCl_3$) δ (ppm) 164.9, 161.6, 154.3, 152.8, 133.6, 131.0, 130.2 (d, *J* = 8.3 Hz), 128.1, 125.2, 121.3 (d, *J* = 2.9 Hz), 117.9 (d, *J* = 22.0 Hz), 115.8, 108.6 (d, *J* = 22.8 Hz), 97.7, 77.8, 31.0, 22.3, 19.8, 13.9.

HRMS (ESI+) m/z calculated for $C_{18}H_{17}FN_2Na$ $[M+Na]^+$: 303.1268, found $[M+Na]^+$: 303.1275.

UV-Vis (MeOH) [λ (nm), ε (l/mol·cm)]: (221,3487), (254, 3319), (327, 3160), (445, 331).

(*E*)-1-(2-Fluorophenyl)-2-[2-(hex-1-yn-1-yl)phenyl]diazene (1e)

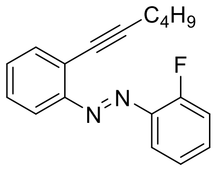

Eluent: hexane/EtOAc, 100:1. Red oil, 56% yield.

^{1}H-NMR (300 MHz, CDCl$_3$) δ (ppm) 7.82 (tt, *J* = 7.7, 1.6 Hz, 1H), 7.75–7.66 (m, 1H), 7.57 (dt, *J* = 7.3, 1.5 Hz, 1H), 7.47–7.18 (m, 5H), 2.53 (t, *J* = 6.8 Hz, 2H), 1.68–1.51 (m, 4H), 0.96 (t, *J* = 6.6 Hz, 3H).

^{19}F-NMR (282 MHz, CDCl$_3$) δ (ppm) -124.47 (dd, *J* = 11.6, 6.0 Hz).

^{13}C-NMR (75 MHz, CDCl$_3$) δ (ppm) 162.1, 158.7, 153.3, 133.5, 132.7 (*d*, *J* = 8.4 Hz), 131.0, 128.2, 125.0, 124.3 (d, *J* = 3.8 Hz), 118.1, 117.1 (d, *J* = 19.8 Hz), 116.2, 97.5, 77.9, 31.0, 22.2, 19.8, 14.0.

HRMS (ESI+) m/z calculated for $C_{18}H_{17}FN_2Na$ [M+Na]$^+$: 303.1268, found [M+Na]$^+$: 303.1270.

UV-Vis (MeOH) [λ (nm), ε (l/mol·cm)]: (220,34692), (255, 21466), (327, 19463), (440, 2399).

(*E*)-4-[(2-(hex-1-yn-1-yl)phenyl)diazenyl]benzonitrile (1f)

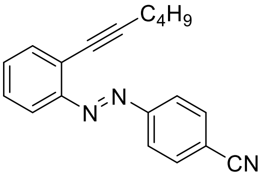

Eluent: hexane/EtOAc, 50:1. Red solid, 30% yield, m.p. = 49-50ºC.

^{1}H-NMR (300 MHz, CDCl$_3$) δ (ppm) 8.08–7.97 (m, 2H), 7.87–7.77 (m, 2H), 7.75–7.67 (m, 1H), 7.61 (dt, *J* = 7.7, 1.4 Hz, 1H), 7.48–7.33 (m, 2H), 2.53 (t, *J* = 6.6 Hz, 2H), 1.68–1.50 (m, 4H), 0.96 (t, *J* = 7.3 Hz, 3H).

^{13}C-NMR (75 MHz, CDCl$_3$) δ (ppm) 154.8, 152.9, 133.9, 133.3, 131.9, 128.3, 126.0, 123.8, 118.7, 115.8, 114.2, 98.2, 77.7, 30.9, 22.1, 19.7, 13.8.

HRMS (ESI+) m/z calculated for $C_{19}H_{18}N_3$ [M+H]$^+$: 288.1495, found [M+H]$^+$: 288.1506.

UV-Vis (MeOH) [λ (nm), ε (l/mol·cm)]: (220, 34813), (321, 18930), (441, 2170).

(*E*)-1-[2-(hex-1-yn-1-yl)phenyl]-2-(3-nitrophenyl)diazene (1g)

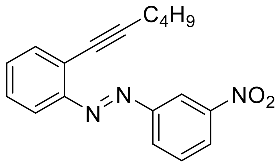

Eluent: hexane/EtOAc, 50:1. Red solid, 47% yield, m.p. = 58-59ºC.

¹H-NMR (300 MHz, CDCl₃) δ (ppm) 8.78 (t, *J* = 2.1 Hz, 1H), 8.36–8.28 (m, 2H), 7.77–7.65 (m, 2H), 7.61 (dd, *J* = 7.7, 1.6 Hz, 1H), 7.51–7.31 (m, 2H), 2.55 (t, *J* = 7.0 Hz, 2H), 1.74–1.47 (m, 5H), 0.94 (t, *J* = 7.3 Hz, 3H).

¹³C-NMR (101 MHz, CDCl₃) δ (ppm) 153.5, 152.7, 149.2, 133.8, 131.8, 130.1, 129.8, 128.3, 126.0, 125.1, 117.3, 115.9, 98.4, 77.6, 30.9, 22.2, 19.7, 13.8.

HRMS (ESI+) m/z calculated for $C_{18}H_{18}N_3O_2$ [M+H]$^+$: 308.1394, found [M+H]$^+$: 308.1397.

UV-Vis (MeOH) [λ (nm), ε (l/mol·cm)]: (220, 38683), (253, 24128), (314, 15631), (436, 2707).

(*E*)-1-[4-((2-(hex-1-yn-1-yl)phenyl)diazenyl)phenyl]ethan-1-one (1h)

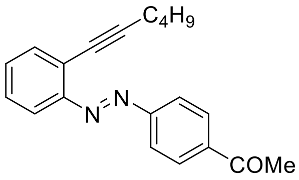

Eluent: hexane/EtOAc, 25:1. Red solid, 25% yield, m.p. = 48-49ºC.

¹H-NMR (300 MHz, CDCl₃) δ (ppm) 8.16–8.08 (m, 2H), 8.06–7.98 (m, 2H), 7.75–7.65 (m, 1H), 7.64–7.55 (m, 1H), 7.45–7.33 (m, 2H), 2.67 (s, 3H), 2.53 (t, *J* = 6.8 Hz, 2H), 1.69–1.50 (m, 4H), 0.96 (t, *J* = 7.2 Hz, 3H).

¹³C-NMR (75 MHz, CDCl₃) δ (ppm) 197.7, 155.3, 153.2, 138.6, 133.8, 131.3, 129.5, 128.2, 125.6, 123.4, 115.9, 97.90, 77.8, 30.9, 27.0, 22.1, 19.7, 13.9.

HRMS (ESI+) m/z calculated for $C_{20}H_{21}N_2O$ [M+H]$^+$: 305.1648, found [M+H]$^+$: 305.1658.

UV-Vis (MeOH) [λ (nm), ε (l/mol·cm)]: (220, 17721), (256, 10281), (326, 10480), (445, 1433).

Methyl (*E*)-4-[(2-(hex-1-yn-1-yl)phenyl)diazenyl]benzoate (1i)

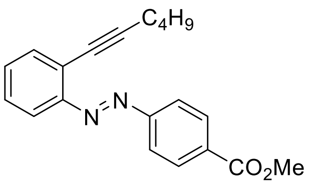

Eluent: hexane/EtOAc, 20:1. Red solid, 40% yield, m.p. = 62-63ºC.

¹H-NMR (300 MHz, CDCl₃) δ (ppm) 8.25–8.12 (m, 2H), 8.00 (dt, *J* = 8.3, 0.6 Hz, 2H), 7.73–7.66 (m, 1H), 7.63–7.55 (m, 1H), 7.45–7.32 (m, 2H), 3.97 (s, 3H), 2.55 (t, *J* = 6.7 Hz, 2H), 1.71–1.54 (m, 4H), 0.98 (t, *J* = 7.2 Hz, 3H).

¹³C-NMR (75 MHz, CDCl₃) δ (ppm) 166.5, 155.3, 153.0, 133.7, 131.9, 131.2, 130.6, 128.2, 125.4, 123.1, 115.8, 97.8, 77.8, 52.5, 31.0, 22.2, 19.8, 14.0.

HRMS (ESI+) m/z calculated for $C_{20}H_{21}N_2O_2$ $[M+H]^+$: 321.1598, found $[M+H]^+$: 321.1608.

UV-Vis (MeOH) [λ (nm), ε (l/mol·cm)]: (230, 23108), (255, 16394), (324, 18300), (459, 1405).

(*E*)-1-(2-Bromo-4-methylphenyl)-2-[2-(hex-1-yn-1-yl)phenyl]diazene (1j)

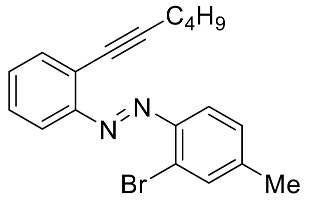

Eluent: hexane/EtOAc, 100:1. Red solid, 28% yield, m.p. = 42-43 ^oC.

^{1}H-NMR (300 MHz, $CDCl_3$) δ (ppm) 7.77 (dd, *J* = 7.4, 2.1 Hz, 1H), 7.70 (d, *J* = 8.2 Hz, 1H), 7.62–7.53 (m, 2H), 7.44–7.32 (m, 2H), 7.18 (ddd, *J* = 8.2, 1.8, 0.8 Hz, 1H), 2.55 (t, *J* = 6.7 Hz, 2H), 2.42 (s, 3H), 1.72–1.51 (m, 4H), 0.99 (t, *J* = 7.1 Hz, 3H).

^{13}C-NMR (75 MHz, $CDCl_3$) δ (ppm) 153.1, 147.7, 143.1, 134.0, 133.5, 130.8, 128.8, 128.2, 126.5, 125.1, 117.9, 116.4, 97.4, 78.0, 31.0, 22.2, 21.4, 19.8, 14.0.

HRMS (ESI+) m/z calculated for $C_{19}H_{19}BrN_2Na$ $[M+Na]^+$: 377.0624, found $[M+Na]^+$: 377.0628.

UV-Vis (MeOH) [λ (nm), ε (l/mol·cm)]: (220, 41382), (238, 32051), (249, 28670), (338, 20945), (443, 2153).

Dimethyl (*E*)-5-[(2-(hex-1-yn-1-yl)phenyl)diazenyl]isophthalate (1k)

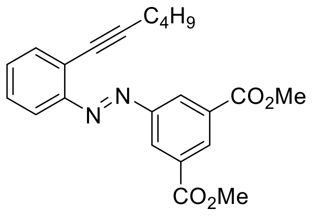

Eluent: hexane/EtOAc, 20:1. Red solid, 51% yield, m.p. = 61-62 ^oC.

^{1}H-NMR (300 MHz, $CDCl_3$) δ (ppm) 8.78 (dd, *J* = 7.7, 1.6 Hz, 3H), 7.73–7.66 (m, 1H), 7.62–7.56 (m, 1H), 7.42 (td, *J* = 7.5, 1.5 Hz, 1H), 7.36 (ddd, *J* = 8.8, 7.4, 1.6 Hz, 1H), 3.99 (s, 6H), 2.54 (t, *J* = 7.1 Hz, 2H), 1.73–1.62 (m, 2H), 1.57–1.46 (m, 2H), 0.91 (t, *J* = 7.3 Hz, 3H).

^{13}C-NMR (101 MHz, $CDCl_3$) δ (ppm) 165.8, 153.1, 152.9, 133.7, 132.5, 131.8, 131.5, 128.2, 128.2, 125.5, 116.0, 98.13, 77.6, 52.7, 30.8, 22.1, 19.6, 13.7.

HRMS (ESI+) m/z calculated for $C_{22}H_{23}N_2O_4$ $[M+H]^+$: 379.1652, found $[M+H]^+$: 379.1649.

UV-Vis (MeOH) [λ (nm), ε (l/mol·cm)]: (227, 5082), (317, 2209), (438, 211).

(*E*)-1-[2-(Hex-1-yn-1-yl)phenyl]-2-(perfluorophenyl)diazene (1l)

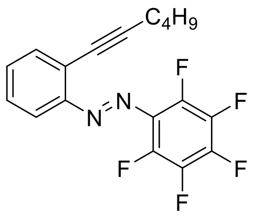

In a 500 mL round bottom flask 2,3,4,5,6-pentafluoroaniline (31.0 mmol, 1.0 equiv.) was dissolved in DCM (78 mL, 0.4 M). A solution of Oxone (62.0 mmol, 2 equiv.) in H_2O (155 mL, 0.4 M) was added slowly, and the mixture was stirred at room temperature for 24 h. The mixture was extracted with DCM (3 x 100 mL). The organic phases were combined, dried over Mg_2SO_4, filtered and concentrated *in vacuo* to afford 1,2,3,4,5-pentafluoro-6-nitrosobenzene in 85% yield, which was used in the next step without further purification.

2-(Hex-1-yn-1-yl)aniline was dissolved in AcOH (173 mL, 0.1 M) and 1,2,3,4,5-pentafluoro-6-nitrosobenzene (26.0 mmol, 1.5 equiv.) was added. The reaction was stirred in an oil bath at 85 ^{0}C until completion. The crude was cooled down to room temperature, diluted with EtOAc (150 mL) and washed with brine (3 x 100 mL) and H_2O (2 x 100 mL). The combined organic phases were dried over Mg_2SO_4, filtered and concentrated *in vacuo* to afford (*E*)-1-[2-(hex-1-yn-1-yl)phenyl]-2-(perfluorophenyl)diazene, which was further purified by flash chromatography using hexane as eluent as colorless oil in 11% yield.

^{1}H-NMR (300 MHz, CDCl$_3$) δ (ppm) 7.72–7.56 (m, 2H), 7.46 (td, *J* = 7.5, 1.4 Hz, 1H), 7.36 (ddd, *J* = 8.1, 7.3, 1.5 Hz, 1H), 2.50 (t, *J* = 6.9 Hz, 2H), 1.66–1.44 (m, 4H), 0.93 (t, *J* = 7.2 Hz, 3H).

^{19}F-NMR (282 MHz, CDCl$_3$) δ (ppm) -149.46 – -149.75 (m), -152.39 (tt, *J* = 21.1, 2.3 Hz), -162.15 – -162.57 (m).

^{13}C-NMR (126 MHz, CDCl$_3$) δ (ppm) 153.8, 133.9, 133.7, 132.5, 128.9, 128.2, 127.9, 126.3, 115.5, 98.6, 77.3, 30.8, 30.6, 22.1, 19.7, 19.4, 13.7.

HRMS (ESI+) m/z calculated for $C_{18}H_{14}F_5N_2$ $[M+H]^+$: 353.1072, found $[M+H]^+$: 353.1079.

UV-Vis (MeOH) [λ (nm), ε (l/mol·cm)]: (220, 78466), (228, 77228), (319, 47403), (440, 5499).

(*E*)-1-[2-(Hex-1-yn-1-yl)phenyl]-2-(*p*-tolyl)diazene (1m)

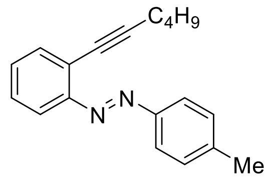

Eluent: hexane/EtOAc, 100:1. Red oil, 46% yield.

^{1}H-NMR (300 MHz, CDCl$_3$) δ (ppm) 7.95–7.83 (m, 2H), 7.71–7.62 (m, 1H), 7.62–7.53 (m, 1H), 7.42–7.27 (m, 4H), 2.53 (t, *J* = 6.7 Hz, 2H), 2.44 (s, 3H), 1.69–1.50 (m, 4H), 0.96 (t, *J* = 7.2 Hz, 3H).

^{13}C-NMR (101 MHz, CDCl$_3$) δ (ppm) 153.5, 151.2, 141.9, 133.6, 130.3, 129.8, 128.2, 124.5, 123.4, 116.0, 97.2, 78.0, 31.0, 22.1, 21.7, 19.7, 13.8.

HRMS (ESI+) m/z calculated for $C_{19}H_{21}N_2$ $[M+H]^+$: 277.1699, found $[M+H]^+$: 277.1704.

UV-Vis (MeOH) [λ (nm), ε (l/mol·cm)]: (220, 41325), (254, 24880), (329, 25784), (435, 3322).

(*E*)-*N*-[4-((2-(Hex-1-yn-1-yl)phenyl)diazenyl)phenyl]-N-methylacetamide (1o)

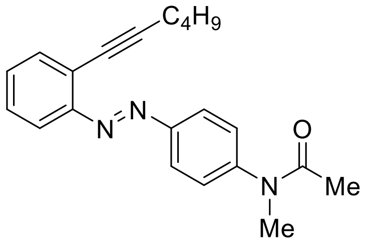

Eluent: hexane/EtOAc, 2:1. Red oil, 41% yield.

^{1}H-NMR (300 MHz, $CDCl_3$) δ (ppm) 8.06–7.98 (m, 2H), 7.69 (dd, *J* = 7.4, 2.1 Hz, 1H), 7.62–7.55 (m, 1H), 7.43–7.32 (m, 4H), 3.33 (s, 3H), 2.53 (t, *J* = 6.8 Hz, 2H), 1.97 (s, 3H), 1.67–1.50 (m, 4H), 0.95 (t, *J* = 7.2 Hz, 3H).

^{13}C-NMR (101 MHz, $CDCl_3$) δ (ppm) 170.5, 153.1, 146.9, 133.8, 131.0, 128.3, 127.8, 125.1, 124.6, 115.9, 97.6, 77.9, 37.3, 31.0, 22.7, 22.1, 19.7, 13.9.

HRMS (ESI+) m/z calculated for $C_{21}H_{24}N_3O$ $[M+H]^+$: 334.1914, found $[M+H]^+$: 334.1909.

UV-Vis (MeOH) [λ (nm), ε (l/mol·cm)]: (220, 94592), (326, 36026), (430, 8056).

(*E*)-1-[2-(Hex-1-yn-1-yl)phenyl]-2-(4-methoxyphenyl)diazene (1p)

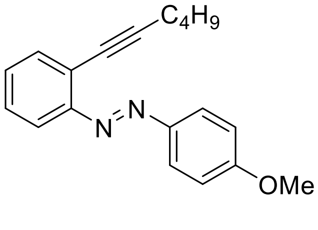

Eluent: hexane/EtOAc, 50:1. Red oil, 70% yield.

^{1}H-NMR (300 MHz, $CDCl_3$) δ (ppm) 8.02–7.93 (m, 2H), 7.69–7.62 (m, 1H), 7.56 (ddd, *J* = 5.7, 3.1, 1.2 Hz, 1H), 7.38–7.29 (m, 2H), 7.05–6.97 (m, 2H), 3.91 (s, 3H), 2.54 (t, *J* = 6.8 Hz, 2H), 1.72–1.52 (m, 4H), 1.03–0.93 (m, 3H).

^{13}C-NMR (75 MHz, $CDCl_3$) δ (ppm) 162.2, 153.4, 147.4, 133.4, 129.9, 128.1, 125.2, 124.1, 115.9, 114.2, 97.0, 78.1, 55.8, 31.1, 22.3, 19.8, 14.0.

HRMS (ESI+) m/z calculated for $C_{19}H_{21}N_2O$ $[M+H]^+$: 293.1648, found $[M+H]^+$: 293.1660.

UV-Vis (MeOH) [λ (nm), ε (l/mol·cm)]: (220, 4936), (254, 2468), (329, 547).

(*E*)-4-[(2-(Hex-1-yn-1-yl)phenyl)diazenyl]phenyl acetate (1q)

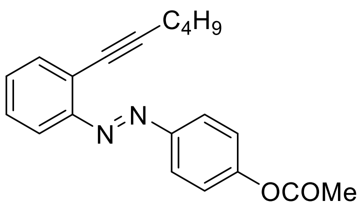

Eluent: hexane/EtOAc, 20:1. Red oil, 42% yield.

^{1}H-NMR (300 MHz, $CDCl_3$) δ (ppm) 8.05–7.96 (m, 2H), 7.69–7.63 (m, 1H), 7.61–7.53 (m, 1H), 7.40–7.31 (m, 2H), 7.29–7.21 (m, 2H), 2.52 (t, *J* = 6.8 Hz, 2H), 2.34 (s, 3H), 1.69–1.50 (m, 4H), 0.96 (t, *J* = 7.2 Hz, 3H).

^{13}C-NMR (75 MHz, $CDCl_3$) δ (ppm) 169.2, 153.2, 150.6, 133.6, 132.3, 130.7, 128.2, 124.8, 124.6, 122.3, 115.9, 97.4, 77.9, 30.9, 22.1, 21.3, 19.7, 13.8.

HRMS (ESI+) m/z calculated for $C_{20}H_{22}N_2O_2$ $[M+H]^+$: 321.1598, found $[M+H]^+$: 321.1605.

UV-Vis (MeOH) [λ (nm), ε (l/mol·cm)]: (226, 4054), (254, 2528), (327, 2577), (440, 248).

(*E*)-1-[5-Chloro-2-(hex-1-yn-1-yl)phenyl]-2-phenyldiazene (1r)

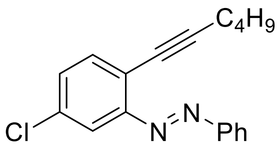

Eluent: hexane/EtOAc, 100:1. Red oil, 20% yield.

^{1}H-NMR (300 MHz, $CDCl_3$) δ (ppm) 8.06–7.90 (m, 2H), 7.69 (d, *J* = 2.2 Hz, 1H), 7.60–7.44 (m, 4H), 7.34 (ddd, *J* = 8.3, 2.2, 0.6 Hz, 1H), 2.54 (t, *J* = 6.8 Hz, 2H), 1.68–1.53 (m, 4H), 0.98 (t, *J* = 7.1 Hz, 3H).

^{13}C-NMR (75 MHz, $CDCl_3$) δ (ppm) 153.6, 152.6, 134.4, 134.2, 131.7, 130.3, 129.2, 123.5, 120.6, 116.3, 98.3, 77.1, 30.9, 22.2, 19.8, 13.9.

HRMS (ESI+) m/z calculated for $C_{18}H_{17}ClN_2Na$ $[M+Na]^+$: 319.0972, found $[M+Na]^+$: 319.0978.

UV-Vis (MeOH) [λ (nm), ε (l/mol·cm)]: (220, 5217), (232, 5067), (257, 4164), (315, 2959), (440, 399).

(*E*)-1-[2-(Hex-1-yn-1-yl)-4-(trifluoromethyl)phenyl]-2-phenyldiazene (1s)

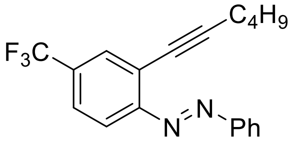

Eluent: hexane/EtOAc, 100:1. Red solid, 67% yield. m.p. = 32-33 ^{o}C.

^{1}H-NMR (300 MHz, $CDCl_3$) δ (ppm) 8.04–7.92 (m, 2H), 7.86–7.80 (m, 1H), 7.73 (d, *J* = 8.5 Hz, 1H), 7.61–7.45 (m, 4H), 2.54 (t, *J* = 6.3 Hz, 2H), 1.69–1.51 (m, 4H), 0.97 (t, *J* = 7.2 Hz, 3H).

^{19}F-NMR (282 MHz, $CDCl_3$) δ (ppm) -63.65 (s).

^{13}C-NMR (75 MHz, $CDCl_3$) δ (ppm) 154.9 (q, *J* = 1.7 Hz), 152.7, 131.9 (q, *J* = 32.7 Hz), 130.6 (q, *J* = 3.9 Hz), 129.2, 128.4, 124.9, 124.8 (q, *J* = 3.6 Hz), 123.7 (q, *J* = 270.0 Hz), 123.6, 120.6, 116.7, 99.0, 76.9, 30.8, 22.2, 19.7, 13.9.

HRMS (ESI+) m/z calculated for $C_{19}H_{17}F_3N_2Na$ $[M+Na]^+$: 353.1236, found $[M+Na]^+$: 353.1231.

UV-Vis (MeOH) [λ (nm), ε (l/mol·cm)]: (220, 38183), (318, 21974), (440, 3164).

Methyl (*E*)-3-(hex-1-yn-1-yl)-4-(phenyldiazenyl)benzoate (1t)

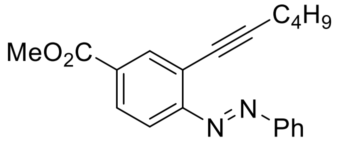

Eluent: hexane/EtOAc, 100:1. Red solid, 44% yield, m.p. = 75-76 ^{o}C.

^{1}H-NMR (500 MHz, $CDCl_3$) δ (ppm) 8.25 (d, *J* = 1.9 Hz, 1H), 8.04–7.95 (m, 3H), 7.67 (dd, *J* = 8.4, 0.5 Hz, 1H), 7.56–7.49 (m, 3H), 3.94 (s, 3H), 2.53 (t, *J* = 6.9 Hz, 2H), 1.69–1.61 (m, 2H), 1.59–1.49 (m, 2H), 0.95 (t, *J* = 7.3 Hz, 3H).

^{13}C-NMR (126 MHz, $CDCl_3$) δ (ppm) 166.2, 155.8, 152.9, 135.1, 131.9, 131.6, 129.3, 129.3, 124.5, 123.6, 116.3, 98.3, 77.2, 52.5, 30.8, 22.1, 19.6, 13.8.

HRMS (ESI+) m/z calculated for $C_{20}H_{20}N_2NaO_2$ $[M+Na]^+$: 343.1417, found $[M+Na]^+$: 343.1417.

UV-Vis (MeOH) [λ (nm), ε (l/mol·cm)]: (220, 8545), (254, 7205), (325, 6512), (456, 432).

(*E*)-1-[2-(Hex-1-yn-1-yl)-4-methylphenyl]-2-phenyldiazene (1u)

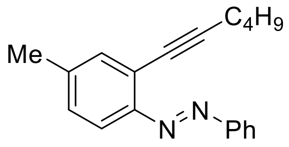

Eluent: hexane/EtOAc, 100:1. Red oil, 31% yield.

^{1}H-NMR (300 MHz, $CDCl_3$) δ (ppm) 8.00–7.91 (m, 2H), 7.62 (d, *J* = 8.3 Hz, 1H), 7.55–7.44 (m, 3H), 7.40 (s, 1H), 7.15 (d, *J* = 8.3 Hz, 1H), 2.54 (t, *J* = 6.7 Hz, 2H), 2.40 (s, 3H), 1.70–1.54 (m, 4H), 0.98 (t, *J* = 7.1 Hz, 3H).

^{13}C-NMR (75 MHz, $CDCl_3$) δ (ppm) 152.9, 151.2, 141.1, 133.9, 130.9, 129.2, 129.0, 124.8, 123.1, 115.7, 96.9, 78.1, 31.1, 22.3, 21.5, 19.8, 14.0.

HRMS (ESI+) m/z calculated for $C_{19}H_{21}N_2$ $[M+H]^+$: 277.1699, found $[M+H]^+$: 277.1706.

UV-Vis (MeOH) [λ (nm), ε (l/mol·cm)]: (220, 3793), (260, 2258), (329, 2198), (438, 287).

(*E*)-1-[2-(Hex-1-yn-1-yl)-3-methylphenyl]-2-phenyldiazene (1v)

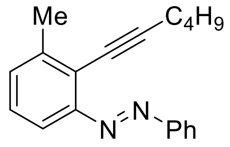

Eluent: hexane/EtOAc, 100:1. Red oil, 39% yield.

^{1}H-NMR (300 MHz, CDCl$_3$) δ (ppm) 8.07–7.91 (m, 2H), 7.63–7.41 (m, 4H), 7.32–7.21 (m, 2H), 2.64–2.52 (m, 5H), 1.72–1.51 (m, 4H), 0.97 (t, *J* = 7.2 Hz, 3H).

^{13}C-NMR (101 MHz, CDCl$_3$) δ (ppm) 153.8, 153.1, 141.8, 131.5, 131.1, 129.1, 123.4, 113.2, 102.0, 76.5, 31.1, 22.2, 20.9, 19.8, 13.8.

HRMS (ESI+) m/z calculated for $C_{19}H_{21}N_2$ [M+H]$^+$: 277.1699 found [M+H]$^+$: 277.1694.

UV-Vis (MeOH) [λ (nm), ε (l/mol·cm)]: (220, 38977), (327, 21453), (440, 3186).

(*E*)-1-(2-Ethynylphenyl)-2-phenyldiazene (1w)

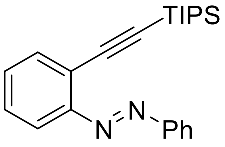

Eluent: hexane/EtOAc, 100:1. Red oil, 95% yield.

^{1}H-NMR (300 MHz, CDCl$_3$) δ (ppm) 8.05–7.96 (m, 2H), 7.74–7.63 (m, 2H), 7.52–7.46 (m, 3H), 7.40 (dd, *J* = 6.0, 3.4 Hz, 2H), 1.16 (s, 21H).

^{13}C-NMR (101 MHz, CDCl$_3$) δ (ppm) 153.7, 152.9, 134.2, 131.4, 130.5, 129.1, 129.0, 124.1, 123.6, 115.9, 104.0, 97.7, 18.9, 11.6.

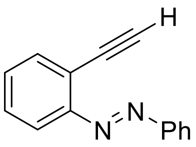

(*E*)-1-phenyl-2-[2-((triisopropylsilyl)ethynyl)phenyl]diazene (1.0 mmol, 1.0 equiv.) was dissolved in THF/MeOH (20:1, 10.5 mL) and treated with TBAF (3.0 mmol, 3.0 equiv.). After 10 min, the mixture was diluted with EtOAc (10 mL), washed with NH_4Cl (2 x 10 mL), dried over Mg_2SO_4 and filtered. The organic phase was concentrated in vacuo to afford (*E*)-1-(2-ethynylphenyl)-2-phenyldiazene as a red oil (91% yield) which was used immediately without further purification.

(*E*)-1-[2-(5-Methylhex-1-yn-1-yl)phenyl]-2-phenyldiazene (1x)

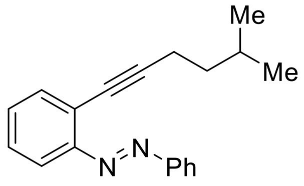

Eluent: hexane/EtOAc, 100:1. Red oil, 44% yield.

^{1}H-NMR (300 MHz, CDCl$_3$) δ (ppm) 8.05–7.94 (m, 2H), 7.74–7.65 (m, 1H), 7.64–7.45 (m, 4H), 7.43–7.31 (m, 2H), 2.55 (t, *J* = 7.2 Hz, 2H), 1.95–1.81 (m, 1H), 1.58 (q, *J* = 7.2 Hz, 2H), 1.01 (d, *J* = 0.6 Hz, 3H), 0.96 (dd, *J* = 6.6, 0.6 Hz, 6H).

^{13}C-NMR (75 MHz, CDCl$_3$) δ (ppm) 153.4, 153.0, 133.6, 131.3, 130.6, 129.1, 128.2, 124.7, 123.4, 116.0, 97.3, 77.9, 37.7, 27.2, 22.4, 18.0.

HRMS (ESI+) m/z calculated for $C_{19}H_{20}N_2Na$ [M+Na]$^+$: 299.1519, found [M+Na]$^+$: 299.1518.

UV-Vis (MeOH) [λ (nm), ε (l/mol·cm)]: (220, 48898), (310, 20624), (430, 2855).

(*E*)-1-(2-(3-Cyclohexylprop-1-yn-1-yl)phenyl)-2-phenyldiazene (1y)

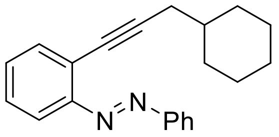

Eluent: hexane/EtOAc, 100:1. Red oil, 54% yield.

^{1}H-NMR (300 MHz, CDCl$_3$) δ (ppm) 8.03–7.92 (m, 2H), 7.72–7.63 (m, 1H), 7.62–7.43 (m, 4H), 7.41–7.31 (m, 2H), 2.43 (d, *J* = 6.6 Hz, 2H), 2.01–1.89 (m, 2H), 1.81–1.59 (m, 4H), 1.36–1.07 (m, 5H).

^{13}C-NMR (75 MHz, CDCl$_3$) δ (ppm) 153.3, 152.8, 133.5, 131.2, 130.5, 129.1, 128.1, 124.7, 123.3, 115.9, 96.3, 78.8, 37.8, 33.0, 27.9, 26.5, 26.4.

HRMS (ESI+) m/z calculated for $C_{21}H_{22}N_2Na$ [M+Na]$^+$: 325.1675 found [M+Na]$^+$: 325.1677.

UV-Vis (MeOH) [λ (nm), ε (l/mol·cm)]: (220, 45046), (254, 27105), (320, 24168), (436, 3520).

(*E*)-1-(4-Bromophenyl)-2-[2-(cyclopropylethynyl)phenyl]diazene (1z)

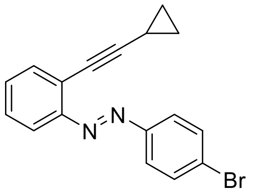

Eluent: hexane/EtOAc, 100:1. Red solid, 25% yield, m.p. = 48-49 ^{o}C.

^{1}**H-NMR (300 MHz, $CDCl_3$)** δ (ppm) 7.89–7.77 (m, 2H), 7.72–7.60 (m, 3H), 7.60–7.51 (m, 1H), 7.40–7.30 (m, 2H), 1.60–1.51 (m, 1H), 1.06–0.74 (m, 4H).

^{13}**C-NMR (75 MHz, $CDCl_3$)** δ (ppm) 153.2, 151.7, 133.7, 132.5, 130.9, 128.2, 125.7, 124.9, 124.8, 116.0, 100.8, 73.0, 9.1, 0.8.

HRMS (ESI+) m/z calculated for $C_{17}H_{14}BrN_2$ [M+H]$^+$: 325.0335, found [M+H]$^+$: 325.0339.

UV-Vis (MeOH) [λ (nm), ε (l/mol·cm)]: (220, 35532), (324, 22240), (430, 2610).

(*E*)-1-(2-(5-Chloropent-1-yn-1-yl)phenyl]-2-phenyldiazene (1aa)

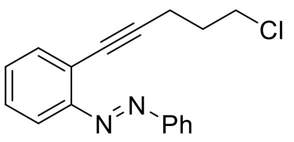

Eluent: hexane/EtOAc, 100:1. Red oil, 27% yield.

^{1}**H-NMR (300 MHz, $CDCl_3$)** δ (ppm) 8.04–7.91 (m, 2H), 7.74–7.65 (m, 1H), 7.63–7.45 (m, 4H), 7.44–7.34 (m, 2H), 3.77 (t, *J* = 6.4 Hz, 2H), 2.73 (t, *J* = 6.7 Hz, 2H), 2.10 (quint, *J* = 6.5 Hz, 2H).

^{13}**C-NMR (75 MHz, $CDCl_3$)** δ (ppm) 153.4, 152.9, 133.5, 131.4, 130.6, 129.3, 128.6, 124.2, 123.3, 116.1, 94.8, 79.0, 43.9, 31.6, 17.4.

HRMS (ESI+) m/z calculated for $C_{17}H_{16}ClN_2$ [M+H]$^+$: 283.0997, found [M+H]$^+$: 283.1005.

UV-Vis (MeOH) [λ (nm), ε (l/mol·cm)]: (220, 39860), (252, 21833), (321,20933), (437, 2772).

(*E*)-4-[2-(Phenyldiazenyl)phenyl]but-3-yn-1-ol (1ab)

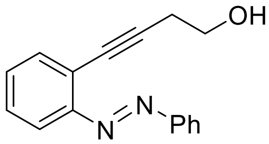

Eluent: hexane/EtOAc, 2:1. Red oil, 37% yield.

^{1}**H-NMR (300 MHz, $CDCl_3$)** δ (ppm) 7.99–7.88 (m, 2H), 7.73–7.66 (m, 1H), 7.61–7.48 (m, 4H), 7.44–7.36 (m, 2H), 3.85 (t, *J* = 6.1 Hz, 2H), 2.79 (t, *J* = 6.1 Hz, 2H), 2.20 (bs, 1H).

^{13}**C-NMR (75 MHz, $CDCl_3$)** δ (ppm) 153.3, 152.8, 133.3, 131.4, 130.6, 129.2, 128.7, 123.7, 123.2, 116.3, 93.2, 80.3, 61.2, 24.5.

HRMS (ESI+) m/z calculated for $C_{16}H_{15}N_2O$ [M+H]$^+$: 251.1179, found [M+H]$^+$: 251.1188.

UV-Vis (MeOH) [λ (nm), ε (l/mol·cm)]: (220, 27834), (316, 11343), (418, 1351).

(*E*)-1-Phenyl-2-[2-(4-phenylbut-1-yn-1-yl)phenyl]diazene (1ac)

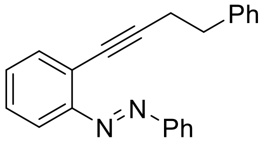

Eluent: hexane/EtOAc, 100:1. Red oil, 25% yield.

^{1}H-NMR (300 MHz, $CDCl_3$) δ (ppm) 8.01–7.91 (m, 2H), 7.73–7.64 (m, 1H), 7.61–7.45 (m, 4H), 7.43–7.17 (m, 7H), 3.00 (t, *J* = 7.4 Hz, 2H), 2.82 (t, *J* = 7.0 Hz, 2H).

^{13}C-NMR (75 MHz, $CDCl_3$) δ (ppm) 153.3, 152.9, 140.8, 133.7, 131.3, 130.6, 129.2, 128.7, 128.6, 128.5, 128.4, 126.4, 124.4, 123.4, 116.0, 96.4, 78.6, 35.3, 22.2.

HRMS (ESI+) m/z calculated for $C_{22}H_{19}N_2$ $[M+H]^+$: 311.1543, found $[M+H]^+$: 311.1548.

UV-Vis (MeOH) [λ (nm), ε (l/mol·cm)]: (220, 30950), (225, 30045), (253, 21142), (320, 18374), (438, 2131).

(*E*)-1-(2-((4-Methoxyphenyl)ethynyl)phenyl)-2-phenyldiazene (1ad)

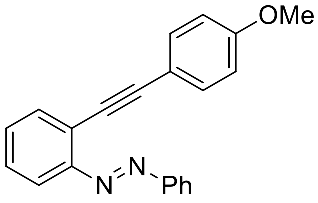

Eluent: hexane/EtOAc, 10:1. Red solid, 47% yield, m.p. = 70-71 ^{o}C.

^{1}H-NMR (500 MHz, $CDCl_3$) δ (ppm) 8.06 – 8.00 (m, 2H), 7.77 – 7.72 (m, 1H), 7.72 – 7.67 (m, 1H), 7.58 – 7.47 (m, 5H), 7.46 – 7.37 (m, 2H), 6.93 – 6.88 (m, 2H), 3.84 (s, 3H).

^{13}C-NMR (126 MHz, $CDCl_3$) δ (ppm) 160.0, 153.1, 153.0, 133.3, 133.3, 131.4, 130.7, 129.3, 128.7, 124.2, 123.4, 116.3, 115.7, 114.2, 96.0, 85.7, 55.5.

HRMS (ESI+) m/z calculated for $C_{21}H_{17}N_2O$ $[M+H]^+$: 313.1335, found $[M+H]^+$: 313.1339.

UV-Vis (MeOH) [λ (nm), ε (l/mol·cm)]: (286, 30463), (438, 1050).

(*E*)-4-[(2-(Hex-1-yn-1-yl)phenyl)diazenyl]-*N*,*N*-dimethylaniline (1n)

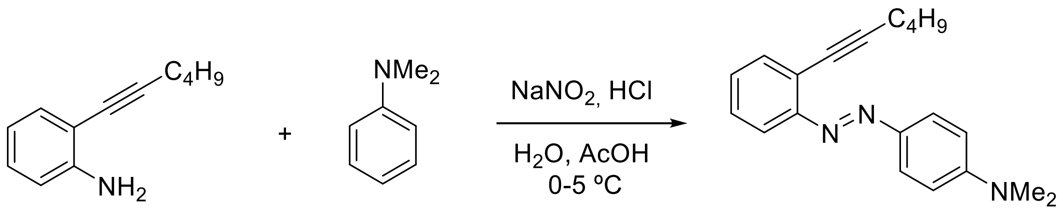

A suspension of 2-(hex-1-yn-1-yl)aniline (5,34 mmol, 1 equiv.) in water (1 M) was stirred for 5 minutes. HCl (1.1 mL) was added to the suspension, furnishing a clear solution. The mixture was cold down in an ice-bath and a solution of $NaNO_2$ (5.87 mmol, 1.1 equiv., 0.45 M) was added dropwise and stirred for 15 minutes. The resulting mixture was added to a cold solution of dimethylaniline (5.87 mmol, 1.1 equiv.) in glacial acetic acid (2.2 mL) and H_2O (5.5 mL). The mixture was stirred for 10 minutes and then an aqueous solution of Na_2CO_3 was added until pH = 6. The precipitate was filtered and washed with hexane. The product was purified by column chromatography with hexane/EtOAc (10:1).

Red solid, 45% yield, m.p. = 64-65 ^oC.

^{1}H-NMR (300 MHz, $CDCl_3$) δ (ppm) 7.98 – 7.89 (m, 2H), 7.68 – 7.62 (m, 1H), 7.56 – 7.50 (m, 1H), 7.35 – 7.27 (m, 2H), 6.79 – 6.75 (m, 2H), 3.09 (s, 6H), 2.53 (t, *J* = 6.9 Hz, 2H), 1.68 – 1.62 (m, 2H), 1.59 – 1.53 (m, 2H), 0.97 (t, *J* = 7.3 Hz, 3H).

^{13}C-NMR (101 MHz, $CDCl_3$) δ (ppm) 154.0, 152.6, 133.4, 129.0, 128.2, 125.6, 123.4, 116.0, 111.7, 96.5, 78.4, 40.5, 31.0, 22.2, 19.7, 13.9.

HRMS (ESI+) m/z calculated for $C_{20}H_{24}N_3$ $[M+H]^+$: 306.1965, found $[M+H]^+$: 306.1959.

UV-vis (MeOH) [λ (nm), ε (l/mol·cm)]: (220, 4644), (236, 3456), (269, 2286), (421, 3563).

General procedure for the synthesis of 2*H*-indazoles (2a-2ay)

Method A

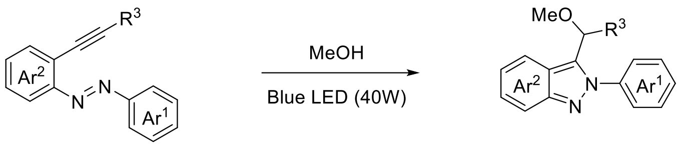

The corresponding alkynylazobenzene **1** (0.1 mmol) was dissolved in MeOH (0.1 M) in a Schlenk tube (5 mL) under argon. The reaction mixture was placed in the photo-reactor and stirred for 36 h under blue light irradiation (40 W, blue LED) at room temperature until completion. The solvent was removed under reduced pressure and the mixture was purified by flash chromatography using mixture hexane:EtOAc as eluent to yield the corresponding indazole **2**.

3-(1-Methoxypentyl)-2-phenyl-2*H*-indazole (2a)

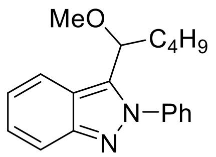

Eluent: hexane/EtOAc, 20:1. White solid, 95% yield (31 mg). (1 mmol scale: 90% yield, 265 mg), m.p. = 61-62 ^{o}C.

^{1}H-NMR (300 MHz, $CDCl_3$) δ (ppm) 7.90 (dd, *J* = 8.5, 1.1 Hz, 1H), 7.75 (dd, *J* = 8.7, 1.0 Hz, 1H), 7.59–7.48 (m, 5H), 7.34 (ddd, *J* = 8.8, 6.6, 1.1 Hz, 1H), 7.11 (ddd, *J* = 8.5, 6.6, 0.9 Hz, 1H), 4.59 (dd, *J* = 7.7, 6.1, 1H), 3.22 (s, 1H), 2.11–1.98 (m, 1H), 1.91–1.78 (m, 1H), 1.38–1.12 (m, 4H), 0.81 (t, *J* = 7.0 Hz, 3H).

^{13}C-NMR (101 MHz, $CDCl_3$) δ (ppm) 148.9, 140.1, 136.6, 129.4, 129.3, 129.3, 126.9, 126.7, 121.9, 121.1, 120.6, 117.8, 76.4, 56.9, 35.6, 28.1, 22.5, 14.0.

HRMS (ESI+) m/z calculated for $C_{19}H_{22}N_2NaO$ $[M+Na]^+$: 317.1624, found $[M+Na]^+$: 317.1629.

2-(4-Bromophenyl)-3-(1-methoxypentyl)-2*H*-indazole (2b)

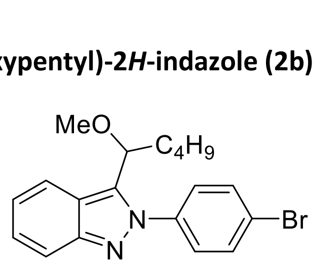

Eluent: hexane/EtOAc, 20:1. Yellow solid, 98% yield (43 mg), m.p. = 72-73 ^{o}C.

^{1}H-NMR (300 MHz, $CDCl_3$) δ (ppm) 7.87 (dt, *J* = 8.5, 1.1 Hz, 1H), 7.78–7.63 (m, 3H), 7.48–7.40 (m, 2H), 7.35 (ddd, *J* = 8.8, 6.6, 1.1 Hz, 1H), 7.12 (ddd, *J* = 8.5, 6.6, 0.9 Hz, 1H), 4.58 (dd, *J* = 7.8, 6.2

Hz, 1H), 3.21 (s, 3H), 2.10–1.95 (m, 1H), 1.90–1.72 (m, 1H), 1.38–1.09 (m, 4H), 0.81 (t, *J* = 8.0 Hz, 3H).

^{13}C-NMR (101 MHz, CDCl$_3$) δ (ppm) 149.1, 139.3, 136.5, 132.5, 128.2, 127.1, 123.4, 122.2, 121.0, 120.9, 117.8, 76.2, 56.9, 35.3, 28.1, 22.5, 14.0.

HRMS (ESI+) m/z calculated for $C_{19}H_{21}BrN_2NaO$ $[M+Na]^+$: 395.0729, found $[M+Na]^+$: 395.0731.

2-(3-Bromophenyl)-3-(1-methoxypentyl)-2*H*-indazole (2c)

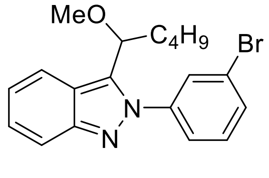

Eluent: hexane/EtOAc, 20:1. Yellow oil, 86% yield (30 mg).

^{1}H-NMR (300 MHz, CDCl$_3$) δ (ppm) 7.88 (dt, *J* = 8.6, 1.1 Hz, 1H), 7.75–7.72 (m, 2H), 7.66 (ddd, *J* = 7.8, 1.9, 1.3 Hz, 1H), 7.50 (ddd, *J* = 8.0, 1.9, 1.2 Hz, 1H), 7.45–7.30 (m, 2H), 7.12 (ddd, *J* = 8.6, 6.6, 0.9 Hz, 1H), 4.60 (dd, *J* = 7.7, 6.1 Hz, 1H), 3.22 (s, 3H), 2.10–1.97 (m, 1H), 1.93–1.77 (m, 1H), 1.40–1.11 (m, 4H), 0.87–0.77 (m, 3H).

^{13}C-NMR (75 MHz, CDCl$_3$) δ (ppm) 149.0, 141.2, 136.7, 132.5, 130.5, 129.9, 127.3, 125.2, 122.8, 122.2, 121.0, 120.8, 117.8, 76.2, 56.9, 35.4, 28.1, 22.5, 14.0.

HRMS (ESI+) m/z calculated for $C_{19}H_{22}BrN_2O$ $[M+H]^+$: 373.0910, found $[M+H]^+$: 373.0921.

2-(3-Fluorophenyl)-3-(1-methoxypentyl)-2*H*-indazole (2d)

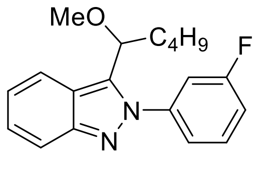

Eluent: hexane/EtOAc, 20:1. White solid, 91% yield (20 mg), m.p. = 55-56 ^oC.

^{1}H-NMR (300 MHz, CDCl$_3$) δ (ppm) 7.88 (dt, *J* = 8.5, 1.2 Hz, 1H), 7.74 (dd, *J* = 8.8, 1.1 Hz, 1H), 7.59–7.46 (m, 1H), 7.40–7.33 (m, 2H), 7.33–7.18 (m, 2H), 7.18–7.07 (m, 1H), 4.62 (dd, *J* = 7.8, 6.1 Hz, 1H), 3.23 (d, *J* = 0.5 Hz, 3H), 2.12–1.98 (m, 1H), 1.89–1.78 (m, 1H), 1.36–1.15 (m, 4H), 0.81 (t, *J* = 6.9 Hz, 3H).

^{19}F-NMR (282 MHz, CDCl$_3$) δ (ppm) -109.57 (td, *J* = 8.6, 6.1 Hz).

^{13}C-NMR (101 MHz, CDCl$_3$) δ (ppm) 162.7 (d, *J* = 249.2 Hz), 149.1, 141.5 (d, *J* = 9.8 Hz), 136.5, 130.6 (d, *J* = 9.0 Hz), 127.2, 122.4 (d, *J* = 3.3 Hz), 122.2, 121.0, 120.9, 117.9, 116.5 (d, *J* = 21.0 Hz), 114.5 (d, *J* = 24.3 Hz), 76.3, 56.9, 35.4, 28.1, 22.5, 14.0.

HRMS (ESI+) m/z calculated for $C_{19}H_{22}FN_2O$ $[M+H]^+$: 313.1711, found $[M+H]^+$: 313.1719.

2-(2-Fluorophenyl)-3-(1-methoxypentyl)-2*H*-indazole (2e)

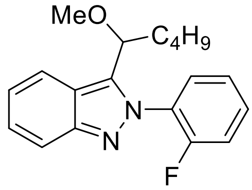

Eluent: hexane/EtOAc, 20:1. Colorless oil, 96% yield (22 mg).

^{1}H-NMR (300 MHz, $CDCl_3$) δ (ppm) 7.92–7.88 (m, 1H), 7.76–7.72 (m, 1H), 7.59–7.46 (m, 2H), 7.38–7.30 (m, 2H), 7.31–7.23 (m, 1H), 7.15–7.09 (m, 1H), 4.44–4.38 (m, 1H), 3.22 (s, 3H), 2.03–1.94 (m, 1H), 1.86–1.74 (m, 1H), 1.33–1.14 (m, 4H), 0.80 (t, *J* = 6.9 Hz, 3H).

^{19}F-NMR (282 MHz, $CDCl_3$) δ (ppm) -119.63 – -120.06 (m).

^{13}C-NMR (75 MHz, $CDCl_3$) δ (ppm) 158.7, 155.3, 149.5, 138.3, 131.7 (d, *J* = 7.9 Hz), 129.7, 127.1, 124.8 (d, *J* = 4.2 Hz), 121.9, 121.1, 119.9, 117.9, 116.8 (d, *J* = 19.8 Hz), 76.7, 57.2, 57.1, 35.9, 27.9, 22.4, 13.9.

HRMS (ESI+) m/z calculated for $C_{19}H_{22}FN_2O$ $[M+H]^+$: 313.1711, found $[M+H]^+$: 313.1714.

4-[3-(1-Methoxypentyl)-2*H*-indazol-2-yl]benzonitrile (2f)

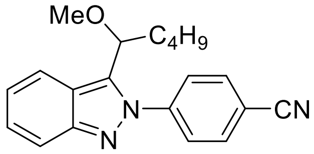

Eluent: hexane/EtOAc, 5:1. Orange solid, 57% yield (brsm, 14 mg), m.p. = 114-115 ^oC.

^{1}H NMR (400 MHz, $CDCl_3$) δ (ppm) 7.90–7.81 (m, 3H), 7.81–7.76 (m, 2H), 7.73 (dt, *J* = 8.8, 1.0 Hz, 1H), 7.37 (ddd, *J* = 8.8, 6.6, 1.1 Hz, 1H), 7.14 (ddd, *J* = 8.5, 6.6, 0.9 Hz, 1H), 4.65 (dd, *J* = 7.8, 6.3 Hz, 1H), 3.25 (s, 3H), 2.10–1.96 (m, 1H), 1.85–1.71 (m, 1H), 1.33–1.14 (m, 4H), 0.80 (t, *J* = 7.1 Hz, 3H).

^{13}C-NMR (101 MHz, $CDCl_3$) δ (ppm) 149.4, 143.9, 136.4, 133.3, 127.6, 127.3, 122.7, 121.8, 120.8, 118.0, 118.0, 113.1, 75.9, 56.9, 35.0, 28.1, 22.4, 13.9.

HRMS (ESI+) m/z calculated for $C_{20}H_{22}N_3O$ $[M+H]^+$: 320.1757, found $[M+H]^+$: 320.1758.

3-(1-Methoxypentyl)-2-(3-nitrophenyl)-2*H*-indazole (2g)

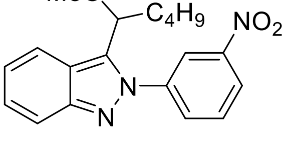

Eluent: hexane/EtOAc, 5:1. Colorless oil, 90% yield (27 mg).

^{1}H-NMR (300 MHz, $CDCl_3$) δ (ppm) 8.51 (t, *J* = 2.2 Hz, 1H), 8.41–8.37 (m, 1H), 8.01–7.98 (m, 1H), 7.91–7.82 (m, 1H), 7.81–7.69 (m, 2H), 7.43–7.32 (m, 1H), 7.21–7.09 (m, 1H), 4.66 (dd, *J* = 7.7, 6.3 Hz, 1H), 3.27 (d, *J* = 0.7 Hz, 3H), 2.14–1.95 (m, 1H), 1.91–1.73 (m, 1H), 1.39–1.12 (m, 4H), 0.80 (t, *J* = 6.9 Hz, 3H).

^{13}C-NMR (101 MHz, $CDCl_3$) δ (ppm) 149.3, 148.5, 141.4, 136.6, 132.3, 130.3, 127.7, 123.9, 122.7, 121.9, 121.6, 120.8, 117.9, 76.1, 56.9, 35.1, 28.1, 22.4, 13.9.

HRMS (ESI+) m/z calculated for $C_{19}H_{21}N_3NaO_3$ $[M+Na]^+$: 362.1475, found $[M+Na]^+$: 362.1475.

1-[4-(3-(1-Methoxypentyl)-2*H*-indazol-2-yl)phenyl]ethan-1-one (2h)

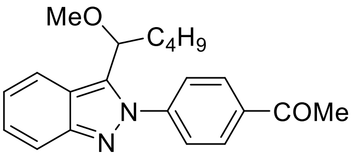

Eluent: hexane/EtOAc, 10:1. Colorless oil, 95% yield (29 mg).

^{1}H-NMR (300 MHz, $CDCl_3$) δ (ppm) 8.19–8.09 (m, 2H), 7.88 (dt, *J* = 8.6, 1.1 Hz, 1H), 7.79–7.63 (m, 3H), 7.36 (ddd, *J* = 8.8, 6.6, 1.1 Hz, 1H), 7.13 (ddd, *J* = 8.6, 6.6, 0.9 Hz, 1H), 4.64 (dd, *J* = 7.8, 6.1 Hz, 1H), 3.23 (s, 3H), 2.69 (s, 3H), 2.14–1.96 (m, 1H), 1.91–1.75 (m, 1H), 1.40–1.09 (m, 4H), 0.80 (t, *J* = 6.9 Hz, 3H).

^{13}C-NMR (126 MHz, $CDCl_3$) δ (ppm) 197.0, 149.2, 143.9, 137.4, 136.6, 129.4, 127.4, 126.8, 122.4, 121.2, 121.0, 117.9, 76.3, 56.9, 35.4, 28.1, 26.9, 22.5, 14.0.

HRMS (ESI+) m/z calculated for $C_{21}H_{25}N_2O_2$ $[M+H]^+$: 337.1911, found $[M+H]^+$: 337.1911.

Methyl 4-[3-(1-methoxypentyl)-2*H*-indazol-2-yl]benzoate (2i)

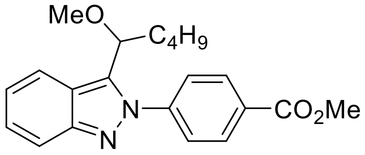

Eluent: hexane/EtOAc, 20:1. Colorless oil, 92% yield (29 mg).

^{1}H-NMR (300 MHz, $CDCl_3$) δ (ppm) 8.28–8.18 (m, 2H), 7.88 (dt, *J* = 8.5, 1.1 Hz, 1H), 7.74 (dt, *J* = 8.8, 1.0 Hz, 1H), 7.69–7.60 (m, 2H), 7.35 (ddd, *J* = 8.8, 6.6, 1.1 Hz, 1H), 7.12 (ddd, *J* = 8.5, 6.6, 0.9 Hz, 1H), 4.62 (dd, *J* = 7.8, 6.1 Hz, 1H), 3.98 (s, 3H), 3.23 (s, 3H), 2.11–1.97 (m, 1H), 1.91–1.75 (m, 1H), 1.37–1.09 (m, 4H), 0.80 (t, *J* = 6.9 Hz, 3H).

^{13}C-NMR (75 MHz, $CDCl_3$) δ (ppm) 166.2, 149.2, 143.8, 136.6, 130.9, 130.7, 127.3, 126.6, 122.3, 121.1, 121.0, 117.9, 76.3, 56.9, 52.6, 35.4, 28.1, 22.5, 14.0.

HRMS (ESI+) m/z calculated for $C_{21}H_{24}N_2NaO_3$ $[M+Na]^+$: 375.1679, found $[M+Na]^+$: 375.1681.

2-(2-Bromo-4-methylphenyl)-3-(1-methoxypentyl)-2*H*-indazole (2j)

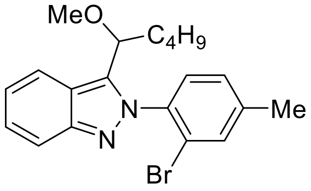

Eluent: hexane/EtOAc, 20:1. Colorless oil, 71% yield (26 mg).

^{1}H-NMR (300 MHz, CDCl$_3$) Mixture of rotamers, δ (ppm) 7.84–7.81 (m, 2H), 7.67 (dd, *J* = 8.9, 4.4 Hz, 2H), 7.52–7.51 (m, 2H), 7.29–7.18 (m, 6H), 7.04 (ddd, *J* = 9.2, 6.7, 1.5 Hz, 2H), 4.23 (ddd, *J* = 13.5, 8.4, 4.5 Hz, 2H), 3.26 (s, 4H), 3.11 (s, 2H), 2.39 (s, 6H), 2.07–1.96 (m, 1H), 1.89–1.78 (m, 2H), 1.62 (ddd, *J* = 14.0, 7.6, 3.0 Hz, 1H), 1.29–1.11 (m, 8H), 0.75 (dt, *J* = 14.2, 7.2 Hz, 6H).

^{13}C-NMR (101 MHz, CDCl$_3$) δ (ppm) 149.1, 149.0, 142.1, 142.1, 137.9, 137.6, 136.5, 134.1, 134.0, 129.5, 129.2, 128.9, 128.8, 127.0, 127.0, 122.0, 121.9, 121.3, 121.3, 120.0, 119.8, 118.0, 117.9, 77.0, 76.6, 58.0, 57.0, 36.3, 35.3, 29.8, 28.3, 28.0, 22.5, 22.5, 21.2, 14.0, 14.0.

HRMS (ESI+) m/z calculated for $C_{20}H_{24}BrN_2O$ [M+H]$^+$: 387.1067, found [M+H]$^+$: 387.1067.

Dimethyl 5-[3-(1-methoxypentyl)-2*H*-indazol-2-yl]isophthalate (2k)

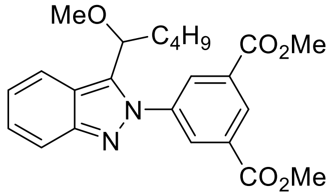

Eluent: hexane/EtOAc, 5:1. Yellow solid, 82% yield (31 mg), m.p. = 93-94 ^{o}C.

^{1}H-NMR (300 MHz, CDCl$_3$) δ (ppm) 8.83 (td, *J* = 1.6, 0.5 Hz, 1H), 8.43 (dd, *J* = 1.6, 0.5 Hz, 2H), 7.88 (dt, *J* = 8.5, 1.0 Hz, 1H), 7.74 (dt, *J* = 8.8, 0.9 Hz, 1H), 7.36 (ddd, *J* = 8.8, 6.6, 1.1 Hz, 1H), 7.19–7.08 (m, 1H), 4.60 (dd, *J* = 7.6, 6.2 Hz, 1H), 3.98 (s, 6H), 3.24 (s, 3H), 2.12–1.97 (m, 1H), 1.92–1.81 (m, 1H), 1.36–1.15 (m, 4H), 0.81 (t, *J* = 7.0 Hz, 3H).

^{13}C-NMR (75 MHz, CDCl$_3$) δ (ppm) 165.2, 149.2, 140.7, 136.8, 131.9, 131.5, 131.1, 127.5, 122.4, 121.1, 121.0, 117.9, 76.4, 57.0, 52.9, 35.6, 28.1, 22.5, 14.0.

HRMS (ESI+) m/z calculated for $C_{23}H_{27}N_2O_5$ [M+H]$^+$: 411.1914, found [M+H]$^+$: 411.1911.

3-(1-Methoxypentyl)-2-(perfluorophenyl)-2*H*-indazole (2l)

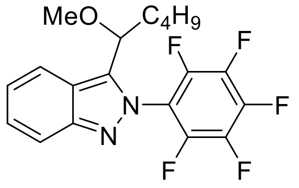

Eluent: hexane/EtOAc, 20:1. Colorless oil, 92% yield (35 mg).

^{1}H-NMR (300 MHz, CDCl$_3$) δ (ppm) 7.82 (dt, *J* = 8.7, 1.1 Hz, 1H), 7.71 (dt, *J* = 8.9, 0.9 Hz, 1H), 7.38 (ddd, *J* = 8.9, 6.6, 1.1 Hz, 1H), 7.15 (ddd, *J* = 8.6, 6.6, 0.9 Hz, 1H), 4.50–4.37 (m, 1H), 3.22 (s, 3H), 2.02–1.88 (m, 1H), 1.79–1.66 (m, 1H), 1.31–1.22 (m, 4H), 0.84 (t, *J* = 6.9 Hz, 3H).

^{19}F-NMR (282 MHz, CDCl$_3$) δ (ppm) -142.46 (dtd, *J* = 22.7, 7.0, 2.7 Hz), -143.45 (dt, *J* = 23.3, 7.3 Hz), -148.49 (tt, *J* = 21.5, 2.9 Hz), -159.02 (dtd, *J* = 64.7, 22.0, 7.0 Hz).

^{13}C-NMR (101 MHz, CDCl$_3$) δ (ppm) 150.3, 139.2, 128.0, 122.9, 120.7, 120.6, 118.1, 76.5, 57.4, 57.4, 57.4, 35.6, 27.9, 22.5, 13.9.

HRMS (ESI+) m/z calculated for $C_{19}H_{17}F_5N_2ONa$ [M+Na]$^+$: 407.1153, found [M+Na]$^+$: 407.1157.

3-(1-Methoxypentyl)-2-(*p*-tolyl)-2*H*-indazole (2m)

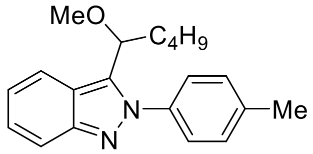

Eluent: hexane/EtOAc, 20:1. Colorless oil, 91% yield (22 mg).

^{1}H-NMR (400 MHz, CDCl$_3$) δ (ppm) 7.89 (dt, *J* = 8.5, 1.1 Hz, 1H), 7.74 (dt, *J* = 8.8, 0.9 Hz, 1H), 7.43–7.28 (m, 5H), 7.10 (ddd, *J* = 8.5, 6.6, 0.9 Hz, 1H), 4.57 (dd, *J* = 7.7, 6.1 Hz, 1H), 3.20 (s, 3H), 2.46 (s, 3H), 2.11–1.95 (m, 1H), 1.94–1.78 (m, 1H), 1.38–1.10 (m, 4H), 0.81 (t, *J* = 6.9 Hz, 3H).

^{13}C-NMR (101 MHz, CDCl$_3$) δ (ppm) 148.8, 139.5, 137.6, 136.6, 129.8, 126.8, 126.5, 121.7, 121.0, 120.4, 117.8, 76.41, 56.9, 35.6, 28.1, 22.5, 21.4, 14.0.

HRMS (ESI+) m/z calculated for $C_{20}H_{25}N_2O$ [M+H]$^+$: 309.1961, found [M+H]$^+$: 309.1967.

4-[3-(1-Methoxypentyl)-2*H*-indazol-2-yl]-*N*,*N*-dimethylaniline (2n)

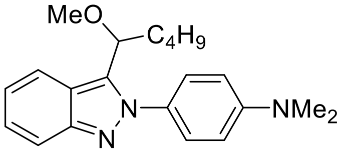

Eluent: hexane/EtOAc, 5:1. Yellow solid, 56% yield (brsm, 15 mg), m.p. = 97-98 ^{o}C.

¹H-NMR (400 MHz, CDCl₃) δ (ppm) 7.88 (dt, *J* = 8.5, 1.1 Hz, 1H), 7.73 (dt, *J* = 8.8, 1.0 Hz, 1H), 7.36–7.27 (m, 3H), 7.08 (ddd, *J* = 8.5, 6.6, 0.9 Hz, 1H), 6.82–6.75 (m, 2H), 4.57 (dd, *J* = 7.7, 6.2 Hz, 1H), 3.19 (s, 3H), 3.05 (s, 6H), 2.11–1.98 (m, 1H), 1.92–1.82 (m, 1H), 1.31–1.19 (m, 4H), 0.82 (t, *J* = 7.0 Hz, 3H).

¹³C-NMR (101 MHz, CDCl₃) δ (ppm) 150.8, 148.6, 136.6, 129.0, 127.3, 126.5, 121.5, 121.0, 120.27, 117.7, 112.0, 76.5, 56.8, 40.7, 35.7, 28.1, 22.6, 14.0.

HRMS (ESI+) m/z calculated for $C_{21}H_{28}N_3O$ $[M+H]^+$: 338.2227, found $[M+H]^+$: 338.2227.

***N*-[4-(3-(1-Methoxypentyl)-2*H*-indazol-2-yl)phenyl]-*N*-methylacetamide (2o)**

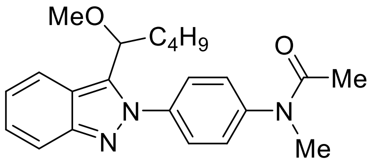

Eluent: hexane/EtOAc, 1:1. White solid, 99% yield (37 mg), m.p. = 84-85 ^oC.

¹H-NMR (300 MHz, CDCl₃) δ (ppm) 7.87 (dt, *J* = 8.6, 1.1 Hz, 1H), 7.73 (dt, *J* = 8.8, 1.0 Hz, 1H), 7.62 (d, *J* = 8.3 Hz, 2H), 7.44–7.32 (m, 3H), 7.13 (ddd, *J* = 8.6, 6.6, 0.9 Hz, 1H), 4.65 (dd, *J* = 7.7, 6.3 Hz, 1H), 3.34 (s, 3H), 3.25 (s, 3H), 2.14–1.91 (m, 4H), 1.88–1.77 (m, 1H), 1.34–1.14 (m, 4H), 0.80 (t, *J* = 6.9 Hz, 3H).

¹³C-NMR (101 MHz, CDCl₃) δ (ppm) 170.3, 148.9, 145.3, 139.4, 136.3, 127.9, 127.1, 122.1, 121.0, 120.8, 117.7, 76.0, 56.8, 37.3, 35.1, 27.9, 22.6, 22.3, 13.8.

HRMS (ESI+) m/z calculated for $C_{22}H_{28}N_3O_2$ $[M+H]^+$: 366.2176, found $[M+H]^+$: 366.2185.

3-(1-Methoxypentyl)-2-(4-methoxyphenyl)-2*H*-indazole (2p)

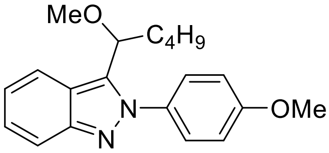

Eluent: hexane/EtOAc, 5:1. Yellow solid, 85% yield (32 mg), m.p. = 96-97 ^oC.

¹H-NMR (400 MHz, CDCl₃) δ (ppm) 7.88 (dt, *J* = 8.5, 1.0 Hz, 1H), 7.73 (dt, *J* = 8.7, 1.0 Hz, 1H), 7.45–7.38 (m, 2H), 7.33 (ddd, *J* = 8.8, 6.6, 1.1 Hz, 1H), 7.10 (ddd, *J* = 8.5, 6.6, 0.9 Hz, 1H), 7.06–7.00 (m, 2H), 4.56 (dd, *J* = 7.7, 6.2 Hz, 1H), 3.90 (s, 3H), 3.21 (s, 3H), 2.10–1.97 (m, 1H), 1.88–1.79 (m, 1H), 1.33–1.16 (m, 4H), 0.81 (t, *J* = 7.0 Hz, 3H).

¹³C-NMR (101 MHz, CDCl₃) δ (ppm) 160.2, 148.8, 136.6, 133.1, 127.9, 126.8, 121.8, 121.0, 120.5, 117.8, 114.4, 76.5, 56.9, 55.8, 35.5, 28.1, 22.5, 14.0.

HRMS (ESI+) m/z calculated for $C_{20}H_{25}N_2O_2$ $[M+H]^+$: 325.1911, found $[M+H]^+$: 325.1912.

4-[3-(1-Methoxypentyl)-2*H*-indazol-2-yl]phenyl acetate (2q)

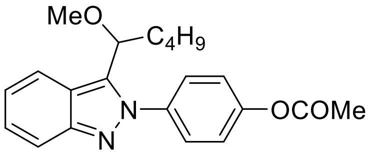

Eluent: hexane/EtOAc, 5:1. White solid, 92% yield (34 mg), m.p. = 152 ^oC (Decomposition).

^{1}H-NMR (400 MHz, CDCl$_3$) δ (ppm) 7.88 (dt, *J* = 8.4, 1.0 Hz, 1H), 7.73 (dt, *J* = 8.8, 1.0 Hz, 1H), 7.58–7.50 (m, 2H), 7.39–7.27 (m, 3H), 7.12 (ddd, *J* = 8.6, 6.6, 0.9 Hz, 1H), 4.61 (dd, *J* = 7.8, 6.1 Hz, 1H), 3.22 (s, 3H), 2.35 (s, 3H), 2.12–1.98 (m, 1H), 1.89–1.79 (m, 1H), 1.31–1.19 (m, 4H), 0.81 (t, *J* = 7.0 Hz, 3H).

^{13}C-NMR (101 MHz, CDCl$_3$) δ (ppm) 169.1, 151.2, 148.9, 137.5, 136.7, 127.7, 127.1, 122.5, 122.0, 121.1, 120.7, 117.8, 76.3, 56.9, 35.5, 28.1, 22.5, 21.3, 14.0.

HRMS (ESI+) m/z calculated for $C_{21}H_{25}N_2O_3$ $[M+H]^+$: 353.1860, found $[M+H]^+$: 353.1861.

6-Chloro-3-(1-methoxypentyl)-2-phenyl-2*H*-indazole (2r)

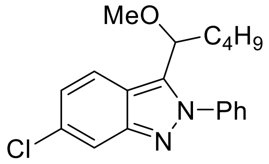

Eluent: hexane/EtOAc, 20:1. Colorless oil, 88% yield (29 mg).

^{1}H-NMR (300 MHz, CDCl$_3$) δ (ppm) 7.84 (dd, *J* = 9.0, 0.8 Hz, 1H), 7.72 (dt, *J* = 1.6, 0.8 Hz, 1H), 7.59–7.52 (m, 3H), 7.53–7.44 (m, 2H), 7.08–7.04 (m, 1H), 4.60–4.49 (m, 1H), 3.21 (d, *J* = 0.7 Hz, 3H), 2.07–1.95 (m, 1H), 1.87–1.75 (m, 1H), 1.32–1.12 (m, 4H), 0.85–0.76 (m, 3H).

^{13}C-NMR (75 MHz, CDCl$_3$) δ (ppm) 149.0, 139.7, 137.5, 132.9, 129.7, 129.4, 126.6, 123.4, 122.5, 118.9, 116.7, 76.37, 57.0, 35.7, 27.9, 22.4, 14.0.

HRMS (ESI+) m/z calculated for $C_{19}H_{22}ClN_2O$ $[M+H]^+$: 329.1415, found $[M+H]^+$: 329.1419.

3-(1-Methoxypentyl)-2-phenyl-5-(trifluoromethyl)-2*H*-indazole (2s)

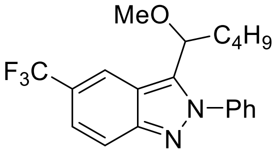

Eluent: hexane/EtOAc, 20:1. White solid, 88% yield (28 mg), m.p. = 66-67 ^oC.

^{1}H-NMR (300 MHz, CDCl$_3$) δ (ppm) 8.36–8.23 (m, 1H), 7.85–7.81 (m, 1H), 7.63–7.45 (m, 6H), 4.59 (dd, *J* = 7.9, 5.8 Hz, 1H), 3.24 (s, 3H), 2.10–1.99 (m, 1H), 1.87–1.75 (m, 1H), 1.37–1.16 (m, 4H), 0.81 (t, *J* = 7.0 Hz, 3H).

^{19}F-NMR (282 MHz, CDCl$_3$) δ (ppm) -60.36 (s).

^{13}C-NMR (75 MHz, CDCl$_3$) δ (ppm) 149.1, 139.6, 139.3, 129.9, 129.5, 126.5, 124.8 (q, *J* = 124.8 Hz), 124.0 (q, *J* = 32.0 Hz), 122.9 (q, *J* = 2.8 Hz), 120.1 (q, *J* = 4.8 Hz), 118.9, 118.9, 76.6, 57.2, 35.9, 28.0, 22.4, 13.9.

HRMS (ESI+) m/z calculated for $C_{20}H_{22}F_3N_2O$ [M+H]$^+$: 363.1679, found [M+H]$^+$: 363.1681.

Methyl 3-(1-methoxypentyl)-2-phenyl-2*H*-indazole-5-carboxylate (2t)

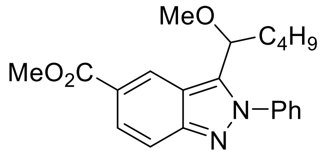

Eluent: hexane/EtOAc, 20:1. Colorless oil, 89% yield (30 mg).

^{1}H-NMR (300 MHz, CDCl$_3$) δ (ppm) 8.73 (dd, *J* = 1.6, 0.9 Hz, 1H), 7.97 (dd, *J* = 9.1, 1.6 Hz, 1H), 7.73 (dd, *J* = 9.1, 0.9 Hz, 1H), 7.61–7.46 (m, 5H), 4.59 (dd, *J* = 7.9, 5.9 Hz, 1H), 3.95 (s, 3H), 3.23 (s, 3H), 2.13–1.99 (m, 1H), 1.91–1.76 (m, 1H), 1.31–1.17 (m, 4H), 0.80 (t, *J* = 6.9 Hz, 3H).

^{13}C-NMR (101 MHz, CDCl$_3$) δ (ppm) 167.6, 150.1, 139.7, 139.7, 129.8, 129.5, 126.8, 126.6, 125.8, 123.9, 119.9, 117.7, 76.5, 57.2, 52.2, 35.8, 28.1, 22.4, 14.0.

HRMS (ESI+) m/z calculated for $C_{21}H_{25}N_2O_3$ [M+H]$^+$: 353.1860, found [M+H]$^+$: 353.1866.

3-(1-Methoxypentyl)-5-methyl-2-phenyl-2*H*-indazole (2u)

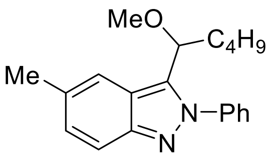

Eluent: hexane/EtOAc, 20:1. Colorless oil, 94% yield (28 mg).

^{1}H-NMR (300 MHz, CDCl$_3$) δ (ppm) 7.69–7.59 (m, 2H), 7.59–7.42 (m, 5H), 7.18 (dd, *J* = 8.8, 1.7 Hz, 1H), 4.55 (dd, *J* = 7.7, 6.2 Hz, 1H), 3.21 (s, 3H), 2.45 (s, 3H), 2.14–1.96 (m, 1H), 1.93–1.75 (m, 1H), 1.33–1.13 (m, 4H), 0.90–0.72 (m, 3H).

^{13}C-NMR (75 MHz, CDCl$_3$) δ (ppm) 147.9, 140.2, 135.5, 131.2, 129.8, 129.3, 129.3, 126.7, 120.8, 119.0, 117.5, 76.3, 56.9, 35.5, 28.1, 22.5, 22.0, 14.0.

HRMS (ESI+) m/z calculated for $C_{20}H_{25}N_2O$ [M+H]$^+$: 309.1961, found [M+H]$^+$: 309.1961.

3-(1-Methoxypentyl)-4-methyl-2-phenyl-2*H*-indazole (2v)

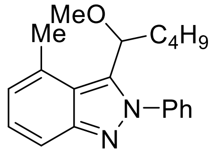

Eluent: hexane/EtOAc, 20:1. White solid, 94% yield (26 mg), m.p. = 61-62 ^oC.

^{1}H-NMR (400 MHz, CDCl$_3$) δ (ppm) 7.59 (dt, *J* = 8.7, 0.9 Hz, 1H), 7.55–7.51 (m, 3H), 7.50–7.46 (m, 2H), 7.24 (dd, *J* = 8.7, 6.7 Hz, 1H), 6.92 (dt, *J* = 6.8, 1.0 Hz, 1H), 4.76–4.68 (m, 1H), 3.22 (s, 3H), 2.73 (s, 3H), 2.04-1.96 (m, 1H), 1.78–1.68 (m, 1H), 1.34–1.08 (m, 4H), 0.80 (t, *J* = 7.2 Hz, 3H).

^{13}C-NMR (101 MHz, CDCl$_3$) δ (ppm) 149.5, 140.8, 136.9, 131.5, 129.5, 129.1, 127.3, 126.8, 123.2, 120.8, 115.6, 76.1, 56.7, 36.4, 28.4, 22.6, 22.4, 14.0.

HRMS (ESI+) m/z calculated for $C_{20}H_{25}N_2O$ [M+H]$^+$: 309.1961, found [M+H]$^+$: 309.1958.

3-(Methoxymethyl)-2-phenyl-2*H*-indazole (2w)

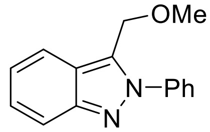

Eluent: hexane/EtOAc, 20:1. Yellow solid, 62% yield (16 mg), m.p. = 54-55 ^oC.

^{1}H-NMR (300 MHz, CDCl$_3$) δ (ppm) 7.80-7.76 (m, 4H), 7.61–7.46 (m, 3H), 7.35 (ddd, *J* = 8.7, 6.6, 1.0 Hz, 1H), 7.17 (ddd, *J* = 8.7, 6.6, 0.8 Hz, 1H), 4.76 (s, 2H), 3.44 (s, 3H).

^{13}C-NMR (101 MHz, CDCl$_3$) δ (ppm) 148.6, 139.9, 131.5, 129.3, 129.0, 126.9, 125.7, 123.1, 122.6, 119.8, 118.1, 63.3, 58.3.

HRMS (ESI+) m/z calculated for $C_{15}H_{15}N_2O$ [M+H]$^+$: 239.1179, found [M+H]$^+$: 239.1186.

3-(1-Methoxy-4-methylpentyl)-2-phenyl-2*H*-indazole (2x)

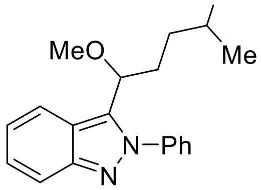

Eluent: hexane/EtOAc, 20:1. Colorless oil, 95% yield (29 mg).

^{1}H-NMR (300 MHz, CDCl$_3$) δ (ppm) 7.89 (dt, *J* = 8.5, 1.1 Hz, 1H), 7.76 (dt, *J* = 8.8, 0.9 Hz, 1H), 7.55.7-52 (m, 5H), 7.35 (ddd, *J* = 8.8, 6.6, 1.1 Hz, 1H), 7.15-7.09 (m, 1H), 4.57 (dd, *J* = 7.7, 6.2 Hz, 1H), 3.22 (s, 3H), 2.10–1.96 (m, 1H), 1.90-1.78 (m, 1H), 1.50–1.34 (m, 2H), 1.28–1.20 (m, 2H), 1.09–0.97 (m, 1H), 0.79 (dd, *J* = 6.6, 1.5 Hz, 6H).

^{13}C-NMR (75 MHz, CDCl$_3$) δ (ppm) 148.9, 140.1, 136.6, 129.5, 129.3, 126.9, 126.7, 121.9, 121.0, 120.6, 117.8, 76.7, 56.9, 35.0, 33.7, 27.9, 22.6, 22.5.

HRMS (ESI+) m/z calculated for $C_{20}H_{25}N_2O$ [M+H]$^+$: 309.1961, found [M+H]$^+$: 309.1966.

3-(2-Cyclohexyl-1-methoxyethyl)-2-phenyl-2*H*-indazole (2y)

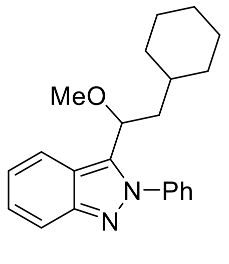

Eluent: hexane/EtOAc, 20:1. White solid, 90% yield (32 mg), m.p. = 83-84 ^{o}C.

^{1}H-NMR (500 MHz, CDCl$_3$) δ (ppm) 7.92-7.90 (m, 1H), 7.75 (d, *J* = 8.7 Hz, 1H), 7.57-7.50 (m, 5H), 7.38–7.31 (m, 1H), 7.11 (ddd, *J* = 8.6, 6.6, 1.0 Hz, 1H), 4.74–4.68 (m, 1H), 3.21 (s, 3H), 2.06–1.97 (m, 1H), 1.69–1.61 (m, 2H), 1.64–1.57 (m, 3H), 1.57–1.45 (m, 2H), 1.37-1.29 (m, 1H), 1.20–1.06 (m, 3H), 0.91–0.71 (m, 2H).

^{13}C-NMR (126 MHz, CDCl$_3$) δ (ppm) 148.9, 140.0, 137.0, 129.4, 129.3, 126.9, 126.7, 121.8, 121.1, 120.5, 117.8, 74.2, 56.9, 43.8, 34.2, 33.82, 32.7, 26.5, 26.2, 26.1.

HRMS (ESI+) m/z calculated for $C_{22}H_{27}N_2O$ [M+H]$^+$: 335.2118, found [M+H]$^+$: 335.2117.

2-(4-Bromophenyl)-3-[cyclopropyl(methoxy)methyl]-2*H*-indazole (2z)

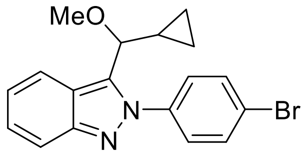

Eluent: hexane/EtOAc, 20:1. White solid, 96% yield (25 mg), m.p. = 123-124 ^{o}C.

^{1}H-NMR (400 MHz, CDCl$_3$) δ (ppm) 7.89 (dt, *J* = 8.7, 1.1 Hz, 1H), 7.73 (dt, *J* = 8.8, 1.0 Hz, 1H), 7.70–7.65 (m, 2H), 7.51–7.46 (m, 2H), 7.35 (ddd, *J* = 8.8, 6.6, 1.1 Hz, 1H), 7.13 (ddd, *J* = 8.5, 6.6, 0.9 Hz, 1H), 4.01 (d, *J* = 8.2 Hz, 1H), 3.29 (s, 3H), 1.46–1.36 (m, 1H), 0.73–0.65 (m, 1H), 0.46–0.37 (m, 2H), 0.12–0.03 (m, 1H).

^{13}C-NMR (101 MHz, CDCl$_3$) δ (ppm) 149.0, 139.3, 135.9, 132.5, 128.3, 127.1, 123.4, 122.4, 121.1, 120.9, 117.8, 79.8, 56.9, 15.7, 5.2, 2.4.

HRMS (ESI+) m/z calculated for $C_{18}H_{18}BrN_2O$ [M+H]$^+$: 357.0597, found [M+H]$^+$: 357.0597.

3-(4-Chloro-1-methoxybutyl)-2-phenyl-2*H*-indazole (2aa)

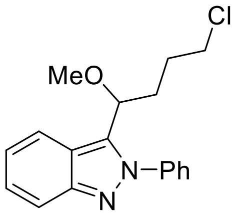

Eluent: hexane/EtOAc, 20:1. Colorless oil, 83% yield (20 mg).

^{1}H-NMR (300 MHz, CDCl$_3$) δ (ppm) 7.88 (dt, *J* = 8.5, 1.1 Hz, 1H), 7.76 (dt, *J* = 8.8, 1.0 Hz, 1H), 7.65–7.44 (m, 5H), 7.35 (ddd, *J* = 8.8, 6.6, 1.1 Hz, 1H), 7.13 (ddd, *J* = 8.5, 6.6, 0.9 Hz, 1H), 4.62 (dd, *J* = 8.4, 5.0 Hz, 1H), 3.47 (t, *J* = 6.4 Hz, 2H), 3.21 (s, 3H), 2.26-2.13 (m, 1H), 2.06–1.72 (m, 3H).

^{13}C-NMR (101 MHz, CDCl$_3$) δ (ppm) 148.9, 139.9, 135.8, 129.6, 129.4, 127.0, 126.6, 122.1, 120.9, 120.5, 117.9, 75.4, 56.3, 44.6, 33.1, 28.9.

HRMS (ESI+) m/z calculated for $C_{18}H_{20}ClN_2O$ [M+H]$^+$: 315.1259, found [M+H]$^+$: 315.1262.

3-Methoxy-3-(2-phenyl-2*H*-indazol-3-yl)propan-1-ol (2ab)

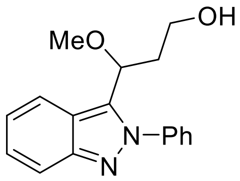

Eluent: hexane/EtOAc, 5:1. Colorless oil, 38% yield (11 mg).

^{1}H-NMR (300 MHz, CDCl$_3$) δ (ppm) δ 7.95–7.86 (m, 1H), 7.81–7.72 (m, 1H), 7.63–7.45 (m, 5H), 7.37 (ddd, *J* = 8.8, 6.6, 1.1 Hz, 1H), 7.14 (ddd, *J* = 8.6, 6.6, 0.9 Hz, 1H), 4.88 (dd, *J* = 9.3, 4.2 Hz, 1H), 3.83–3.68 (m, 2H), 3.22 (s, 3H), 2.43–2.33 (m, 1H), 2.06–1.97 (m, 1H).

^{13}C-NMR (101 MHz, CDCl$_3$) δ (ppm) 148.9, 139.9, 135.5, 129.6, 129.5, 127.1, 126.6, 122.3, 120.8, 120.7, 118.0, 75.6, 60.7, 57.0, 38.3.

HRMS (ESI+) m/z calculated for $C_{17}H_{19}N_2O_2$ [M+H]$^+$: 283.1441, found [M+H]$^+$: 283.1445.

3-(1-Methoxy-3-phenylpropyl)-2-phenyl-2*H*-indazole (2ac)

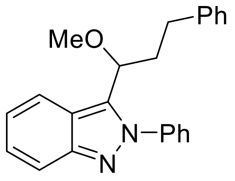

Eluent: hexane/EtOAc, 20:1. Colorless oil, 95% yield (25 mg).

^{1}H-NMR (300 MHz, CDCl$_3$) δ (ppm) 7.91 (dd, *J* = 8.5, 1.1 Hz, 1H), 7.76 (dd, *J* = 8.8, 1.0 Hz, 1H), 7.51–7.30 (m, 6H), 7.29–7.02 (m, 7H), 4.59 (dd, *J* = 8.7, 4.7 Hz, 1H), 3.21 (s, 3H), 2.81–2.58 (m, 2H), 2.49–2.38 (m 1H), 2.16–2.04 (m, 1H).

^{13}C-NMR (75 MHz, CDCl$_3$) δ (ppm) 148.9, 141.0, 139.8, 136.0, 129.2, 129.15, 1285, 128.5, 126.9, 126.3, 126.0, 121.9, 120.9, 120.6, 117.8, 75.0, 56.8, 37.2, 31.9.

HRMS (ESI+) m/z calculated for $C_{23}H_{22}N_2NaO$ $[M+Na]^+$: 365.1624, found $[M+Na]^+$: 365.1620.

3-[Methoxy(4-methoxyphenyl)methyl]-2-phenyl-2H-indazole (2ad)

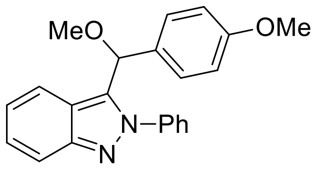

Eluent: hexane/EtOAc, 20:1. Yellow oil, 99% yield (47 mg).

^{1}H-NMR (300 MHz, CDCl$_3$) δ (ppm) 7.80 – 7.69 (m, 2H), 7.54 – 7.38 (m, 5H), 7.33 (dd, *J* = 9.0, 6.4 Hz, 1H), 7.24 – 7.14 (m, 2H), 7.08 (dd, *J* = 8.7, 6.6 Hz, 1H), 6.89 – 6.79 (m, 2H), 5.61 (s, 1H), 3.78 (s, 3H), 3.31 (s, 3H).

^{13}C-NMR (126 MHz, CDCl$_3$) δ (ppm) 159.5, 149.0, 140.0, 135.5, 131.4, 129.3, 129.2, 128.7, 126.8, 126.5, 122.3, 121.4, 120.8, 117.8, 114.0, 78.4, 57.2, 55.4.

HRMS (ESI+) m/z calculated for $C_{22}H_{21}N_2O_2$ $[M+H]^+$: 345.1598, found $[M+H]^+$: 345.1603.

Method B

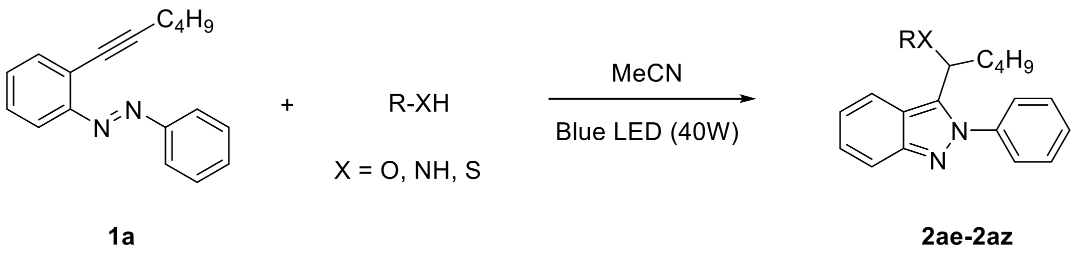

The corresponding alkynylazobenzene **1** (0.1 mmol, 1.0 equiv.) and RXH (0.15 mmol, 1.5 equiv.) were dissolved in MeCN (0.1 M) in a Schlenk tube (5 mL). The reaction mixture was irradiated with 40 W of blue LED at room temperature until completion. The solvents were removed under reduced pressure and the crude was purified by column chromatography using mixtures of hexane and ethyl acetate as eluent to yield the corresponding indazoles **2**.

3-(1-Ethoxypentyl)-2-phenyl-2*H*-indazole (2ae)

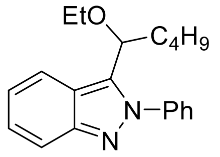

Eluent: hexane/EtOAc, 20:1. Colorless oil, 75% yield (28 mg).

^{1}H-NMR (400 MHz, CDCl$_3$) δ(ppm) 7.93 (dt, *J* = 8.5, 1.1 Hz, 1H), 7.74 (dt, *J* = 8.8, 1.0 Hz, 1H), 7.58–7.48 (m, 5H), 7.34 (ddd, *J* = 8.8, 6.6, 1.1 Hz, 1H), 7.10 (ddd, *J* = 8.5, 6.6, 0.9 Hz, 1H), 4.68 (dd, *J* = 7.8, 6.1 Hz, 1H), 3.44–3.25 (m, 2H), 2.09–1.99 (m, 1H), 1.84 (m, 1H), 1.38–1.15 (m, 4H), 1.13 (t, *J* = 7.0 Hz, 3H), 0.80 (t, *J* = 7.0 Hz, 3H).

^{13}C-NMR (126 MHz, CDCl$_3$) δ(ppm) 148.9, 140.1, 137.4, 129.4, 129.3, 129.3, 126.9, 126.7, 126.7, 121.7, 121.2, 120.6, 117.7, 74.6, 64.5, 35.9, 28.1, 22.5, 15.4, 14.0.

HRMS (ESI+) m/z calculated for $C_{20}H_{25}N_2O$ [M+H]$^+$: 309.1961, found [M+H]$^+$: 309.1967.

3-[1-(*tert*-Butoxy)pentyl]-2-phenyl-2*H*-indazole (2af)

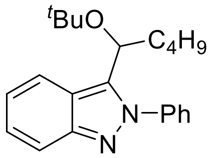

Eluent: hexane/EtOAc, 20:1. White solid, 51% yield (20 mg), m.p. = 115-116 ^{o}C.

^{1}H-NMR (300 MHz, CDCl$_3$) δ (ppm) 8.00 (dt, *J* = 8.5, 0.9 Hz, 1H), 7.69 (dt, *J* = 8.8, 0.9 Hz, 1H), 7.63–7.45 (m, 5H), 7.36–7.27 (m, 1H), 7.09–7.04 (m, 1H), 4.78 (dd, *J* = 9.2, 3.9 Hz, 1H), 2.05–1.93 (m, 1H), 1.79–1.63 (m, 1H), 1.53–1.40 (m, 1H), 1.33–1.22 (m, 3H), 0.98 (s, 9H), 0.85 (t, *J* = 7.1 Hz, 3H).

^{13}C-NMR (101 MHz, CDCl$_3$) δ (ppm) 149.0, 140.7, 140.4, 129.3, 129.3, 126.8, 126.4, 122.2, 121.1, 120.7, 117.5, 74.4, 67.5, 37.8, 28.4, 28.3, 22.5, 14.1.

HRMS (ESI+) m/z calculated for $C_{22}H_{28}N_2NaO$ [M+Na]$^+$: 359.2094, found [M+Na]$^+$: 359.2098.

3-(1-Isopropoxypentyl)-2-phenyl-2*H*-indazole (2ag)

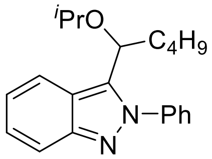

Eluent: hexane/EtOAc, 20:1. White solid, 69% yield (17 mg), m.p. = 107-108 ^{o}C.

^{1}H-NMR (400 MHz, CDCl$_3$) δ(ppm) 7.95 (dt, *J* = 8.5, 1.1 Hz, 1H), 7.73 (dt, *J* = 8.8, 1.0 Hz, 1H), 7.57–7.48 (m, 5H), 7.34 (ddd, *J* = 8.7, 6.6, 1.1 Hz, 1H), 7.10 (ddd, *J* = 8.6, 6.6, 0.9 Hz, 1H), 4.74 (dd, *J* = 8.2, 5.6 Hz, 1H), 3.43 (hept, *J* = 6.1 Hz, 1H), 2.07–1.97 (m, 1H), 1.84–1.76 (m, 1H), 1.39–1.30 (m, 1H), 1.28–1.15 (m, 4H), 1.02 (dd, *J* = 6.1, 2.5 Hz, 6H), 0.81 (t, *J* = 7.1 Hz, 3H).

^{13}C-NMR (101 MHz, CDCl$_3$) δ(ppm) 148.9, 140.1, 138.2, 129.5, 129.3, 127.0, 126.7, 121.6, 121.5, 120.6, 117.6, 71.5, 69.3, 36.3, 28.2, 23.4, 22.4, 21.1, 14.0.

HRMS (ESI+) m/z calculated for $C_{21}H_{27}N_2O$ [M+H]$^+$: 323.2118, found [M+H]$^+$: 323.2117.

3-[1-(But-3-en-1-yloxy)pentyl]-2-phenyl-2*H*-indazole (2ah)

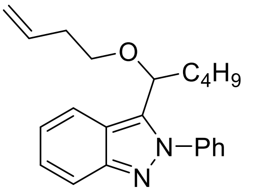

Eluent: hexane/EtOAc, 20:1. Colorless oil, 66% yield (19 mg).

^{1}H-NMR (300 MHz, CDCl$_3$) δ (ppm) 7.92 (dt, *J* = 8.5, 1.1 Hz, 1H), 7.74 (dt, *J* = 8.8, 1.0 Hz, 1H), 7.60–7.47 (m, 5H), 7.34 (ddd, *J* = 8.8, 6.6, 1.1 Hz, 1H), 7.11 (ddd, *J* = 8.5, 6.6, 0.9 Hz, 1H), 5.73 (ddt, *J* = 17.0, 10.2, 6.7 Hz, 1H), 5.13–4.88 (m, 2H), 4.68 (dd, *J* = 7.9, 5.9 Hz, 1H), 3.34 (ddt, *J* = 31.0, 8.9, 6.7 Hz, 2H), 2.30–2.22 (m, 2H), 2.12–2.00 (m, 1H), 1.89–1.77 (m, 1H), 1.34–1.15 (m, 4H), 0.81 (t, *J* = 7.0 Hz, 3H).

^{13}C-NMR (101 MHz, CDCl$_3$) δ (ppm) 148.9, 140.2, 137.1, 135.2, 129.34 129.3, 129.3, 126.9, 126.7, 121.7, 121.2, 120.6, 117.8, 116.5, 74.7, 68.5, 35.8, 34.3, 28.1, 22.4, 14.0.

HRMS (ESI+) m/z calculated for $C_{22}H_{27}N_2O$ [M+H]$^+$: 335.2118, found [M+H]$^+$: 335.2127.

2-Phenyl-3-[1-(prop-2-yn-1-yloxy)pentyl]-2*H*-indazole (2ai)

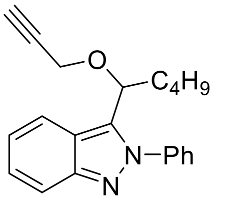

Eluent: hexane/EtOAc, 20:1. Colorless oil, 78% yield (22 mg).

^{1}H-NMR (300 MHz, CDCl$_3$) δ (ppm) 7.91–7.87 (m, 1H), 7.75 (dt, *J* = 8.7, 0.9 Hz, 1H), 7.60–7.48 (m, 5H), 7.34 (ddd, *J* = 8.9, 6.5, 1.1 Hz, 1H), 7.12 (ddd, *J* = 8.5, 6.6, 0.9 Hz, 1H), 5.02 (dd, *J* = 7.9, 6.0 Hz, 1H), 4.16 (dd, *J* = 16.0, 2.5 Hz, 1H), 3.90 (dd, *J* = 16.0, 2.4 Hz, 1H), 2.29 (t, *J* = 2.3 Hz, 1H), 2.11–1.97 (m, 1H), 1.93–1.75 (m, 1H), 1.31–1.17 (m, 4H), 0.80 (t, *J* = 7.1 Hz, 3H).

^{13}C-NMR (101 MHz, CDCl$_3$) δ (ppm) 149.0, 140.0, 135.6, 129.4, 129.2, 126.9, 126.8, 122.1, 120.9, 120.8, 117.9, 79.5, 74.9, 73.0, 55.9, 35.2, 27.9, 22.3, 13.9.

HRMS (ESI+) m/z calculated for $C_{21}H_{23}N_2O$ [M+H]$^+$: 319.1805, found [M+H]$^+$: 319.1818.

3-[1-(2-Fluoroethoxy)pentyl]-2-phenyl-2*H*-indazole (2aj)

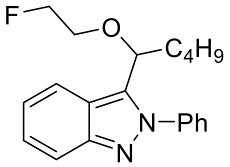

Eluent: hexane/EtOAc, 20:1. Yellow solid, 60% yield (27 mg), m.p. = 58-59 ^{o}C.

^{1}H-NMR (300 MHz, $CDCl_3$) δ (ppm) 7.91 (dd, *J* = 8.5, 1.1 Hz, 1H), 7.75 (dd, *J* = 8.8, 1.0 Hz, 1H), 7.54 (dd, *J* = 4.4, 2.3 Hz, 5H), 7.34 (ddd, *J* = 8.8, 6.6, 1.1 Hz, 1H), 7.12 (ddd, *J* = 8.5, 6.6, 0.9 Hz, 1H), 4.78 (dd, *J* = 7.8, 6.0 Hz, 1H), 4.57–4.53 (m, 1H), 4.41–4.37 (m, 1H), 3.63–3.54 (m, 1H), 3.49 (dd, *J* = 4.8, 3.5 Hz, 1H), 2.16–2.04 (m, 1H), 1.93–1.81 (m, 1H), 1.36–1.17 (m, 4H), 0.81 (t, *J* = 7.0 Hz, 3H).

^{19}F-NMR (282 MHz, $CDCl_3$) δ (ppm) -41.98 (tt, *J* = 47.7, 29.2 Hz).

^{13}C-NMR (101 MHz, $CDCl_3$) δ (ppm) 148.9, 140.1, 136.2, 129.5, 129.4, 127.0, 126.7, 122.1, 120.9, 120.7, 117.9, 83.0 (d, *J* = 169.8 Hz), 75.3, 68.1 (d, *J* = 19.8 Hz), 35.7, 28.0, 22.4, 14.0.

HRMS (ESI+) m/z calculated for $C_{20}H_{24}FN_2O$ $[M+H]^+$: 327.1867, found $[M+H]^+$: 327.1878.

3-[1-(3-Iodopropoxy)pentyl]-2-phenyl-2*H*-indazole (2ak)

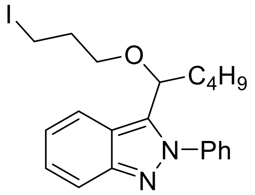

Eluent: hexane/EtOAc, 20:1. Colorless oil, 53% yield (21 mg).

^{1}H-NMR (400 MHz, $CDCl_3$) δ (ppm) 7.88 (dt, *J* = 8.5, 1.1 Hz, 1H), 7.79–7.70 (m, 1H), 7.56–7.50 (m, 5H), 7.34 (ddd, *J* = 8.8, 6.6, 1.1 Hz, 1H), 7.11 (ddd, *J* = 8.5, 6.6, 0.9 Hz, 1H), 4.66 (dd, *J* = 8.1, 5.8 Hz, 1H), 3.43–3.12 (m, 4H), 2.13–2.04 (m, 1H), 1.99–1.93 (m, 2H), 1.89–1.80 (m, 1H), 1.34–1.19 (m, 4H), 0.82 (t, *J* = 7.0 Hz, 3H).

^{13}C-NMR (101 MHz, $CDCl_3$) δ (ppm) 149.0, 140.0, 136.9, 129.5, 129.4, 126.9, 126.7, 121.9, 121.0, 120.5, 117.9, 75.1, 68.4, 35.8, 33.7, 28.1, 22.4, 14.0, 3.4.

HRMS (ESI+) m/z calculated for $C_{21}H_{26}IN_2O$ $[M+H]^+$: 449.1084, found $[M+H]^+$: 449.1088.

3-(1-Phenoxypentyl)-2-phenyl-2*H*-indazole (2al)

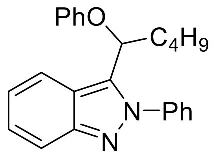

Eluent: hexane/EtOAc, 20:1. Yellow solid, 81% yield (32 mg), m.p. = 79-80 °C.

^{1}H-NMR (400 MHz, CDCl$_3$) δ (ppm) 7.97 (dt, *J* = 8.6, 1.1 Hz, 1H), 7.71 (dt, *J* = 8.8, 1.0 Hz, 1H), 7.61–7.48 (m, 3H), 7.49–7.37 (m, 2H), 7.31 (ddd, *J* = 8.7, 6.6, 1.1 Hz, 1H), 7.14–7.09 (m, 3H), 6.95–6.82 (m, 1H), 6.76–6.63 (m, 2H), 5.45 (dd, *J* = 8.3, 5.3 Hz, 1H), 2.41–2.27 (m, 1H), 2.11–1.99 (m, 1H), 1.54–1.28 (m, 4H), 0.85 (t, *J* = 7.2 Hz, 3H).

^{13}C-NMR (101 MHz, CDCl$_3$) δ (ppm) 157.8, 149.0, 140.0, 136.0, 129.6, 129.5, 129.4, 126.9, 126.6, 122.1, 121.9, 121.1, 120.6, 117.9, 116.7, 73.9, 35.9, 28.1, 22.4, 14.0.

HRMS (ESI+) m/z calculated for $C_{24}H_{25}N_2O$ [M+H]$^+$: 357.1961, found [M+H]$^+$: 357.1962.

3-[1-((1*S*,2*S*,4*R*)-Bicyclo[2.2.1]heptan-2-yl)methoxy)pentyl]-2-phenyl-2*H*-indazole y 3-[1-((1*S*,2*R*,4*R*)-Bicyclo[2.2.1]heptan-2-yl)methoxy)pentyl]-2-phenyl-2*H*-indazole (2am)

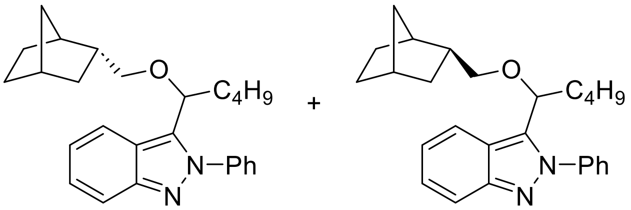

Eluent: hexane/EtOAc, 20:1. Colourless oil, 48% yield (19 mg).

^{1}H-NMR (300 MHz, CDCl$_3$) Mixture of diastereomers, δ (ppm) 7.97–7.87 (m, 1H), 7.73 (d, *J* = 8.9 Hz, 1H), 7.59–7.49 (m, 5H), 7.39–7.30 (m, 1H), 7.16–7.04 (m, 1H), 4.67–4.60 (m, 1H), 3.34–2.89 (m, 2H), 2.25–2.21 (m, 1H), 2.12–1.98 (m, 3H), 1.88–1.74 (m, 1H), 1.65–1.57 (m, 1H), 1.52–1.00 (m, 10H), 0.86–0.75 (m, 3H), 0.52–0.43 (m, 1H).

^{13}C-NMR (101 MHz, CDCl$_3$) δ (ppm) 149.0, 148.9, 140.2, 140.2, 137.6, 137.6, 129.3, 129.3, 129.3, 126.9, 126.9, 126.7, 126.7, 121.6, 121.6, 121.5, 121.5, 121.4, 121.3, 121.3, 120.5, 120.5, 117.7, 117.6, 74.9, 74.9, 74.8, 74.6, 73.5, 73.3, 71.5, 71.3, 42.2, 42.0, 40.0, 40.0, 39.8, 39.8, 38.8, 38.6, 38.6, 38.4, 36.8, 36.7, 36.2, 36.2, 35.9, 35.9, 35.9, 35.8, 35.4, 35.2, 34.5, 34.5, 34.0, 33.8, 30.1, 29.9, 29.9, 29.1, 29.0, 28.2, 28.1, 22.7, 22.5, 22.5, 22.4, 22.4, 14.0, 14.0.

HRMS (ESI+) m/z calculated for $C_{26}H_{32}N_2ONa$ [M+Na]$^+$: 411.2407, found [M+Na]$^+$: 411.2402.

3-[1-(((3*R*,5*R*,7*R*)-Adamantan-1-yl)methoxy)pentyl]-2-phenyl-2*H*-indazole (2an)

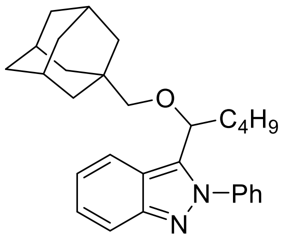

Eluent: hexane/EtOAc, 20:1. White solid, 43% yield (22 mg), m.p. = 92-93 ^{o}C.

^{1}H-NMR (300 MHz, CDCl$_3$) δ (ppm) 7.90 (dd, *J* = 8.6, 1.1 Hz, 1H), 7.73 (dd, *J* = 8.8, 1.0 Hz, 1H), 7.54–7.50 (m, 5H), 7.39–7.27 (m, 1H), 7.09 (dd, *J* = 8.5, 6.6 Hz, 1H), 4.57 (dd, *J* = 8.3, 5.5 Hz, 1H), 2.90 (d, *J* = 8.3 Hz, 1H), 2.75 (d, *J* = 8.3 Hz, 1H), 2.03 (d, *J* = 4.9 Hz, 1H), 1.95–1.91 (m, 3H), 1.77 (dt, *J* = 8.9, 4.7 Hz, 1H), 1.71–1.58 (m, 7H), 1.48 (d, *J* = 2.7 Hz, 6H), 1.27–1.17 (m, 4H), 0.81 (t, *J* = 7.0 Hz, 3H).

^{13}C-NMR (101 MHz, CDCl$_3$) δ (ppm) 148.9, 140.2, 137.7, 129.3, 129.3, 126.9, 126.7, 121.6, 121.5, 120.4, 117.6, 80.2, 75.3, 39.8, 37.3, 36.0, 34.2, 28.4, 28.2, 22.4, 14.0.

HRMS (ESI+) m/z calculated for $C_{29}H_{36}N_2NaO$ [M+Na]$^+$: 451.2720, found [M+Na]$^+$: 451.2722.

2-Phenyl-3-[(*S*)-1-(3-(((*R*)-2,5,7,8-tetramethyl-2-((4*R*,8*R*)-4,8,12-trimethyltridecyl)chroman-6-yl)oxy)propoxy)pentyl]-2*H*-indazole and 2-phenyl-3-[(*R*)-1-(3-(((*R*)-2,5,7,8-tetramethyl-2-((4*R*,8*R*)-4,8,12-trimethyltridecyl)chroman-6-yl)oxy)propoxy)pentyl]-2*H*-indazole (2ao)

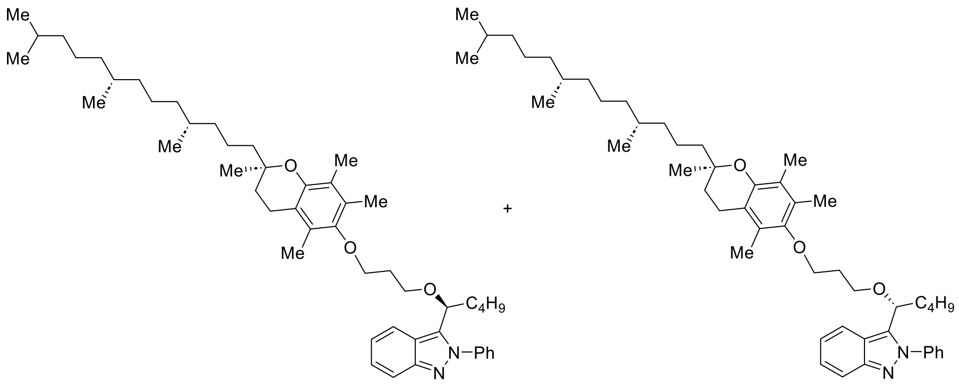

Eluent: hexane/EtOAc, 10:1. Colorless oil, 40% yield (31 mg).

^{1}H-NMR (400 MHz, CDCl$_3$) δ (ppm) 7.93 (d, *J* = 8.6 Hz, 1H), 7.75 (d, *J* = 8.8 Hz, 1H), 7.53 (s, 5H), 7.38–7.33 (m, 1H), 7.10 (dd, *J* = 8.5, 6.5 Hz, 1H), 4.72 (dd, *J* = 7.9, 6.0 Hz, 1H), 3.73 (dd, *J* = 10.7, 4.7 Hz, 1H), 3.66–3.58 (m, 1H), 3.54 (td, *J* = 7.3, 6.1, 3.2 Hz, 2H), 2.55 (t, *J* = 6.8 Hz, 2H), 2.15–1.95 (m, 11H), 1.78 (dt, *J* = 13.3, 6.8 Hz, 4H), 1.53 (dd, *J* = 13.2, 6.8 Hz, 2H), 1.45–1.02 (m, 26H), 0.93–0.77 (m, 15H).

^{13}C-NMR (101 MHz, CDCl$_3$) δ (ppm) 149.0, 148.2, 147.9, 140.1, 137.2, 129.4, 129.3, 128.5, 127.9, 126.9, 126.8, 126.5, 126.3, 126.2, 125.9, 125.2, 122.9, 121.8, 121.2, 120.5, 120.1, 118.7, 117.8,

117.6, 112.3, 75.0, 74.9, 69.8, 66.2, 41.8, 40.2, 40.2, 39.5, 37.7, 37.6, 37.6, 37.5, 37.5, 37.5, 37.4, 35.9, 32.9, 32.9, 32.8, 32.8, 32.4, 31.4, 31.4, 30.8, 29.8, 29.0, 28.2, 28.1, 25.0, 24.9, 24.6, 24.0, 22.9, 22.8, 22.8, 22.5, 21.2, 21.2, 20.8, 19.9, 19.8, 19.8, 19.8, 19.7, 14.0, 12.8, 11.9, 11.9.

HRMS (ESI+) m/z calculated for $C_{50}H_{75}N_2O_3$ [M+H]$^+$: 751.5772, found [M+H]$^+$: 751.5790.

(8*R*,9*S*,13*S*,14*S*)-13-Methyl-3-[3-((1-(2-phenyl-2*H*-indazol-3-yl)pentyl)oxy)propoxy]-6,7,8,9,11,12,13,14,15,16-decahydro-17*H*-cyclopenta[*a*]phenanthren-17-one (2ap)

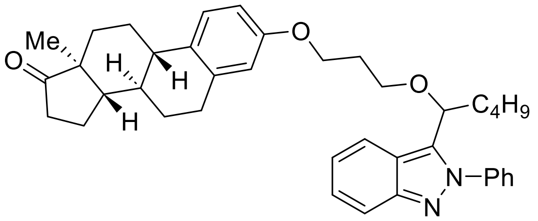

Eluent: hexane/EtOAc, 10:1. Colorless oil, 47% yield (25 mg).

^{1}H-NMR (400 MHz, CDCl$_3$) δ (ppm) 7.89 (dd, *J* = 8.6, 1.2 Hz, 1H), 7.73 (dd, *J* = 8.8, 1.1 Hz, 1H), 7.51 (q, *J* = 1.9 Hz, 5H), 7.38–7.29 (m, 1H), 7.16 (d, *J* = 8.6 Hz, 1H), 7.09–7.03 (m, 1H), 6.62 (dd, *J* = 8.6, 2.8 Hz, 1H), 6.54 (d, *J* = 2.7 Hz, 1H), 4.67 (dd, *J* = 7.9, 6.0 Hz, 1H), 4.00 (ddd, *J* = 8.7, 5.8, 2.3 Hz, 1H), 3.95–3.84 (m, 1H), 3.45 (t, *J* = 6.1 Hz, 2H), 2.92–2.81 (m, 2H), 2.58–2.35 (m, 2H), 2.23–1.77 (m, 9H), 1.60–1.41 (m, 5H), 1.33–1.11 (m, 5H), 0.91 (s, 3H), 0.79 (t, *J* = 6.9 Hz, 3H).

^{13}C-NMR (101 MHz, CDCl$_3$) δ (ppm) 157.0, 148.9, 140.1, 137.8, 137.1, 132.1, 129.4, 129.3, 126.9, 126.7, 126.4, 121.8, 121.1, 120.5, 117.8, 114.7, 114.6, 112.2, 112.2, 75.0, 65.7, 64.8, 50.6, 48.2, 44.1, 38.5, 36.0, 35.8, 31.7, 29.9, 29.8, 28.1, 26.7, 26.1, 22.4, 21.7, 14.0.

HRMS (ESI+) m/z calculated for $C_{39}H_{46}N_2NaO_3$ [M+Na]$^+$: 613.3401, found [M+Na]$^+$: 613.3402.

1-(2-Phenyl-2*H*-indazol-3-yl)pentan-1-ol (2aq)

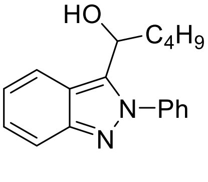

Eluent: hexane/EtOAc, 4:1. Colorless oil, 83% yield (31 mg).

^{1}H-NMR (300 MHz, CDCl$_3$) δ (ppm) 7.87 (d, *J* = 8.5 Hz, 1H), 7.67 (d, *J* = 8.8 Hz, 1H), 7.52–7.34 (m, 5H), 7.35–7.27 (m, 1H), 7.07 (dd, *J* = 8.5, 6.6 Hz, 1H), 4.97 (td, *J* = 7.3, 2.9 Hz, 1H), 3.00 (s, 1H), 2.12–1.87 (m, 2H), 1.33–1.09 (m, 4H), 0.80 (t, *J* = 6.8 Hz, 3H).

^{13}C-NMR (75 MHz, CDCl$_3$) δ(ppm) 148.8, 139.7, 138.2, 129.2, 129.2, 126.8, 126.4, 121.8, 121.1, 120.0, 117.7, 67.2, 36.8, 28.1, 22.4, 14.0.

HRMS (ESI+) m/z calculated for $C_{18}H_{21}N_2O$ [M+H]$^+$: 281.1648, found [M+H]$^+$: 281.1649.

1-(2-Phenyl-2*H*-indazol-3-yl)pentyl hexanoate (2ar)

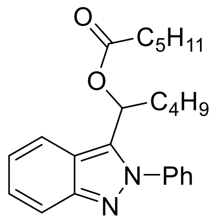

Eluent: hexane/EtOAc, 20:1. Colorless oil, 63% yield (26 mg).

^{1}H-NMR (400 MHz, $CDCl_3$) δ(ppm) 7.87 (dt, *J* = 8.5, 1.1 Hz, 1H), 7.74 (dd, *J* = 8.8, 1.0 Hz, 1H), 7.69–7.62 (m, 2H), 7.60–7.49 (m, 3H), 7.33 (ddd, *J* = 8.8, 6.6, 1.1 Hz, 1H), 7.12 (ddd, *J* = 8.5, 6.6, 0.9 Hz, 1H), 6.09 (dd, *J* = 8.0, 6.7 Hz, 1H), 2.33 (td, *J* = 7.5, 4.2 Hz, 2H), 2.15–2.01 (m, 1H), 1.96–1.87 (m, 1H), 1.63–1.55 (m, 2H), 1.30–1.11 (m, 8H), 0.84 (t, *J* = 7.0 Hz, 3H), 0.78 (t, *J* = 6.8 Hz, 3H).

^{13}C-NMR (101 MHz, $CDCl_3$) δ(ppm) 173.2, 148.9, 139.9, 135.1, 129.5, 129.4, 129.3, 126.7, 126.6, 122.1, 120.6, 120.4, 118.1, 69.2, 34.7, 34.3, 31.3, 27.6, 24.7, 22.4, 22.2, 14.0, 13.9.

HRMS (ESI+) m/z calculated for $C_{24}H_{31}N_2O_2$ $[M+H]^+$: 379.2380, found $[M+H]^+$: 379.2385.

1-(2-Phenyl-2*H*-indazol-3-yl)pentyl benzoate (2as)

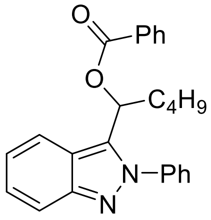

Eluent: hexane/EtOAc, 20:1. Colorless oil, 55% yield (24 mg).

^{1}H-NMR (400 MHz, $CDCl_3$) δ(ppm) 8.09–8.02 (m, 2H), 7.94 (dt, *J* = 8.5, 1.1 Hz, 1H), 7.78–7.67 (m, 3H), 7.61–7.54 (m, 4H), 7.45 (dd, *J* = 8.4, 7.1 Hz, 2H), 7.32 (ddd, *J* = 8.7, 6.6, 1.1 Hz, 1H), 7.12 (ddd, *J* = 8.5, 6.7, 0.9 Hz, 1H), 6.35 (dd, *J* = 7.9, 6.7 Hz, 1H), 2.31–2.18 (m, 1H), 2.13–2.02 (m, 1H), 1.26–1.20 (m, 4H), 0.80 (t, *J* = 6.9 Hz, 3H).

^{13}C-NMR (101 MHz, $CDCl_3$) δ(ppm) 165.8, 148.9, 140.0, 134.9, 133.4, 129.9, 129.8, 129.6, 129.4, 128.6, 126.8, 126.7, 122.3, 120.6, 120.5, 118.2, 69.9, 34.9, 27.7, 22.2, 14.0.

HRMS (ESI+) m/z calculated for $C_{25}H_{25}N_2O_2$ $[M+H]^+$: 385.1911, found $[M+H]^+$: 385.1912.

1-(2-Phenyl-2*H*-indazol-3-yl)pentyl 1-methylcyclohexane-1-carboxylate (2at)

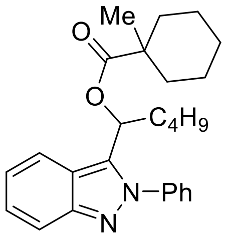

Eluent: hexane/EtOAc, 20:1. Colorless oil, 59% yield (25 mg).

^{1}H-NMR (300 MHz, CDCl$_3$) δ (ppm) 7.87 (dt, *J* = 8.5, 1.1 Hz, 1H), 7.74 (dt, *J* = 8.8, 1.0 Hz, 1H), 7.70–7.63 (m, 2H), 7.63–7.46 (m, 3H), 7.38–7.29 (m, 1H), 7.11 (ddd, *J* = 8.5, 6.6, 0.9 Hz, 1H), 6.10 (dd, *J* = 8.1, 6.6 Hz, 1H), 2.16–1.87 (m, 4H), 1.55–1.12 (m, 15H), 0.84–0.70 (m, 3H).

^{13}C-NMR (101 MHz, CDCl$_3$) δ (ppm) 177.0, 148.9, 140,0, 135.2, 129.4, 129.4, 126.8, 126.6, 121.9, 120.8, 120.4, 118.0, 68.9, 43.5, 35.7, 35.6, 34.9, 27.7, 25.8, 23.2, 23.2, 22.1, 13.9.

HRMS (ESI+) m/z calculated for $C_{26}H_{33}N_2O_2$ [M+H]$^+$: 405.2537, found [M+H]$^+$: 405.2548.

1-(2-Phenyl-2*H*-indazol-3-yl)pentyl (3r,5r,7r)-adamantane-1-carboxylate (2au)

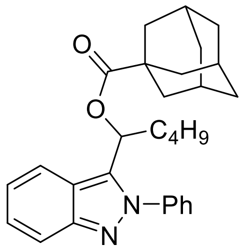

Eluent: hexane/EtOAc, 20:1. Colorless oil, 54% yield (29 mg).

^{1}H-NMR (300 MHz, CDCl$_3$) δ (ppm) 7.88 (dt, *J* = 8.5, 1.1 Hz, 1H), 7.74 (d, *J* = 8.6 Hz, 1H), 7.72–7.64 (m, 2H), 7.62–7.48 (m, 3H), 7.33 (ddd, *J* = 8.9, 6.6, 1.1 Hz, 1H), 7.12 (dd, *J* = 8.4, 6.6 Hz, 1H), 6.03 (dd, *J* = 8.2, 6.4 Hz, 1H), 2.14–1.98 (m, 4H), 1.88 (d, *J* = 2.9 Hz, 7H), 1.75–1.64 (m, 6H), 1.22–1.13 (m, 4H), 0.83–0.72 (m, 3H).

^{13}C-NMR (101 MHz, CDCl$_3$) δ (ppm) 176.9, 148.9, 140.0, 135.3, 129.4, 129.4, 126.7, 126.6, 121.9, 120.8, 120.3, 118.1, 69.1, 41.1, 39.0, 36.6, 34.9, 28.0, 27.7, 22.2, 13.9.

HRMS (ESI+) m/z calculated for $C_{29}H_{34}N_2NaO_2$ [M+Na]$^+$: 465.2512, found [M+Na]$^+$: 465.2511.

3-[1-(Ethylthio)pentyl]-2-phenyl-2*H*-indazole (2av)

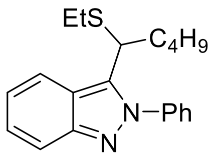

Eluent: hexane/EtOAc, 20:1. Yellow oil, 77% yield (27 mg).

^{1}H-NMR (300 MHz, CDCl$_3$) δ (ppm) 7.98 (dt, *J* = 8.6, 1.1 Hz, 1H), 7.73 (dt, *J* = 8.8, 1.0 Hz, 1H), 7.60–7.49 (m, 5H), 7.33 (ddd, *J* = 8.8, 6.6, 1.1 Hz, 1H), 7.10 (ddd, *J* = 8.6, 6.6, 1.0 Hz, 1H), 4.27 (dd, *J* = 8.8, 6.6 Hz, 1H), 2.34 (q, *J* = 7.4 Hz, 2H), 2.25–2.15 (m, 1H), 2.14–2.02 (m, 1H), 1.30–1.17 (m, 4H), 1.03 (t, *J* = 7.4 Hz, 3H), 0.83–0.79 (m, 3H).

^{13}C-NMR (75 MHz, CDCl$_3$) δ (ppm) 137.2, 131.1, 129.5, 129.3, 126.9, 126.8, 123.0, 121.4, 121.3, 119.9, 117.9, 40.6, 35.2, 30.1, 25.5, 22.4, 14.5, 13.9.

HRMS (ESI+) m/z calculated for $C_{20}H_{25}N_2S$ [M+H]$^+$: 325.1733, found [M+H]$^+$: 325.1723.

2-Phenyl-3-[1-(phenylthio)pentyl]-2*H*-indazole (2aw)

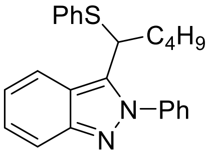

Eluent: hexane/EtOAc, 20:1. Yellow oil, 60% yield (22 mg).

^{1}H-NMR (400 MHz, CDCl$_3$) δ (ppm) 7.94 (dt, *J* = 8.6, 1.1 Hz, 1H), 7.72 (dt, *J* = 8.8, 1.0 Hz, 1H), 7.50–7.43 (m, 1H), 7.43–7.38 (m, 2H), 7.34 (ddd, *J* = 8.8, 6.6, 1.1 Hz, 1H), 7.30–7.22 (m, 2H), 7.19–7.11 (m, 4H), 7.11–7.07 (m, 1H), 7.07–7.02 (m, 2H), 4.44 (dd, *J* = 9.5, 6.1 Hz, 1H), 2.33–2.25 (m, 1H), 2.17–2.08 (m, 1H), 1.22–1.17 (m, 4H), 0.83–0.74 (m, 3H).

^{13}C-NMR (101 MHz, CDCl$_3$) δ (ppm) 149.0, 139.5, 136.4, 134.7, 133.1, 129.3, 129.1, 129.1, 128.6, 126.8, 126.7, 121.4, 121.4, 119.9, 118.1, 46.3, 35.1, 30.1, 22.3, 13.9.

HRMS (ESI+) m/z calculated for $C_{24}H_{25}N_2S$ [M+H]$^+$: 373.1733, found [M+H]$^+$: 373.1739.

2-[(1-(2-Phenyl-2*H*-indazol-3-yl)pentyl)thio]ethanol (2ax)

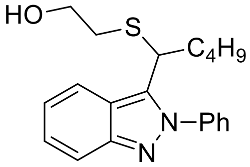

Eluent: hexane/EtOAc, 5:1. Colorless oil, 65% yield (31 mg).

^{1}H-NMR (300 MHz, CDCl$_3$) δ (ppm) 7.97 (d, *J* = 8.5 Hz, 1H), 7.73 (d, *J* = 8.7 Hz, 1H), 7.58 – 7.42 (m, 5H), 7.34 (dd, *J* = 8.6, 6.9 Hz, 1H), 7.11 (dd, *J* = 8.5, 6.5 Hz, 1H), 4.28 (dd, *J* = 8.8, 6.7 Hz, 1H), 3.37 (q, *J* = 5.3 Hz, 2H), 2.48 (q, *J* = 5.8 Hz, 2H), 2.29 – 1.99 (m, 3H), 1.30 – 1.14 (m, 4H), 0.81 (t, *J* = 6.7 Hz, 3H).

^{13}C-NMR (126 MHz, CDCl$_3$) δ (ppm) 149.1, 139.7, 136.7, 129.7, 129.4, 127.0, 126.7, 121.7, 121.1, 119.9, 118.1, 60.6, 40.7, 35.3, 34.7, 30.1, 22.4, 13.9.

HRMS (ESI+) m/z calculated for $C_{20}H_{25}N_2OS$ [M+H]$^+$: 341.1682, found [M+H]$^+$: 341.1687.

FTIR: 3309, 2954, 2926, 2857, 1625, 1507, 1513, 1500, 1455, 1379, 1265, 1071, 1045, 1023, 769, 734, 695.

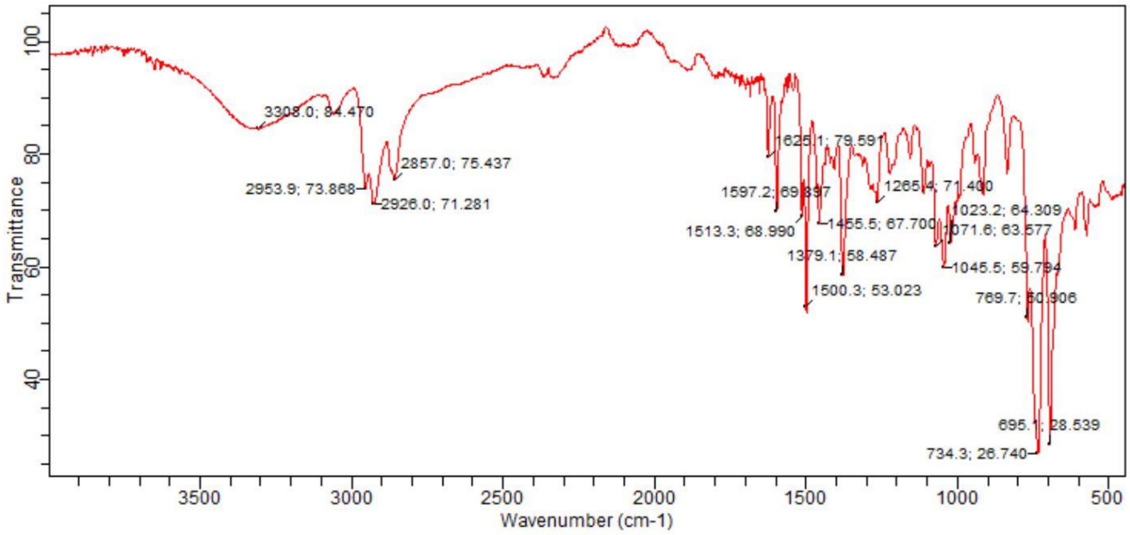

2-[(1-(2-Phenyl-2*H*-indazol-3-yl)pentyl)oxy]ethane-1-thiol (2ax´)

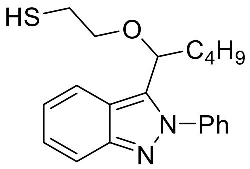

Eluent: hexane/EtOAc, 5:1. Colorless oil, 23% yield (11 mg).

^{1}H-NMR (300 MHz, CDCl$_3$) δ (ppm) 7.90 (dt, *J* = 8.6, 1.1 Hz, 1H), 7.74 (dt, *J* = 8.7, 0.9 Hz, 1H), 7.66–7.41 (m, 5H), 7.40–7.29 (m, 1H), 7.11 (ddd, *J* = 8.5, 6.6, 0.9 Hz, 1H), 4.71 (dd, *J* = 8.0, 5.9 Hz, 1H), 3.46 (dt, *J* = 9.4, 6.2 Hz, 1H), 3.36 (dt, *J* = 9.3, 6.5 Hz, 1H), 2.73–2.47 (m, 2H), 2.16–2.01 (m, 1H), 1.95–1.79 (m, 1H), 1.51 (t, *J* = 8.2 Hz, 1H), 1.25 (s, 4H), 0.82 (t, *J* = 7.0 Hz, 3H).

^{13}C-NMR (101 MHz, CDCl$_3$) δ (ppm) 149.0, 140.0, 136.6, 129.5, 129.4, 127.0, 126.7, 122.0, 121.0, 120.5, 117.9, 75.0, 70.7, 35.8, 28.2, 24.6, 22.5, 14.0.

HRMS (ESI+) m/z calculated for $C_{20}H_{25}N_2OS$ [M+H]$^+$: 341.1682, found [M+H]$^+$: 341.1688.

FTIR: 3064, 2954, 2924, 2853, 1597, 1500, 1457, 1379, 1287, 1090, 1073, 767, 747, 695.

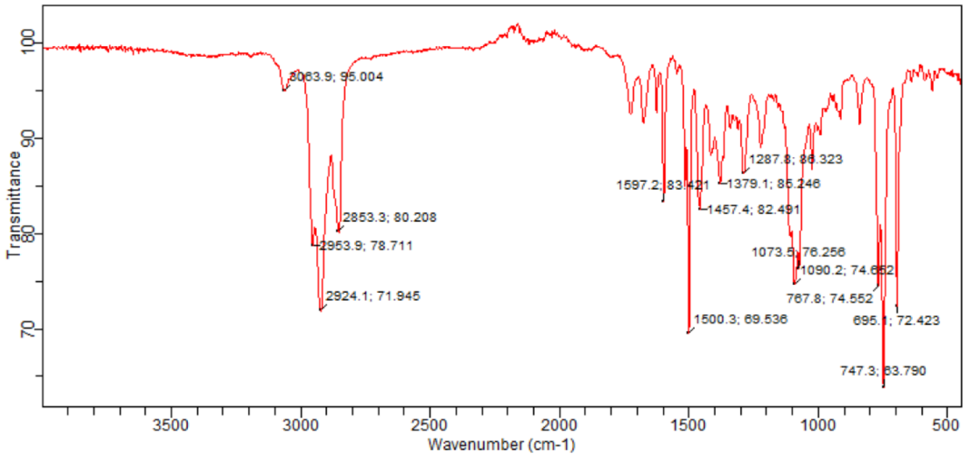

N-[1-(2-Phenyl-2*H*-indazol-3-yl)pentyl]acetamide (2ay)

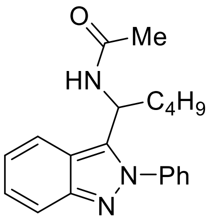

Eluent: hexane/EtOAc, 5:1. Colorless oil, 36% yield (15 mg).

^{1}H-NMR (400 MHz, $CDCl_3$) δ (ppm) 7.78–7.74 (m, 2H), 7.67–7.60 (m, 2H), 7.60–7.49 (m, 3H), 7.33 (ddd, *J* = 8.8, 6.6, 1.0 Hz, 1H), 7.16–7.07 (m, 1H), 5.94 (d, *J* = 7.6 Hz, 1H), 5.38 (q, *J* = 7.7 Hz, 1H), 2.06–1.87 (m, 5H), 1.19–1.03 (m, 4H), 0.75 (t, *J* = 7.1 Hz, 3H).

^{13}C-NMR (101 MHz, $CDCl_3$) δ (ppm) 169.2, 148.9, 139.8, 136.2, 129.5, 129.4, 126.8, 126.6, 122.0, 119.6, 119.6, 118.6, 47.4, 35.4, 28.3, 23.3, 22.3, 13.9.

HRMS (ESI+) m/z calculated for $C_{20}H_{24}N_3O$ $[M+H]^+$: 322.1914, found $[M+H]^+$: 322.1918.

3-[1-(1*H*-Indol-1-yl)pentyl]-2-phenyl-2*H*-indazole (2az)

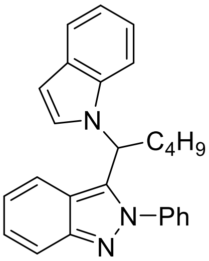

Eluent: hexane/EtOAc, 20:1. Colorless oil, 62% yield (27 mg).

^{1}H-NMR (400 MHz, CDCl$_3$) δ (ppm) 7.93 (dt, *J* = 8.6, 1.1 Hz, 1H), 7.79 (dt, *J* = 8.8, 1.0 Hz, 1H), 7.63–7.56 (m, 1H), 7.51–7.43 (m, 1H), 7.38 (ddd, *J* = 8.0, 6.6, 0.9 Hz, 3H), 7.25–7.16 (m, 4H), 7.10–7.03 (m, 2H), 6.88–6.81 (m, 1H), 6.48 (dd, *J* = 3.3, 0.8 Hz, 1H), 5.77 (t, *J* = 7.9 Hz, 1H), 2.58–2.46 (m, 2H), 1.32–1.21 (m, 4H), 0.81 (t, *J* = 7.0 Hz, 3H).

^{13}C-NMR (101 MHz, CDCl$_3$) δ (ppm) 148.9, 139.4, 135.6, 134.0, 129.7, 129.4, 128.6, 126.7, 126.6, 125.3, 123.1, 121.8, 121.1, 120.9, 120.2, 119.8, 118.7, 109.3, 102.7, 53.0, 34.6, 28.9, 22.3, 13.9.

HRMS (ESI+) m/z calculated for $C_{26}H_{26}N_3$ $[M+H]^+$: 380.2121, found $[M+H]^+$: 380.2129.

Synthetic applications

2-[(1,1'-Biphenyl)-4-yl]-3-(1-methoxypentyl)-2*H*-indazole (2ba)

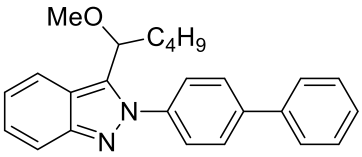

In a 5 mL Schlenk tube under argon flow, **2b** (0.061 mmol, 1 equiv.), phenylboronic acid (0.128 mmol, 2 equiv.), $Pd(PPh_3)_4$ (0.003 mmol, 5 mol %) and K_2CO_3 (0.128 mmol, 2 equiv.) were dissolved in a mixture of dioxane/H_2O (2:1, 0.1 M). The reaction was stirred in an oil bath at 110 ^oC for 18 hours. Afterwards the mixture was let cool down to room temperature, EtOAc was added (5 mL) and washed with H_2O (2 x 5 mL). The organic phase was dried over anhydrous Mg_2SO_4, filtered and concentrated under reduced pressure. The crude product was purified by column chromatography, using a mixture of Hex/EtOAc (20:1) to afford **2ba**.

White solid (20 mg, 89% yield), m.p. = 87-88 ^oC.

^{1}H-NMR (300 MHz, CDCl$_3$) δ (ppm) 7.91 (d, *J* = 8.4 Hz, 1H), 7.77 (d, *J* = 8.5 Hz, 3H), 7.72 – 7.63 (m, 2H), 7.64 – 7.56 (m, 2H), 7.50 (t, *J* = 7.4 Hz, 2H), 7.47 – 7.30 (m, 2H), 7.13 (dd, *J* = 8.5, 6.6 Hz, 1H), 4.67 (dd, *J* = 7.7, 6.1 Hz, 1H), 3.25 (s, 3H), 2.18 – 2.01 (m, 1H), 1.97 – 1.82 (m, 1H), 1.37 – 1.19 (m, 4H), 0.82 (t, *J* = 6.8 Hz, 3H).

^{13}C-NMR (75 MHz, CDCl$_3$) δ (ppm) 149.0, 142.3, 139.9, 139.2, 136.6, 129.1, 128.1, 128.0, 127.4, 127.0, 121.9, 121.1, 120.7, 117.8, 76.4, 56.9, 35.6, 28.1, 22.5, 14.0.

HRMS (ESI+) m/z calculated for $C_{25}H_{27}N_2O$ $[M+H]^+$: 371.2118, found $[M+H]^+$: 371.2129.

3-[3-(1-Methoxypentyl)-2*H*-indazol-2-yl]aniline (2bb)

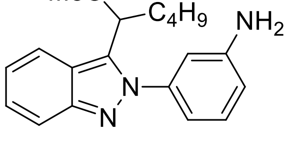

In a 5 mL Schlenk tube under argon flow, **2g** (0.059 mmol, 1.0 equiv.), NH_4Cl (0.029 mmol, 0.5 equiv.) and Fe powder (0.59 mmol, 10 equiv.) were suspended in EtOH/H_2O (4:1, 0.1 M). The reaction was stirred in an oil bath at 80 ^{o}C for 3 hours. The mixture was cooled down to room temperature, EtOAc was added (5 mL) and washed with H_2O (2 x 5 mL). The organic phase was dried over anhydrous Mg_2SO_4, filtered and concentrated *in vacuo* to afford **2bb**.

Yellow oil (17 mg, 93% yield)

^{1}H-NMR (500 MHz, CDCl$_3$) δ (ppm) 7.89 (dt, *J* = 8.5, 1.1 Hz, 1H), 7.73 (dt, *J* = 8.7, 1.0 Hz, 1H), 7.32 (ddd, *J* = 8.7, 6.6, 1.1 Hz, 1H), 7.30 – 7.26 (m, 1H), 7.09 (ddd, *J* = 8.5, 6.6, 1.0 Hz, 1H), 6.88 – 6.74 (m, 3H), 4.64 (dd, *J* = 7.8, 6.0 Hz, 1H), 3.89 (s, 2H), 3.20 (s, 3H), 2.10 – 2.02 (m, 1H), 1.90-1.83 (m, 1H), 1.36 – 1.21 (m, 4H), 0.82 (t, *J* = 7.2 Hz, 3H).

^{13}C-NMR (126 MHz, CDCl$_3$) δ (ppm) 149.0, 147.6, 141.0, 136.5, 129.9, 126.8, 121.7, 121.2, 120.4, 117.8, 116.4, 115.8, 113.1, 76.4, 56.9, 35.7, 28.1, 22.5, 14.0.

HRMS (ESI+) m/z calculated for $C_{18}H_{24}N_3O$ $[M+H]^+$: 310.1914, found $[M+H]^+$: 310.1918.

5-(3-(1-Methoxypentyl)-2H-indazol-2-yl)isophthalic acid (2bc)

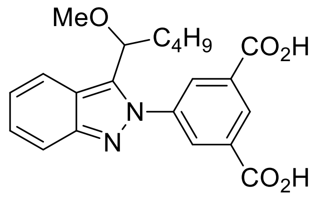

In a 5 mL Schlenk tube under argon flow, **2k** (0.075 mmol, 1.0 equiv.) was dissolved in THF/MeOH (3:1, 0.25 M). An aqueous solution of KOH (30% w/v, 30 µL) was added dropwise at room temperature. The reaction was stirred in an oil bath at 50 ^{o}C for 18 hours. The mixture was cooled down to room temperature, H_2O was added (5 mL) and washed with Et_2O (2 x 5 mL). The aqueous layer was acidified with aq. HCl (1 M) and extracted with DCM. The organic phase was dried over anhydrous Mg_2SO_4, filtered and concentrated under pressure to afford **2bc**.

White solid (28 mg, 99% yield) m.p. = 230 ^{o}C (decomposition).

^{1}H-NMR (500 MHz, CD$_3$COCD$_3$) δ (ppm) 8.82 (s, 1H), 8.51 (d, *J* = 1.6 Hz, 2H), 7.93 (d, *J* = 8.6 Hz, 1H), 7.70 (d, *J* = 8.8 Hz, 1H), 7.36 (dd, *J* = 8.8, 6.6 Hz, 1H), 7.13 (dd, *J* = 8.6, 6.6 Hz, 1H), 4.78 (dd, *J* = 7.9, 6.0 Hz, 1H), 3.25 (s, 3H), 2.12 – 2.06 (m, 1H), 1.95 – 1.88 (m, 1H), 1.39 – 1.33 (m, 1H), 1.26 – 1.19 (m, 3H), 0.79 (t, *J* = 7.1 Hz, 3H).

^{13}C-NMR (126 MHz, CD$_3$COCD$_3$) δ (ppm) 166.0, 149.9, 141.8, 137.4, 133.3, 132.1, 131.5, 127.7, 122.8, 122.0, 121.9, 121.9, 118.6, 76.8, 57.0, 35.9, 28.8, 22.9, 14.1.

HRMS (ESI+) m/z calculated for $C_{21}H_{23}N_2O_5$ $[M+H]^+$: 383.1601, found $[M+H]^+$: 383.1608.

3-[1-(Ethylsulfonyl)pentyl]-2-phenyl-2*H*-indazole (2bd)

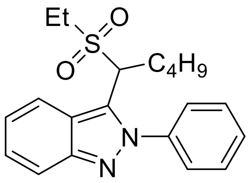

In a 5 mL Schlenk tube under argon flow, **2av** (0.092 mmol, 1.0 equiv.) was dissolved in DCM (0.15 M). The reaction was stirred at room temperature for 16 hours after addition of *m*CPBA at 0 ^{o}C. The mixture was diluted with DCM (5 mL) and washed with a saturated aqueous $NaHCO_3$ solution (2 × 5 mL). The organic phase was dried over anhydrous Mg_2SO_4, filtered and concentrated under pressure. The crude product was purified by column chromatography with a mixture of Hex/EtOAc (5:1).

Yellow oil (16 mg, 49% yield).

^{1}H-NMR (300 MHz, $CDCl_3$) δ (ppm) 7.98 (d, *J* = 8.6 Hz, 1H), 7.78 (d, *J* = 8.8 Hz, 1H), 7.71 – 7.44 (m, 5H), 7.43 – 7.32 (m, 1H), 7.25 – 7.14 (m, 1H), 4.56 (dd, *J* = 11.5, 4.0 Hz, 1H), 2.70 (q, *J* = 7.4 Hz, 2H), 2.64 – 2.52 (m, 1H), 2.43 (d, *J* = 13.0 Hz, 1H), 1.31 – 1.21 (m, 6H), 1.13 (t, *J* = 7.5 Hz, 3H), 0.84 (t, *J* = 6.8 Hz, 3H).

^{13}C-NMR (101 MHz, $CDCl_3$) δ (ppm) 149.3, 139.2, 130.2, 129.8, 127.3, 127.2, 123.4, 121.4, 121.4, 121.0, 118.4, 61.4, 45.8, 29.9, 29.3, 22.5, 13.8, 5.9.

HRMS (ESI+) m/z calculated for $C_{26}H_{26}N_3$ $[M+H]^+$: 357.1631, found $[M+H]^+$: 357.1621.

X-Ray crystallographic data

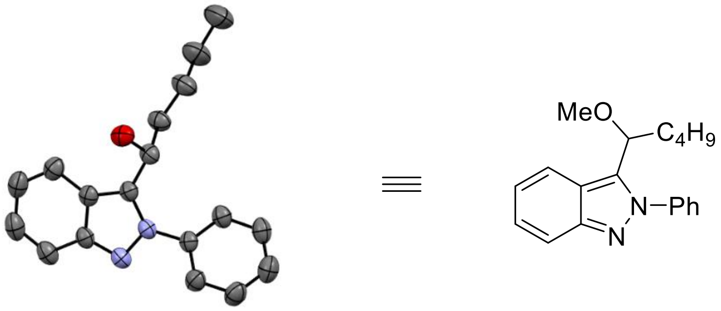

X-Ray Crystallographic Data for **2a** (CCDC number: 2255195). Thermal ellipsoids are shown at the 50% probability level.

Crystallised from	Hexane/decane
Empirical formula	$C_{19}H_{22}N_2O$
Formula weight	294.38
Temperature/K	230.0
Crystal system	triclinic
Space group	P-1
a/Å	8.9835(5)
b/Å	14.0643(7)
c/Å	14.4375(8)
α/°	77.169(3)
β/°	81.257(3)
γ/°	71.901(3)
Volume/Å^3	1683.78(16)
Z	4
ρ_{calc}g/cm^3	1.161
μ/mm^{-1}	0.564
F(000)	632.0
Crystal size/mm^3	0.2 × 0.15 × 0.03
Radiation	CuKα (λ = 1.54178)
2Θ range for data collection/°	6.304 to 145.23
Index ranges	$-11 \leq h \leq 11$, $-17 \leq k \leq 17$, $-17 \leq l \leq 17$
Reflections collected	37875
Independent reflections	6548 [R_{int} = 0.1124, R_{sigma} = 0.0685]
Data/restraints/parameters	6548/0/396
Goodness-of-fit on F^2	1.025
Final R indexes [I>=2σ (I)]	R_1 = 0.0733, wR_2 = 0.1709
Final R indexes [all data]	R_1 = 0.1202, wR_2 = 0.2047
Largest diff. peak/hole / e Å^{-3}	0.59/-0.49

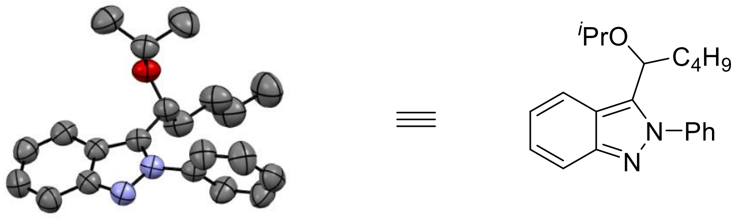

X-Ray Crystallographic Data for **2ag** (CCDC 2255196). Thermal ellipsoids are shown at the 50% probability level.

Crystallised from	Hexane/Ethyl acetate
Empirical formula	$C_{21}H_{26}N_2O$
Formula weight	322.44
Temperature/K	230.0
Crystal system	triclinic
Space group	P-1
a/Å	9.5743(7)
b/Å	10.7541(7)
c/Å	11.0541(7)
α/°	66.627(4)
β/°	81.478(4)
γ/°	66.312(3)
Volume/Å^3	956.66(12)
Z	2
ρ_{calc}g/cm^3	1.119
μ/mm^{-1}	0.534
F(000)	348.0
Crystal size/mm^3	0.3 × 0.15 × 0.04
Radiation	CuKα (λ = 1.54178)
2Θ range for data collection/°	9.676 to 133.14
Index ranges	$-11 \leq h \leq 11$, $-12 \leq k \leq 12$, $-13 \leq l \leq 13$
Reflections collected	15568
Independent reflections	3307 [R_{int} = 0.0774, R_{sigma} = 0.0546]
Data/restraints/parameters	3307/0/220
Goodness-of-fit on F^2	1.040
Final R indexes [I>=2σ (I)]	R_1 = 0.0816, wR_2 = 0.2283
Final R indexes [all data]	R_1 = 0.1129, wR_2 = 0.2731
Largest diff. peak/hole / e Å^{-3}	0.55/-0.26

DFT calculations

All reported structures were optimized at Density Functional Theory level as implemented in Gaussian 16.[5] The geometry optimizations were performed using B3LYP functional[6] with 6-31G(d,p) basis set. Solvent effects were considered in all the calculations applying the continuum polarizable continuum model (CPCM) using acetonitrile as solvent at 298.15 K. The structures were optimized without geometrical constraint. Stationary points were characterized by frequency calculations (no negative frequency for minima and one negative frequency for transition states).

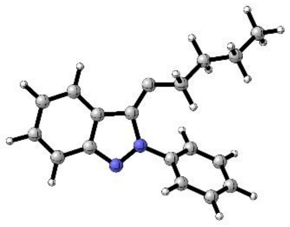

Carbene

N	-0.76297600	0.78223400	-0.08274200
N	-1.93532800	1.39759700	-0.11825700
C	-2.87770700	0.41715900	-0.06474200
C	-4.28214900	0.57101900	-0.11311300
C	-2.26925600	-0.85475500	0.10261500
C	-5.04335900	-0.57258200	0.01414400
C	-3.06564600	-2.00782100	0.22365200
C	-4.43930600	-1.85001500	0.18799500
H	-6.12621600	-0.50469700	-0.01713700
H	-2.60158000	-2.98020800	0.34776500
H	-4.72970800	1.54961500	-0.25023500
H	-5.08123100	-2.71905900	0.29607300
C	0.40336800	1.60563900	0.03635700
C	0.60753200	2.62717500	-0.89443500
C	1.28883900	1.41009500	1.09983600
C	1.72695200	3.44832200	-0.76762300
H	-0.09996800	2.76319200	-1.70449900
C	2.40840800	2.23406900	1.21139400
H	1.09474000	0.63194500	1.82969300
C	2.63048500	3.25125300	0.27991500
H	1.89448000	4.23924300	-1.49161900
H	3.09970600	2.08714000	2.03494800
H	3.50173000	3.89174800	0.37297600
C	-0.85325800	-0.63719400	0.06122600
C	0.10215200	-1.65413000	0.02898200
C	1.44125400	-1.44760000	-0.58849800
H	1.74819900	-0.41767400	-0.82464700
H	1.30198500	-1.94209600	-1.57001000
C	2.58347300	-2.18687900	0.13514100
H	2.27406100	-3.22407800	0.31388700
H	2.72968200	-1.73469800	1.12568000
C	3.90874300	-2.15371500	-0.63432800

H	3.76339600	-2.61199700	-1.62227500
H	4.19409500	-1.10989900	-0.82249500
C	5.04673800	-2.86891900	0.10013200
H	4.80509400	-3.92395400	0.27250500
H	5.97914300	-2.83007800	-0.47265400
H	5.23694900	-2.40999100	1.07714800ç

E(RB3LYP) = -806.172820859
Zero-point correction= 0.314385 (Hartree/Particle)
Thermal correction to Energy= 0.331970
Thermal correction to Enthalpy= 0.332914
Thermal correction to Gibbs Free Energy= 0.266724
Sum of electronic and zero-point Energies= -805.858436
Sum of electronic and thermal Energies= -805.840851
Sum of electronic and thermal Enthalpies= -805.839907
Sum of electronic and thermal Free Energies= -805.906097

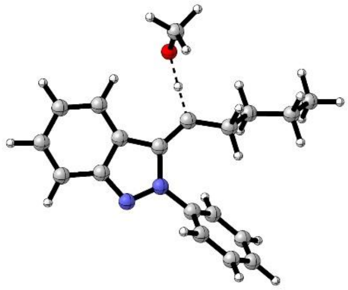

$TS_{Carbene-I}$

N	-0.46283500	-1.36040500	0.02423100
N	-1.50723200	-2.14309400	-0.09445300
C	-2.60059000	-1.33096200	-0.20217400
C	-3.94421700	-1.72980200	-0.35436100
C	-2.21614200	0.03117900	-0.16523300
C	-4.88064500	-0.72035200	-0.47182300
C	-3.17704000	1.04551400	-0.28497400
C	-4.49834800	0.64672600	-0.43922000
H	-5.92948500	-0.97057400	-0.59310300
H	-2.85973000	2.08753600	-0.26359100
H	-4.21587100	-2.77921700	-0.37848500
H	-5.27160700	1.40237300	-0.54066600
C	0.83244300	-1.98237100	0.07450600
C	1.11693900	-2.86564500	1.11667500
C	1.75157700	-1.73512800	-0.94745800
C	2.35977100	-3.49775700	1.14371100
H	0.37865200	-3.04251500	1.89077400
C	2.99078400	-2.37328400	-0.90688700
H	1.49399500	-1.06658700	-1.76151500
C	3.29623800	-3.25049200	0.13704200
H	2.59508400	-4.18076500	1.95322900
H	3.71208100	-2.19101000	-1.69657100

H	4.26219700	-3.74442100	0.16334600
C	-0.78537800	0.03003700	0.02413500
C	-0.01077200	1.13400900	0.30218900
C	1.42899900	1.18545000	0.66218300
H	1.92358200	0.22137800	0.80094300
H	1.47815000	1.72384800	1.62107400
C	2.20642300	2.04507600	-0.37167400
H	1.67409400	2.99405800	-0.50330000
H	2.19090700	1.53840500	-1.34512800
C	3.65809100	2.29552700	0.05170400
H	3.66692600	2.80232300	1.02569200
H	4.16377400	1.33258300	0.20121000
C	4.43527000	3.13031600	-0.97046400
H	3.96887200	4.11096700	-1.11698000
H	5.46687600	3.29668200	-0.64383500
H	4.47059200	2.63086300	-1.94521300
H	-0.58976700	2.28218200	0.15369700
O	-1.14464600	3.47156600	0.00975700
C	-1.25648600	3.94672200	1.30549200
H	-0.68958400	4.88501900	1.48970100
H	-2.30172700	4.16624600	1.61236700
H	-0.88187500	3.22400700	2.06962600

E(RB3LYP) = -921.915434543
Zero-point correction= 0.363433 (Hartree/Particle)
Thermal correction to Energy= 0.384872
Thermal correction to Enthalpy= 0.385816
Thermal correction to Gibbs Free Energy= 0.310157
Sum of electronic and zero-point Energies= -921.552001
Sum of electronic and thermal Energies= -921.530563
Sum of electronic and thermal Enthalpies= -921.529619
Sum of electronic and thermal Free Energies= -921.605278
Frequency = -909.8277

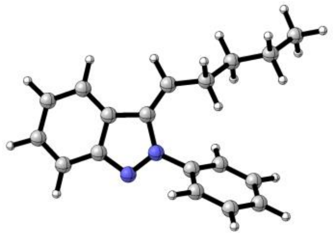

Intermediate I

N	-0.72774600	0.80437800	-0.01022900
N	-1.85968100	1.41750200	0.18114100
C	-2.84988000	0.47427400	0.15456400
C	-4.22745600	0.70001200	0.31932900
C	-2.30463400	-0.81708000	-0.04394700
C	-5.04709300	-0.41567200	0.28042800
C	-3.14284700	-1.92994300	-0.08378100
C	-4.50981900	-1.70795200	0.08308700

H	-6.11846900	-0.30281600	0.40281300
H	-2.76039900	-2.93349700	-0.23261100
H	-4.61419800	1.70143800	0.46741200
H	-5.18536400	-2.55664200	0.06213600
C	0.48423200	1.58158900	0.03320800
C	0.65700800	2.60089000	-0.90397200
C	1.42002000	1.33418200	1.03990700
C	1.81275600	3.37803900	-0.84006100
H	-0.09359100	2.76827000	-1.66810100
C	2.56992900	2.12107600	1.08932000
H	1.24270500	0.55828300	1.77633400
C	2.76773200	3.13737500	0.15074700
H	1.96578800	4.16866200	-1.56675000
H	3.30423800	1.94500300	1.86795200
H	3.66536400	3.74534500	0.19469800
C	-0.87905700	-0.60213400	-0.19412500
C	0.06087300	-1.51374300	-0.57175700
C	1.49503300	-1.38390400	-0.91965900
H	1.82478000	-0.34829100	-0.98243600
H	1.60596900	-1.83448200	-1.91664900
C	2.39694300	-2.18511700	0.05923400
H	2.04620700	-3.22288300	0.11028200
H	2.29427900	-1.76666500	1.06706000
C	3.86835000	-2.15156800	-0.37134200
H	3.95672700	-2.56255900	-1.38511900
H	4.20373500	-1.10830500	-0.42879700
C	4.77458400	-2.93282000	0.58435900
H	4.48099100	-3.98702600	0.63612000
H	5.81791600	-2.89228800	0.25617900
H	4.72759600	-2.52328400	1.59940400
H	-0.33232700	-2.52643900	-0.64953500

E(RB3LYP) = -806.694545634
Zero-point correction= 0.329434 (Hartree/Particle)
Thermal correction to Energy= 0.346983
Thermal correction to Enthalpy= 0.347927
Thermal correction to Gibbs Free Energy= 0.281952
Sum of electronic and zero-point Energies= -806.365112
Sum of electronic and thermal Energies= -806.347563
Sum of electronic and thermal Enthalpies= -806.346619
Sum of electronic and thermal Free Energies= -806.412593

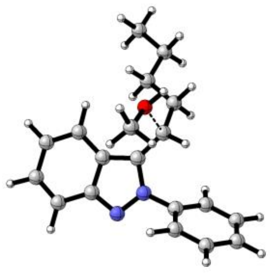

TSI-2a

N	-1.25299900	0.65853800	-0.29490400
N	-1.31819100	1.95390300	-0.54243100
C	-0.04439200	2.41158900	-0.43205900
C	0.37464800	3.75265500	-0.57339800

C	0.86746100	1.35269400	-0.13633800
C	1.71323600	4.02021900	-0.38852900
C	2.22820300	1.66587300	0.07809000
C	2.62570200	2.98319600	-0.05741700
H	2.08103800	5.03652900	-0.48334400
H	2.95028700	0.90929300	0.34956000
H	-0.34550000	4.52896600	-0.80664700
H	3.66958100	3.23591900	0.09887900
C	1.32416500	-2.10578500	-0.14534700
H	1.25475800	-3.01848600	0.45822200
H	0.97256200	-2.41144600	-1.14449600
C	2.78015700	-1.64614800	-0.24122000
H	3.11113600	-1.27286400	0.73488500
H	2.86418400	-0.82218700	-0.95699600
C	3.70157200	-2.79116600	-0.68517800
H	3.35535100	-3.17920000	-1.65205800
H	3.61591000	-3.62147300	0.02784500
C	5.16634500	-2.36058100	-0.79883800
H	5.54759500	-1.99630000	0.16162000
H	5.28691200	-1.55511900	-1.53169400
C	-2.46908300	-0.10560700	-0.36361600
C	-3.53863800	0.27564500	0.45037400
C	-2.57838300	-1.17499300	-1.25624100
C	-4.73230700	-0.43940700	0.37563700
H	-3.42698700	1.11474500	1.12744700
C	-3.77765100	-1.88605400	-1.31495500
H	-1.75291000	-1.42747800	-1.91268100
C	-4.85175300	-1.52108600	-0.50144400
H	-5.56723300	-0.15351900	1.00672500
H	-3.87217700	-2.71586600	-2.00730900
H	-5.78319300	-2.07538500	-0.55371200
H	5.79947800	-3.19575600	-1.11390600
C	0.24139000	-1.17130700	0.31608300
C	0.04502300	0.18845700	-0.06069200
H	-0.69774700	-1.69958900	0.45943100
O	0.70091200	-1.09147400	2.18403400
H	0.70456100	-2.00655100	2.51196200
C	-0.14100000	-0.28217300	3.04509300
H	-0.11077000	0.73061200	2.64775400
H	-1.16535300	-0.65981500	3.06083200
H	0.29137900	-0.29645800	4.04672600

E(RB3LYP) = -922.417955099
Zero-point correction= 0.384045 (Hartree/Particle)
Thermal correction to Energy= 0.404773
Thermal correction to Enthalpy= 0.405718
Thermal correction to Gibbs Free Energy= 0.333386
Sum of electronic and zero-point Energies= -922.033911
Sum of electronic and thermal Energies= -922.013182
Sum of electronic and thermal Enthalpies= -922.012238
Sum of electronic and thermal Free Energies= -922.084569
Frequency: -197.1003

Copies of NMR Spectra

Methyl 4-aminobenzoate

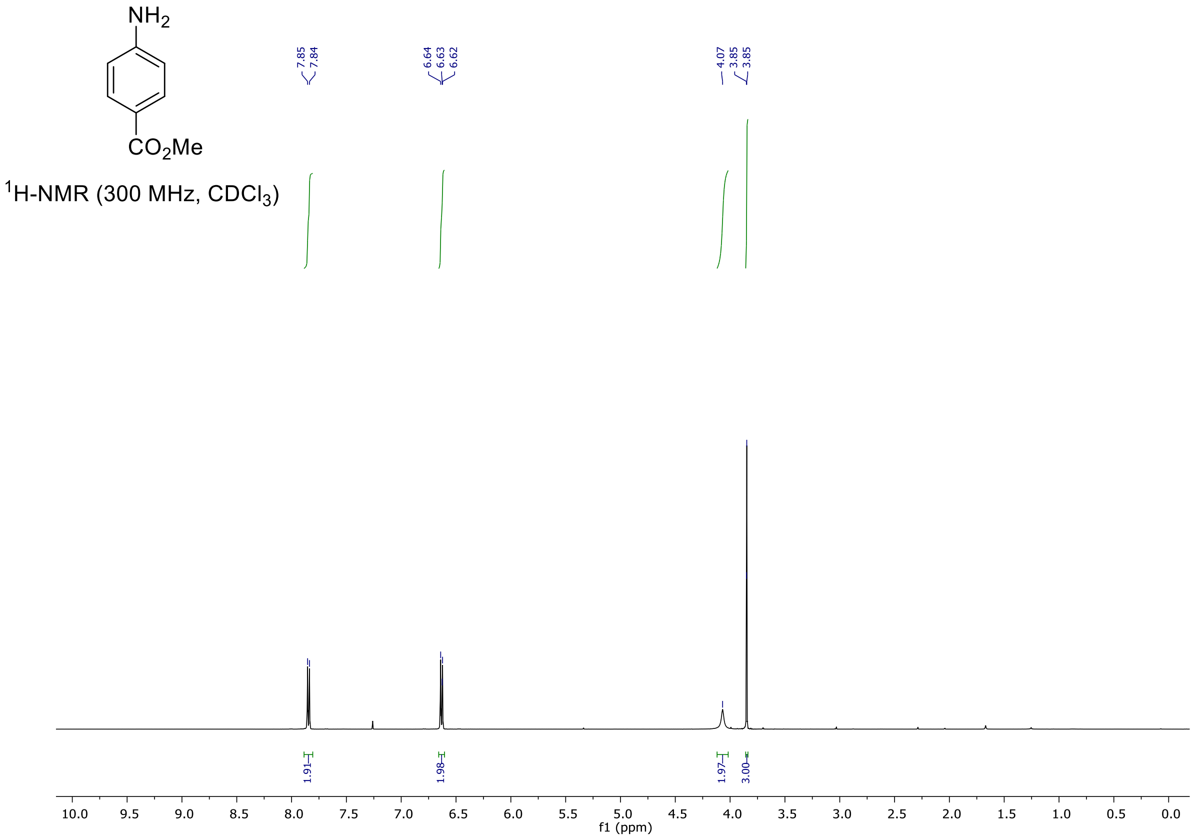

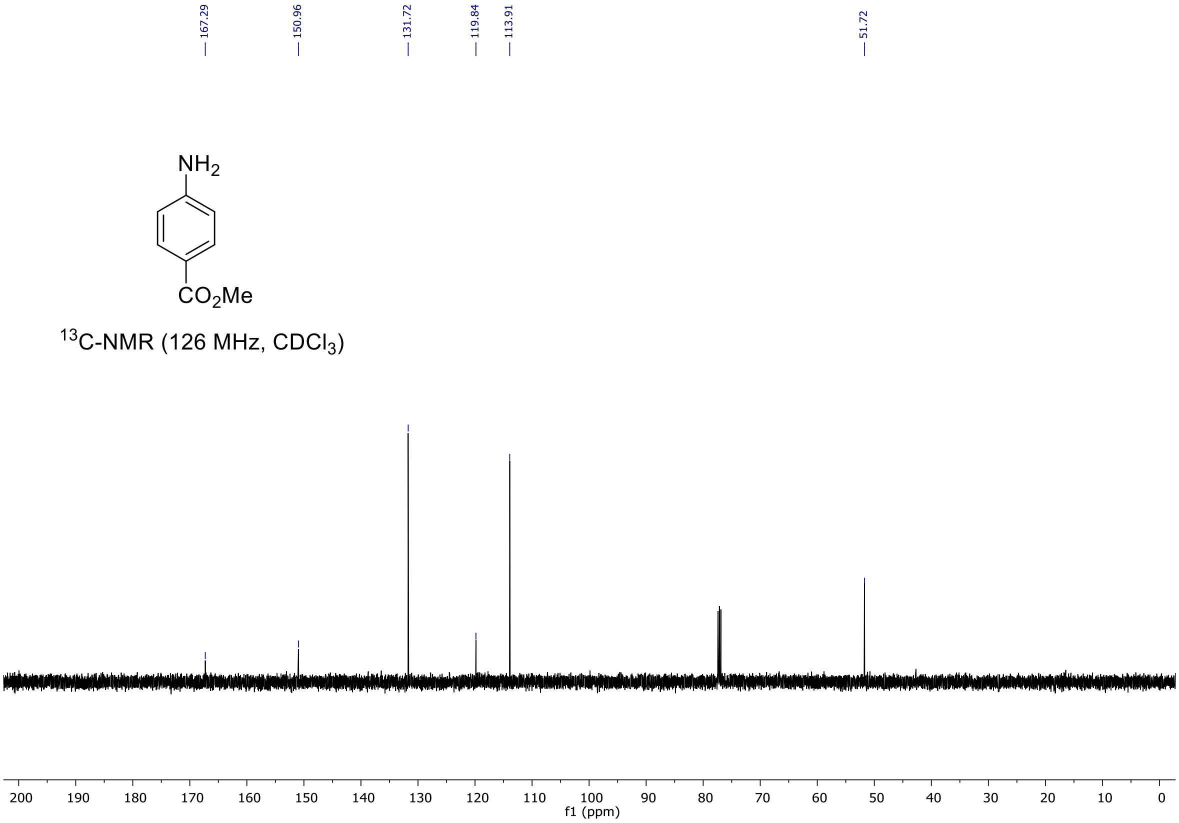

2-(Hex-1-yn-1yl)aniline

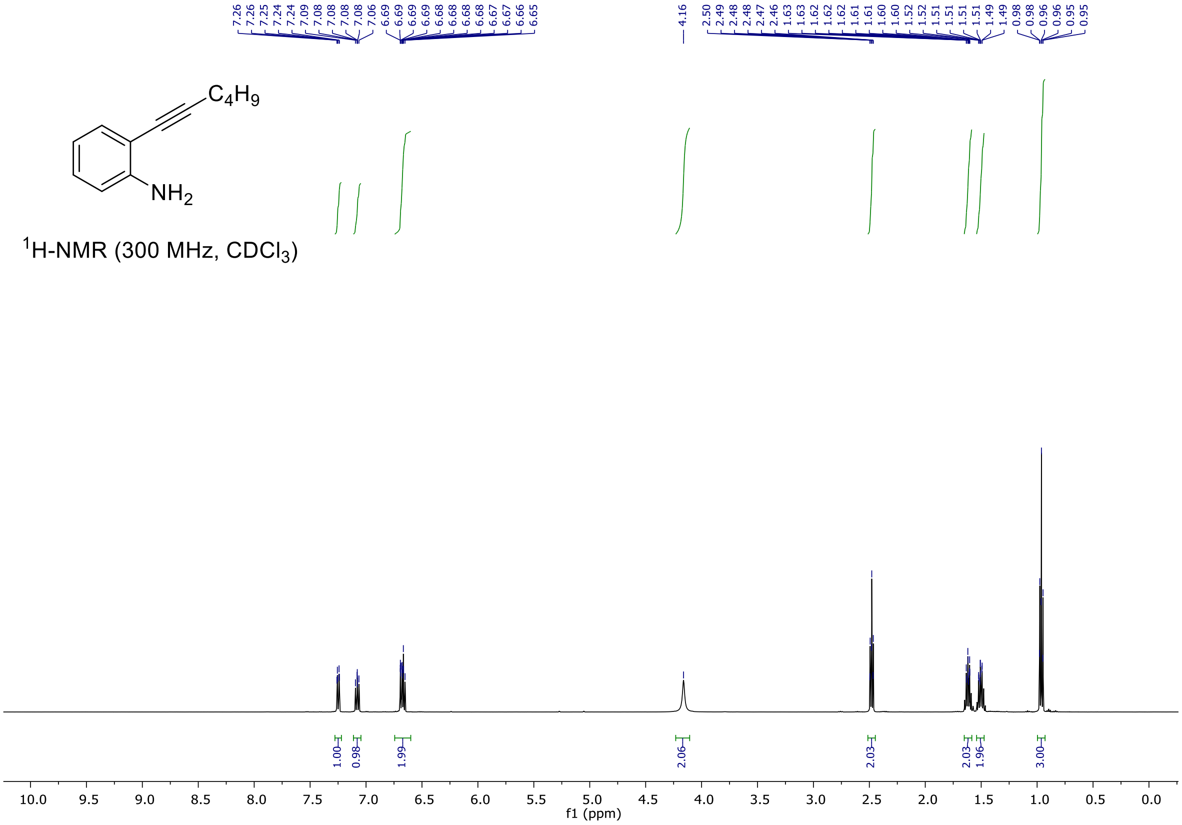

^{1}H-NMR (300 MHz, $CDCl_3$)

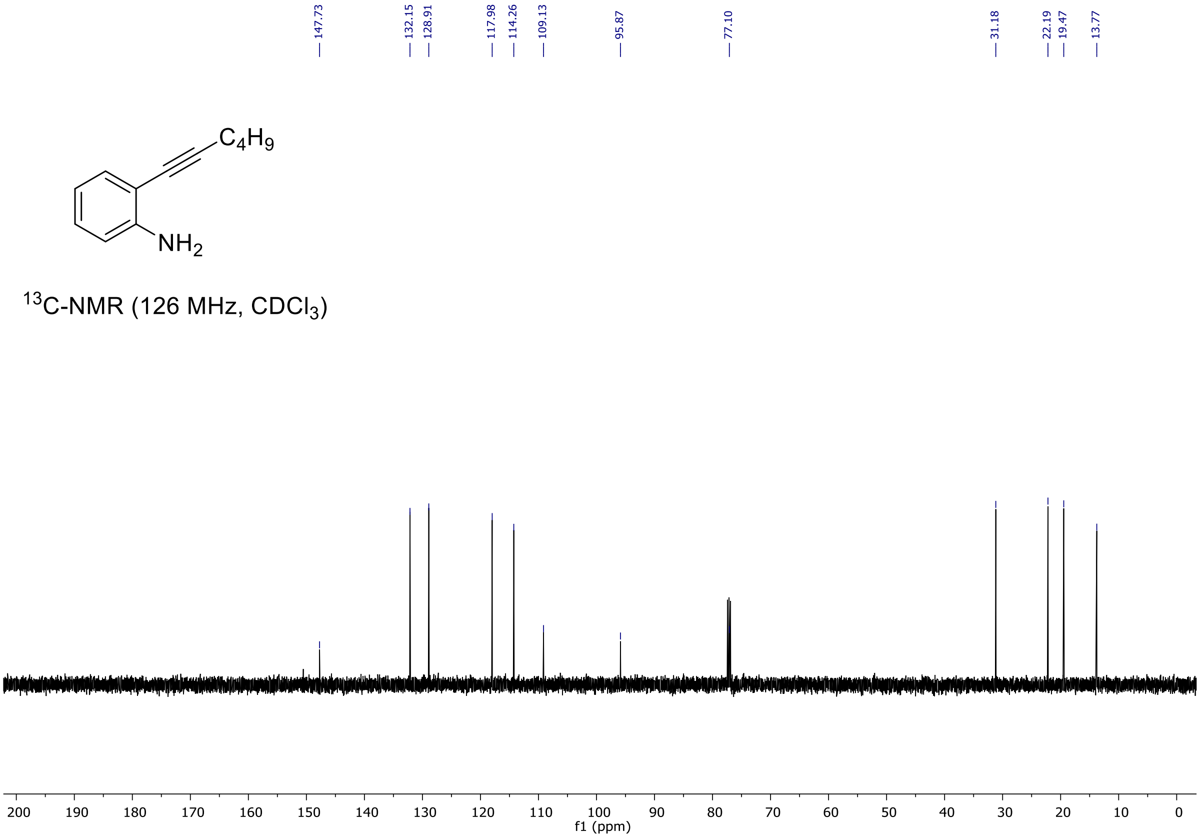

^{13}C-NMR (126 MHz, $CDCl_3$)

3-[(2,5,7,8-Tetramethyl-2-(4,8,12-trimethyltridecyl)chroman-6-yl)oxy]propa- n-1-ol

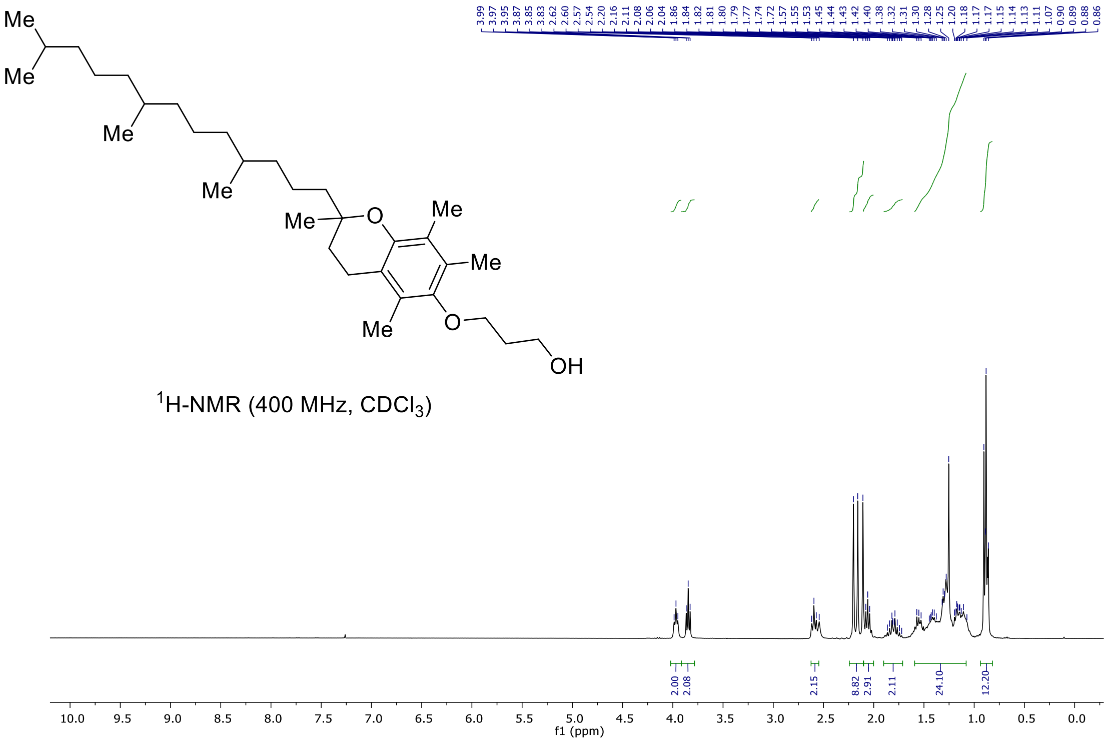

^{1}H-NMR (400 MHz, $CDCl_3$)

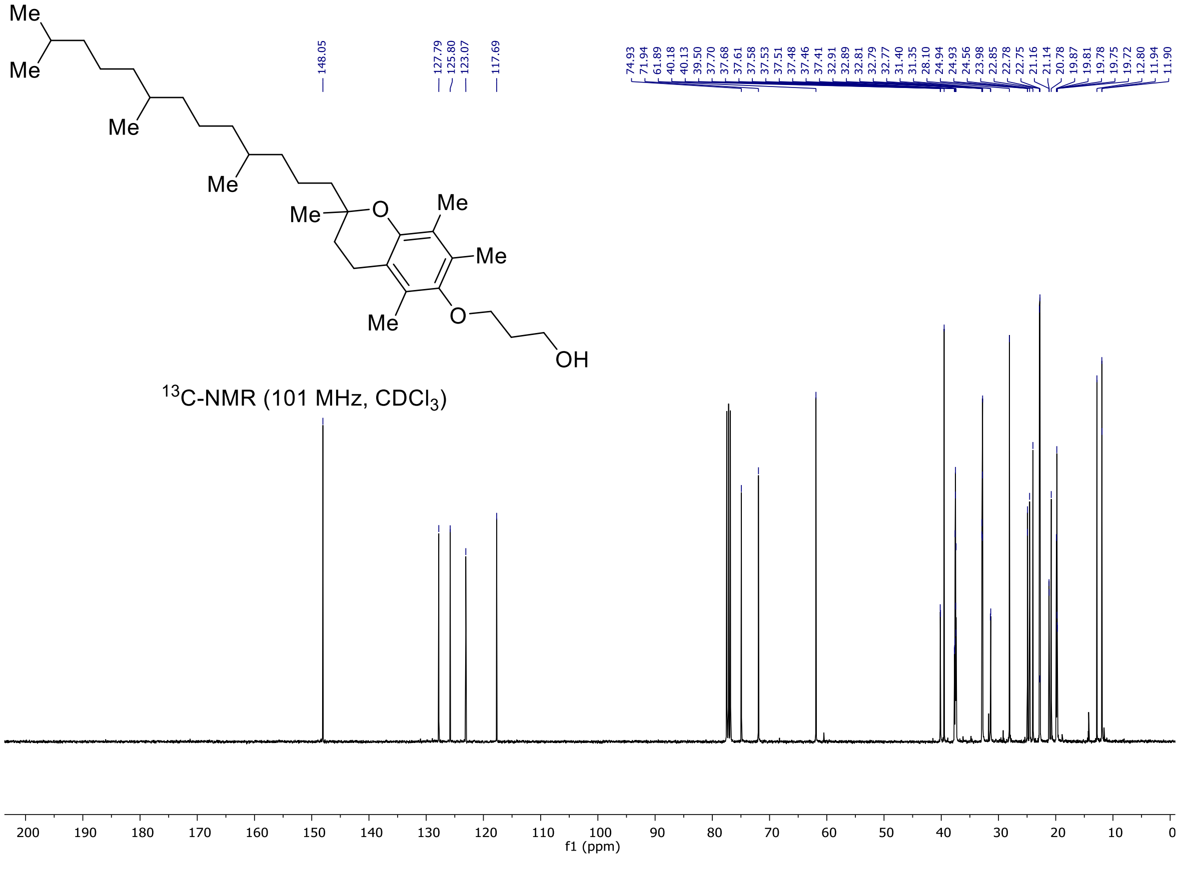

^{13}C-NMR (101 MHz, $CDCl_3$)

(8*R*,9*S*,13*S*,14*S*)-3-(3-Hydroxypropoxy)-13-methyl-7,8,9,11,12,13,15,16-octa-hydro-6H-cyclopenta[a]phenanthren-17(14H)-one

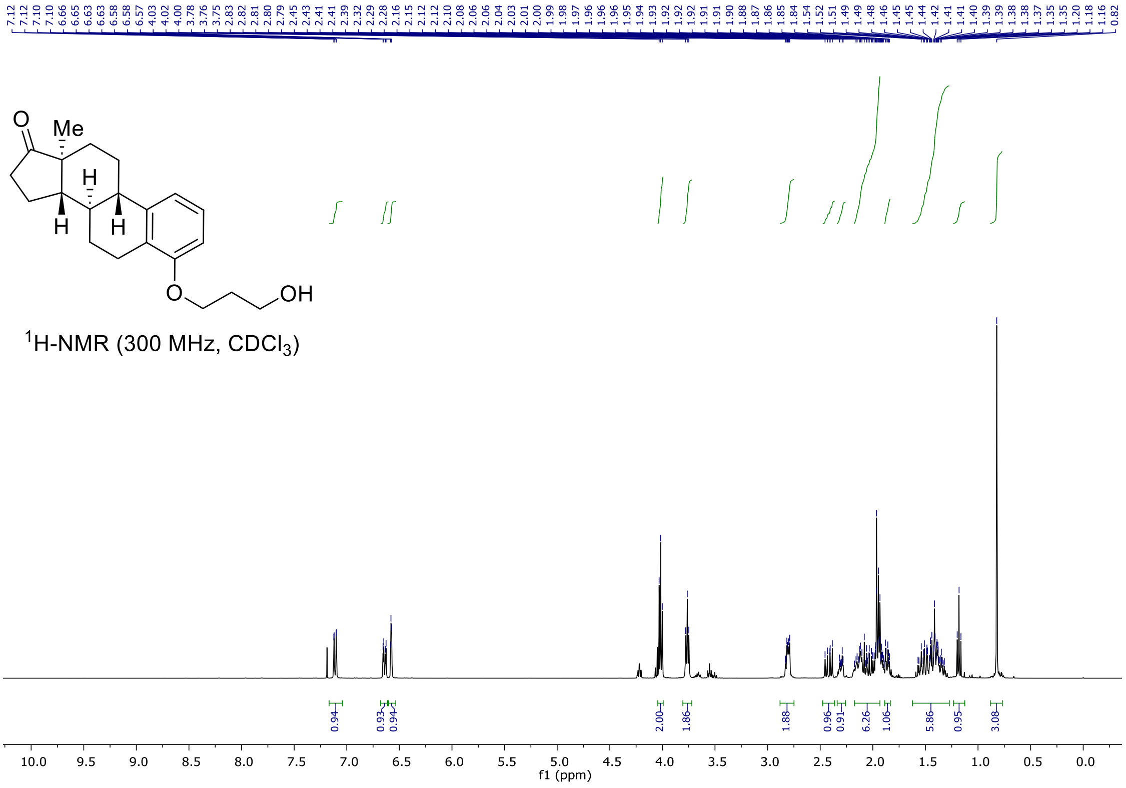

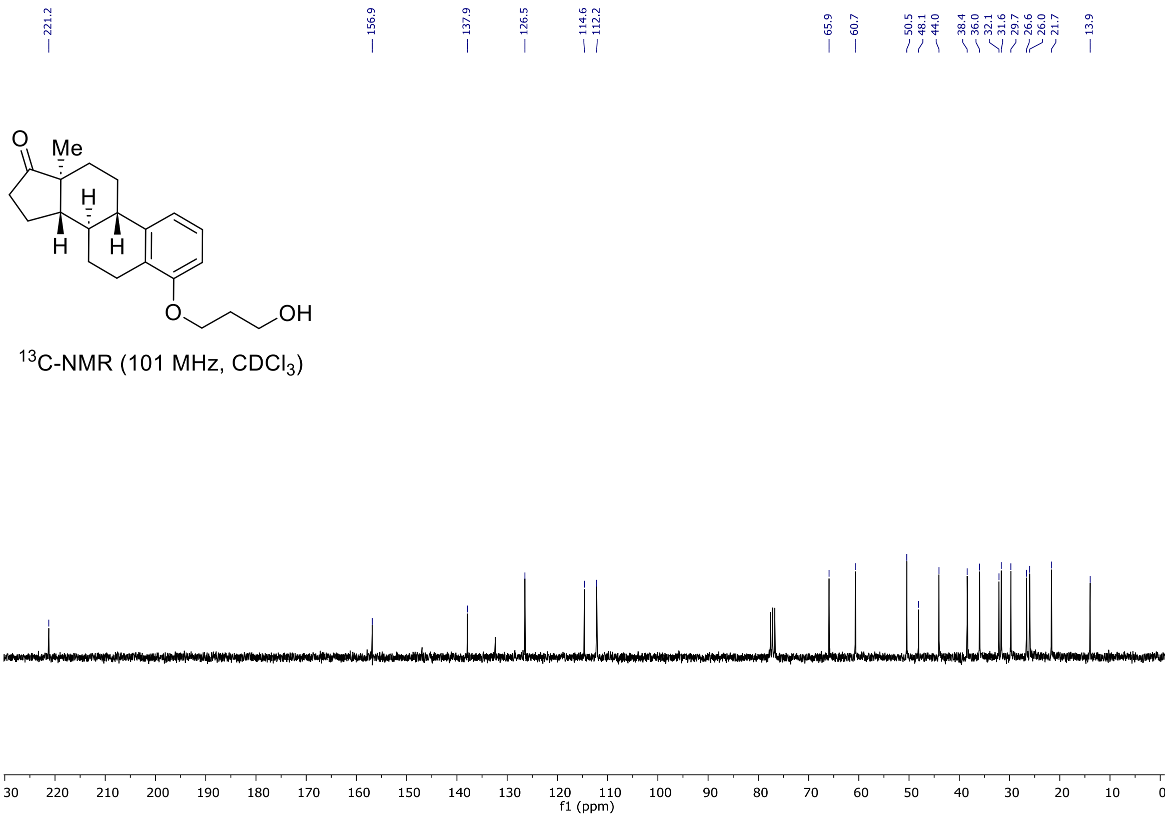

(*E*)-1-(2-Iodophenyl)-2-phenyldiazene

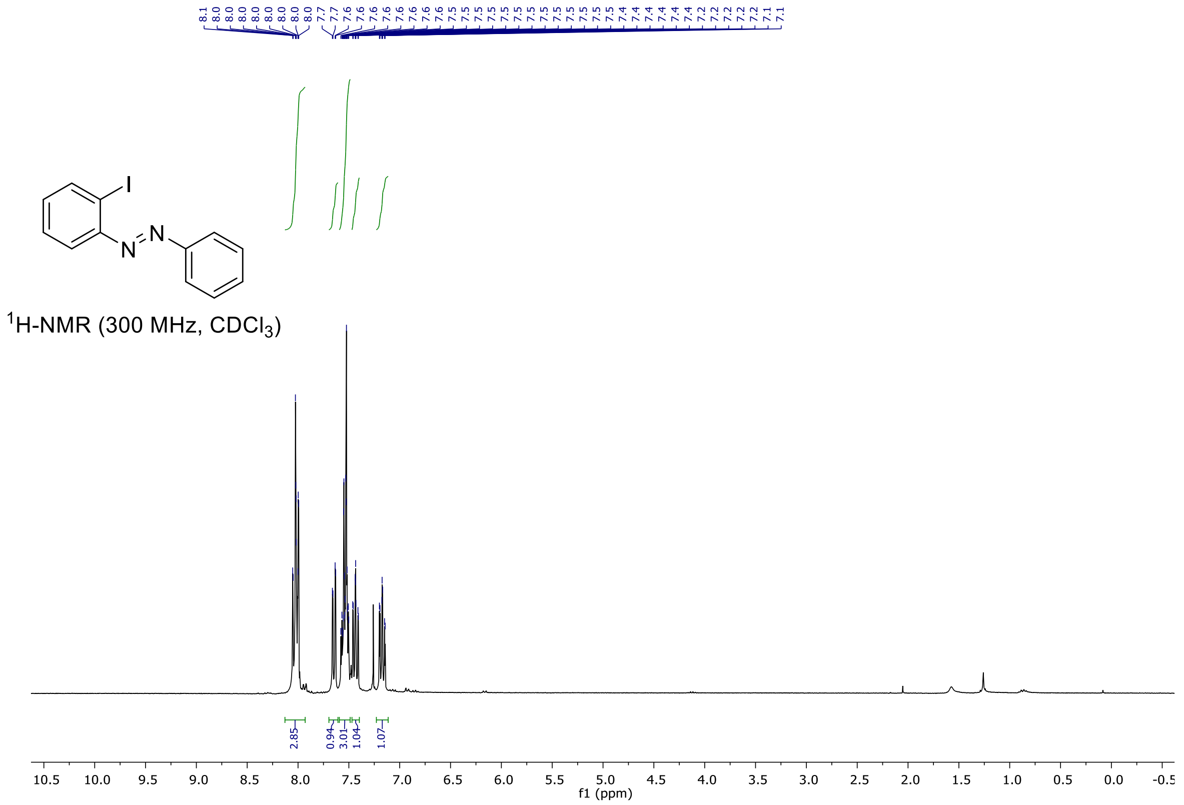

^{1}H-NMR (300 MHz, $CDCl_3$)

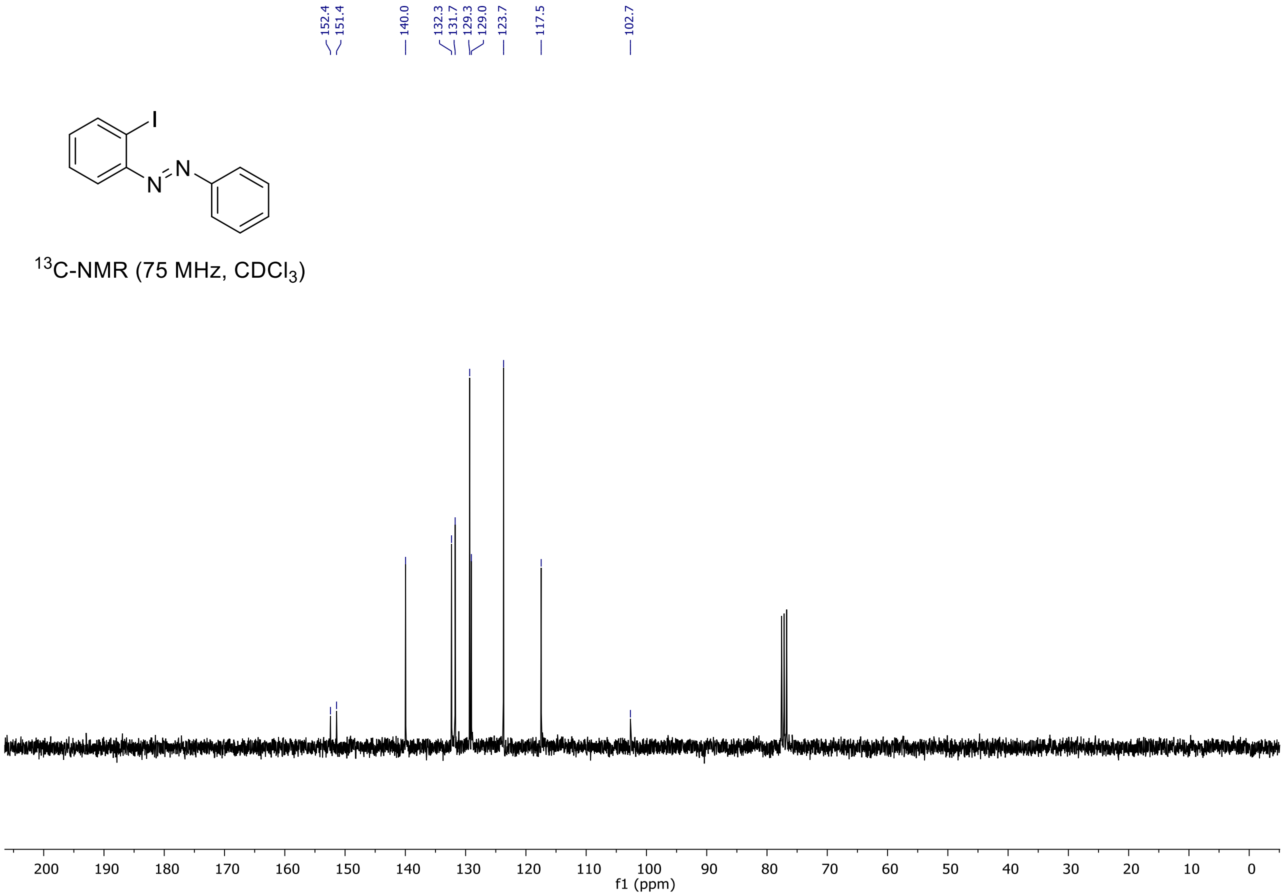

^{13}C-NMR (75 MHz, $CDCl_3$)

(*E*)-1-(4-Bromophenyl)-2-(2-iodophenyl)diazene

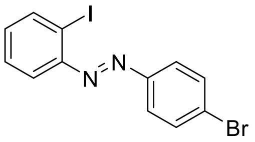

^{1}H-NMR (300 MHz, $CDCl_3$)

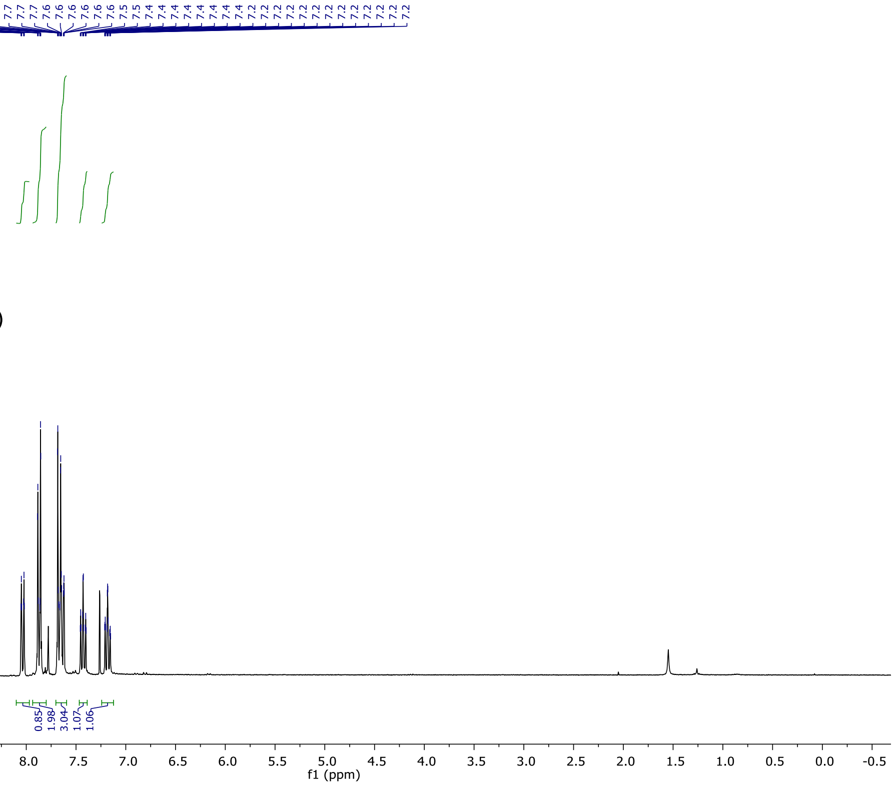

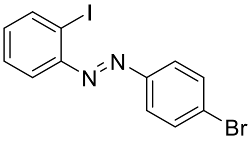

^{13}C-NMR (75 MHz, $CDCl_3$)

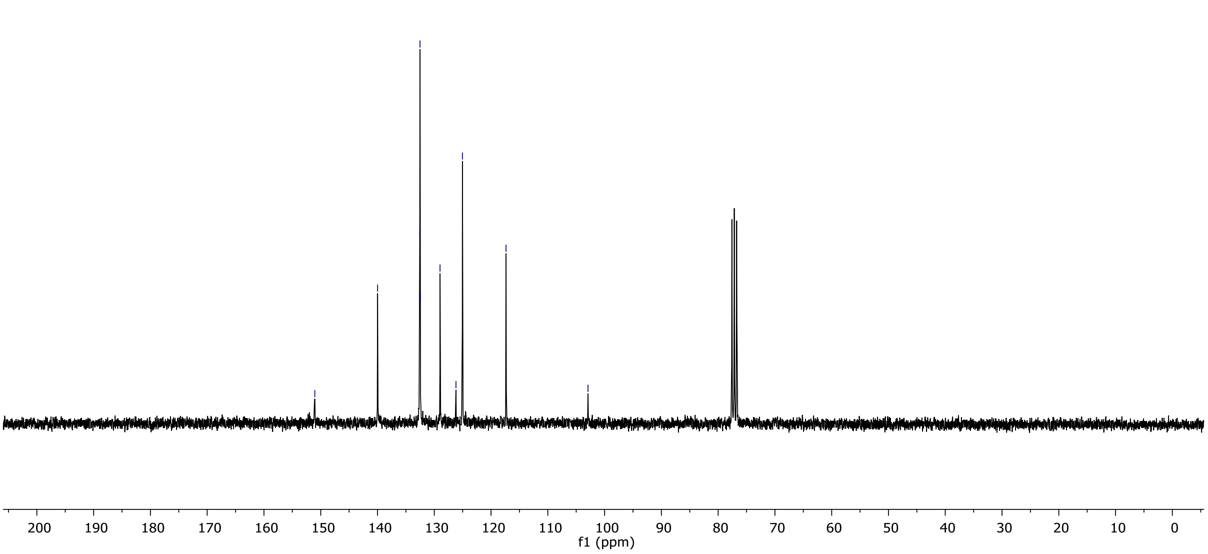

(*E*)-1-(3-Bromophenyl)-2-(2-iodophenyl)diazene

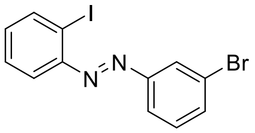

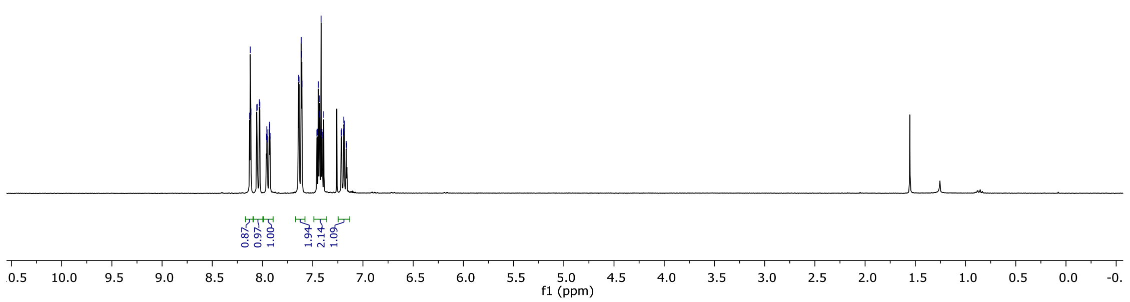

^{1}H-NMR (300 MHz, $CDCl_3$)

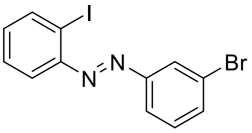

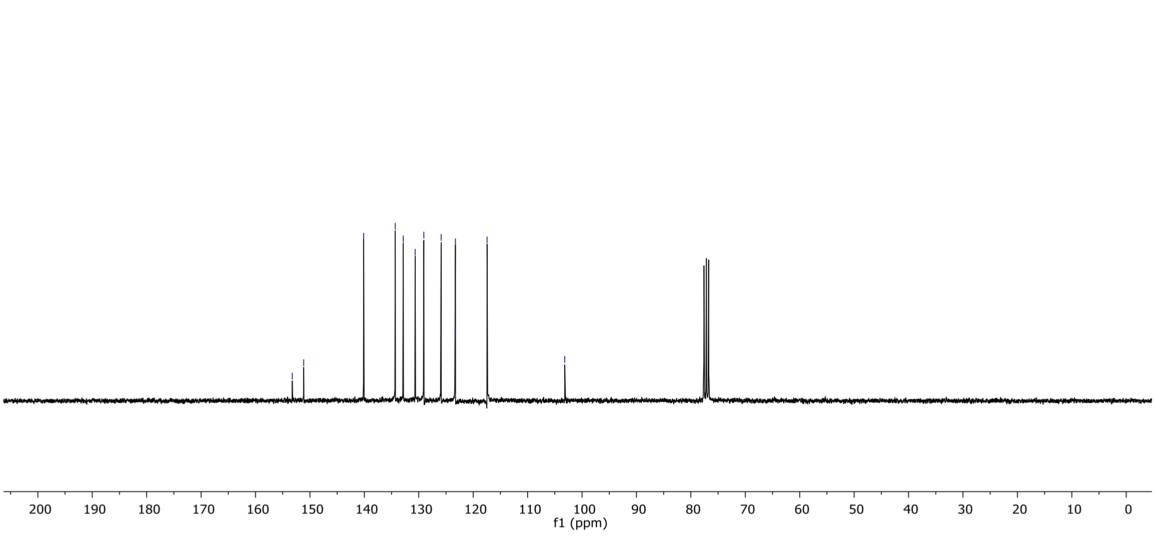

^{13}C-NMR (75 MHz, $CDCl_3$)

(*E*)-1-(3-Fluorophenyl)-2-(2-iodophenyl)diazene

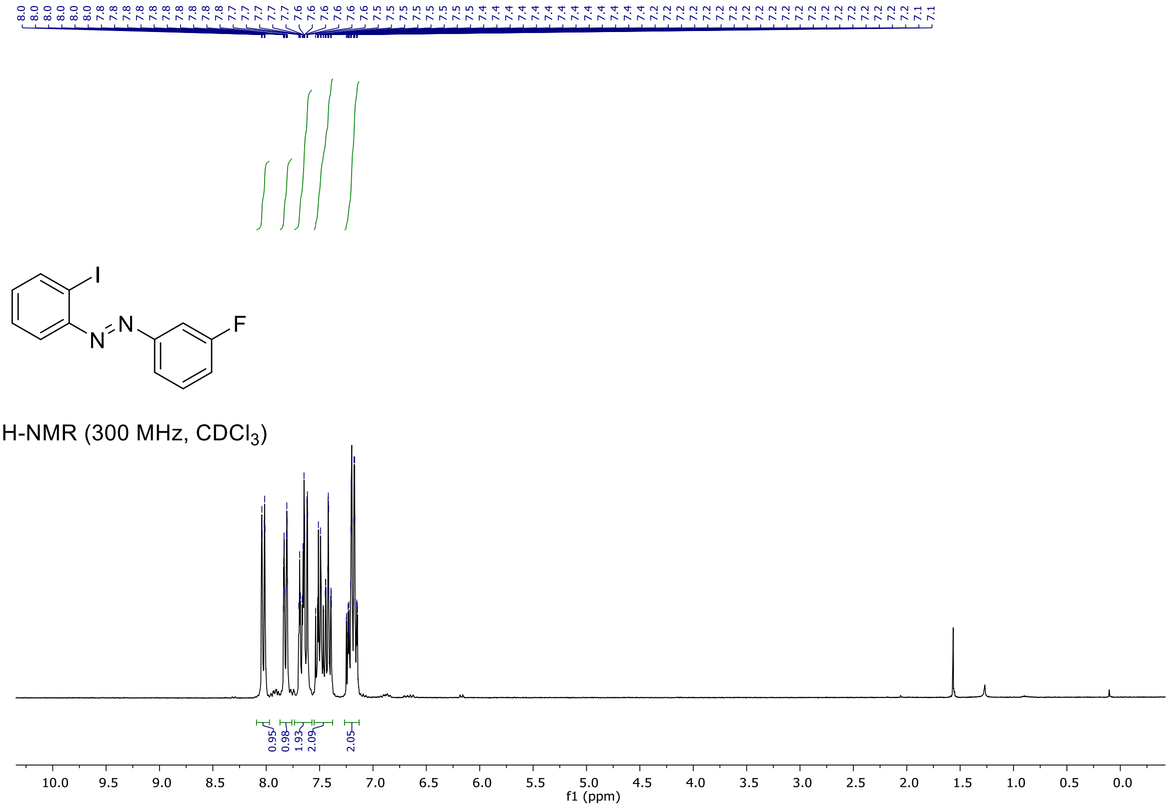

^{1}H-NMR (300 MHz, $CDCl_3$)

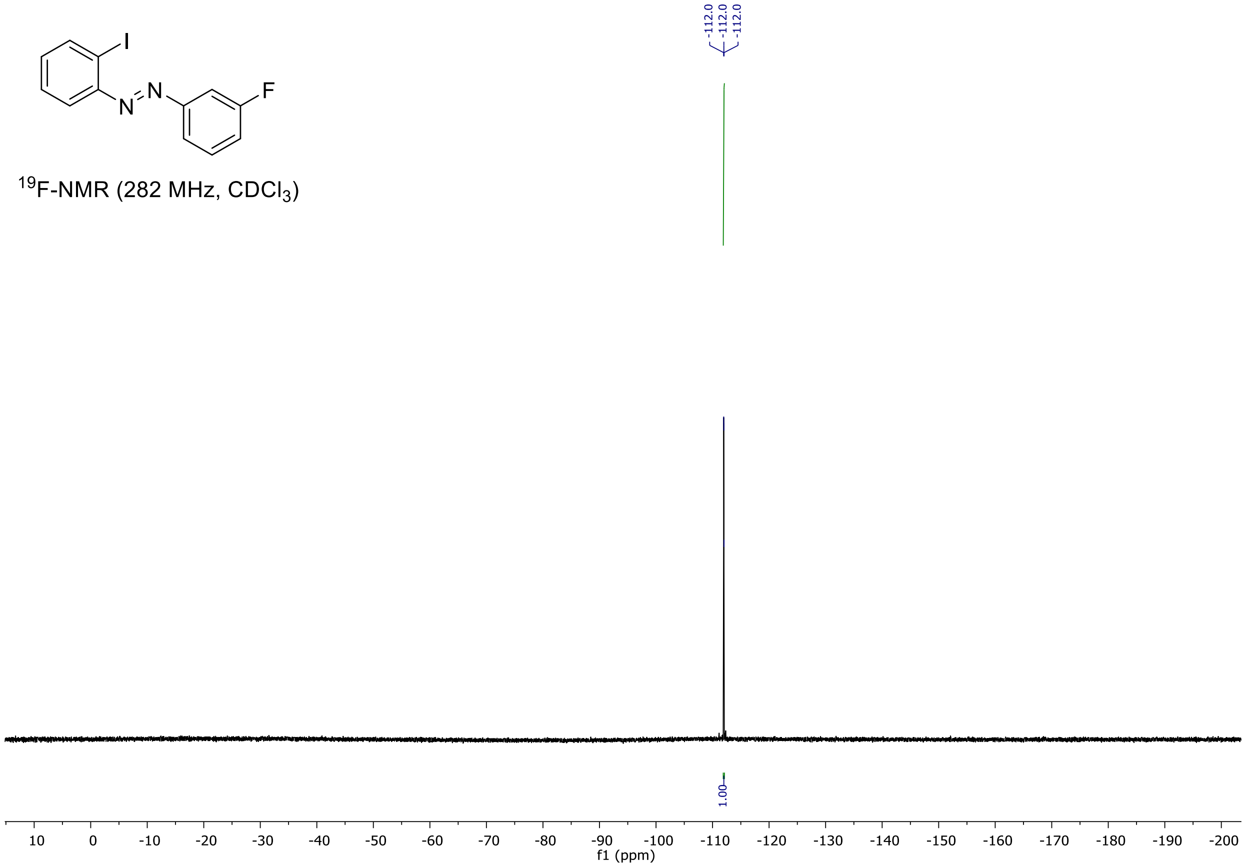

^{19}F-NMR (282 MHz, $CDCl_3$)

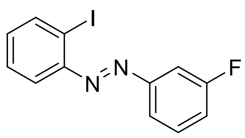

^{13}C-NMR (75 MHz, $CDCl_3$)

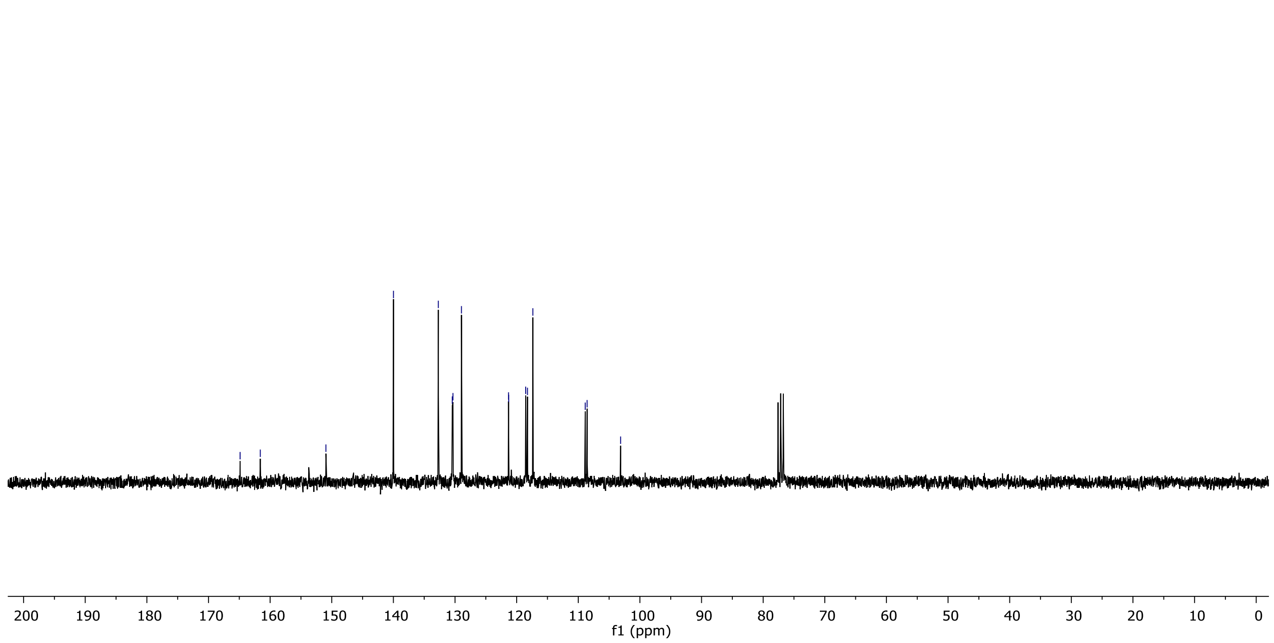

(*E*)-1-(2-Fluorophenyl)-2-(2-iodophenyl)diazene

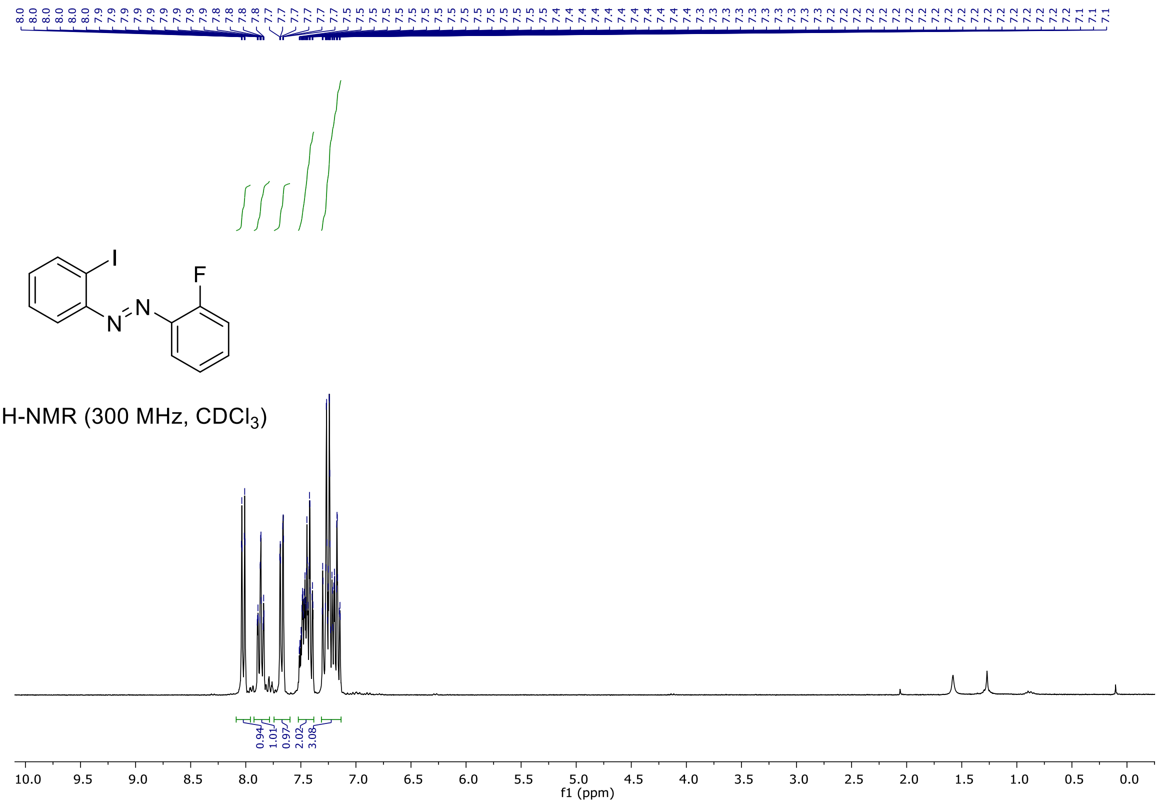

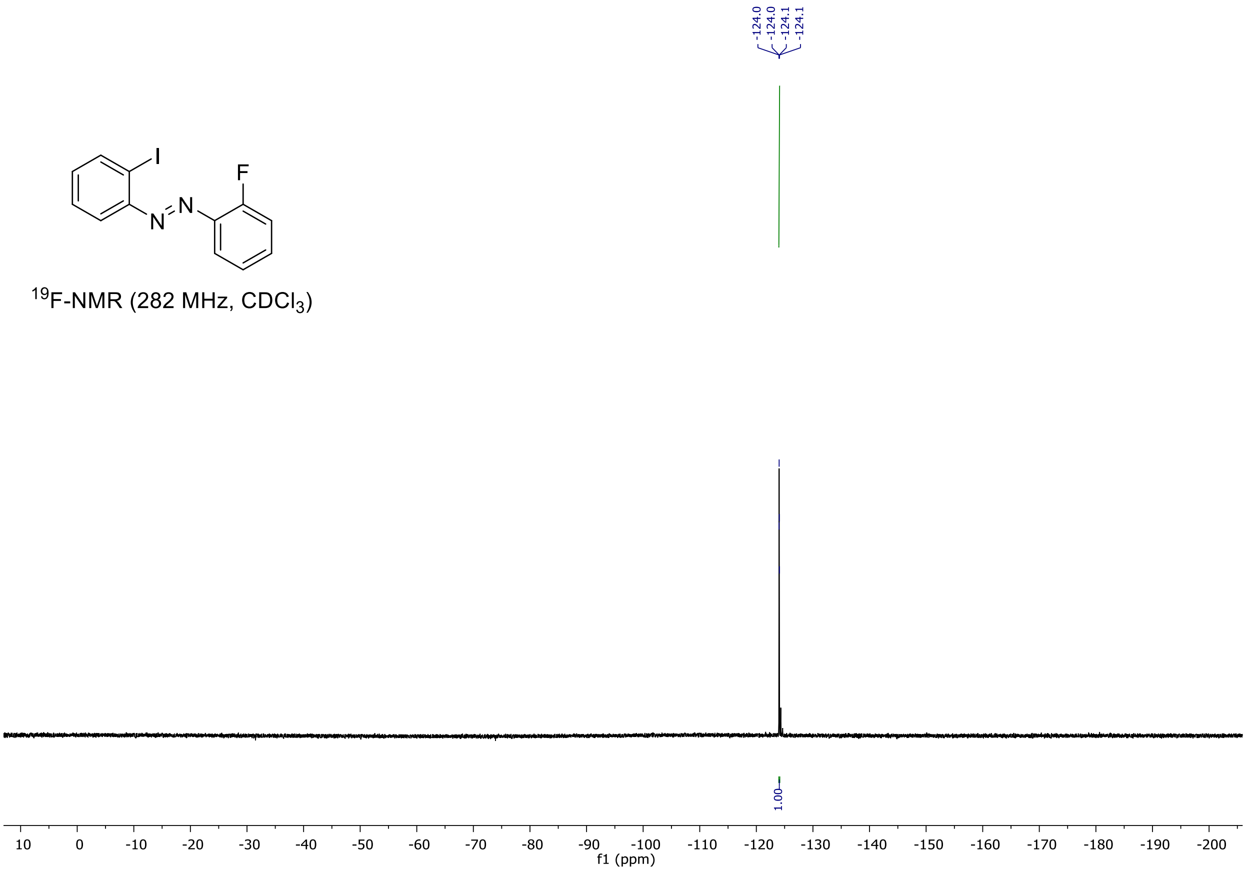

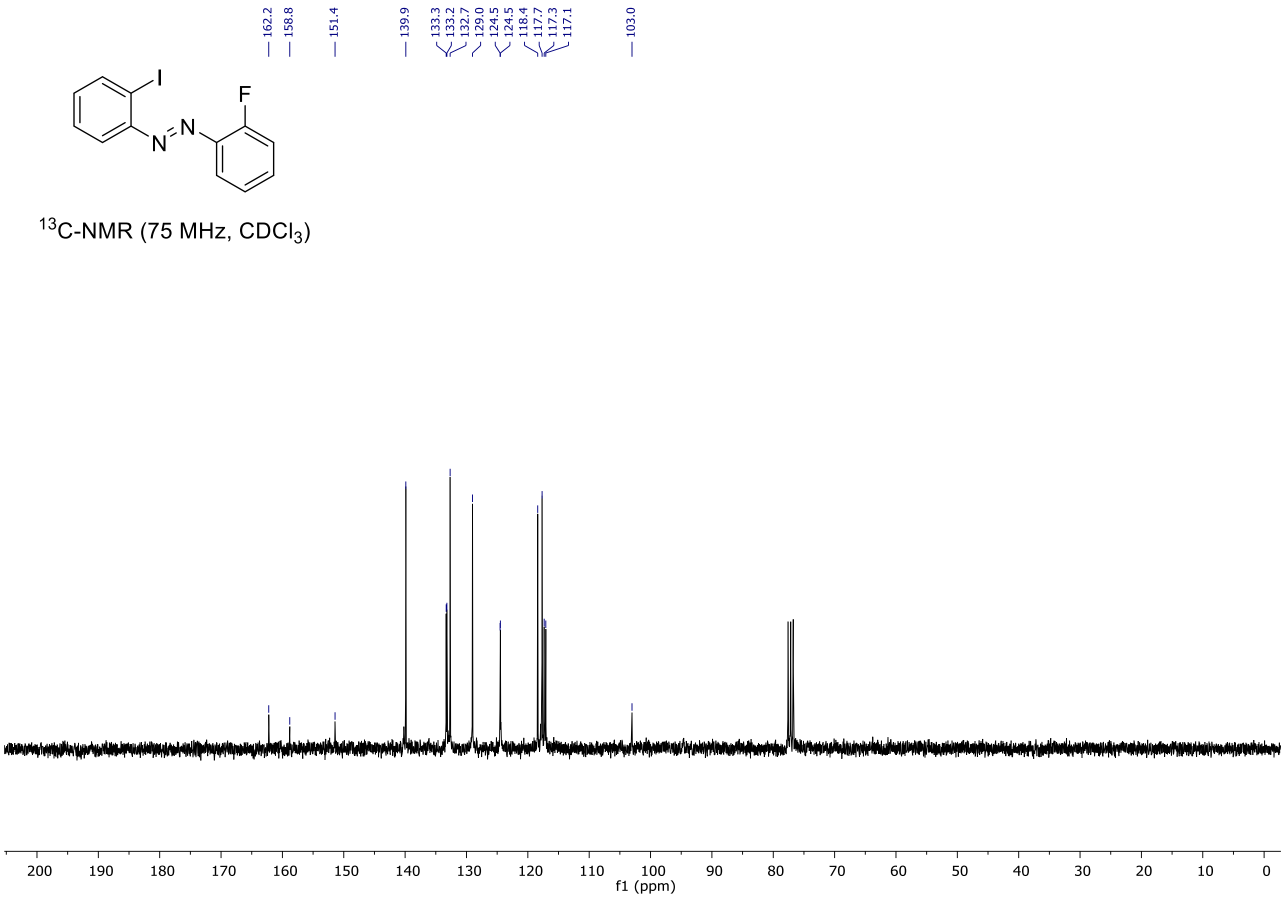
I
F
N
N
13C-NMR (75 MHz, CDCl3)
162.2
158.8
151.4
139.9
133.3
133.2
132.7
129.0
124.5
124.5
118.4
117.7
117.3
117.1
103.0
200 190 180 170 160 150 140 130 120 110 100 90 80 70 60 50 40 30 20 10 0
f1 (ppm)

(E)-4-[(2-iodophenyl)diazinyl]benzonitrile

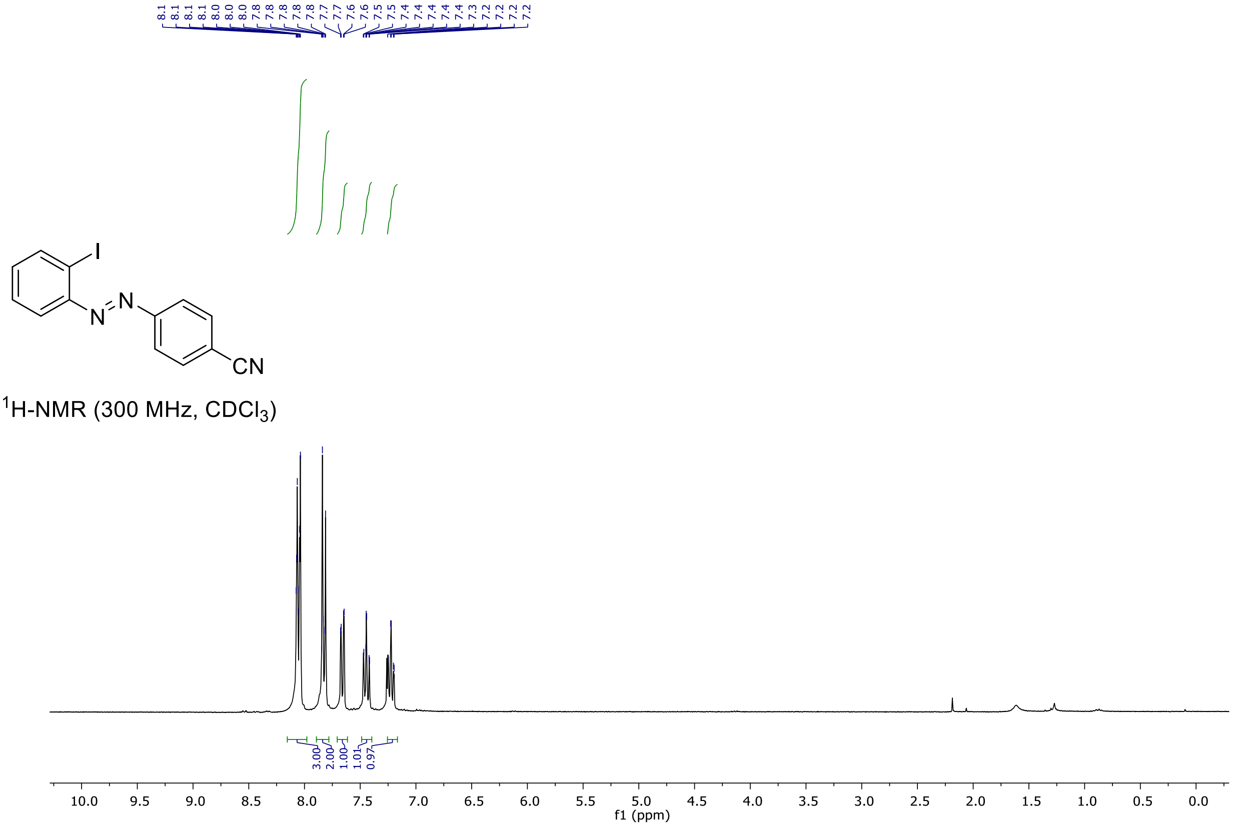

^{1}H-NMR (300 MHz, $CDCl_3$)

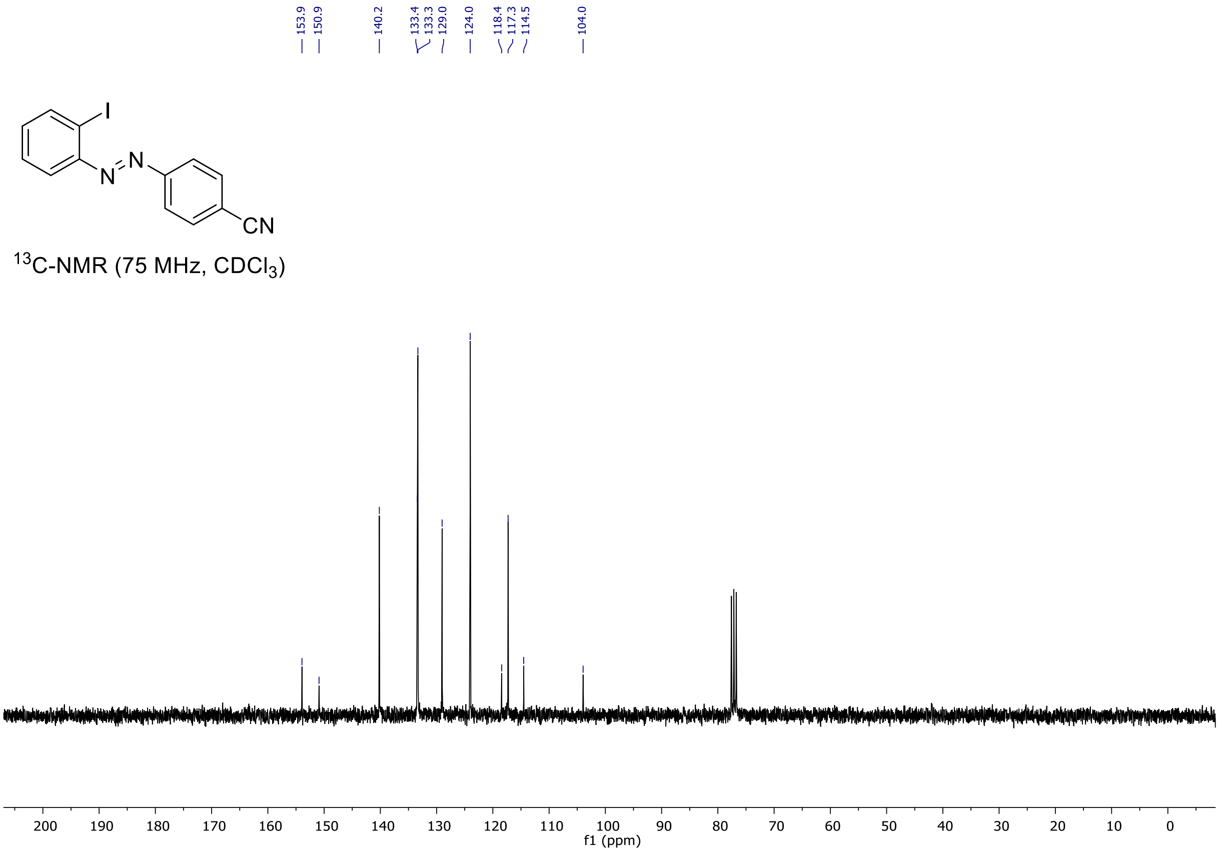

^{13}C-NMR (75 MHz, $CDCl_3$)

(*E*)-1-(2-Iodophenyl)-2-(3-nitrophenyl)diazene

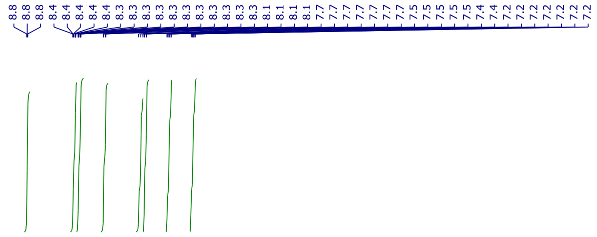

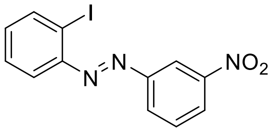

^{1}H-NMR (400 MHz, $CDCl_3$)

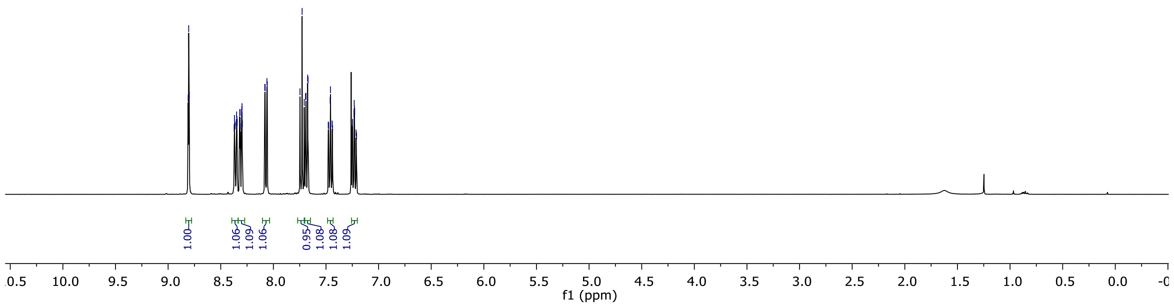

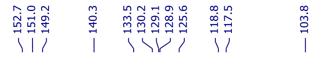

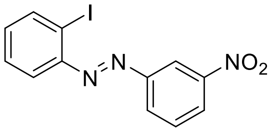

^{13}C-NMR (101 MHz, $CDCl_3$)

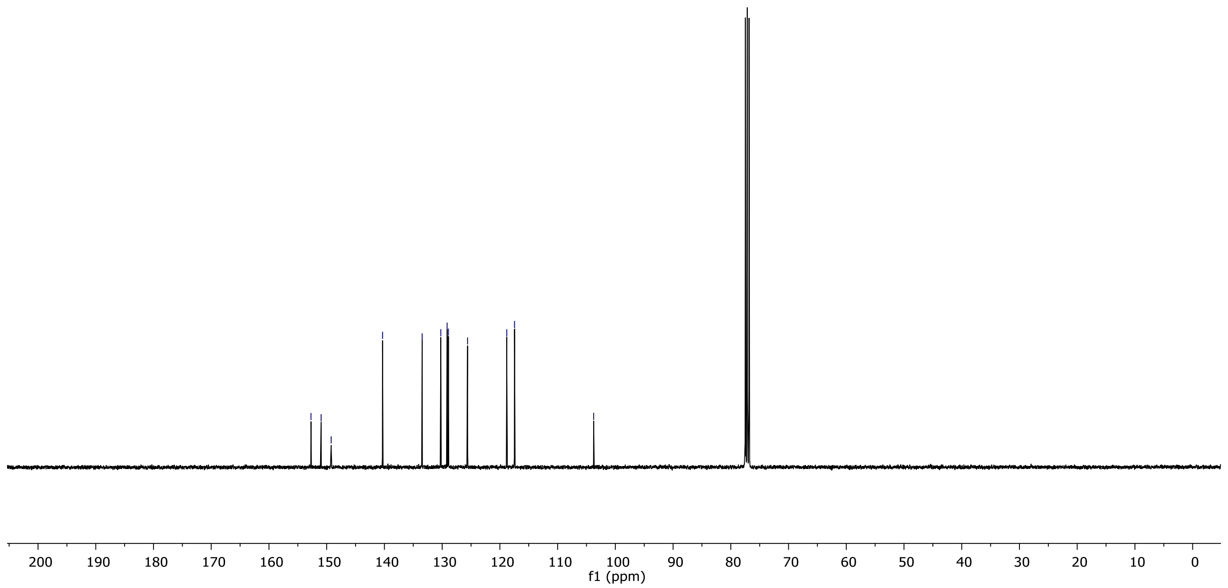

(*E*)-1-[4-((2-Iodophenyl)diazenyl)phenyl]ethan-1-one

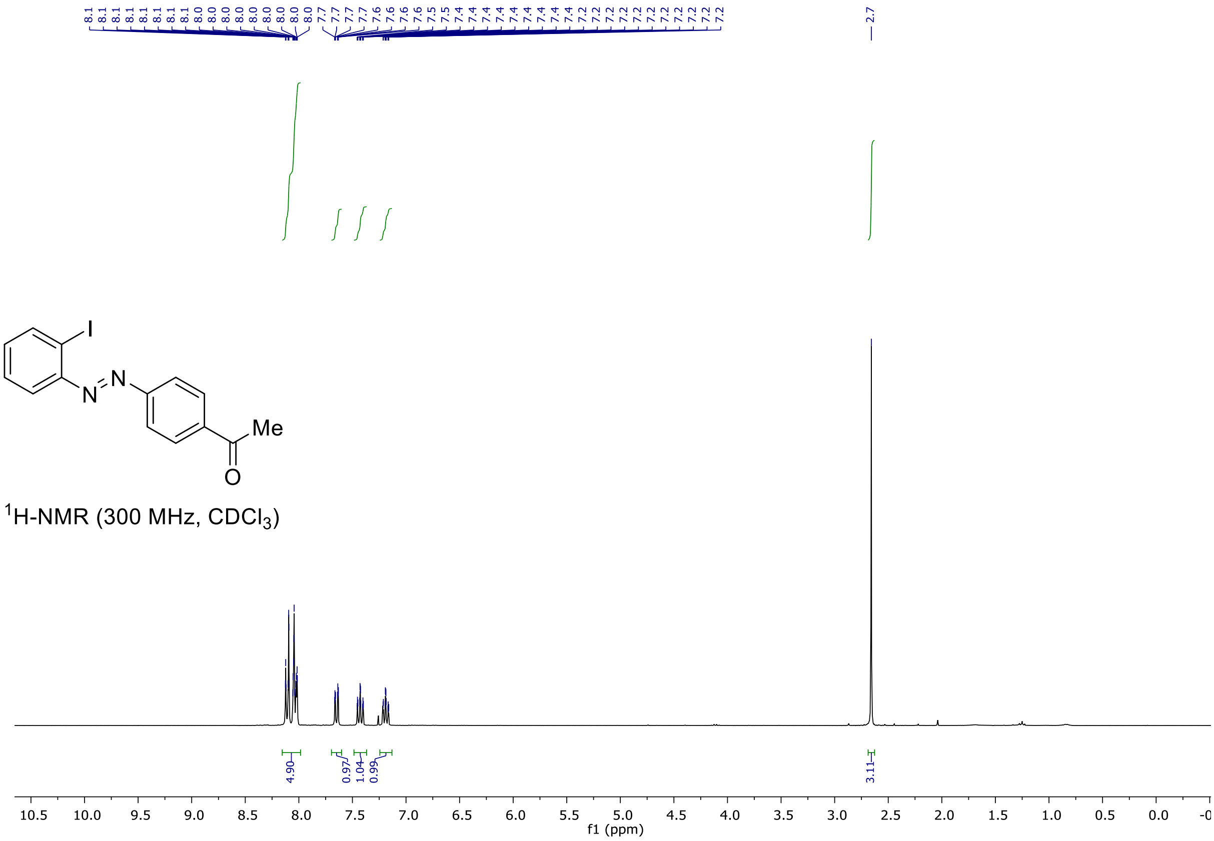

^{1}H-NMR (300 MHz, $CDCl_3$)

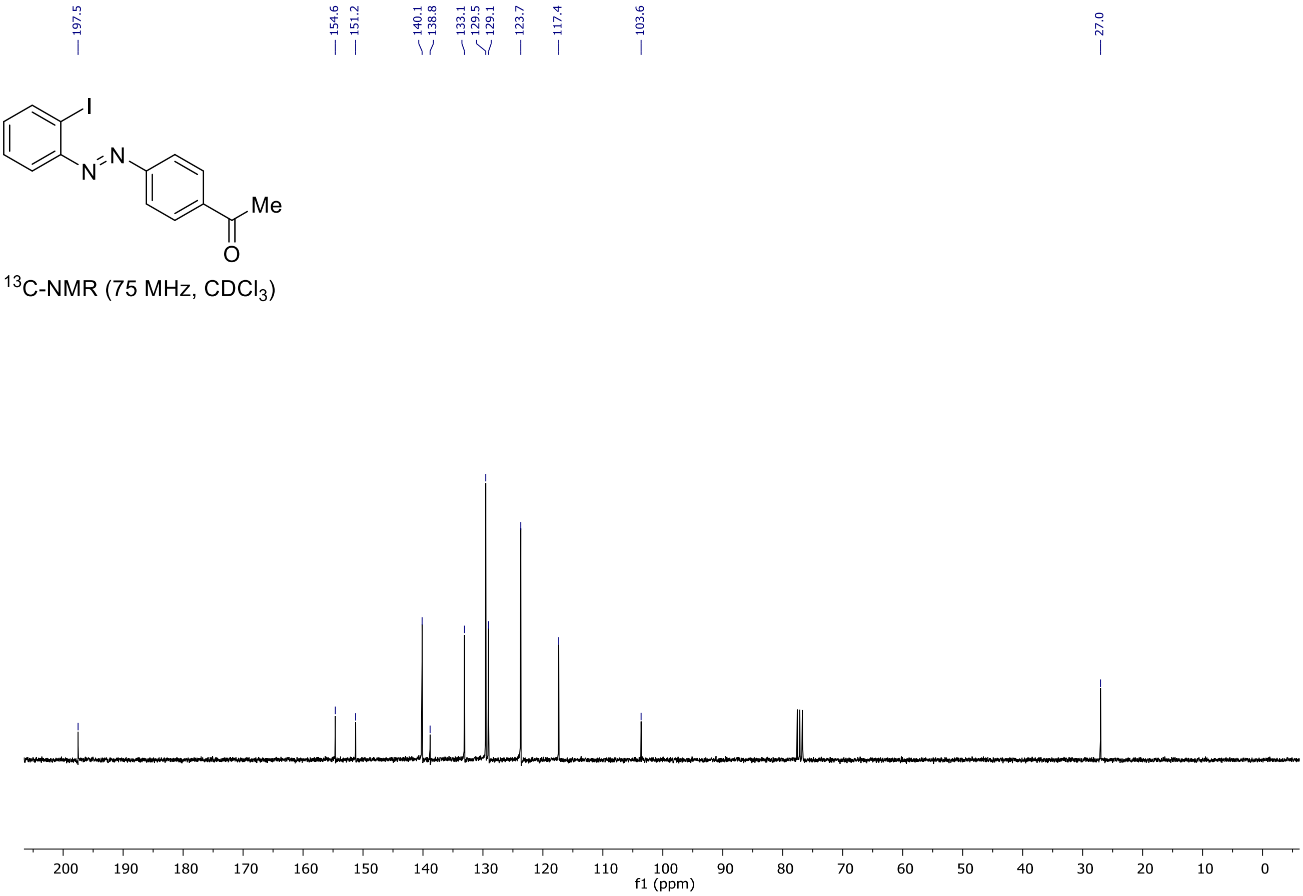

^{13}C-NMR (75 MHz, $CDCl_3$)

Methyl (*E*)-4-((2-iodophenyl)diazenyl)benzoate

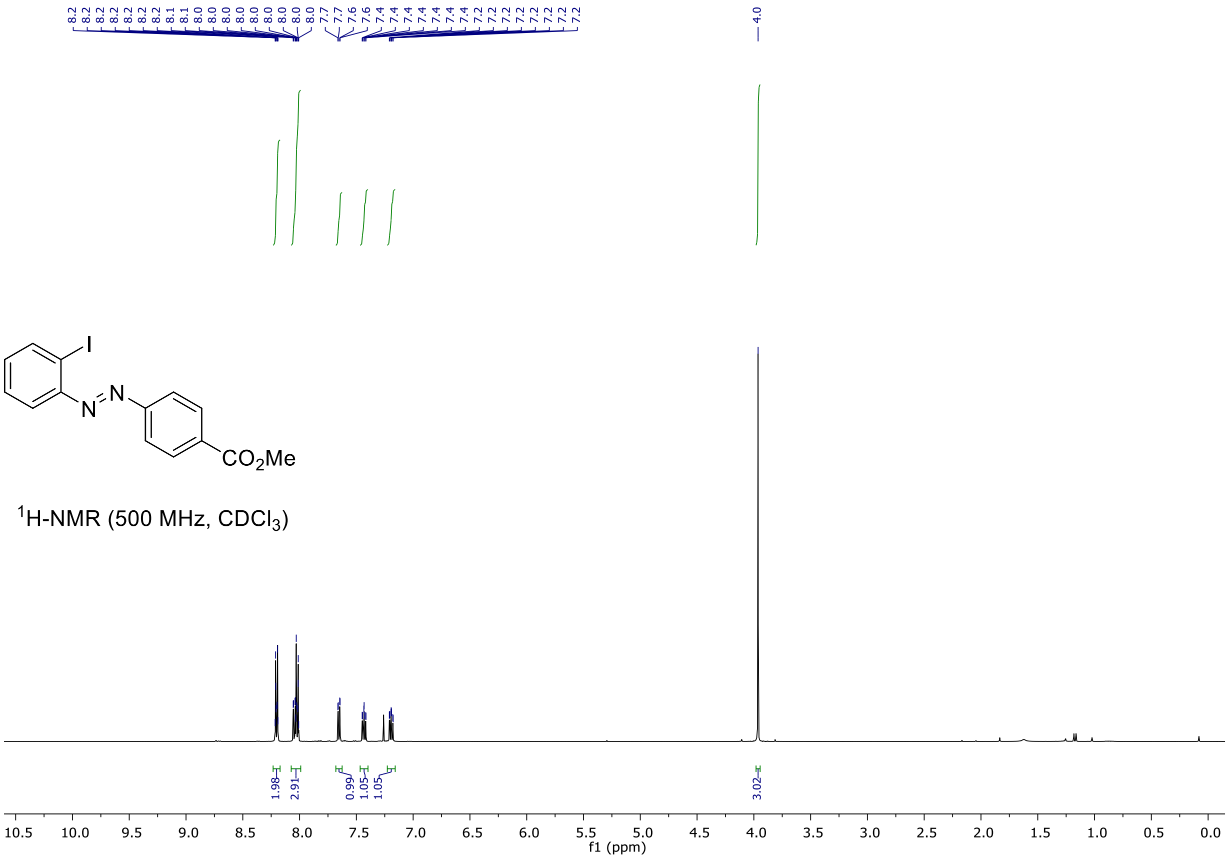

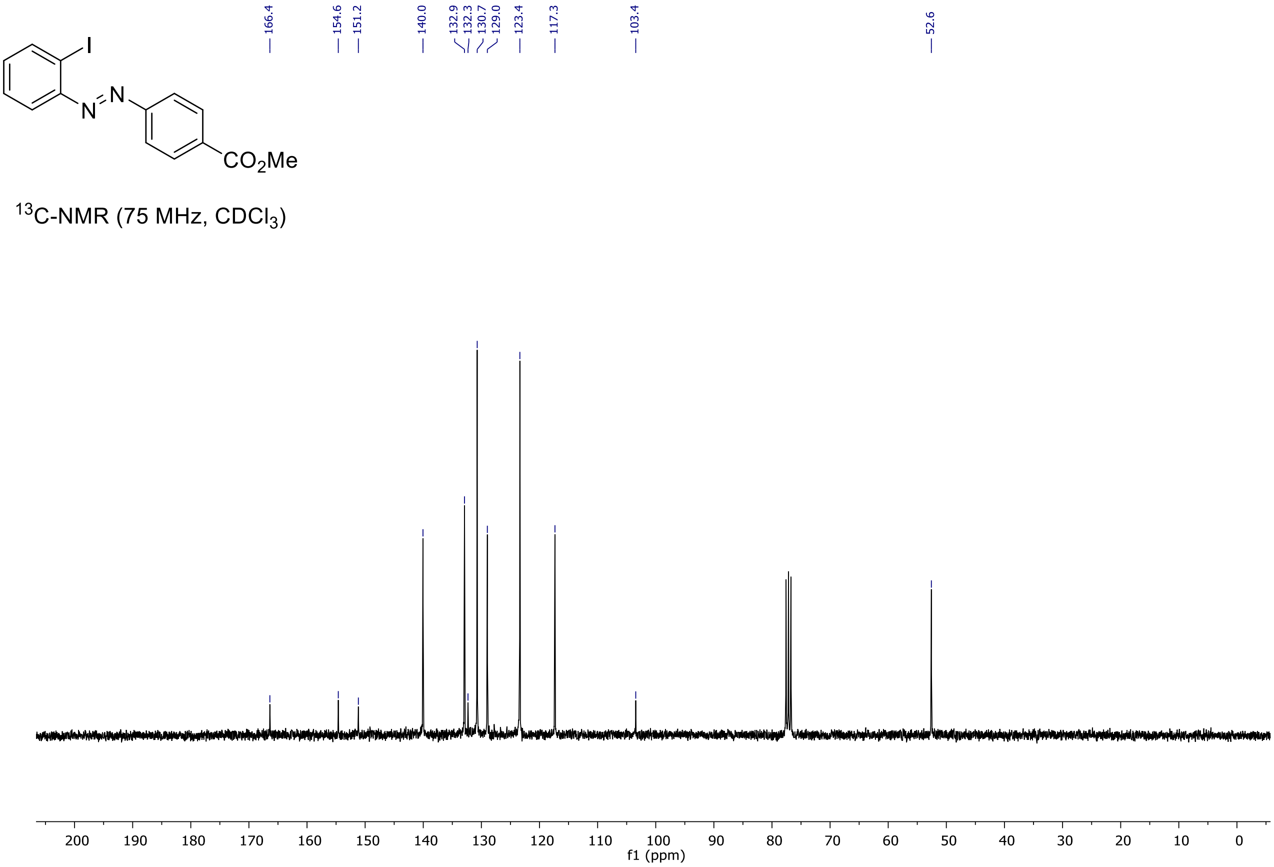

(*E*)-1-(2-Bromo-4-methylphenyl)-2-(2-iodophenyl)diazene (3l)

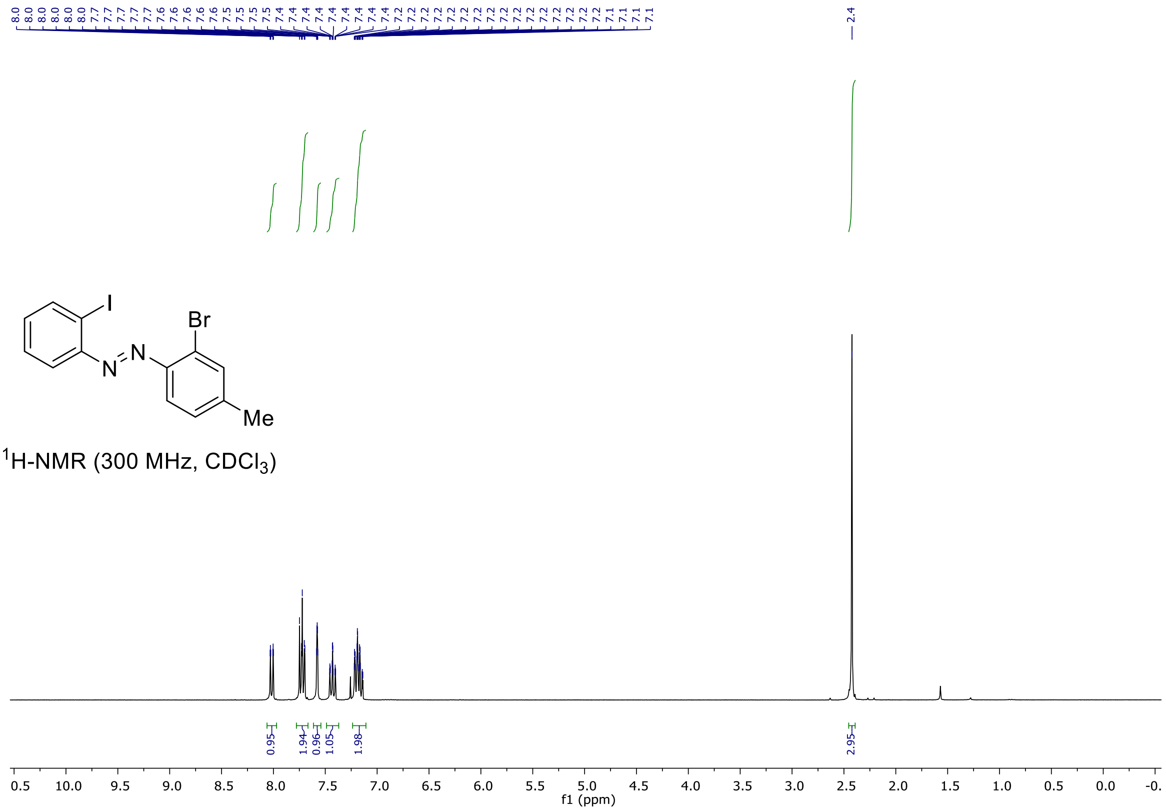

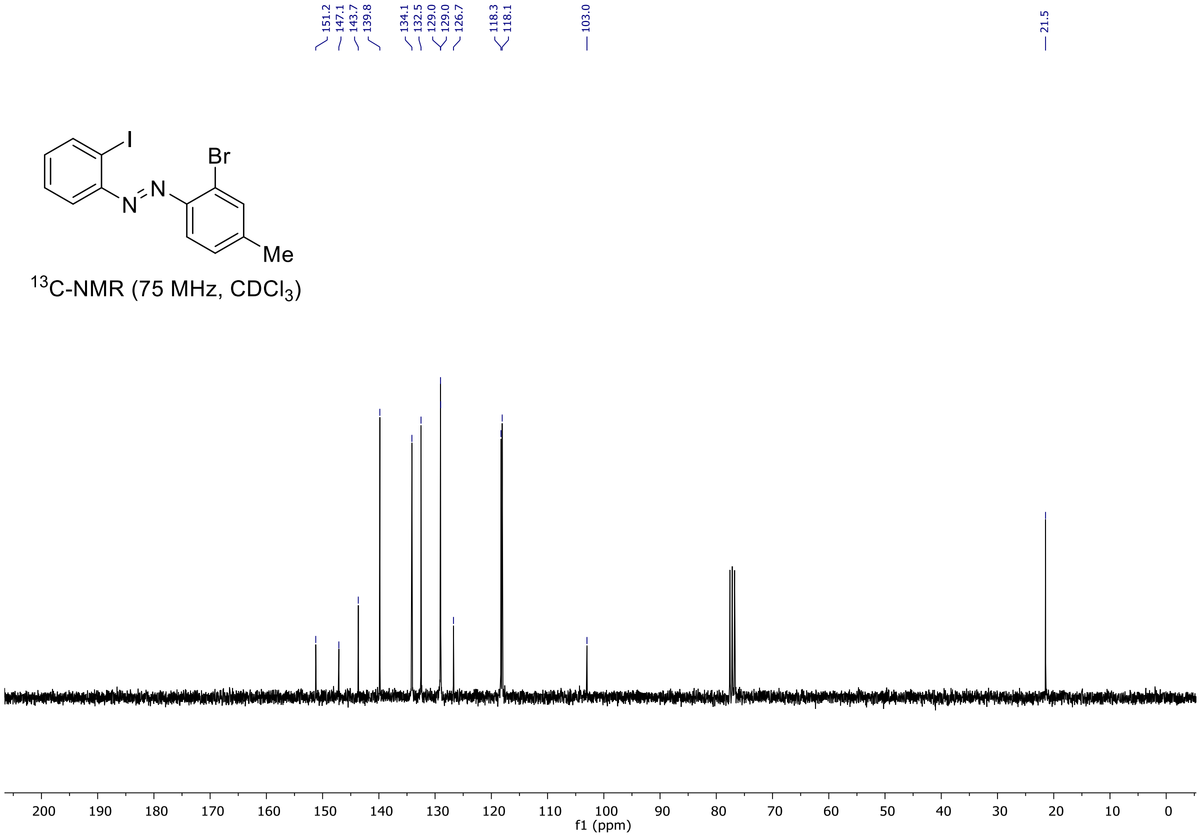

Dimethyl (*E*)-5-[(2-iodophenyl)diazinyl]isophthalate

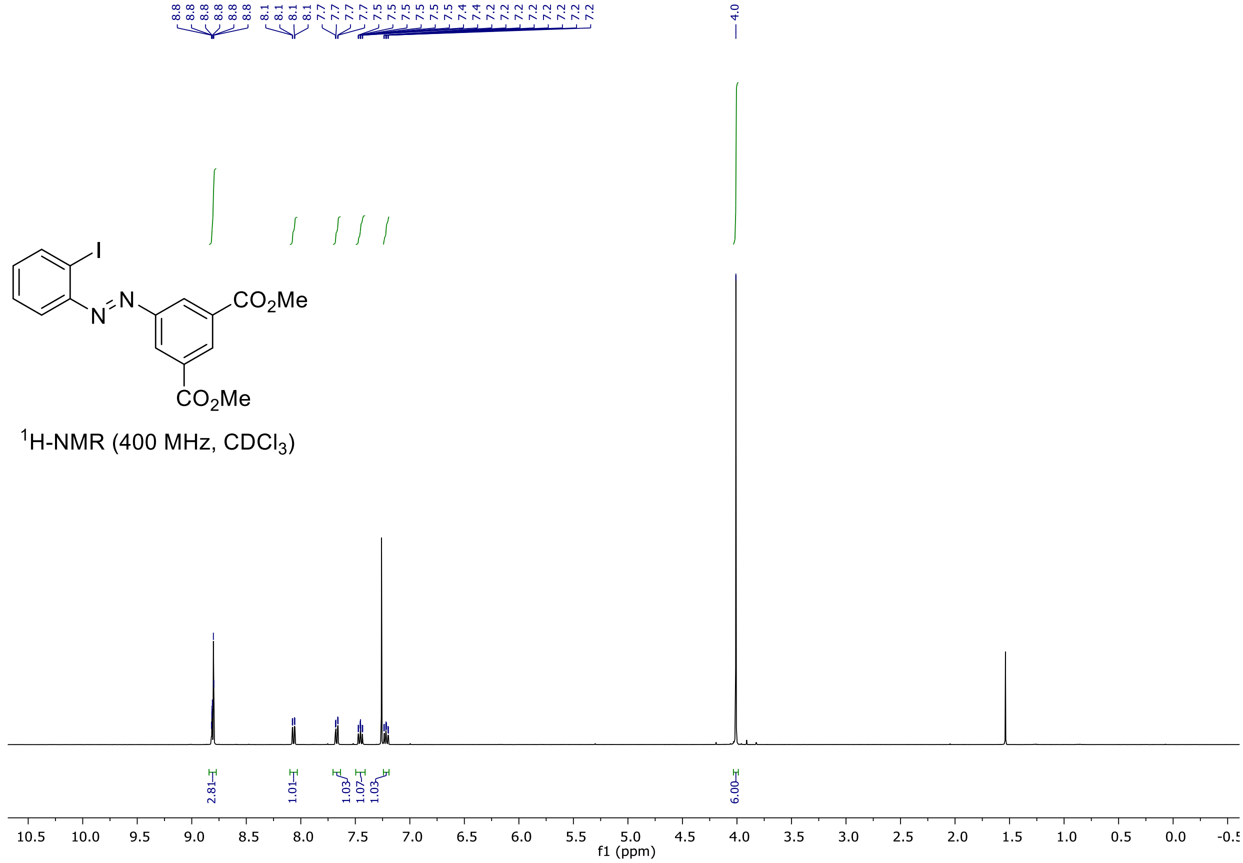

^{1}H-NMR (400 MHz, $CDCl_3$)

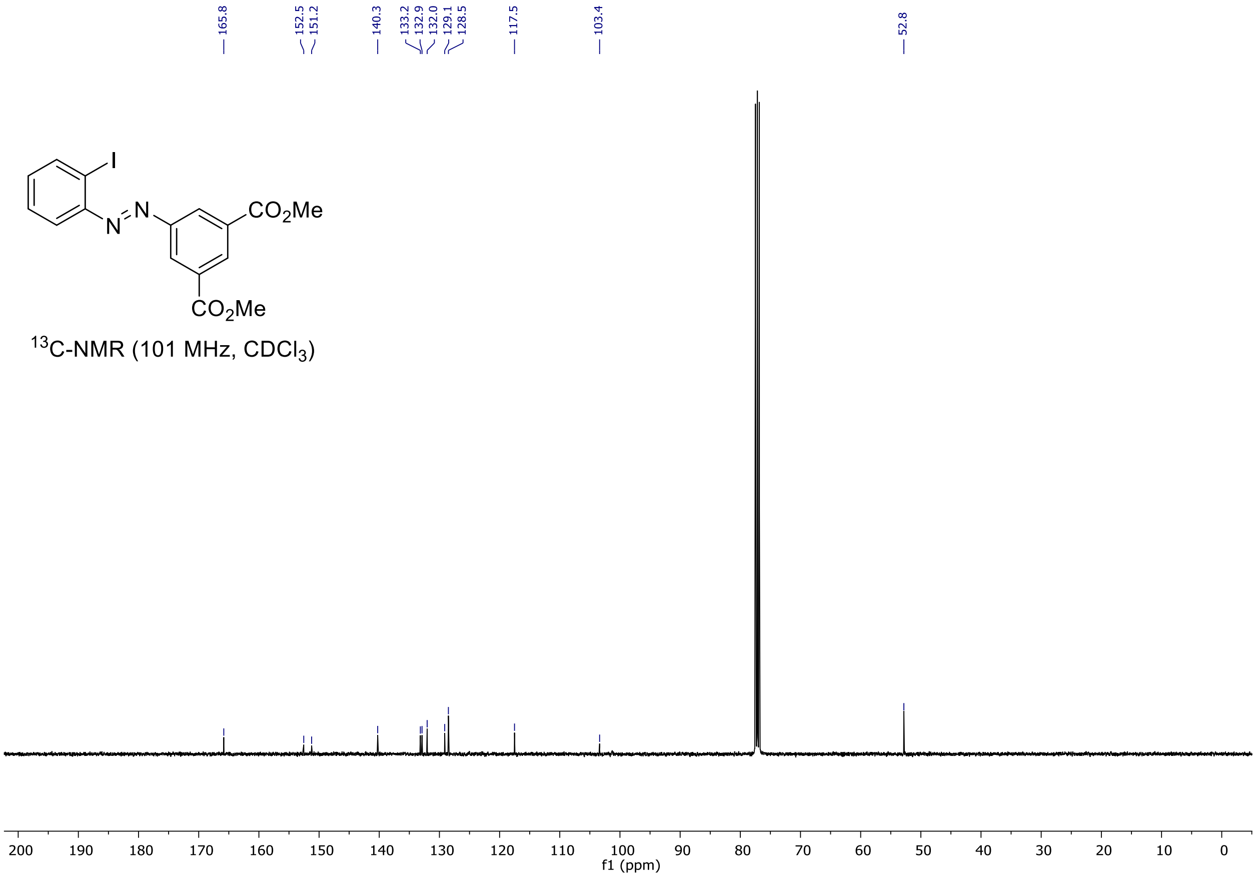

^{13}C-NMR (101 MHz, $CDCl_3$)

(*E*)-1-(2-Iodopjenyl)-2-(*p*-tolyl)diazene

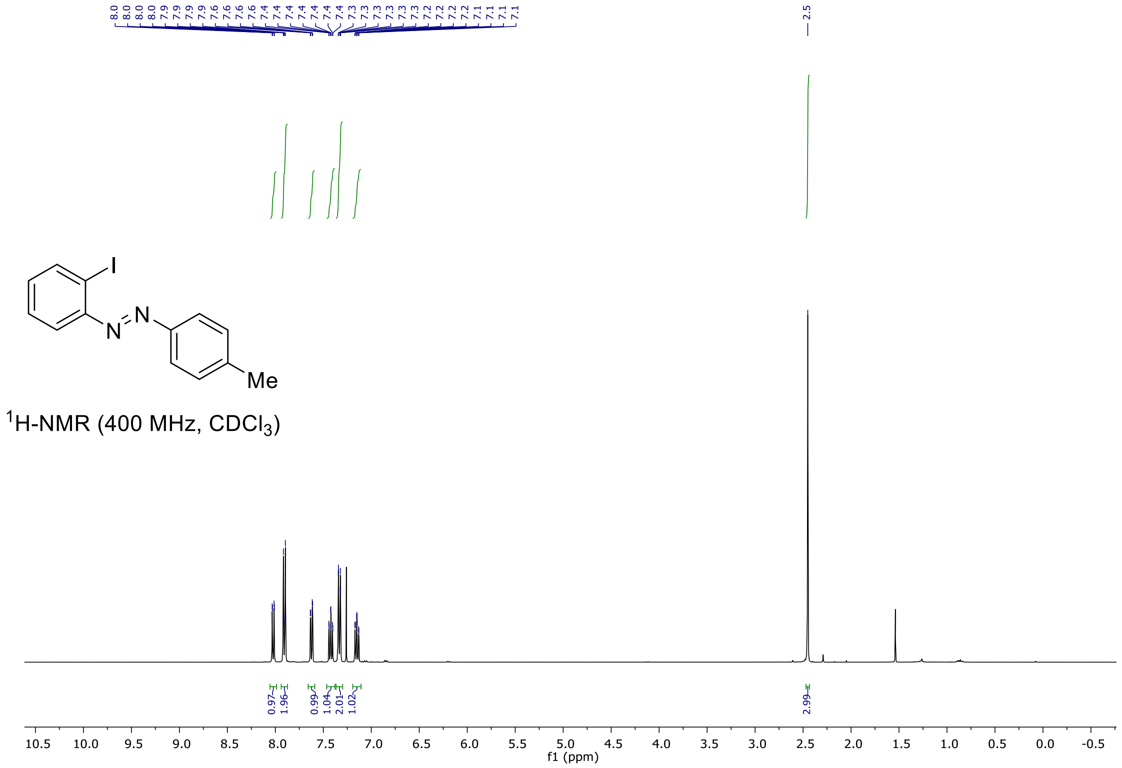

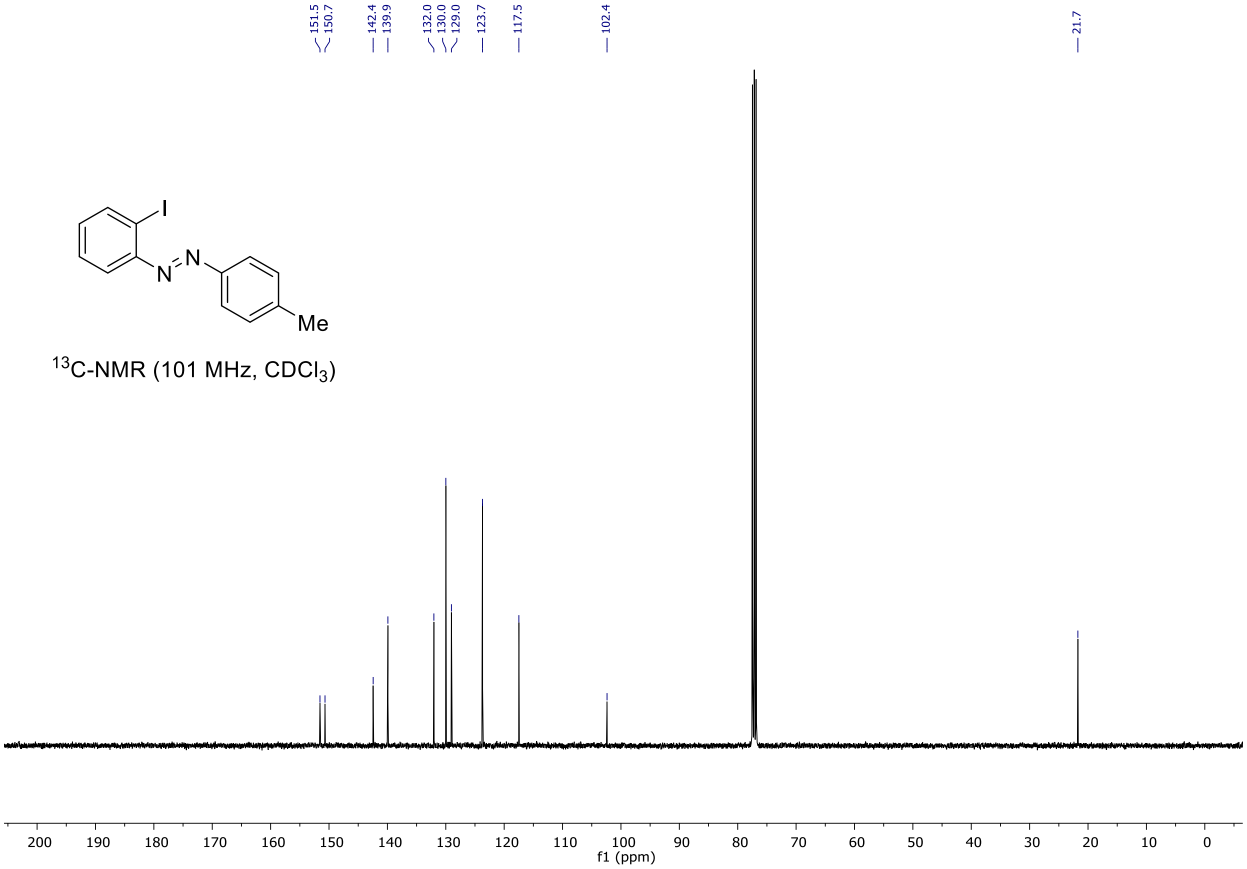

(*E*)-N-[4-((2-iodophenyl)diazenyl)phenyl]-N-methylacetamide

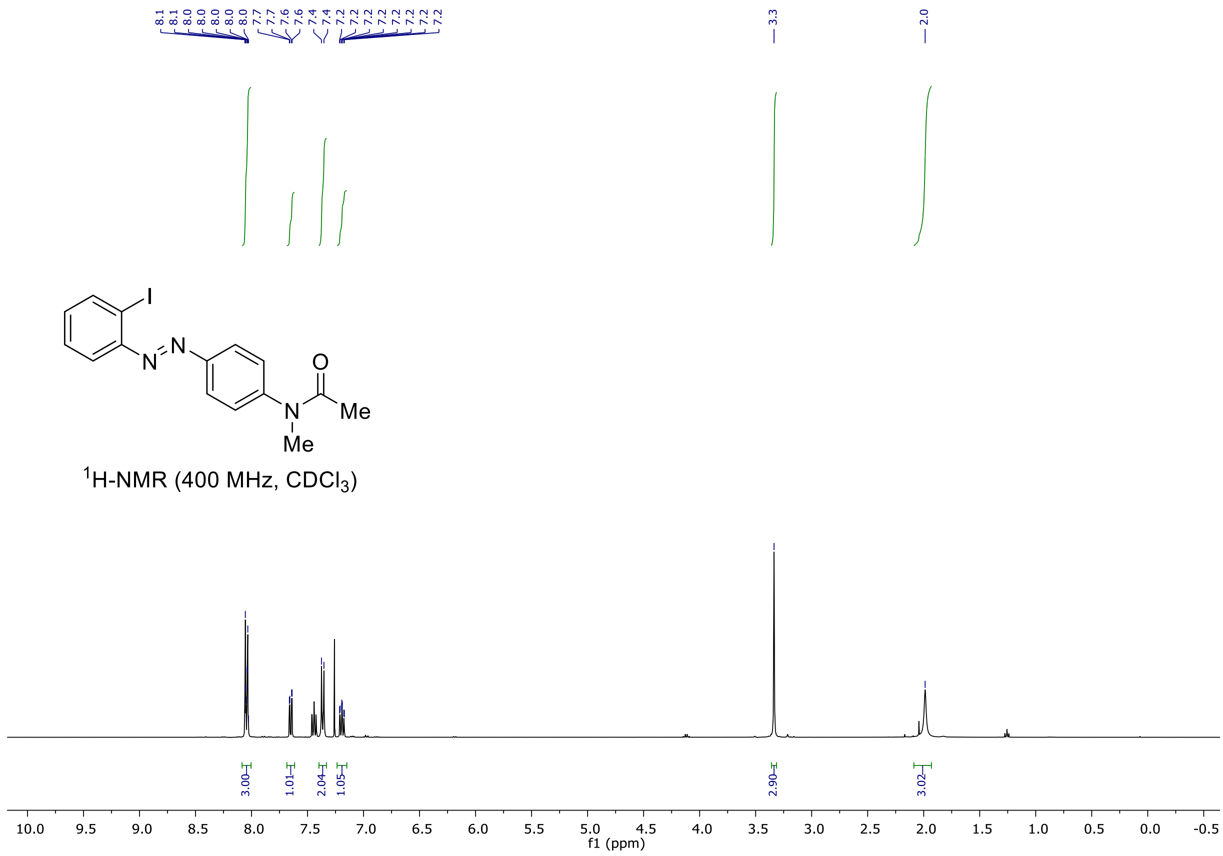

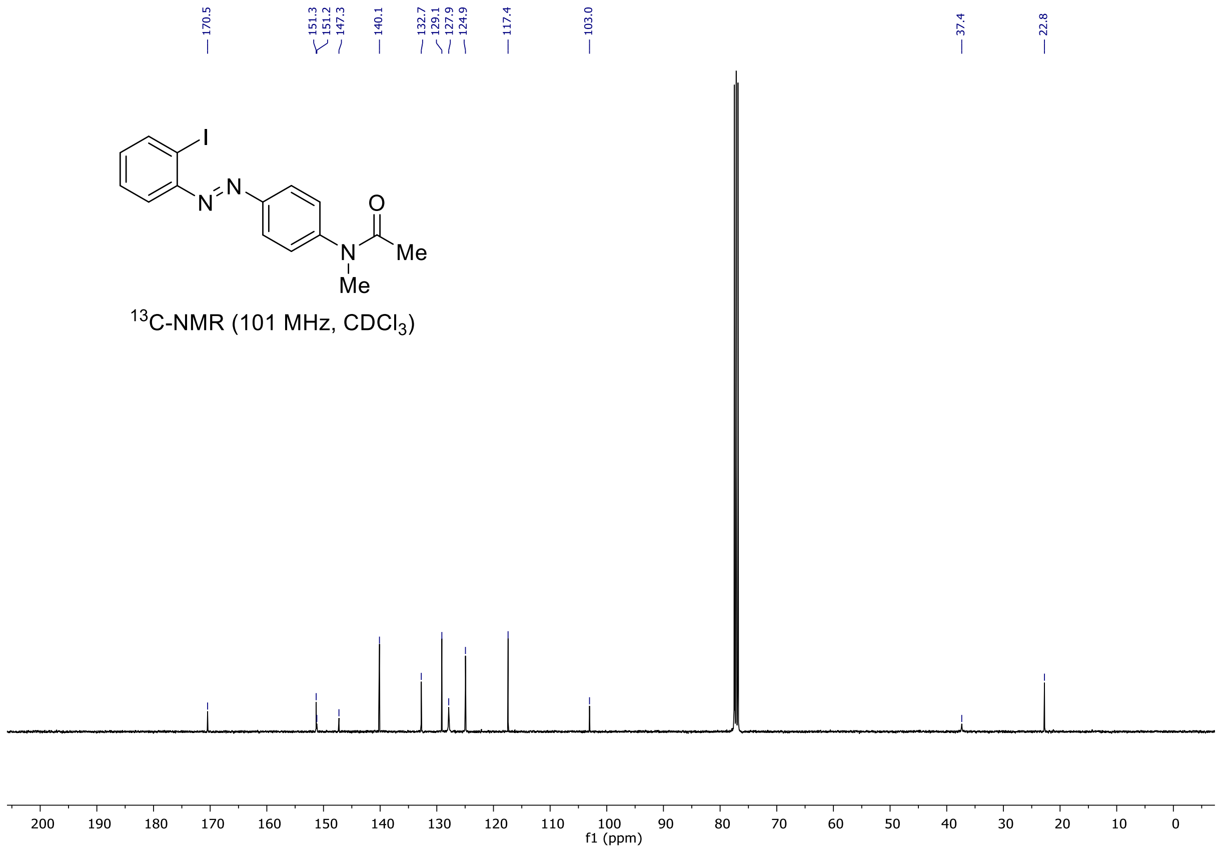

(*E*)-4-[(2-Iodophenyl)diazenyl]phenol

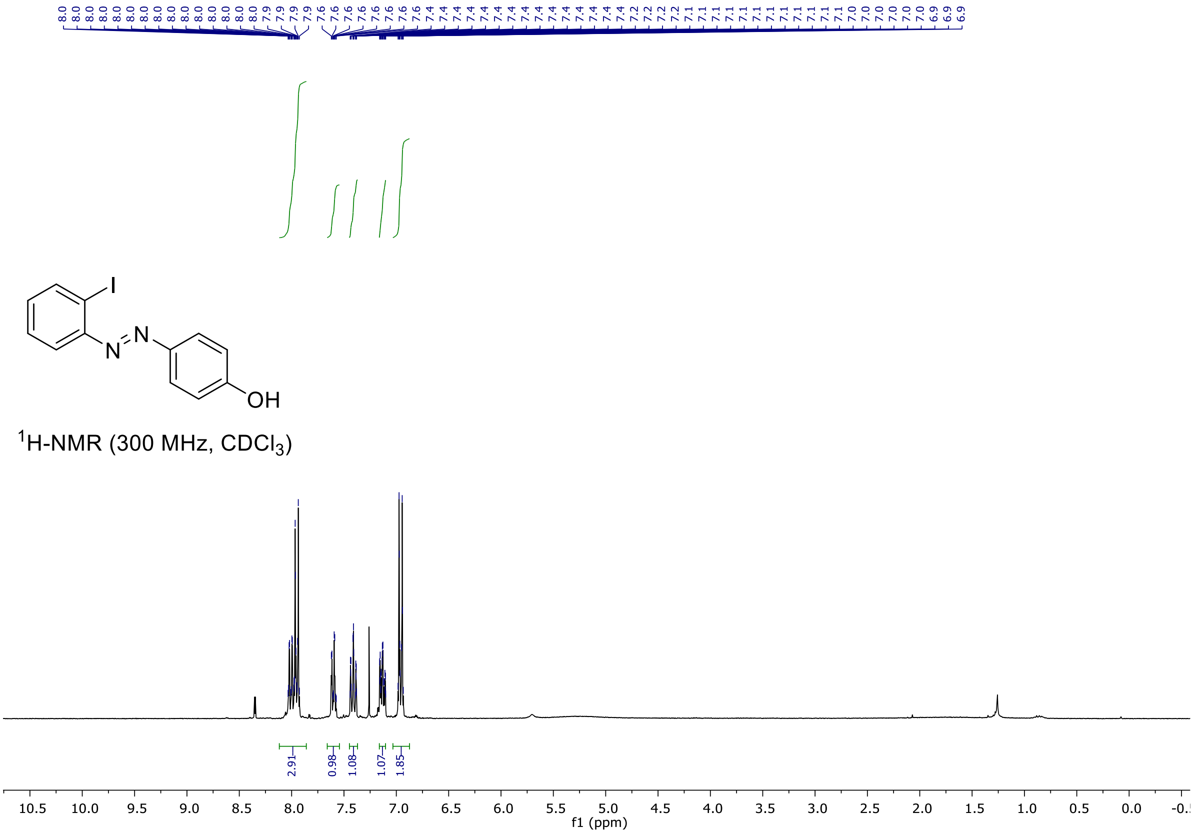

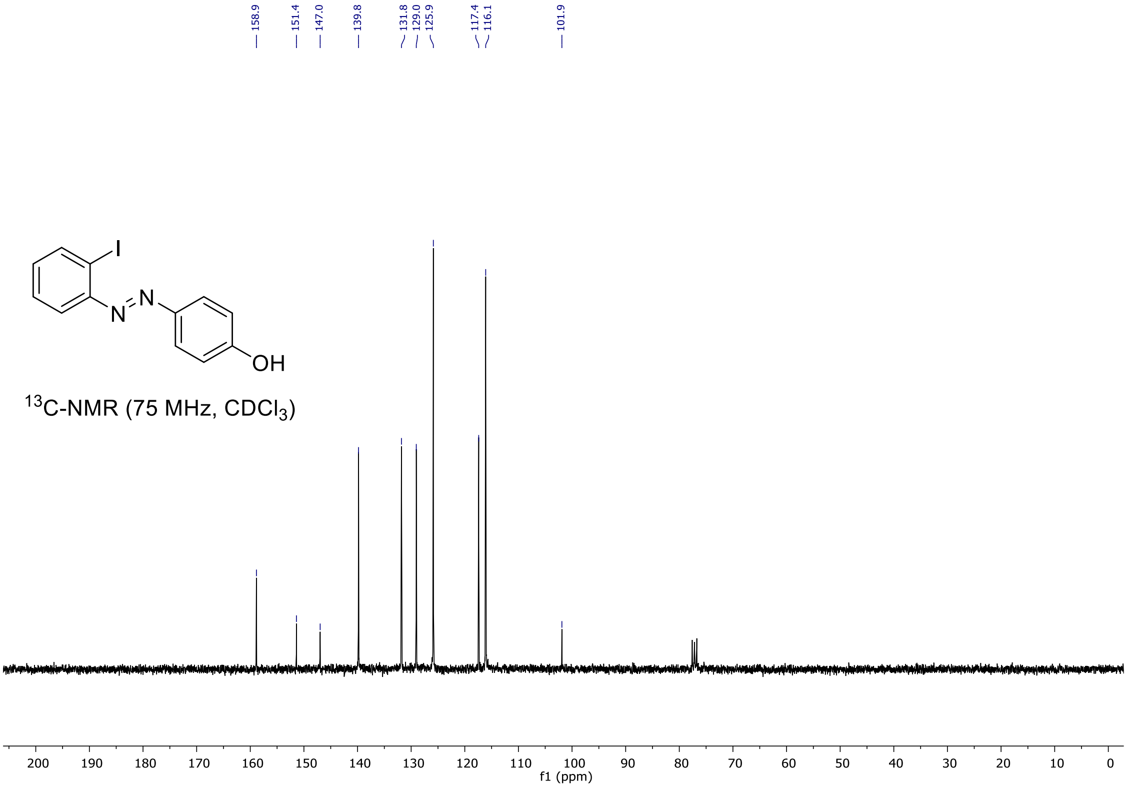

(*E*)-1-(2-Iodophenyl)-2-(4-methoxyphenyl)diazene

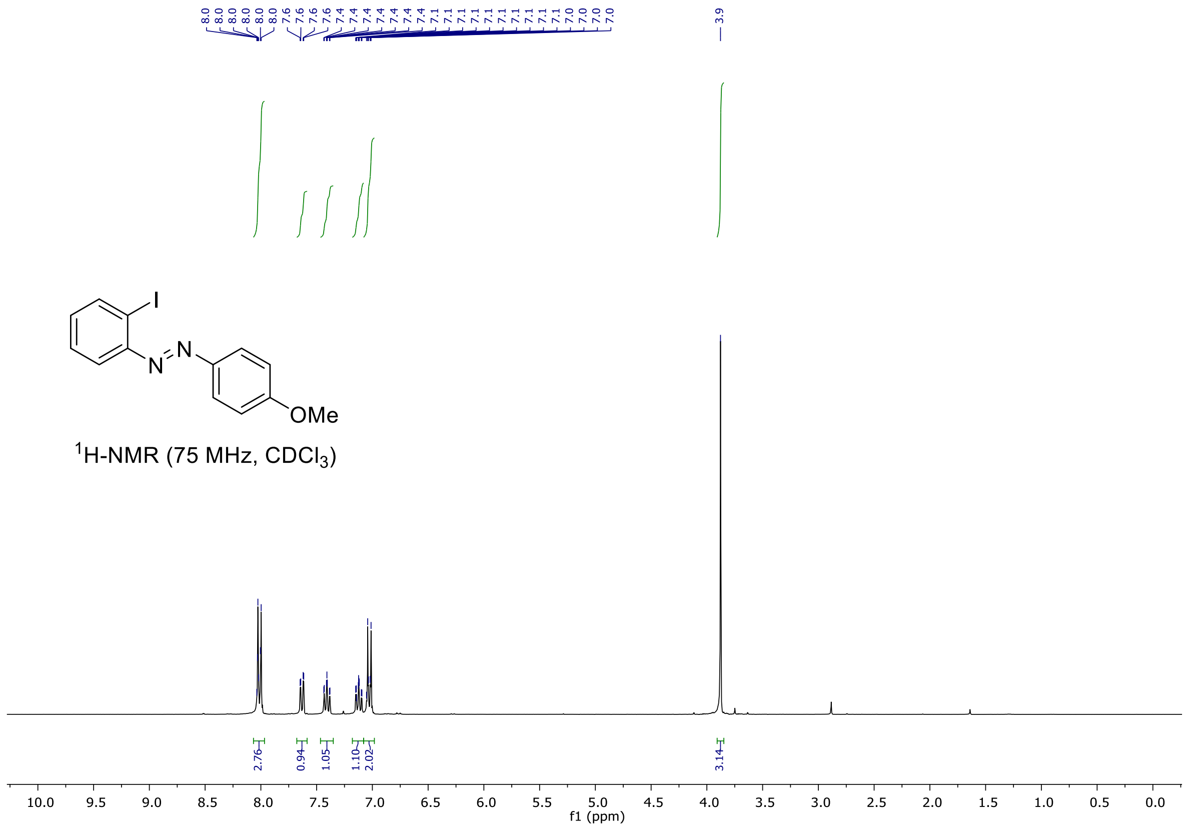

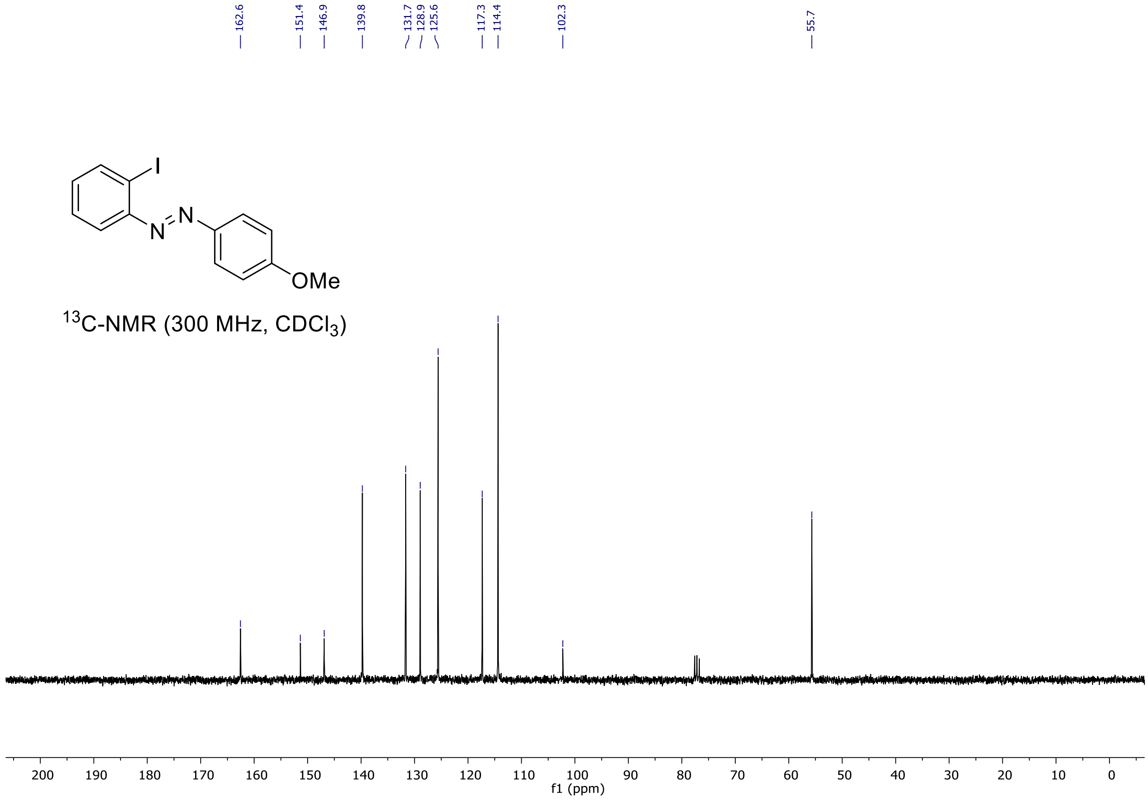

(*E*)-4-[(2-iodophenyl)diazenyl]phenyl acetate

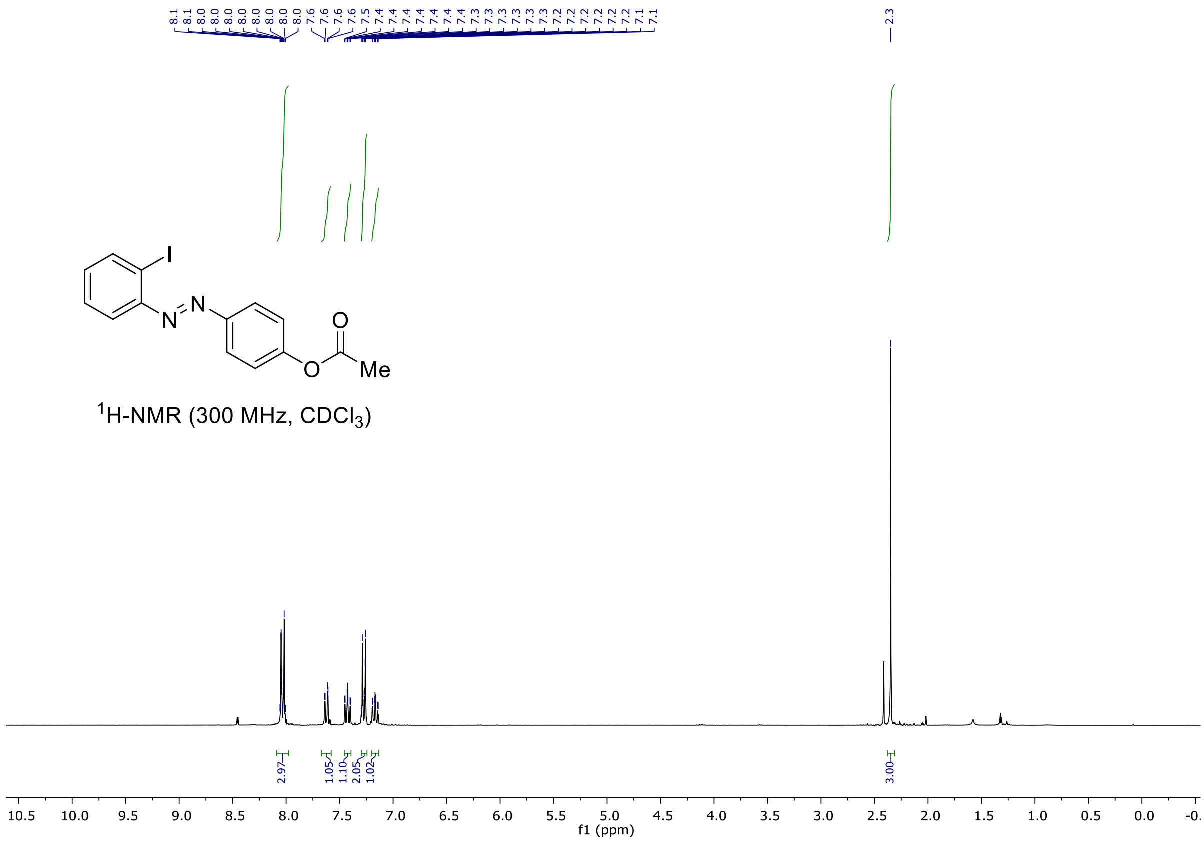

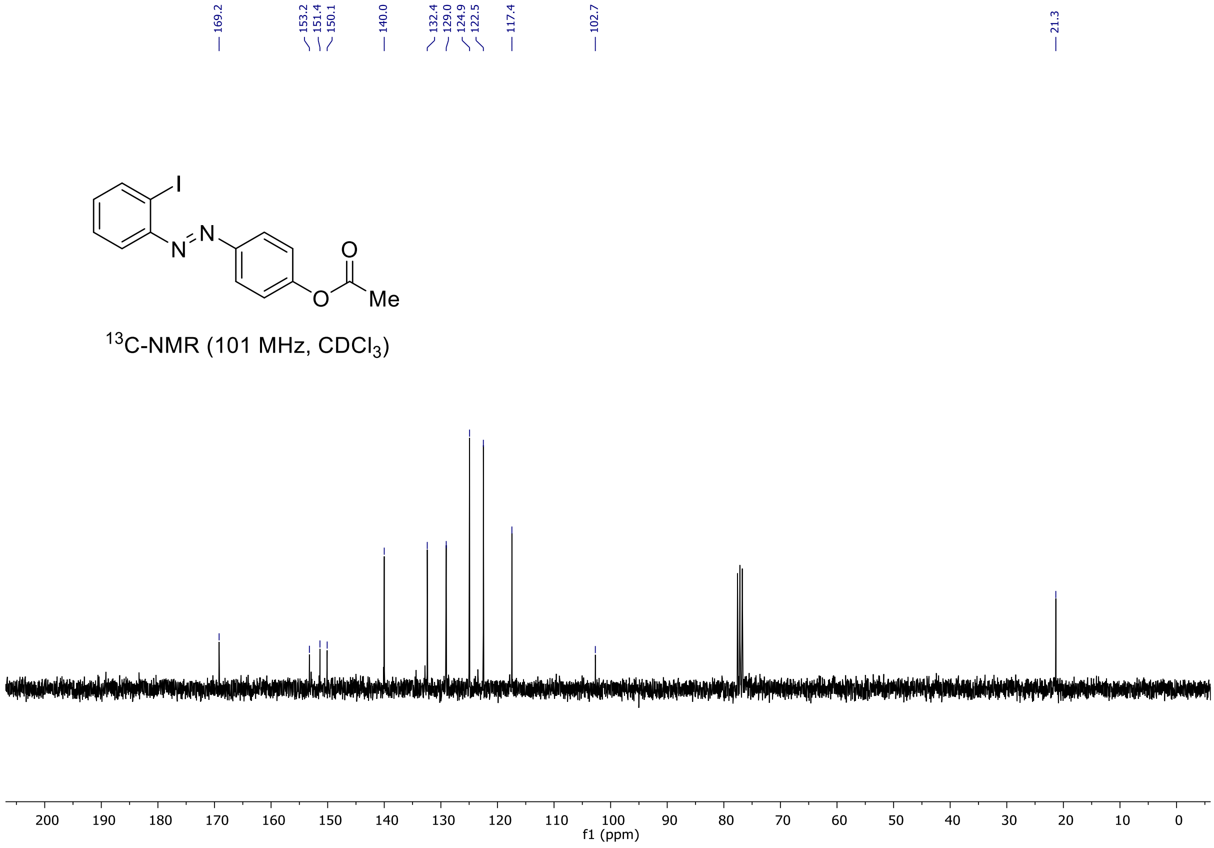

(*E*)-1-(5-Chloro-2-iodophenyl)-2-phenyldiazene

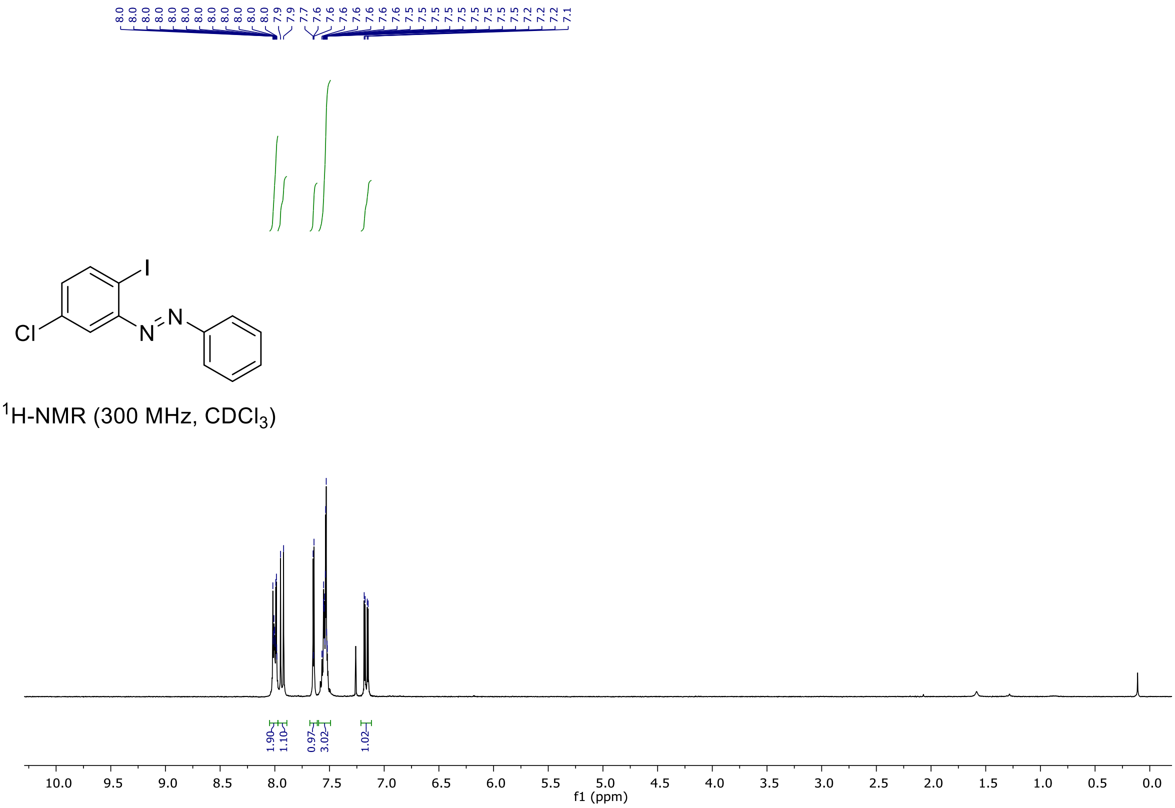

^{1}H-NMR (300 MHz, $CDCl_3$)

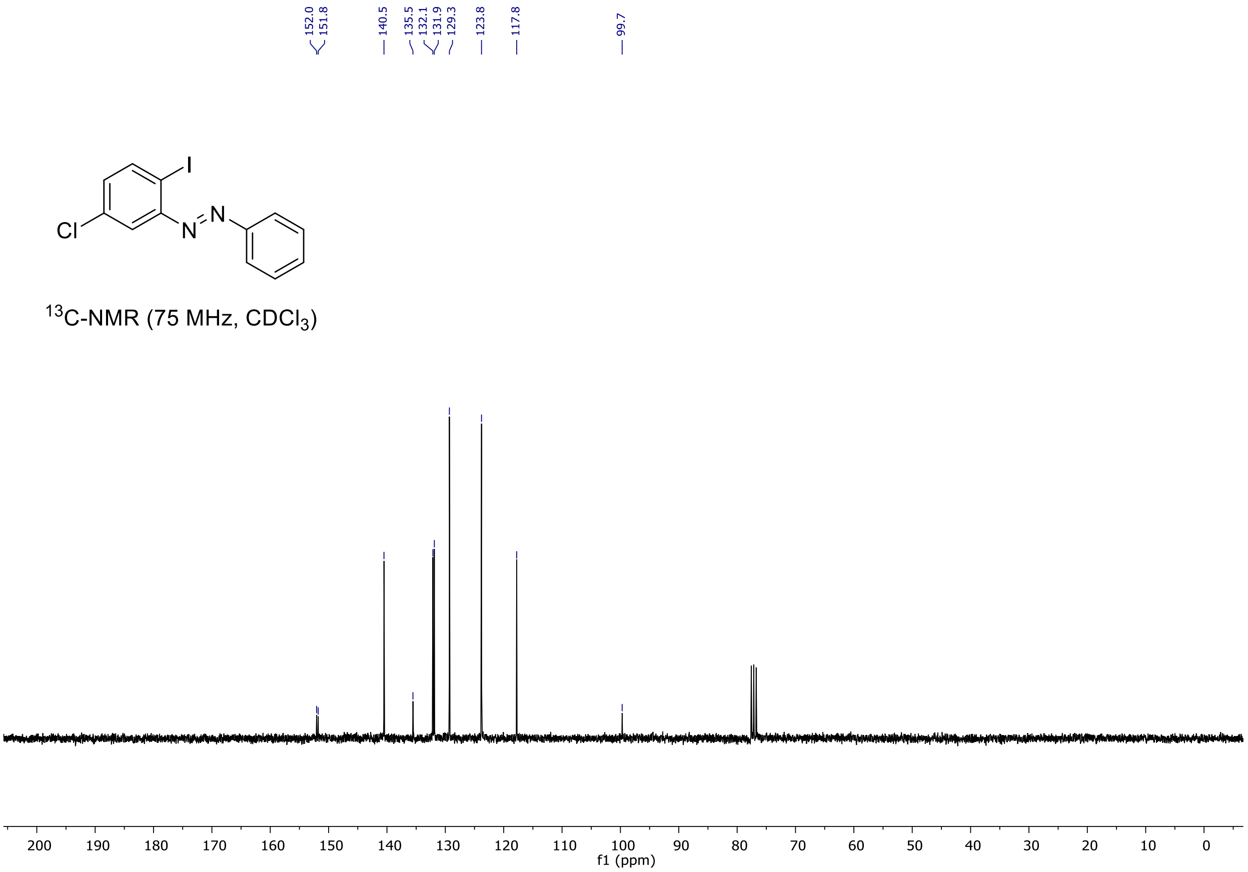

^{13}C-NMR (75 MHz, $CDCl_3$)

(*E*)-1-(2-Iodo-4-(trifluoromethyl)phenyl)-2-phenyldiazene

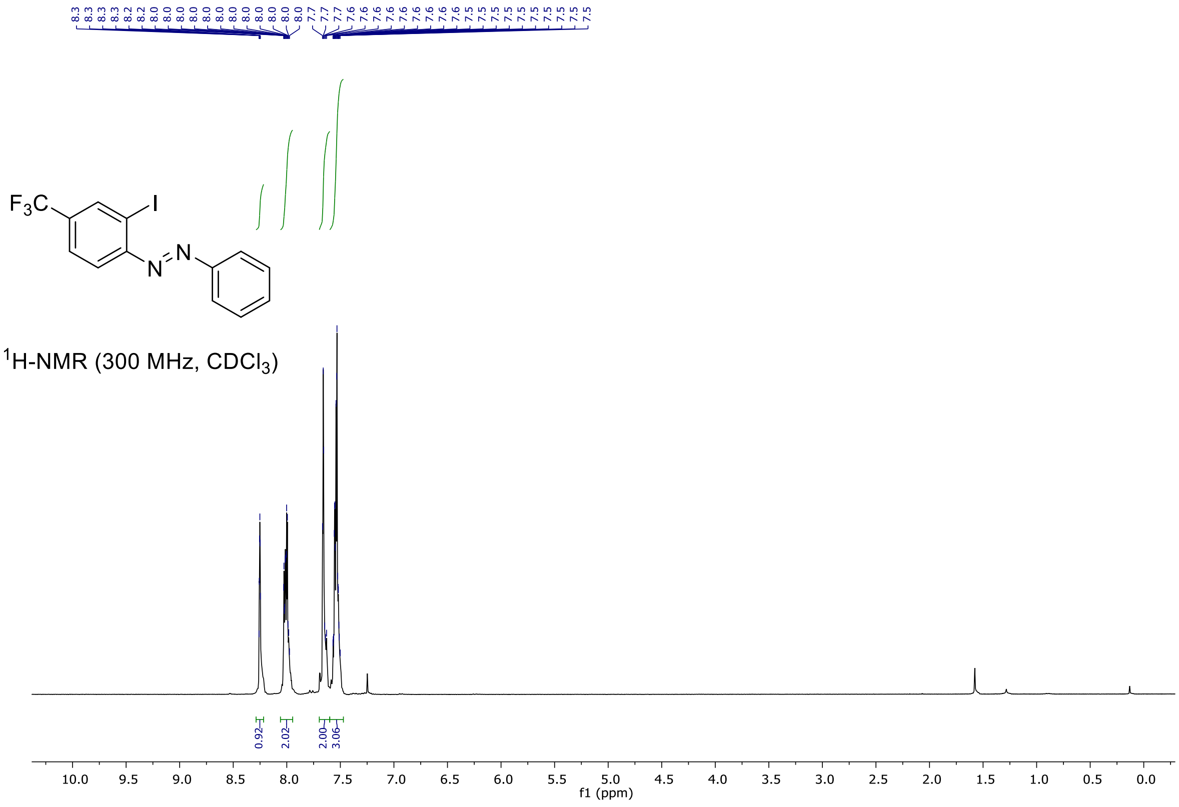

^{1}H-NMR (300 MHz, $CDCl_3$)

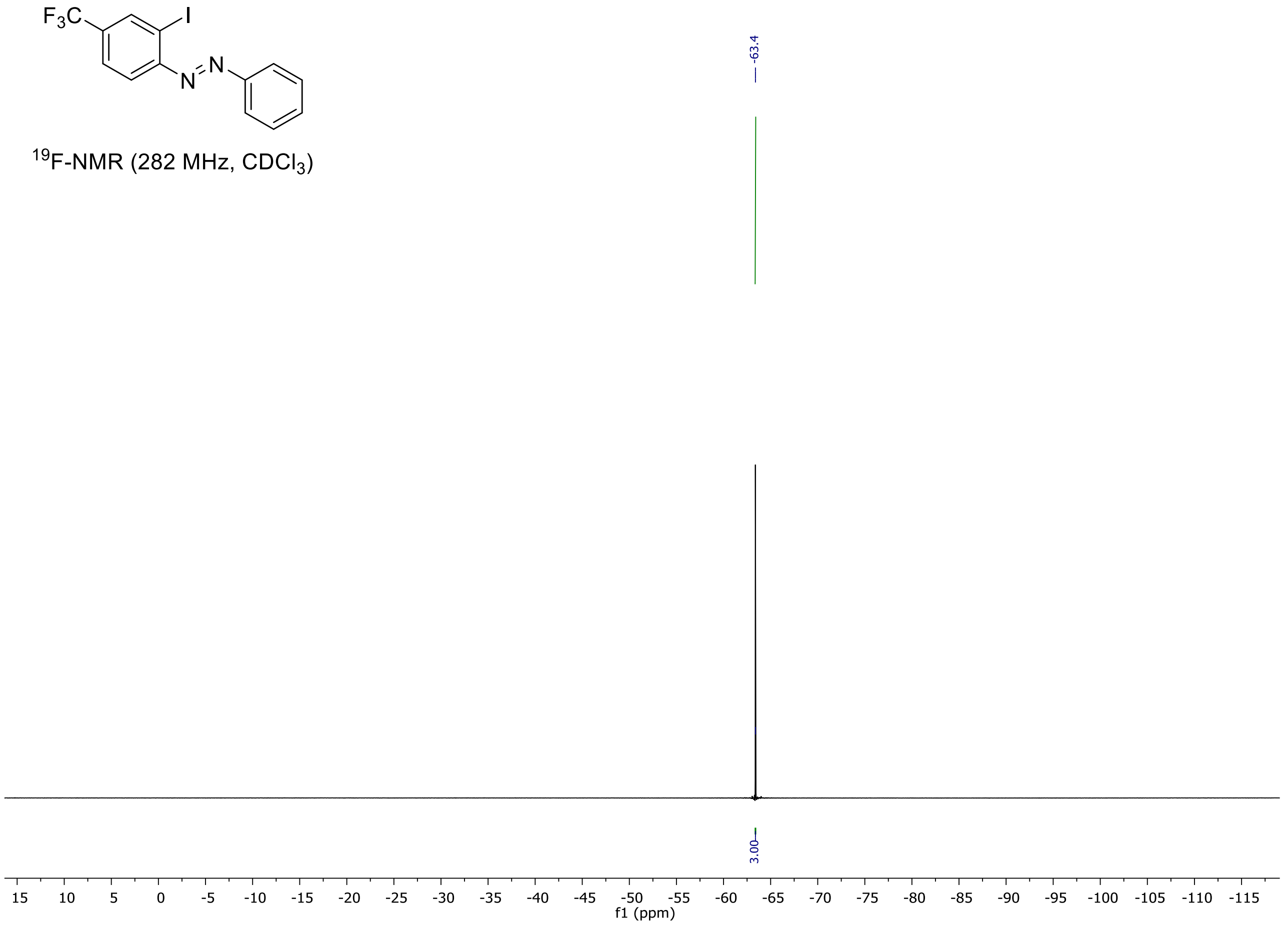

^{19}F-NMR (282 MHz, $CDCl_3$)

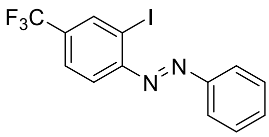

^{13}C-NMR (75 MHz, $CDCl_3$)

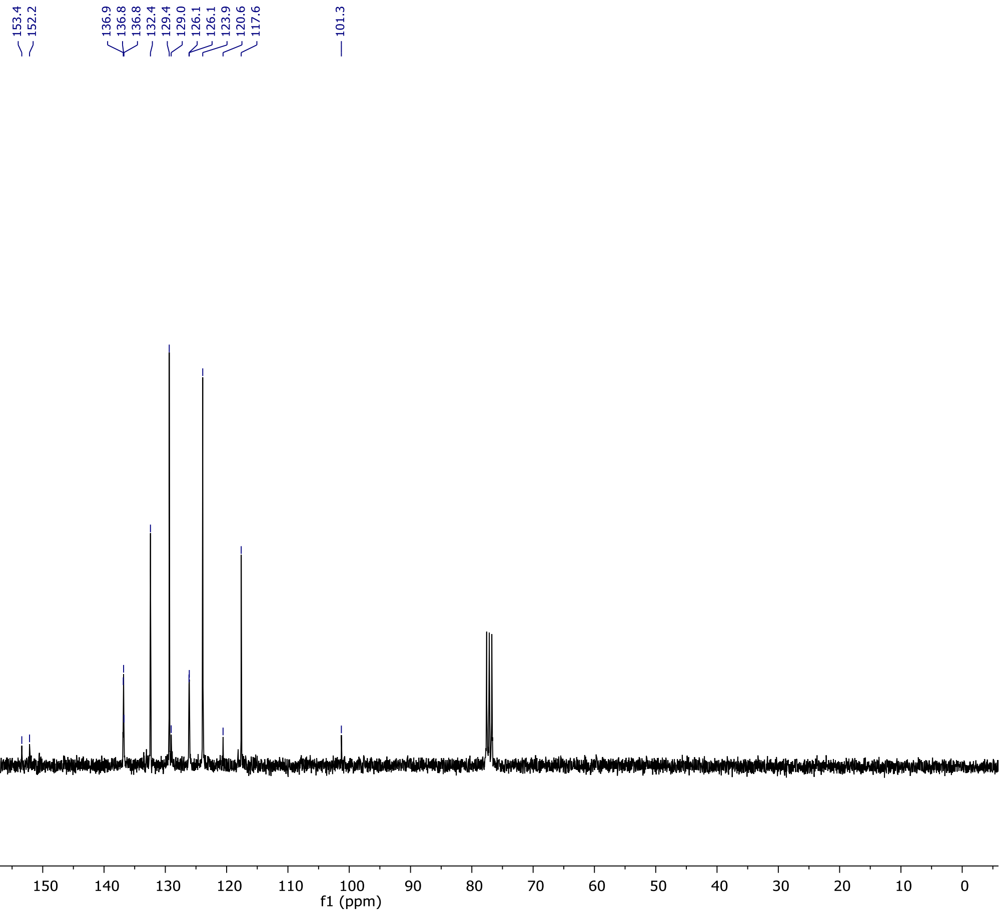

Methyl (*E*)-3-iodo-4-(phenyldiazenyl)benzoate

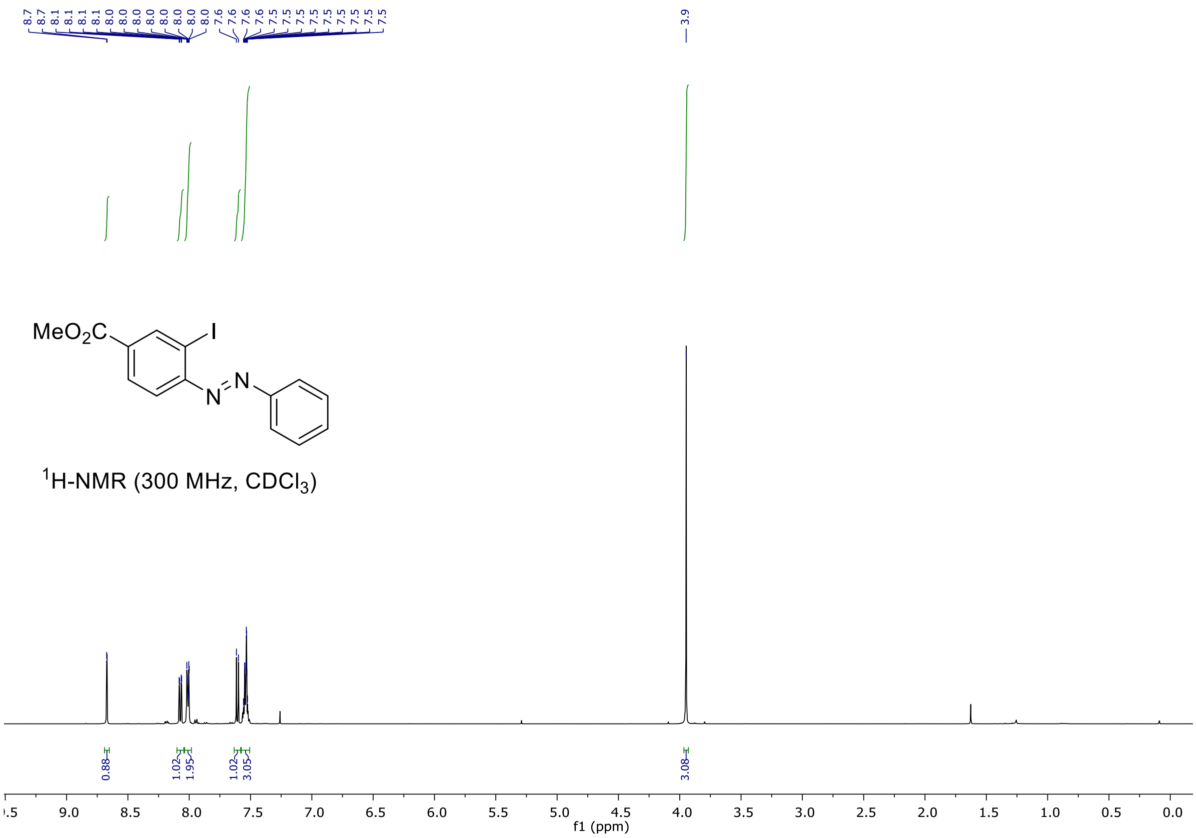

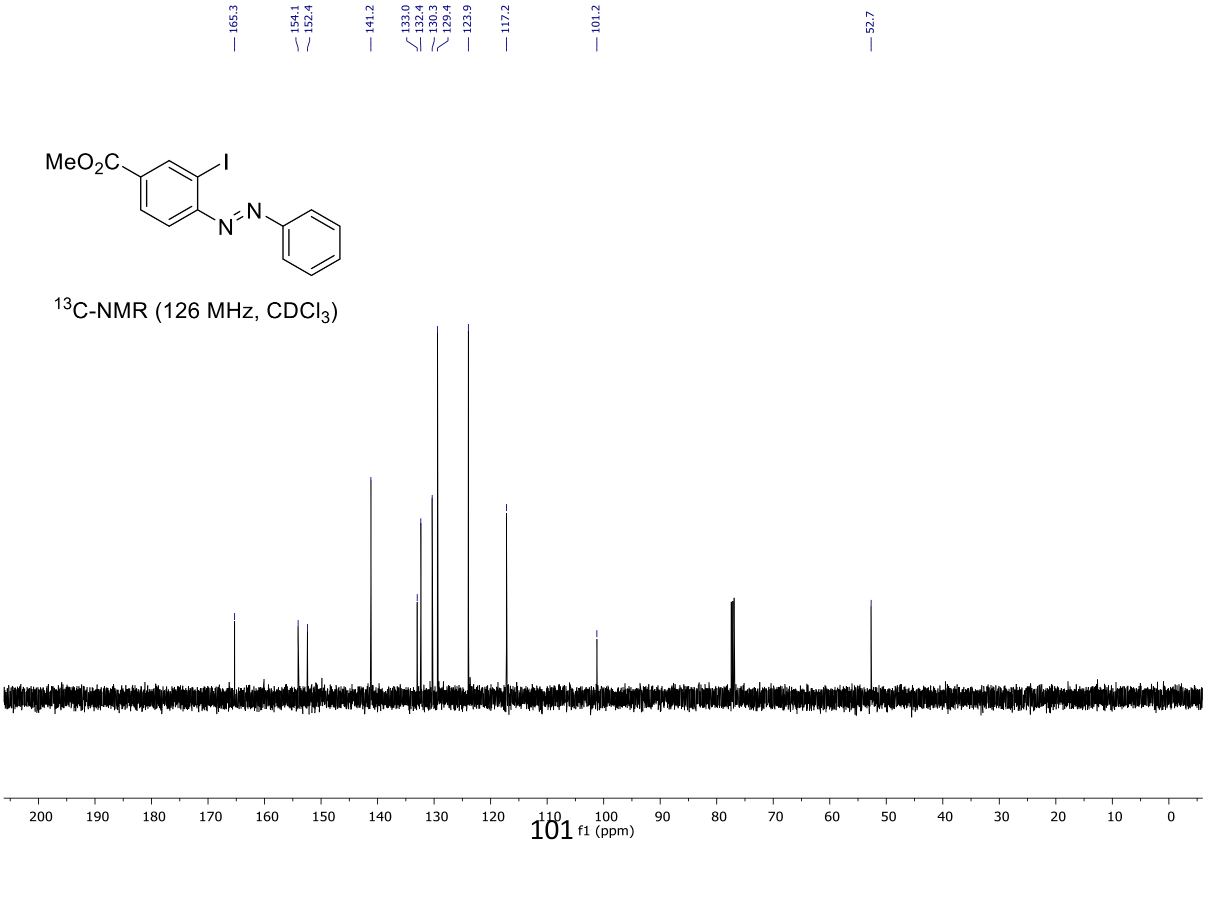

(*E*)-1-(2-Iodo-4-methylphenyl)-2-phenyldiazene

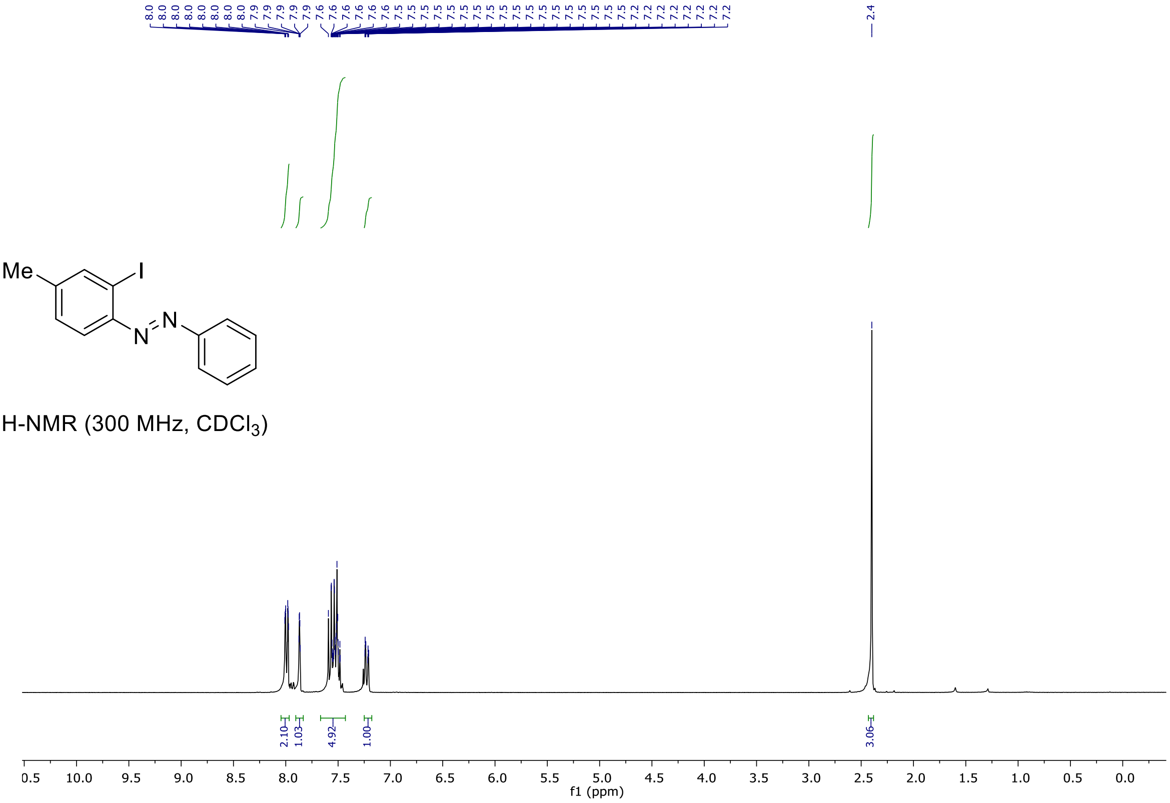

^{1}H-NMR (300 MHz, $CDCl_3$)

^{13}C-NMR (75 MHz, $CDCl_3$)

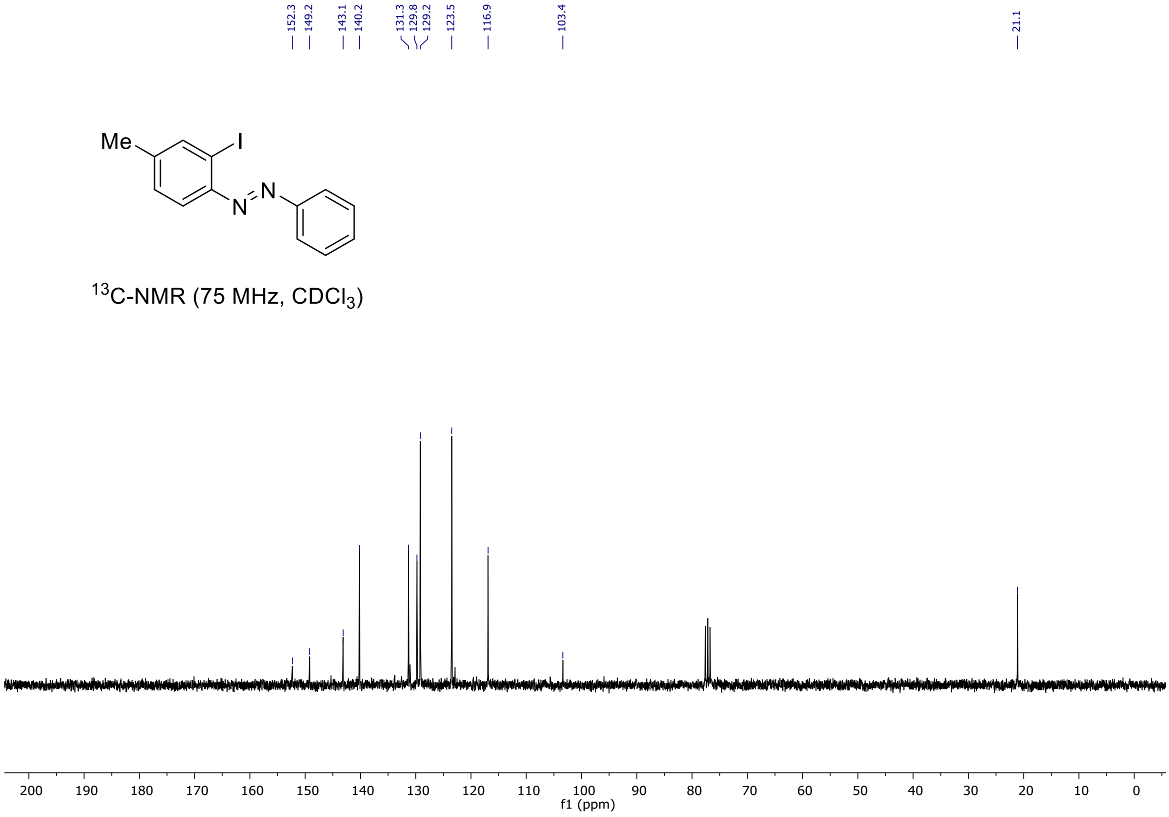

(*E*)-1-(2-Iodo-3-methylphenyl)-2-phenyldiazene

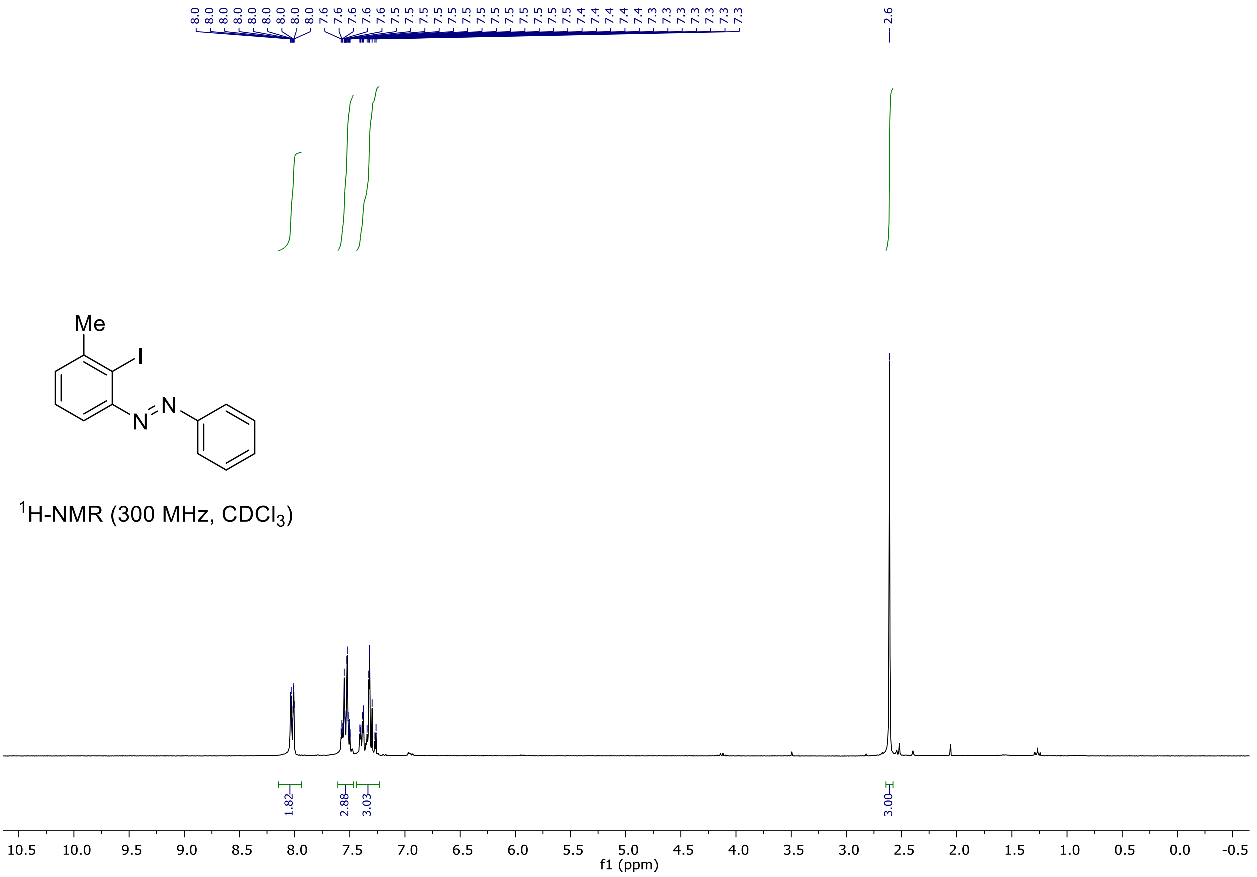

^{1}H-NMR (300 MHz, $CDCl_3$)

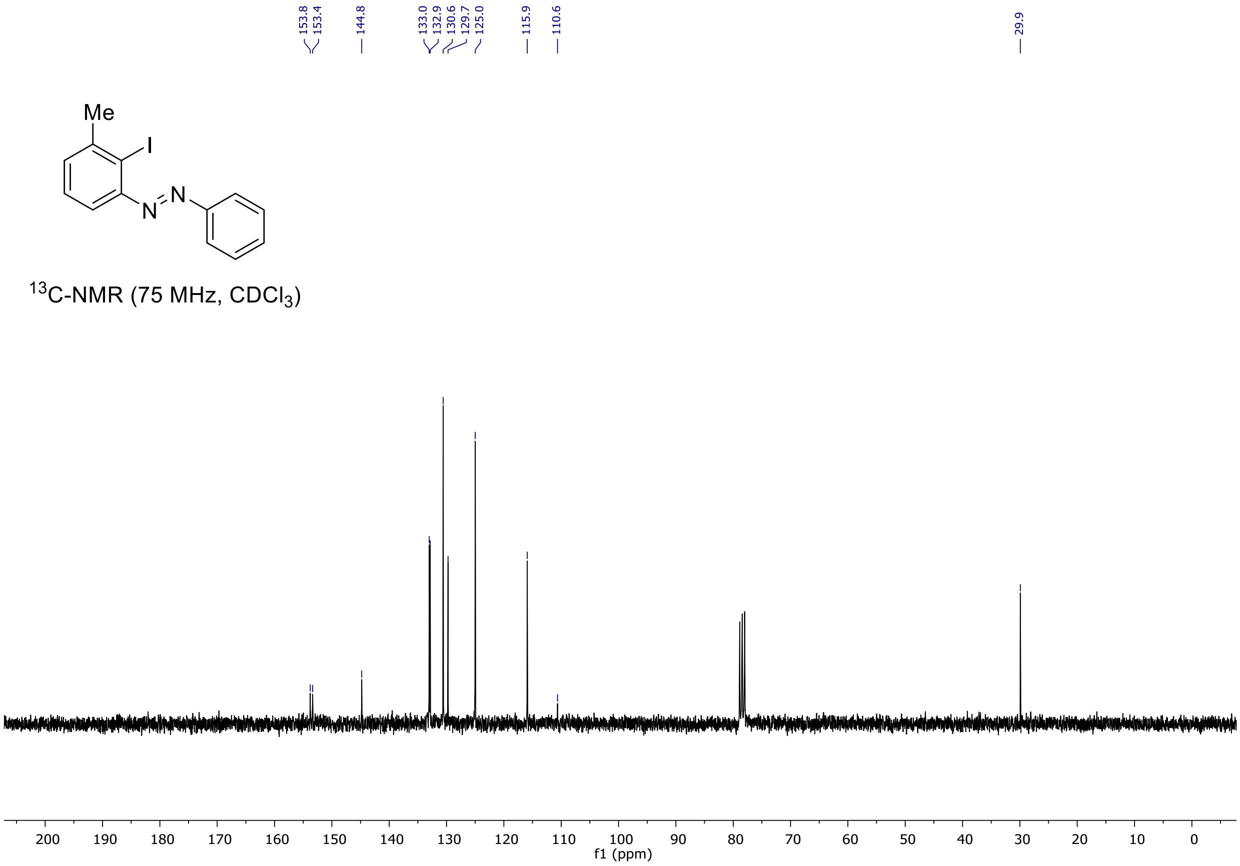

^{13}C-NMR (75 MHz, $CDCl_3$)

(*E*)-1-[2-(Hex-1-yn-1-yl)phenyl]-2-phenyldiazene (1a)

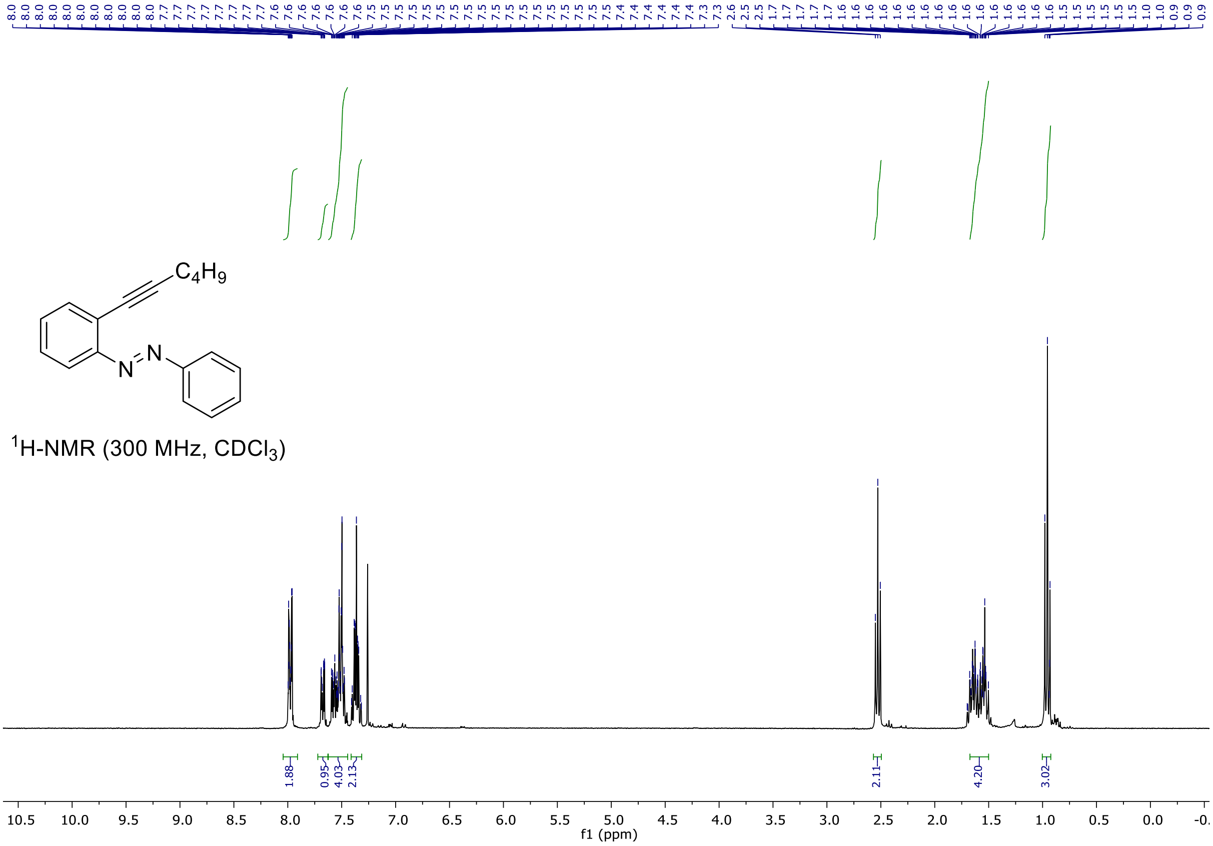

^{1}H-NMR (300 MHz, $CDCl_3$)

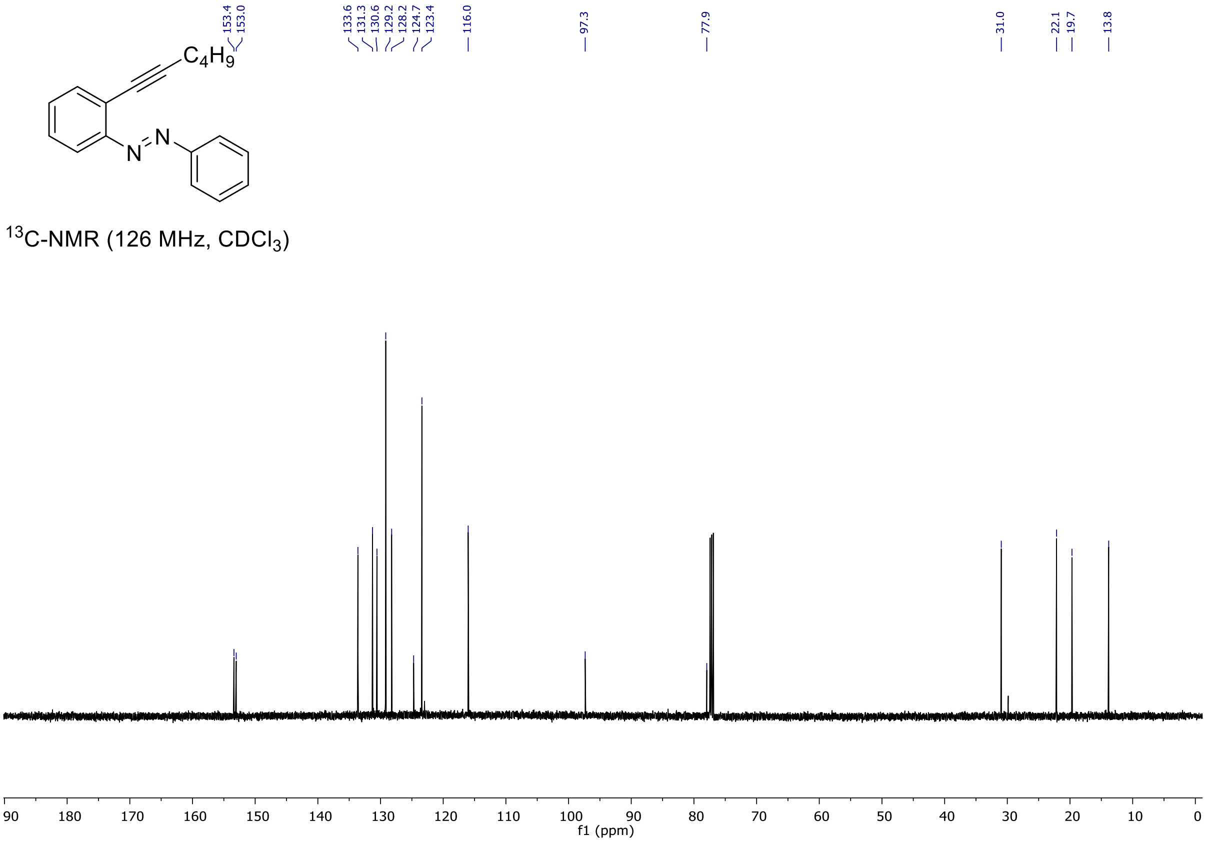

^{13}C-NMR (126 MHz, $CDCl_3$)

(*Z*)-1-[2-(Hex-1-yn-1-yl)phenyl]-2-phenyldiazene (1a’)

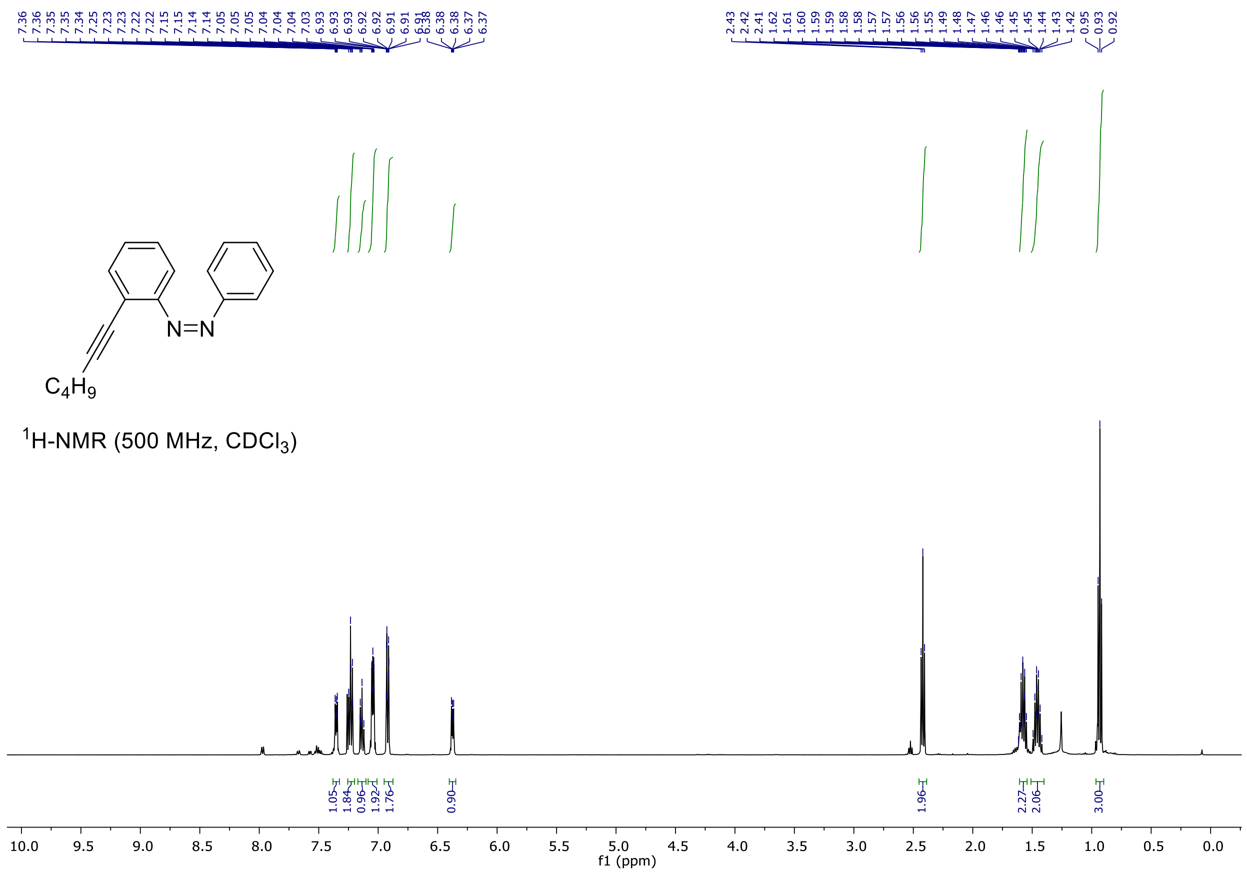

^{1}H-NMR (500 MHz, $CDCl_3$)

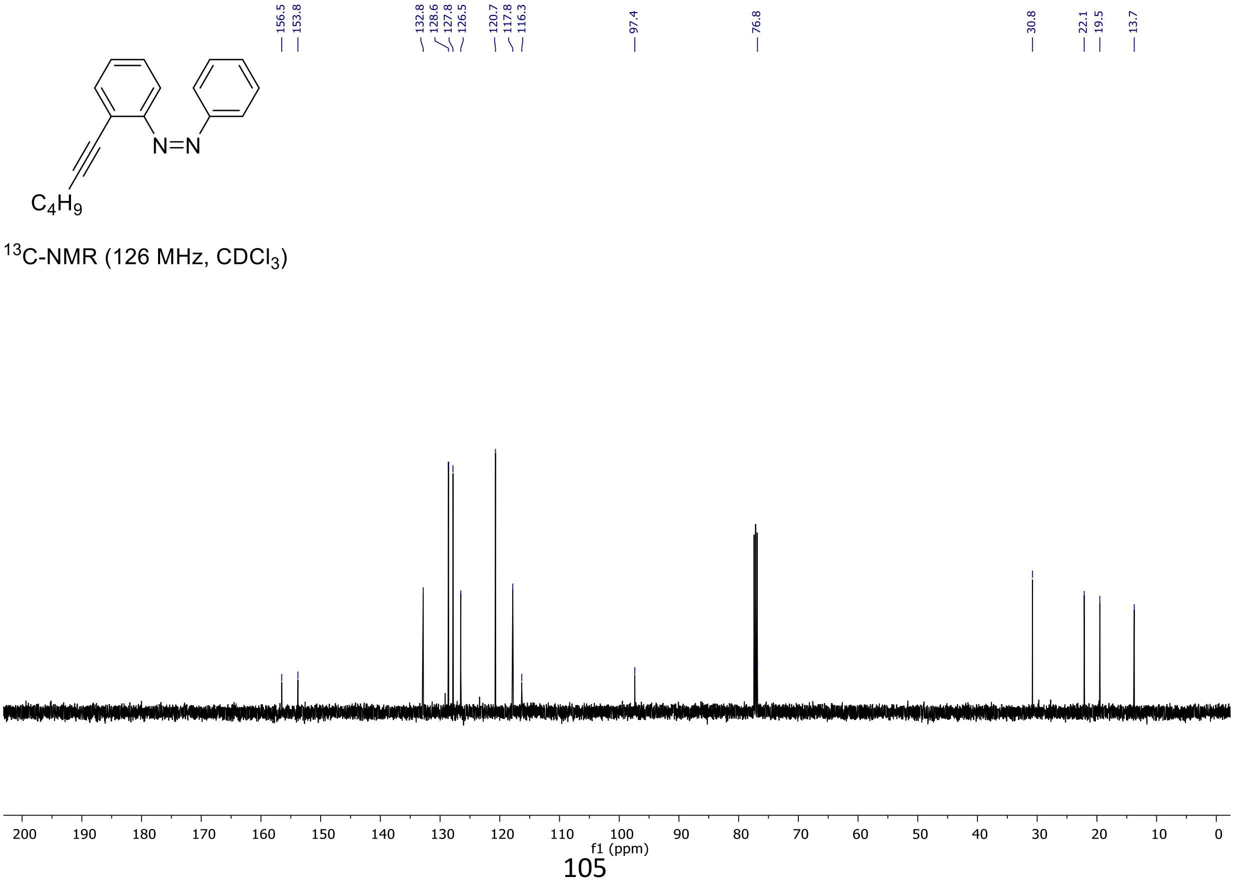

^{13}C-NMR (126 MHz, $CDCl_3$)

(*E*)-1-(4-Bromophenyl)-2-[2-(hex-1-yn-1-yl)phenyl]diazene (1b)

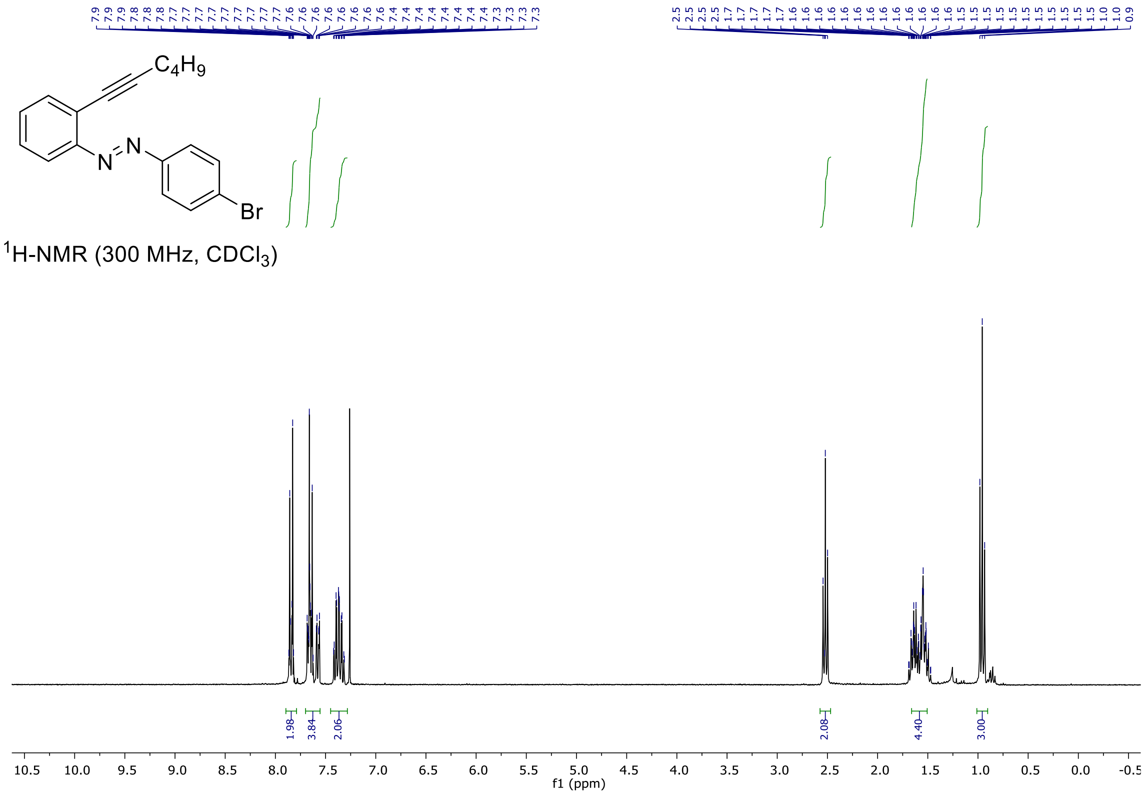

^{1}H-NMR (300 MHz, $CDCl_3$)

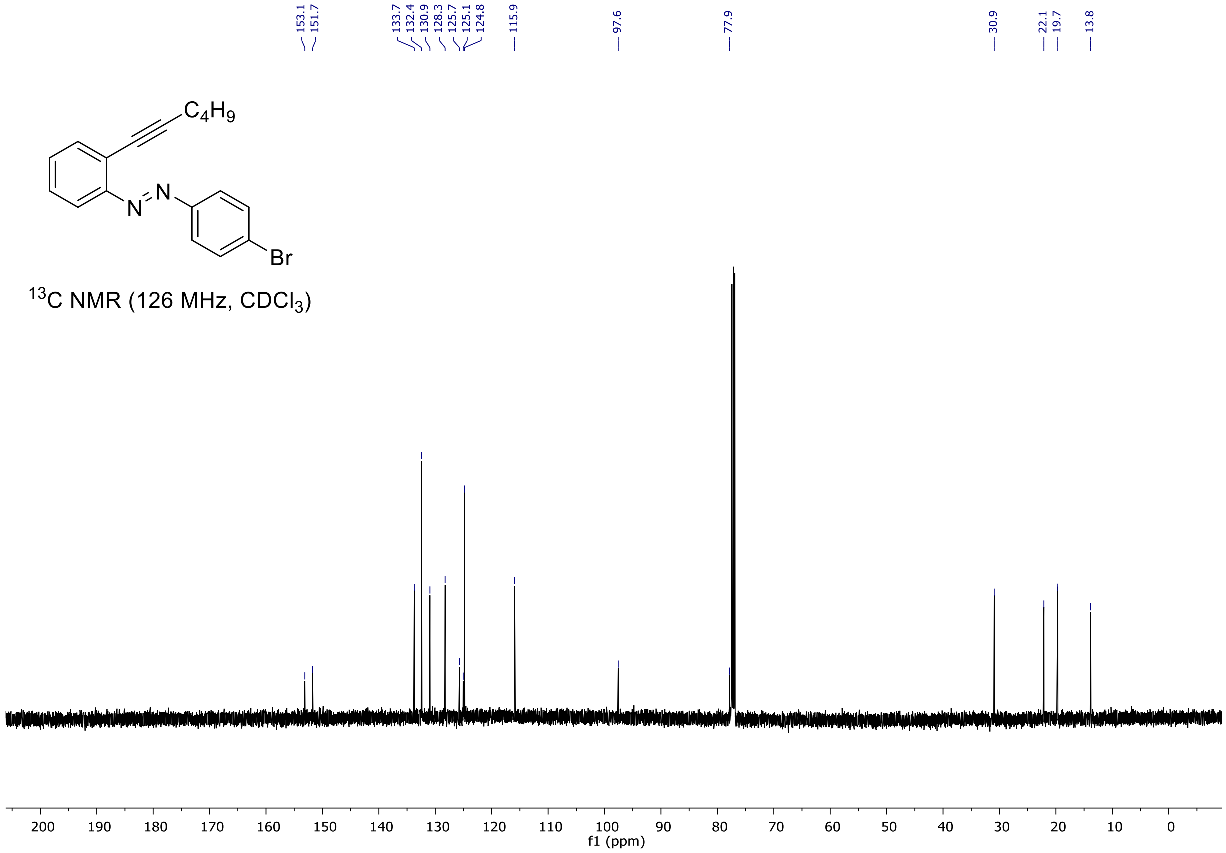

^{13}C NMR (126 MHz, $CDCl_3$)

(*E*)-1-(3-Bromophenyl)-2-[2-(hex-1-yn-1-yl)phenyl]diazene (1c)

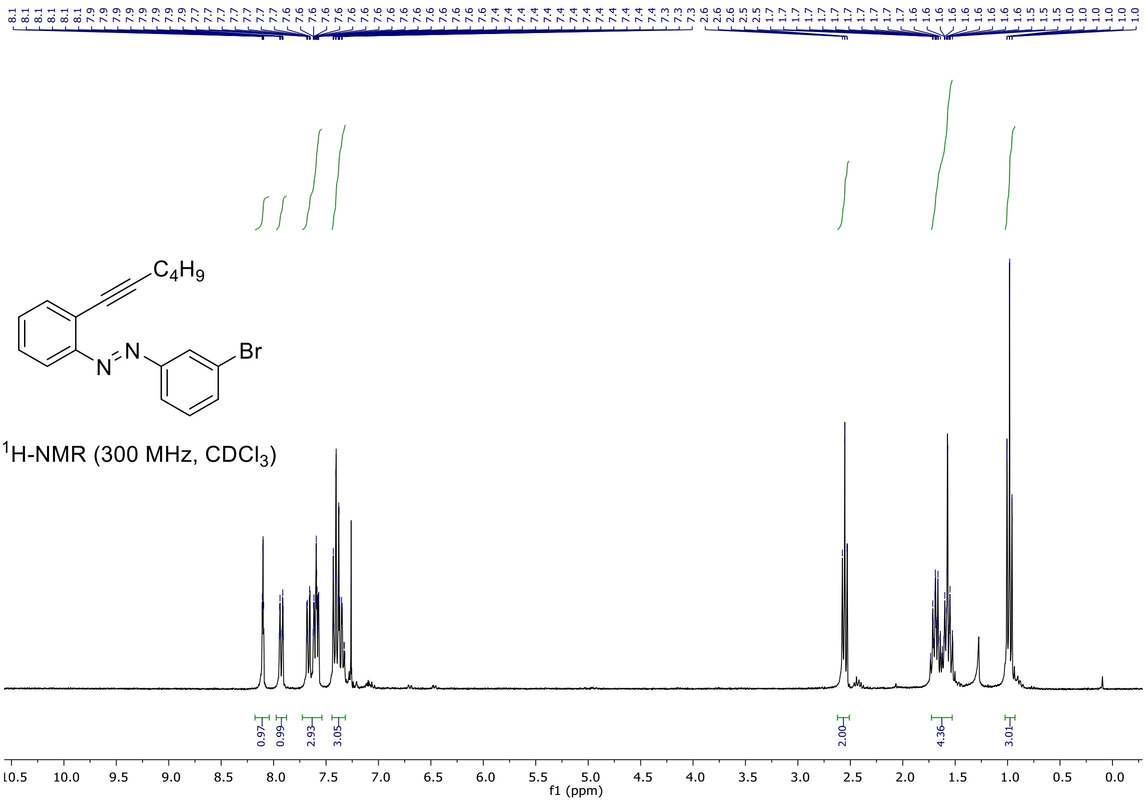

^{1}H-NMR (300 MHz, $CDCl_3$)

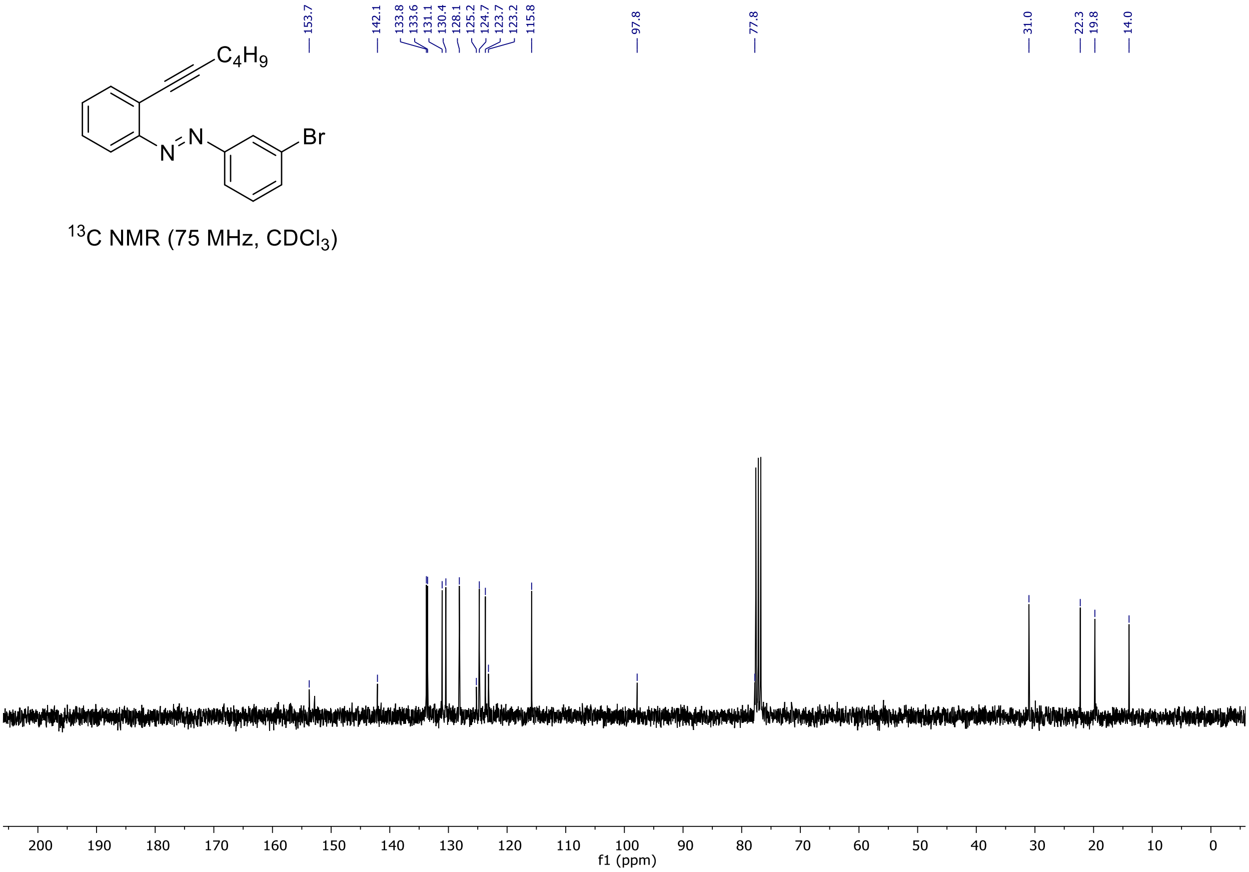

^{13}C NMR (75 MHz, $CDCl_3$)

(*E*)-1-(3-Fluorophenyl)-2-[2-(hex-1-yn-1-yl)phenyl]diazene (1d)

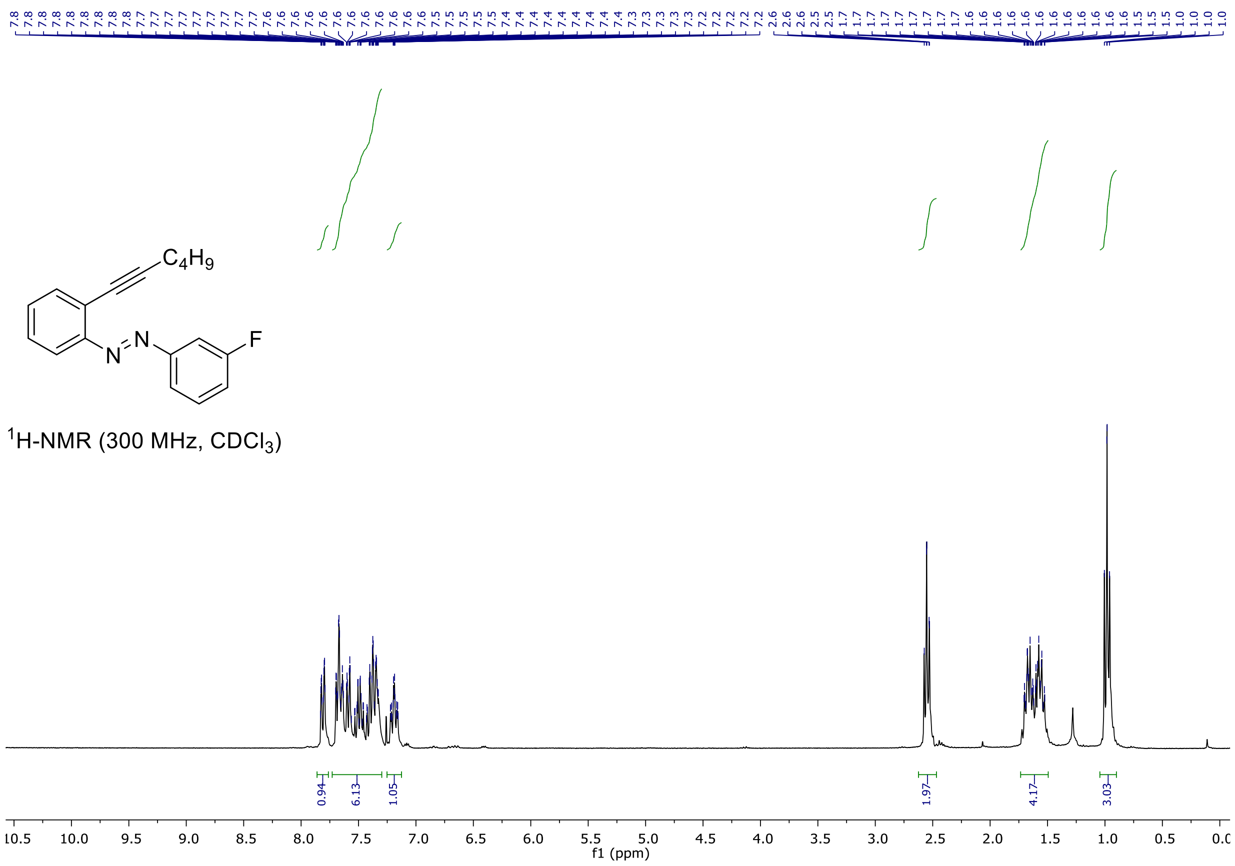

^{1}H-NMR (300 MHz, $CDCl_3$)

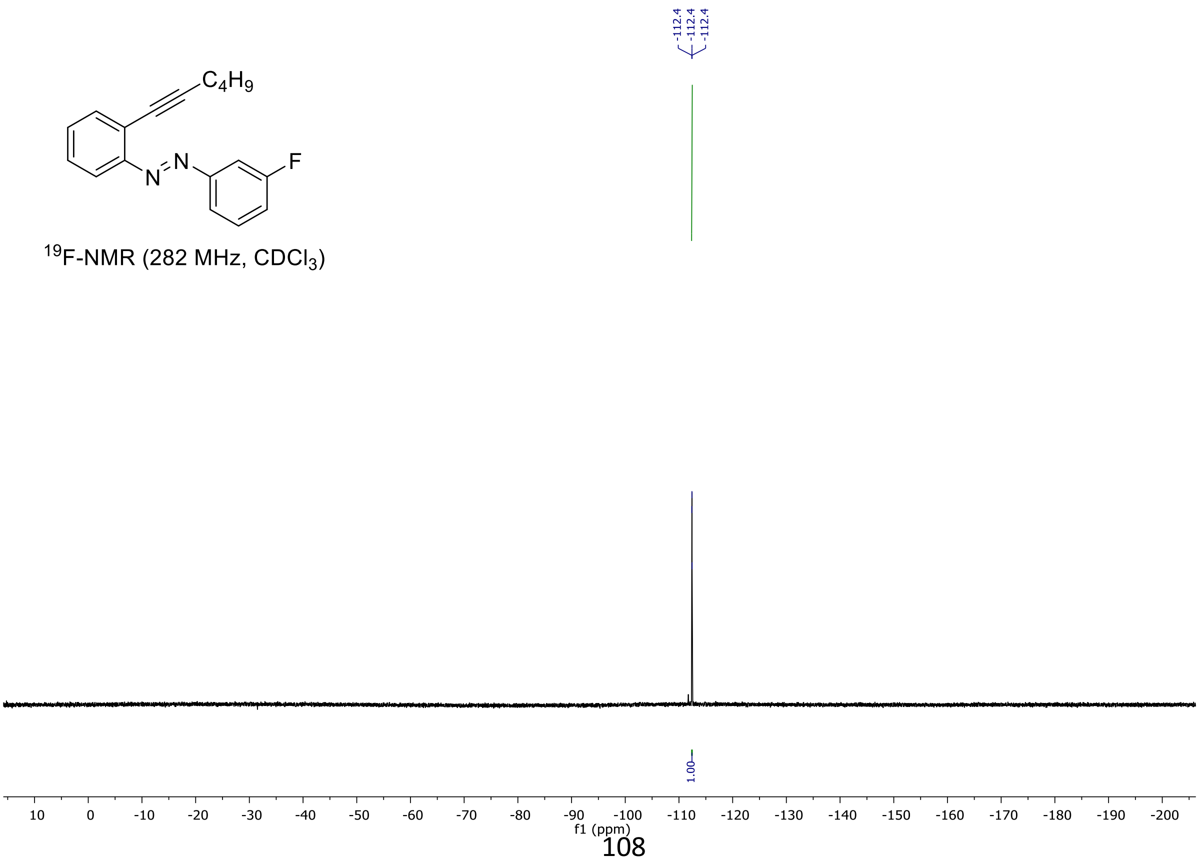

^{19}F-NMR (282 MHz, $CDCl_3$)

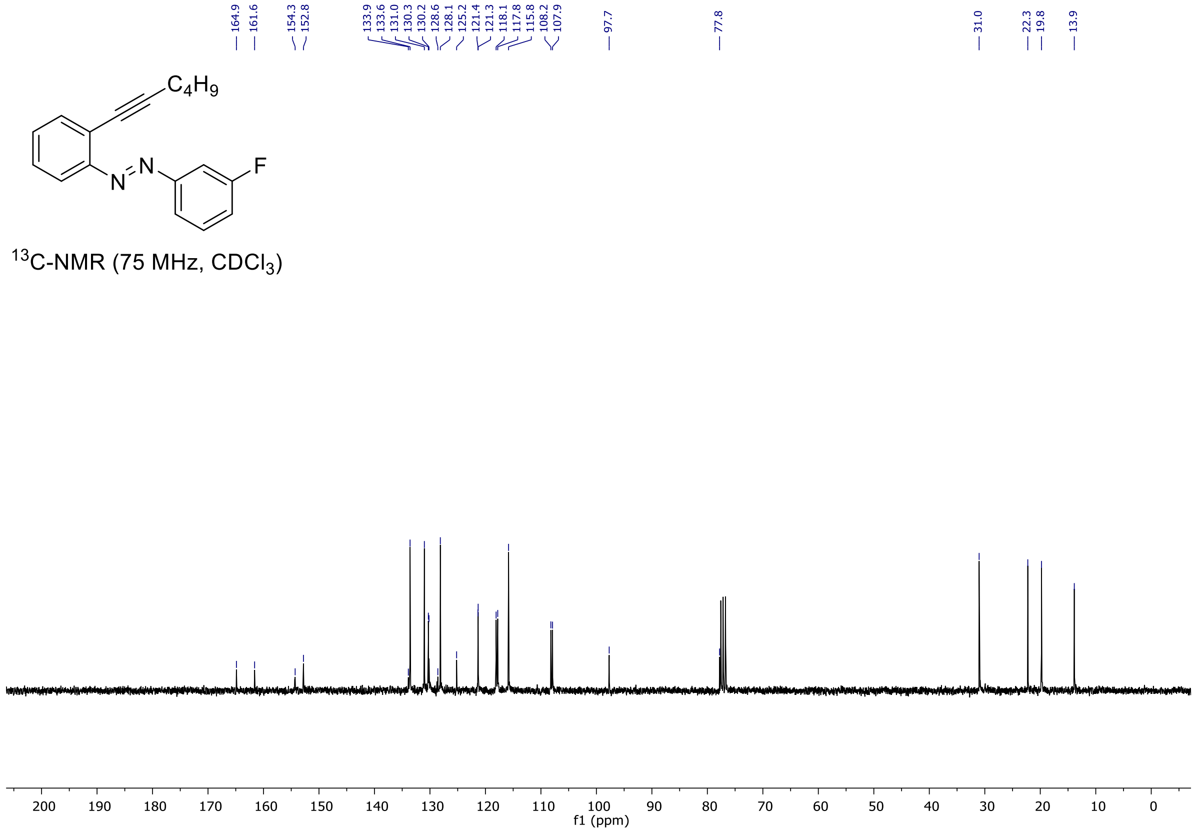

^{13}C-NMR (75 MHz, $CDCl_3$)

(*E*)-1-(2-Fluorophenyl)-2-[2-(hex-1-yn-1-yl)phenyl]diazene (1e)

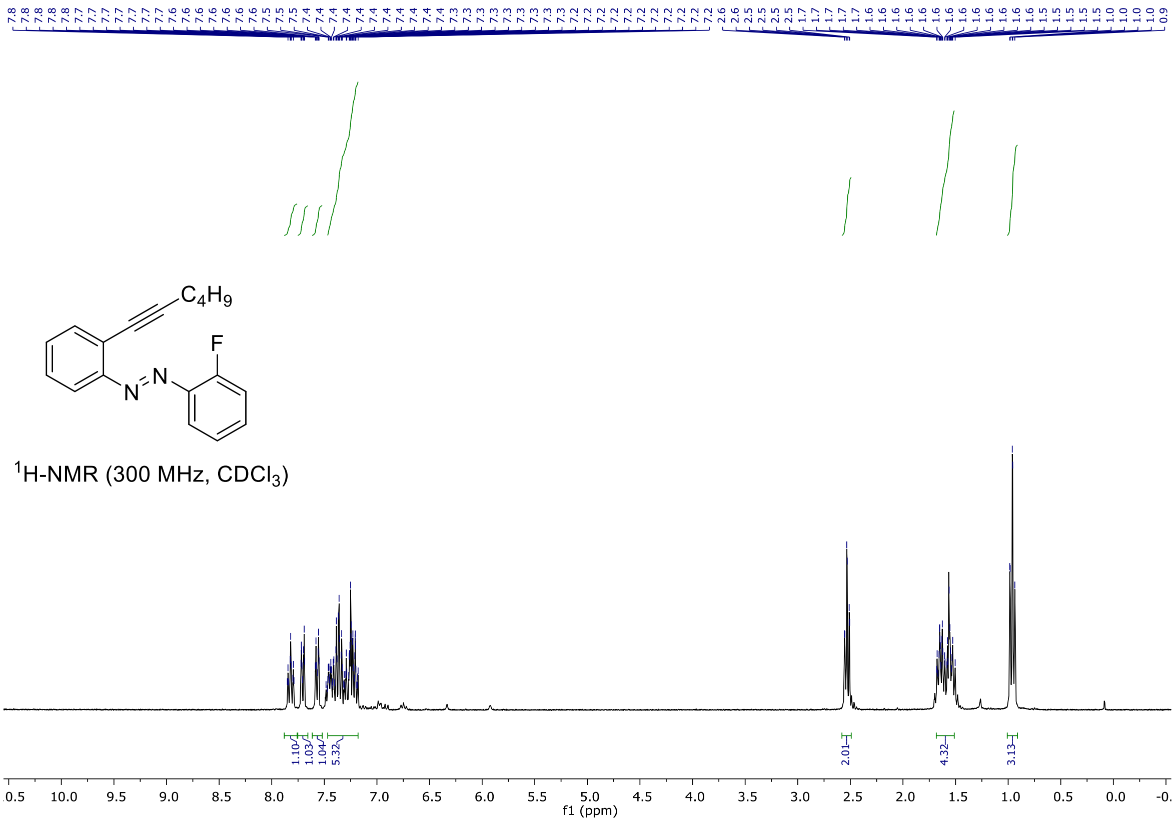

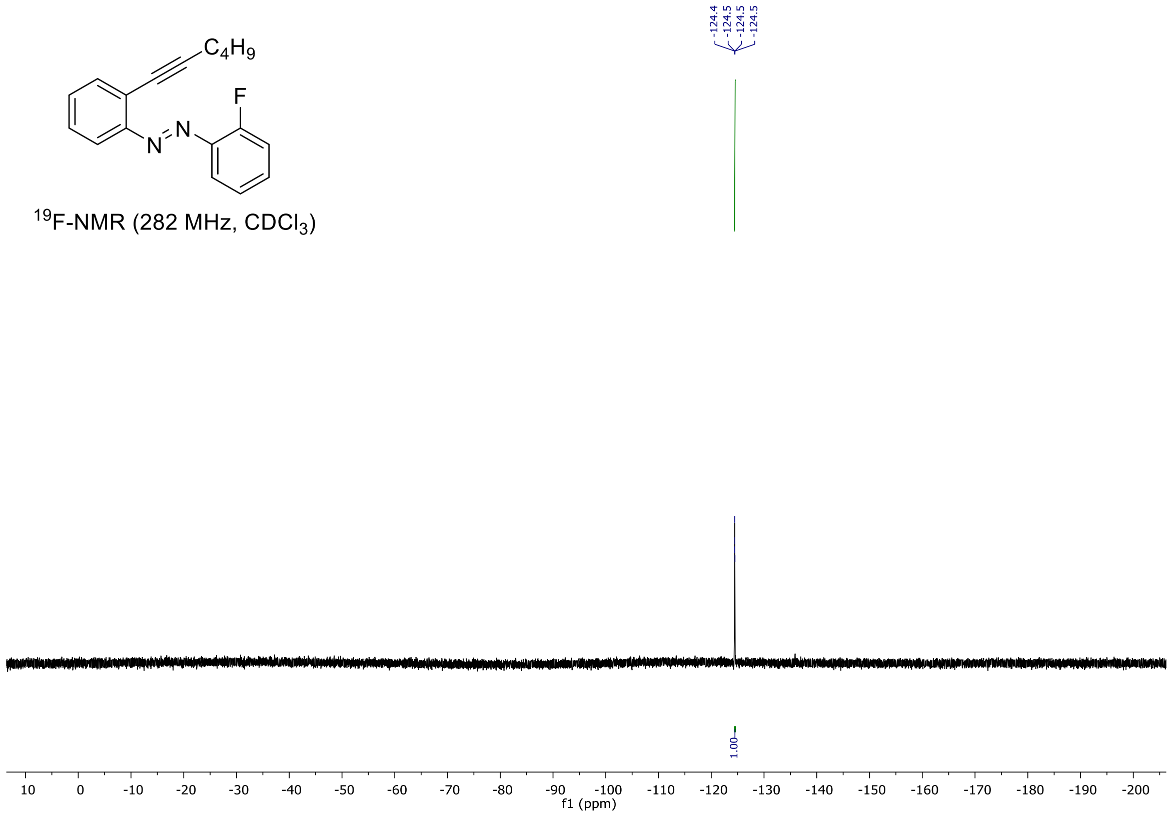

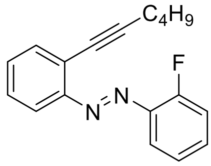

^{13}C-NMR (75 MHz, $CDCl_3$)

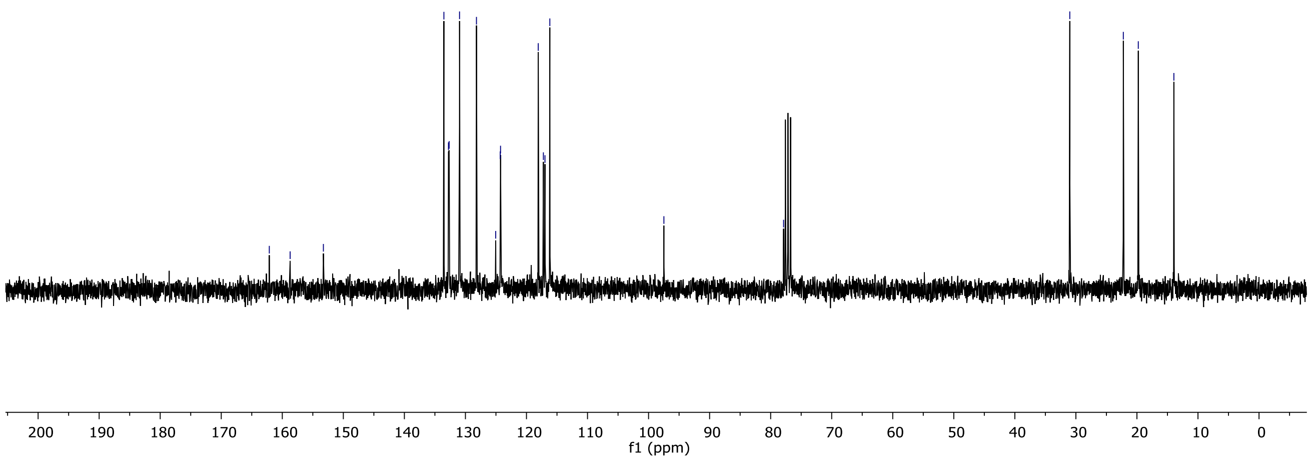

(*E*)-4-[(2-(Hex-1-yn-1-yl)phenyl)diazenyl]benzonitrile (1f)

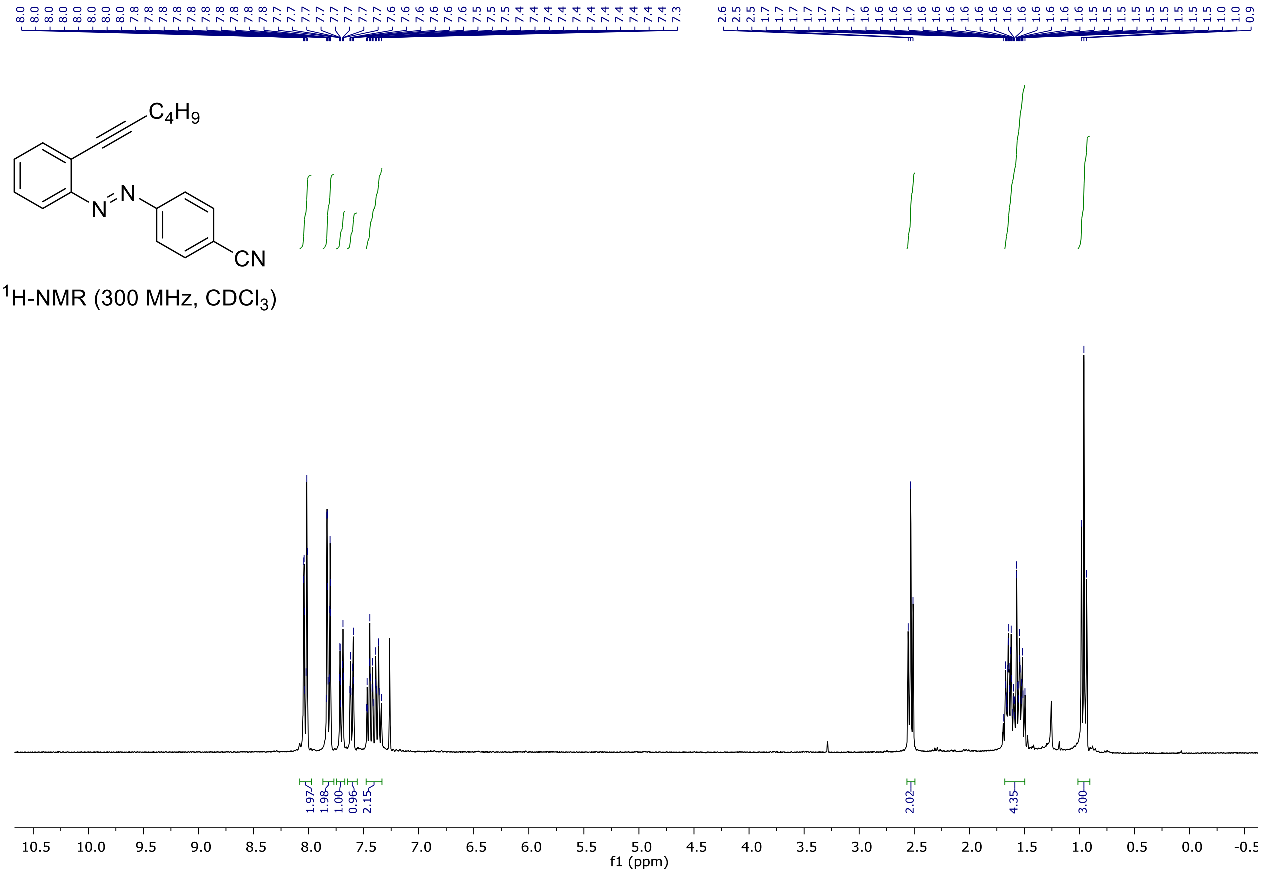

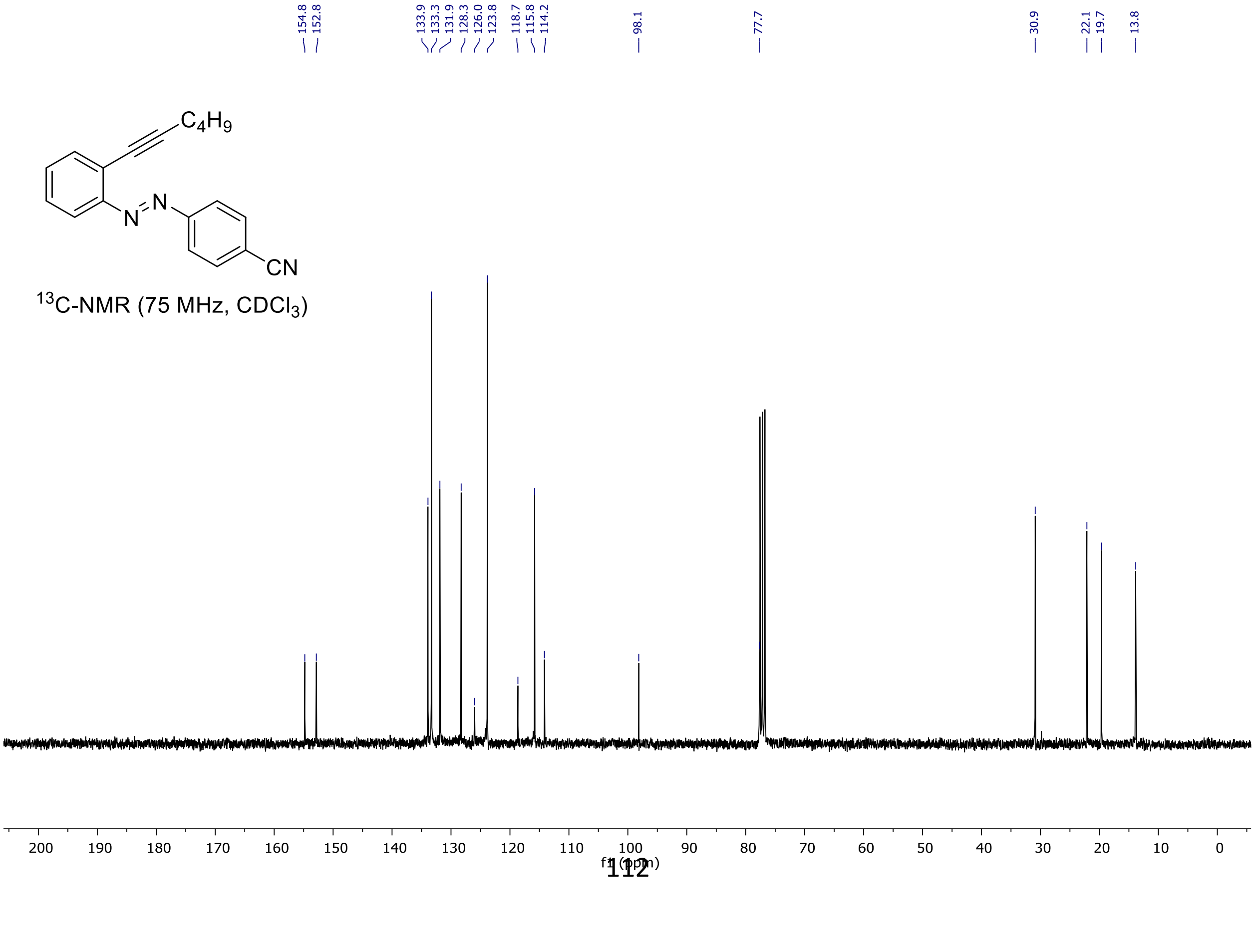

(*E*)-1-[2-(Hex-1-yn-1-yl)phenyl]-2-(3-nitrophenyl)diazene (1g)

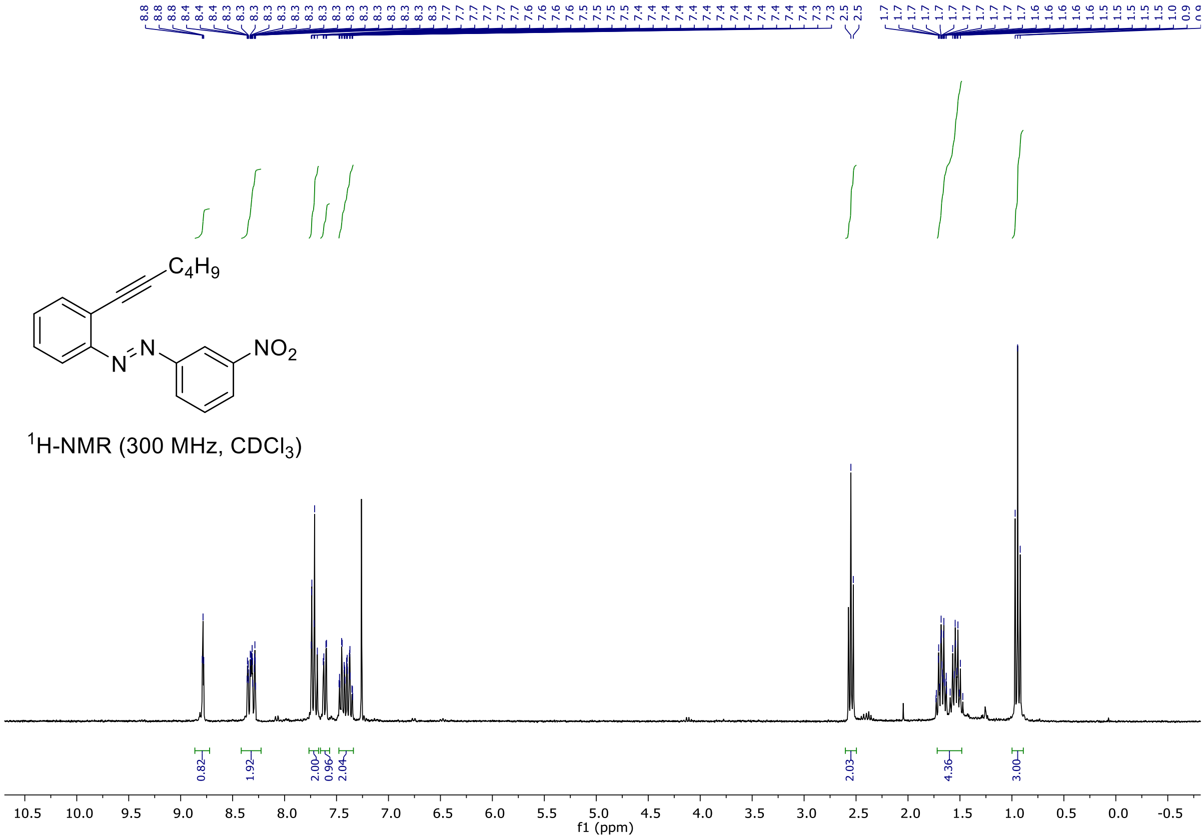

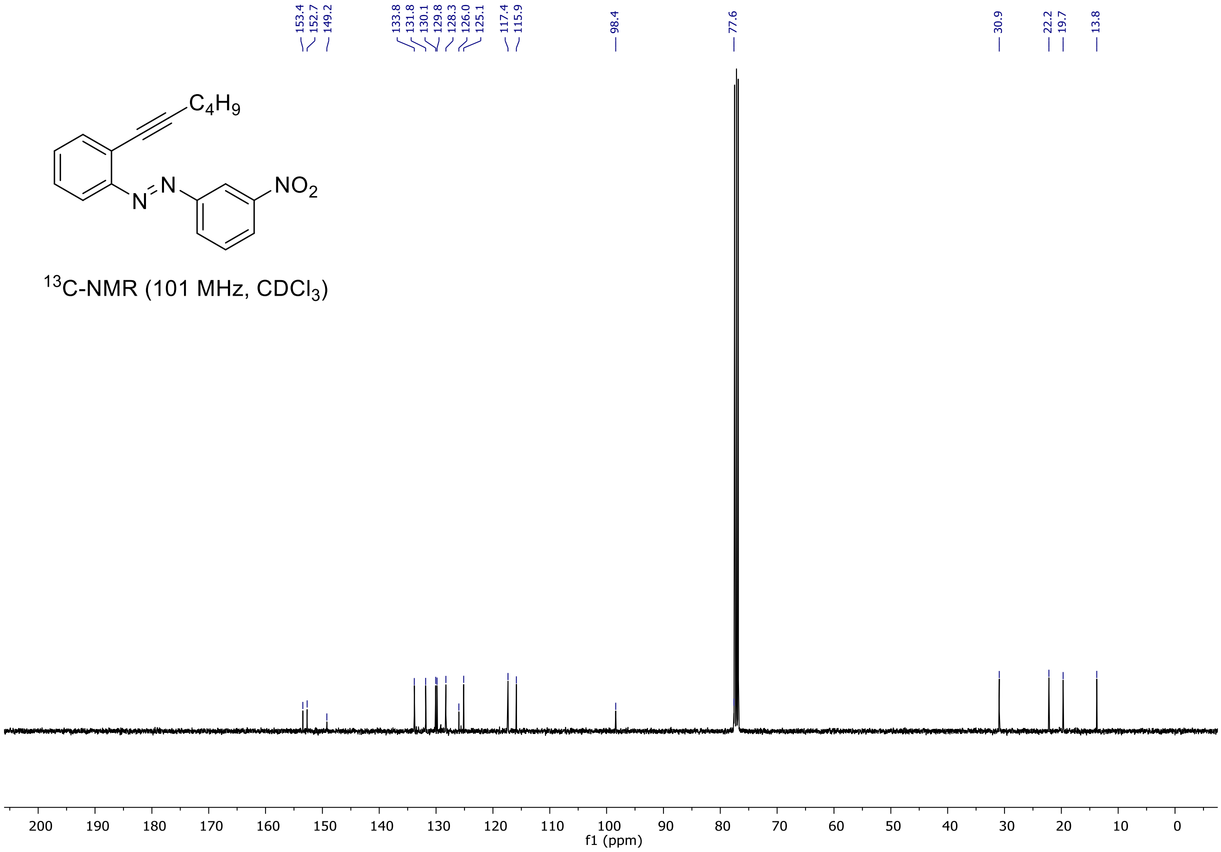

(*E*)-1-[4-((2-(Hex-1-yn-1-yl)phenyl)diazenyl)phenyl]ethan-1-one (1h)

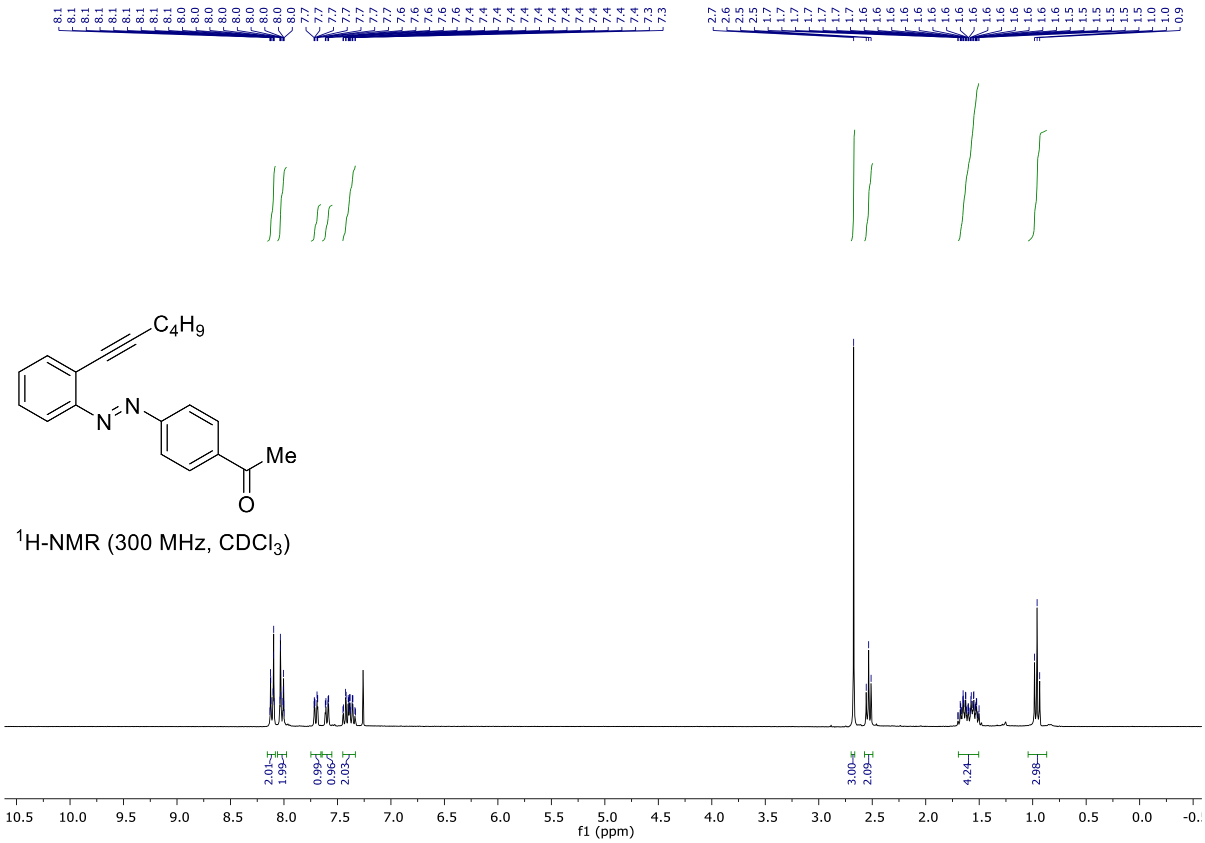

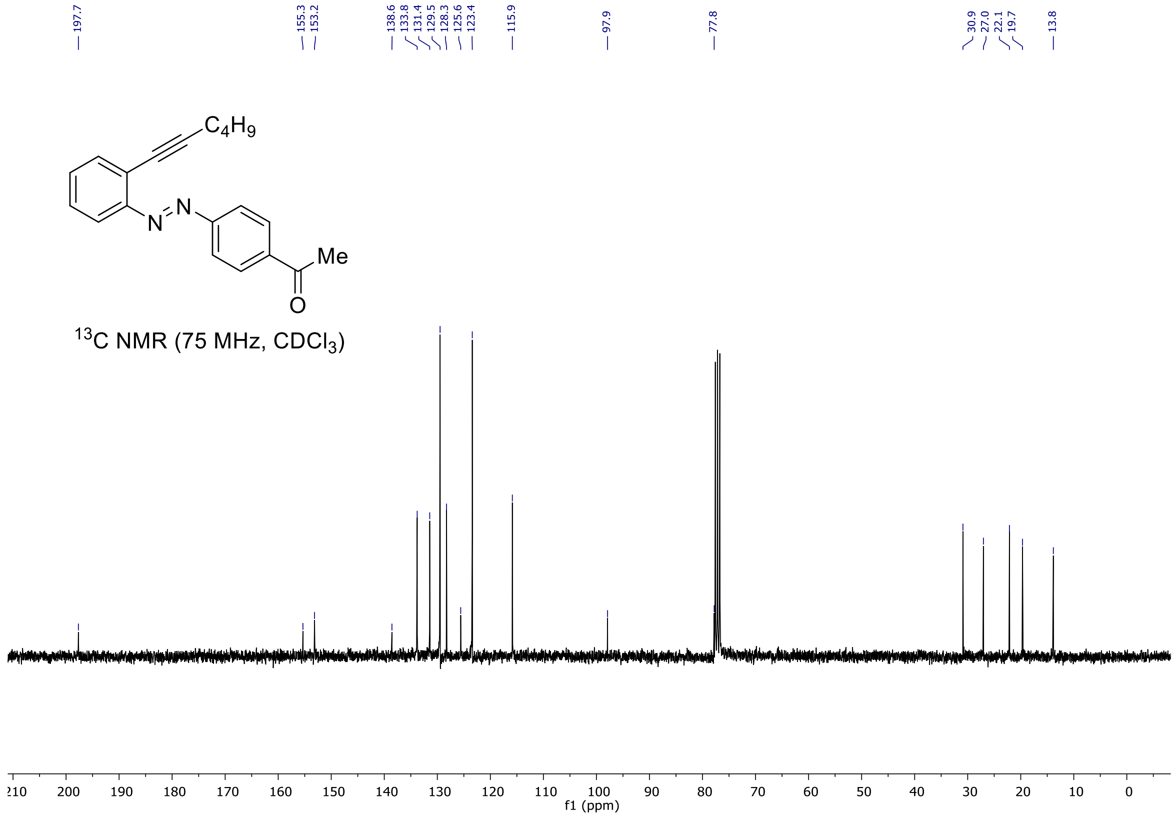

Methyl (*E*)-4-[(2-(hex-1-yn-1-yl)phenyl)diazenyl]benzoate (1i)

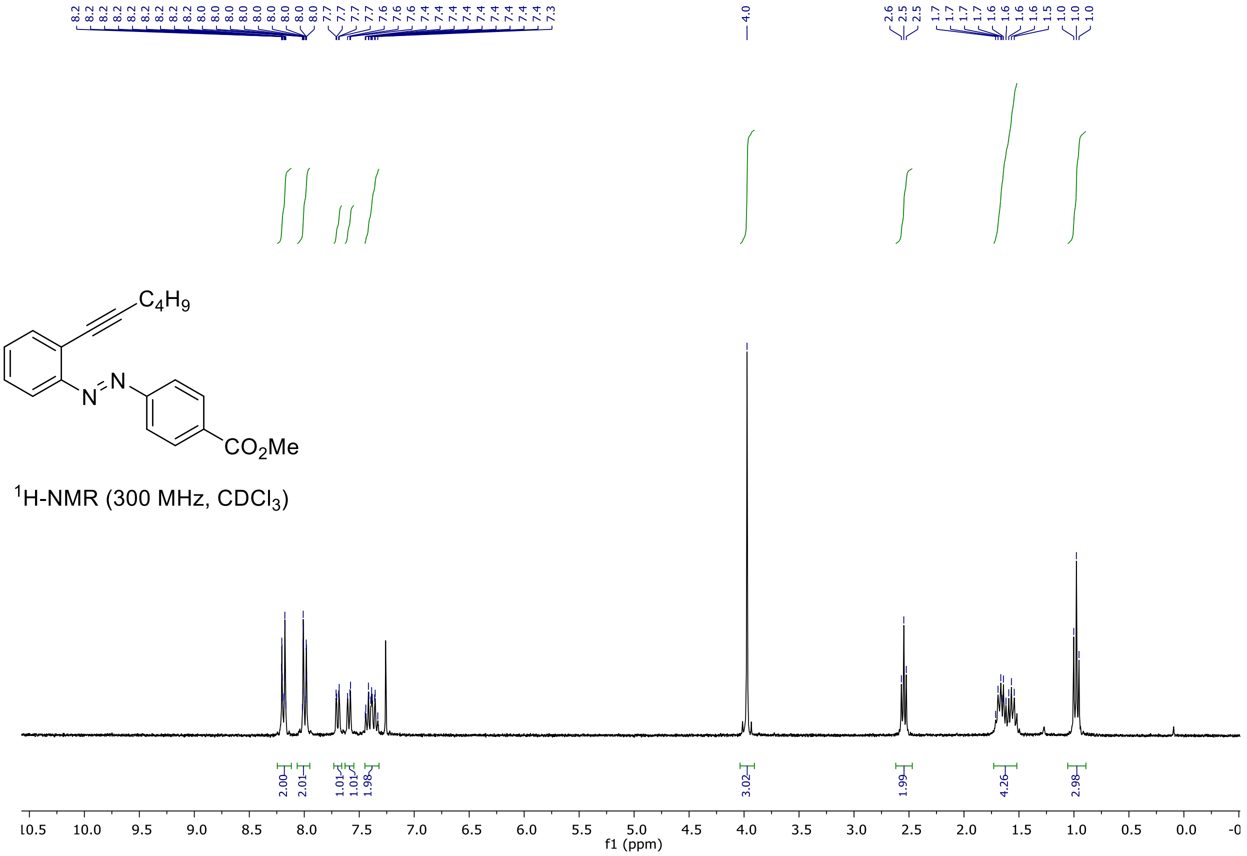

^{1}H-NMR (300 MHz, $CDCl_3$)

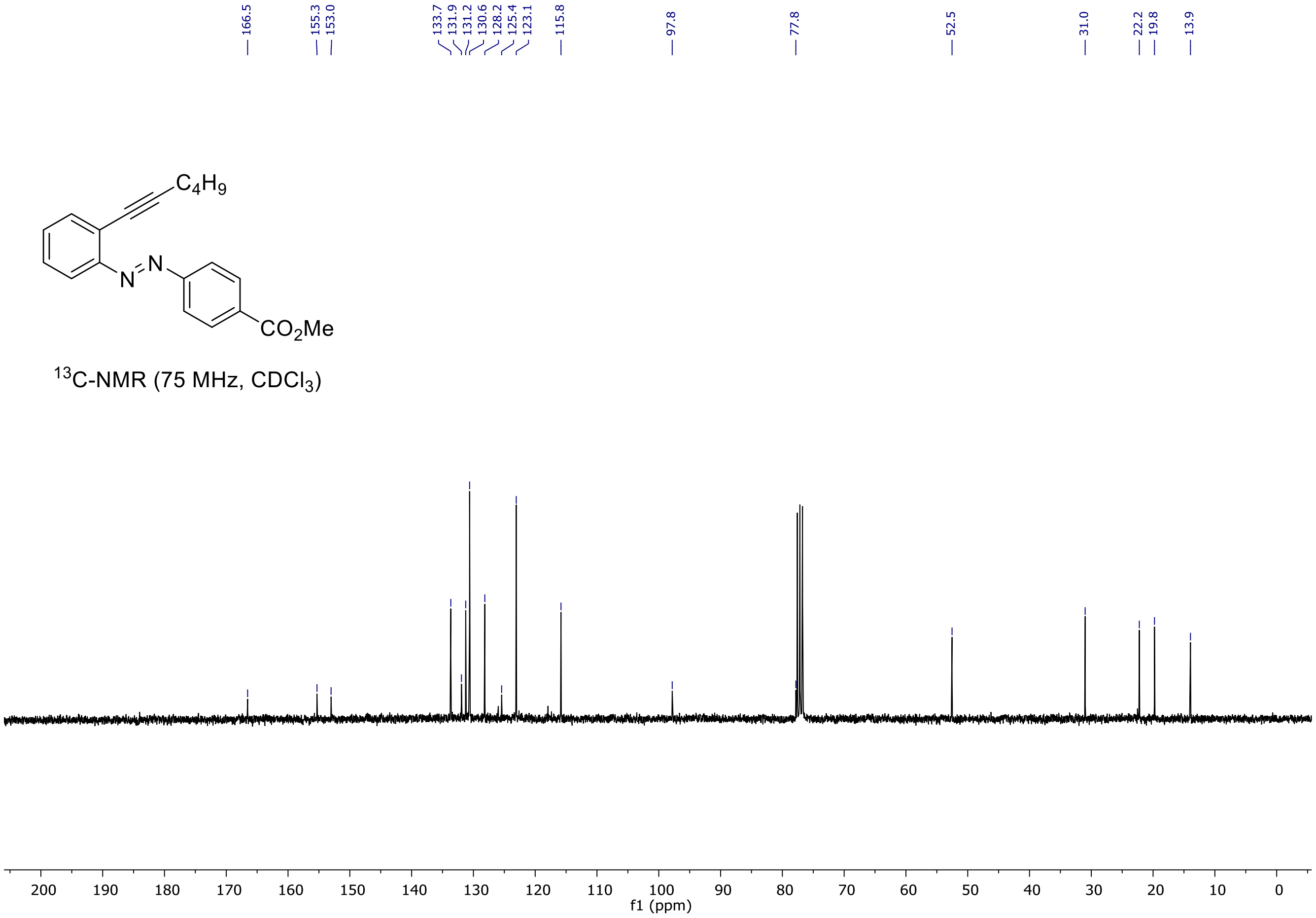

^{13}C-NMR (75 MHz, $CDCl_3$)

(*E*)-1-(2-Bromo-4-methylphenyl)-2-[2-(hex-1-yn-1-yl)phenyl]diazene (1j)

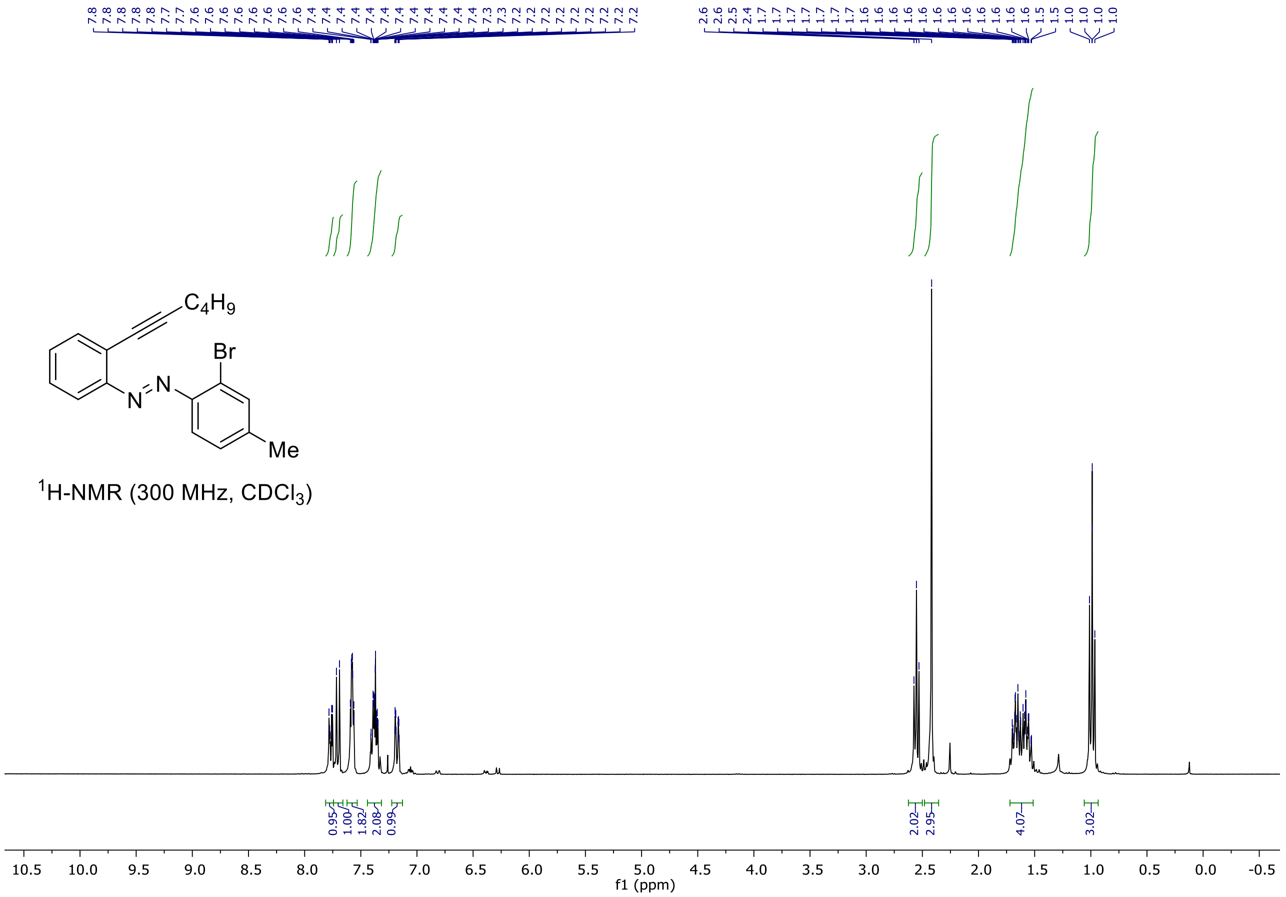

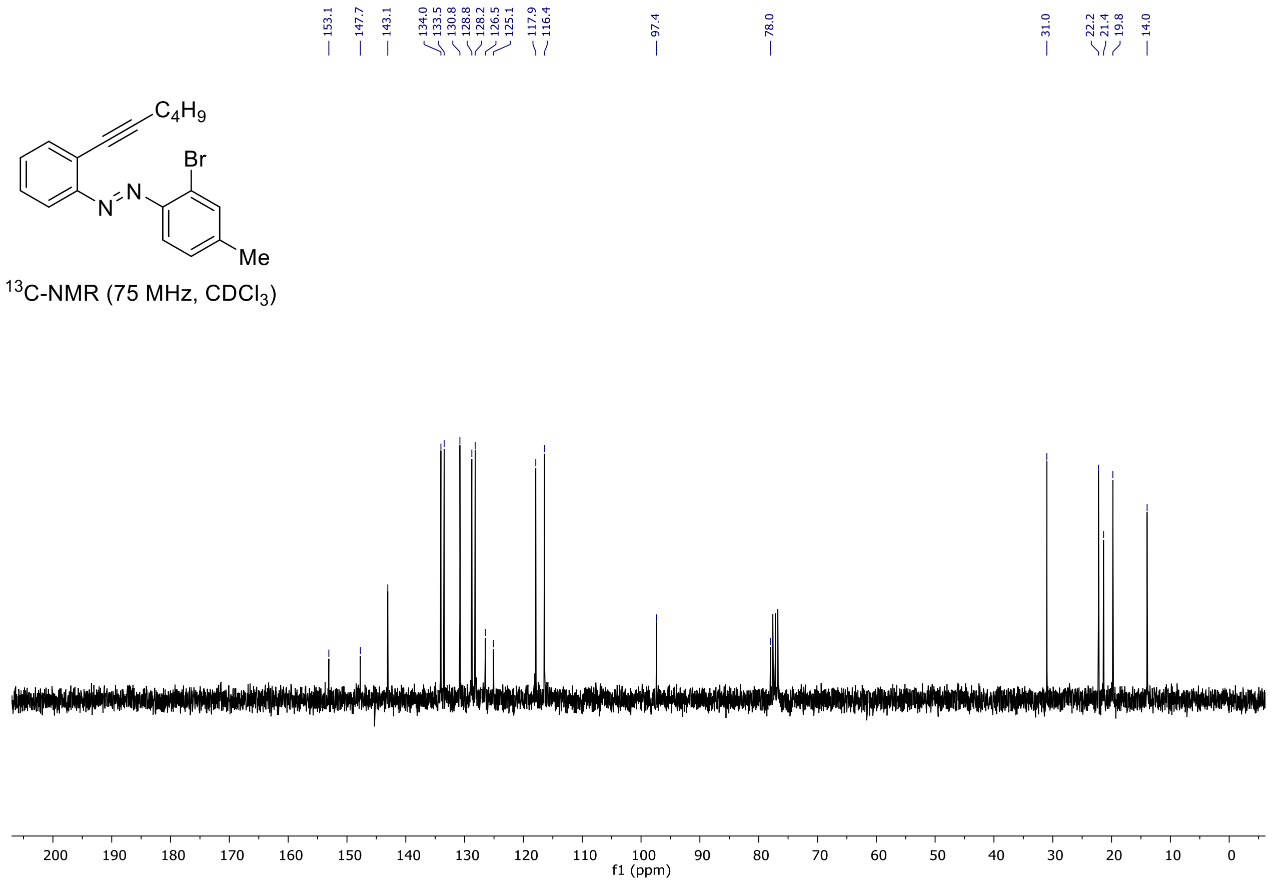

Dimethyl (*E*)-5-[(2-(hex-1-yn-1-yl)phenyl)diazenyl]isophthalate (1k)

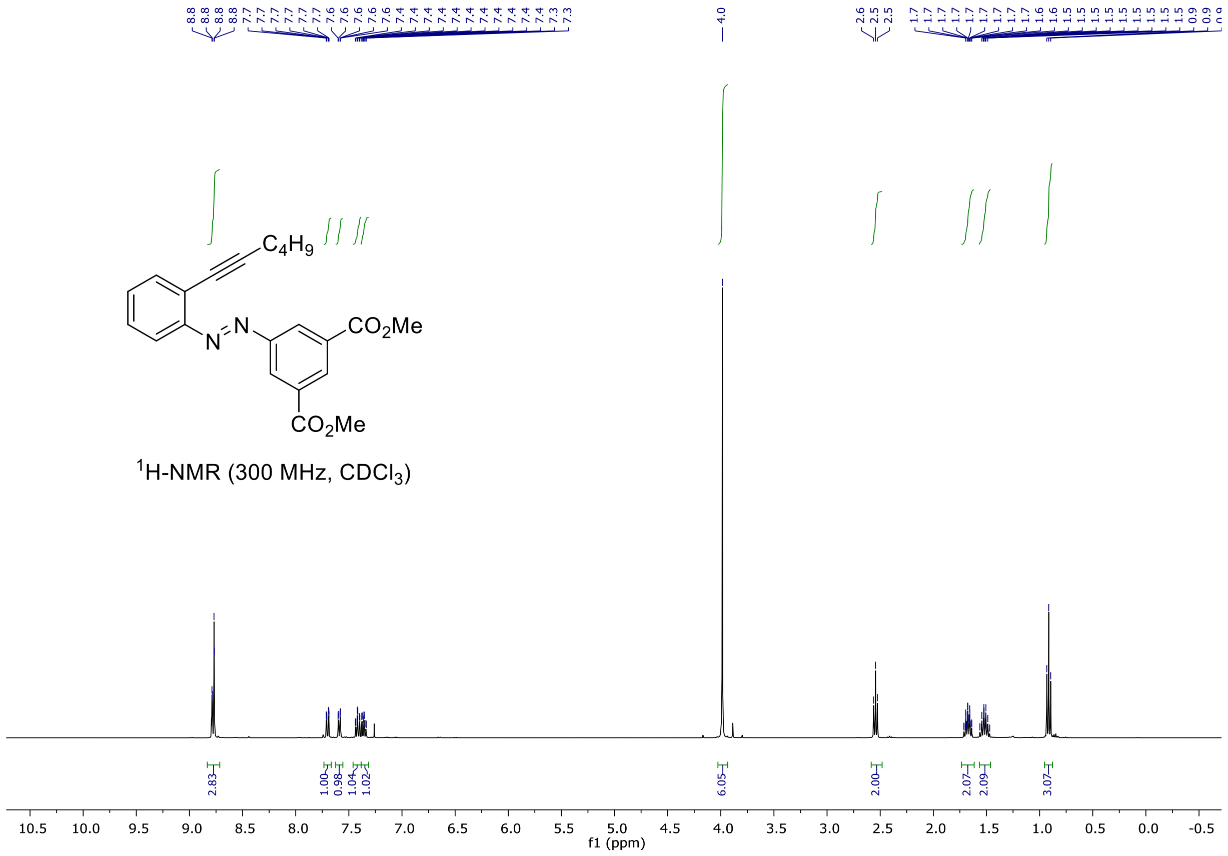

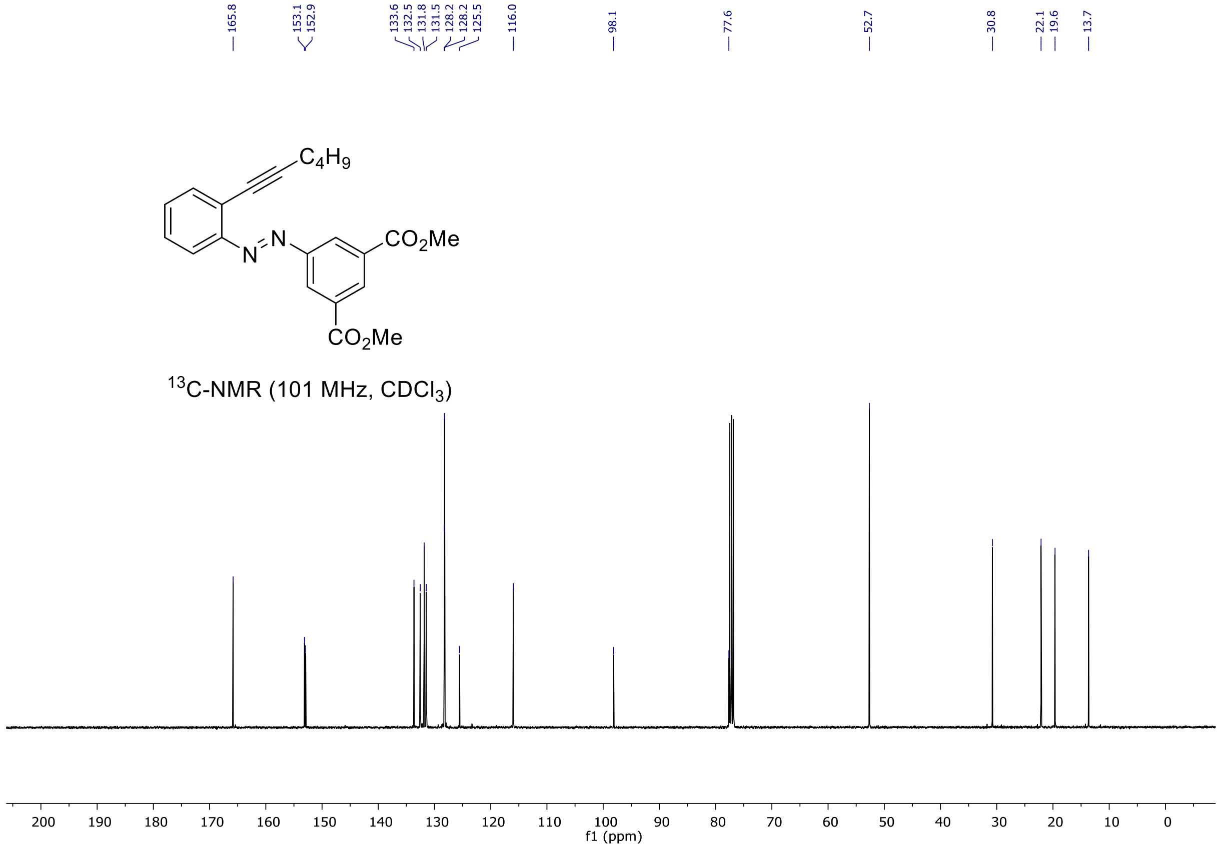

(*E*)-1-[2-(Hex-1-yn-1-yl)phenyl]-2-(perfluorophenyl)diazene (1l)

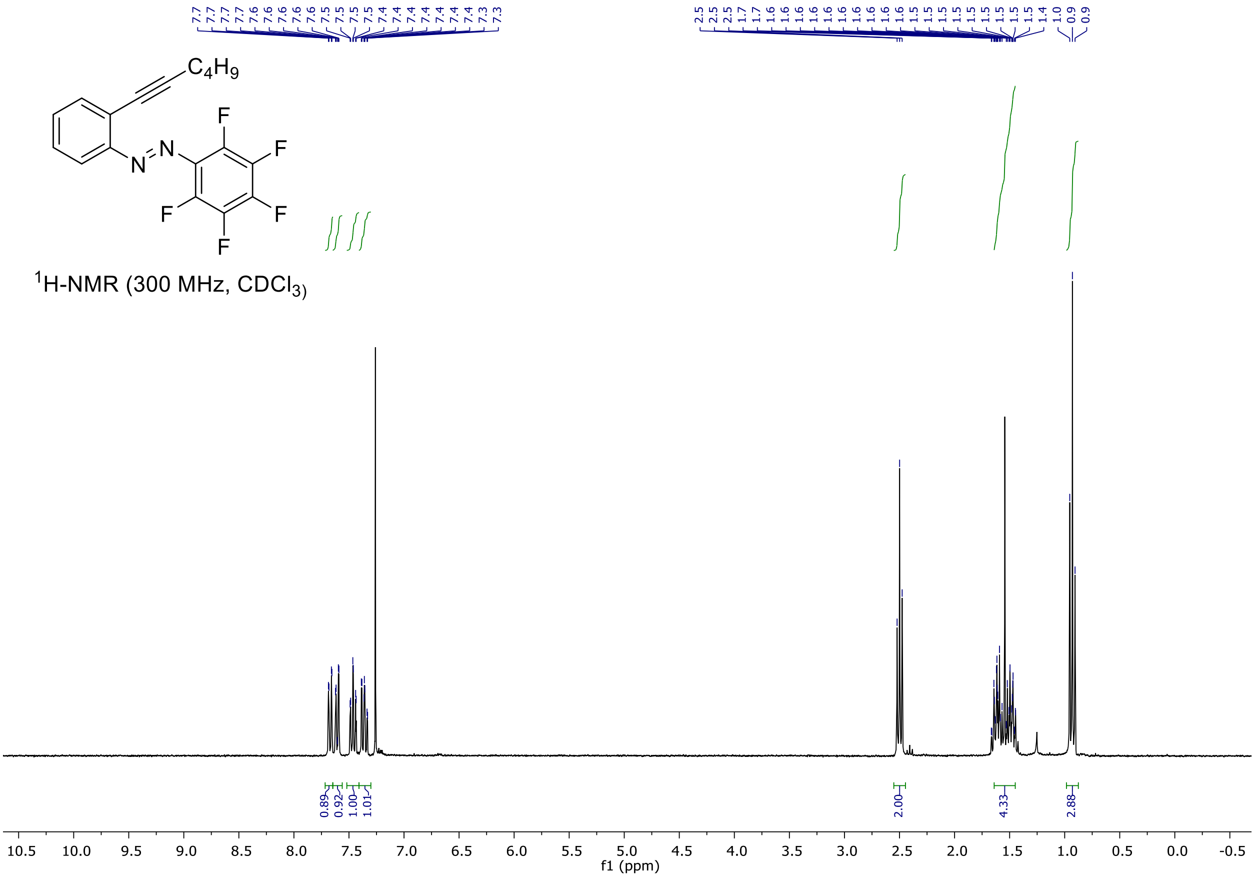

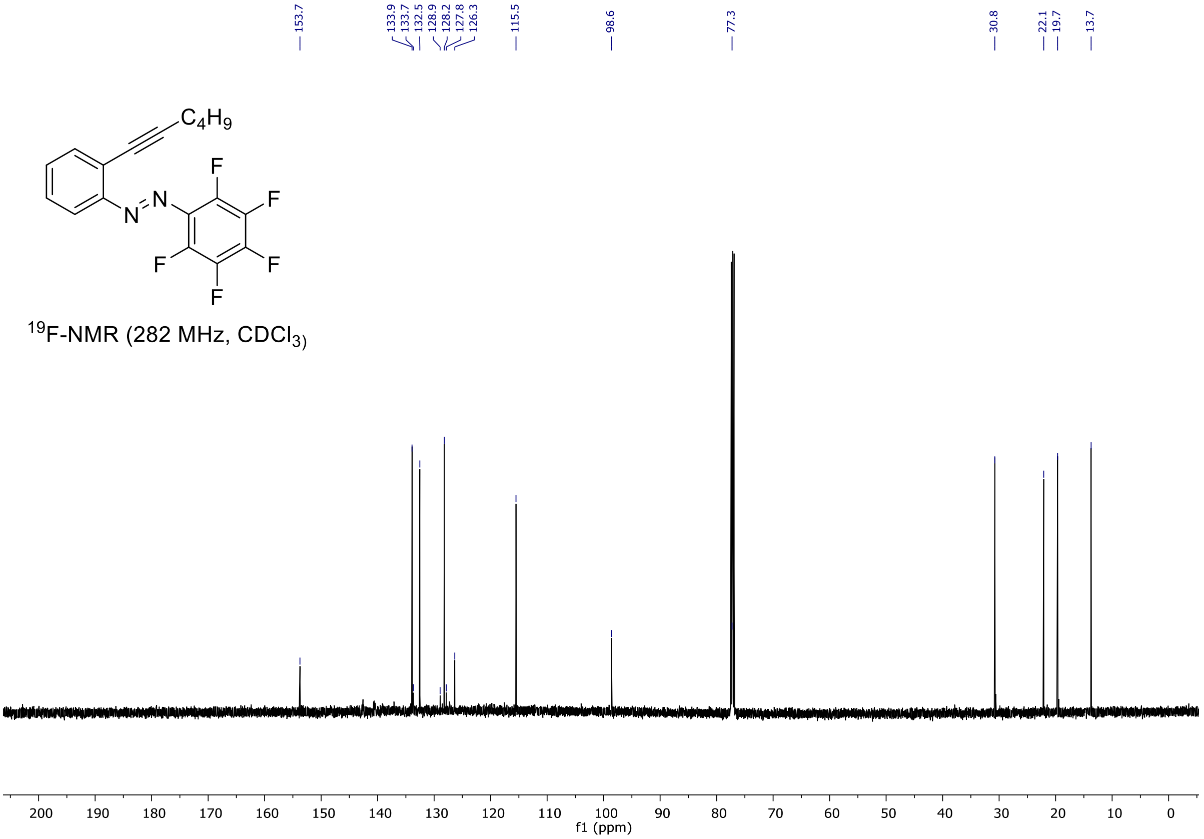

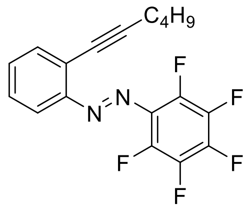

^{13}C-NMR (126 MHz, $CDCl_3$)

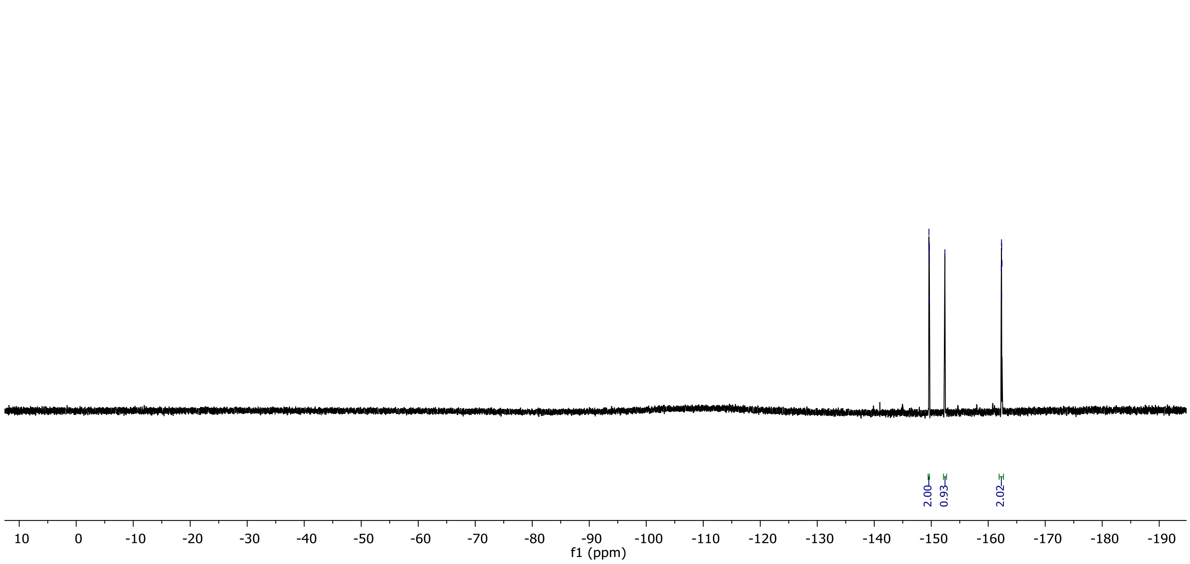

(*E*)-1-[2-(Hex-1-yn-1-yl)phenyl]-2-(*p*-tolyl)diazene (1m)

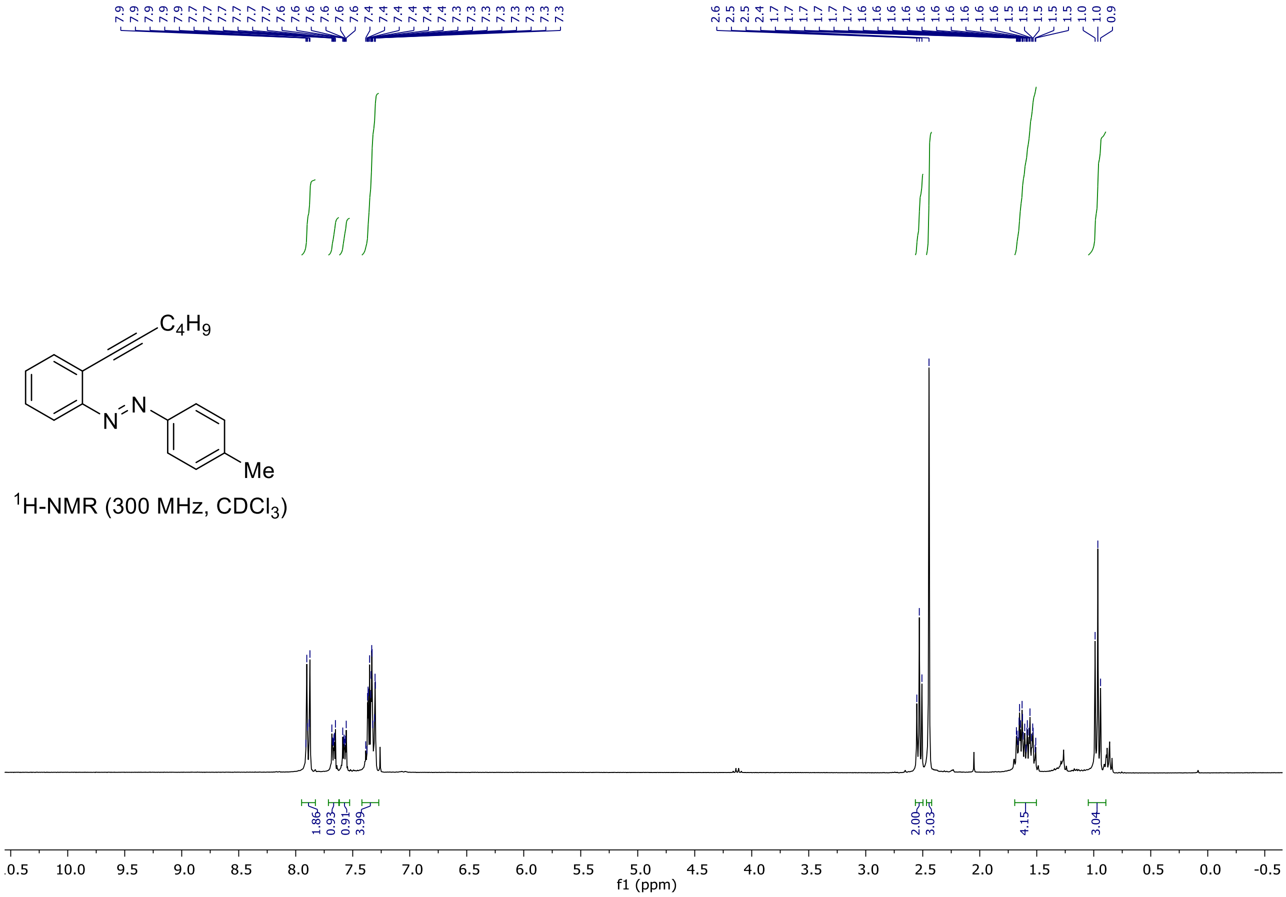

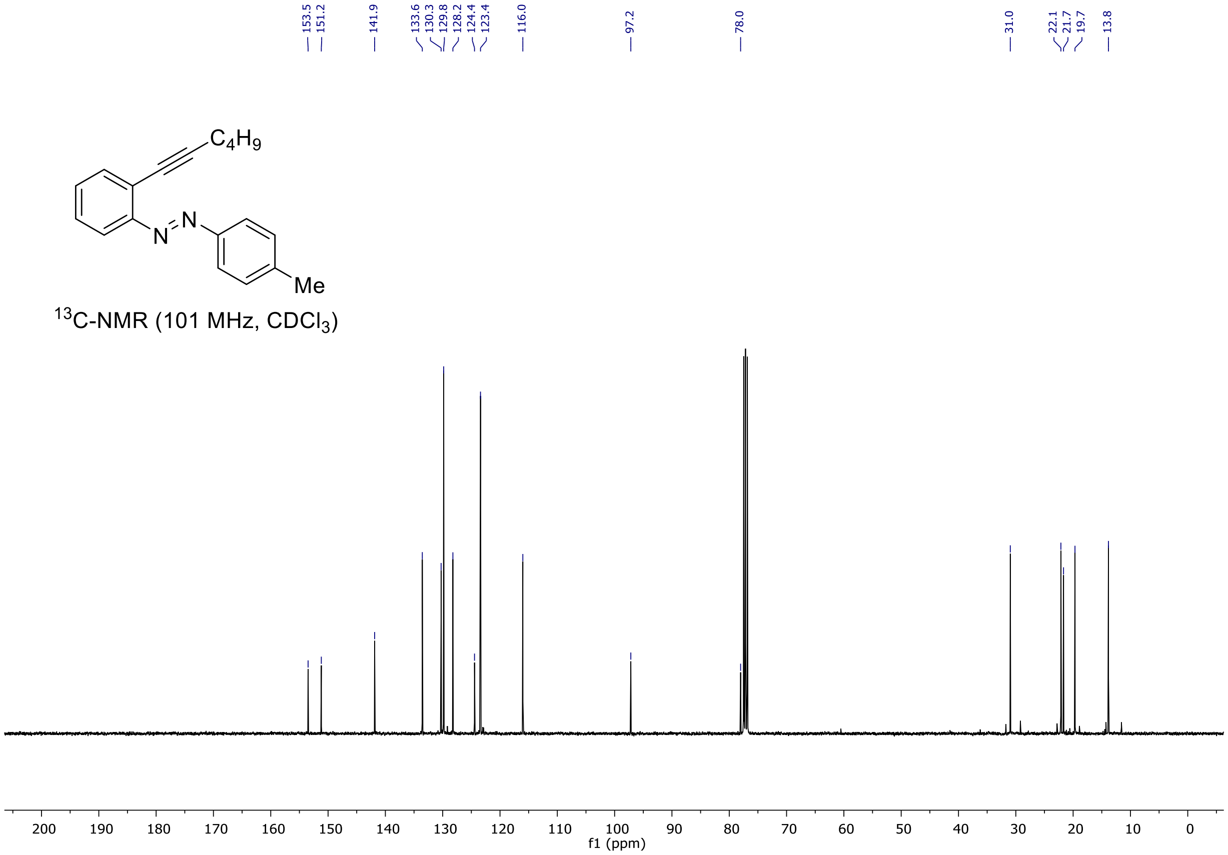

(*E*)-4-[(2-(Hex-1-yn-1-yl)phenyl)diazenyl]-N,N-dimethylaniline (1n)

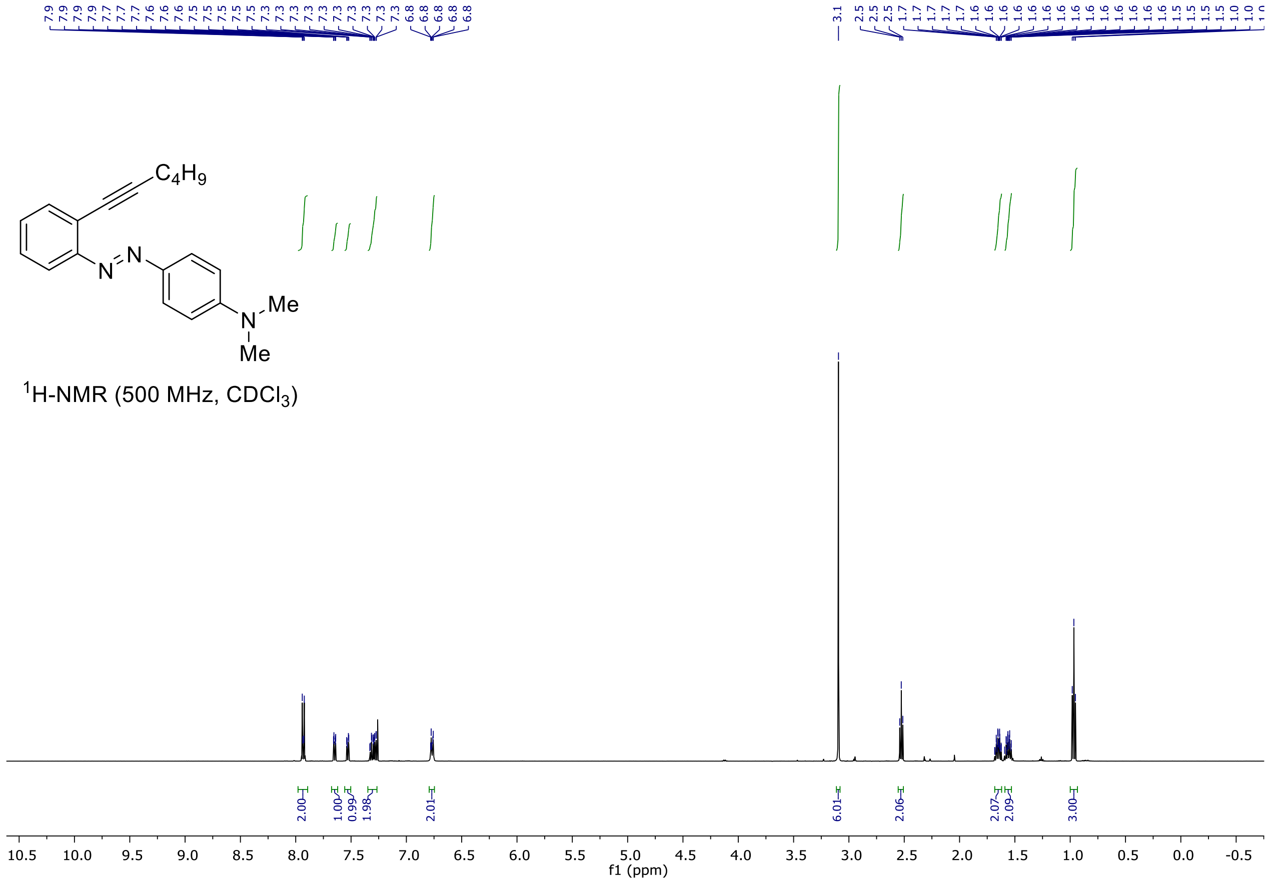

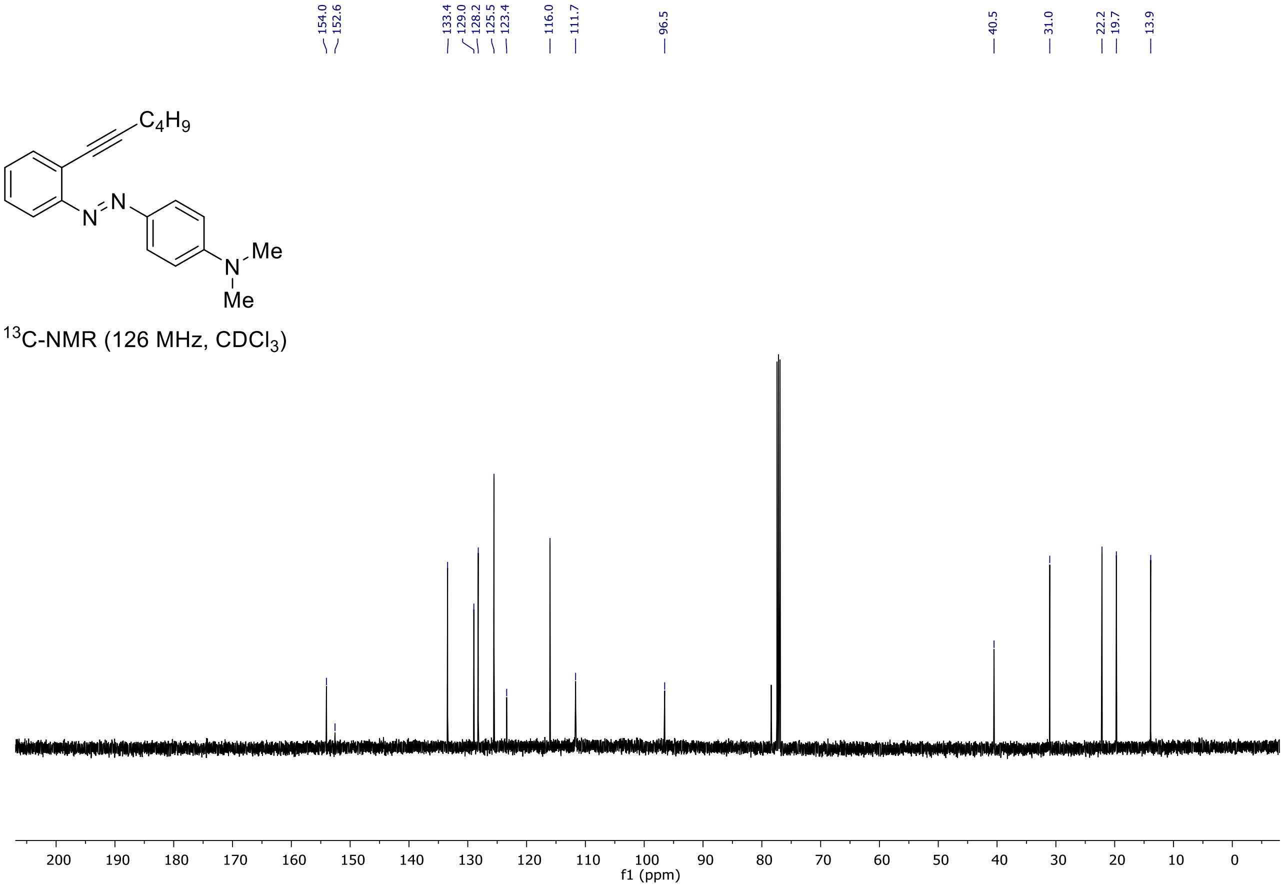

(*E*)-*N*-[4-((2-(Hex-1-yn-1-yl)phenyl)diazenyl)phenyl]-*N*-methylacetamide (1o)

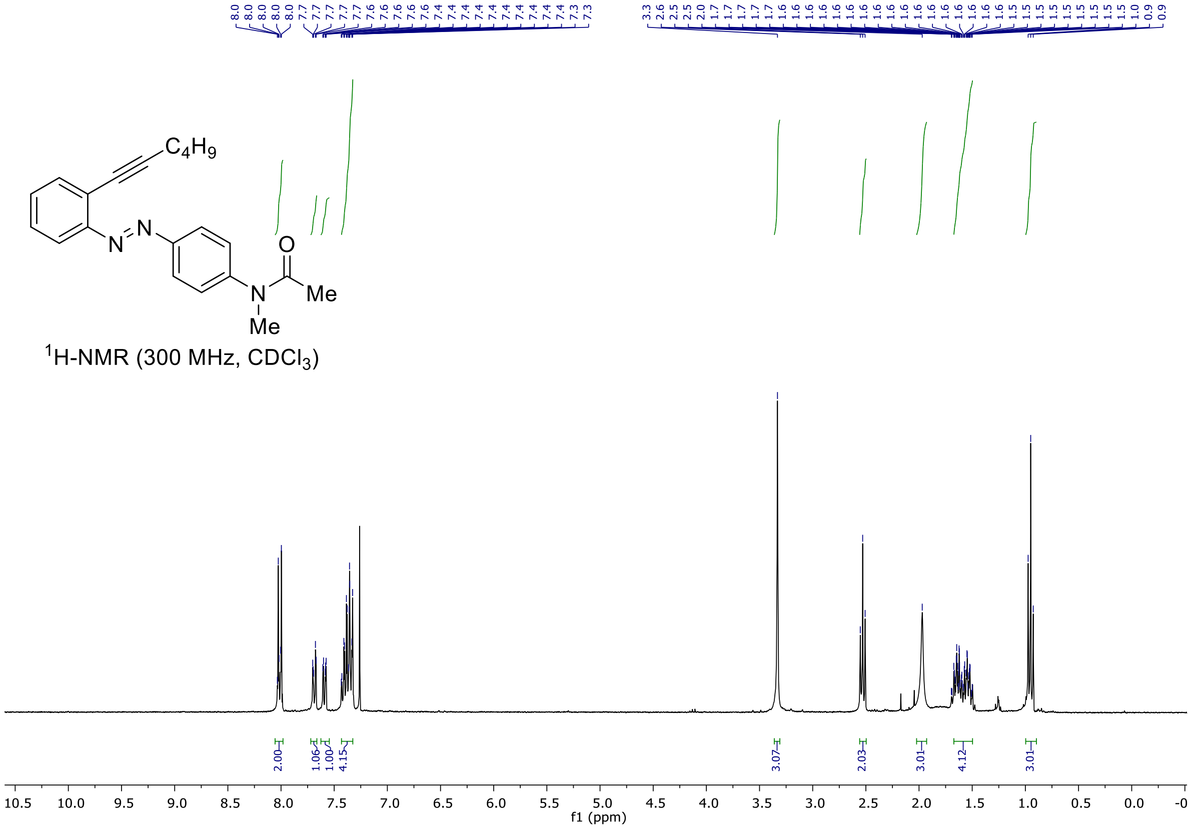

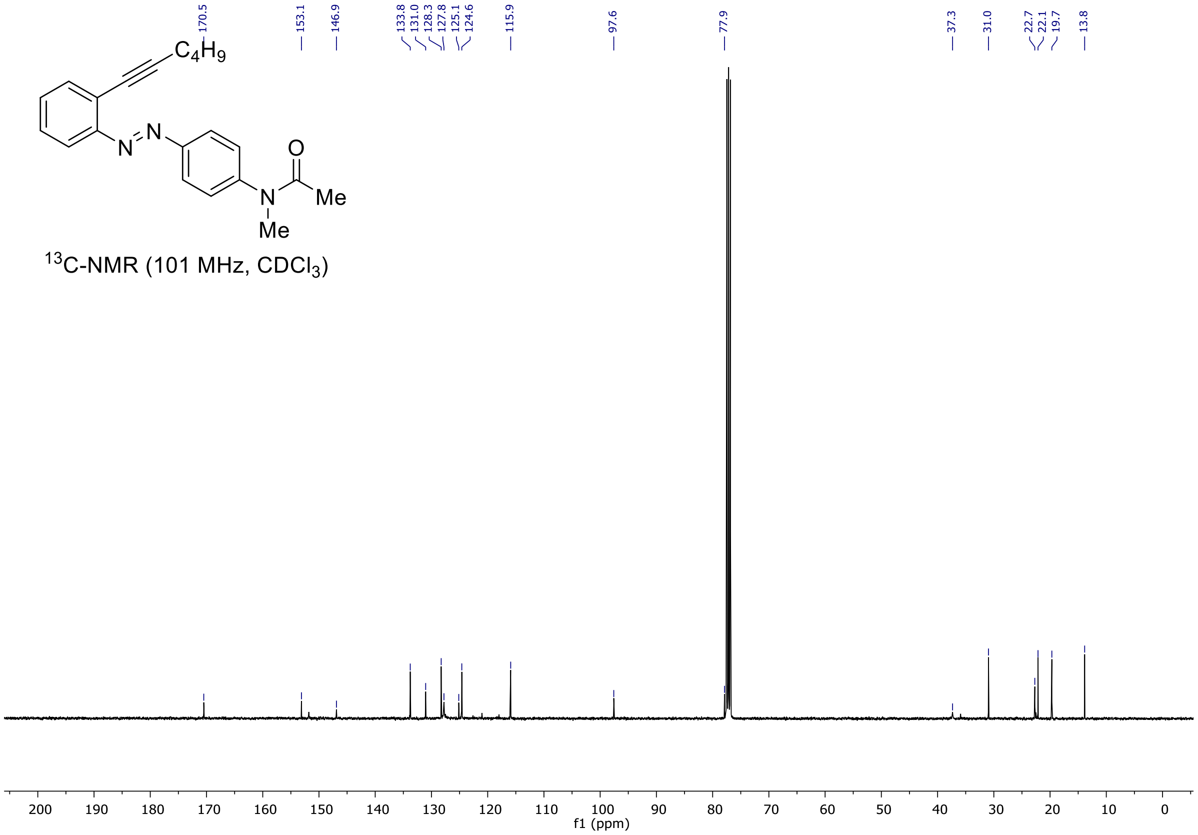

(*E*)-1-[2-(Hex-1-yn-1-yl)phenyl]-2-(4-methoxyphenyl)diazene (1p)

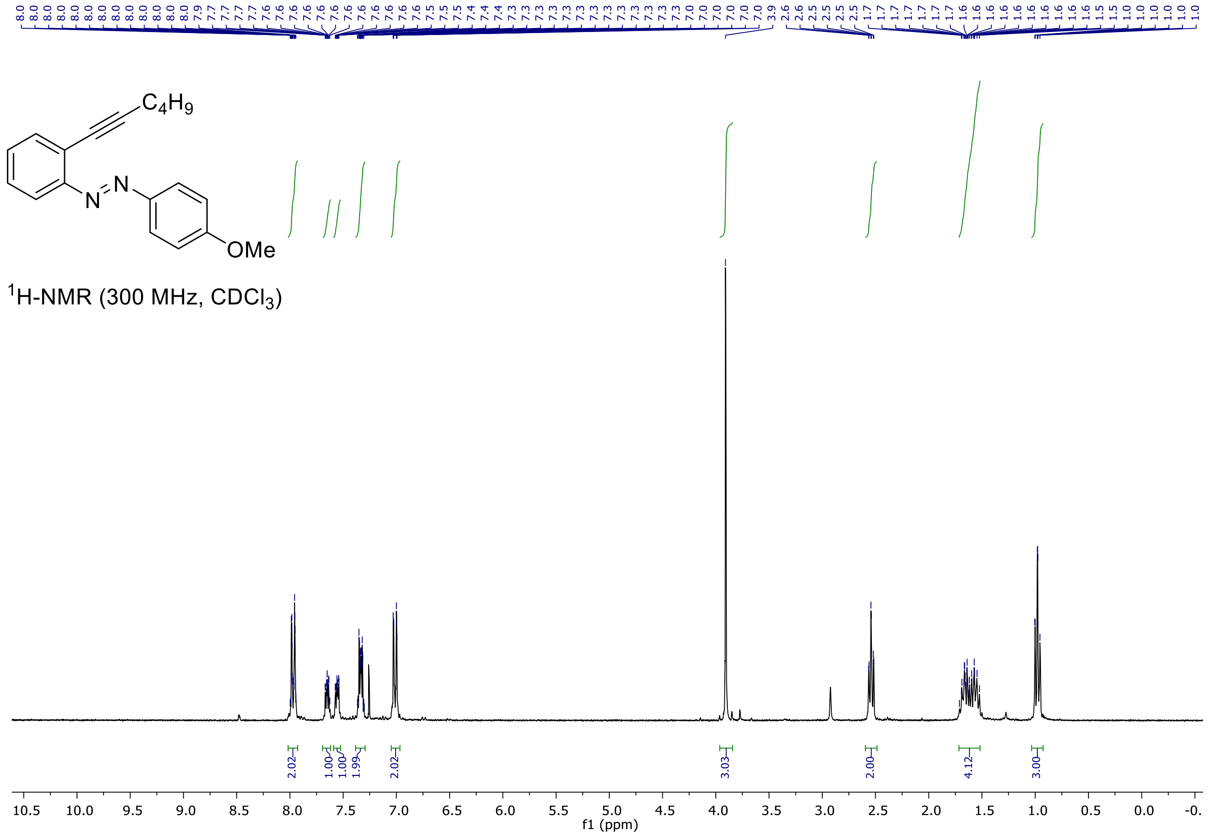

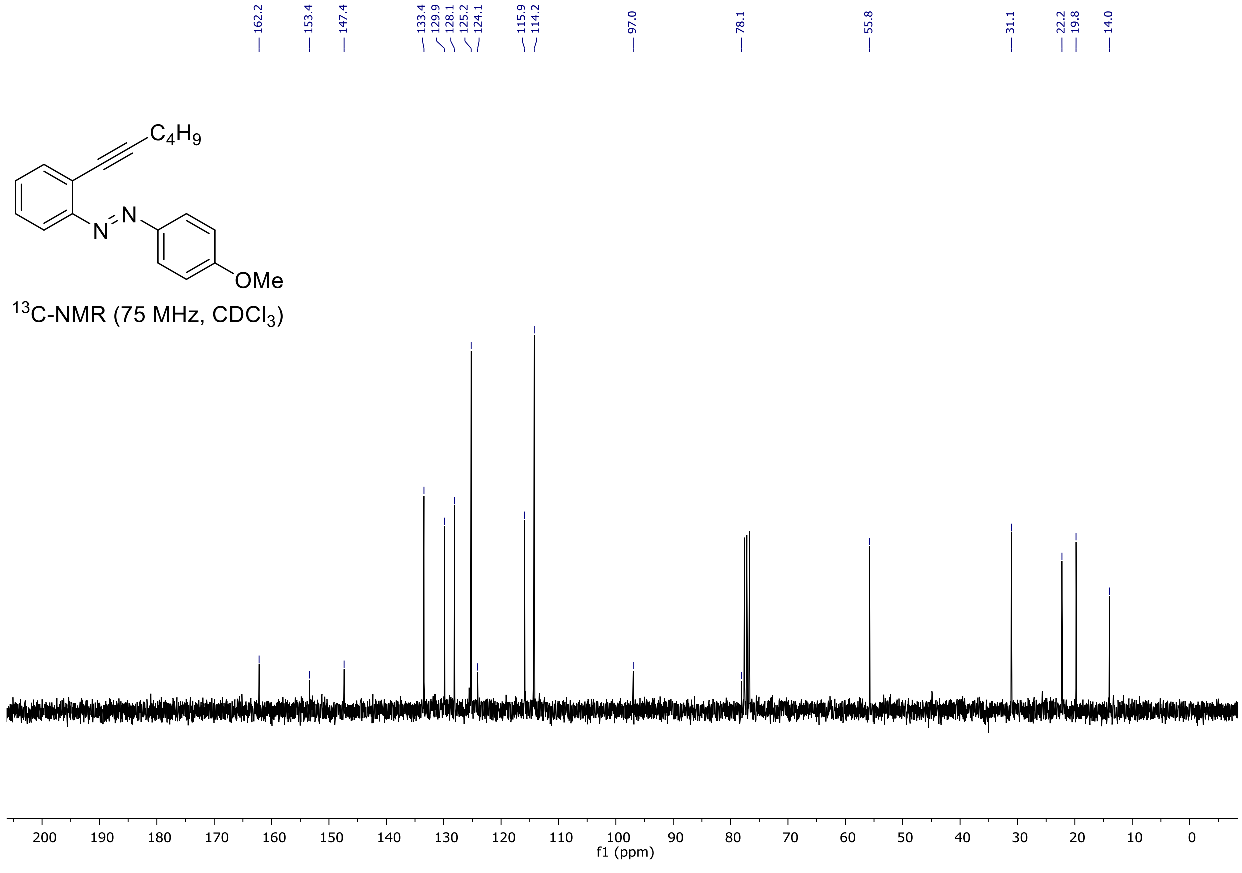

(*E*)-4-[(2-(Hex-1-yn-1-yl)phenyl)diazenyl]phenyl acetate (1q)

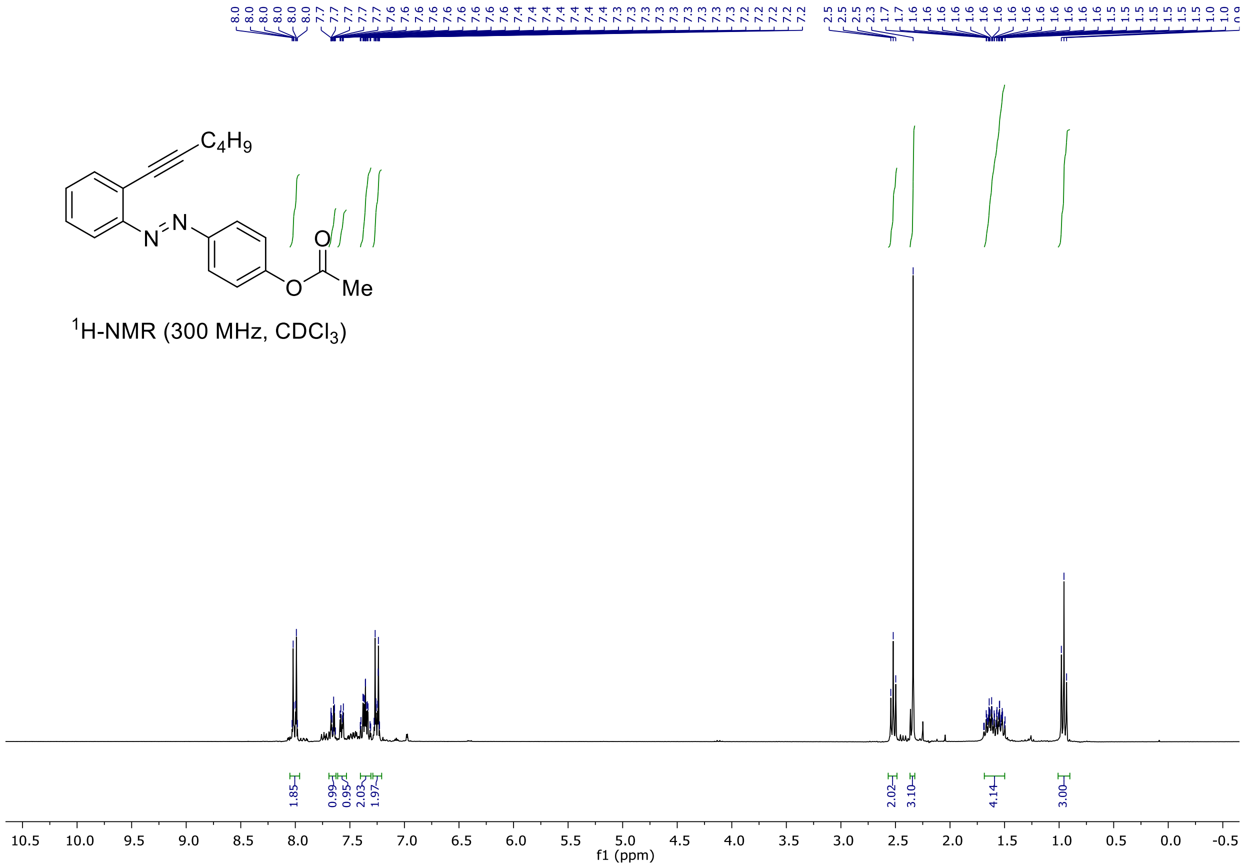

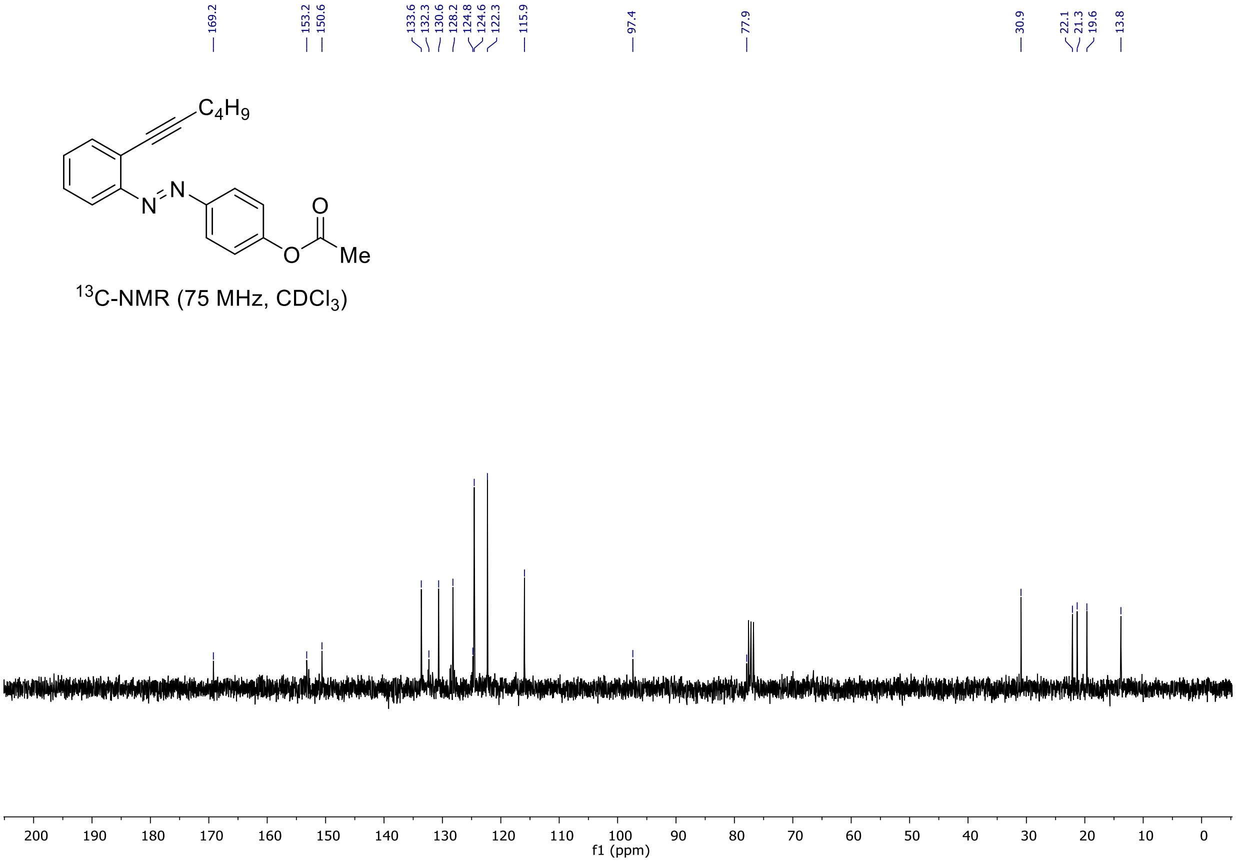

(*E*)-1-[5-Chloro-2-(hex-1-yn-1-yl)phenyl]-2-phenyldiazene (1r)

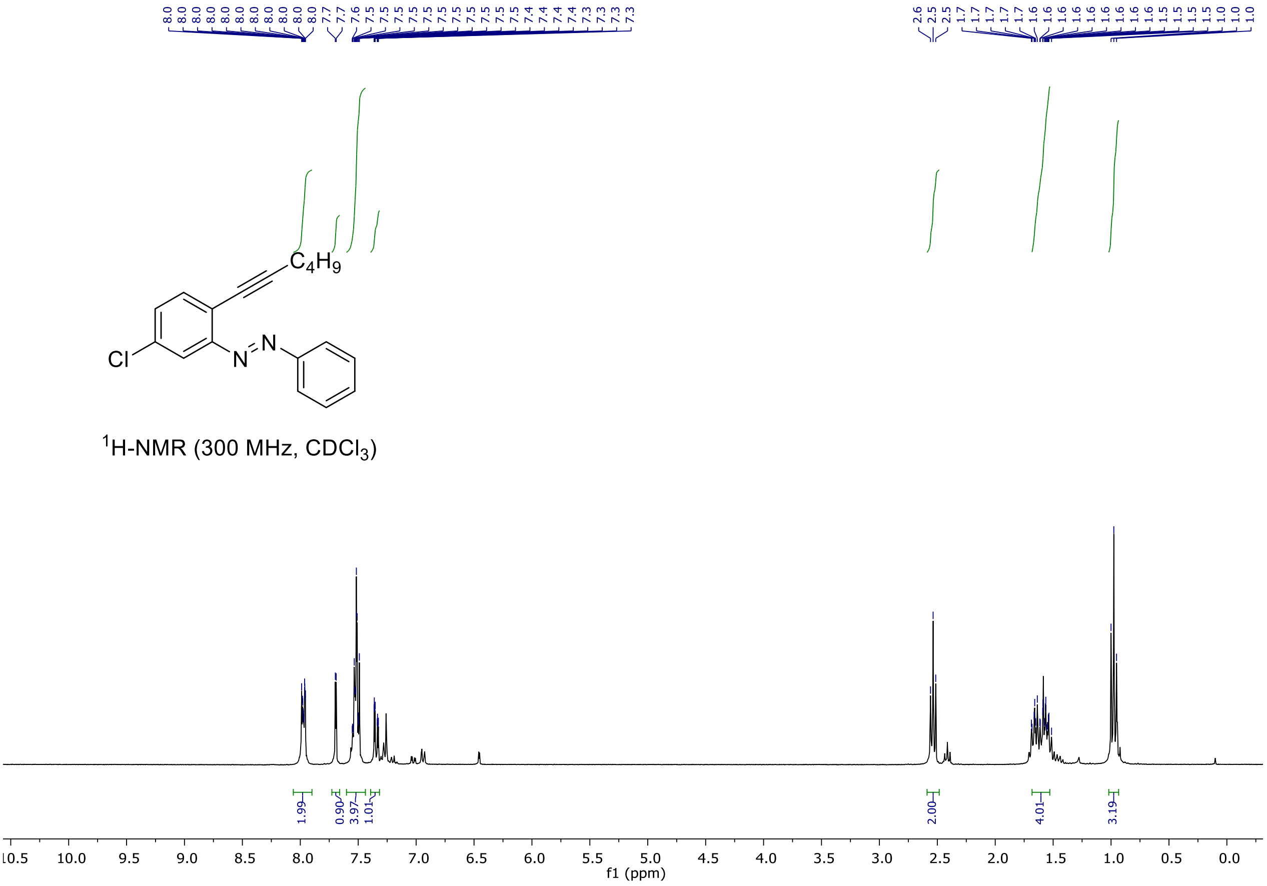

^{1}H-NMR (300 MHz, $CDCl_3$)

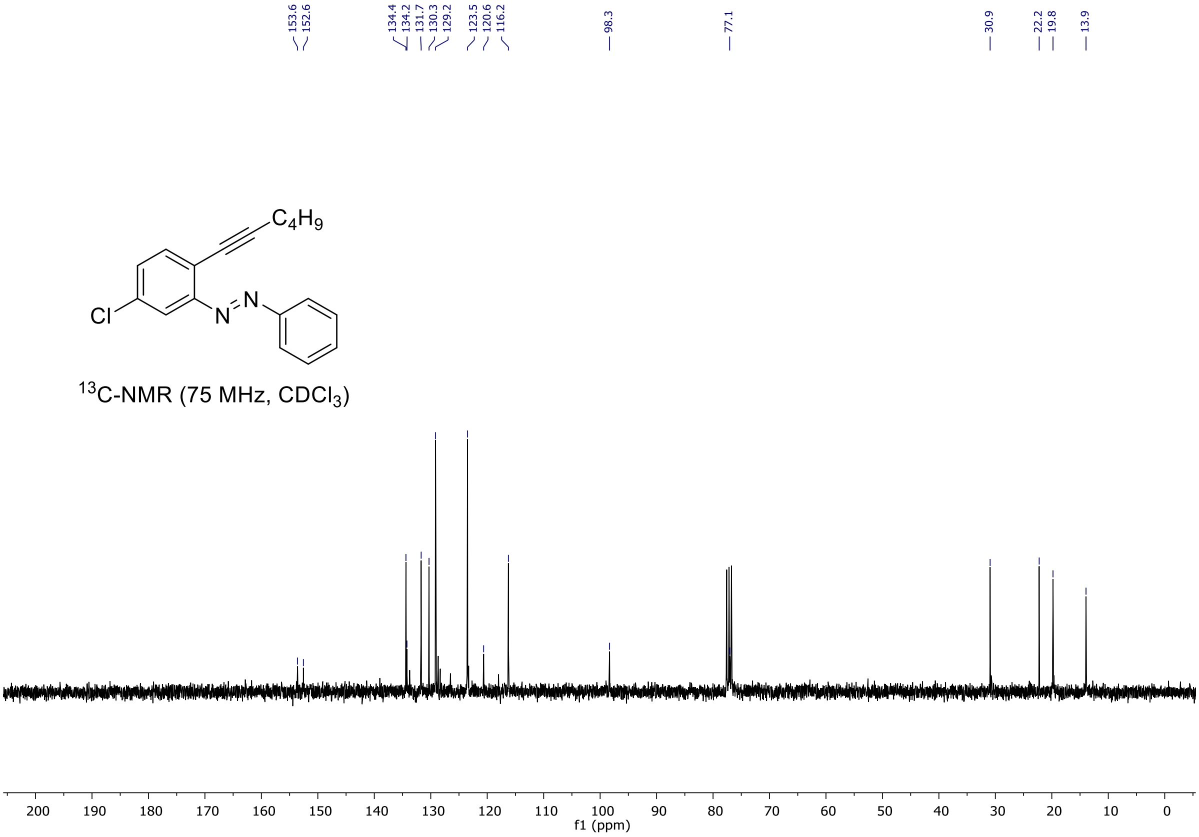

^{13}C-NMR (75 MHz, $CDCl_3$)

(*E*)-1-[2-(Hex-1-yn-1-yl)-4-(trifluoromethyl)phenyl]-2-phenyldiazene (1s)

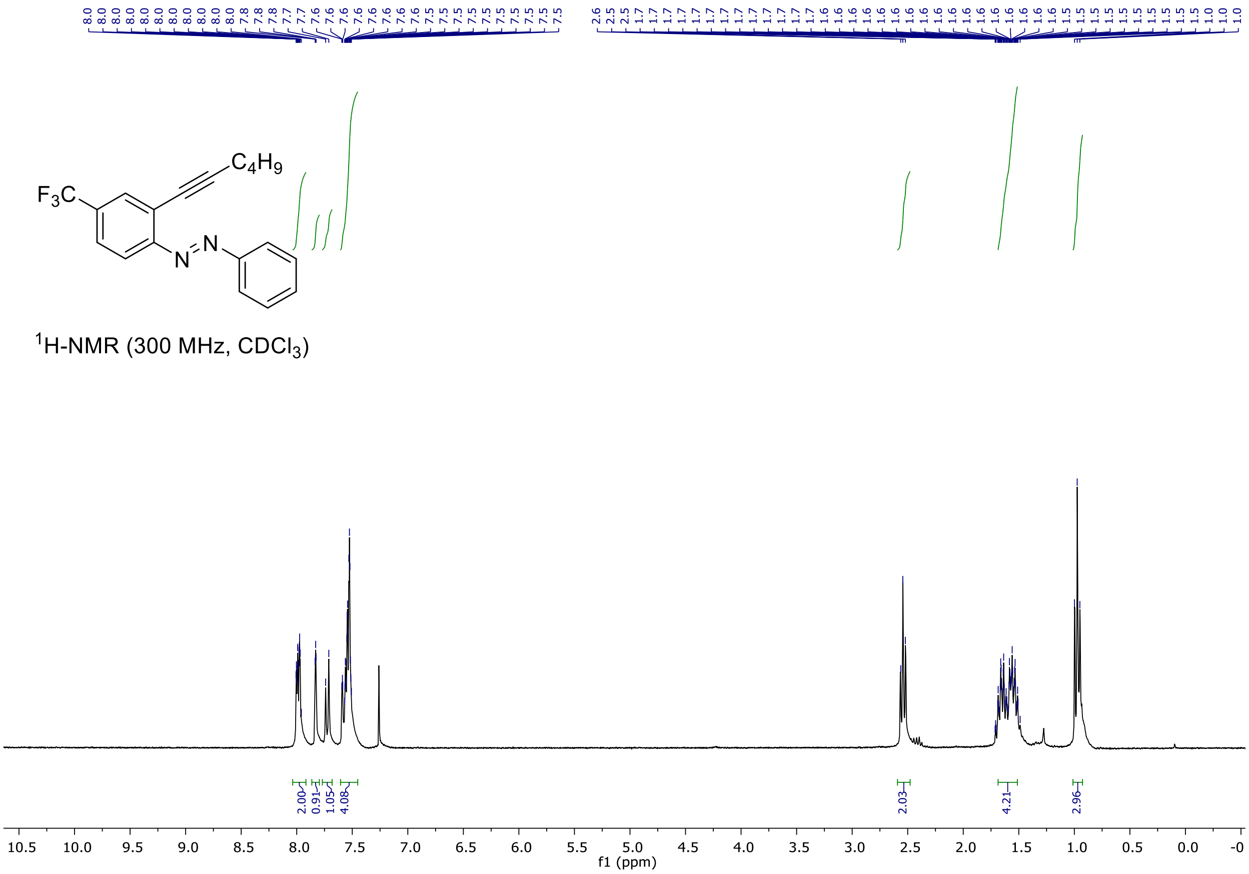

^{1}H-NMR (300 MHz, $CDCl_3$)

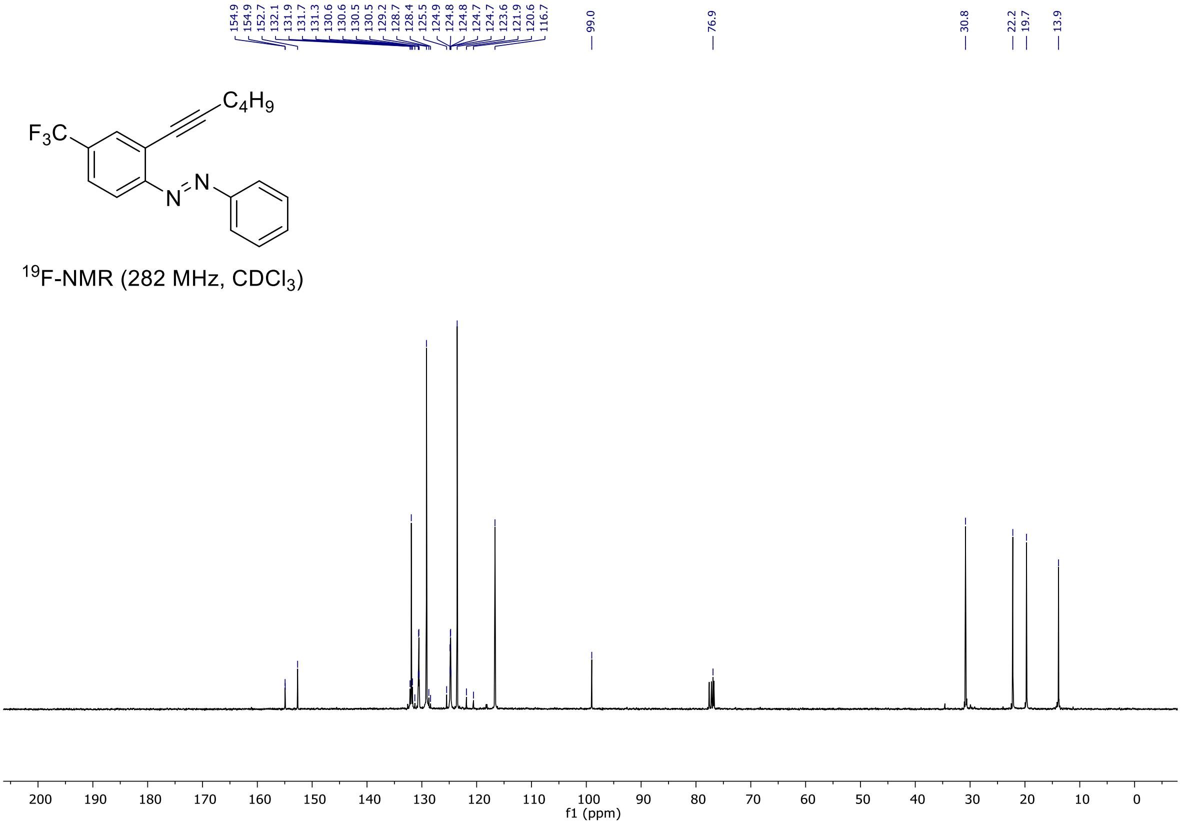

^{19}F-NMR (282 MHz, $CDCl_3$)

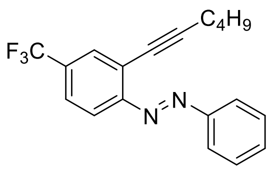

^{13}C-NMR (75 MHz, $CDCl_3$)

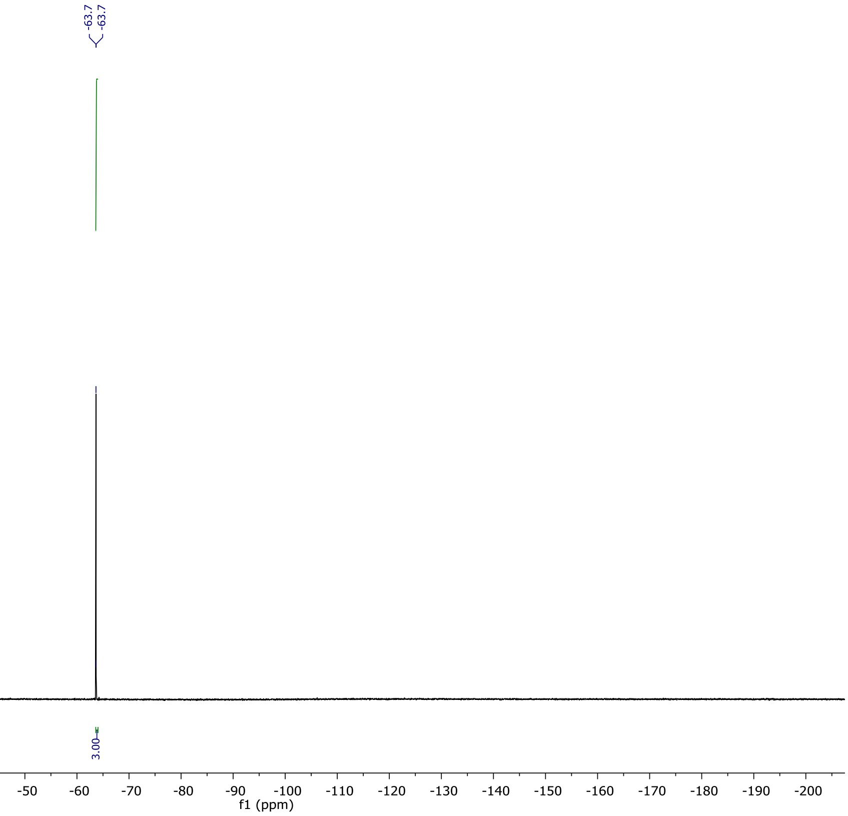

Methyl (*E*)-3-(hex-1-yn-1-yl)-4-(phenyldiazenyl)benzoate (1t)

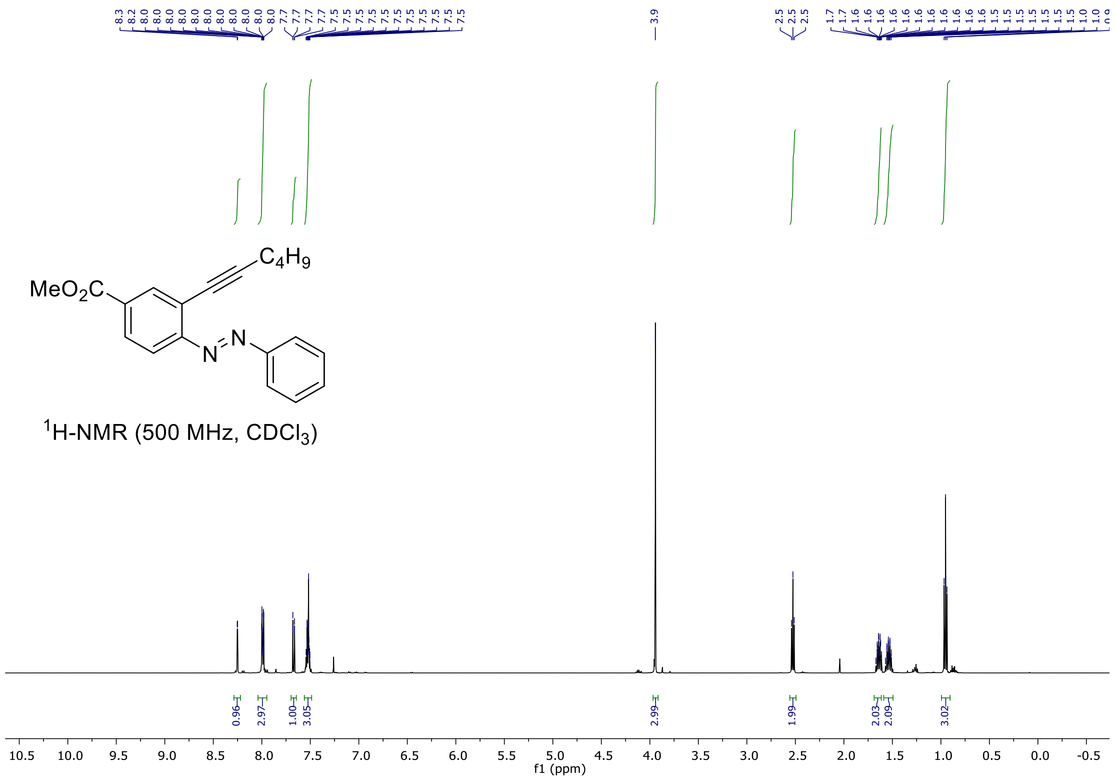

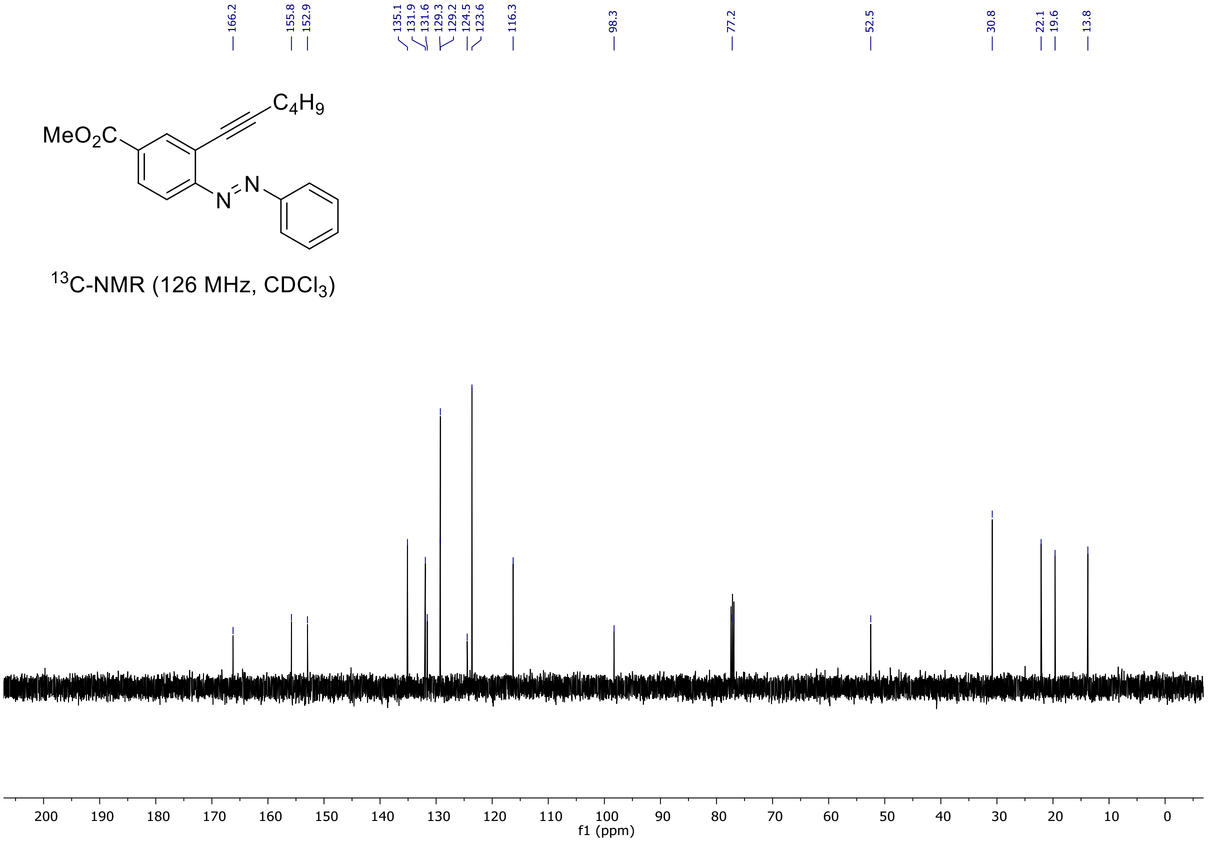

(*E*)-1-[2-(Hex-1-yn-1-yl)-4-methylphenyl]-2-phenyldiazene (1u)

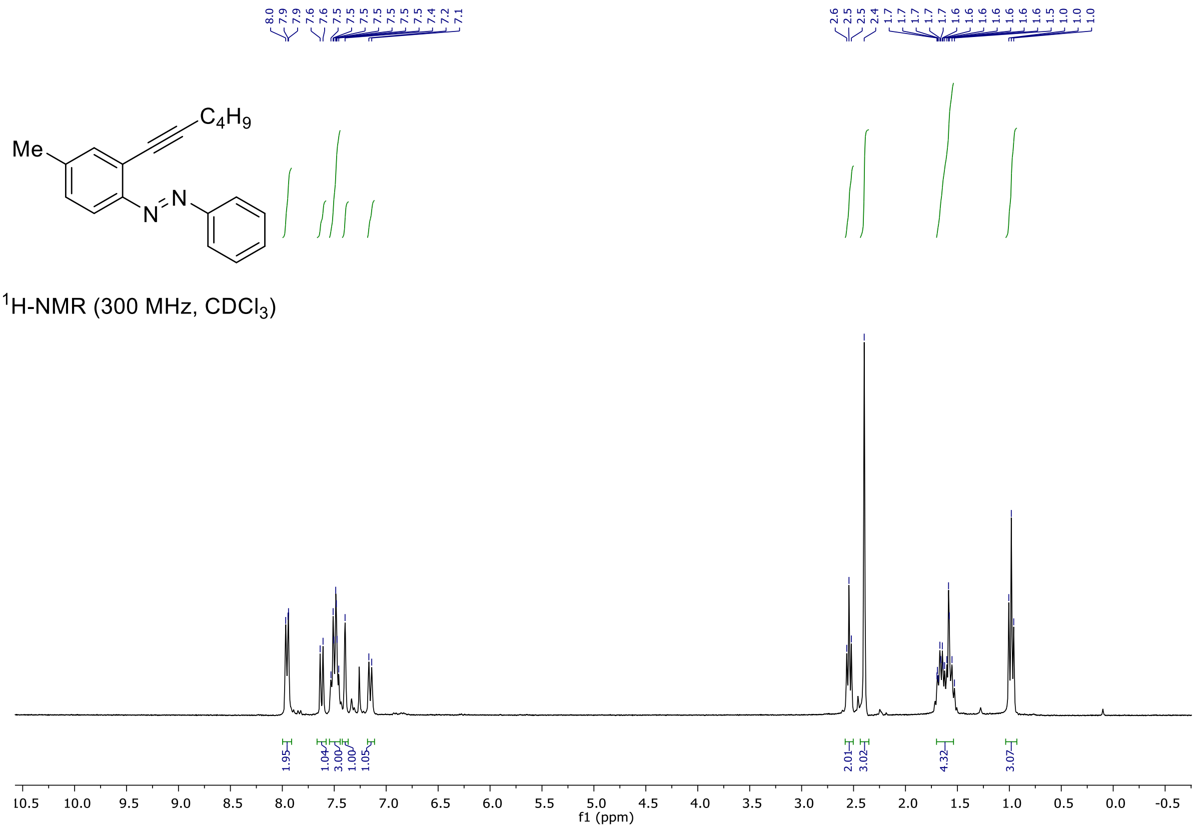

^{1}H-NMR (300 MHz, $CDCl_3$)

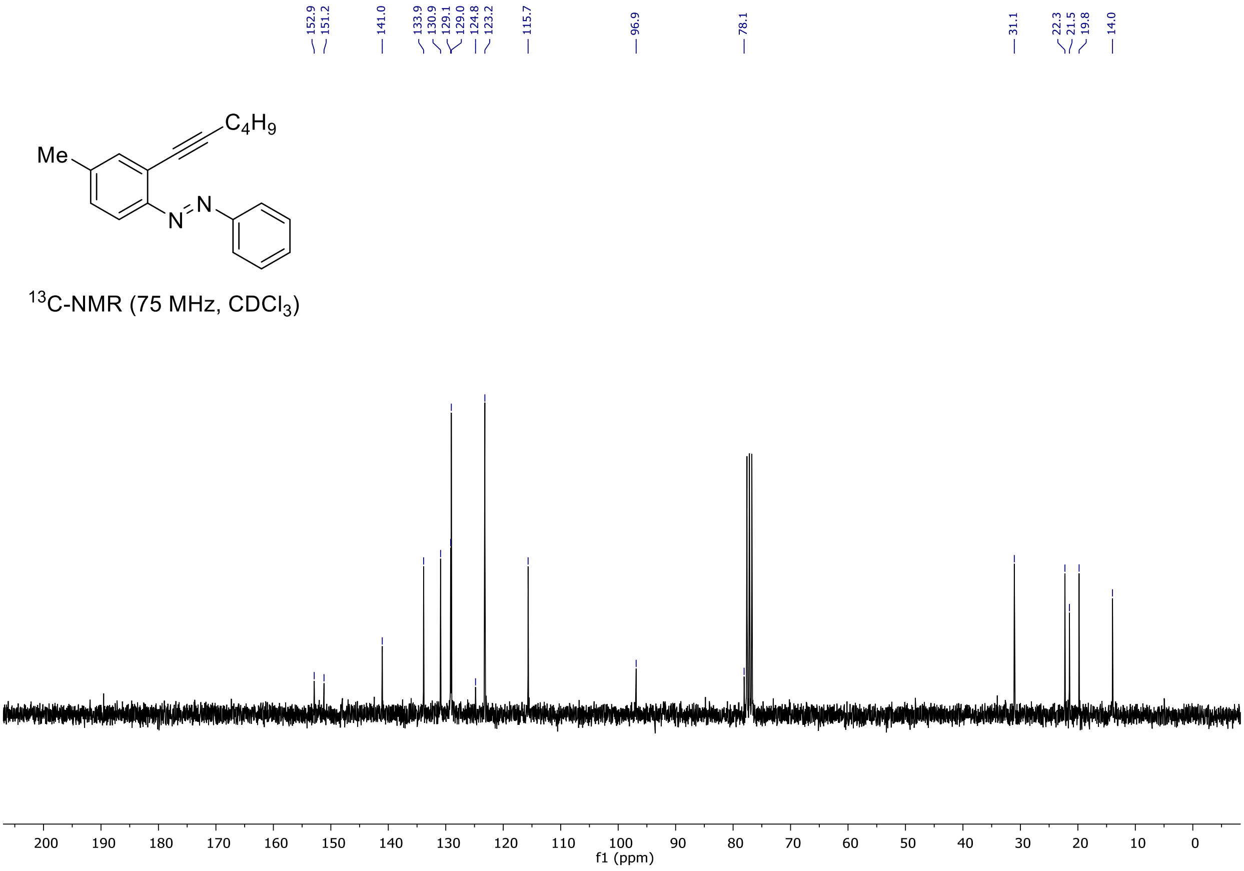

^{13}C-NMR (75 MHz, $CDCl_3$)

(*E*)-1-[2-(Hex-1-yn-1-yl)-3-methylphenyl]-2-phenyldiazene (1v)

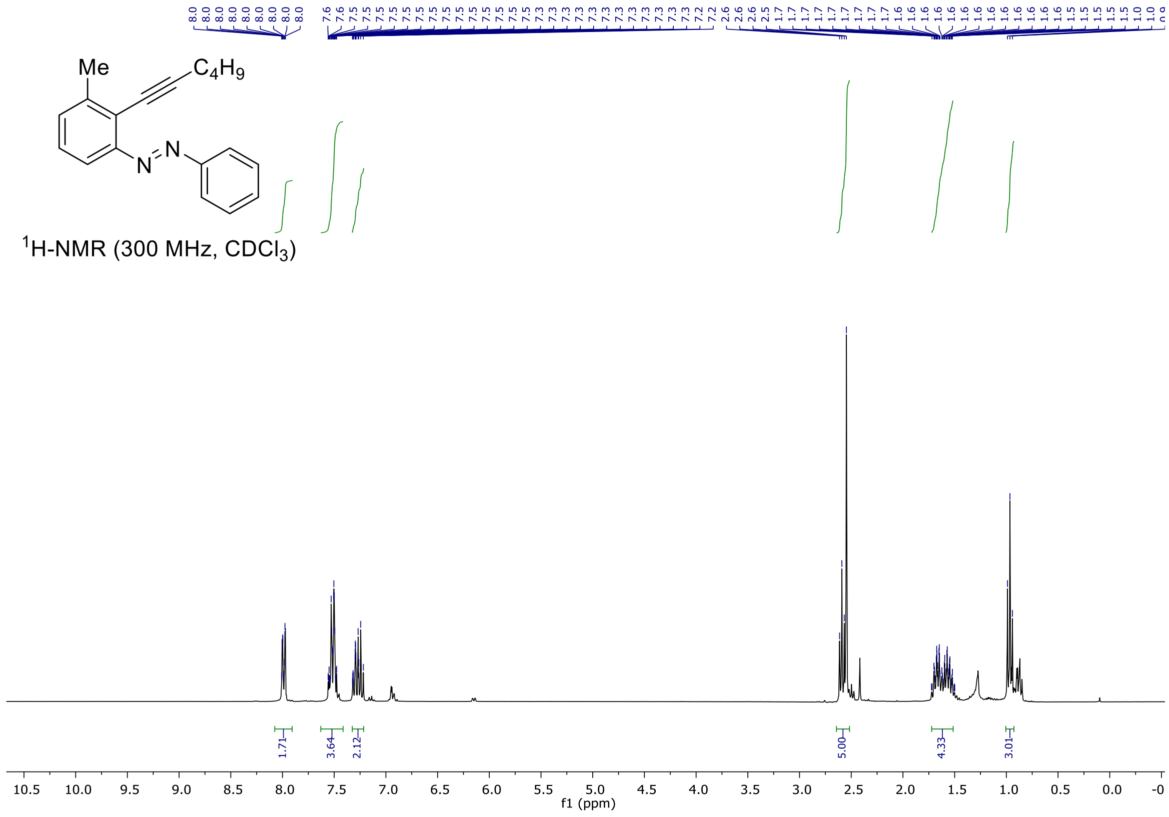

^{1}H-NMR (300 MHz, $CDCl_3$)

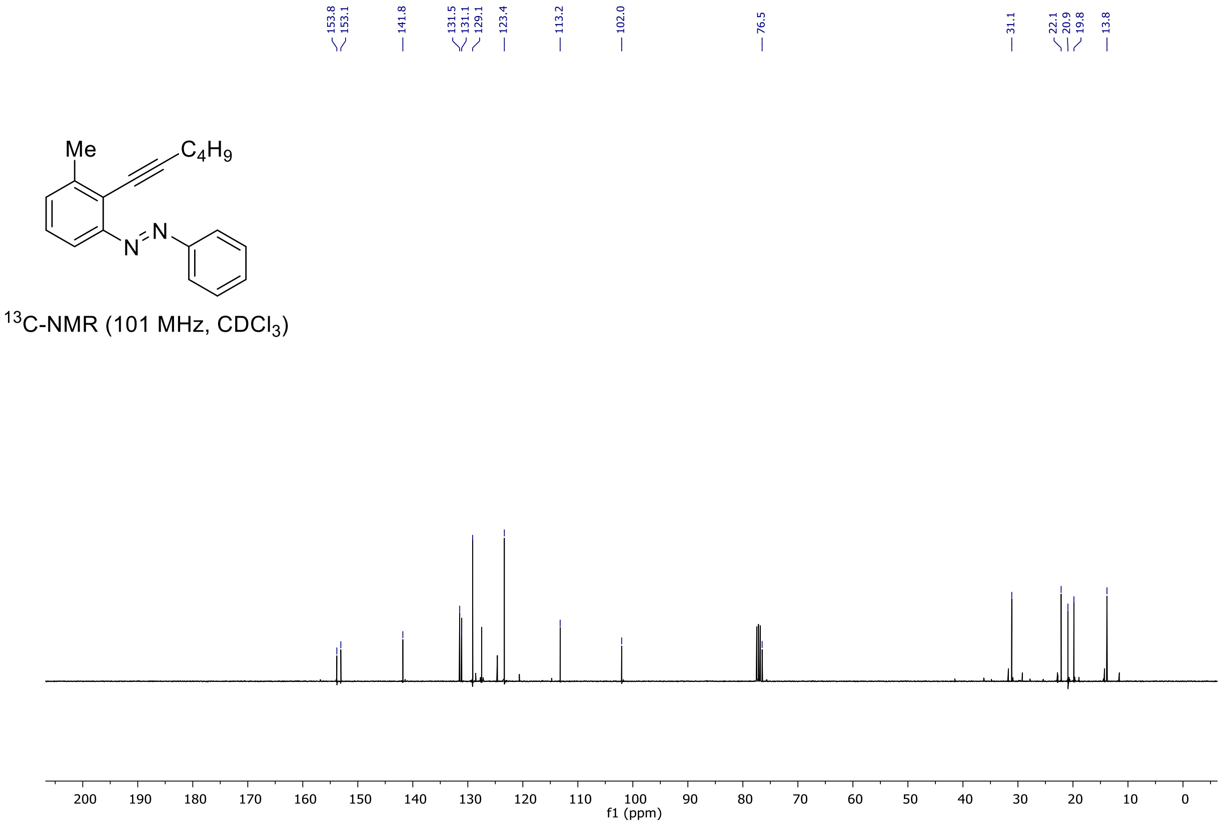

^{13}C-NMR (101 MHz, $CDCl_3$)

(*E*)-1-(2-Ethynylphenyl)-2-phenyldiazene

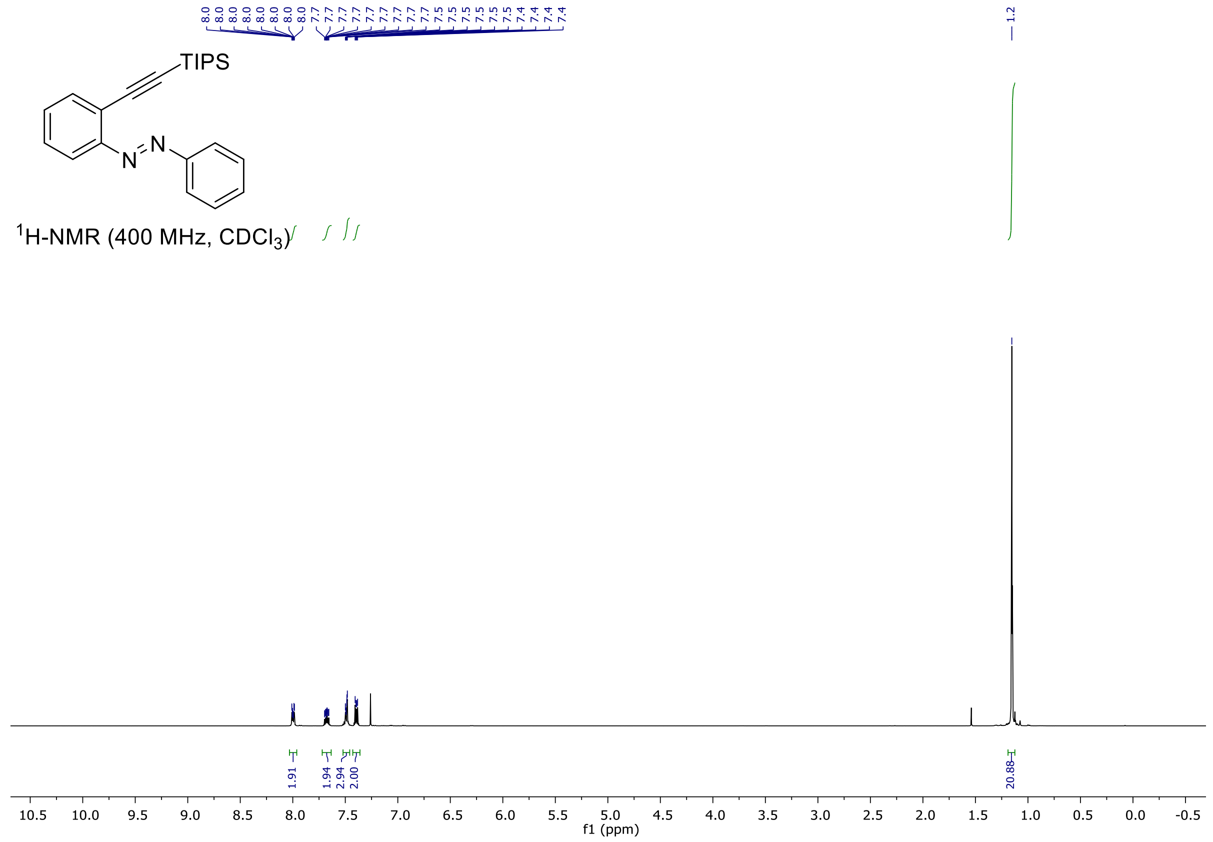

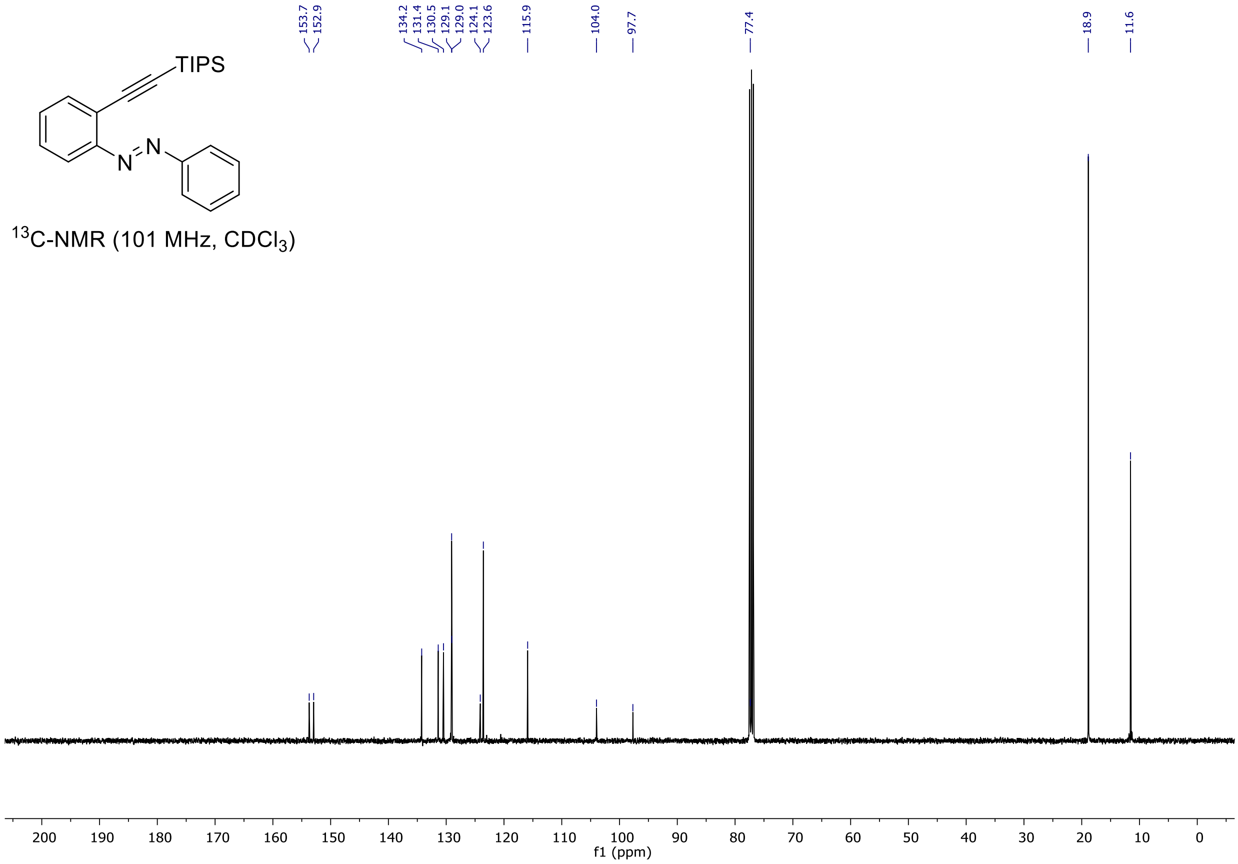

(*E*)-1-[2-(5-methylhex-1-yn-1-yl)phenyl]-2-phenyldiazene (1x)

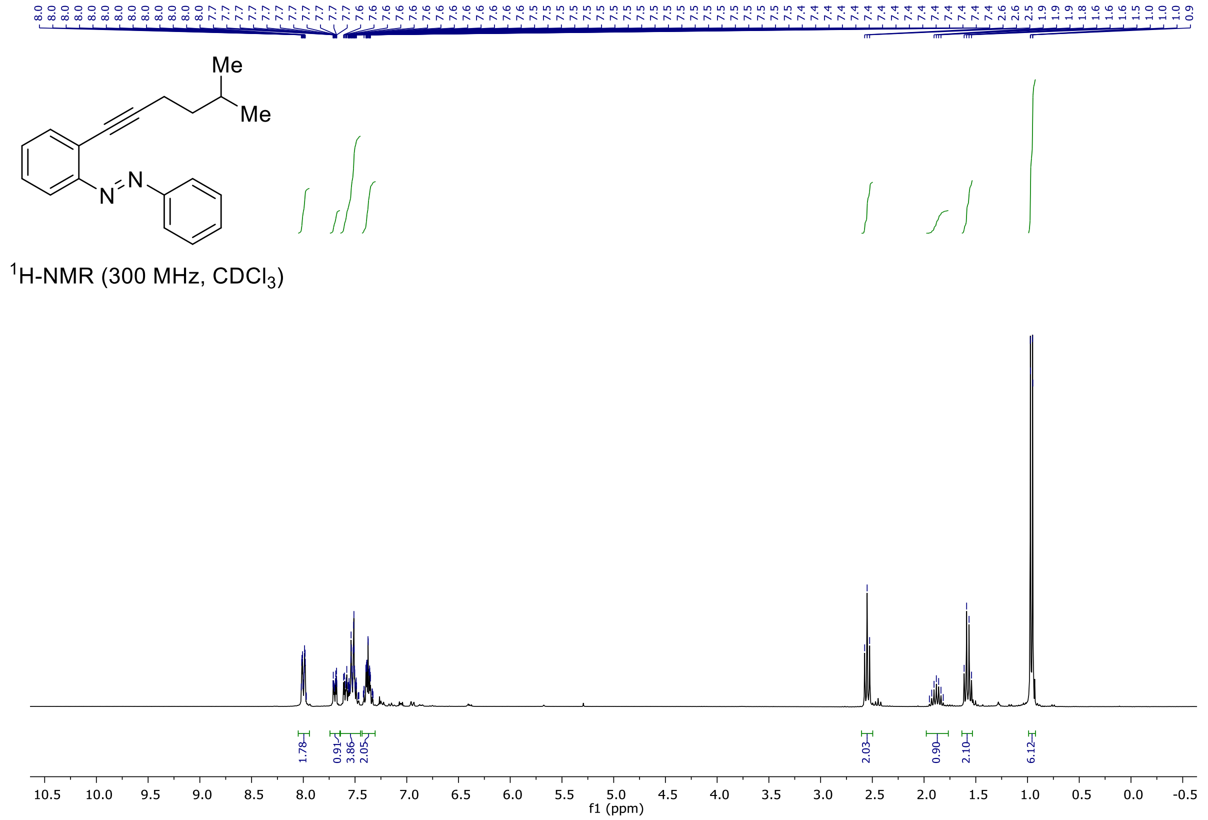

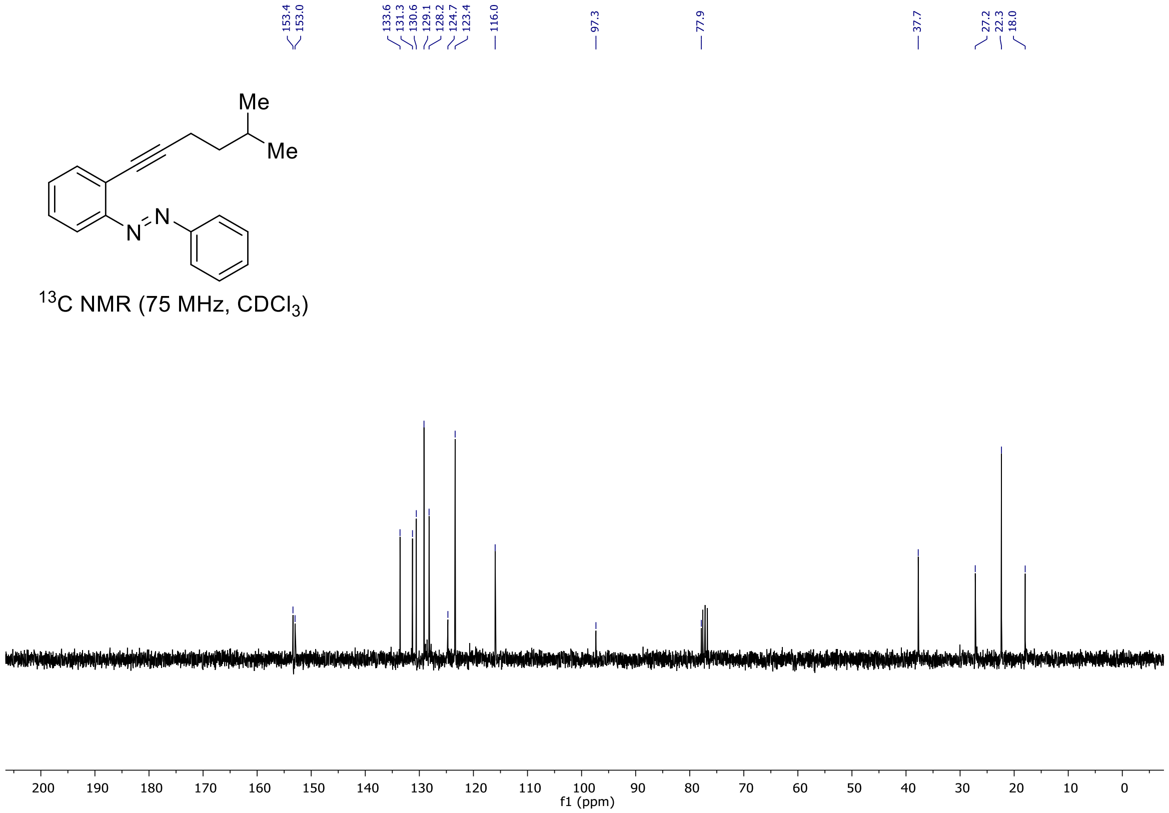

(*E*)-1-[2-(3-Cyclohexylprop-1-yn-1-yl)phenyl]-2-phenyldiazene (1y)

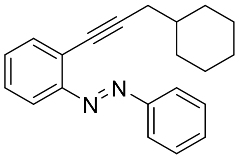

^{1}H-NMR (300 MHz, $CDCl_3$)

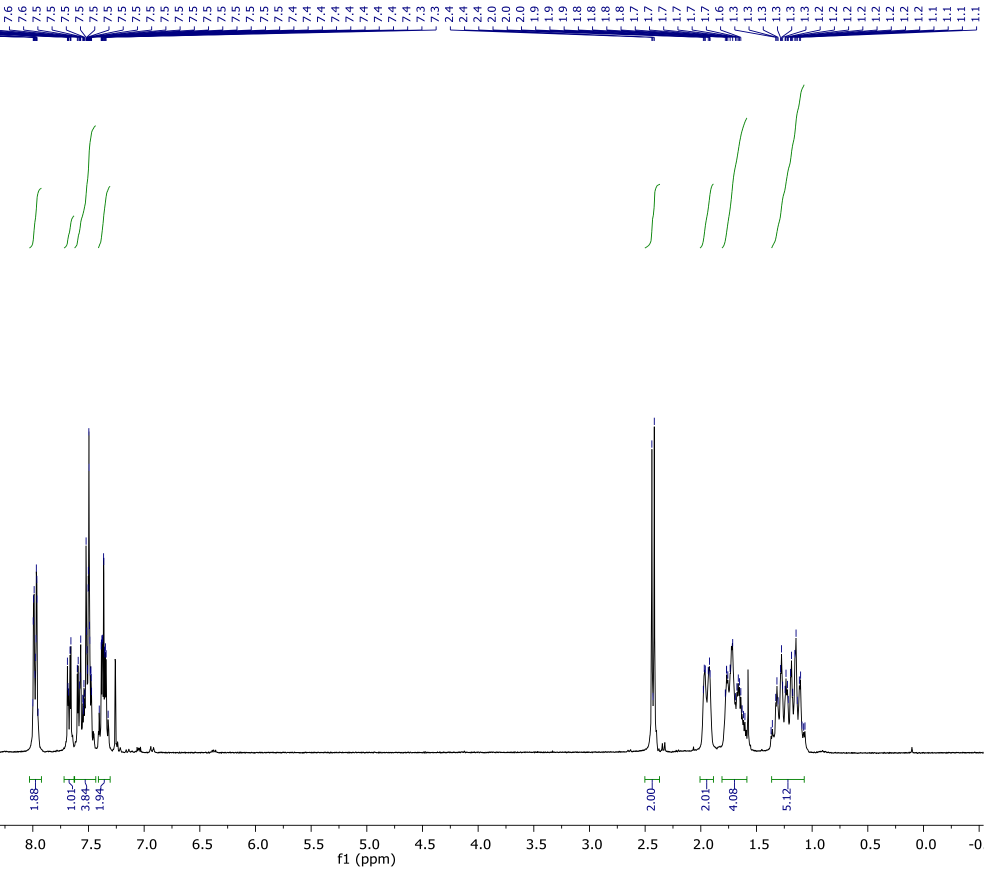

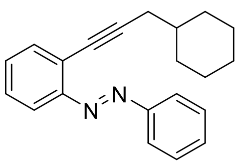

^{13}C-NMR (75 MHz, $CDCl_3$)

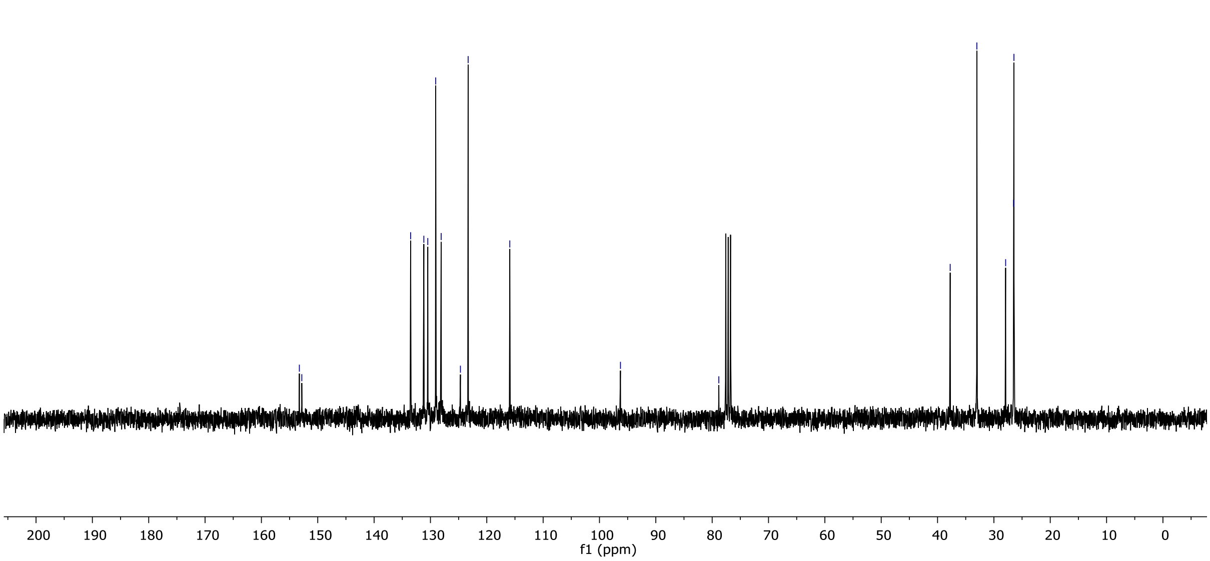

(*E*)-1-(4-Bromophenyl)-2-[2-(cyclopropylethynyl)phenyl]diazene (1z)

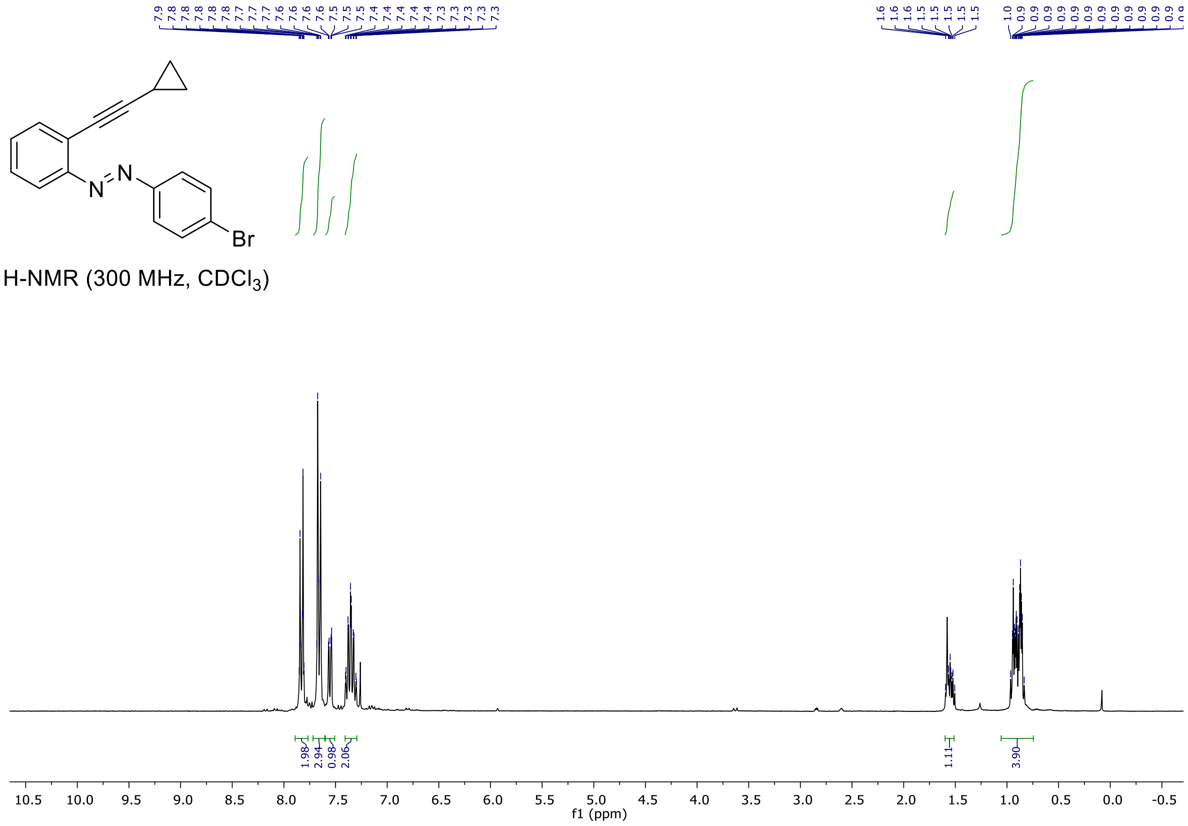

^{1}H-NMR (300 MHz, $CDCl_3$)

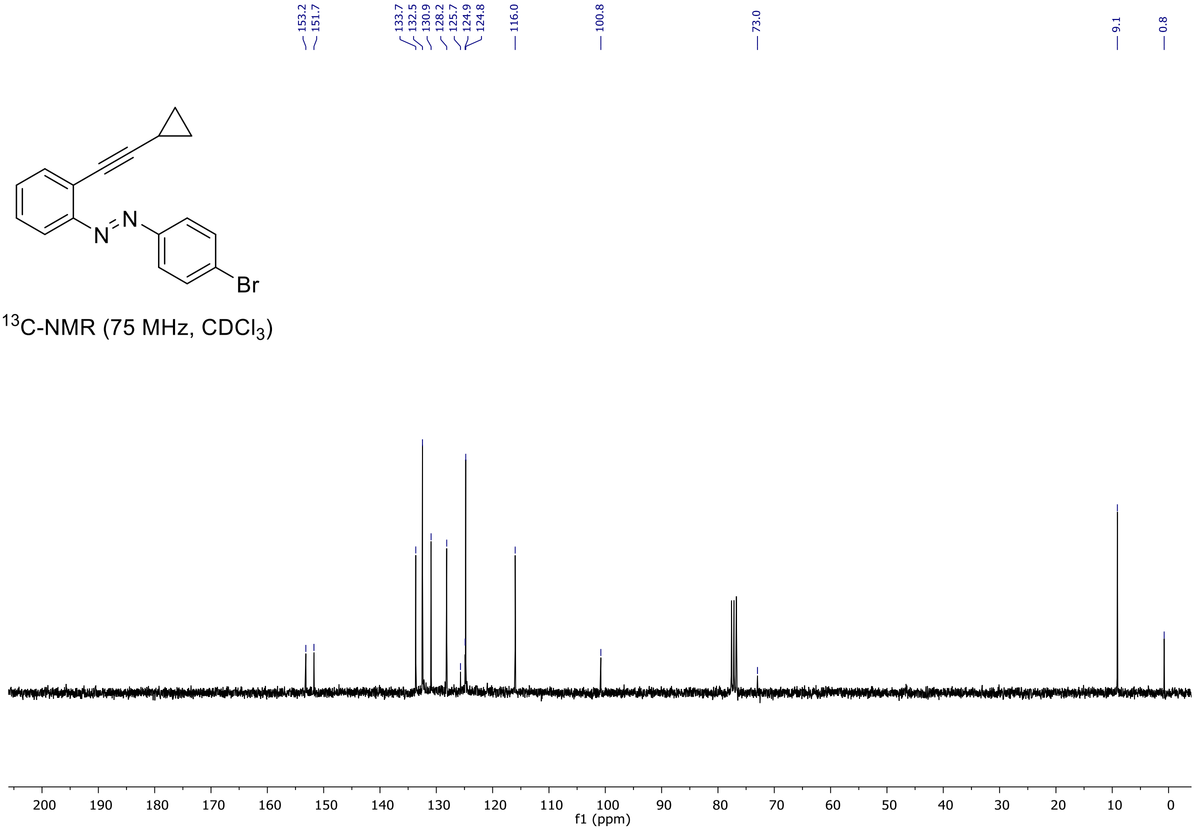

^{13}C-NMR (75 MHz, $CDCl_3$)

(*E*)-1-(2-(5-Chloropent-1-yn-1-yl)phenyl]-2-phenyldiazene (1aa)

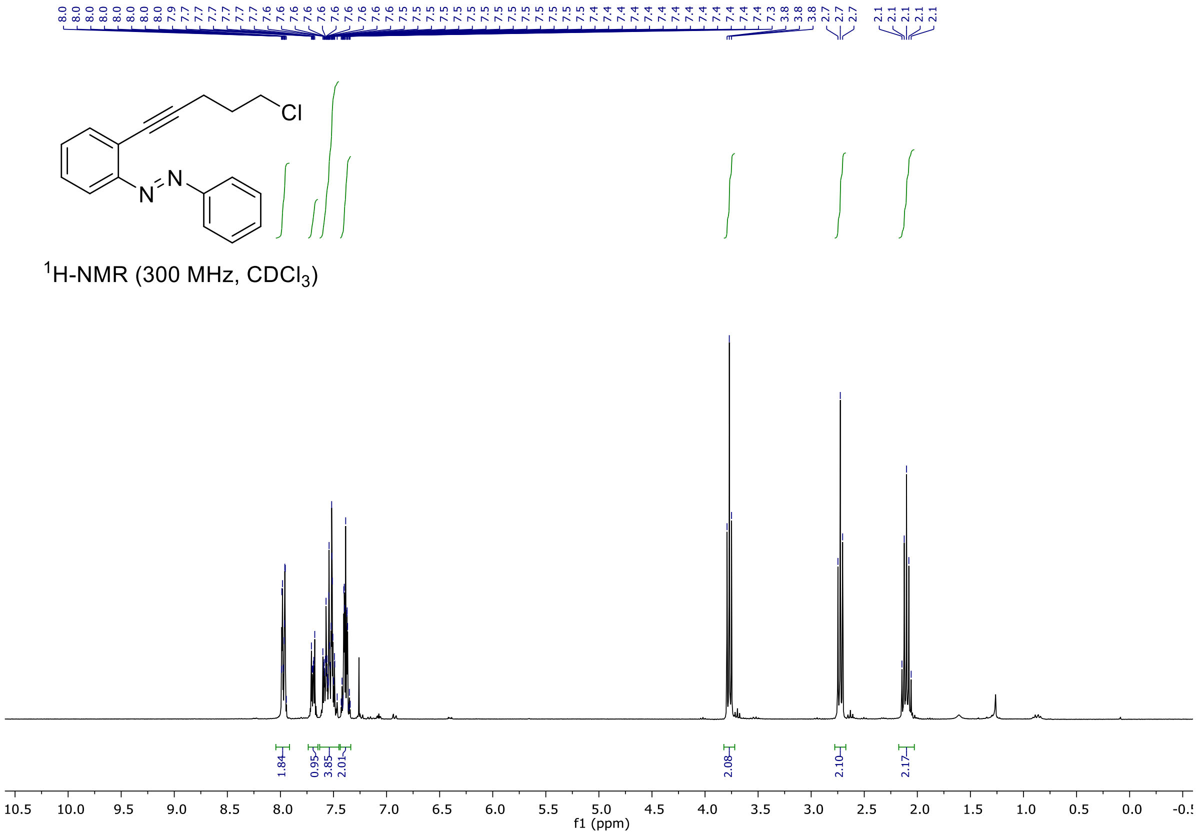

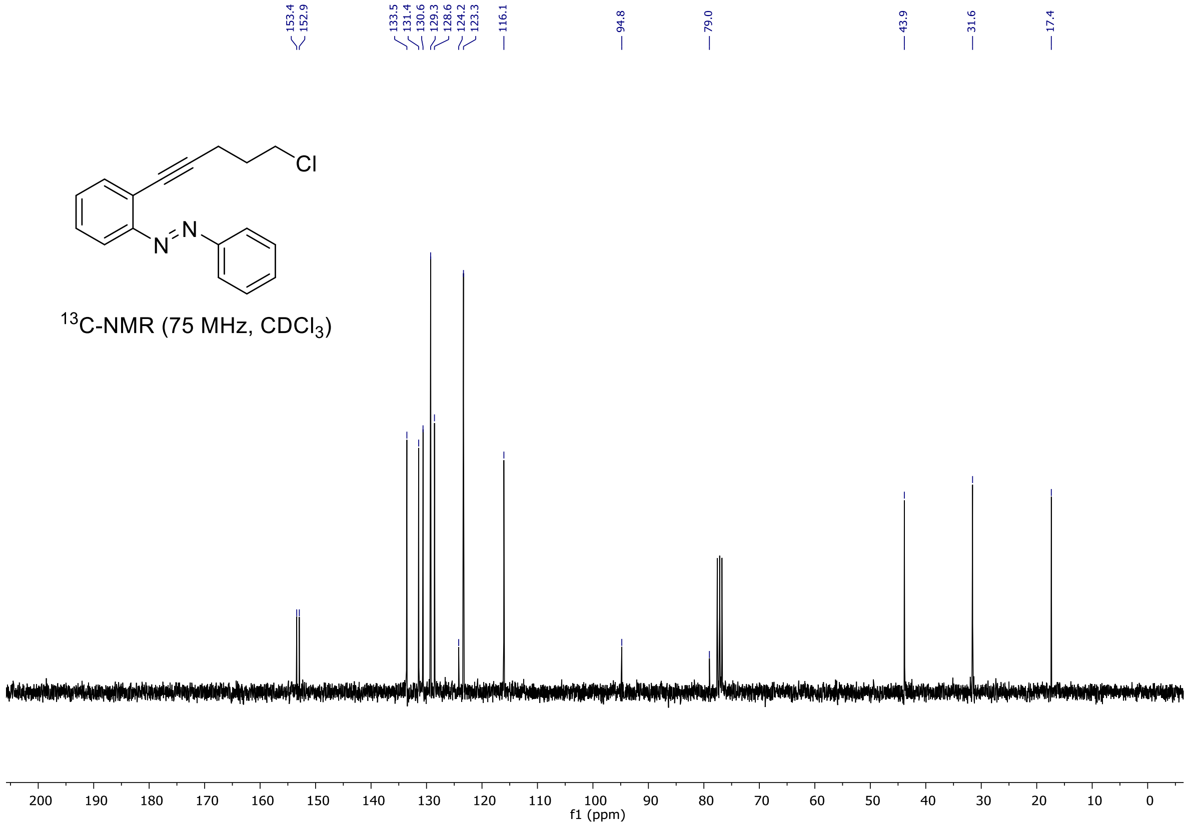

(*E*)-4-[2-(Phenyldiazenyl)phenyl]but-3-yn-1-ol (1ab)

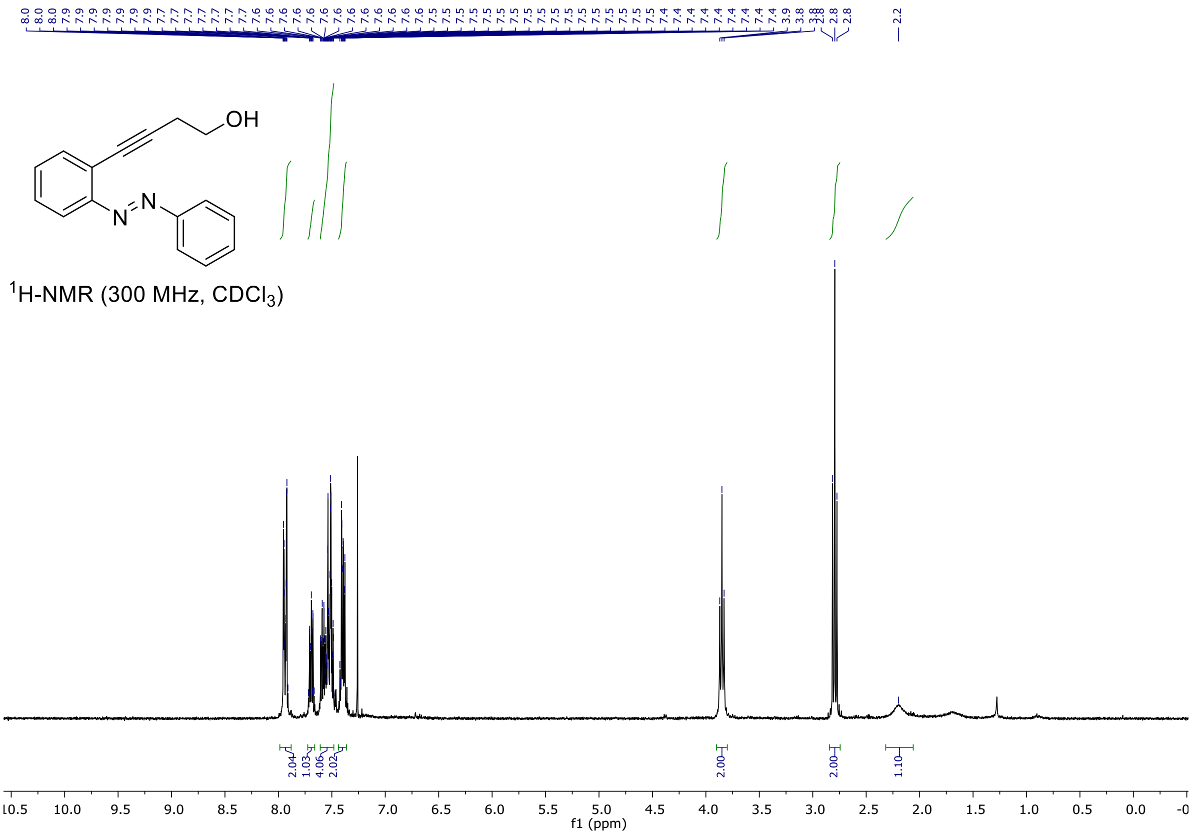

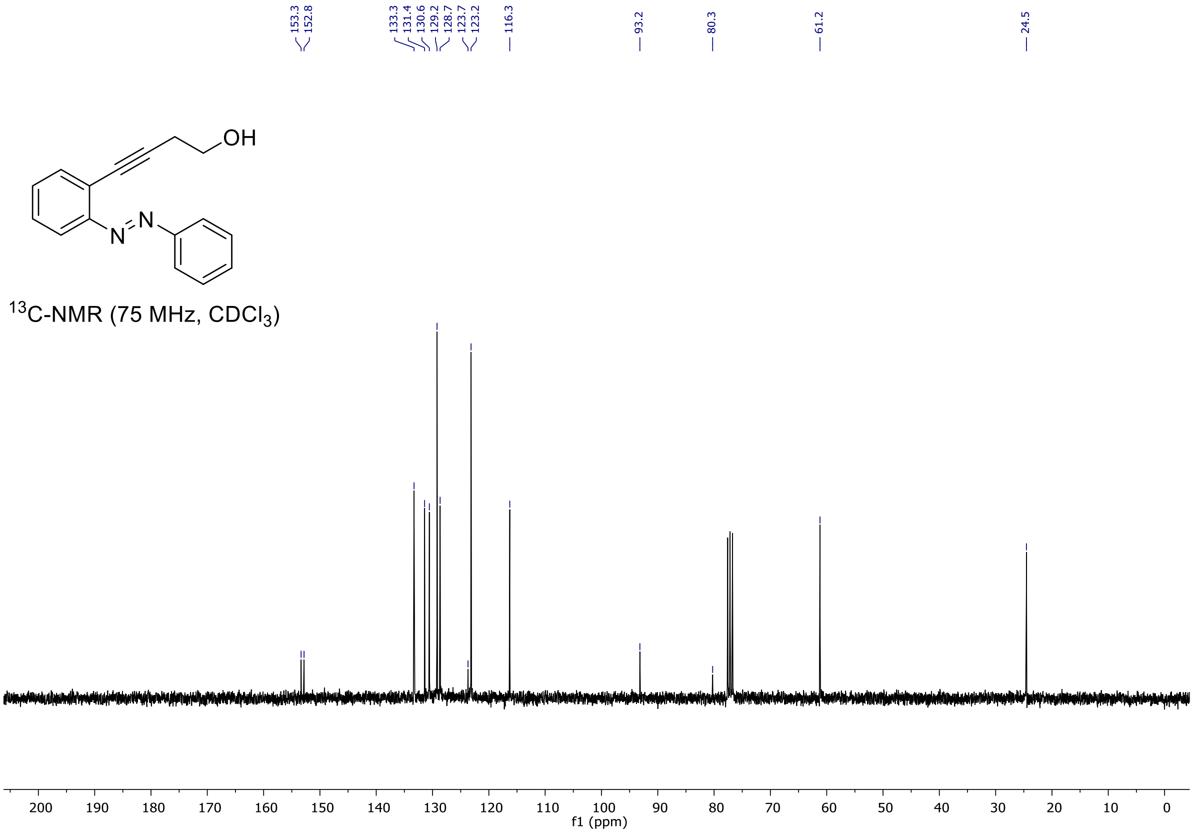

(*E*)-1-Phenyl-2-[2-(4-phenylbut-1-yn-1-yl)phenyl]diazene (1ac)

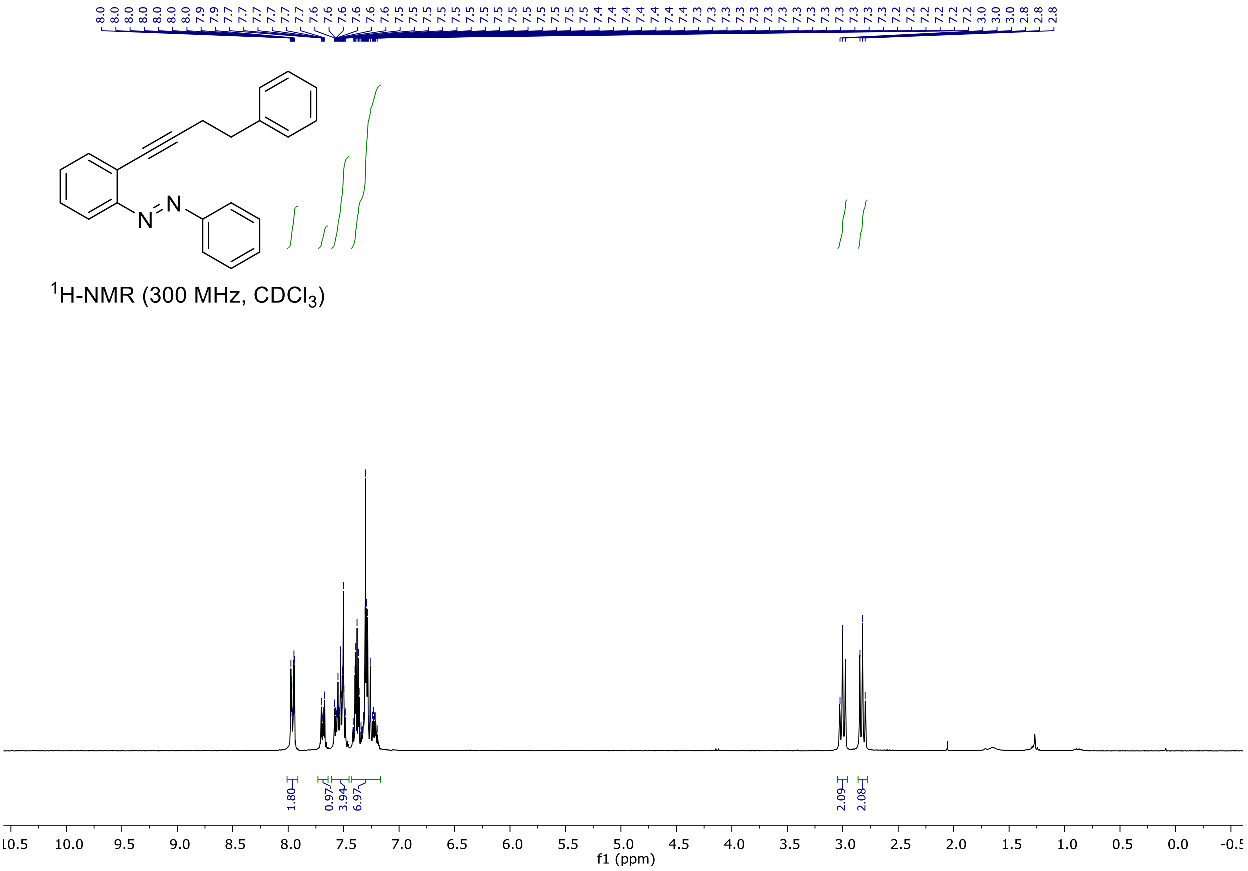

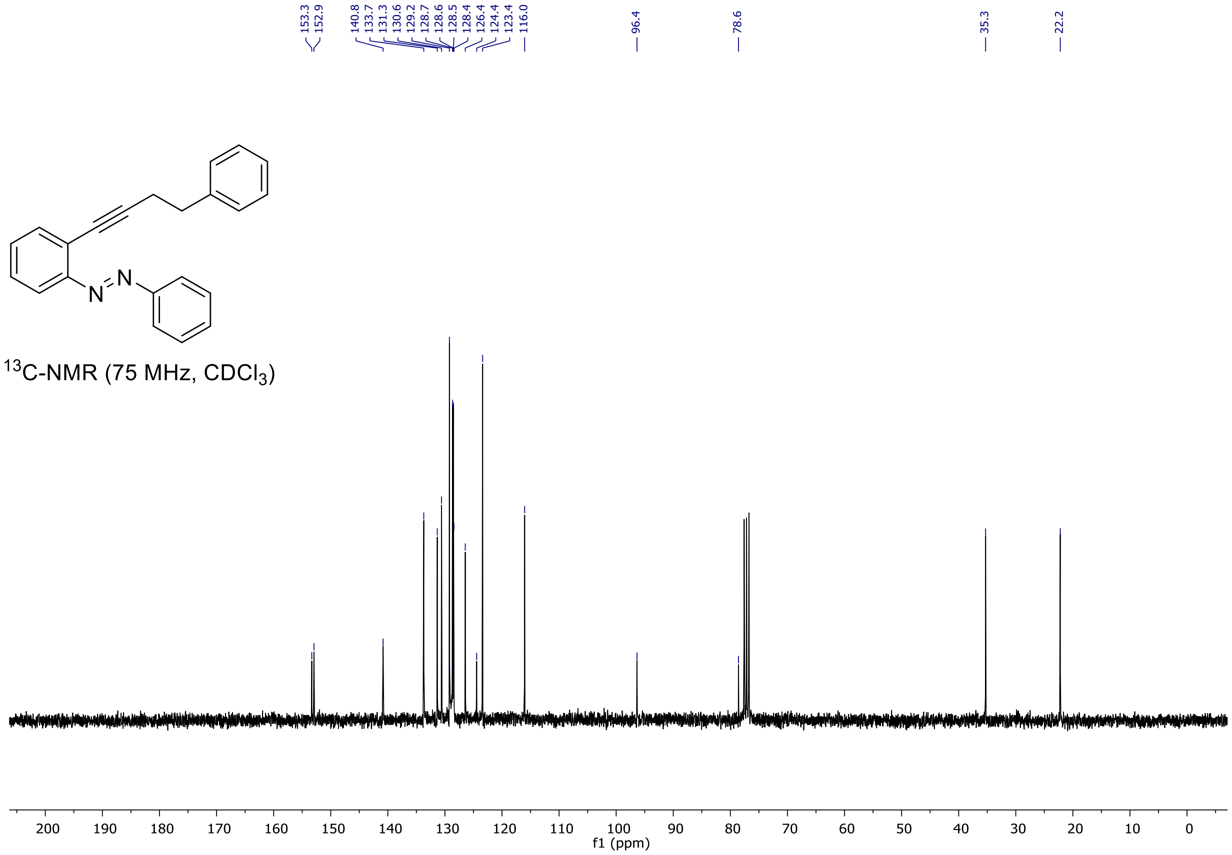

(*E*)-1-[2-((4-Methoxyphenyl)ethynyl)phenyl]-2-phenyldiazene (1ad)

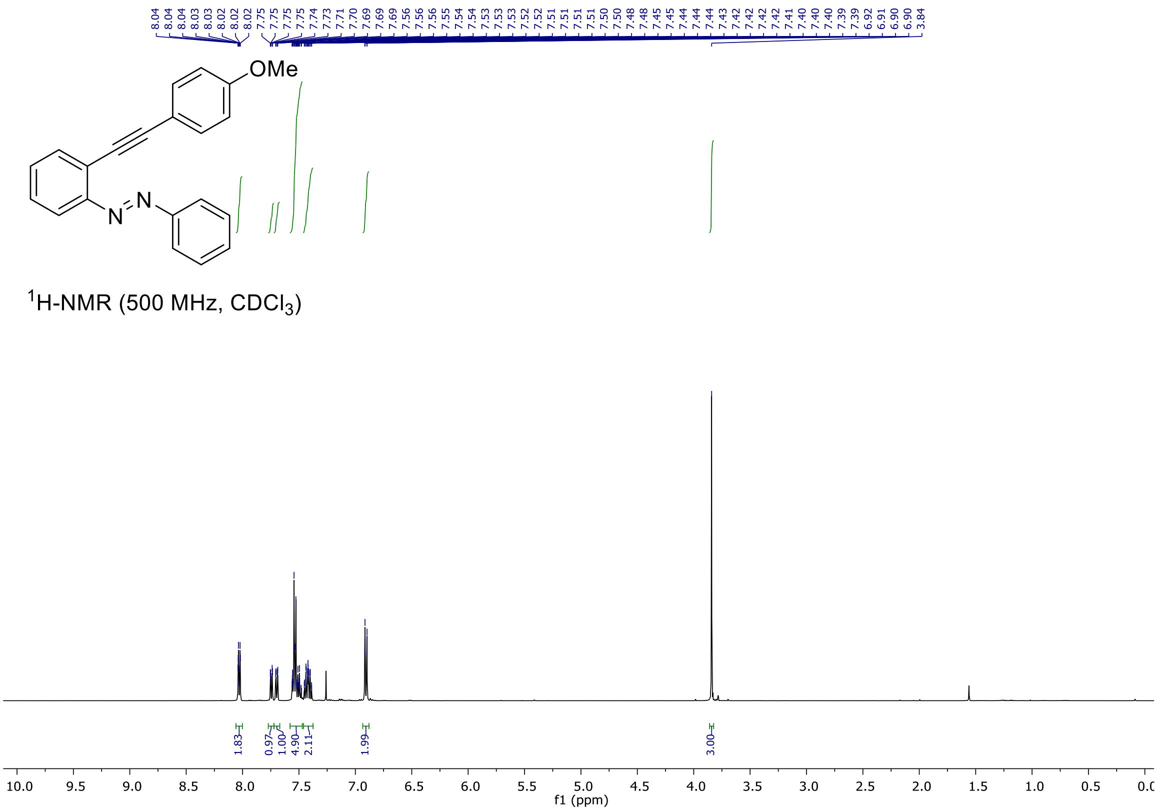

^{1}H-NMR (500 MHz, $CDCl_3$)

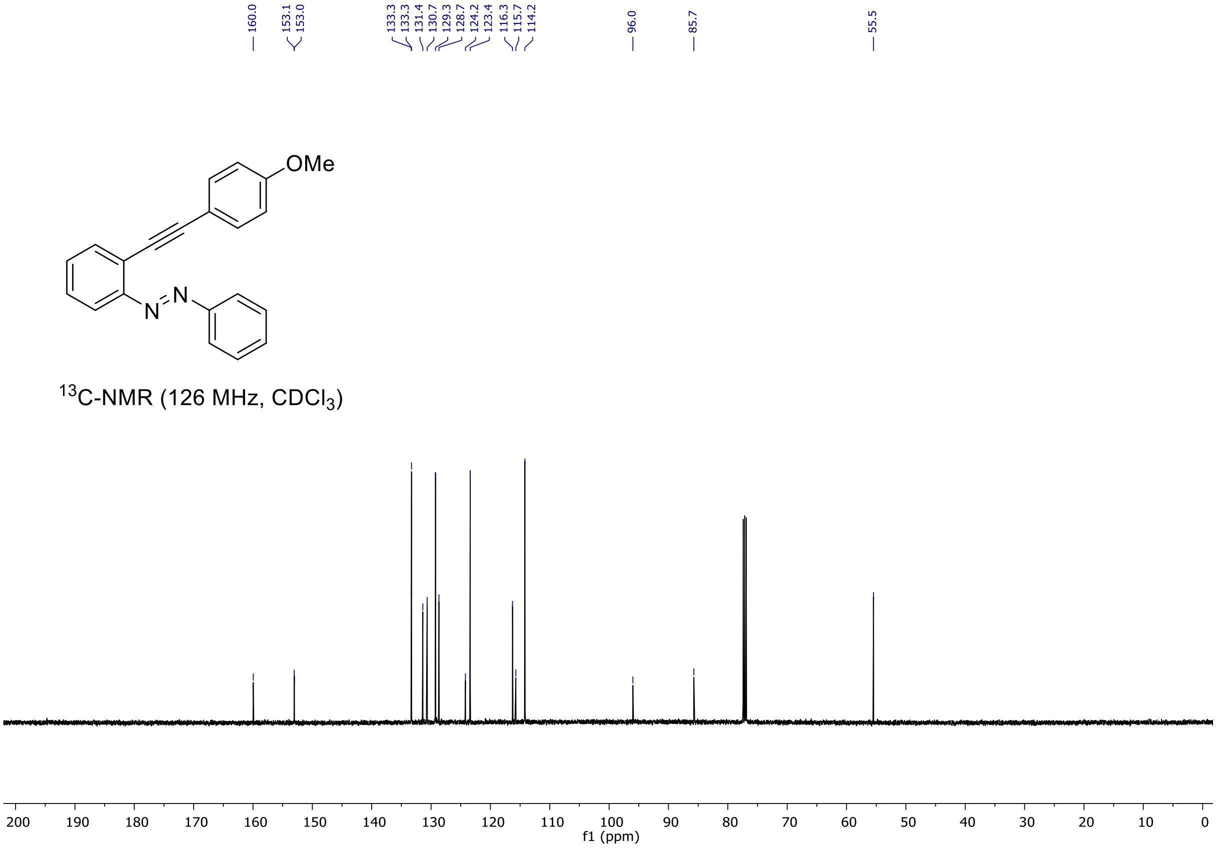

^{13}C-NMR (126 MHz, $CDCl_3$)

3-(1-Methoxypentyl)-2-phenyl-2*H*-indazole (2a)

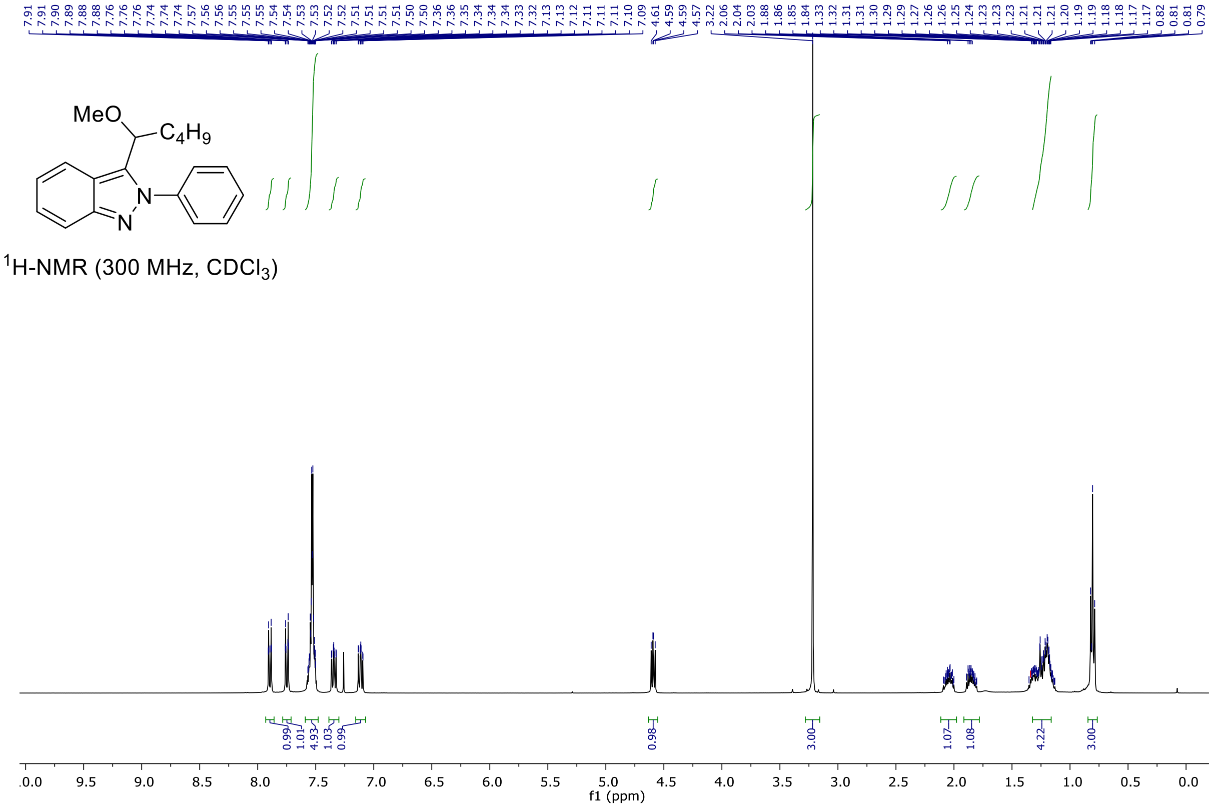

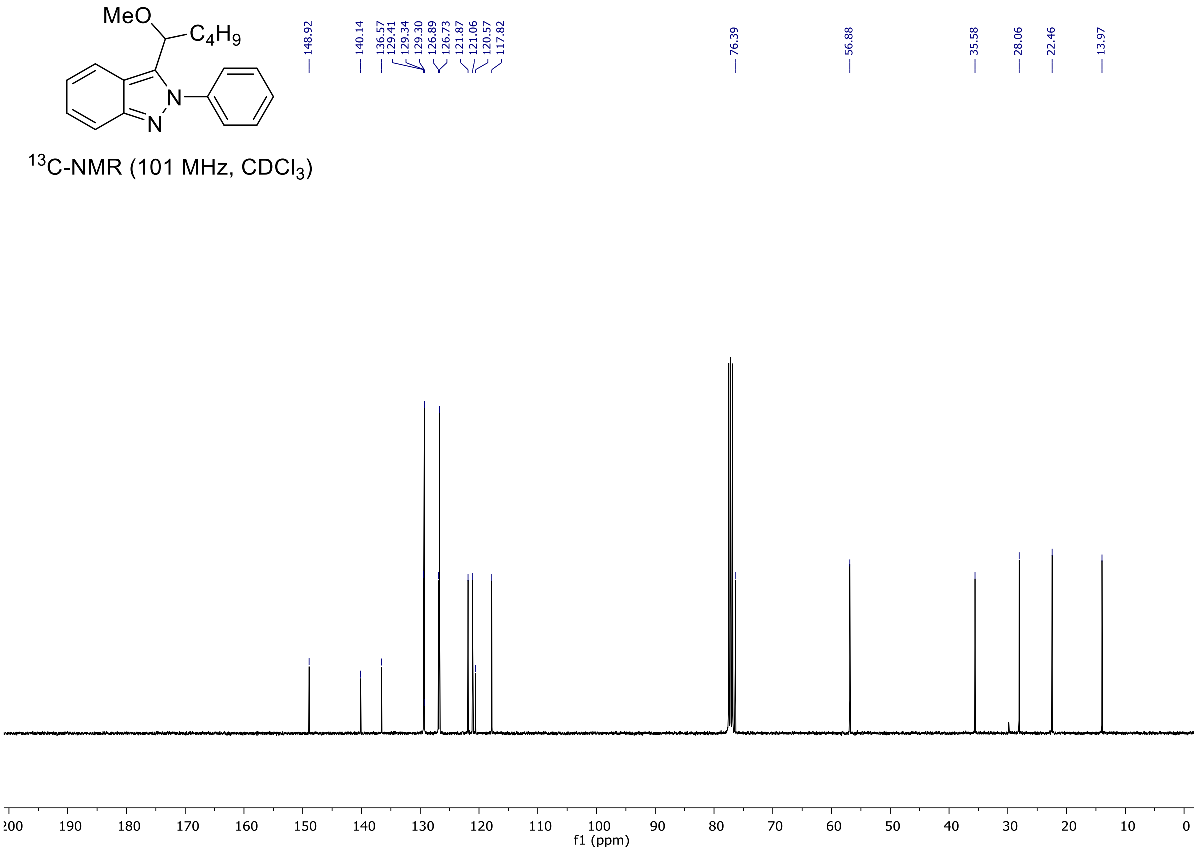

2-(4-Bromophenyl)-3-(1-methoxypentyl)-2*H*-indazole (2b)

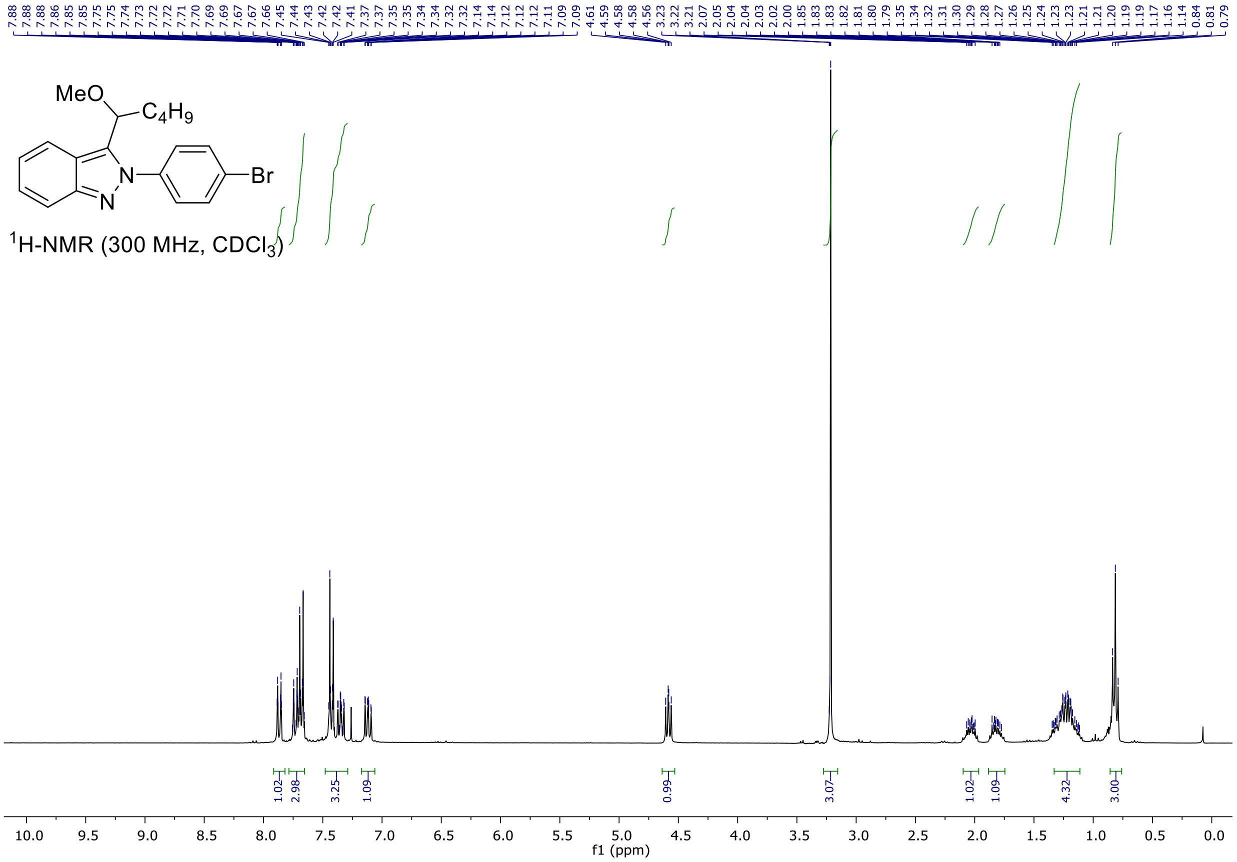

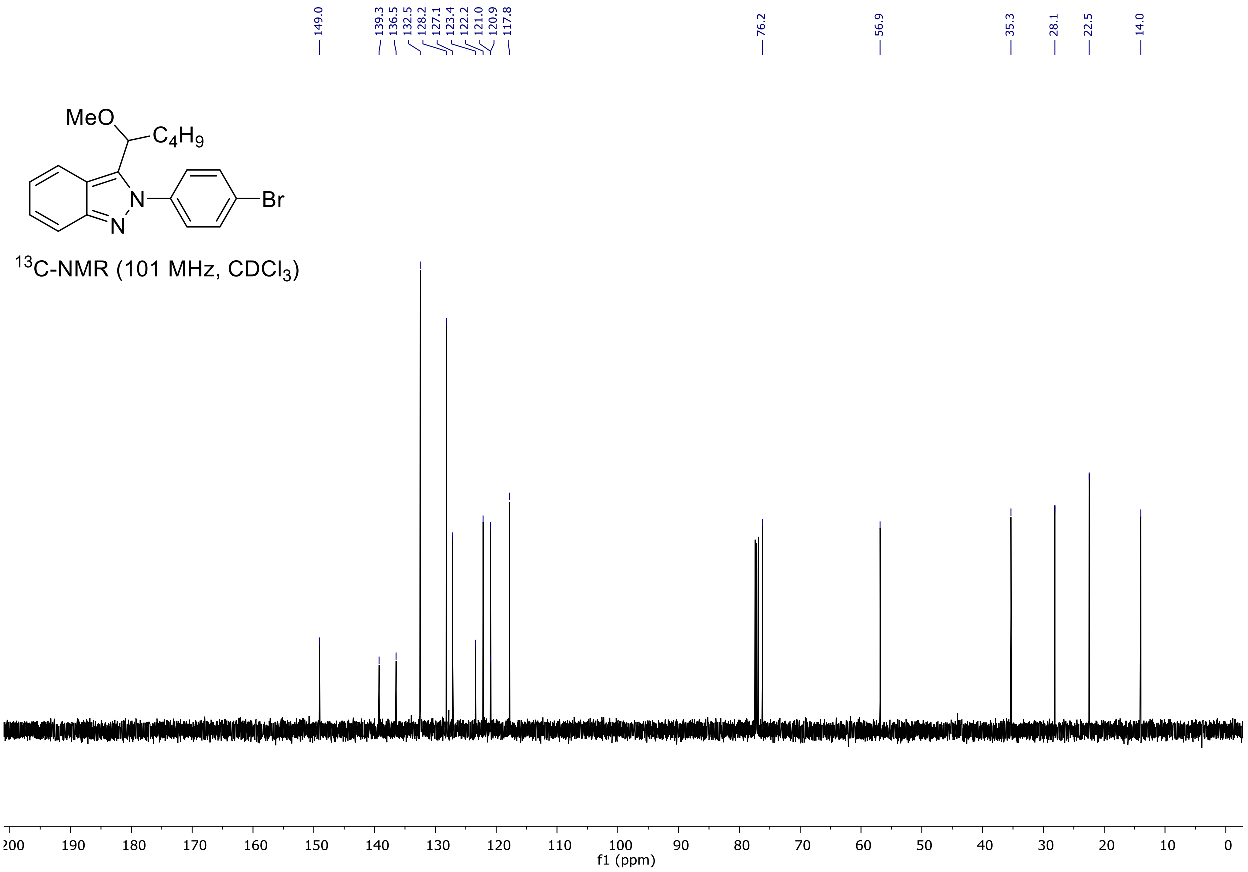

2-(3-Bromophenyl)-3-(1-methoxypentyl)-2*H*-indazole (2c)

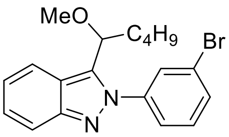

^{1}H-NMR (300 MHz, $CDCl_3$)

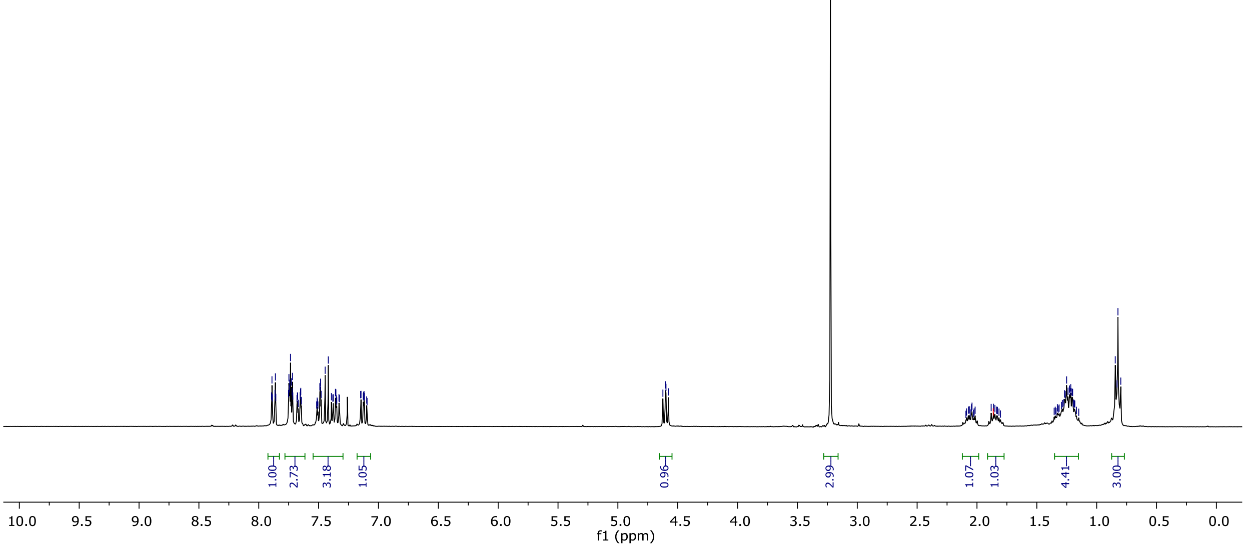

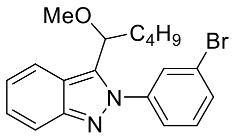

^{13}C-NMR (75 MHz, $CDCl_3$)

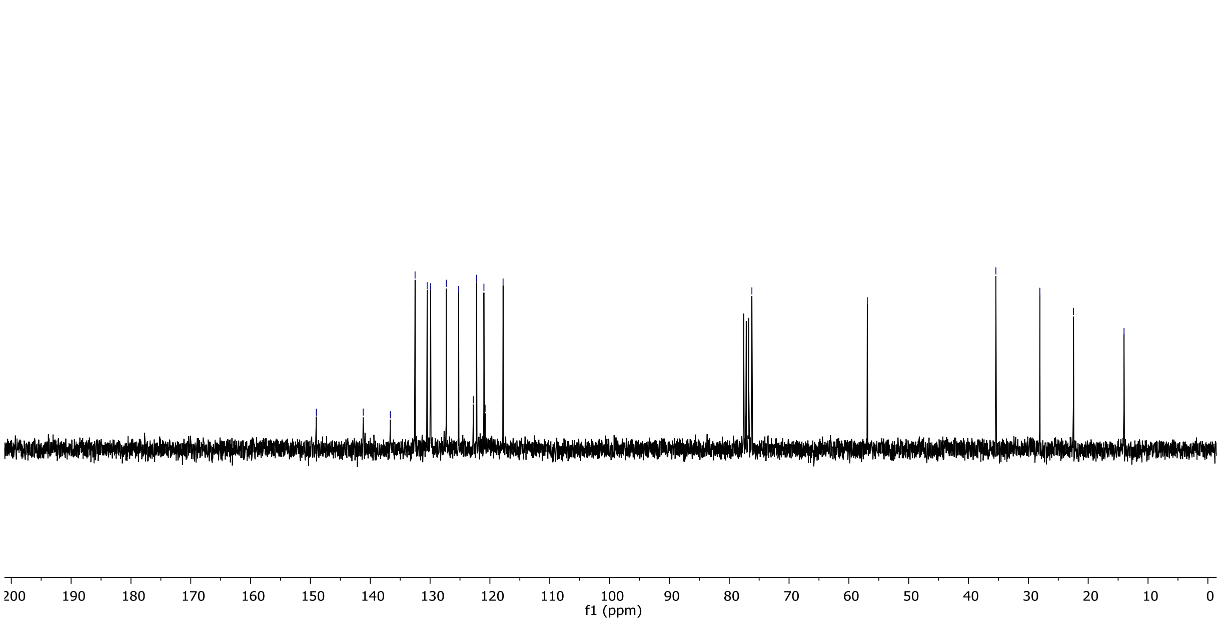

2-(3-Fluorophenyl)-3-(1-methoxypentyl)-2*H*-indazole (2d)

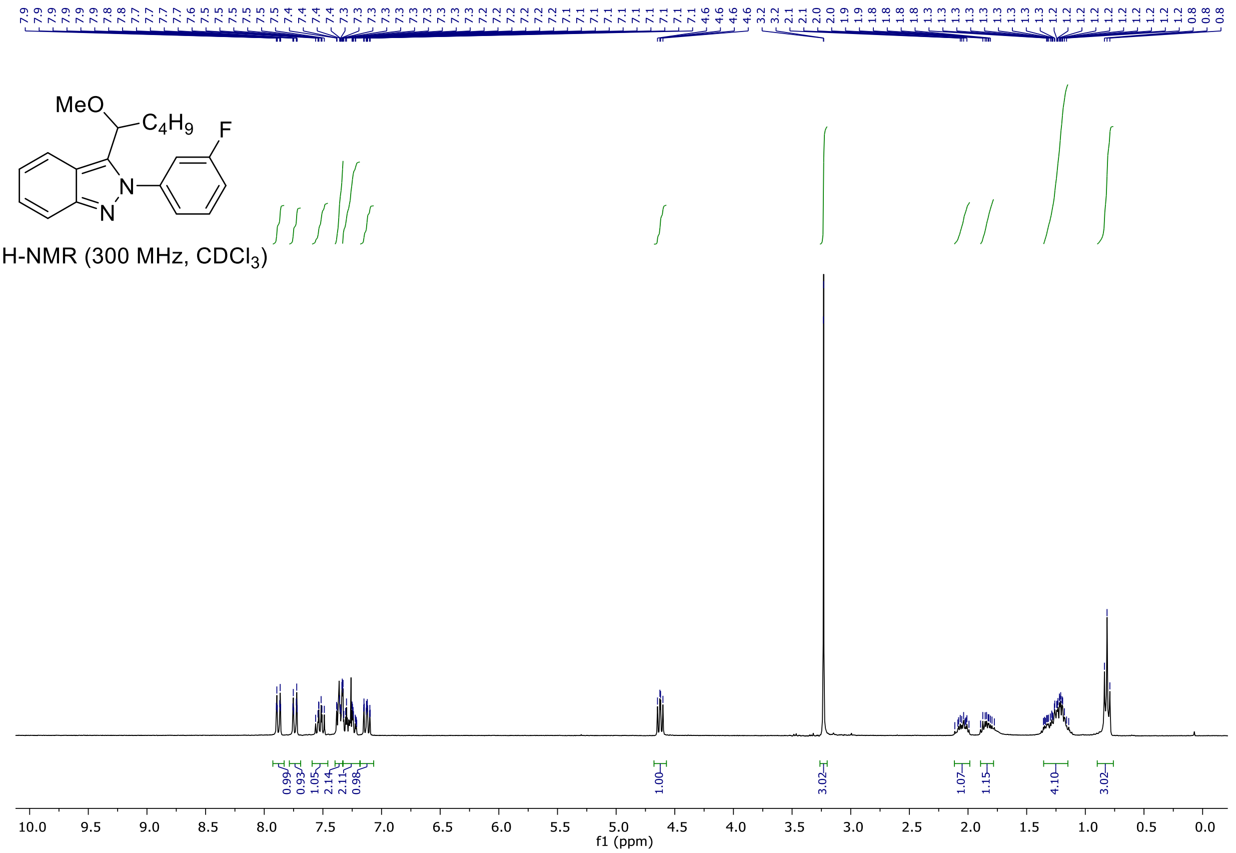

^{1}H-NMR (300 MHz, $CDCl_3$)

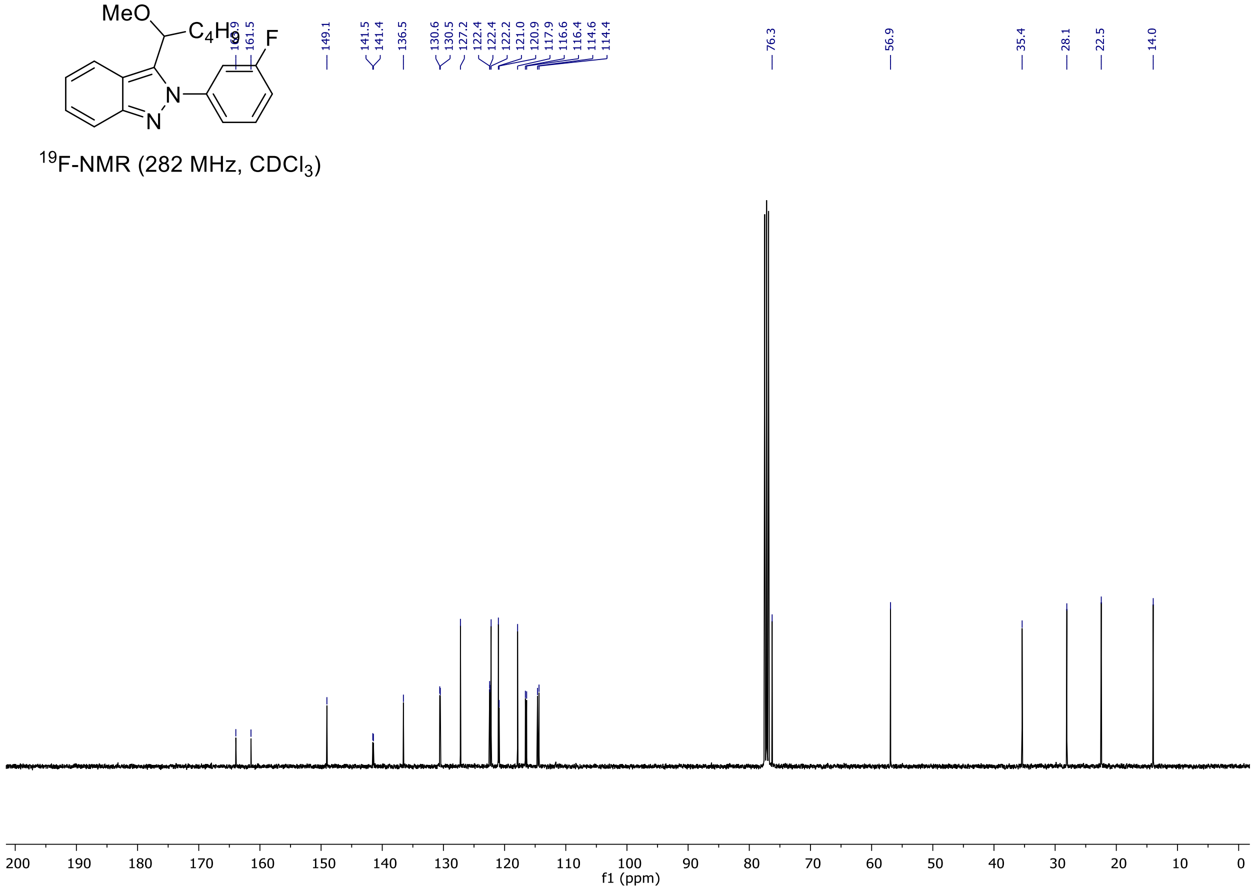

^{19}F-NMR (282 MHz, $CDCl_3$)

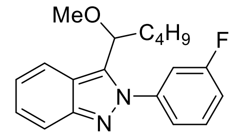

^{13}C-NMR (101 MHz, $CDCl_3$)

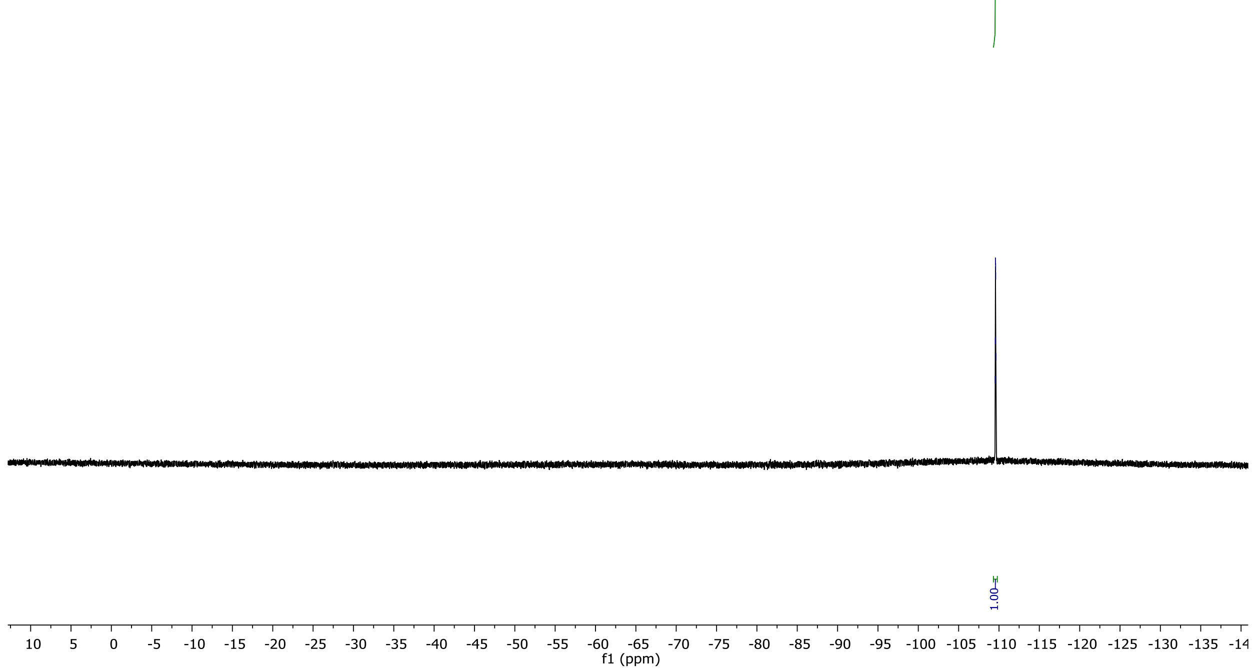

2-(2-Fluorophenyl)-3-(1-methoxypentyl)-2*H*-indazole (2e)

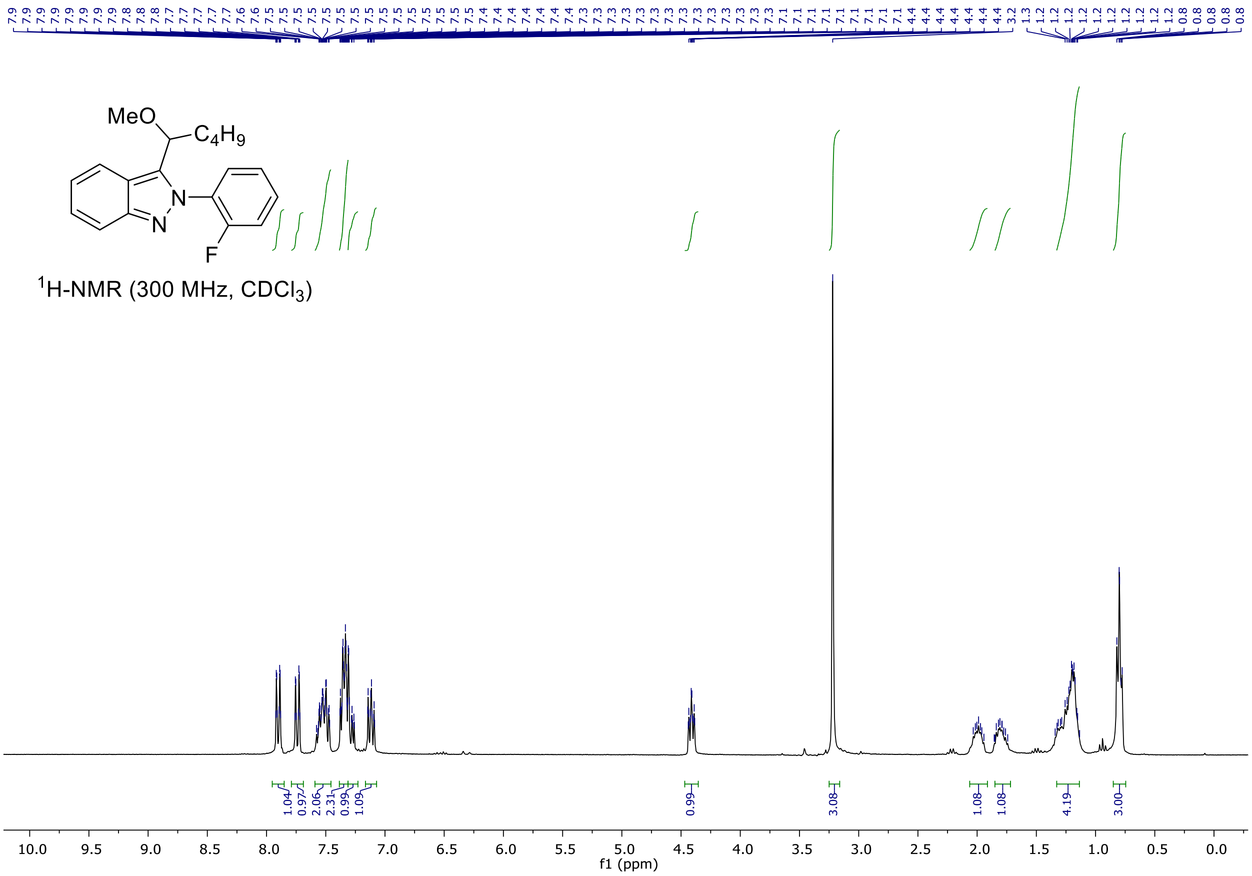

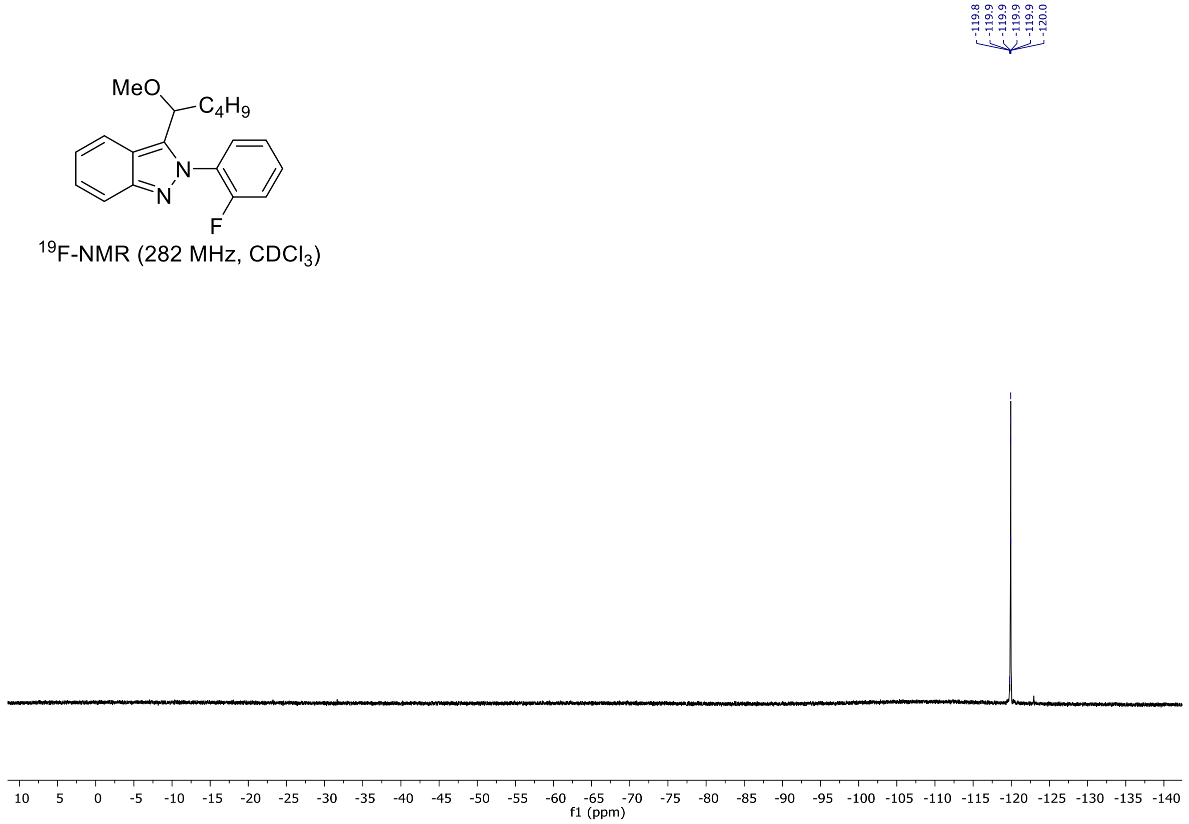

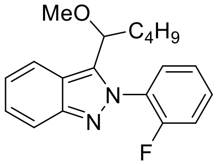

^{13}C-NMR (75 MHz, $CDCl_3$)

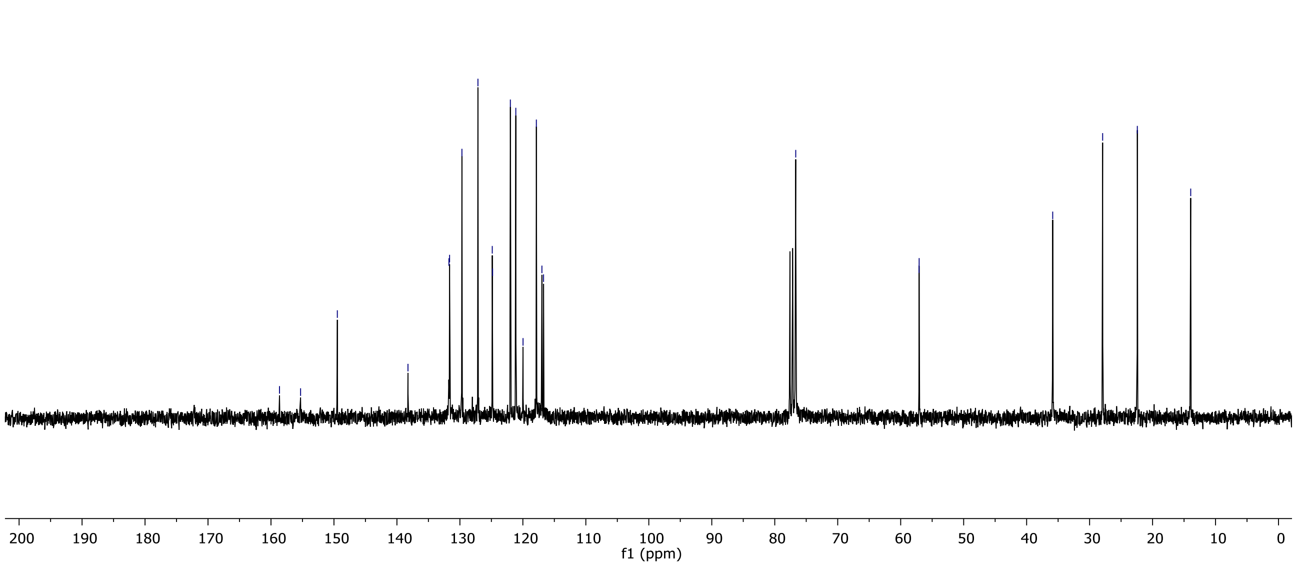

4-[3-(1-Methoxypentyl)-2*H*-indazol-2-yl]benzonitrile (2f)

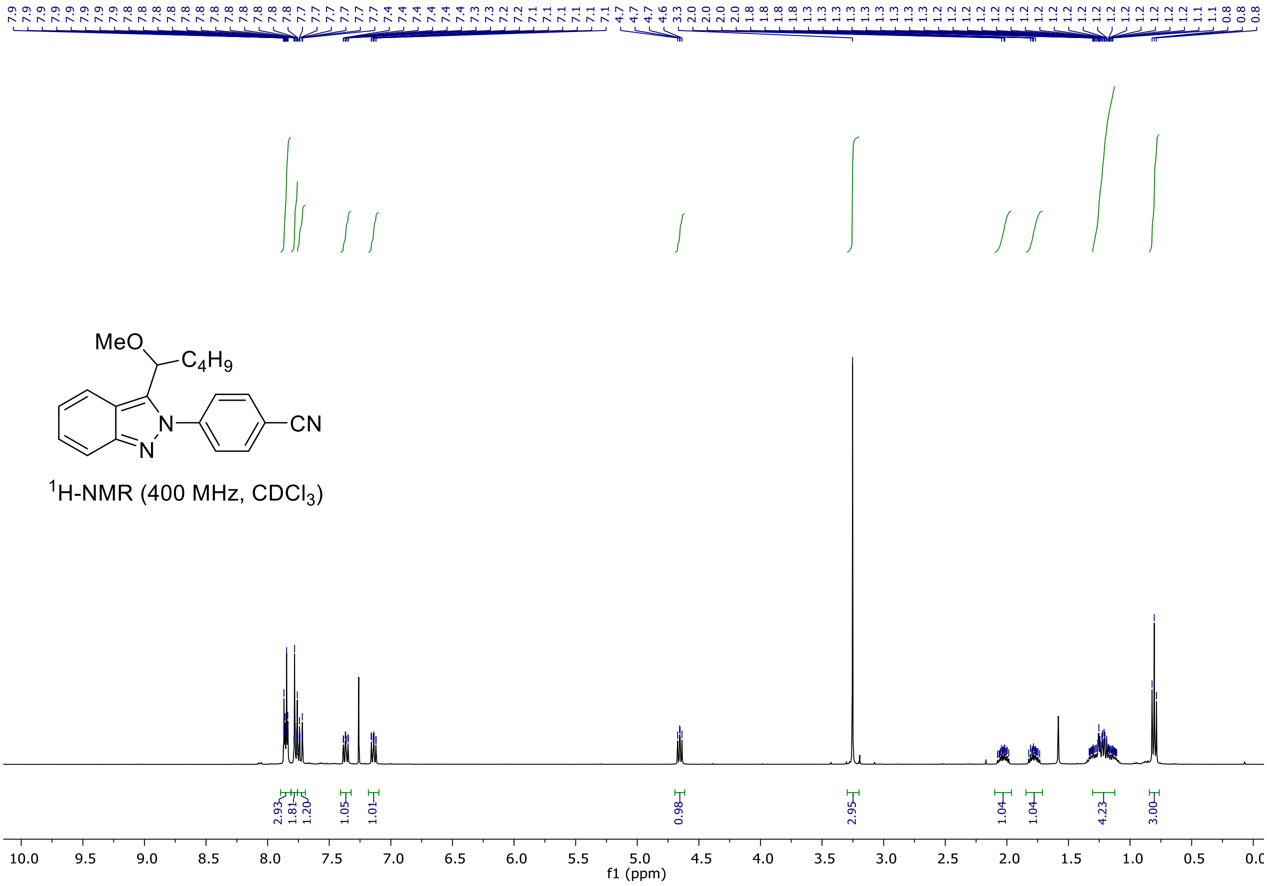

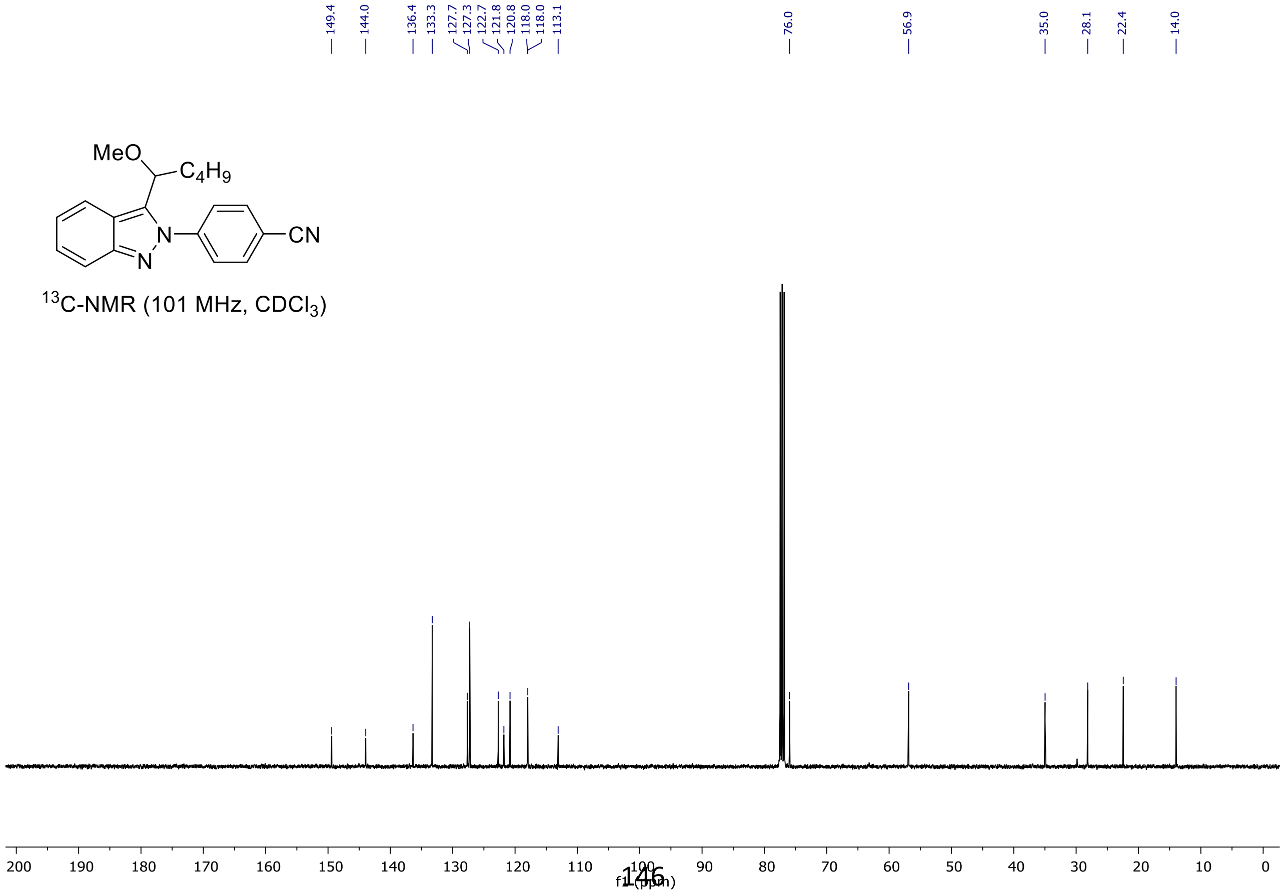

3-(1-Methoxypentyl)-2-(3-nitrophenyl)-2*H*-indazole (2g)

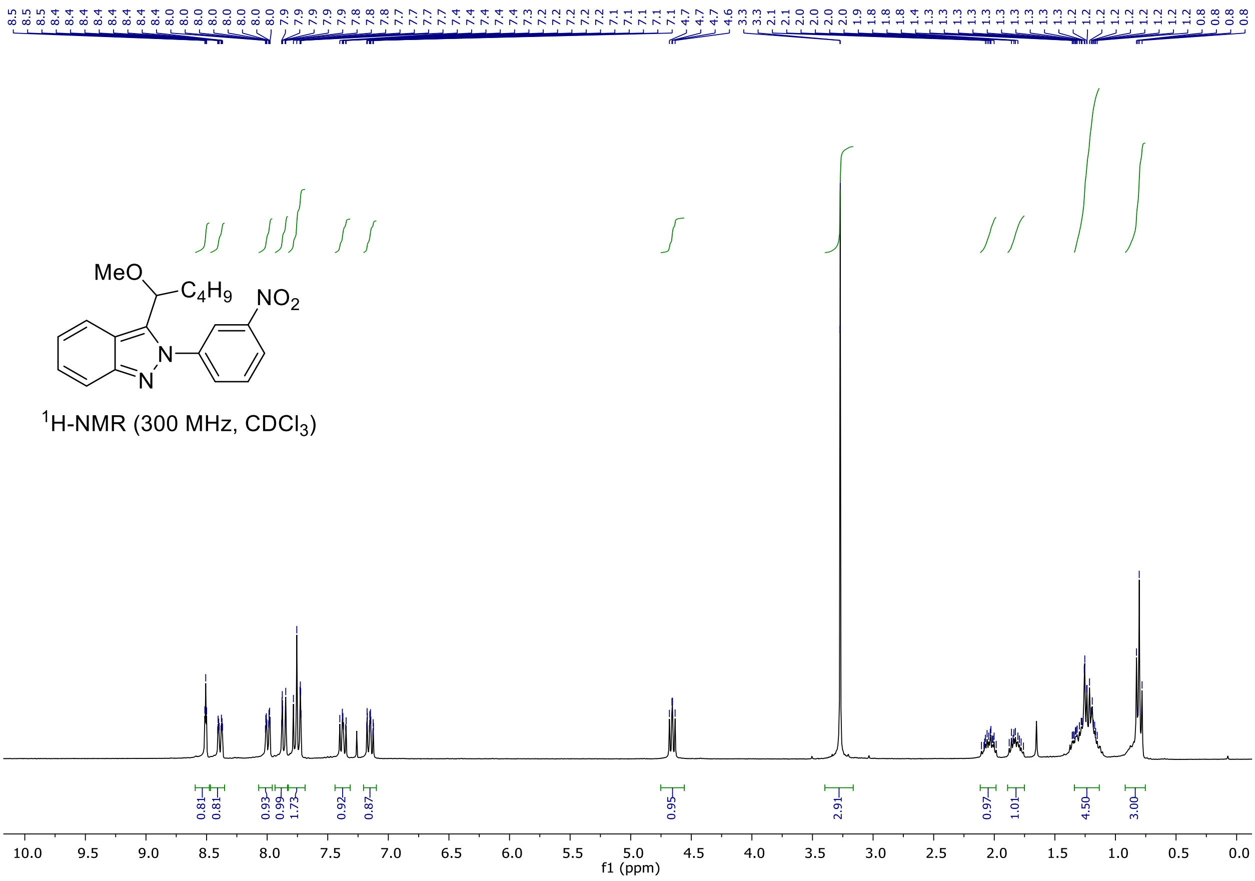

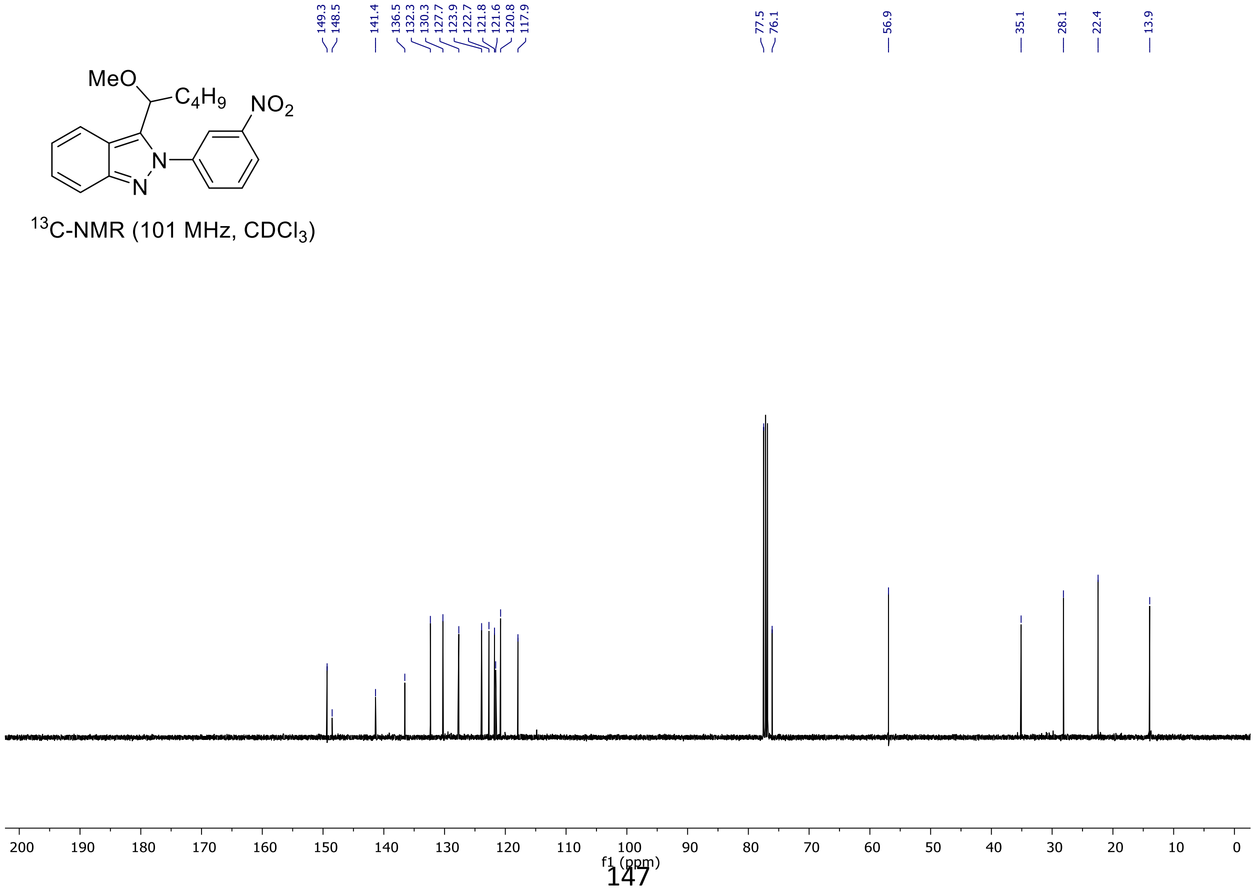

1-[4-(3-(1-Methoxypentyl)-2*H*-indazol-2-yl)phenyl]ethan-1-one (2h)

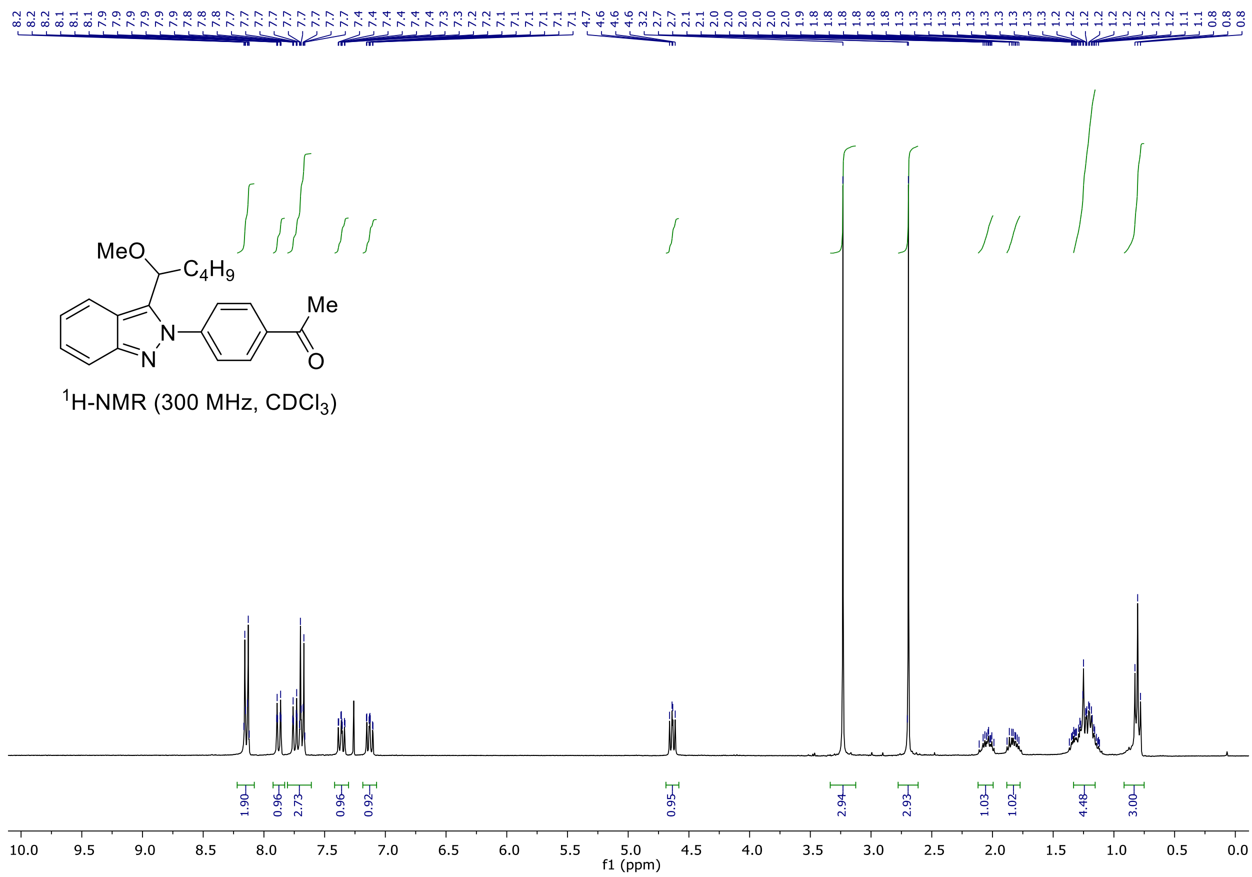

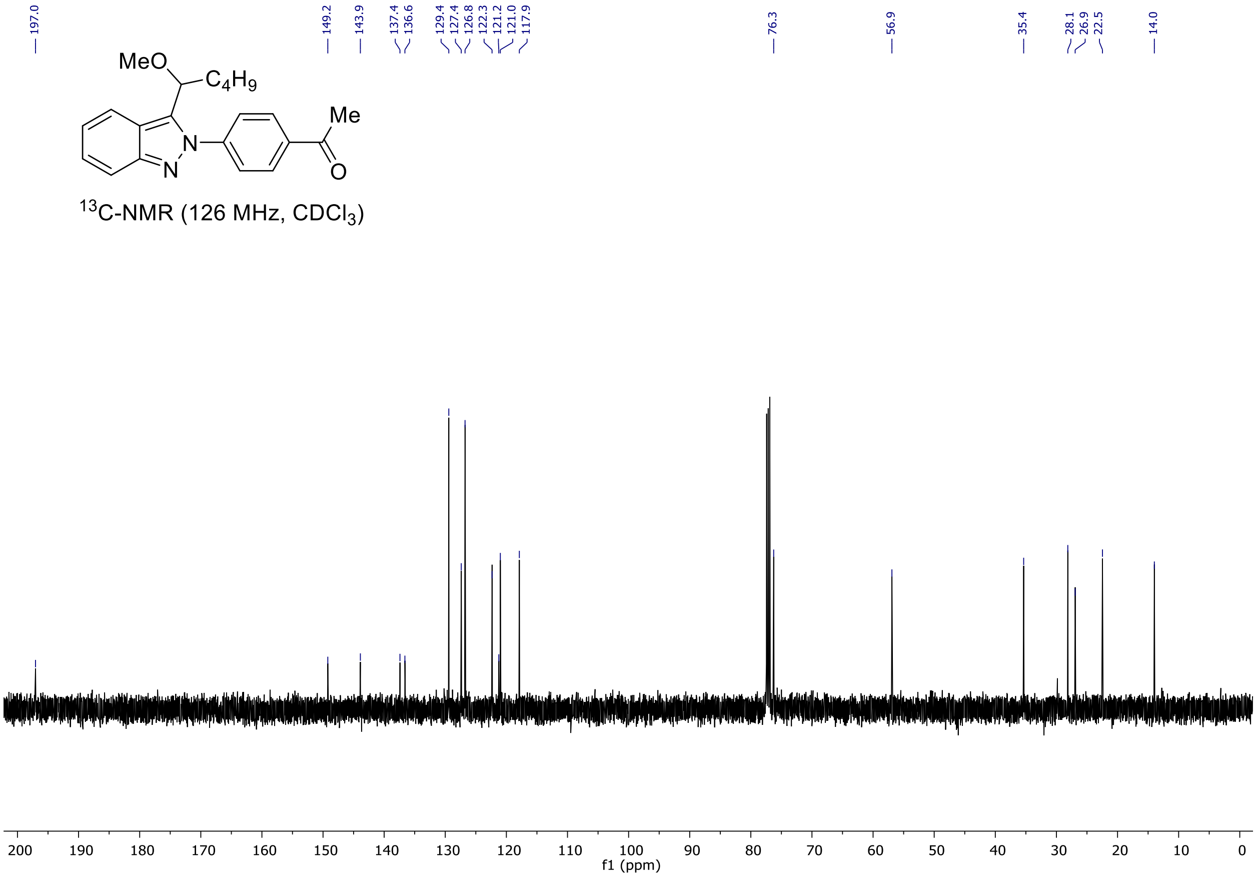

Methyl 4-[3-(1-methoxypentyl)-2*H*-indazol-2-yl]benzoate (2i)

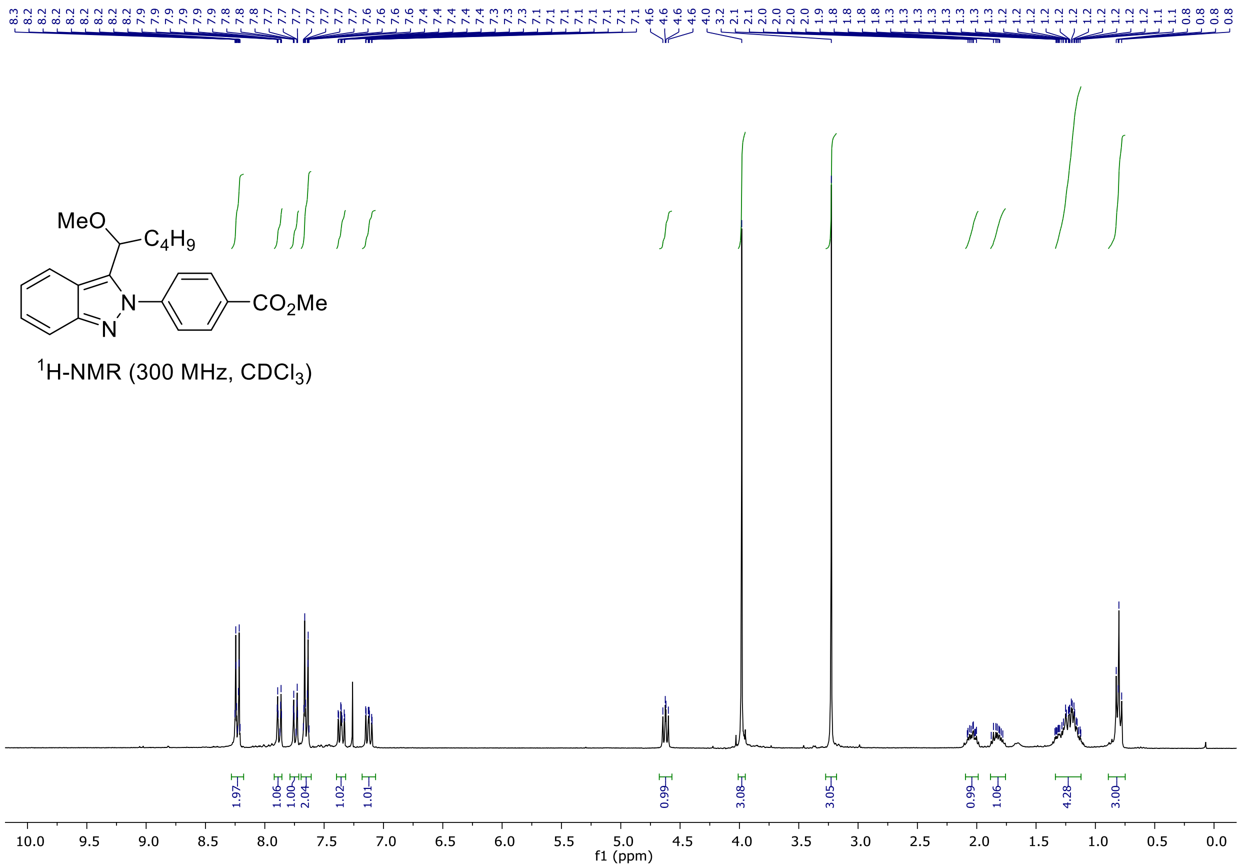

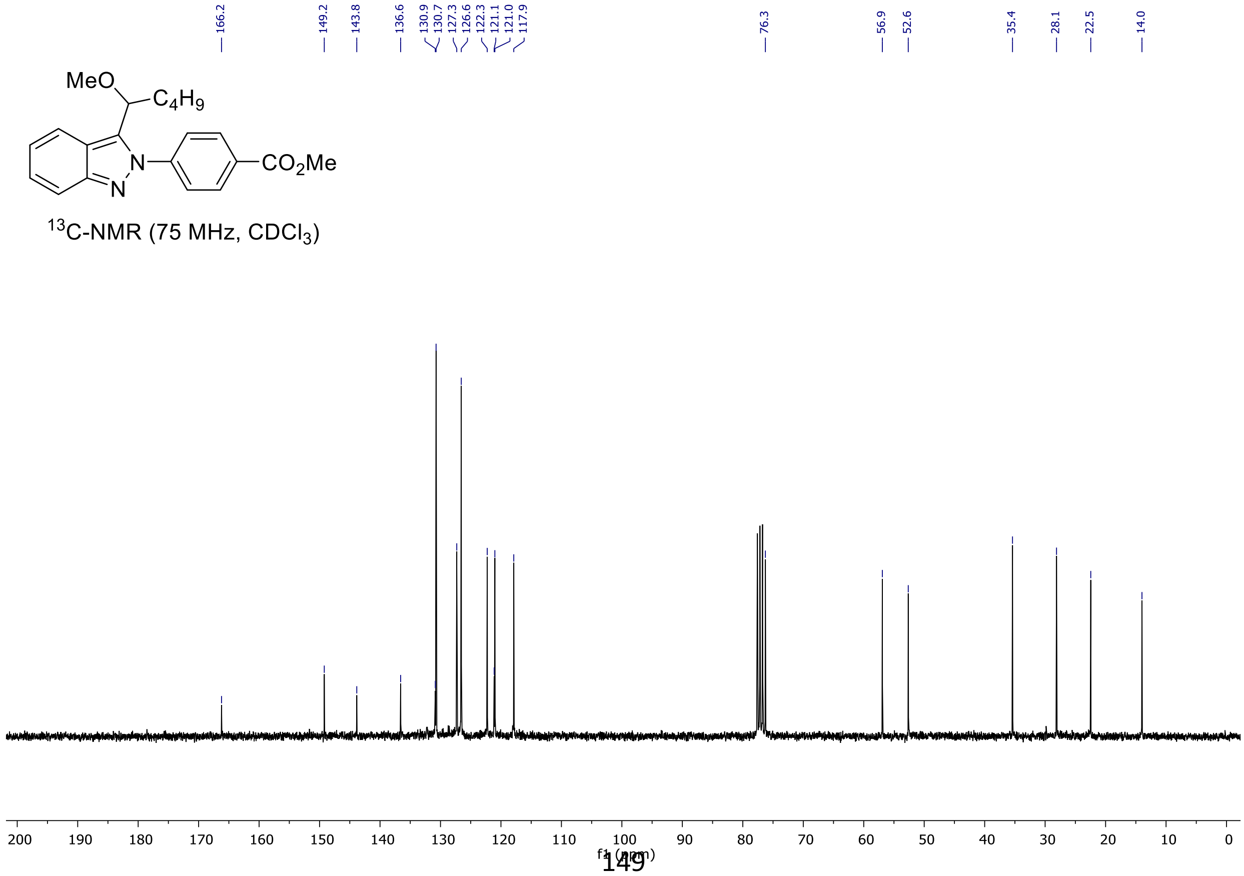

2-(2-Bromo-4-methylphenyl)-3-(1-methoxypentyl)-2*H*-indazole (2j)

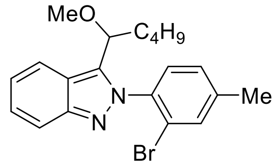

^{1}H-NMR (300 MHz, $CDCl_3$)

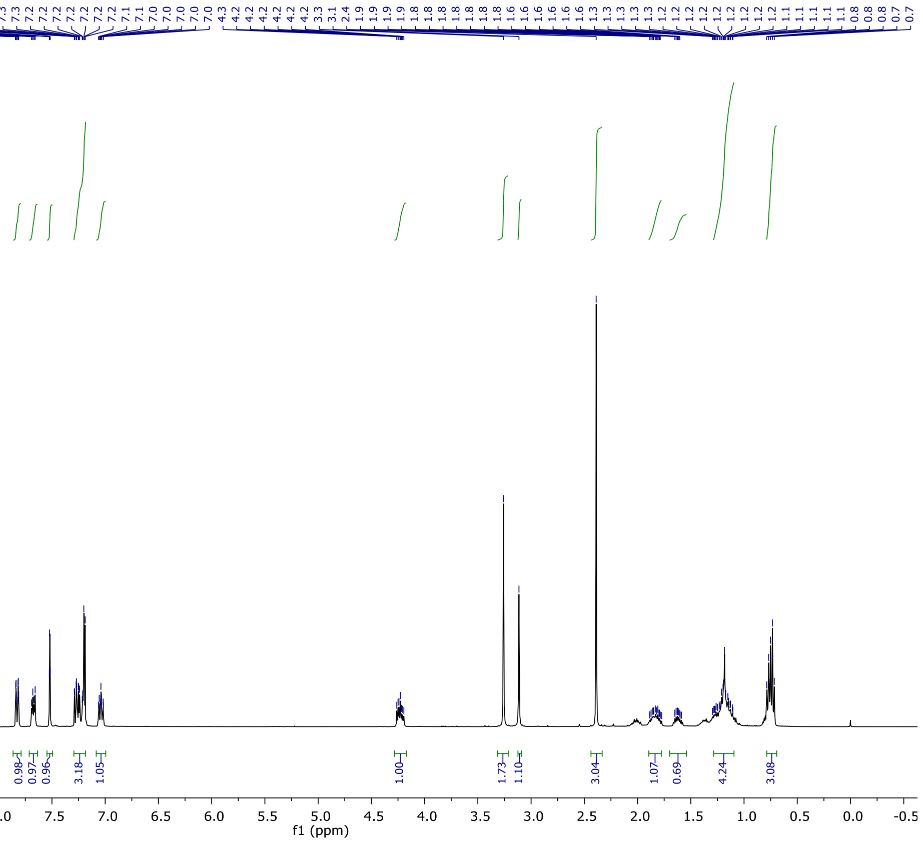

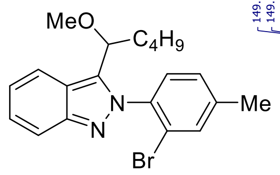

^{13}C-NMR (101 MHz, $CDCl_3$)

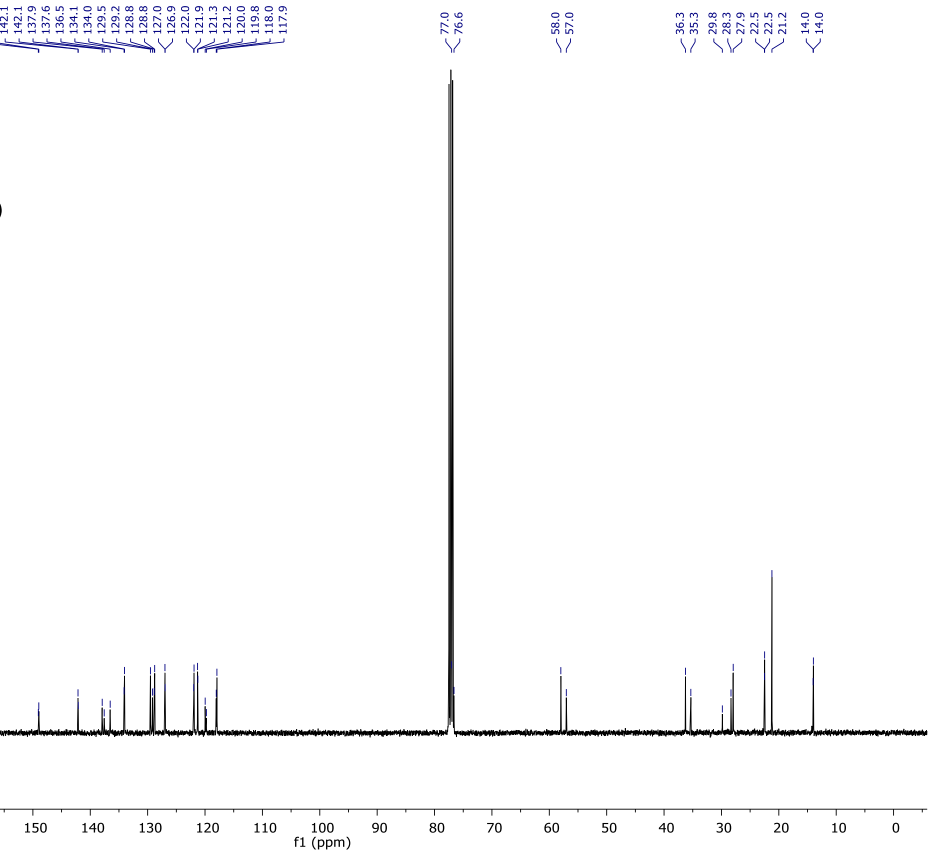

Dimethyl 5-[3-(1-methoxypentyl)-2*H*-indazol-2-yl]isophthalate (2k)

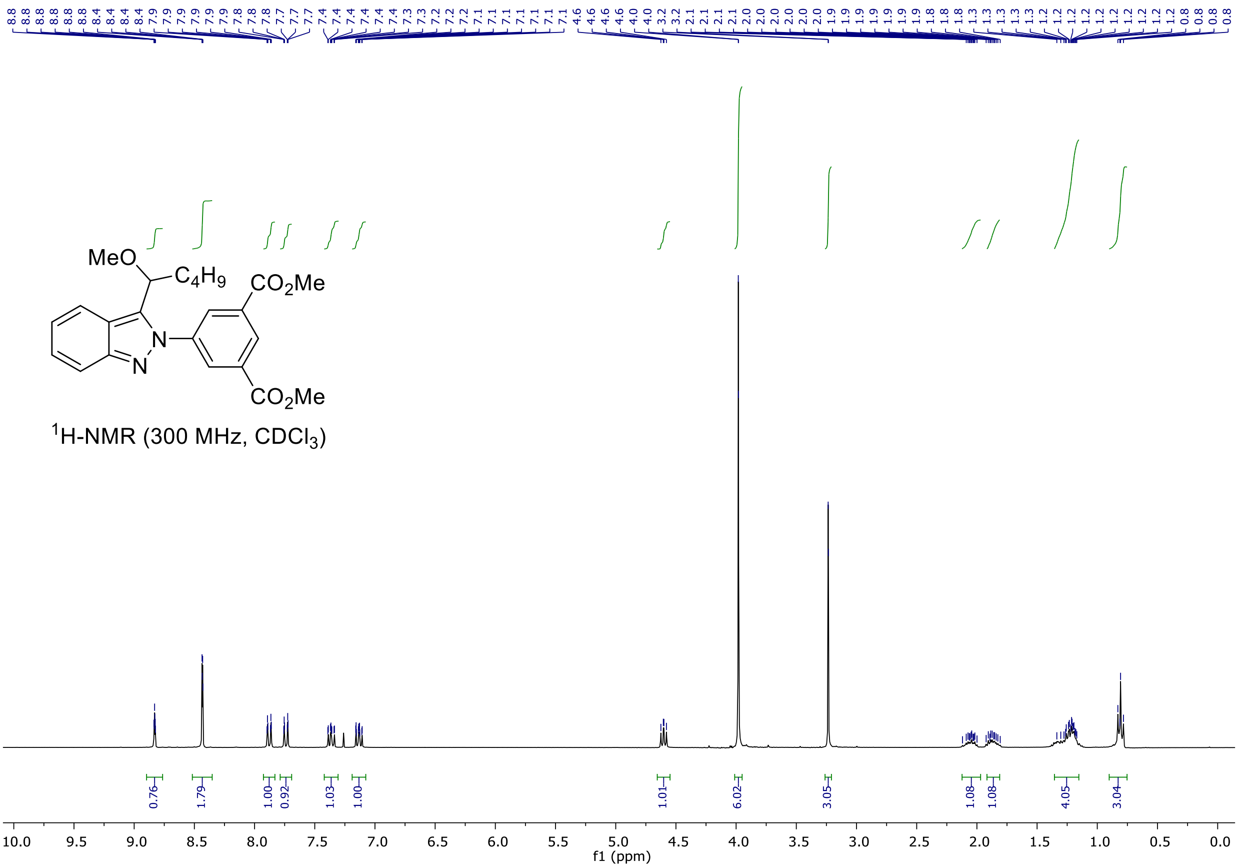

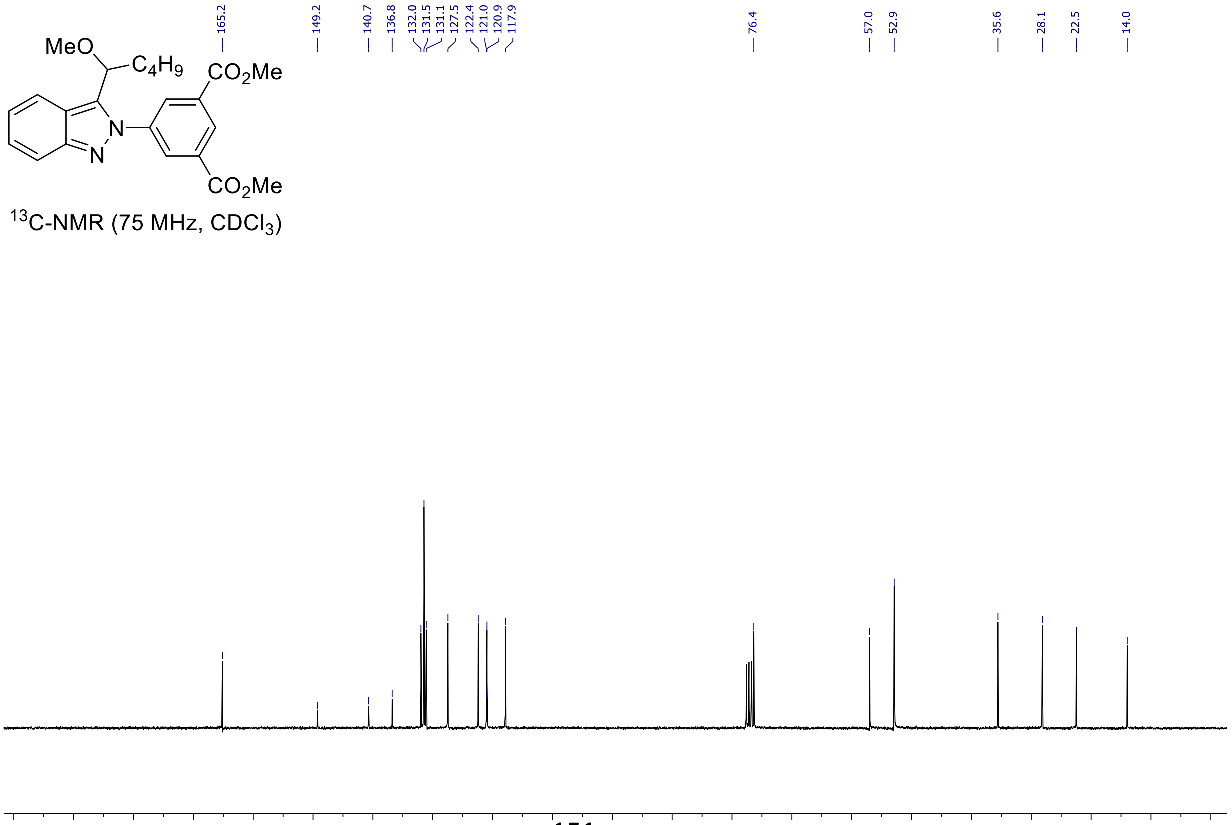

3-(1-Methoxypentyl)-2-(perfluorophenyl)-2*H*-indazole (2l)

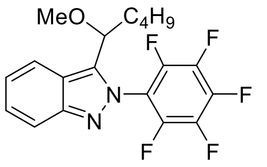

^{1}H-NMR (300 MHz, $CDCl_3$)

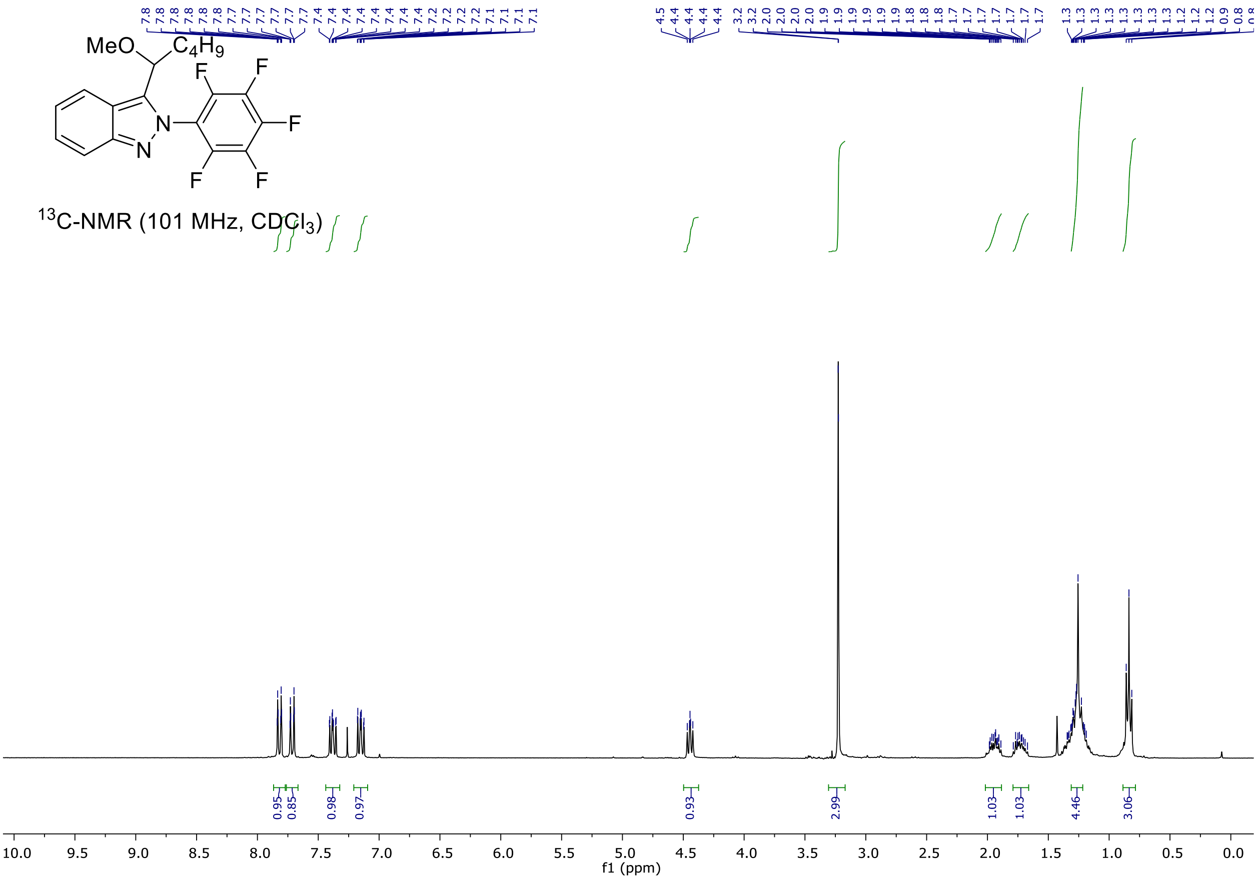
MeO
C4H9
F
N
13C-NMR (101 MHz, CDCl3)
f1 (ppm)

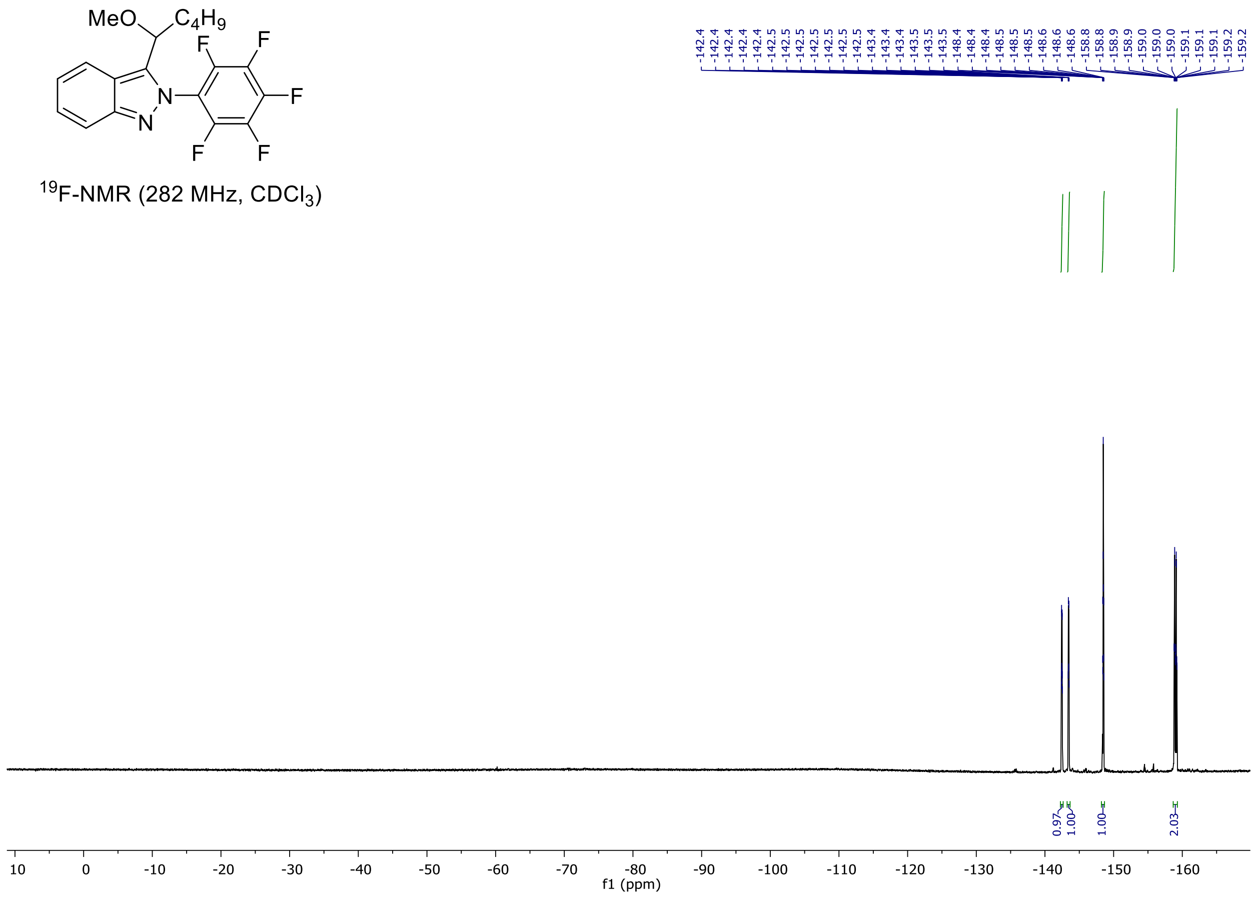
MeO
C4H9
F
N
19F-NMR (282 MHz, CDCl3)
f1 (ppm)

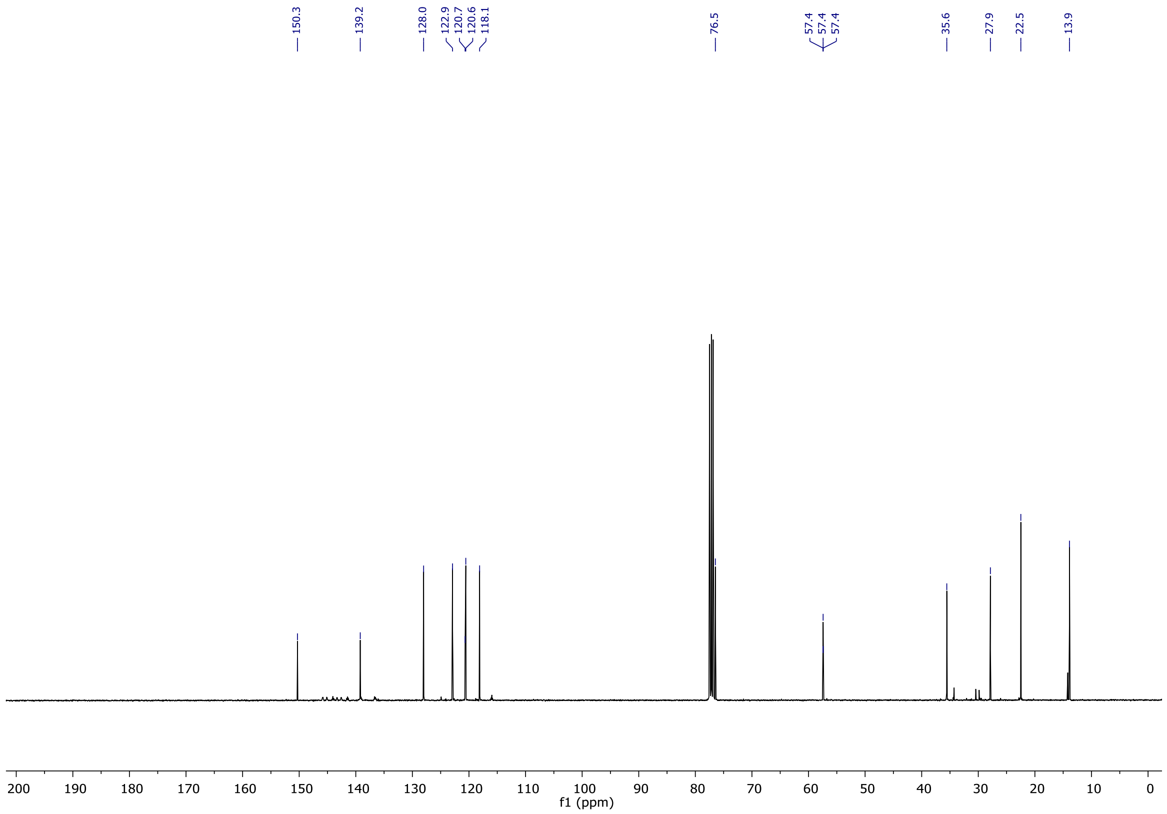
150.3
139.2
128.0
122.9
120.7
120.6
118.1
76.5
57.4
57.4
57.4
35.6
27.9
22.5
13.9
200 190 180 170 160 150 140 130 120 110 100 90 80 70 60 50 40 30 20 10 0
f1 (ppm)

3-(1-Methoxypentyl)-2-(*p*-tolyl)-2*H*-indazole (2m)

^{1}H-NMR (300 MHz, $CDCl_3$)

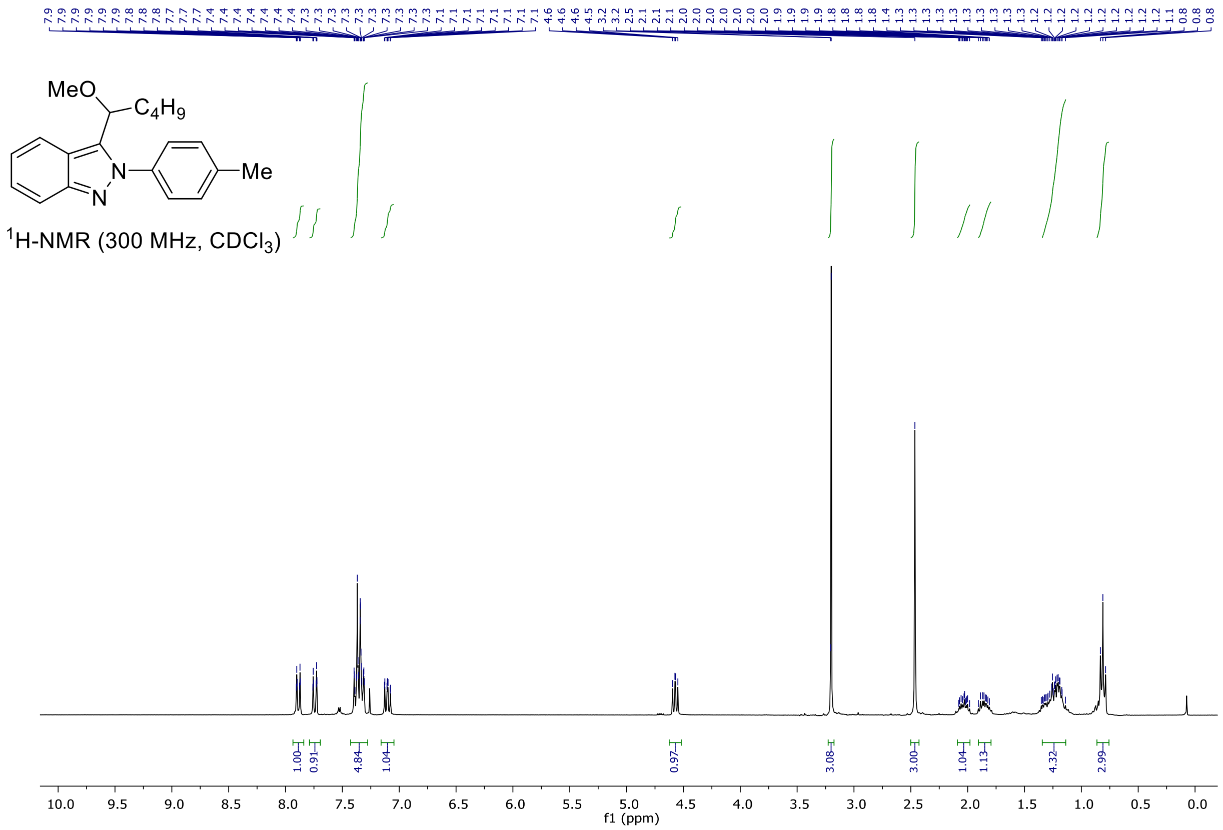

^{13}C-NMR (101 MHz, $CDCl_3$)

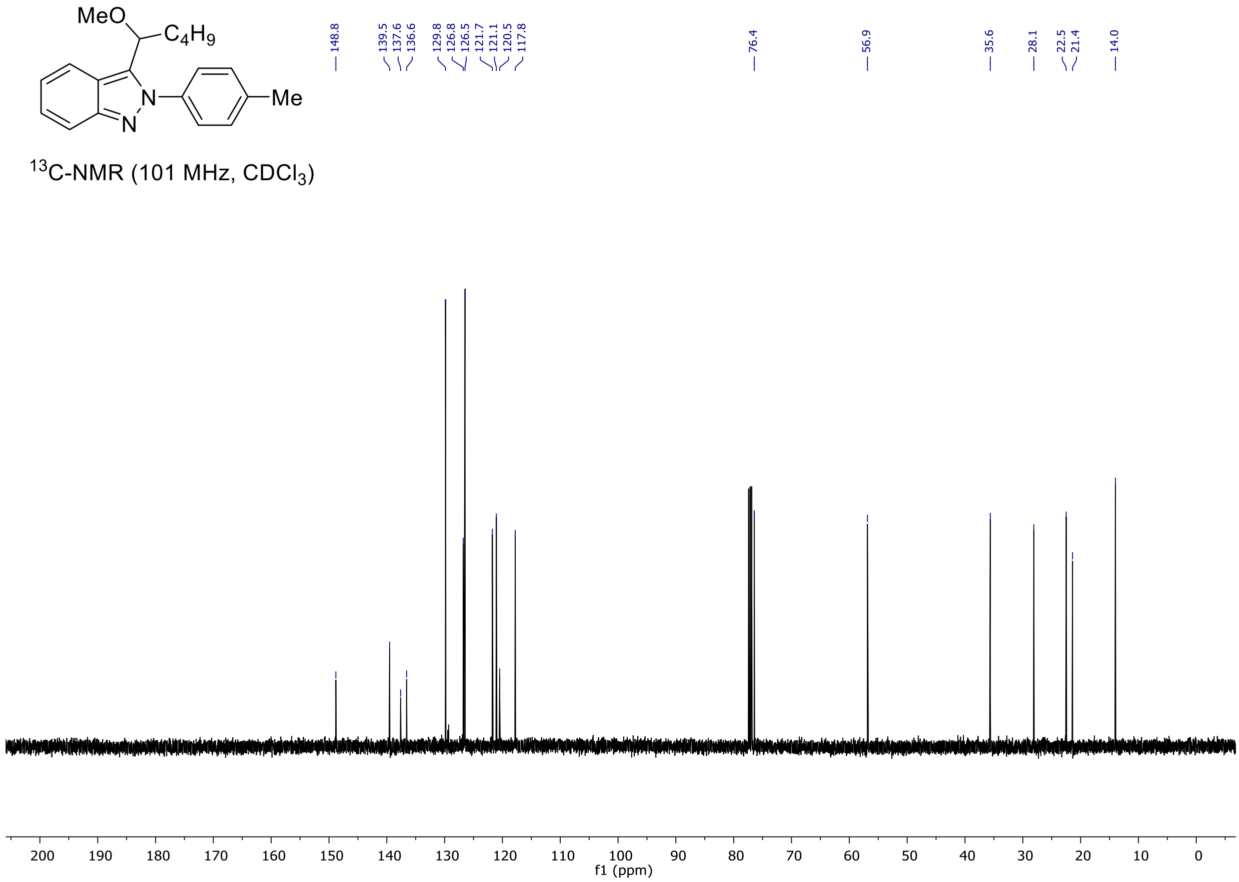

4-[3-(1-Methoxypentyl)-2*H*-indazol-2-yl]-*N,N*-dimethylaniline (2n)

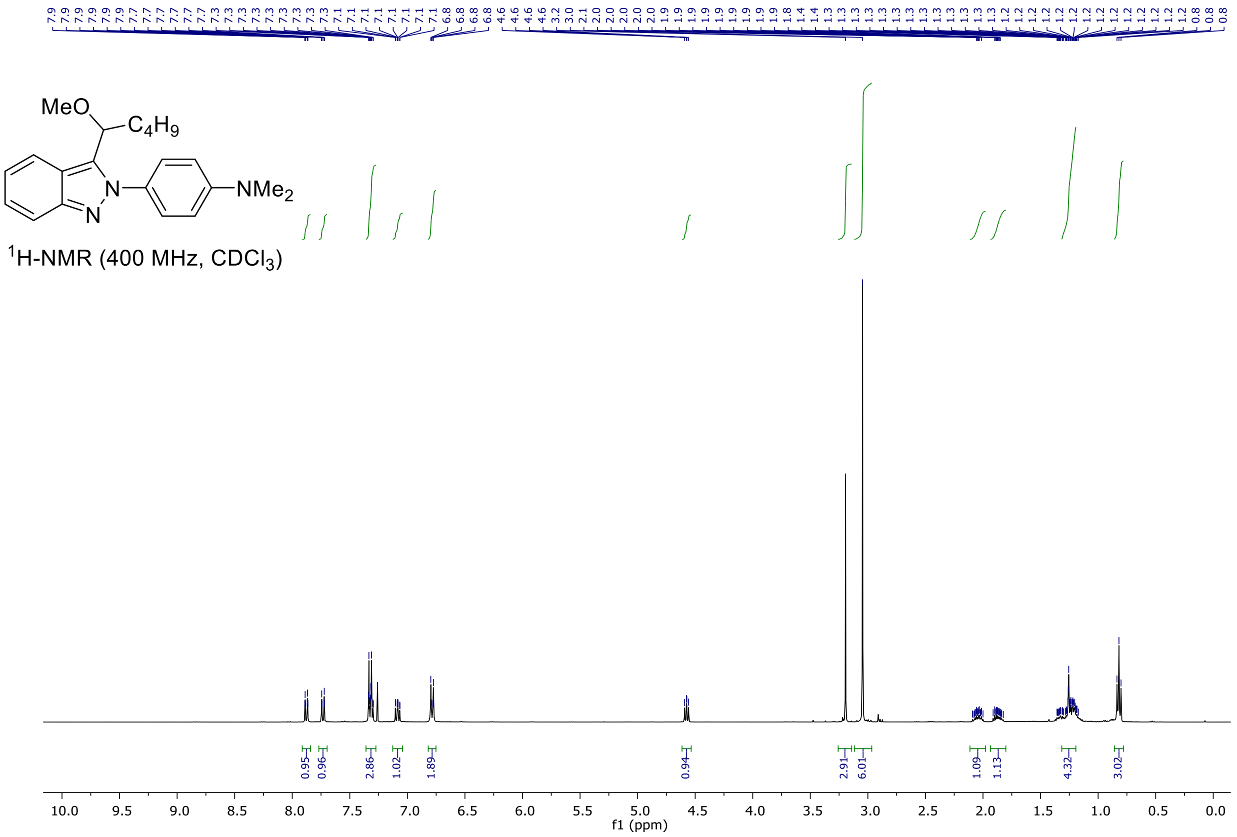

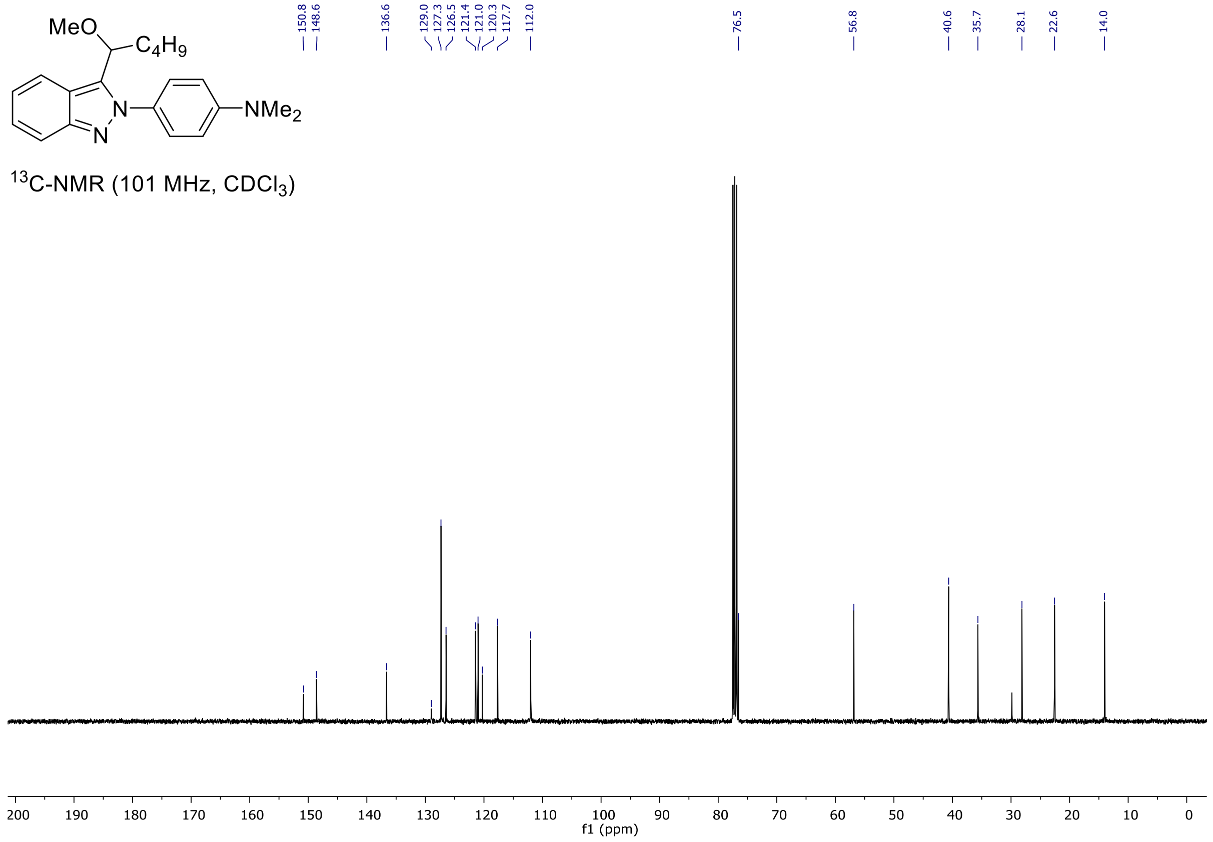

N-[4-(3-(1-Methoxypentyl)-2*H*-indazol-2-yl)phenyl]-*N*-methylacetamide (2o)

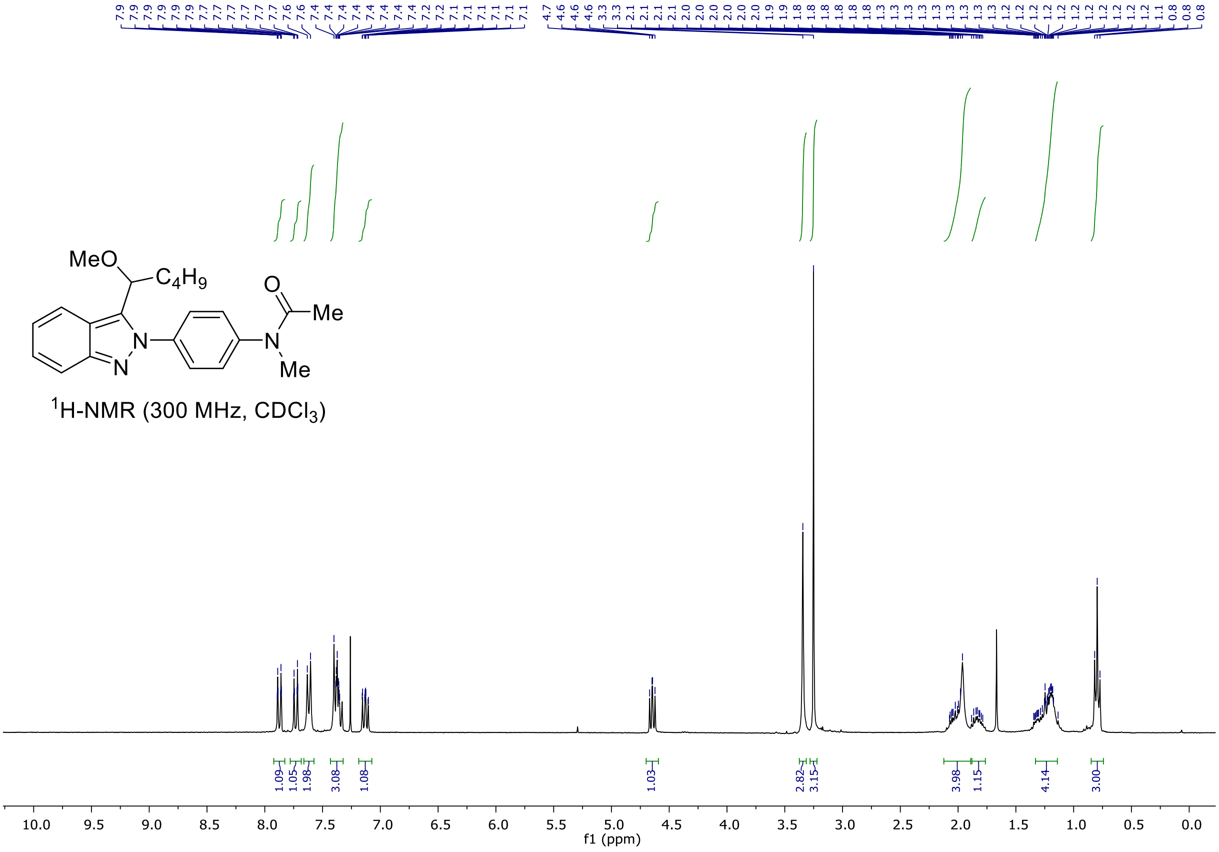

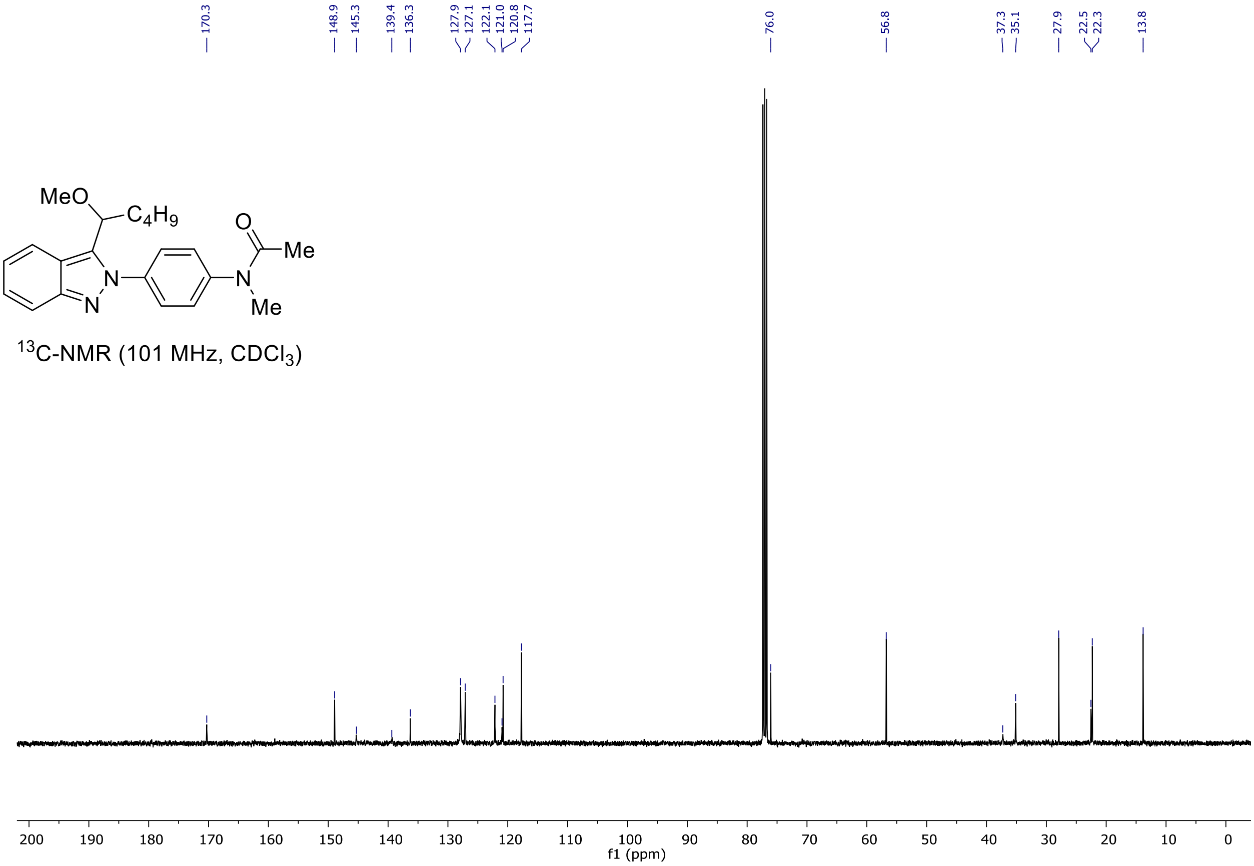

3-(1-Methoxypentyl)-2-(4-methoxyphenyl)-2*H*-indazole (2p)

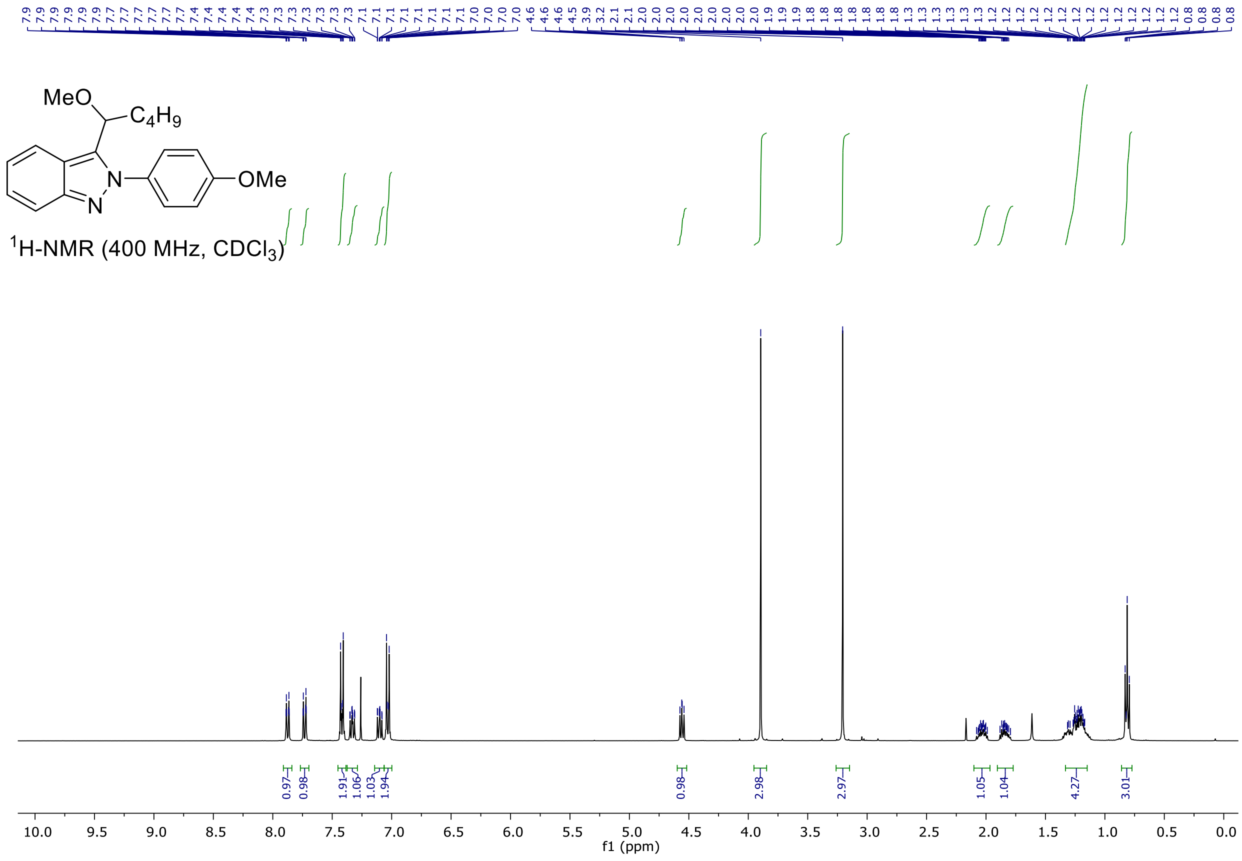

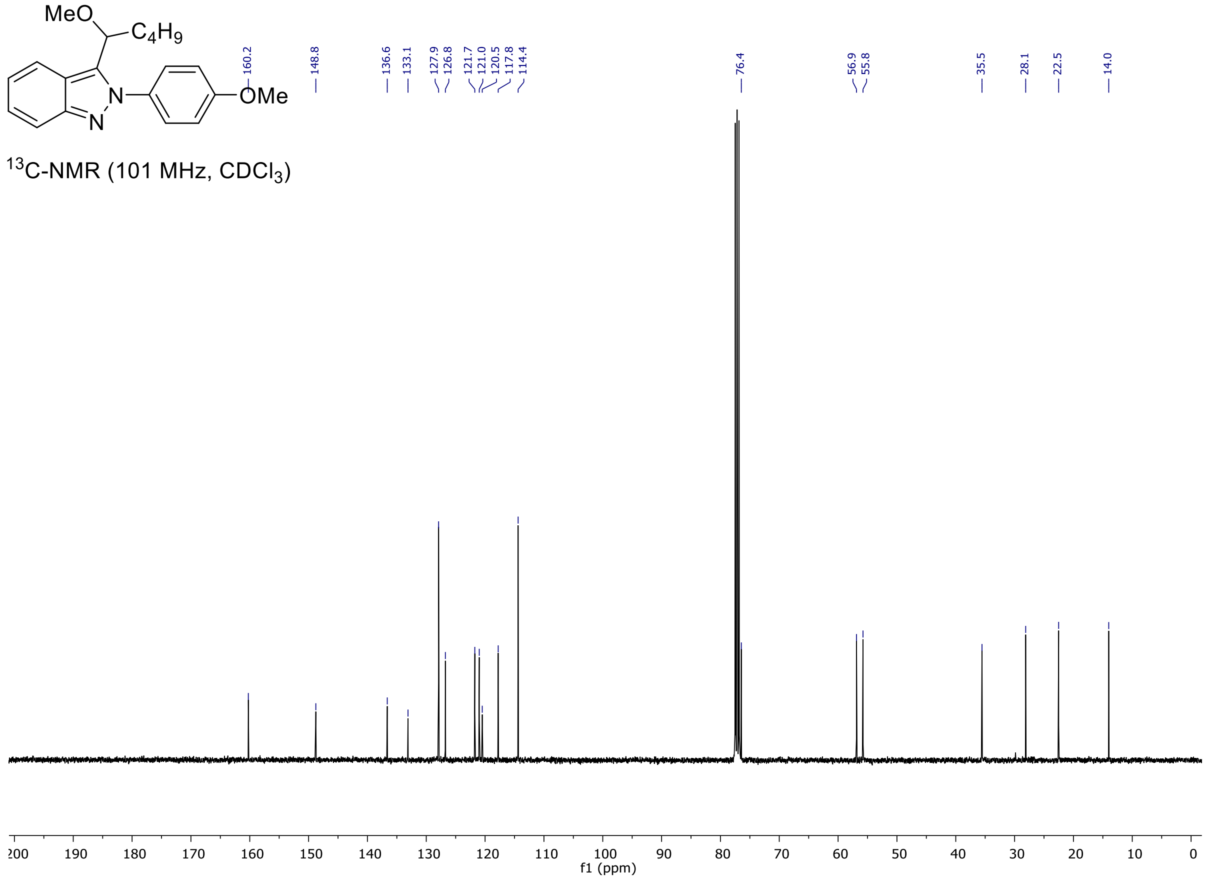

4-[3-(1-Methoxypentyl)-2*H*-indazol-2-yl]phenyl acetate (2q)

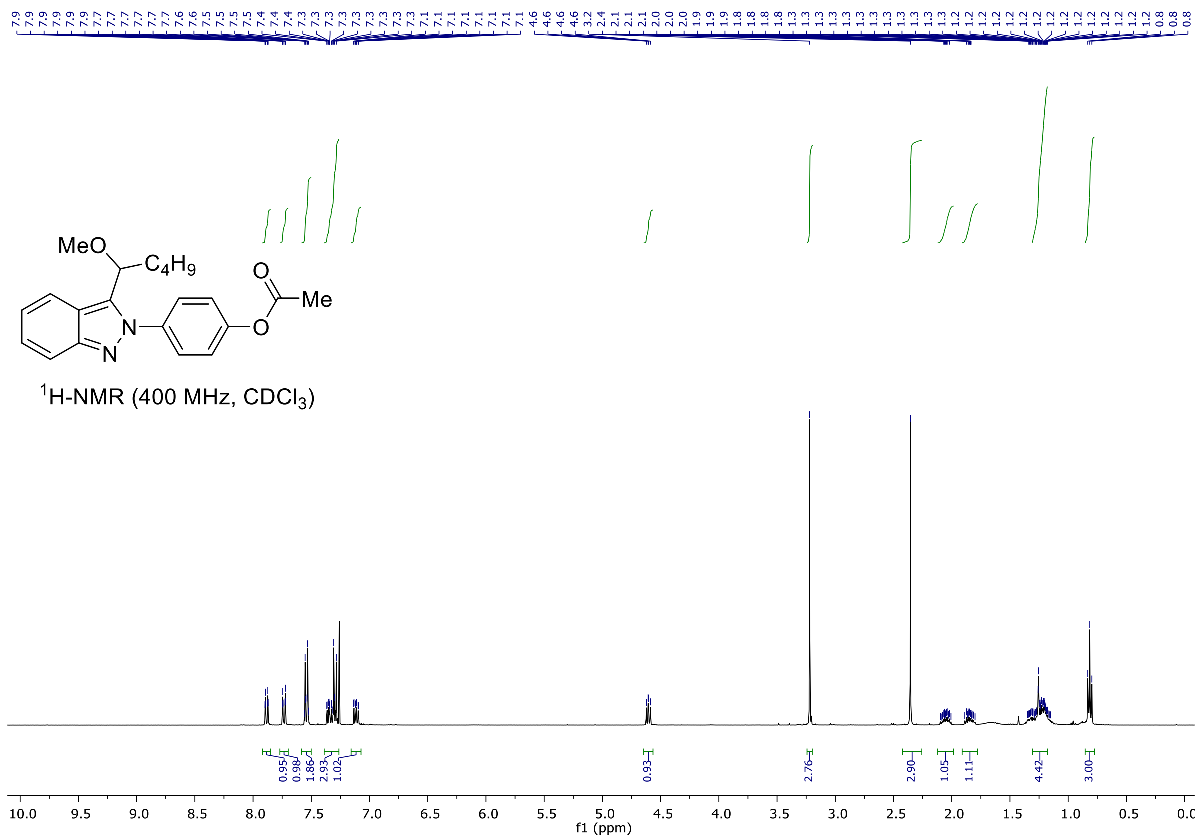

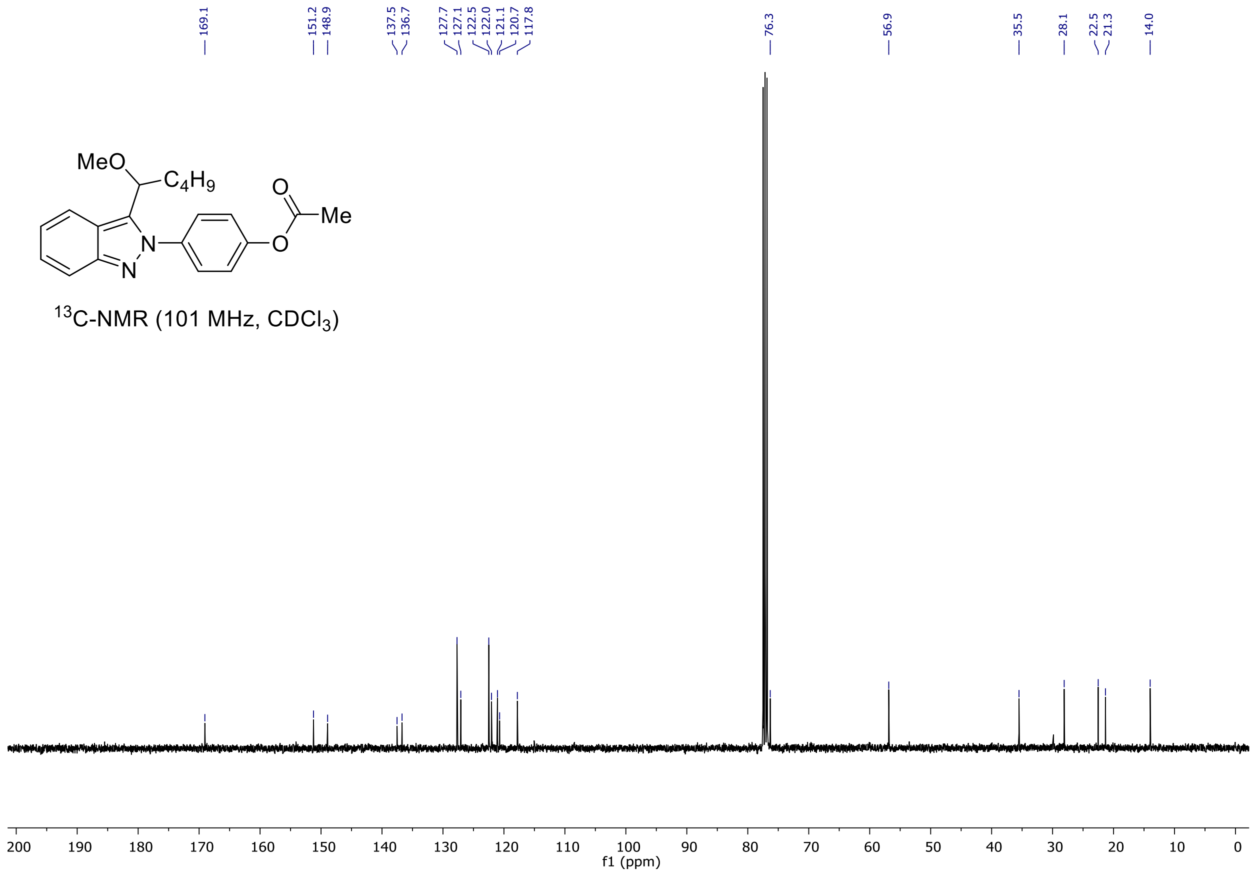

6-Chloro-3-(1-methoxypentyl)-2-phenyl-2*H*-indazole (2r)

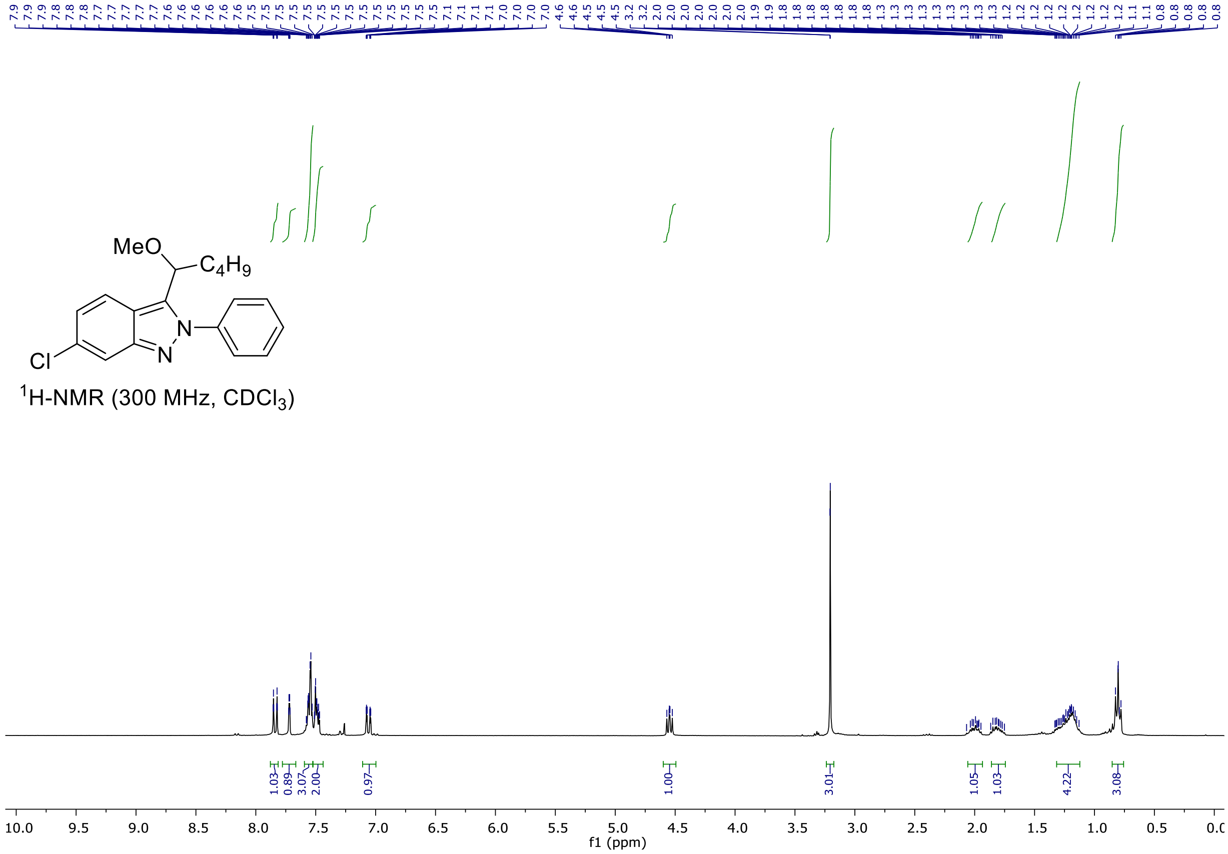

^{1}H-NMR (300 MHz, $CDCl_3$)

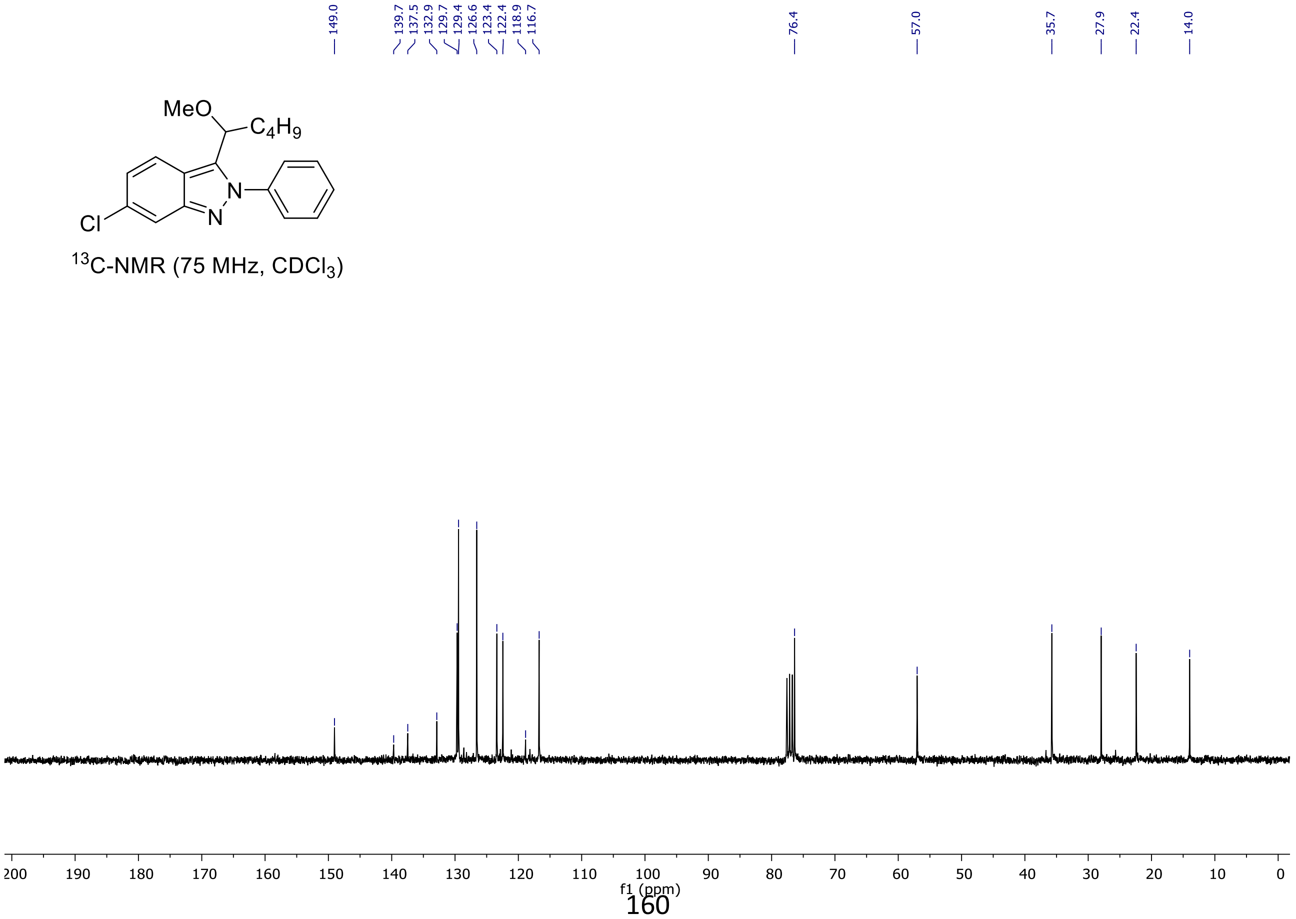

^{13}C-NMR (75 MHz, $CDCl_3$)

3-(1-Methoxypentyl)-2-phenyl-5-(trifluoromethyl)-2*H*-indazole (2s)

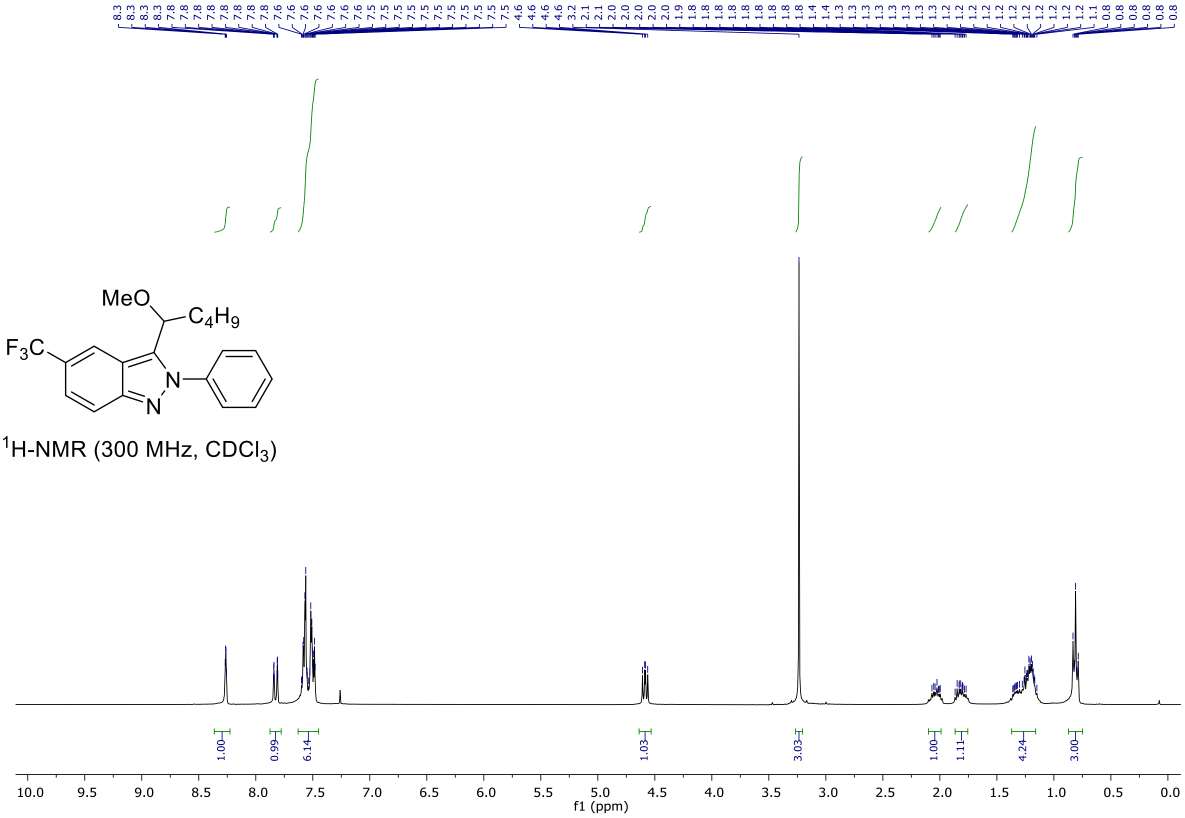

^{1}H-NMR (300 MHz, $CDCl_3$)

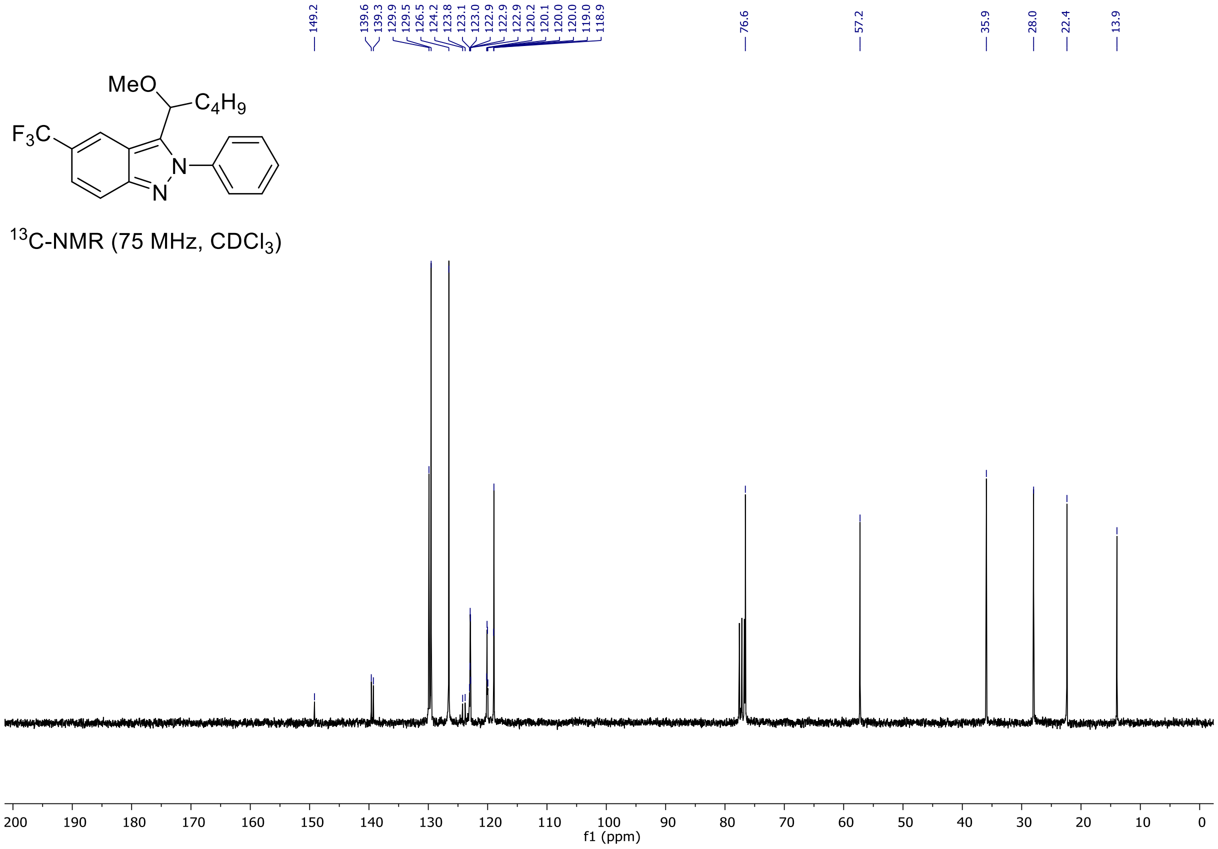

^{13}C-NMR (75 MHz, $CDCl_3$)

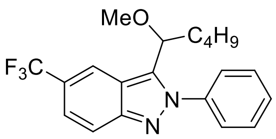

^{19}F-NMR (282 MHz, $CDCl_3$)

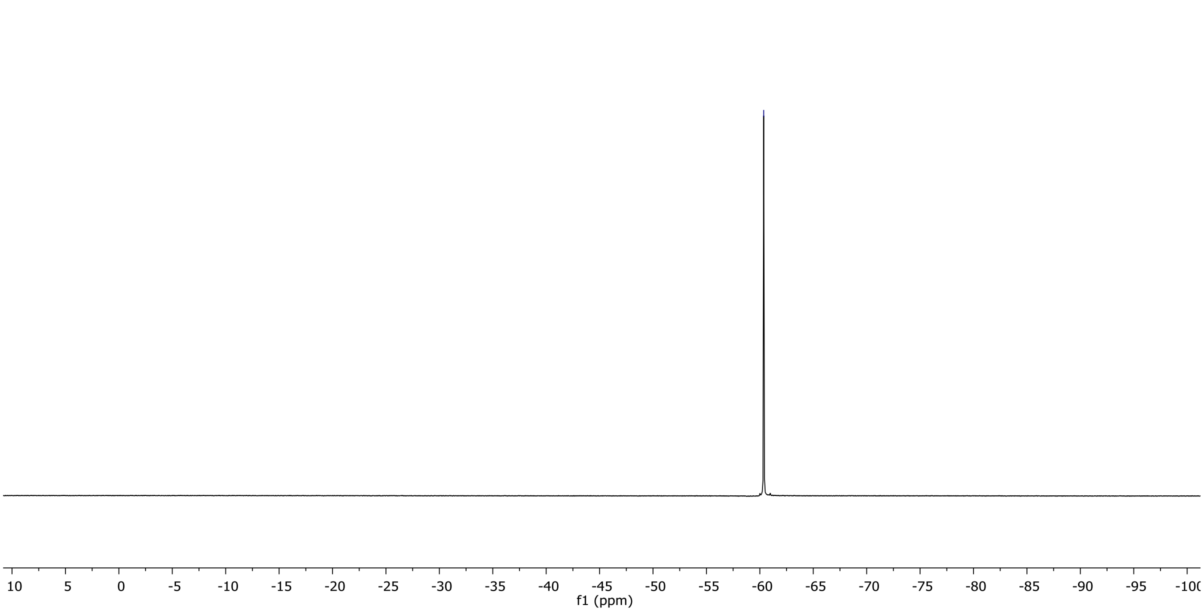

Methyl 3-(1-methoxypentyl)-2-phenyl-2*H*-indazole-5-carboxylate (2t)

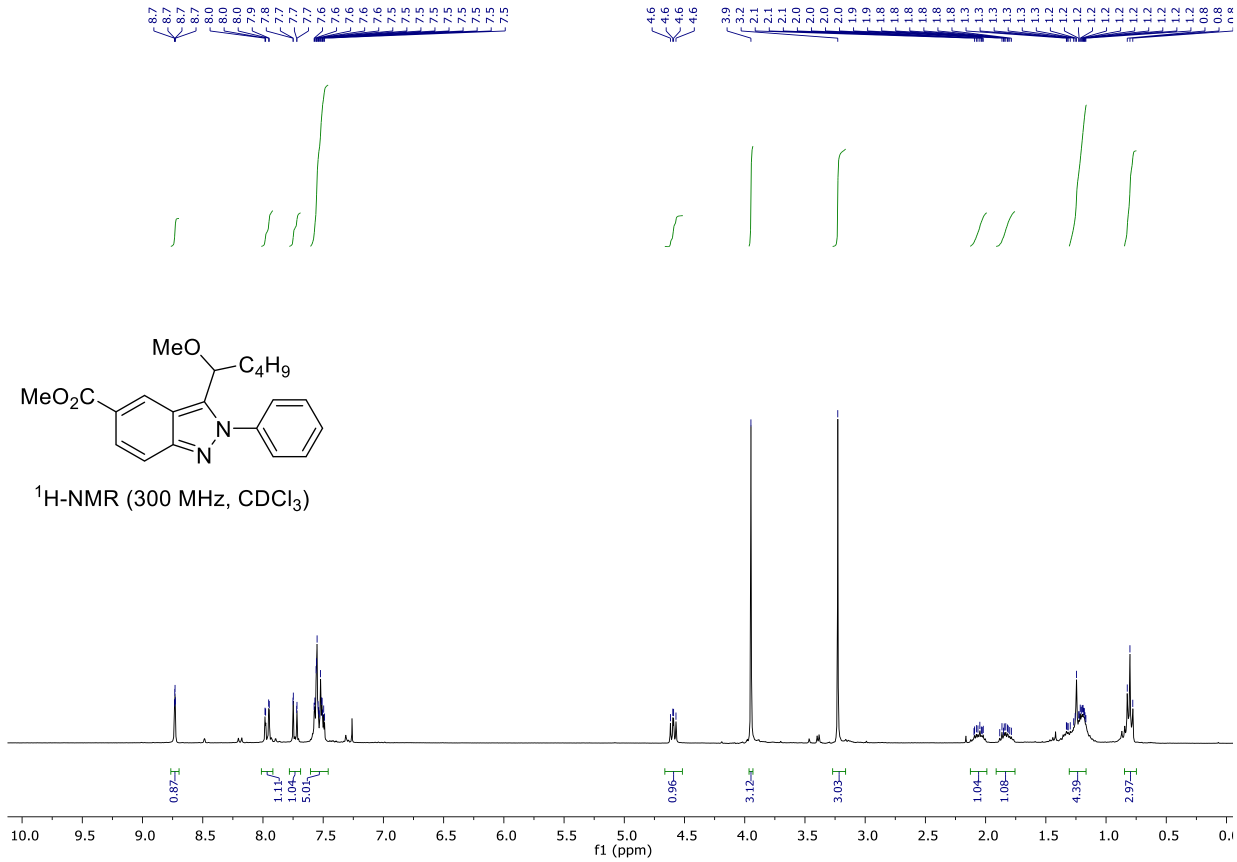

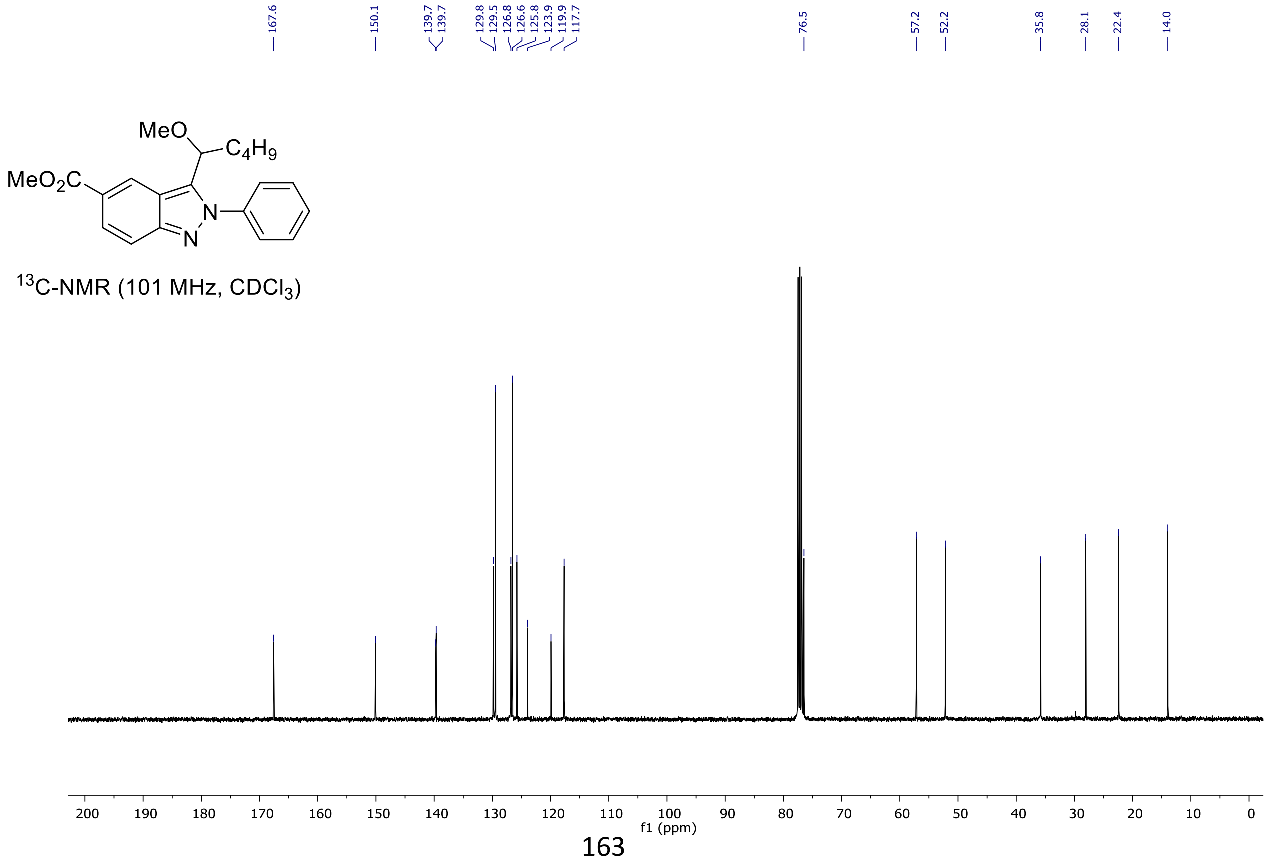

3-(1-Methoxypentyl)-5-methyl-2-phenyl-2*H*-indazole (2u)

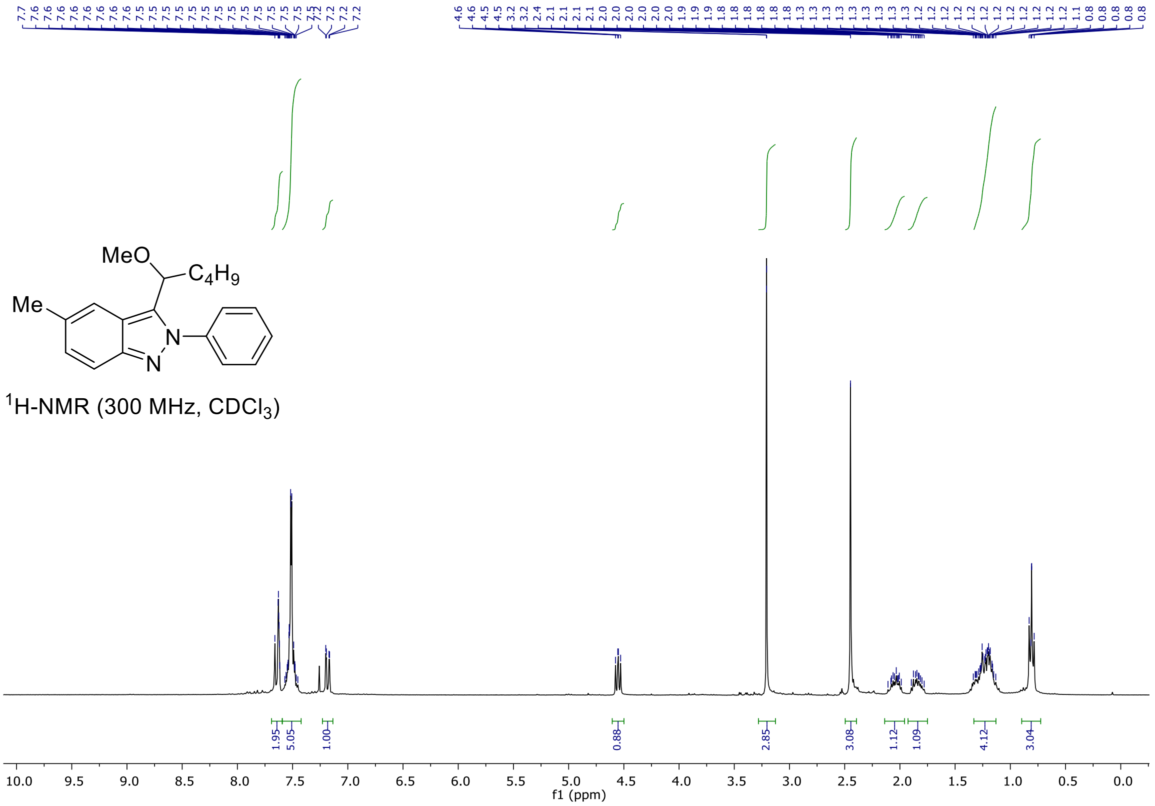

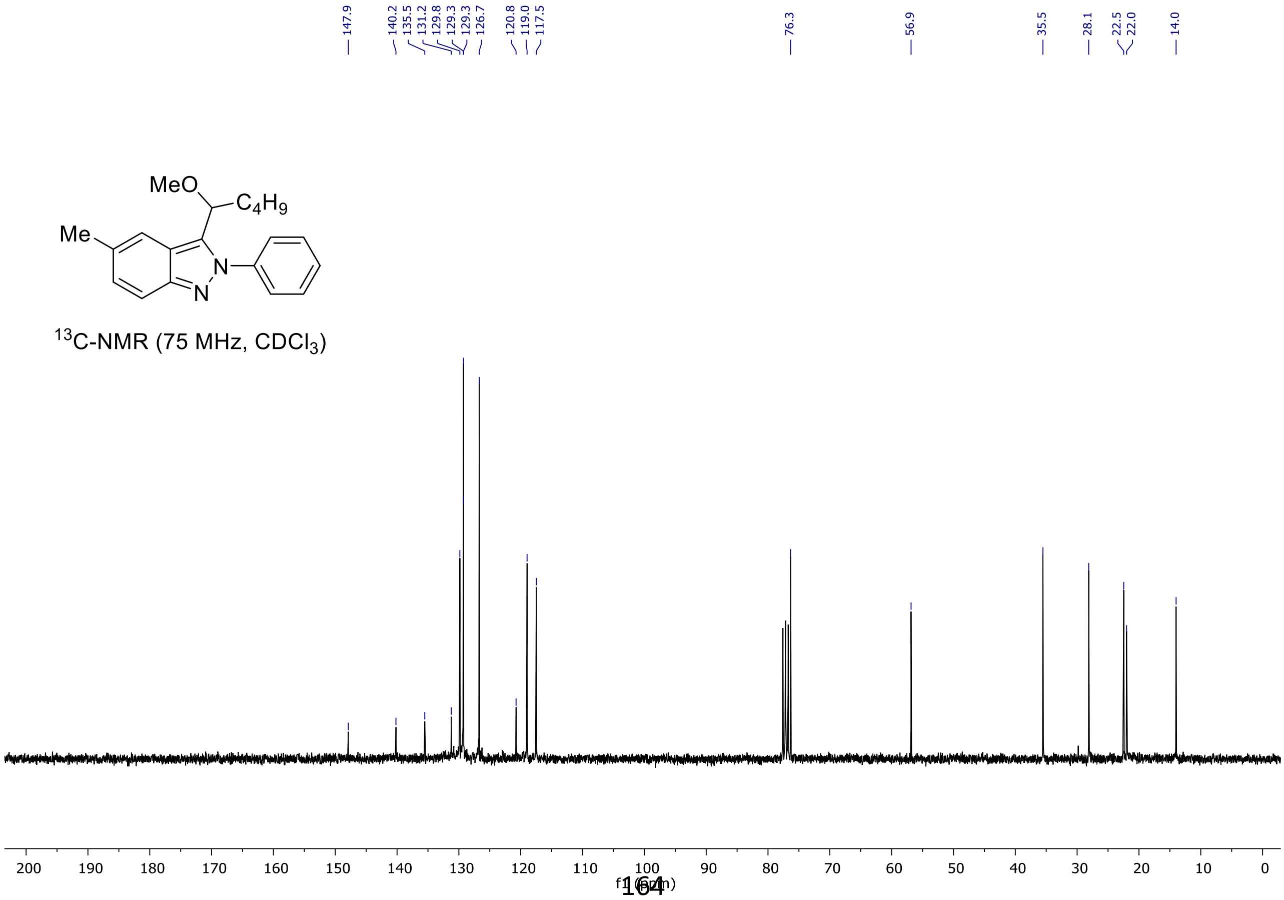

3-(1-Methoxypentyl)-4-methyl-2-phenyl-2*H*-indazole (2v)

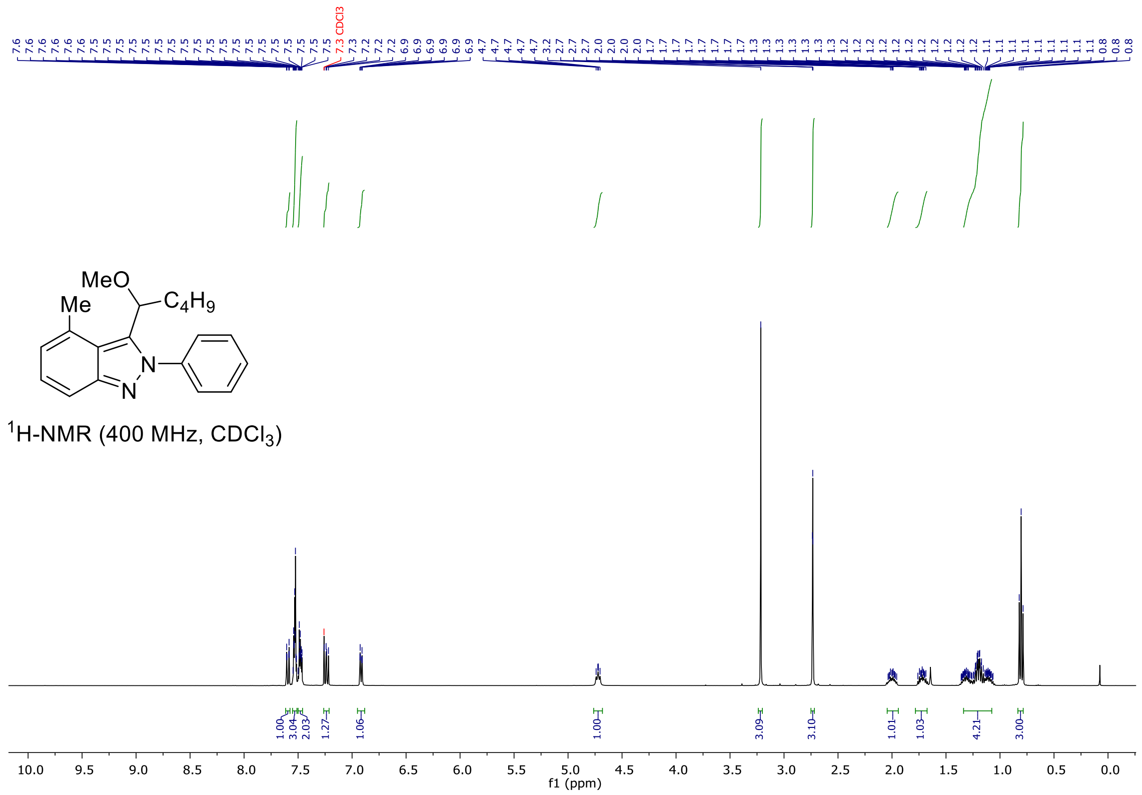

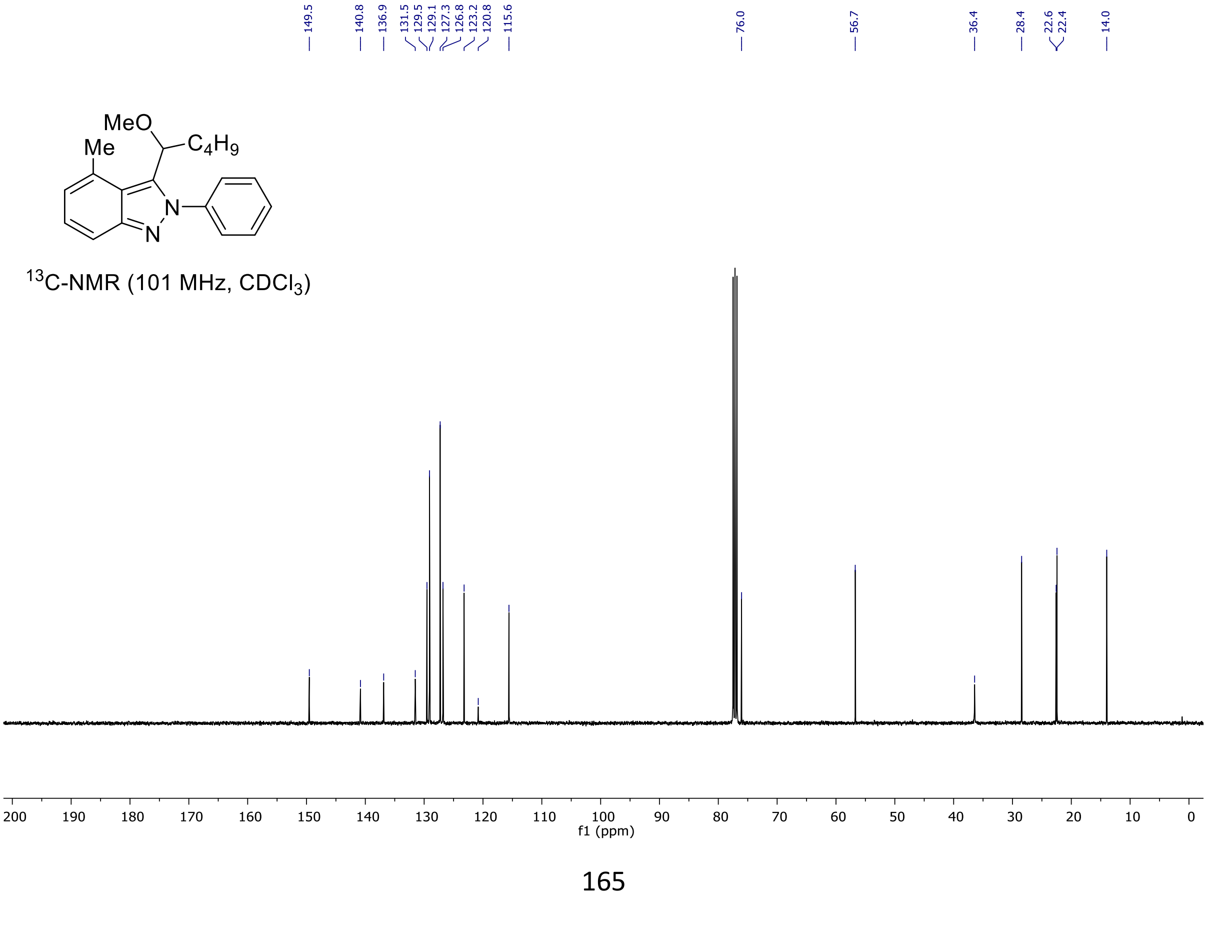

3-(Methoxymethyl)-2-phenyl-2*H*-indazole (2w)

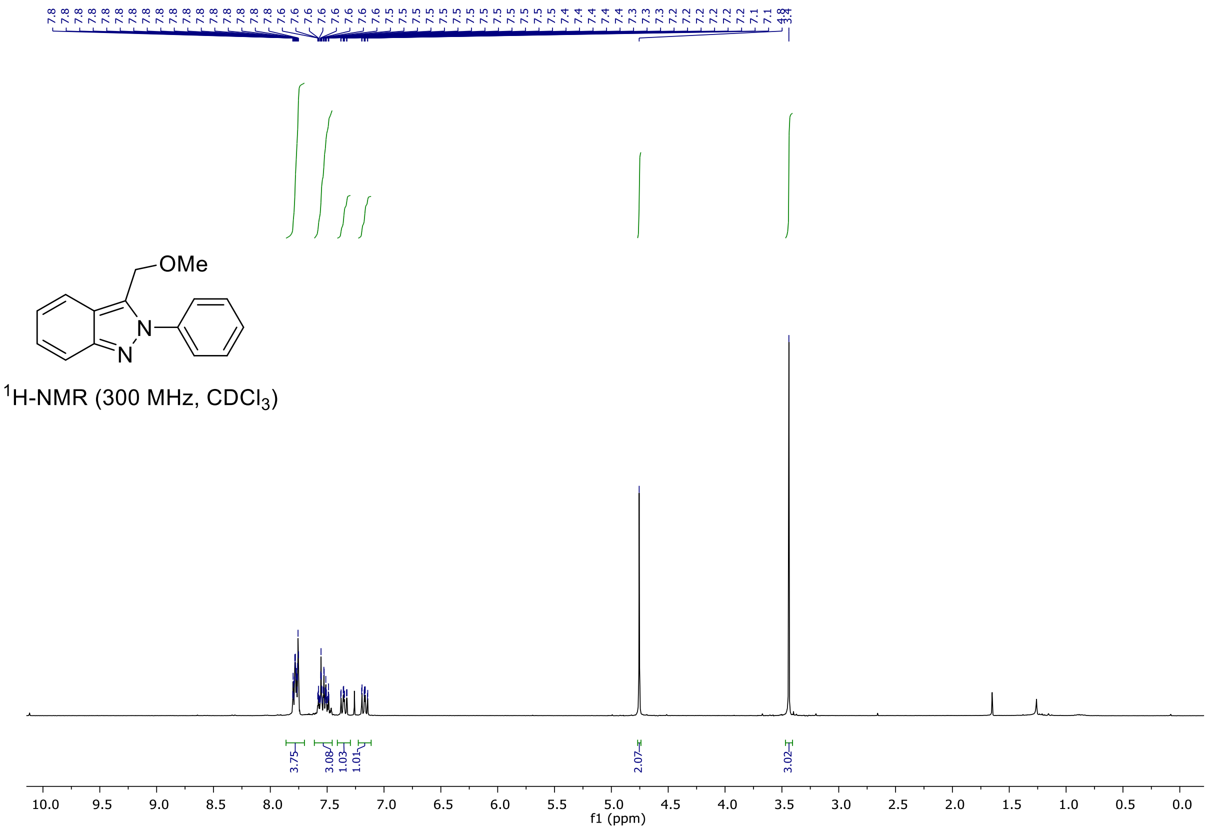

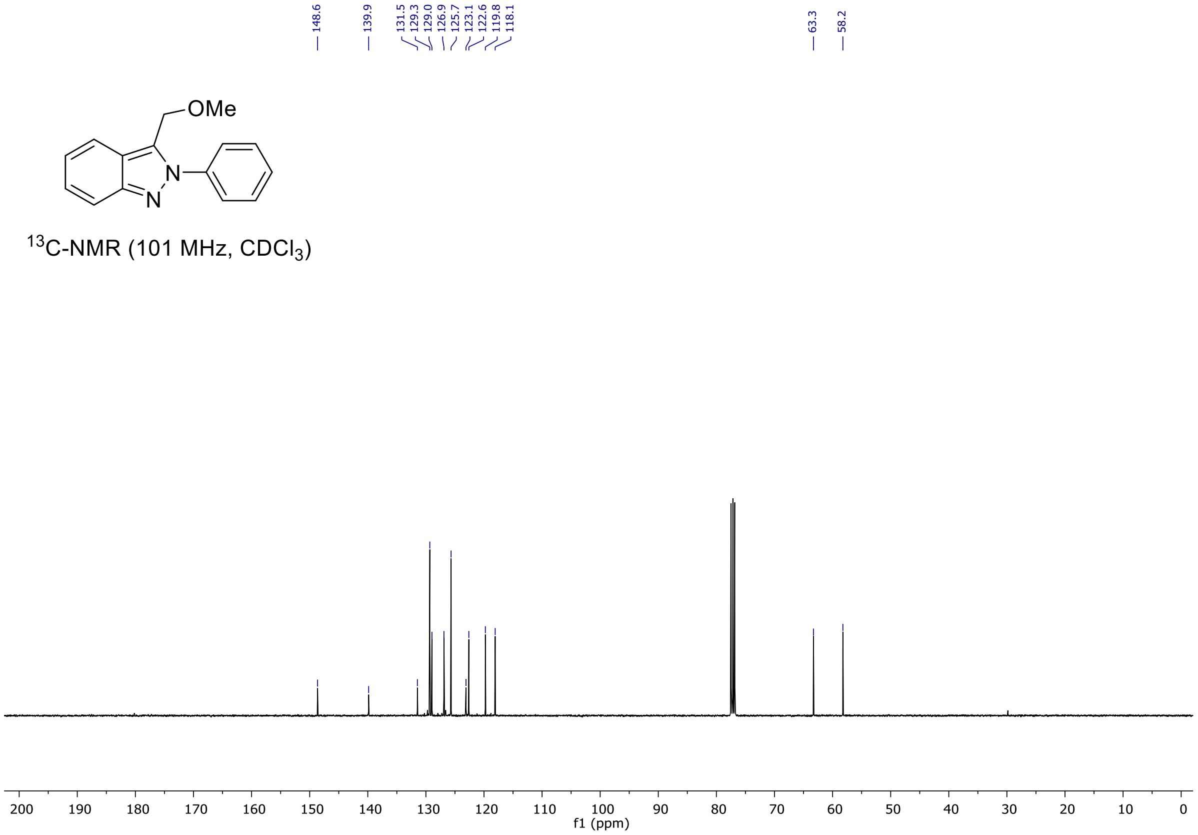

3-(1-Methoxy-4-methylpentyl)-2-phenyl-2*H*-indazole (2x)

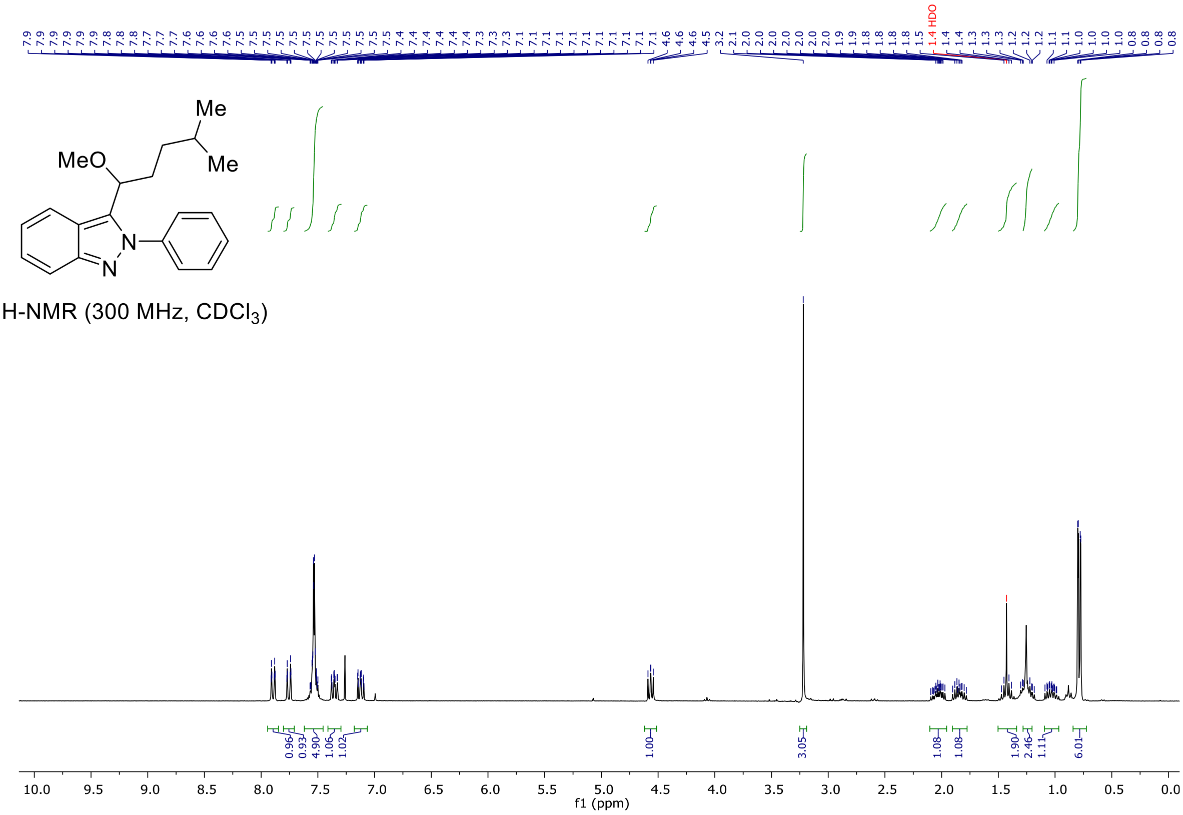

^{1}H-NMR (300 MHz, $CDCl_3$)

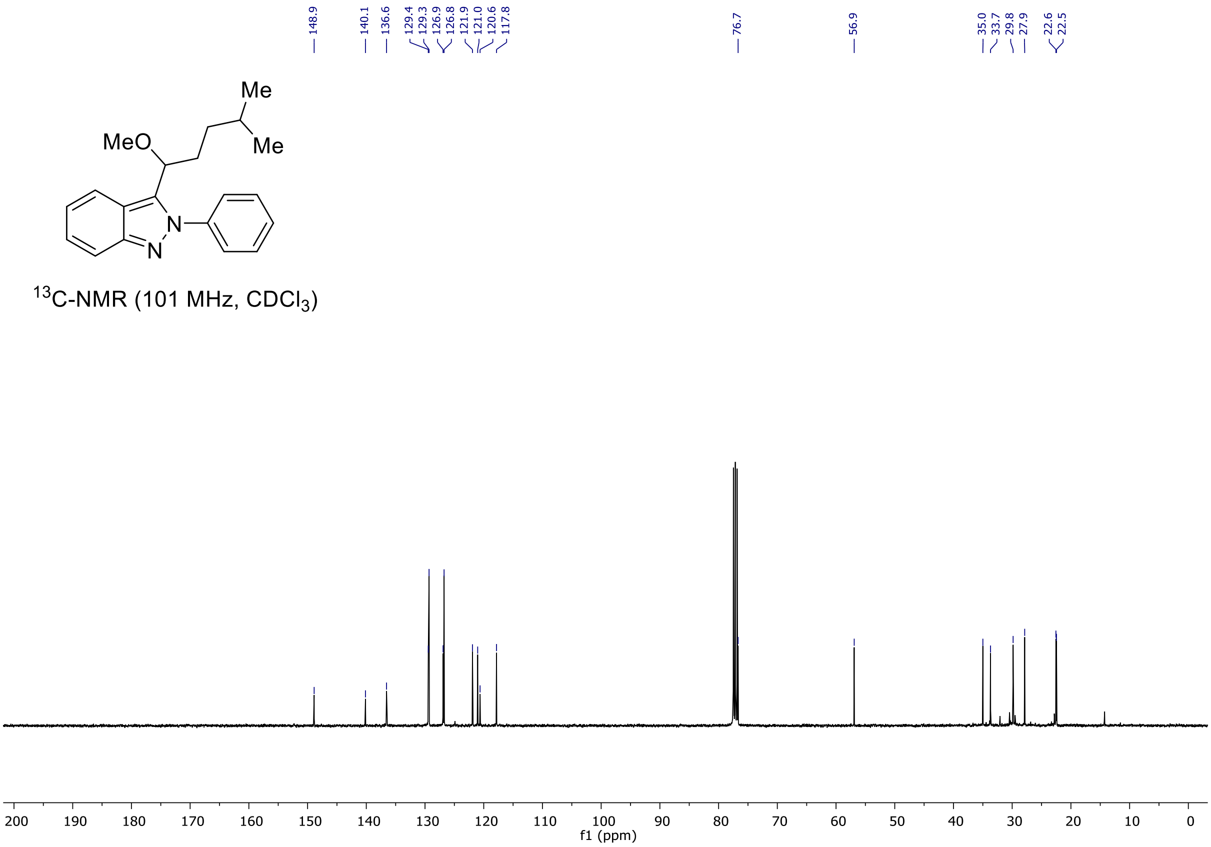

^{13}C-NMR (101 MHz, $CDCl_3$)

3-(2-Cyclohexyl-1-methoxyethyl)-2-phenyl-2*H*-indazole (2y)

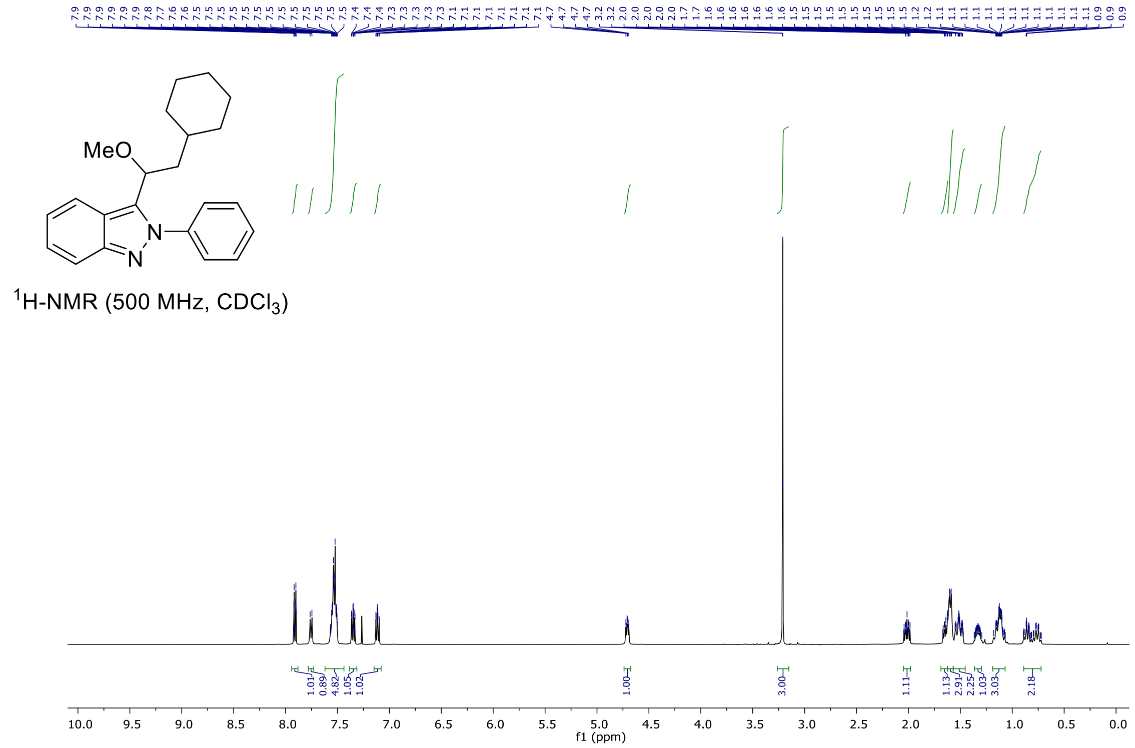

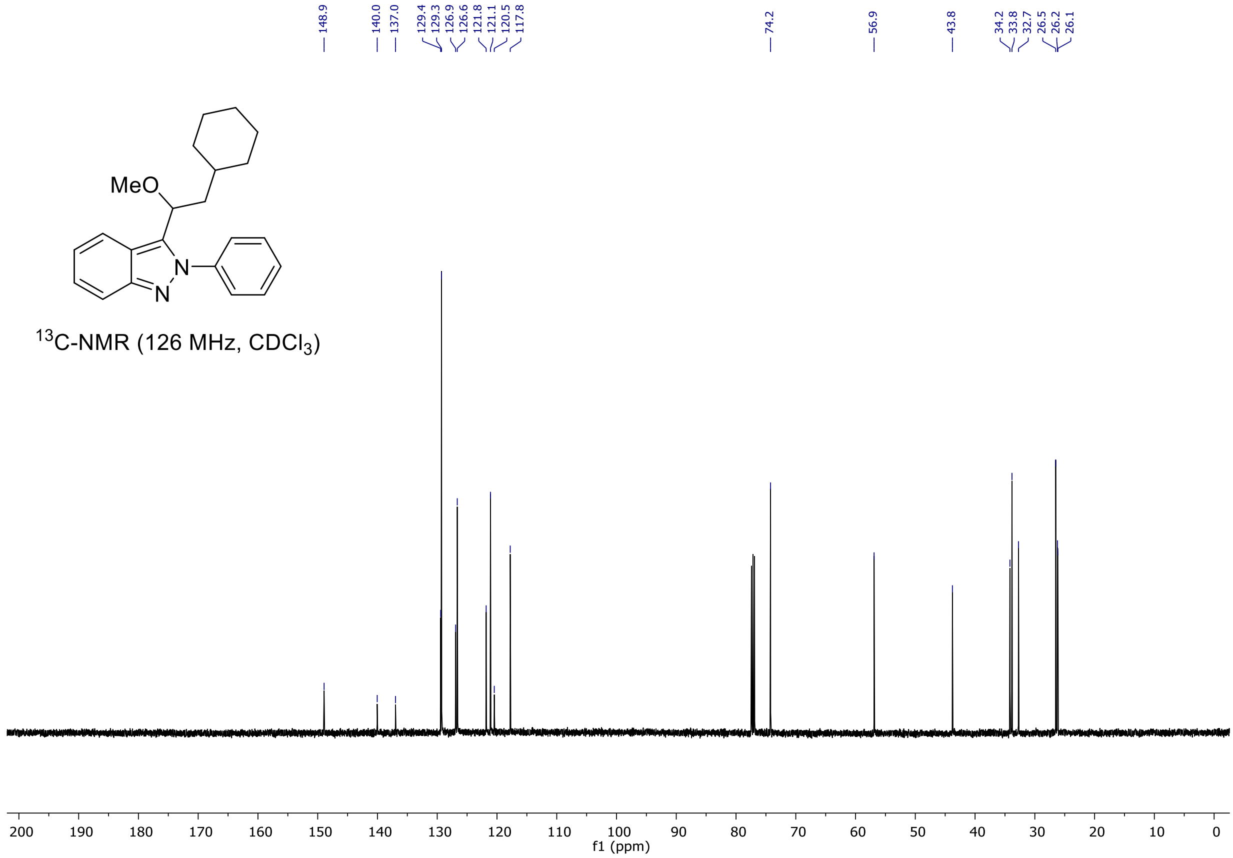

2-(4-Bromophenyl)-3-[cyclopropyl(methoxy)methyl]-2*H*-indazole (2z)

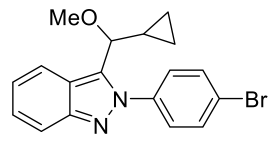

^{1}H-NMR (400 MHz, $CDCl_3$)

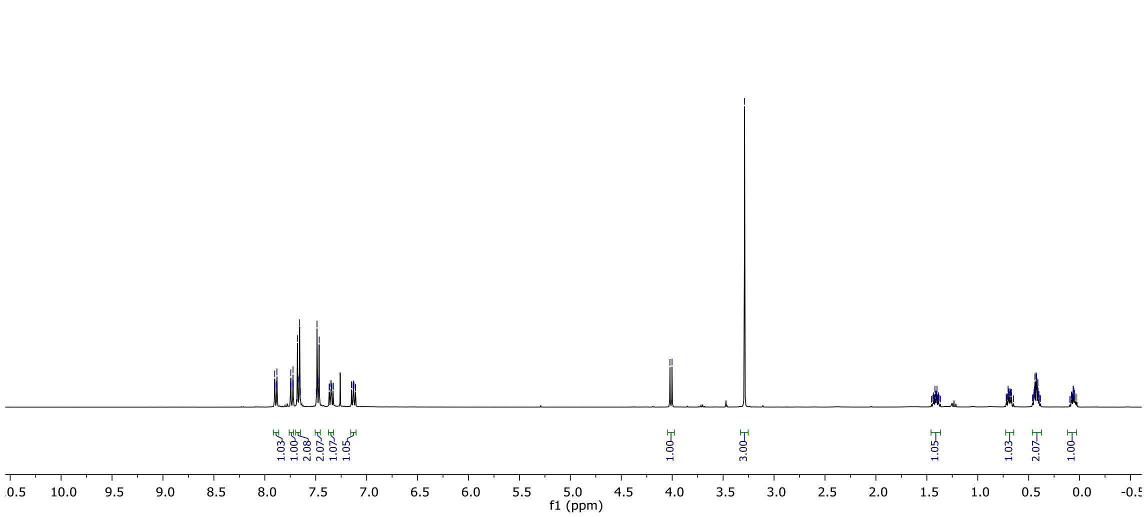

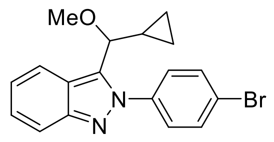

^{13}C-NMR (101 MHz, $CDCl_3$)

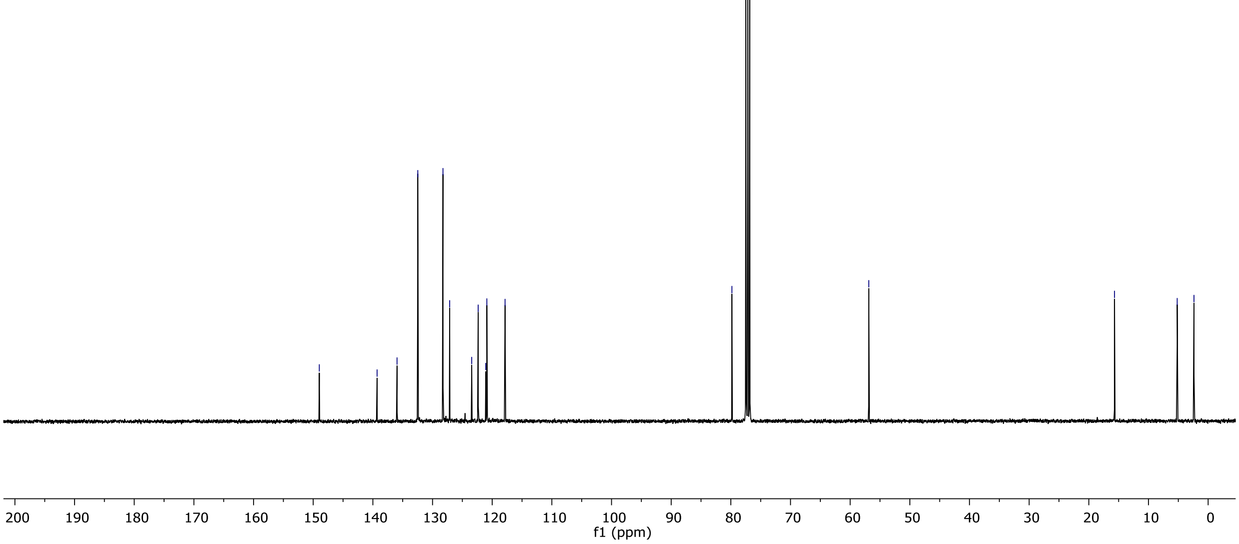

3-(4-Chloro-1-methoxybutyl)-2-phenyl-2*H*-indazole (2aa)

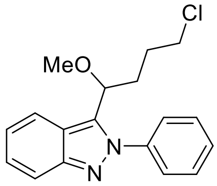

^{1}H-NMR (300 MHz, $CDCl_3$)

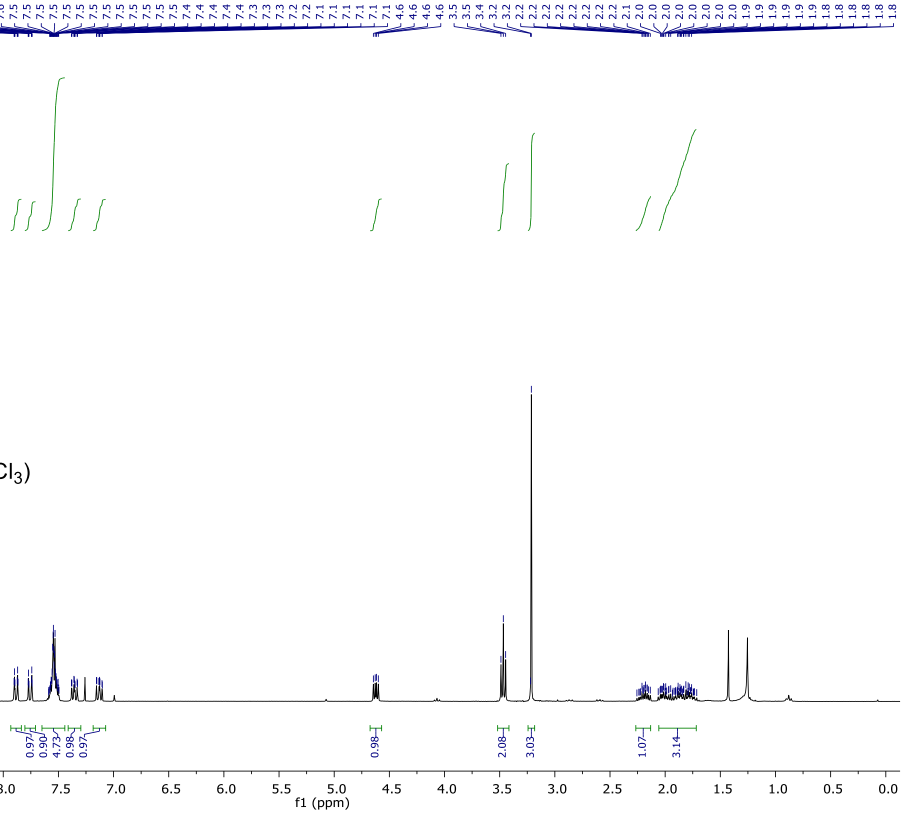

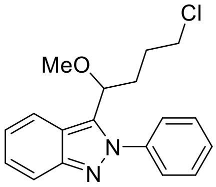

^{13}C-NMR (101 MHz, $CDCl_3$)

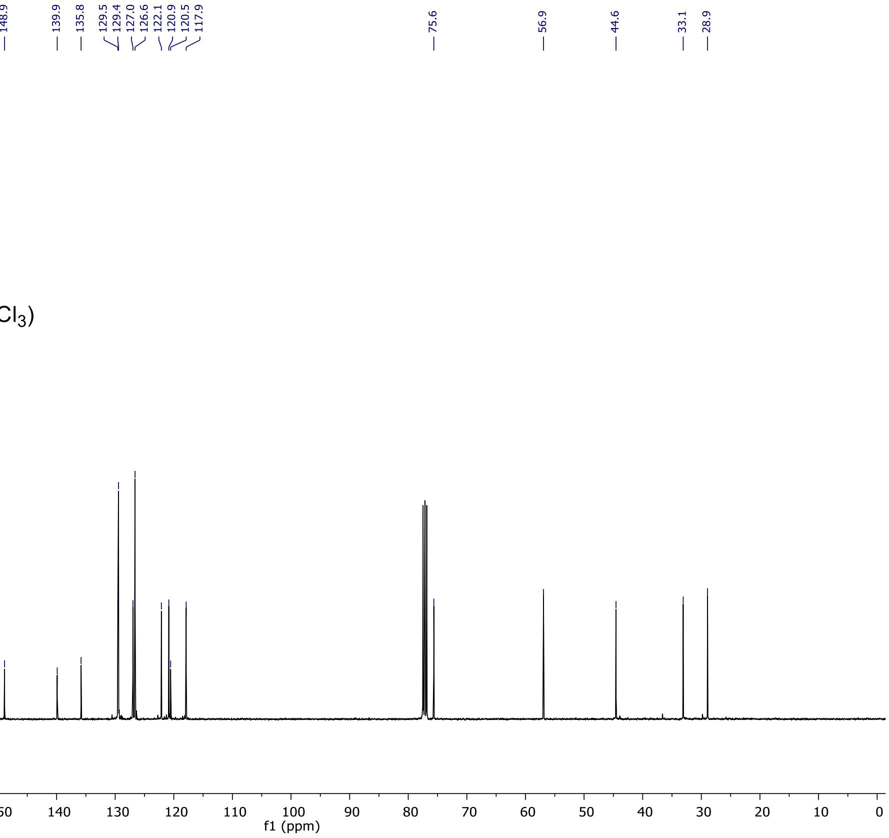

3-Methoxy-3-(2-phenyl-2*H*-indazol-3-yl)propan-1-ol (2ab)

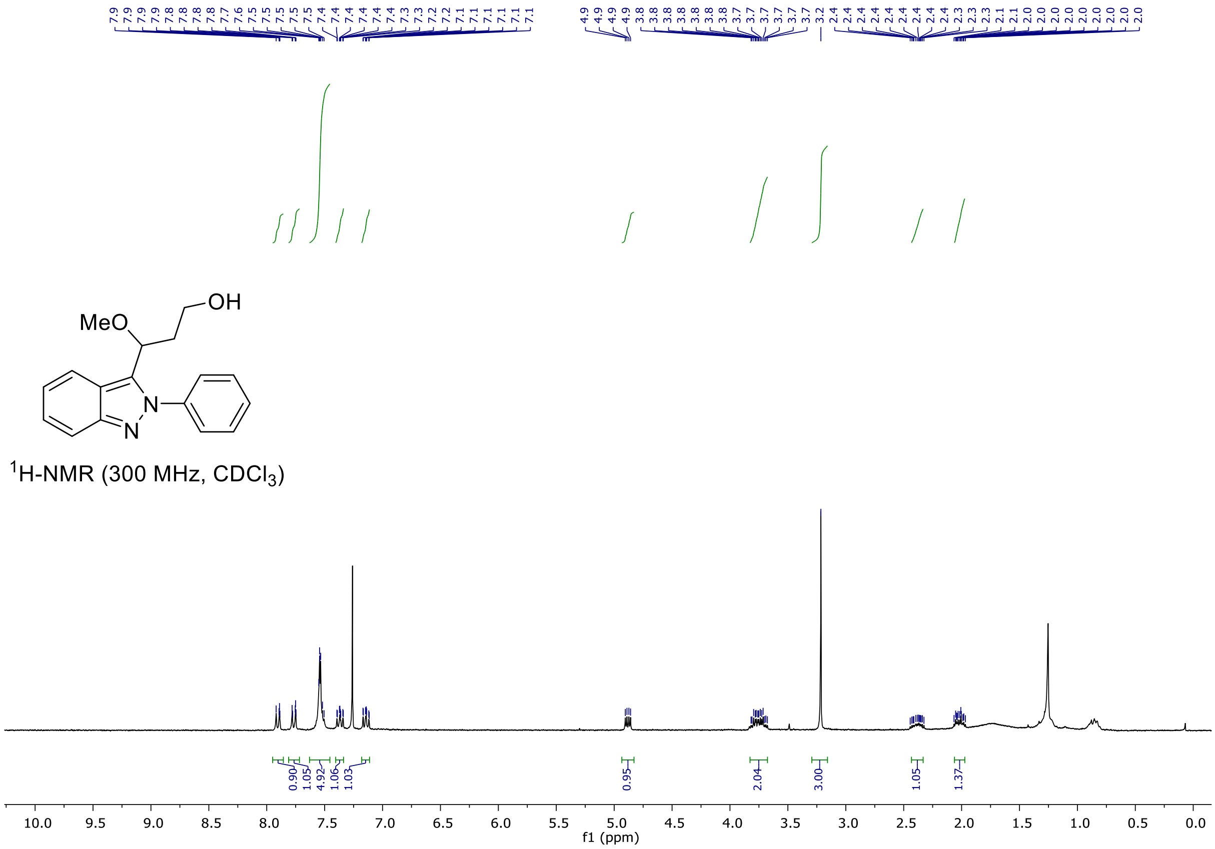

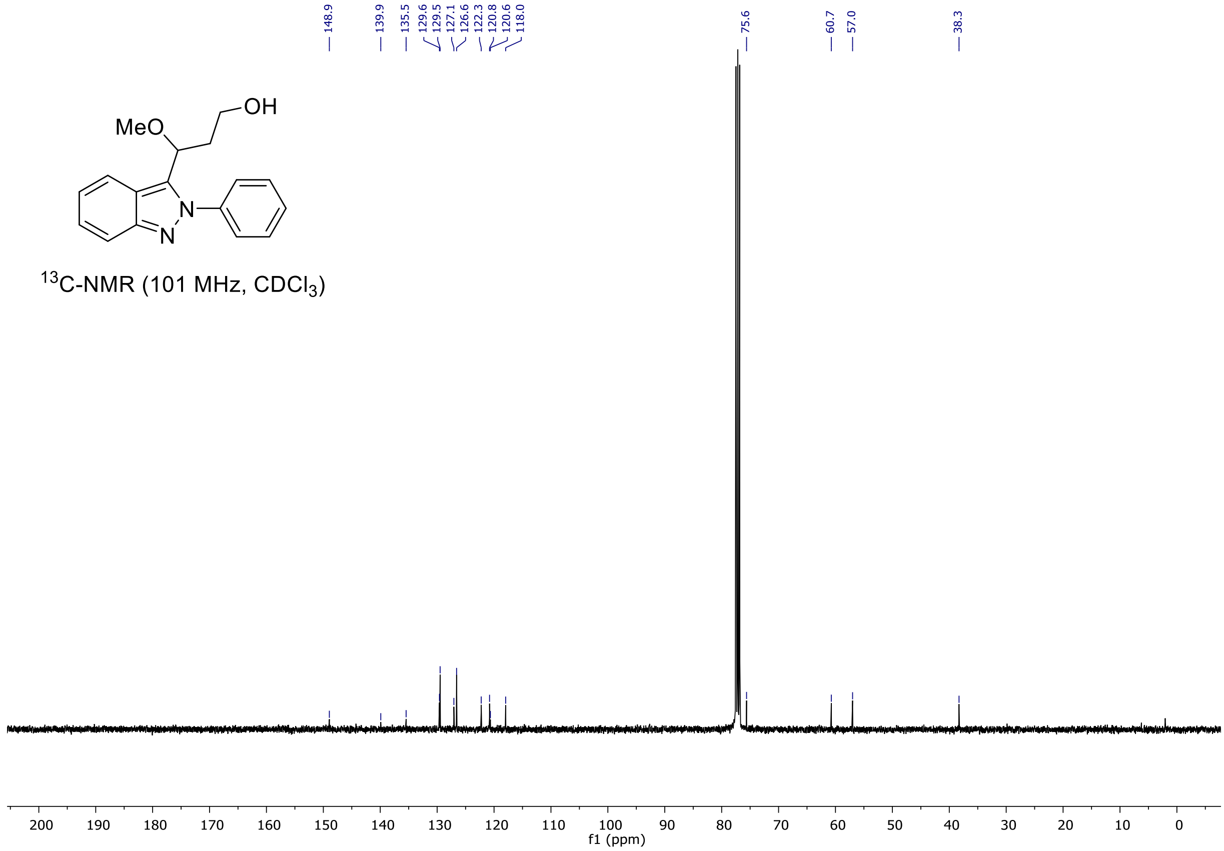

3-(1-Methoxy-3-phenylpropyl)-2-phenyl-2*H*-indazole (2ac)

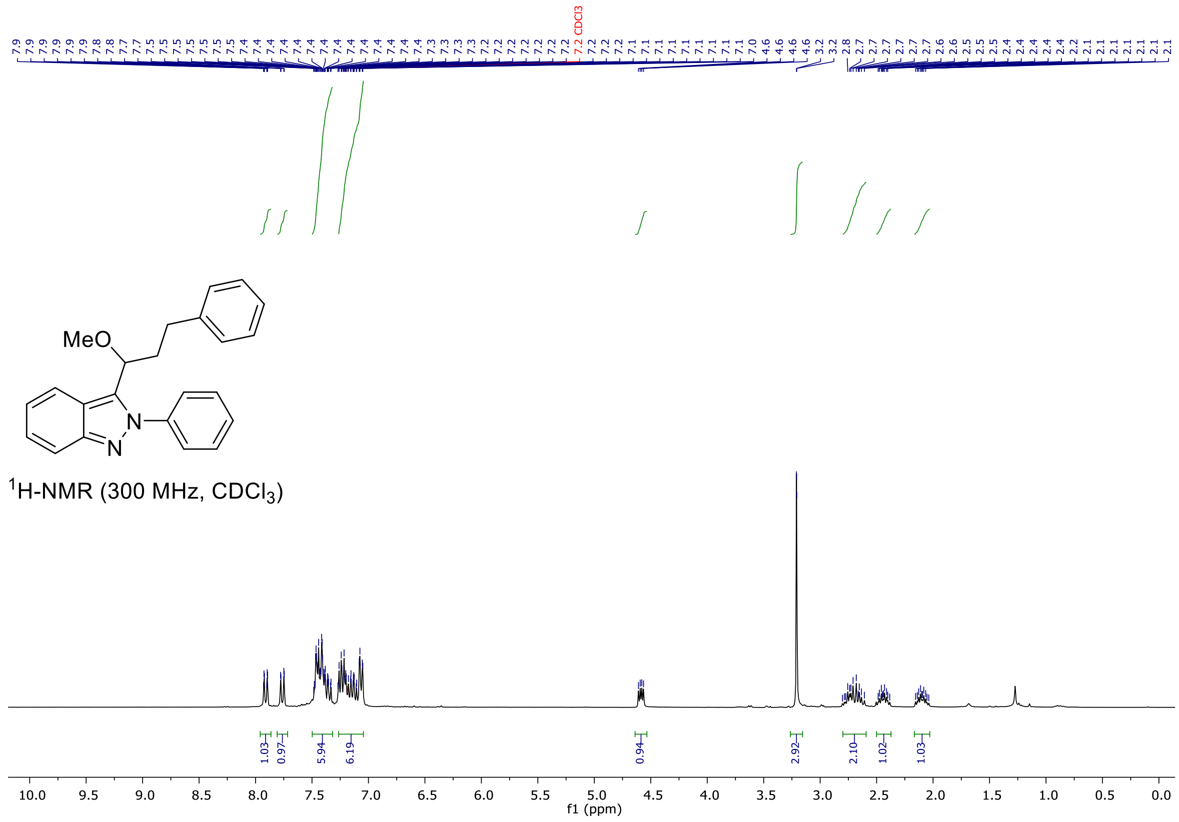

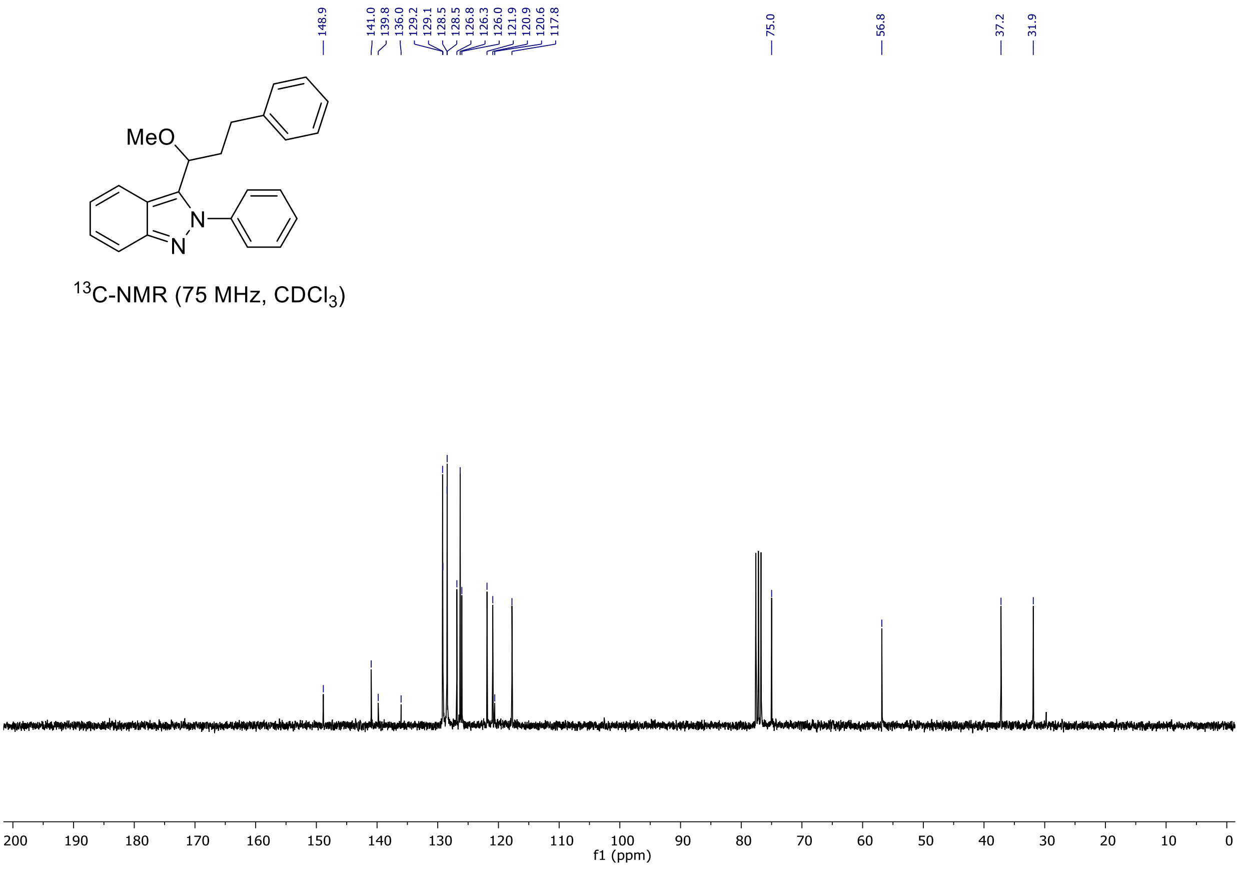

3-[Methoxy(4-methoxyphenyl)methyl]-2-phenyl-2*H*-indazole (2ad)

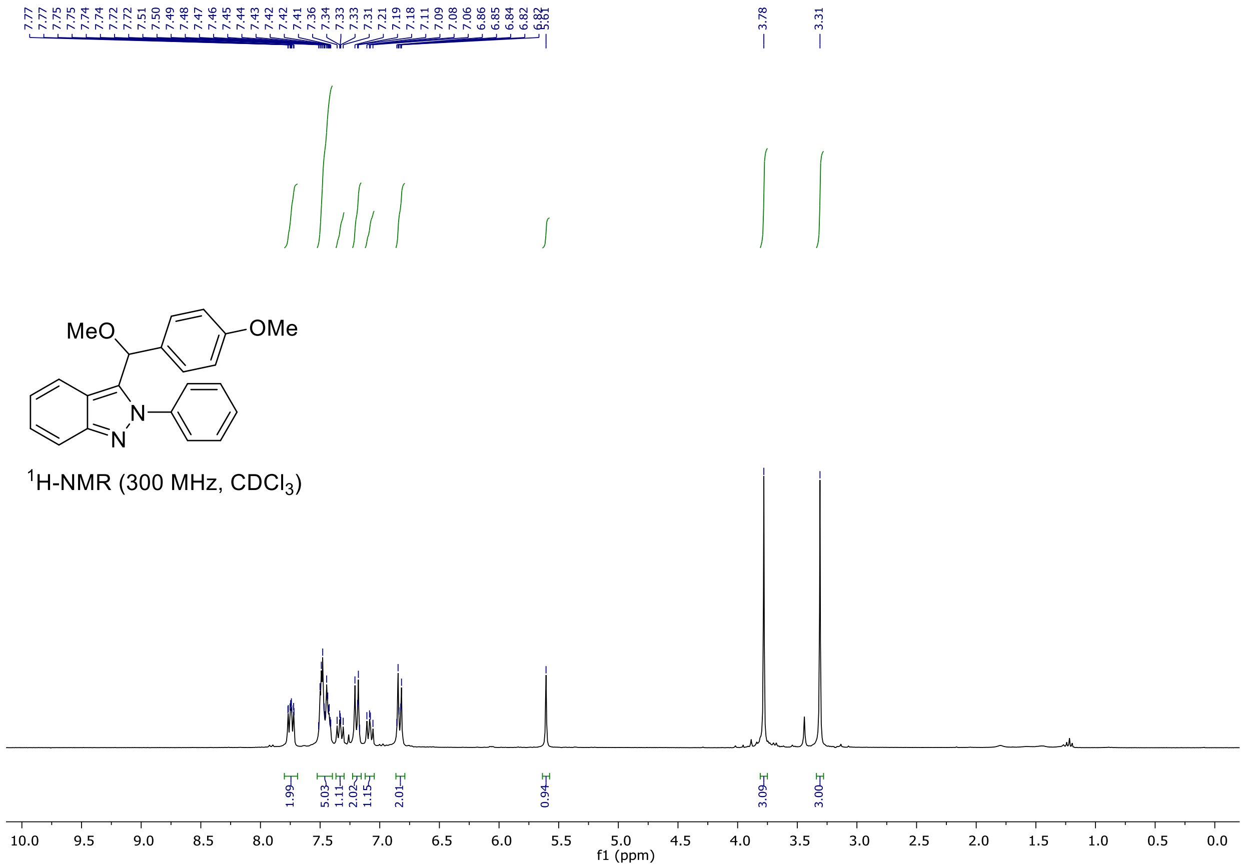

^{1}H-NMR (300 MHz, $CDCl_3$)

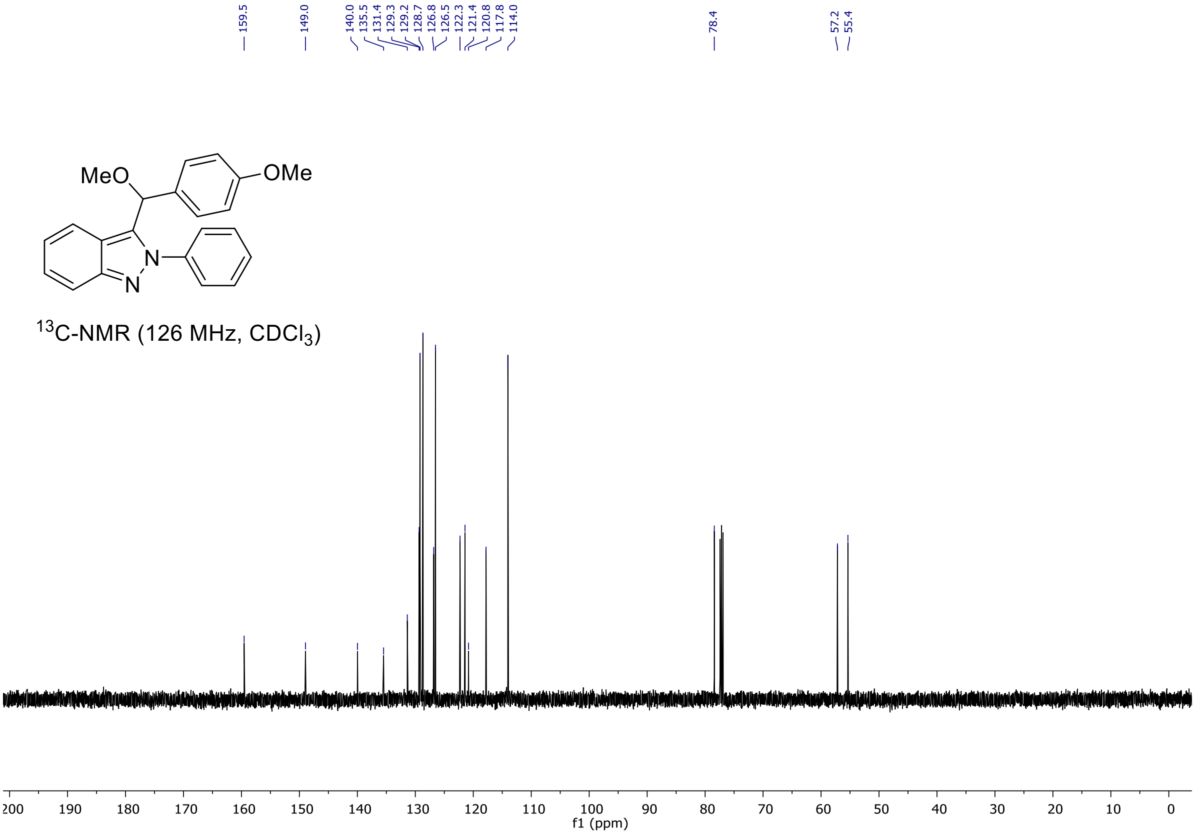

^{13}C-NMR (126 MHz, $CDCl_3$)

3-(1-Ethoxypentyl)-2-phenyl-2*H*-indazole (2ae)

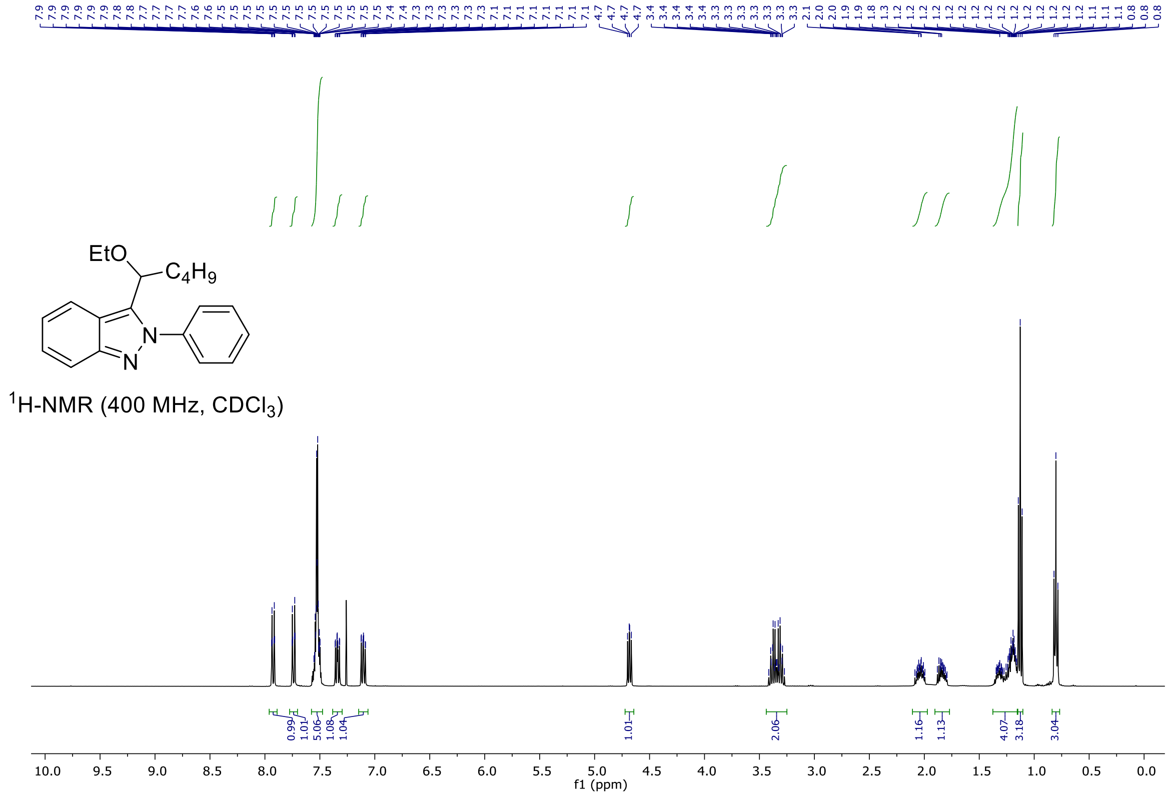

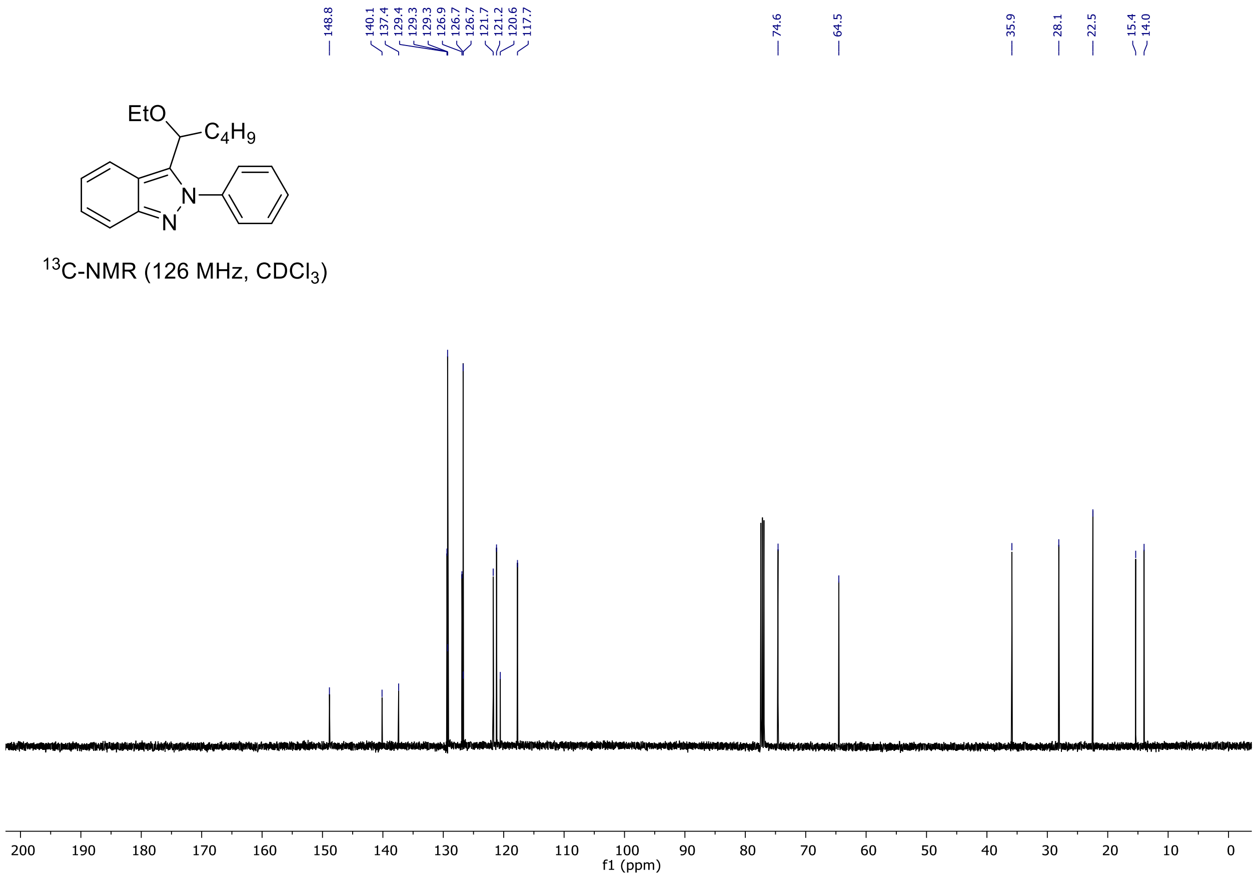

3-[1-(*tert*-Butoxy)pentyl]-2-phenyl-2*H*-indazole (2af)

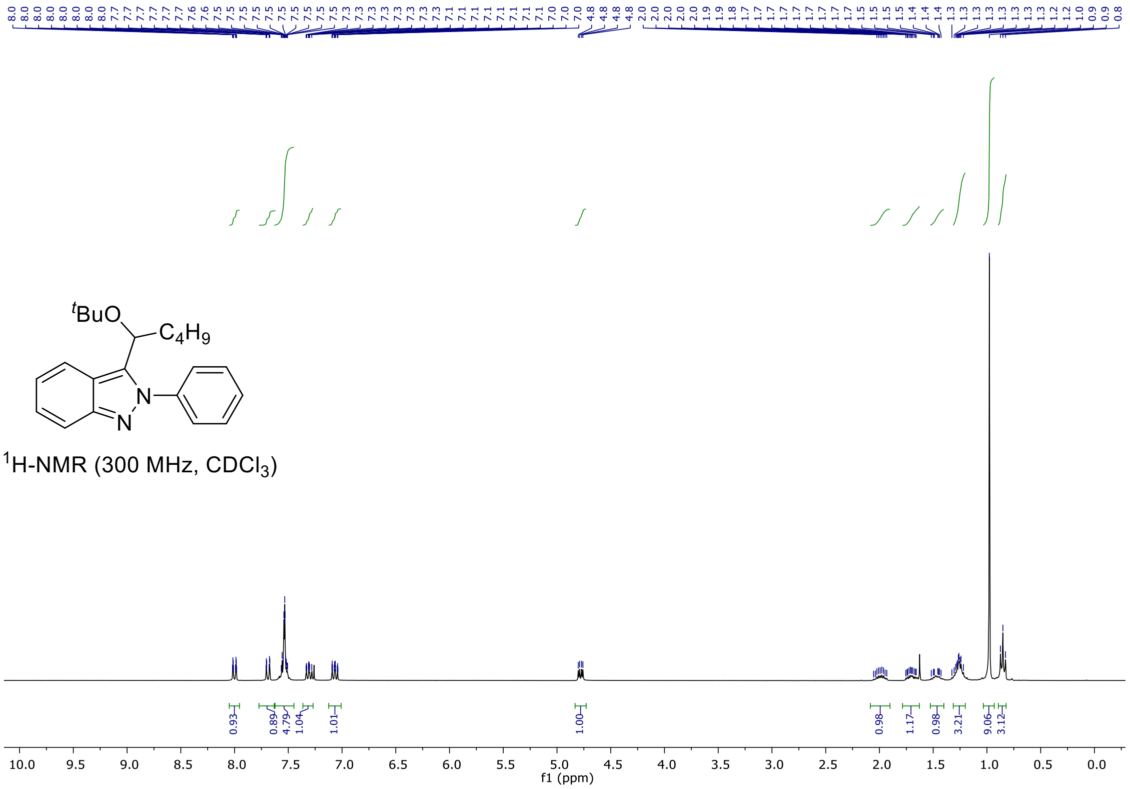

^{1}H-NMR (300 MHz, $CDCl_3$)

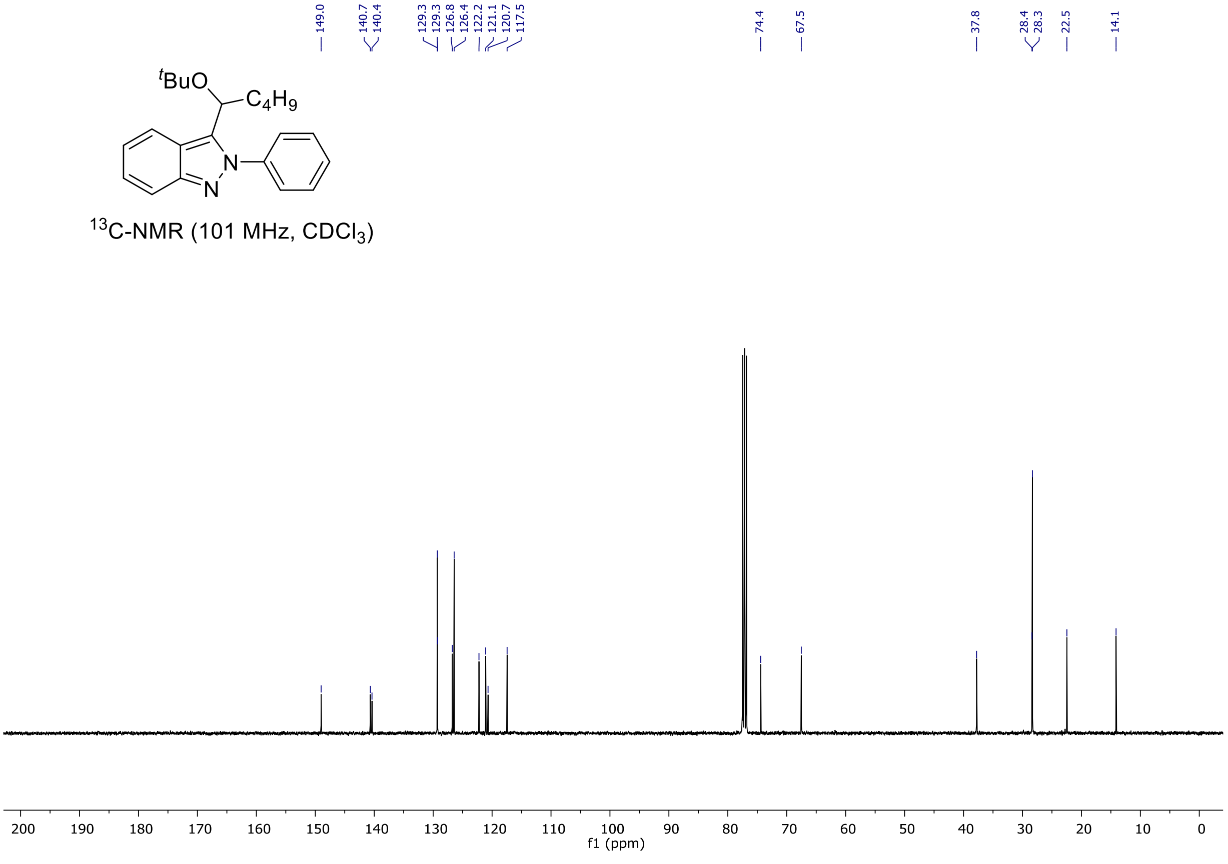

^{13}C-NMR (101 MHz, $CDCl_3$)

3-(1-Isopropoxypentyl)-2-phenyl-2*H*-indazole (2ag)

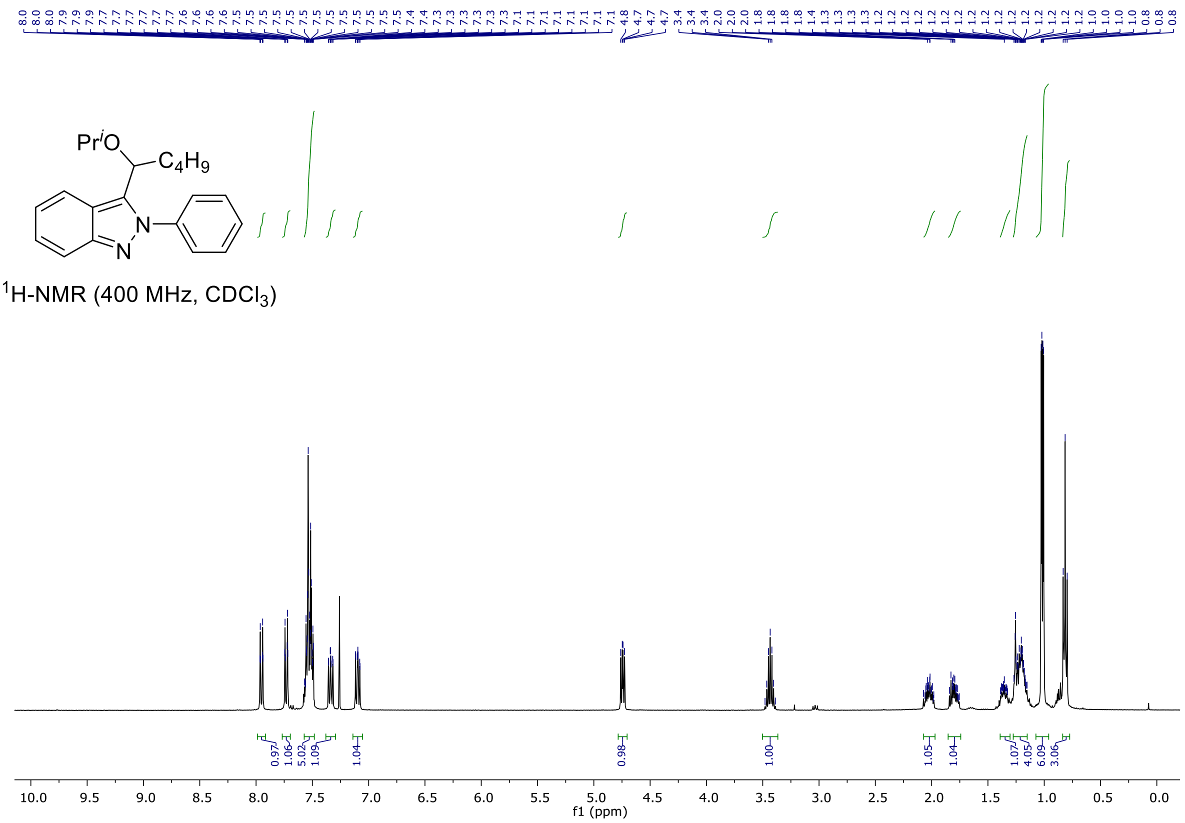

^{1}H-NMR (400 MHz, $CDCl_3$)

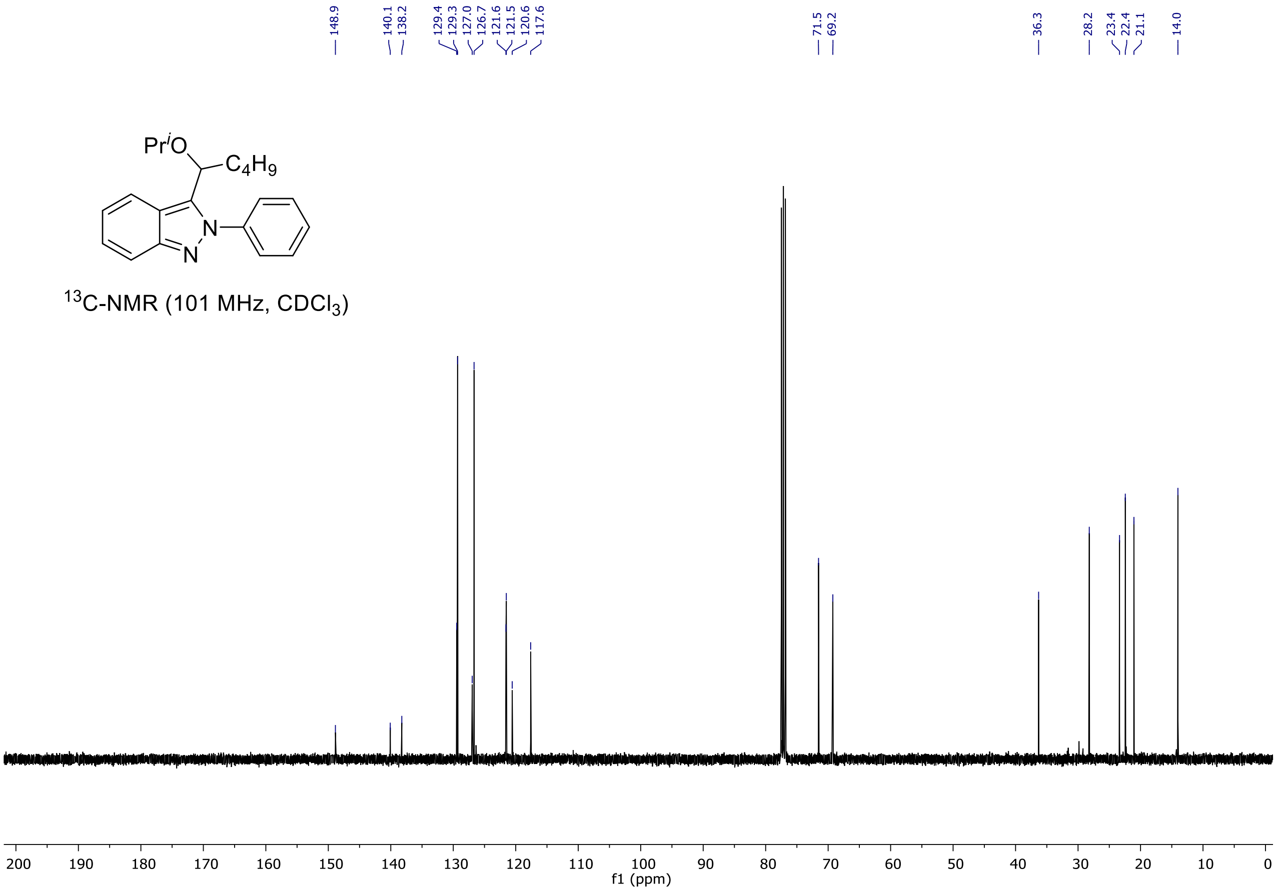

^{13}C-NMR (101 MHz, $CDCl_3$)

3-[1-(But-3-en-1-yloxy)pentyl]-2-phenyl-2*H*-indazole (2ah)

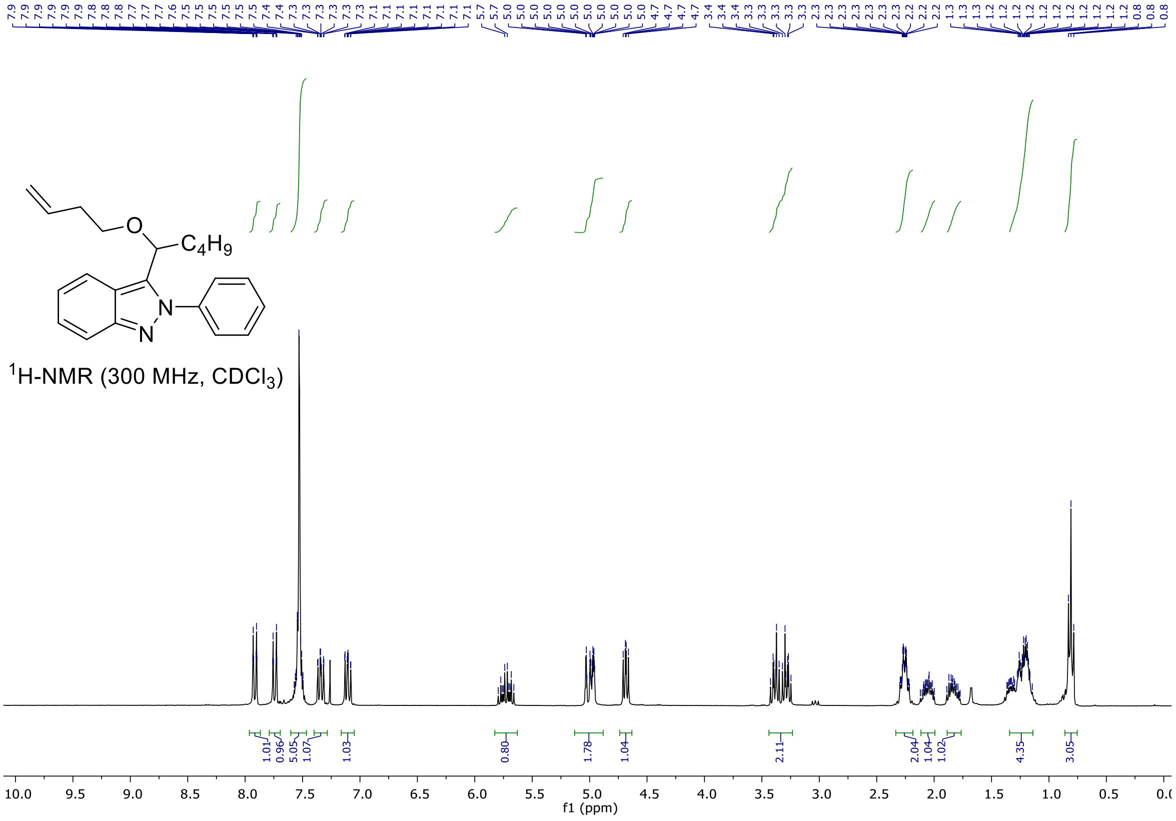

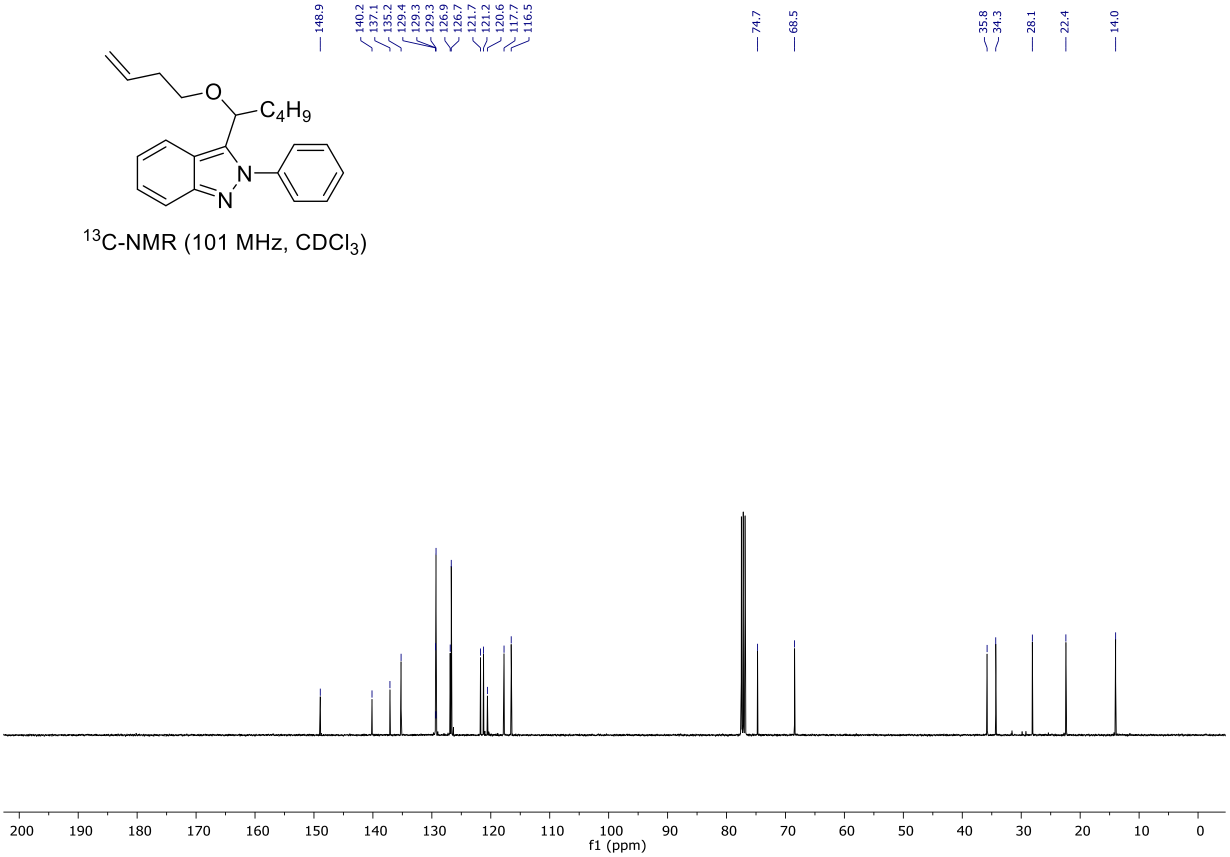

2-Phenyl-3-[1-(prop-2-yn-1-yloxy)pentyl]-2*H*-indazole (2ai)

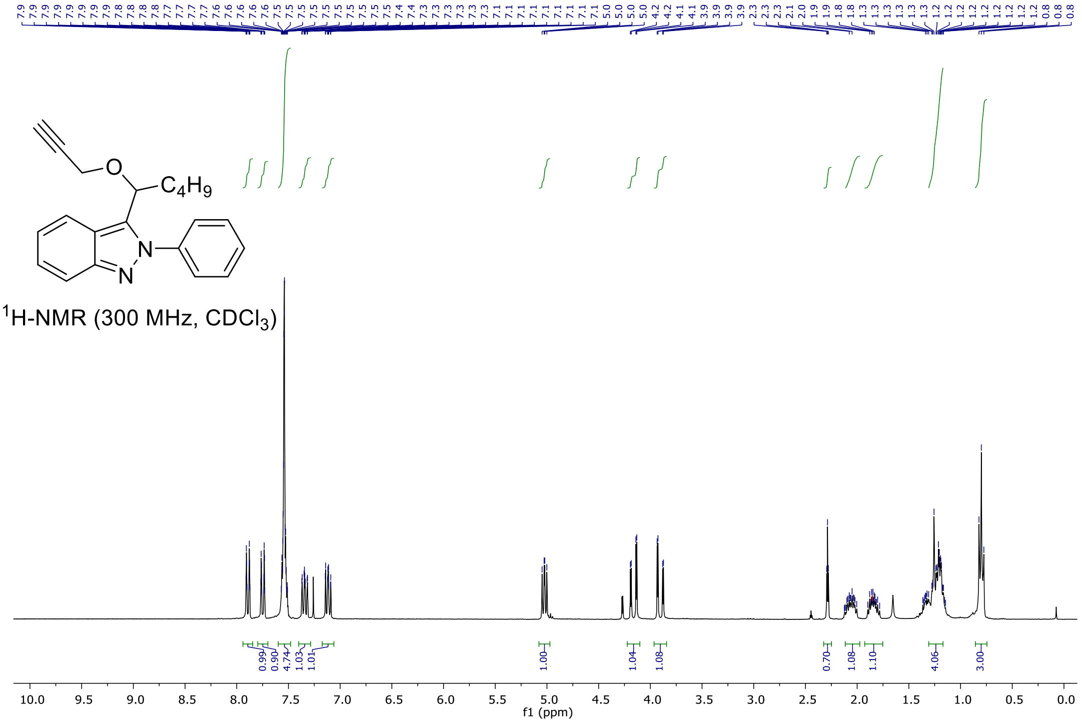

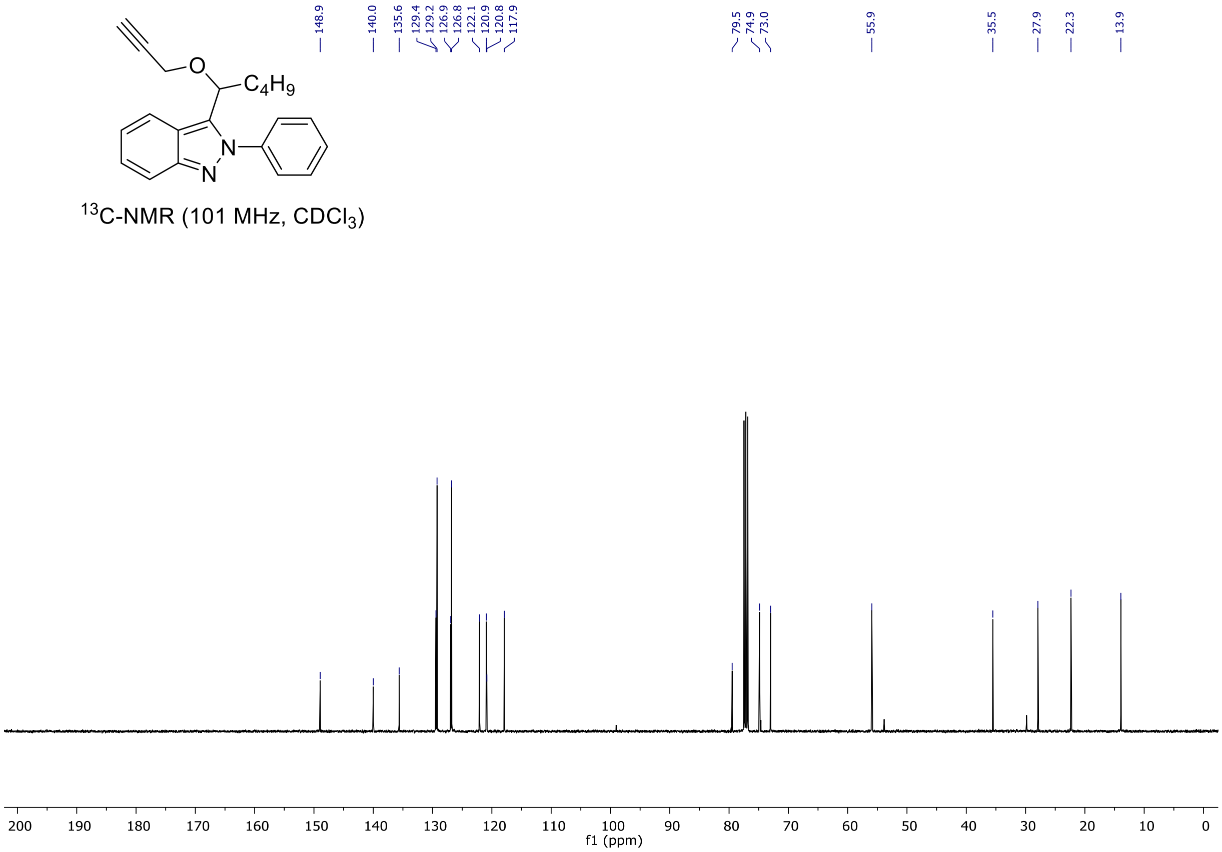

3-[1-(2-Fluoroethoxy)pentyl]-2-phenyl-2*H*-indazole (2aj)

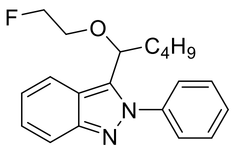

^{1}H-NMR (300 MHz, CDCl$_3$)

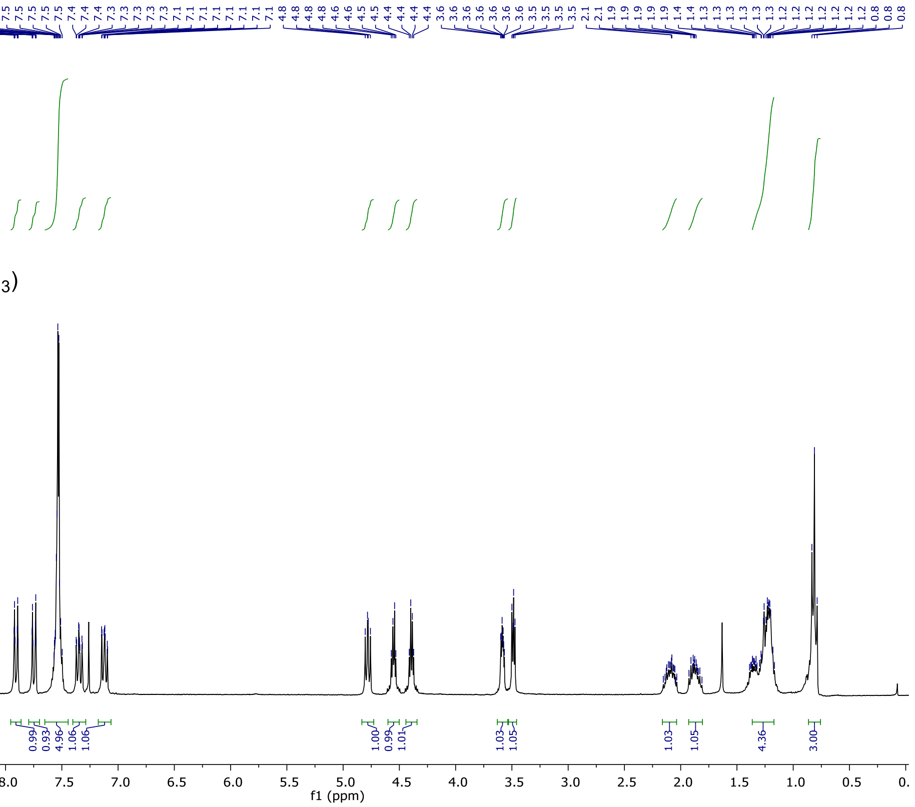

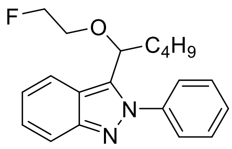

^{19}F-NMR (282 MHz, CDCl$_3$)

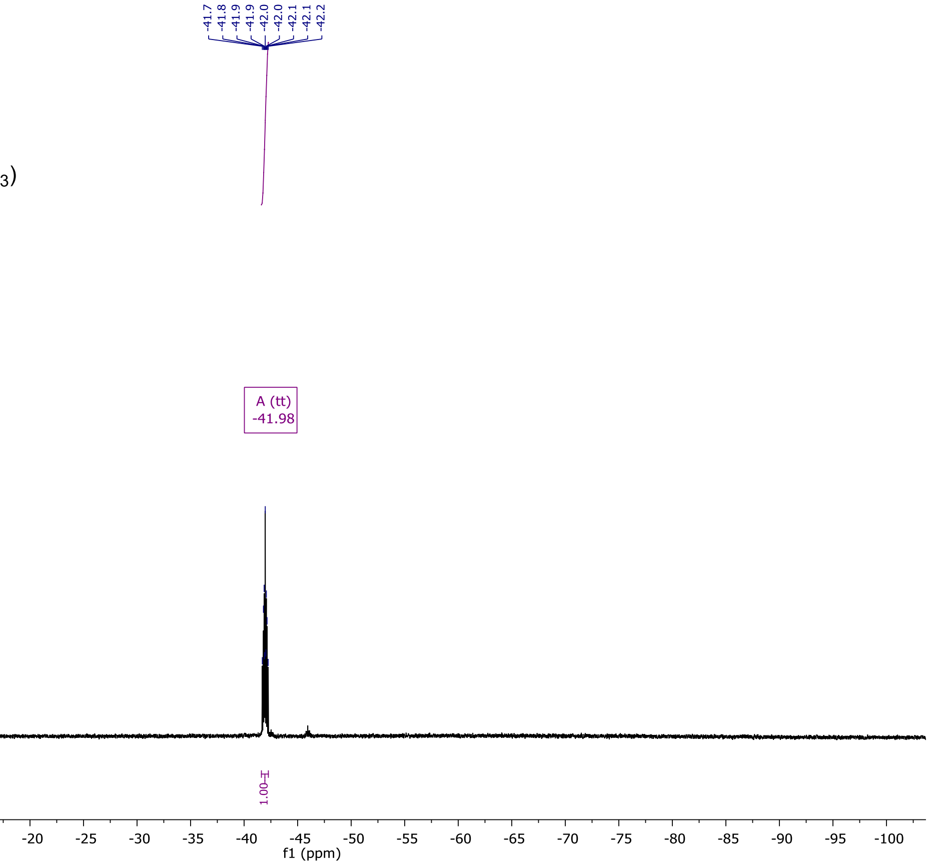

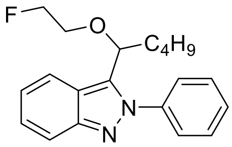

^{13}C-NMR (101 MHz, $CDCl_3$)

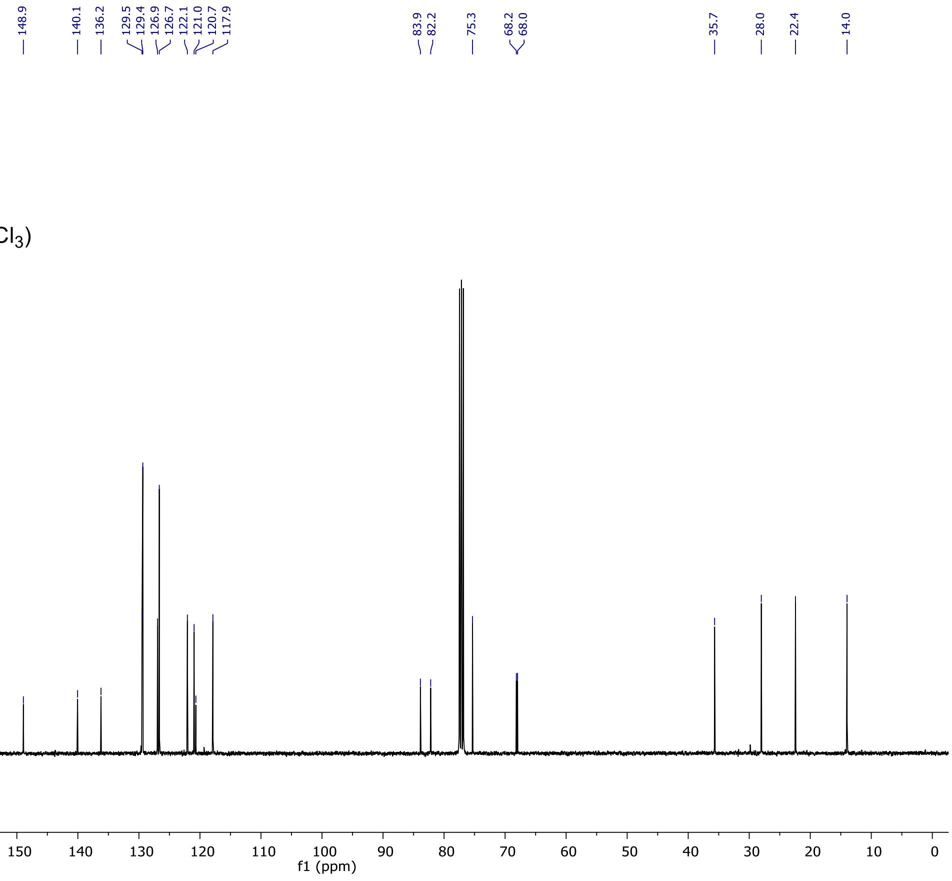

3-[1-(3-Iodopropoxy)pentyl]-2-phenyl-2*H*-indazole (2ak)

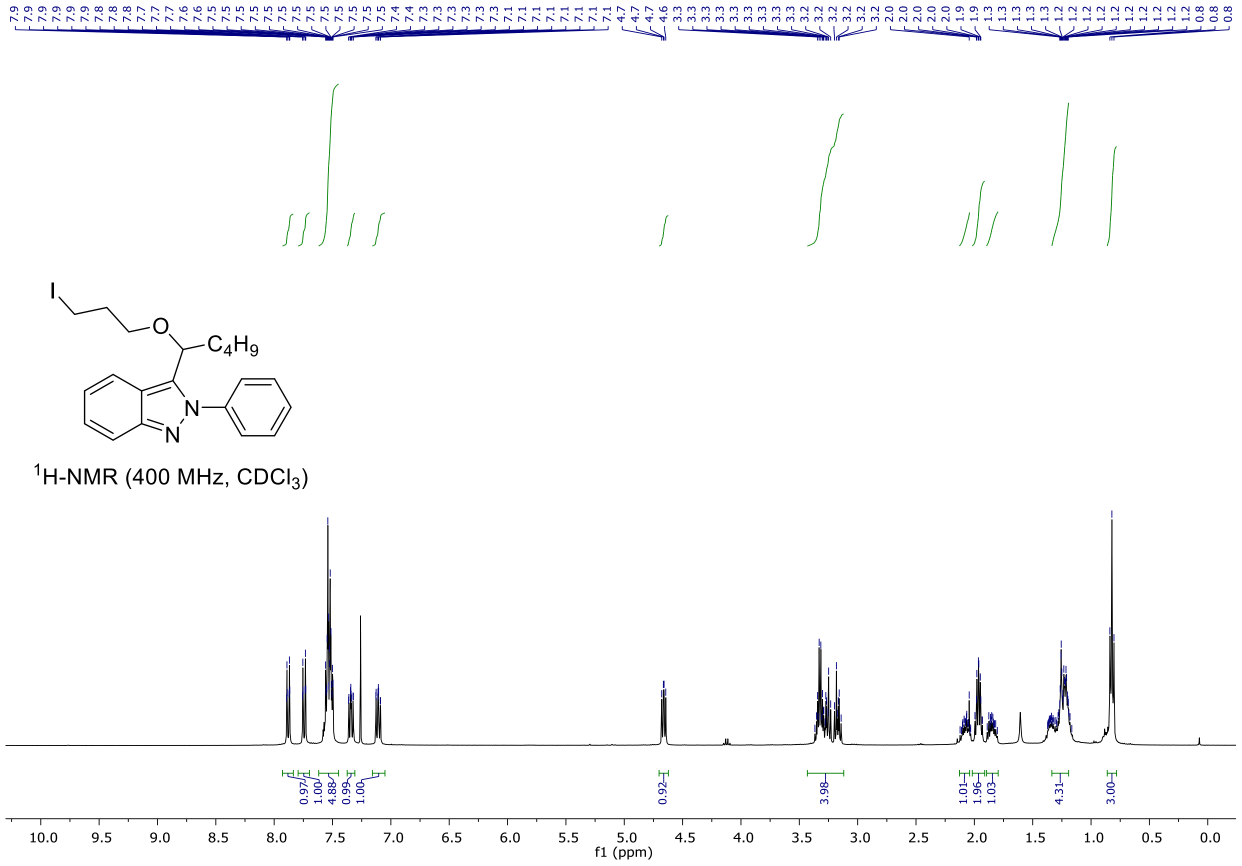

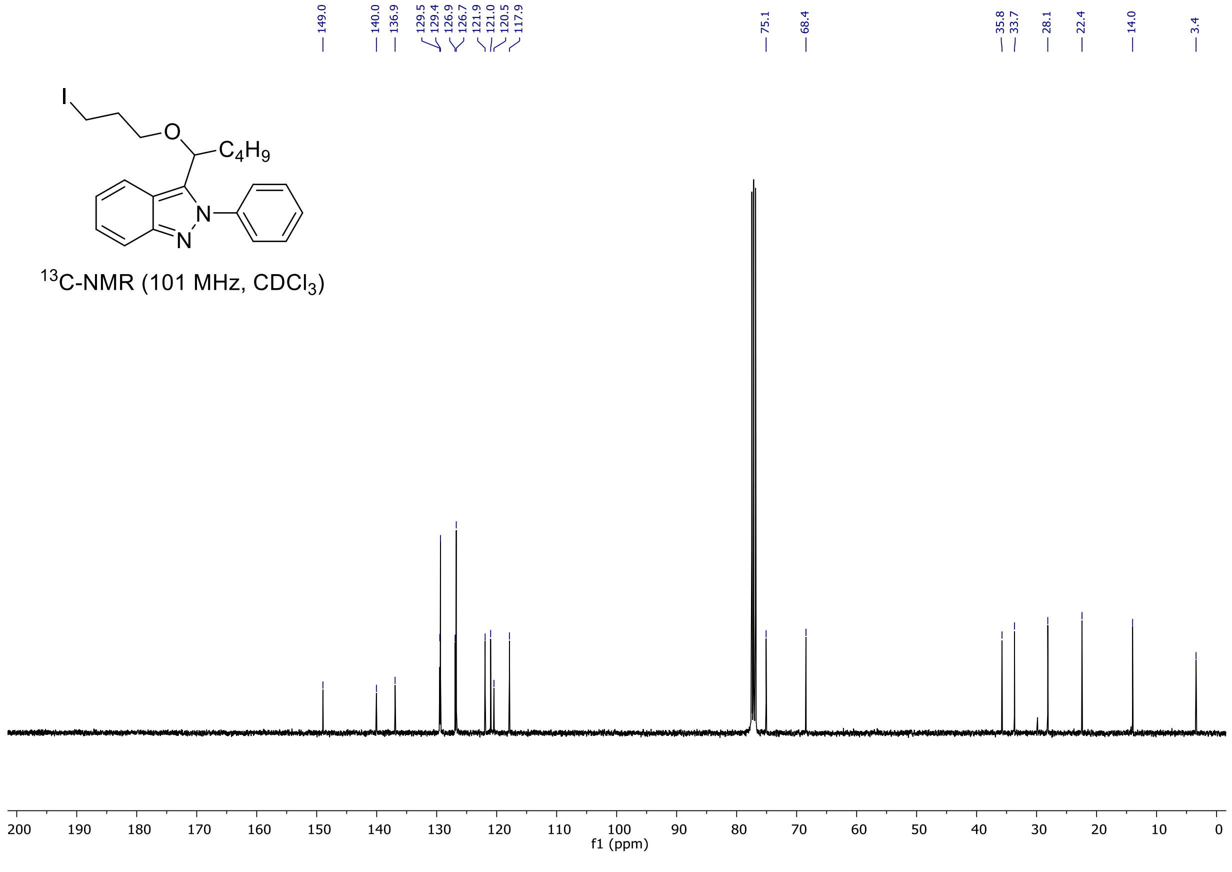

3-(1-Phenoxypentyl)-2-phenyl-2*H*-indazole (2al)

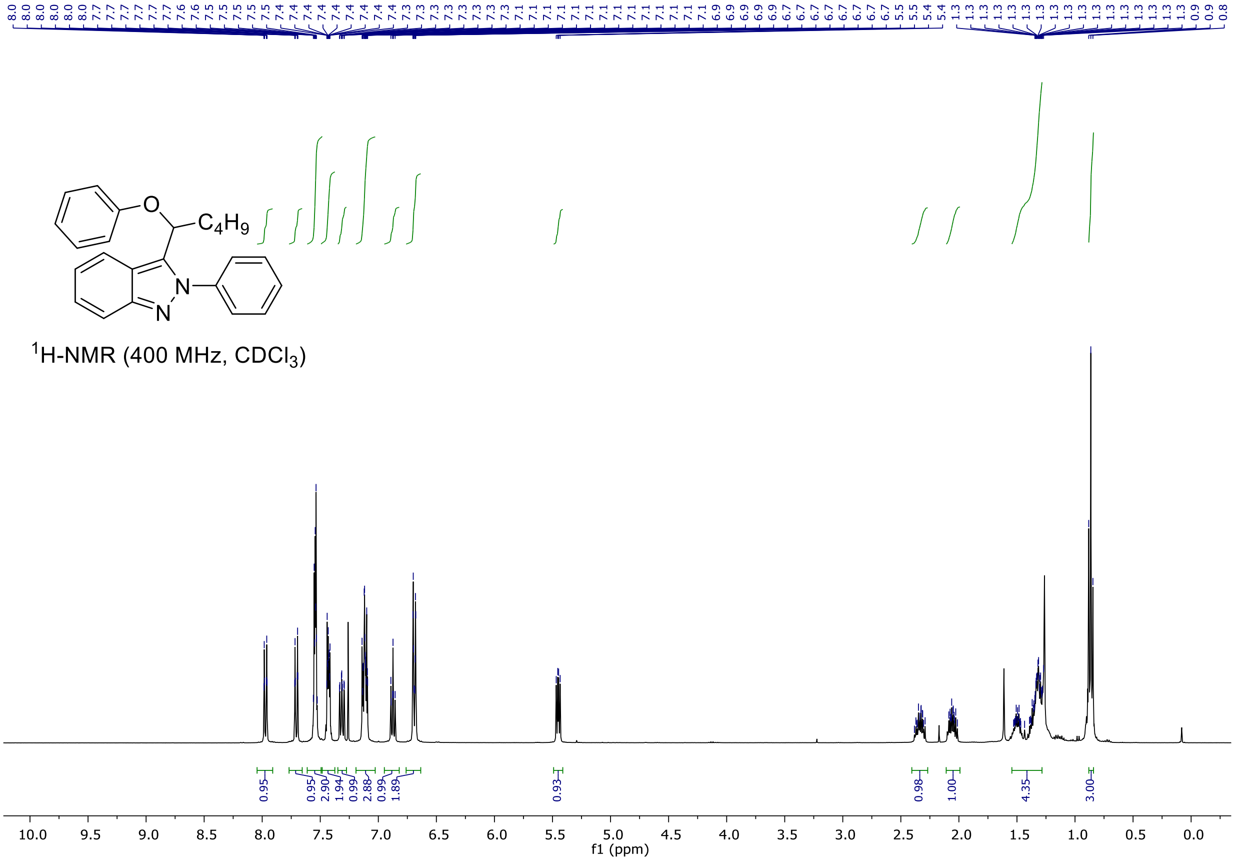

^{1}H-NMR (400 MHz, $CDCl_3$)

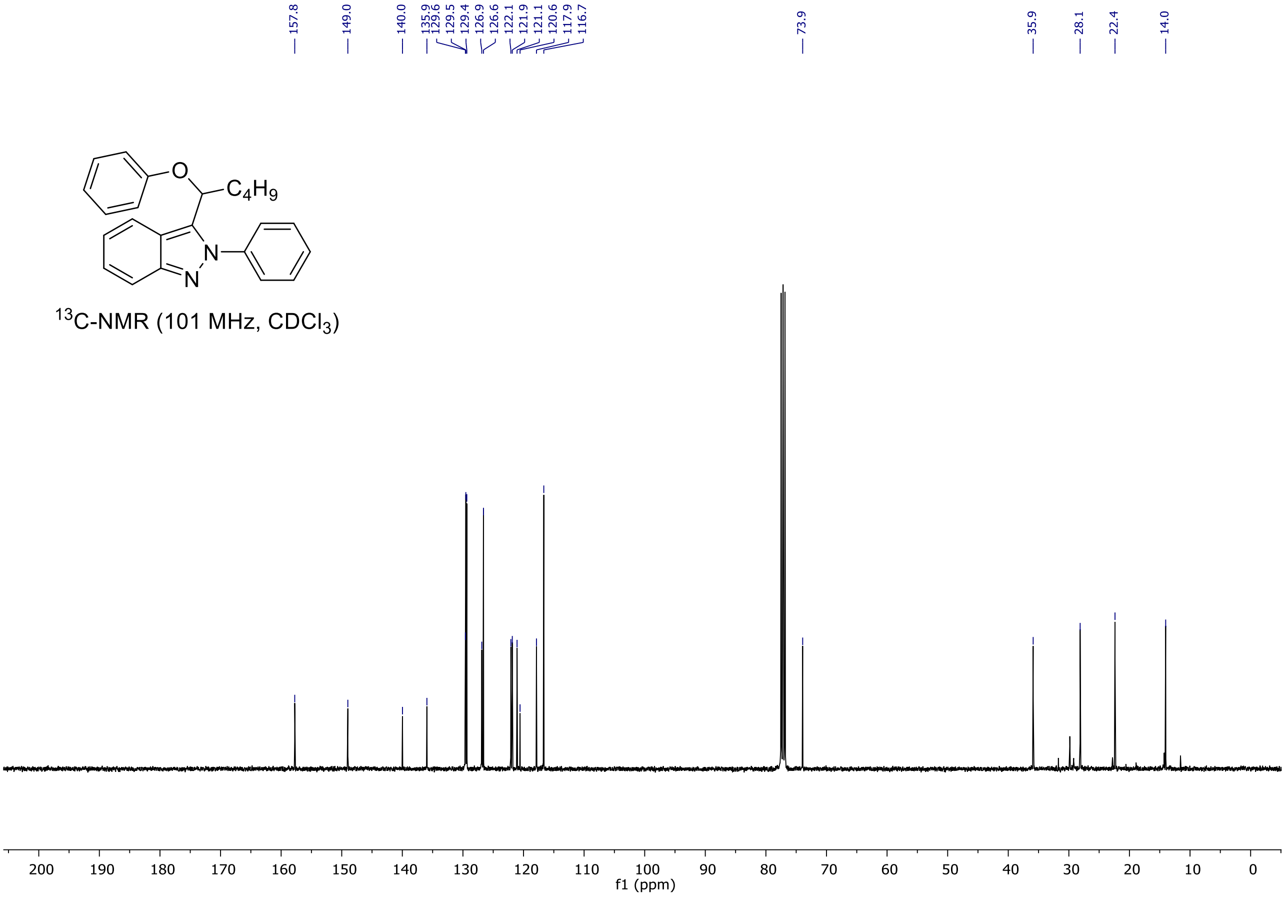

^{13}C-NMR (101 MHz, $CDCl_3$)

3-[1-((1S,2S,4R)-Bicyclo[2.2.1]heptan-2-yl)methoxy)pentyl]-2-phenyl-2H-indazole y 3-[1-((1S,2R,4R)-Bicyclo[2.2.1]heptan-2-yl)methoxy)pentyl]-2-phenyl-2H-indazole (2am)

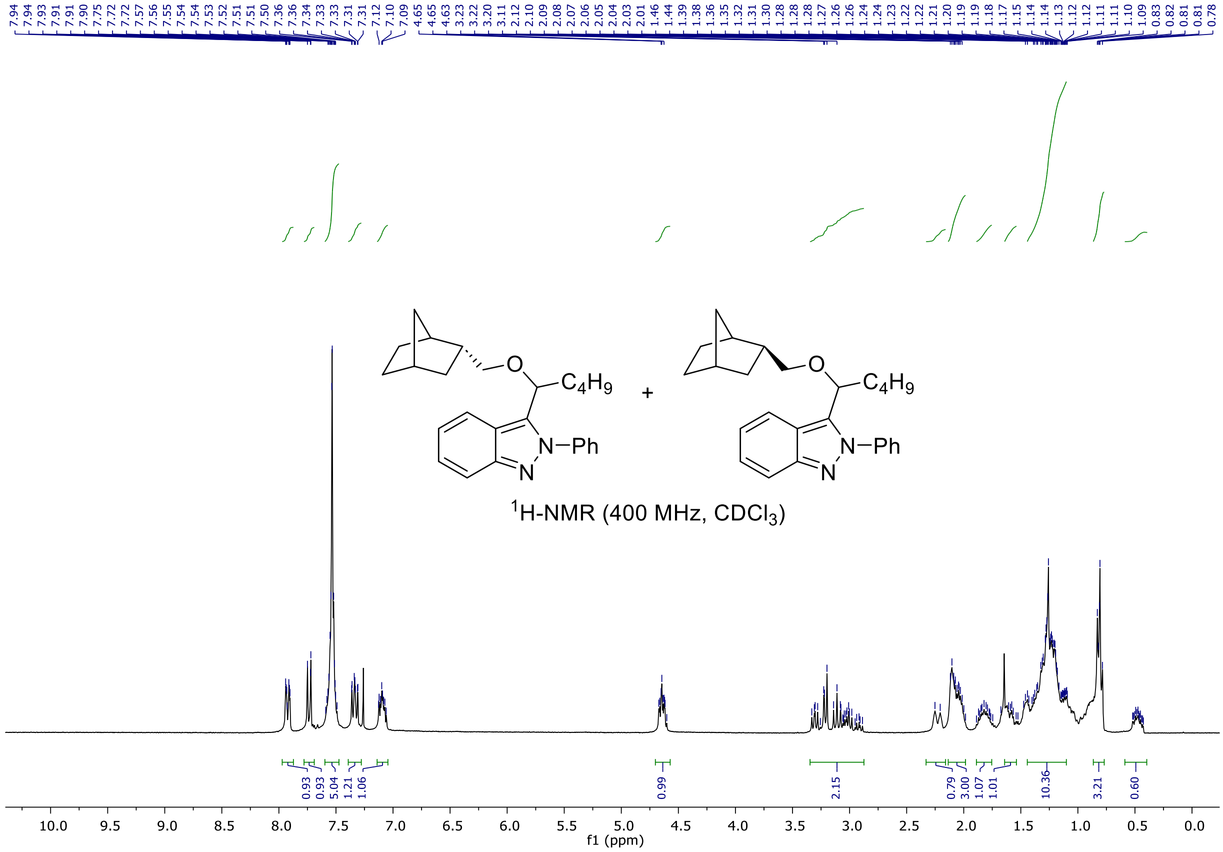

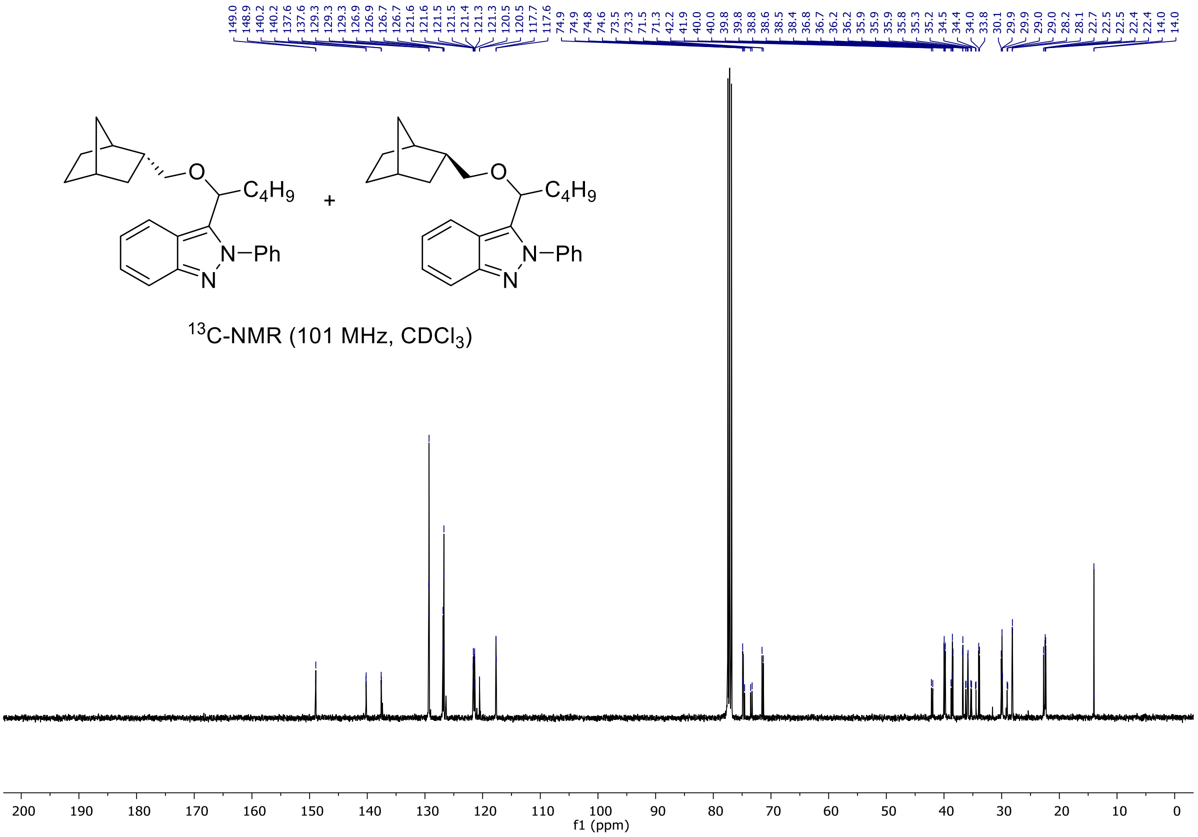

3-[1-(2-((3*R*,5*R*,7*R*)-Adamantan-1-yl)methoxy)pentyl]-2-phenyl-2*H*-indazole (2an)

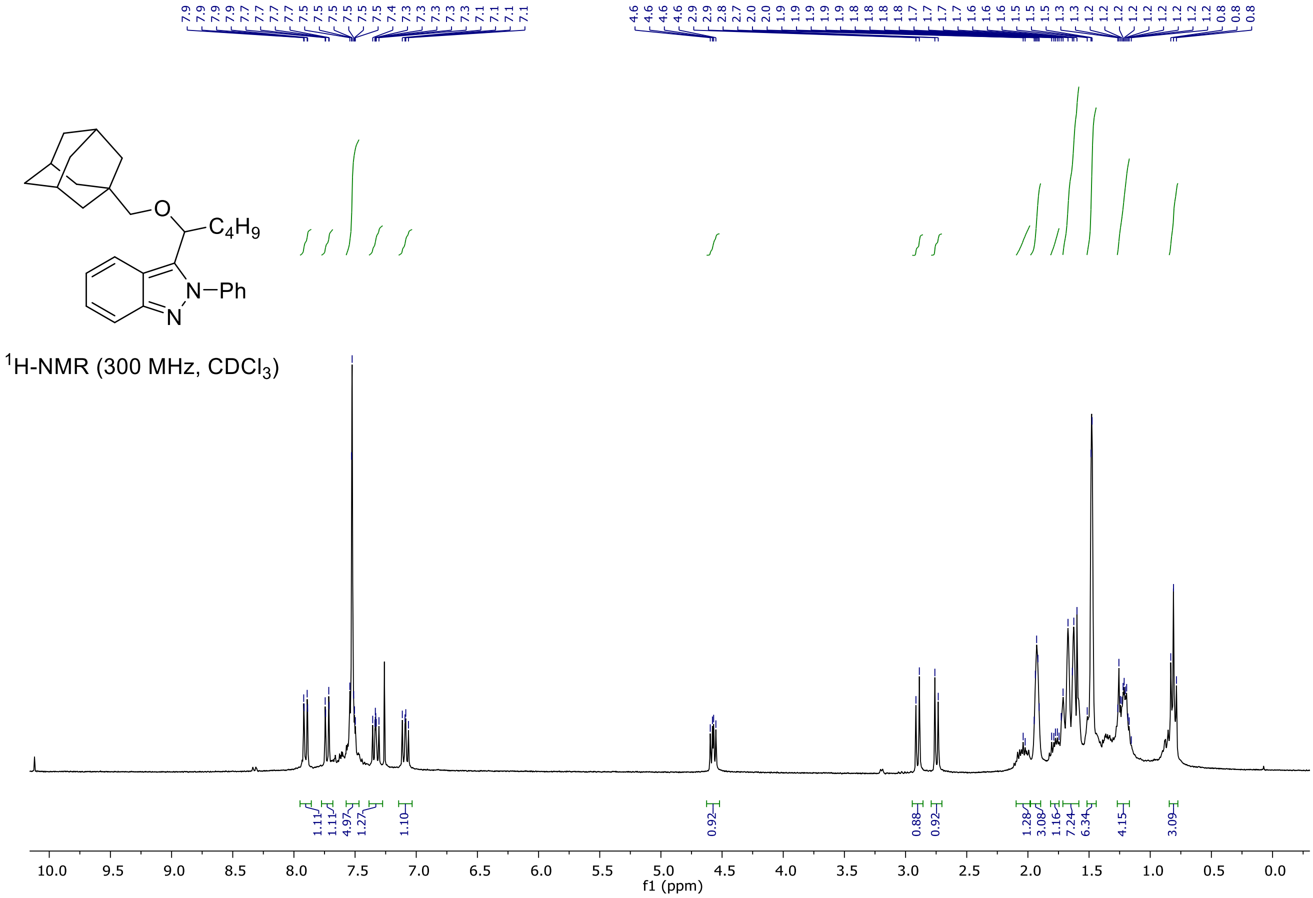

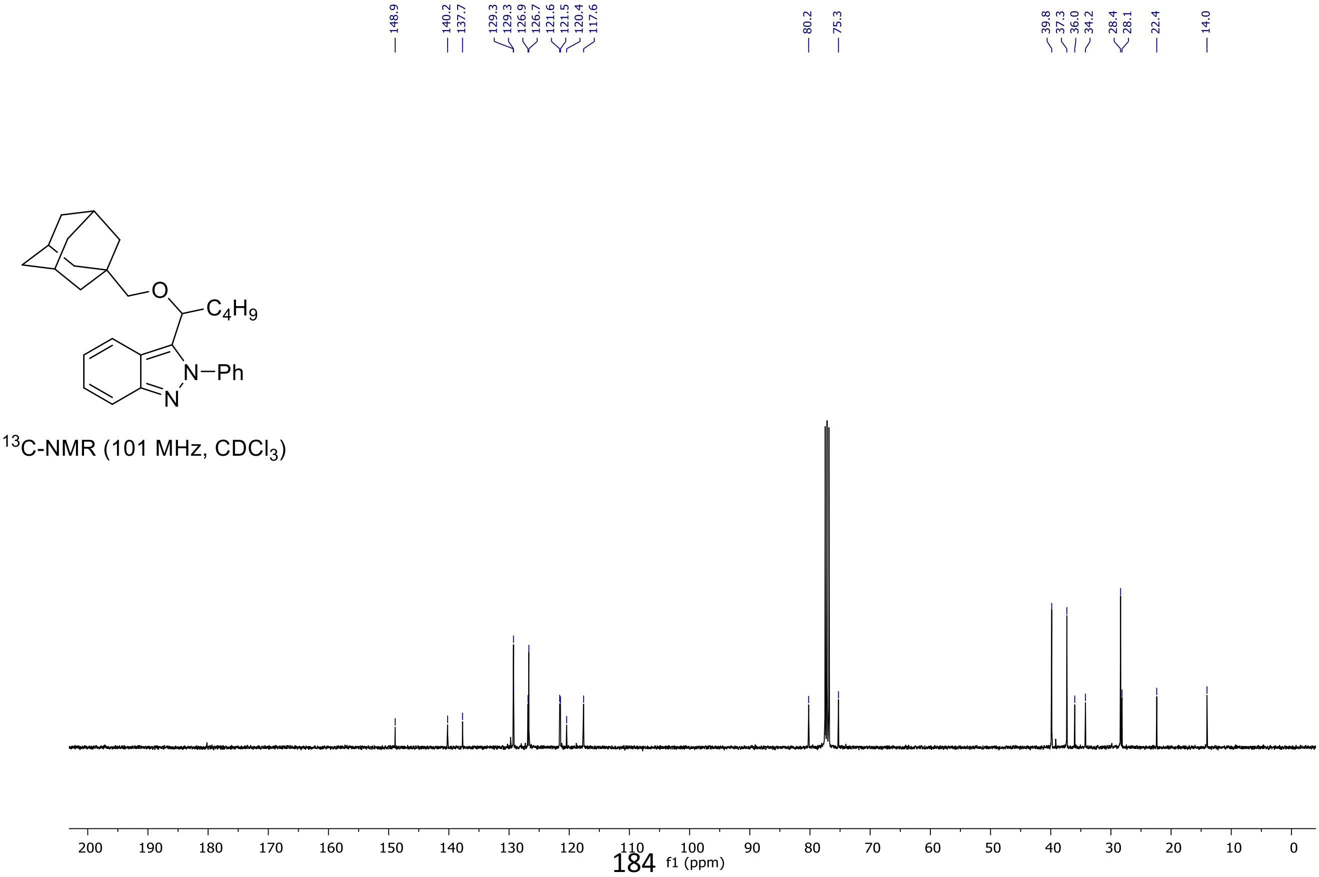

2-Phenyl-3-[(*S*)-1-(3-(((*R*)-2,5,7,8-tetramethyl-2-((4*R*,8*R*)-4,8,12-trimethyltridecyl)chroman-6-yl)oxy)propoxy)pentyl]-2*H*-indazole and 2-phenyl-3-[(*R*)-1-(3-(((*R*)-2,5,7,8-tetramethyl-2-((4*R*,8*R*)-4,8,12-trimethyltridecyl)chroman-6-yl)oxy)propoxy)pentyl]-2*H*-indazole (2ao)

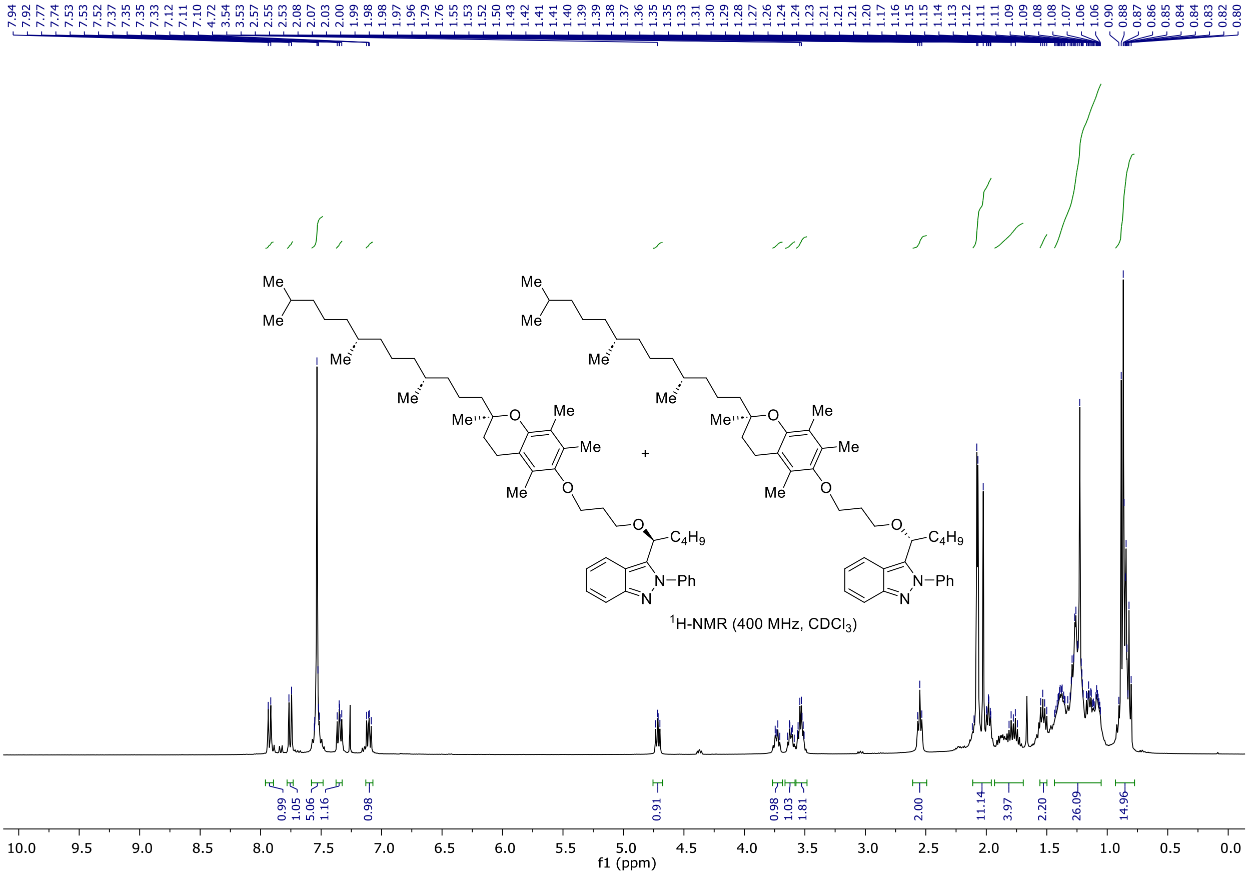

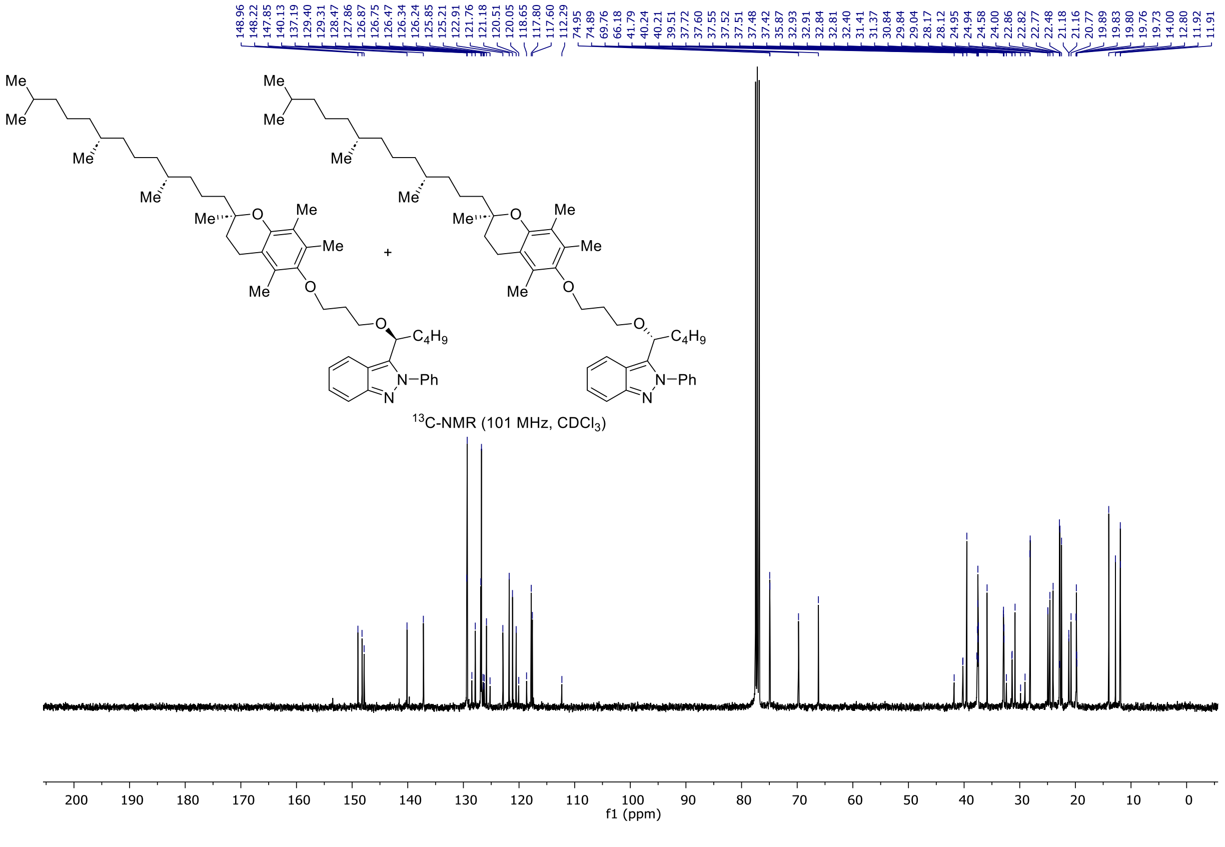

(8*R*,9*S*,13*S*,14*S*)-13-Methyl-3-[3-((1-(2-phenyl-2*H*-indazol-3-yl)pentyl)oxy)propoxy]-6,7,8,9,11,12,13,14,15,16-decahydro-17*H*-cyclopenta[*a*]phenanthren-17-one (2ap)

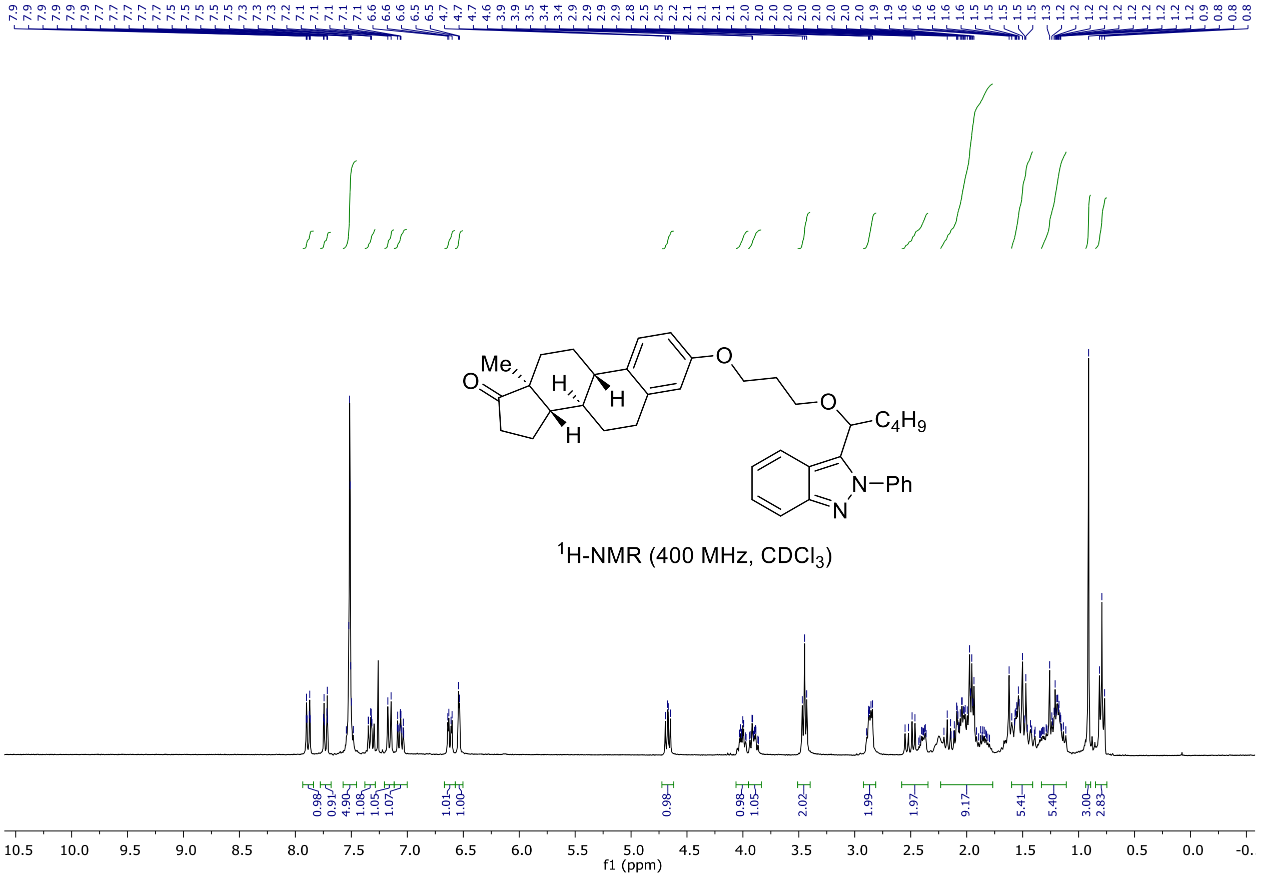

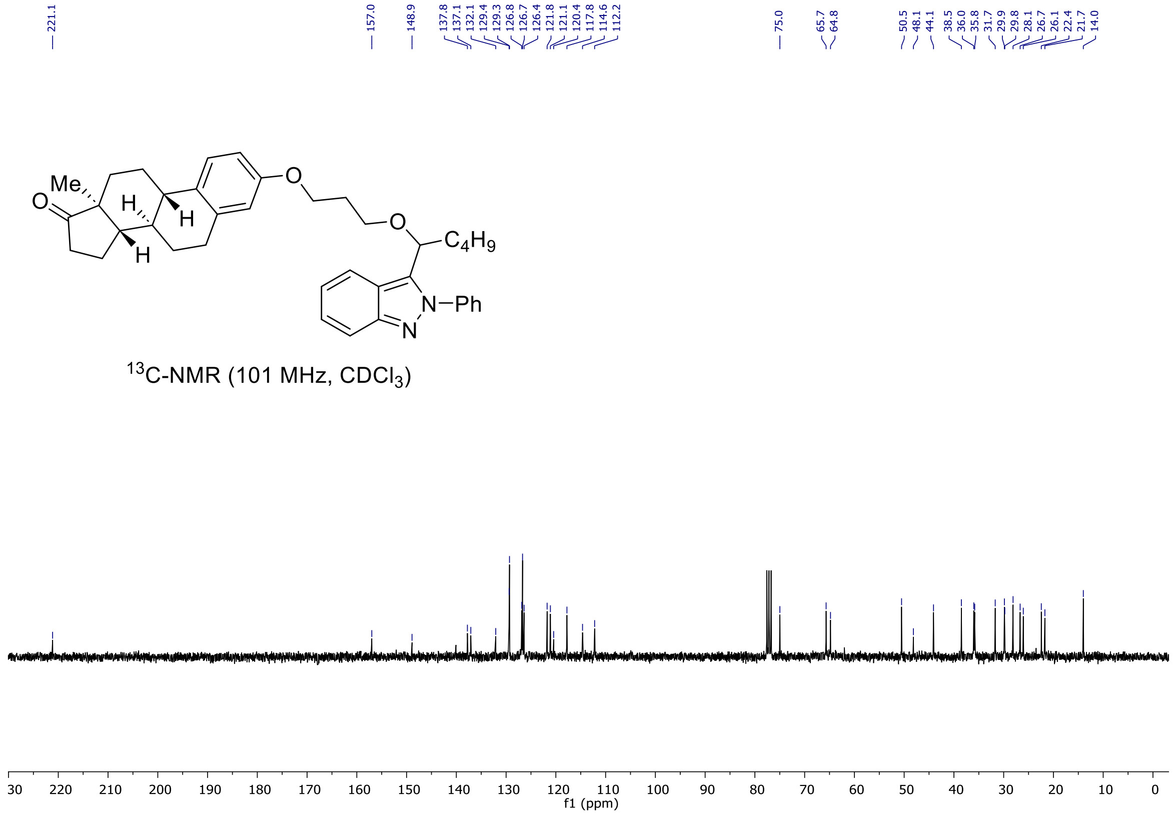

1-(2-Phenyl-2*H*-indazol-3-yl)pentan-1-ol (2aq)

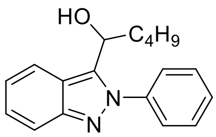

^{1}H-NMR (300 MHz, $CDCl_3$)

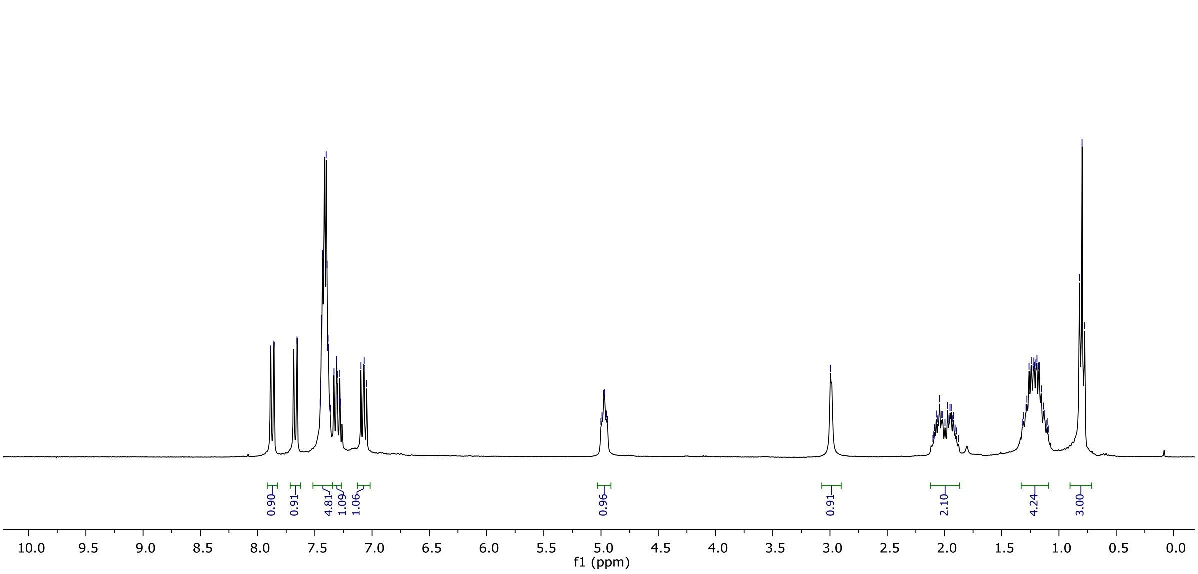

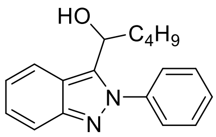

^{13}C-NMR (75 MHz, $CDCl_3$)

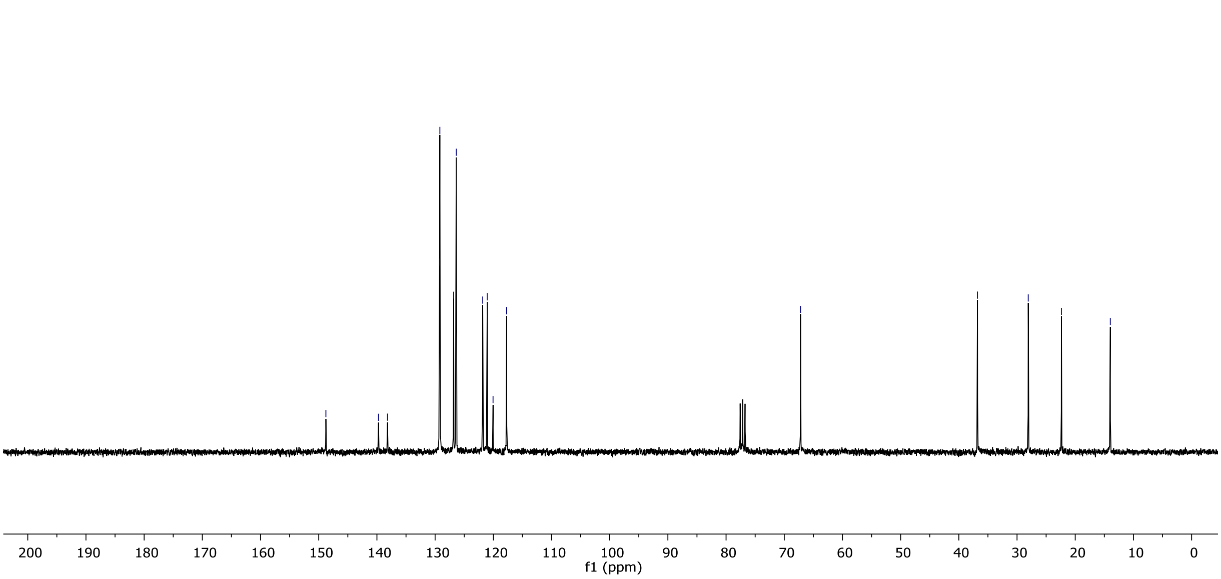

1-(2-Phenyl-2*H*-indazol-3-yl)pentyl hexanoate (2ar)

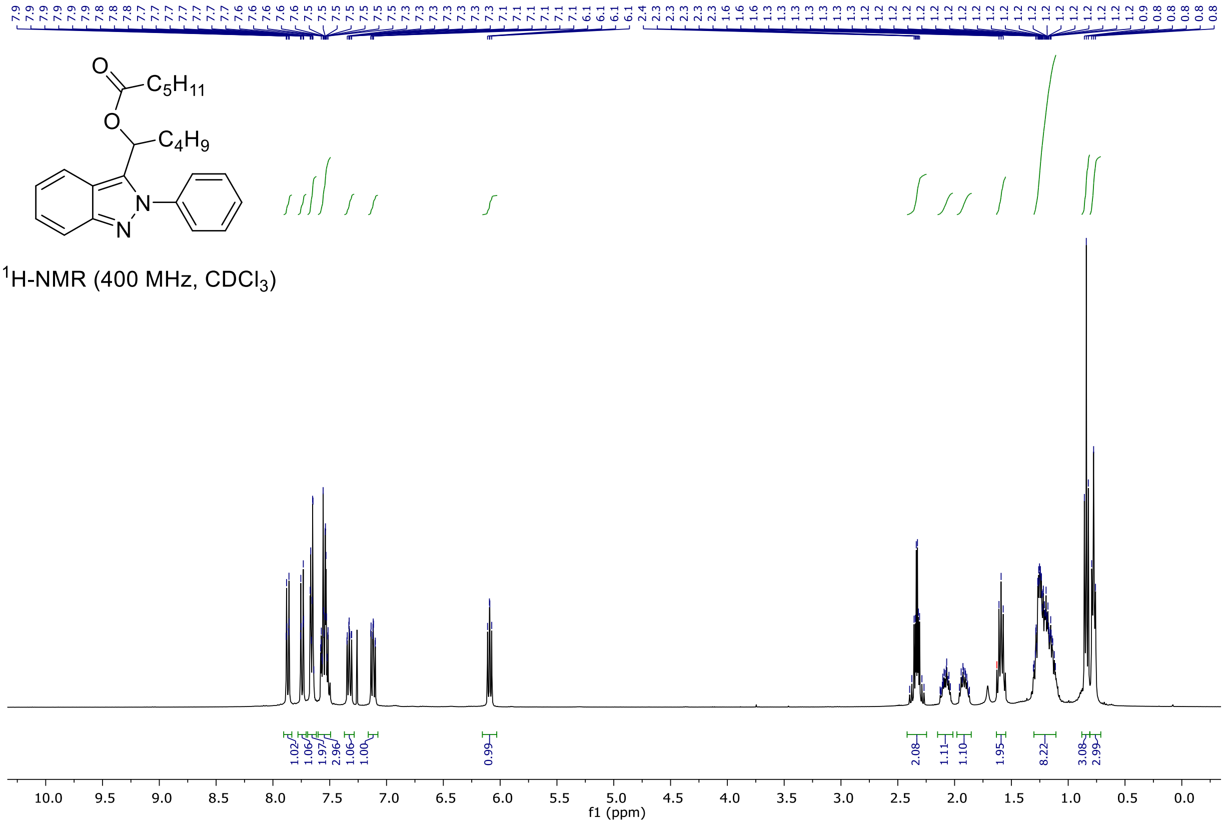

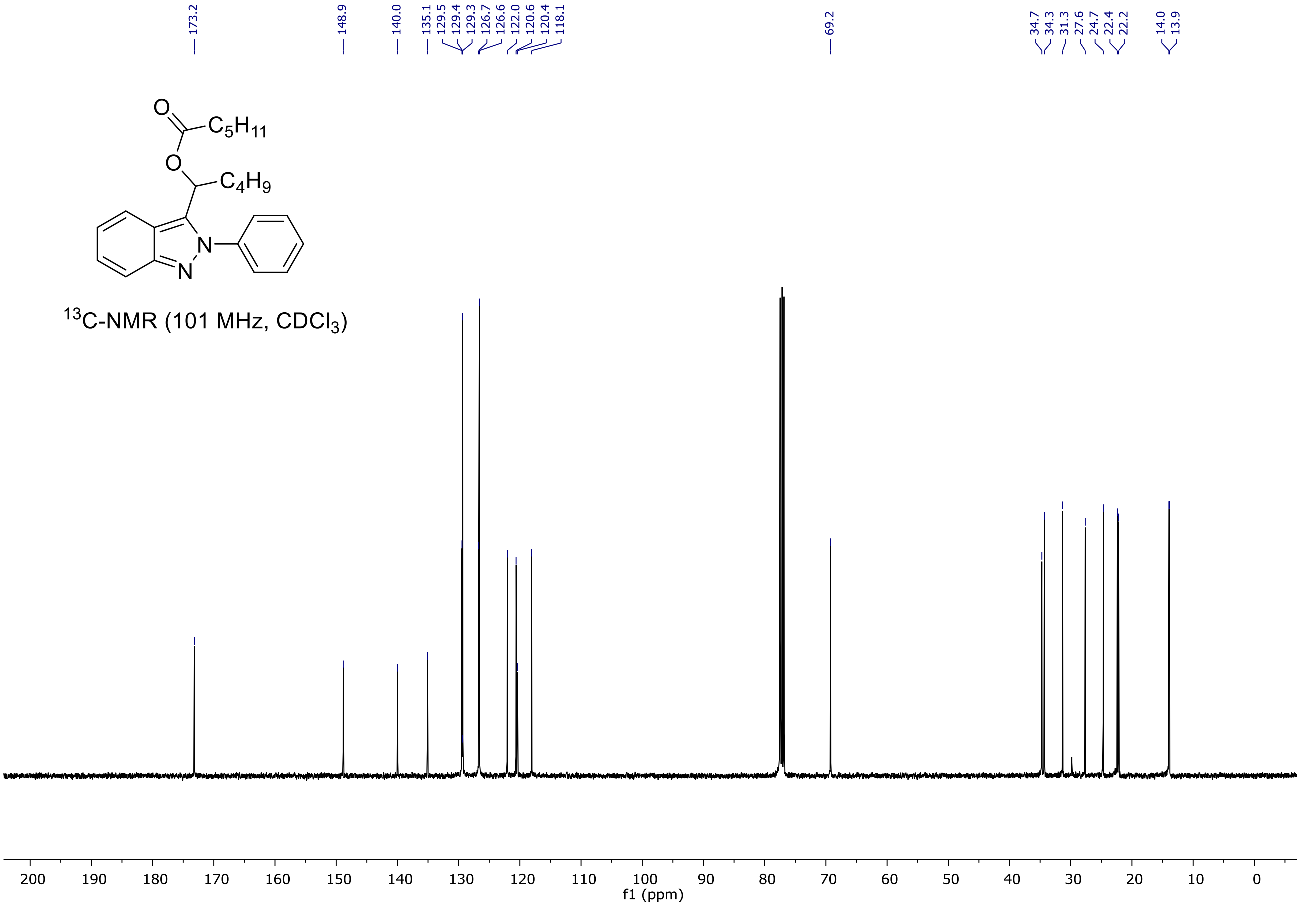

1-(2-Phenyl-2*H*-indazol-3-yl)pentyl benzoate (2as)

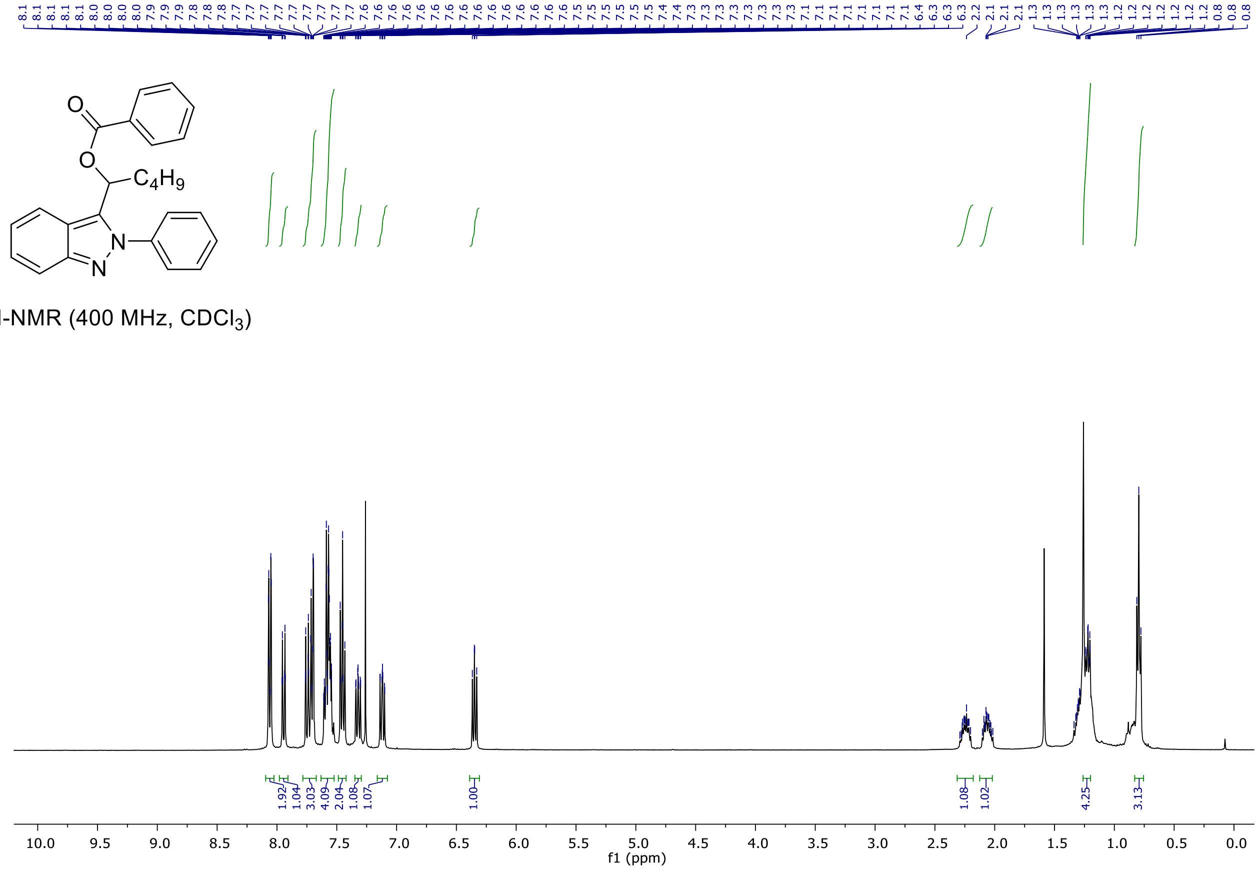

^{1}H-NMR (400 MHz, $CDCl_3$)

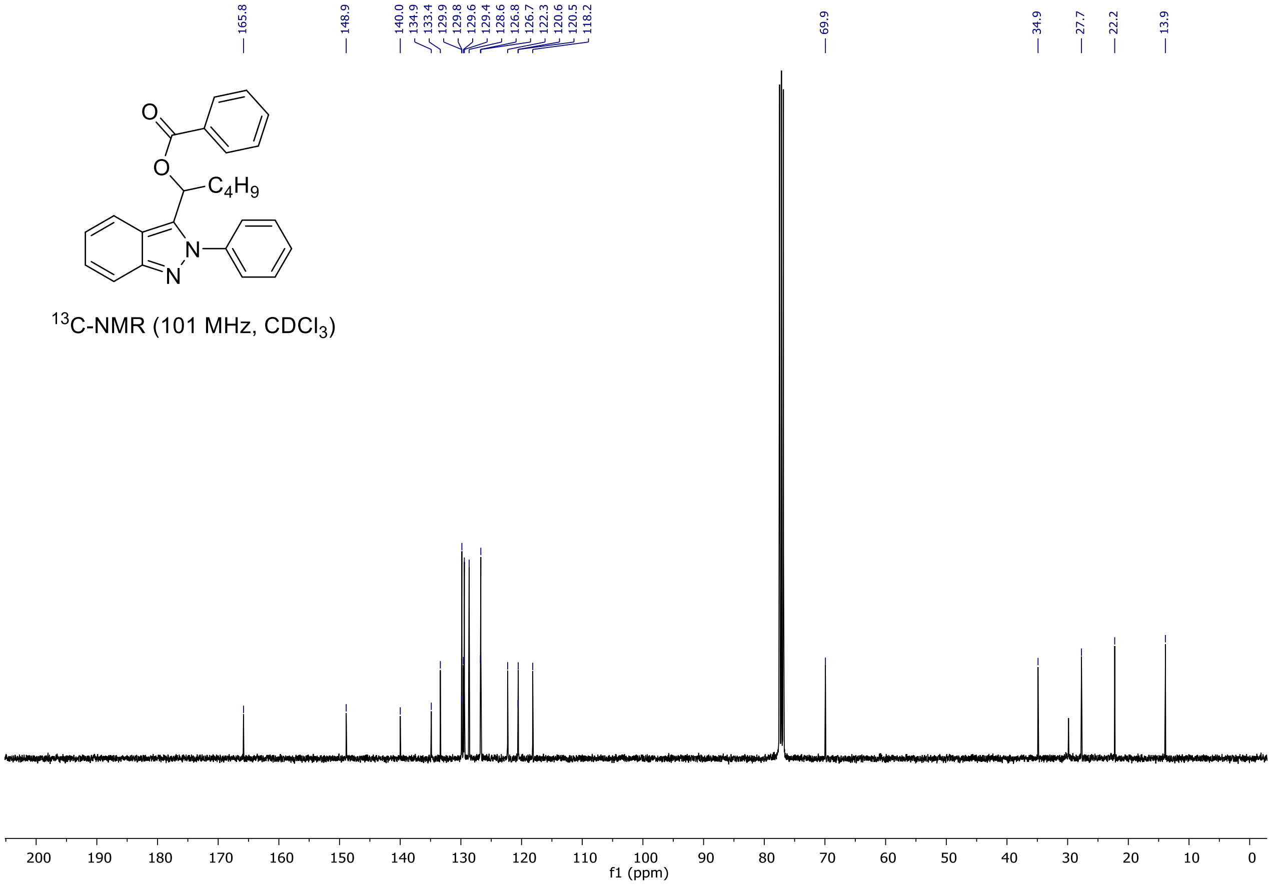

^{13}C-NMR (101 MHz, $CDCl_3$)

1-(2-Phenyl-2*H*-indazol-3-yl)pentyl 1-methylcyclohexane-1-carboxylate (2at)

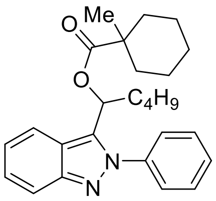

^{1}H-NMR (300 MHz, $CDCl_3$)

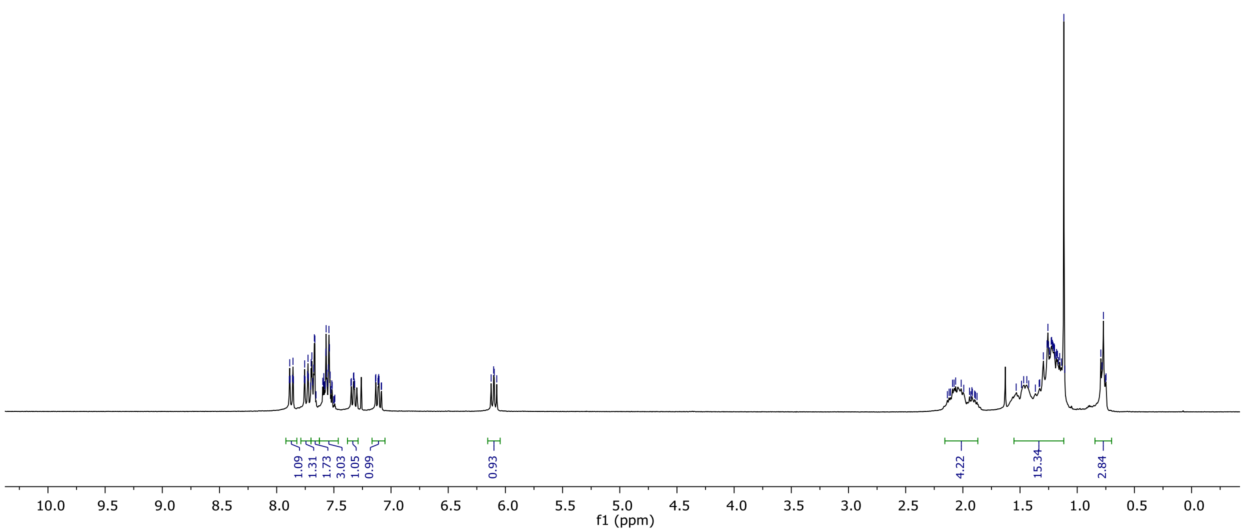

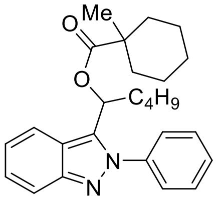

^{13}C-NMR (101 MHz, $CDCl_3$)

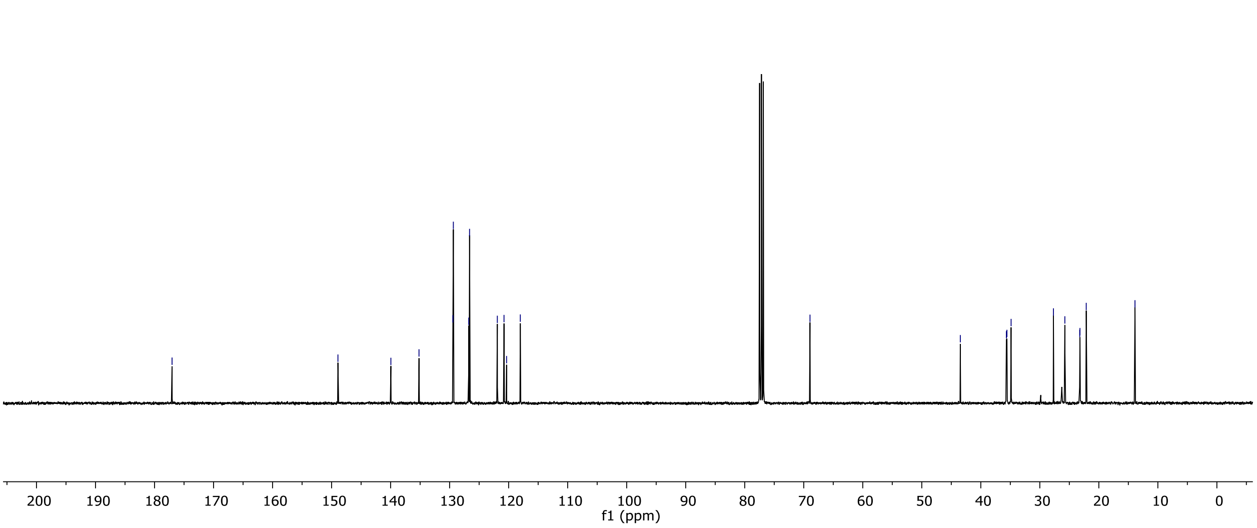

1-(2-Phenyl-2*H*-indazol-3-yl)pentyl (3r,5r,7r)-adamantane-1-carboxylate (2au)

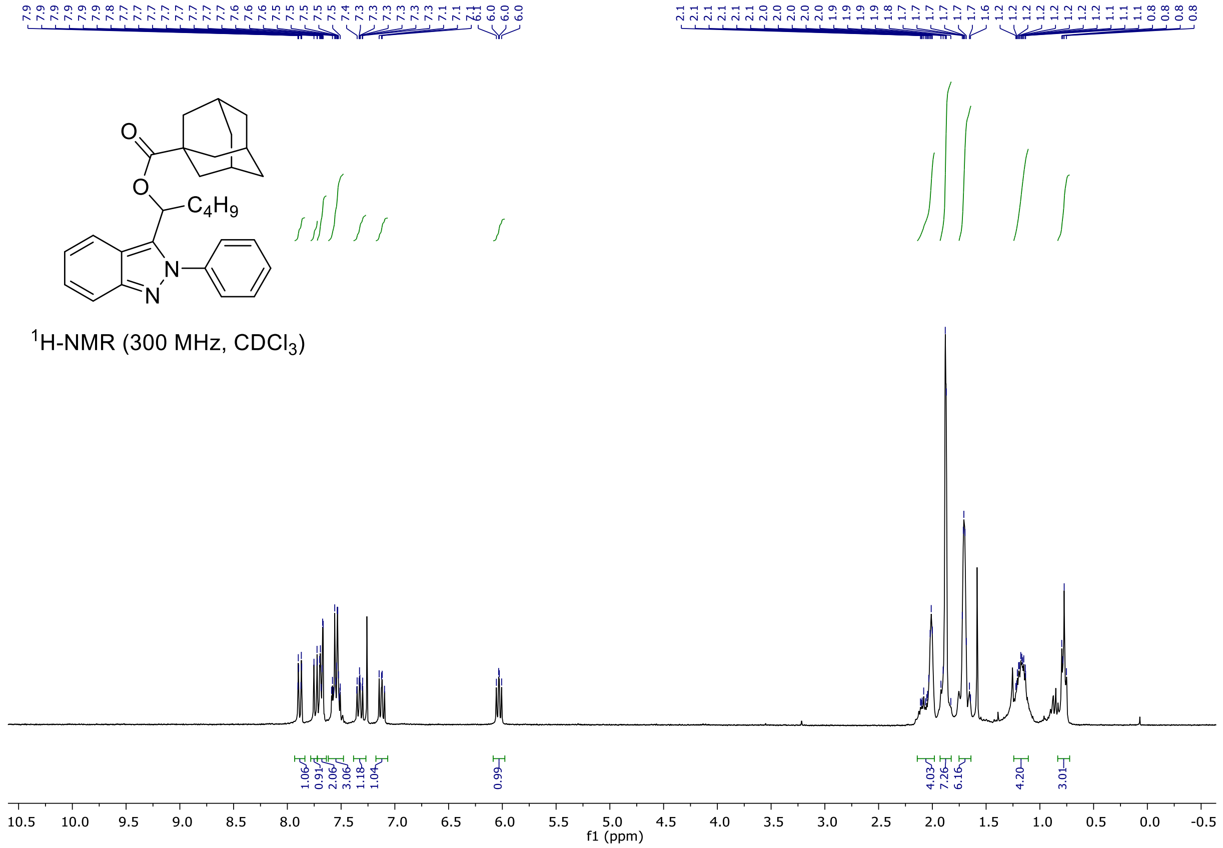

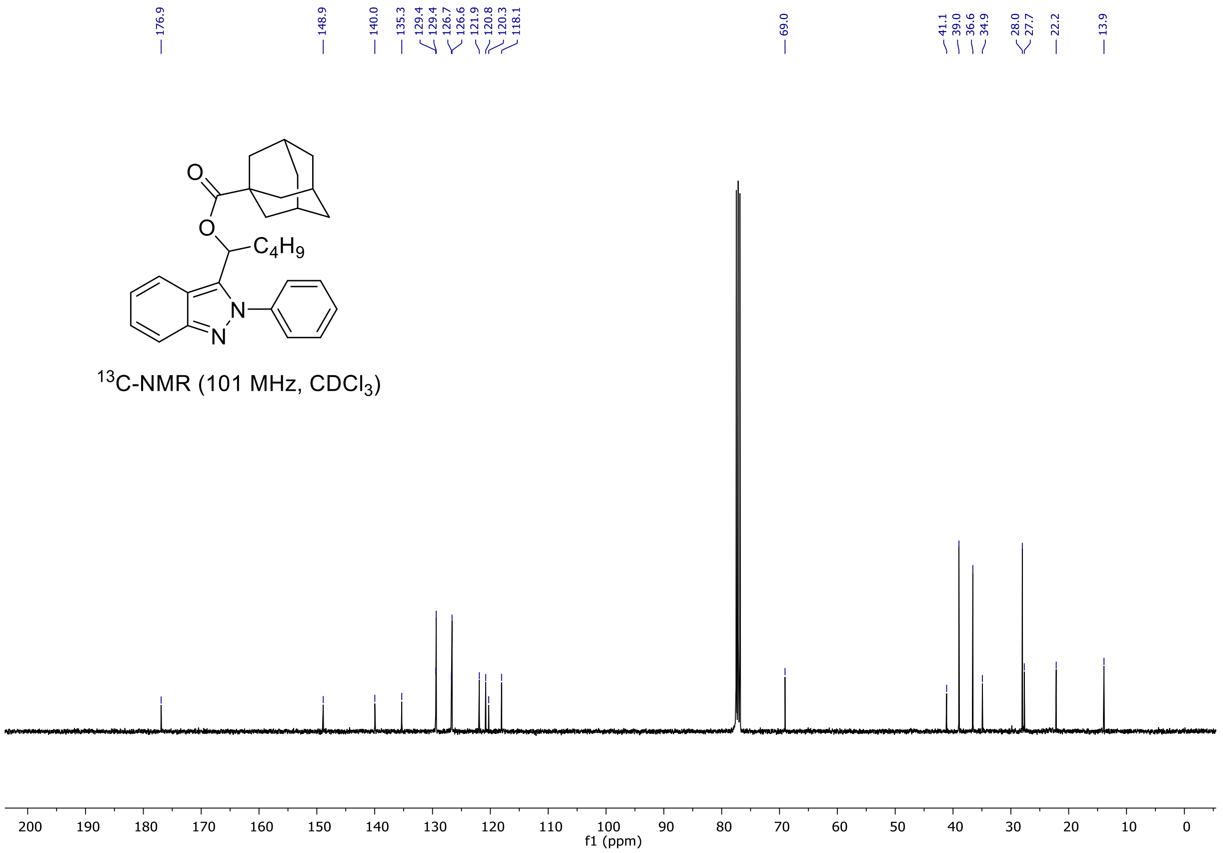

3-[1-(Ethylthio)pentyl]-2-phenyl-2*H*-indazole (2av)

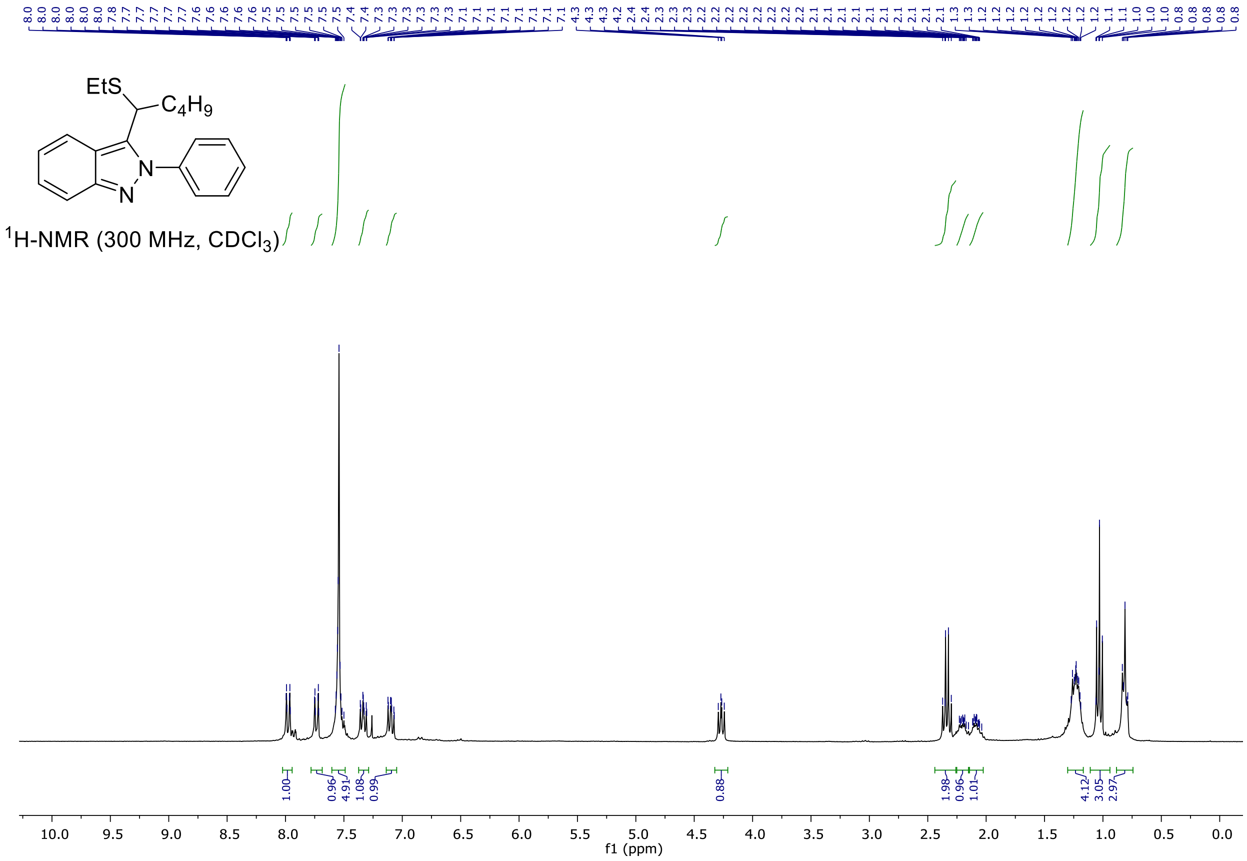

^{1}H-NMR (300 MHz, $CDCl_3$)

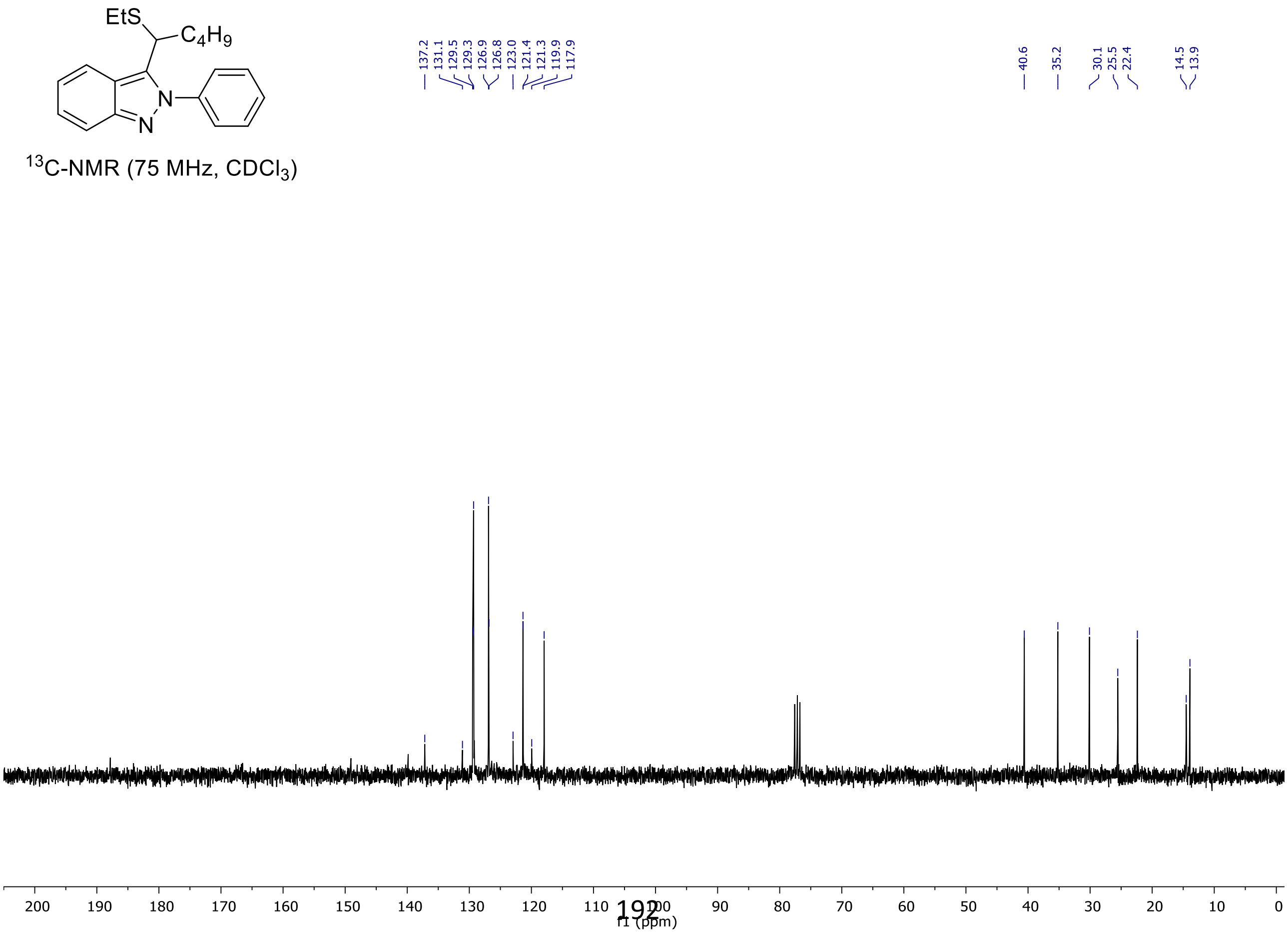

^{13}C-NMR (75 MHz, $CDCl_3$)

2-Phenyl-3-[1-(phenylthio)pentyl]-2*H*-indazole (2aw)

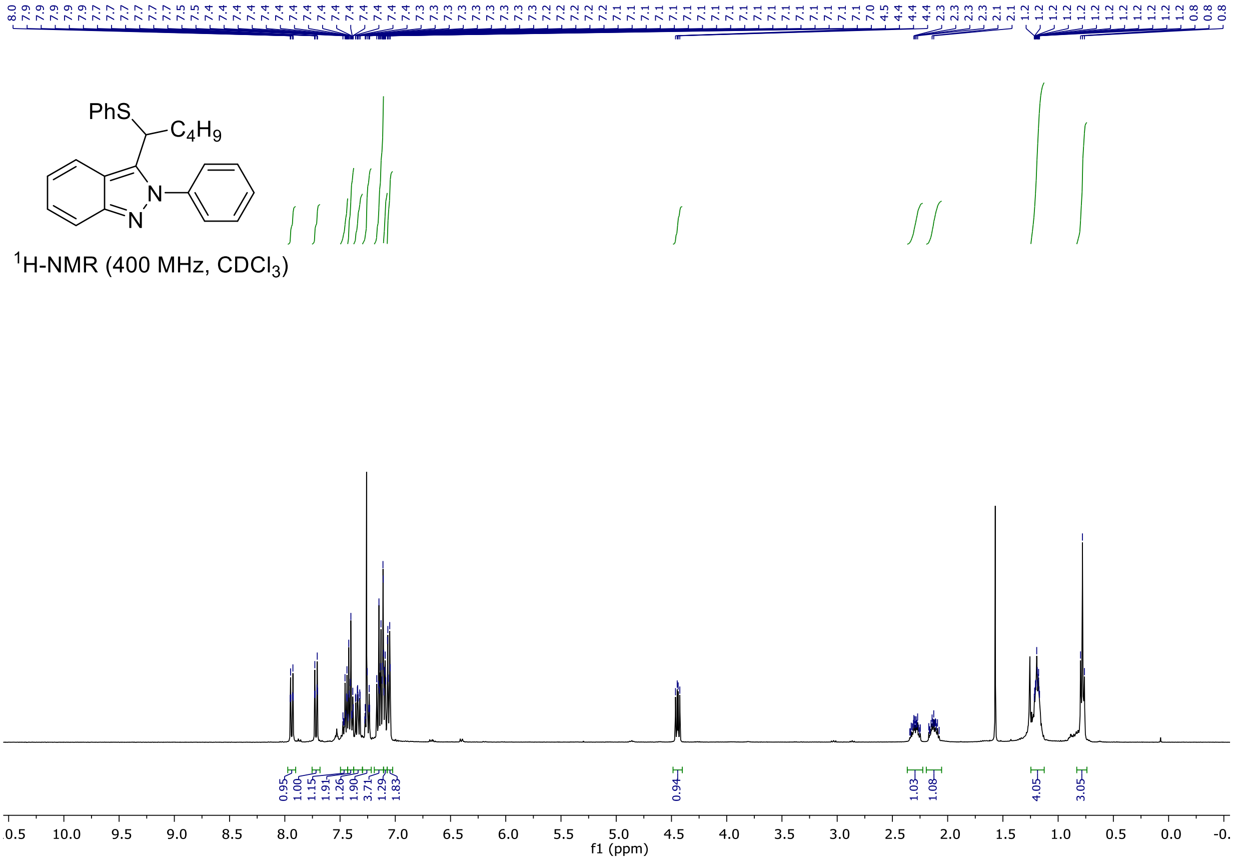

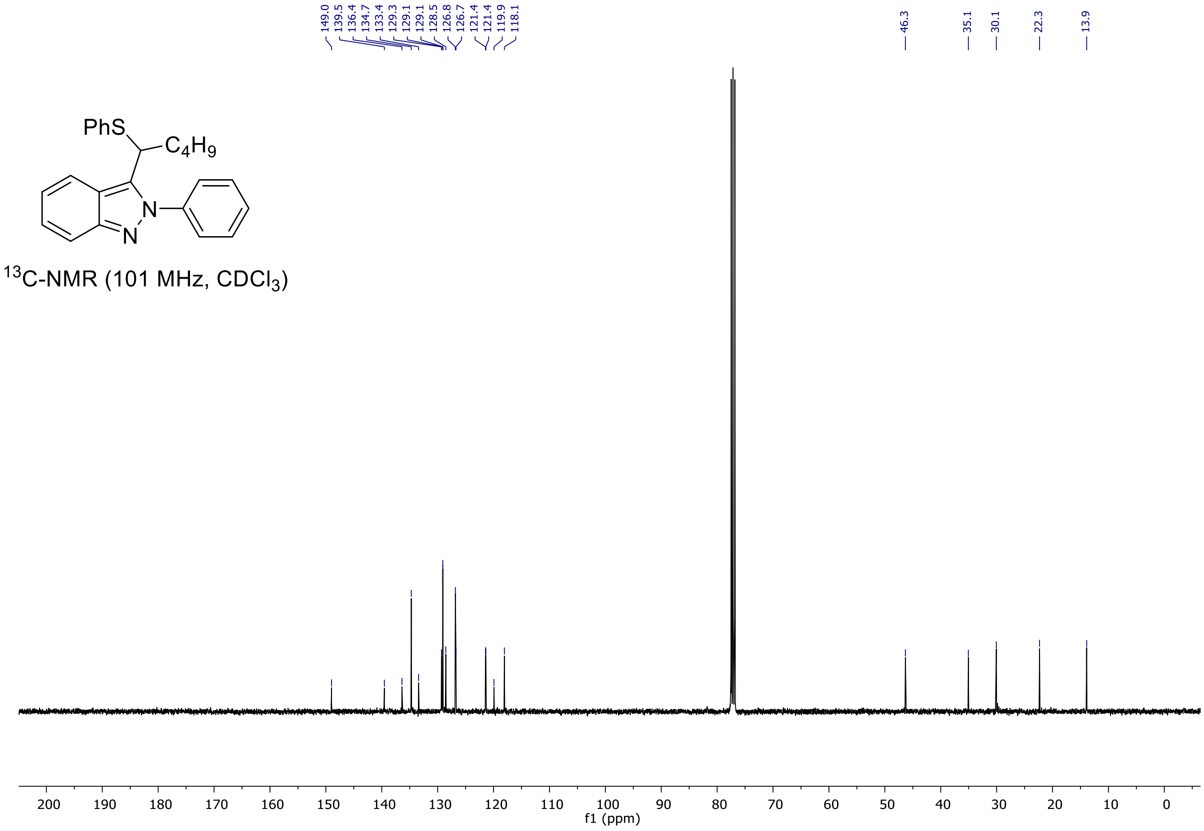

2-[(1-(2-Phenyl-2*H*-indazol-3-yl)pentyl)thio]ethanol (2ax)

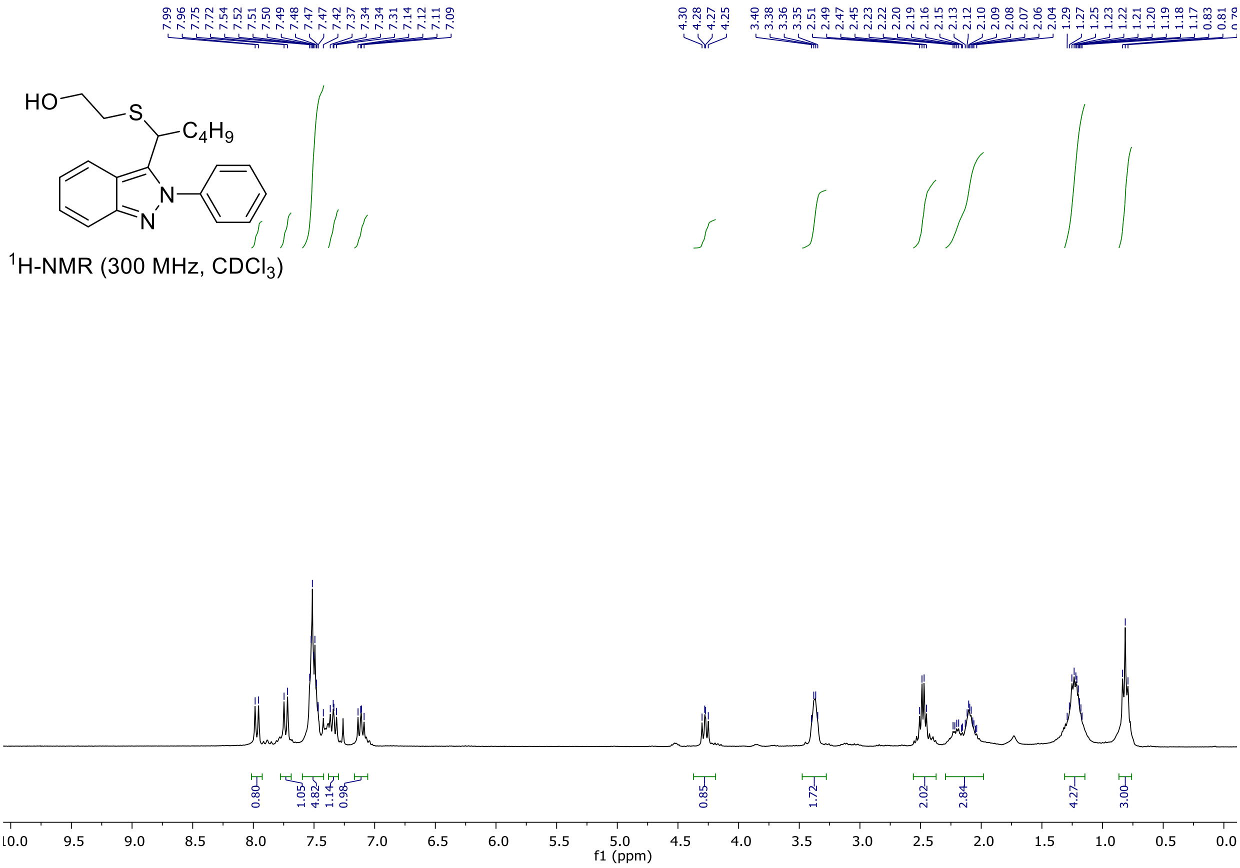

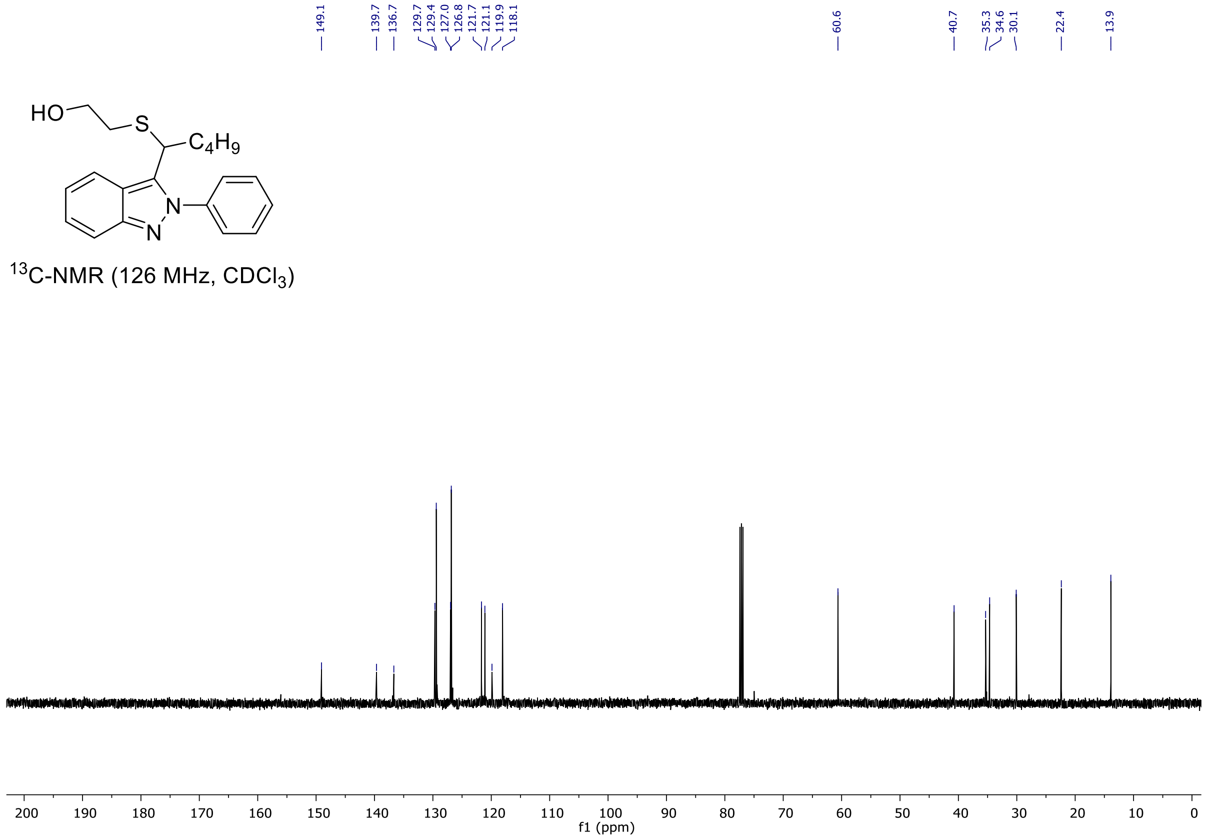

2-[(1-(2-Phenyl-2*H*-indazol-3-yl)pentyl)oxy]ethane-1-thiol (2ax´)

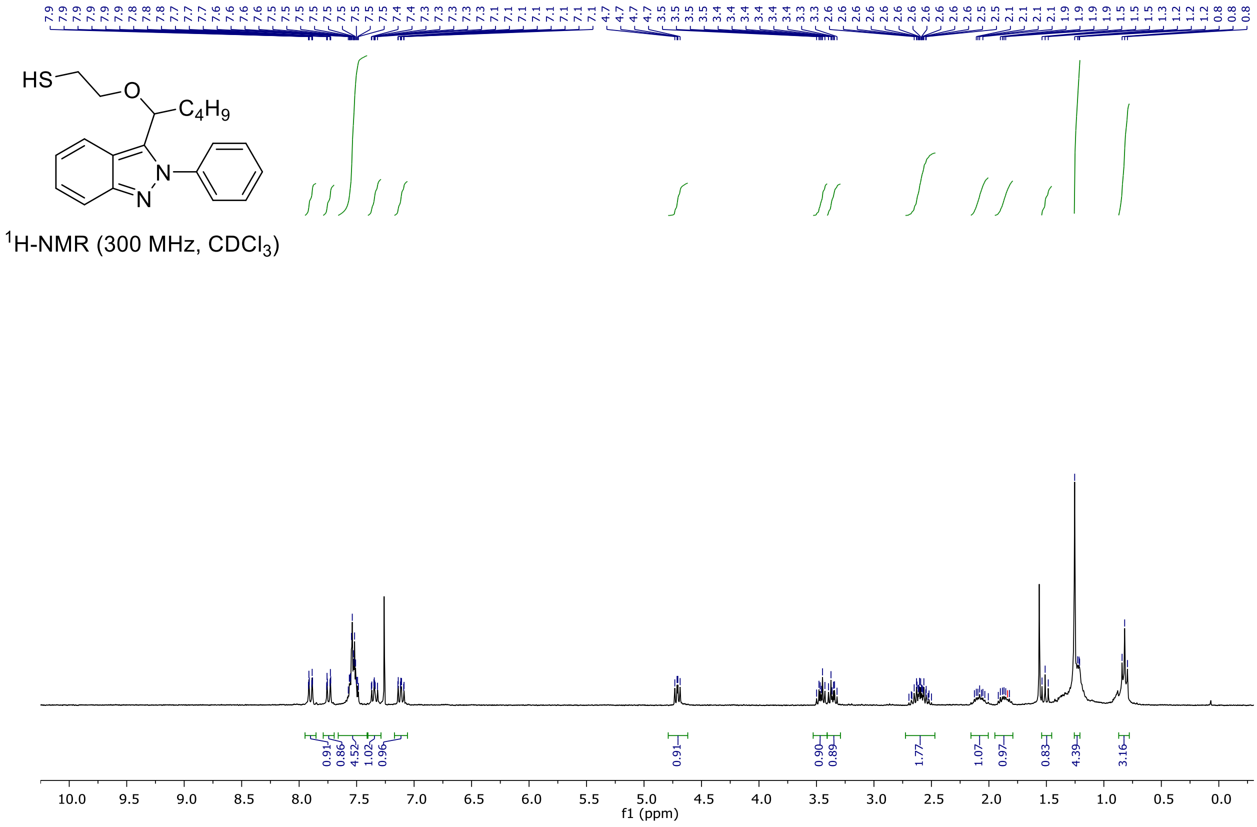

^{1}H-NMR (300 MHz, $CDCl_3$)

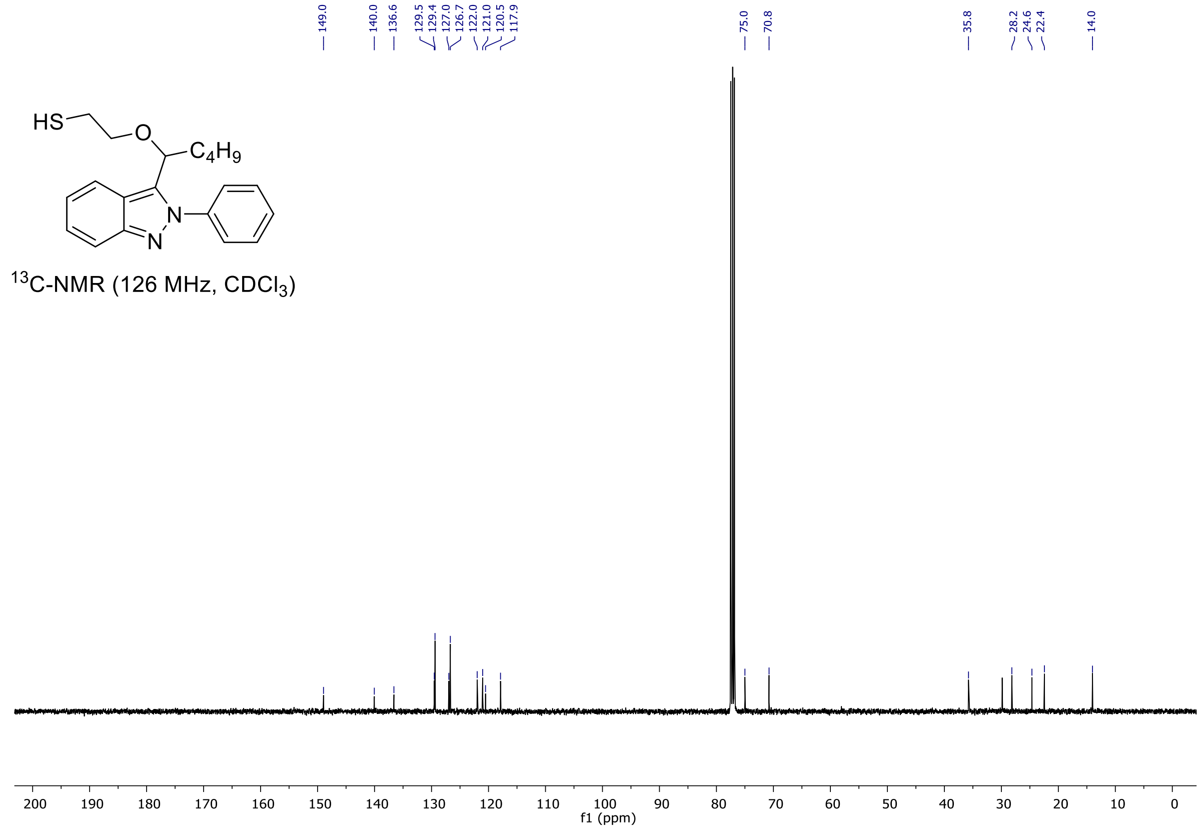

^{13}C-NMR (126 MHz, $CDCl_3$)

N-[1-(2-Phenyl-2*H*-indazol-3-yl)pentyl]acetamide (2ay)

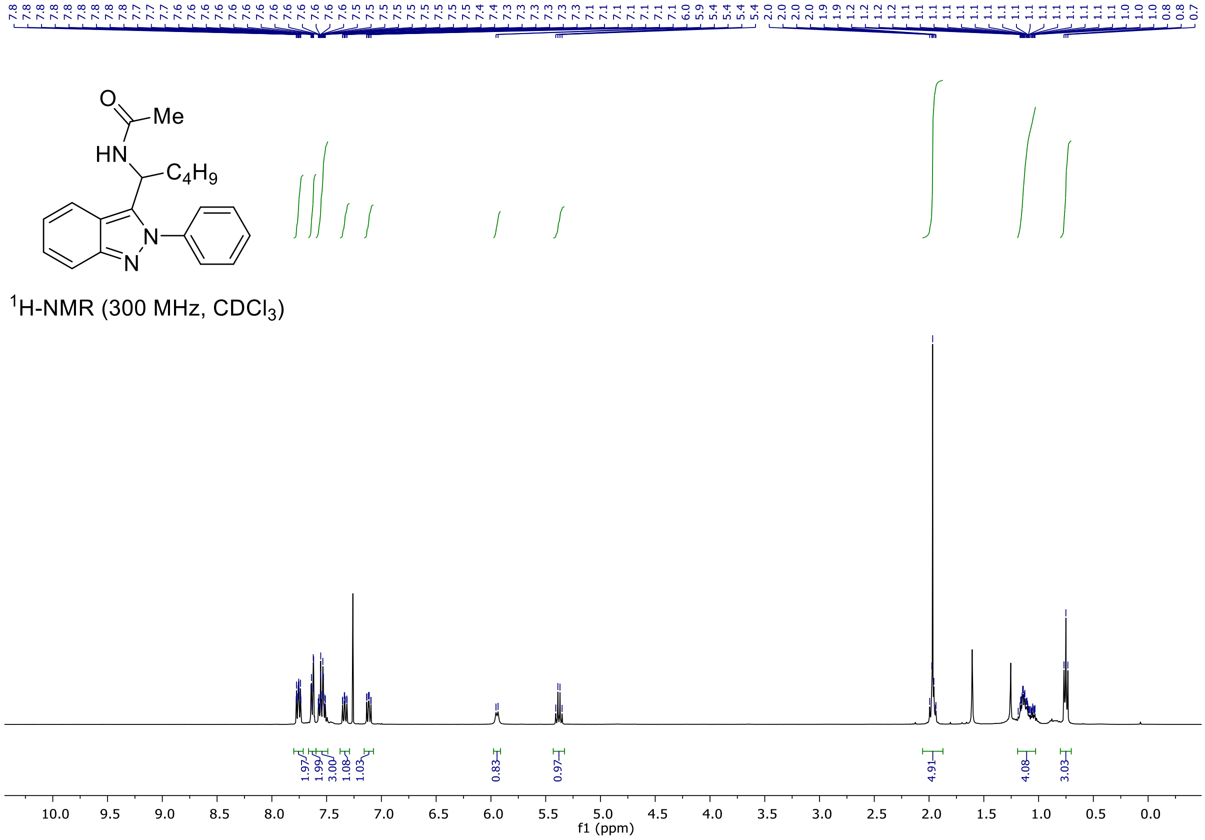

^{1}H-NMR (300 MHz, $CDCl_3$)

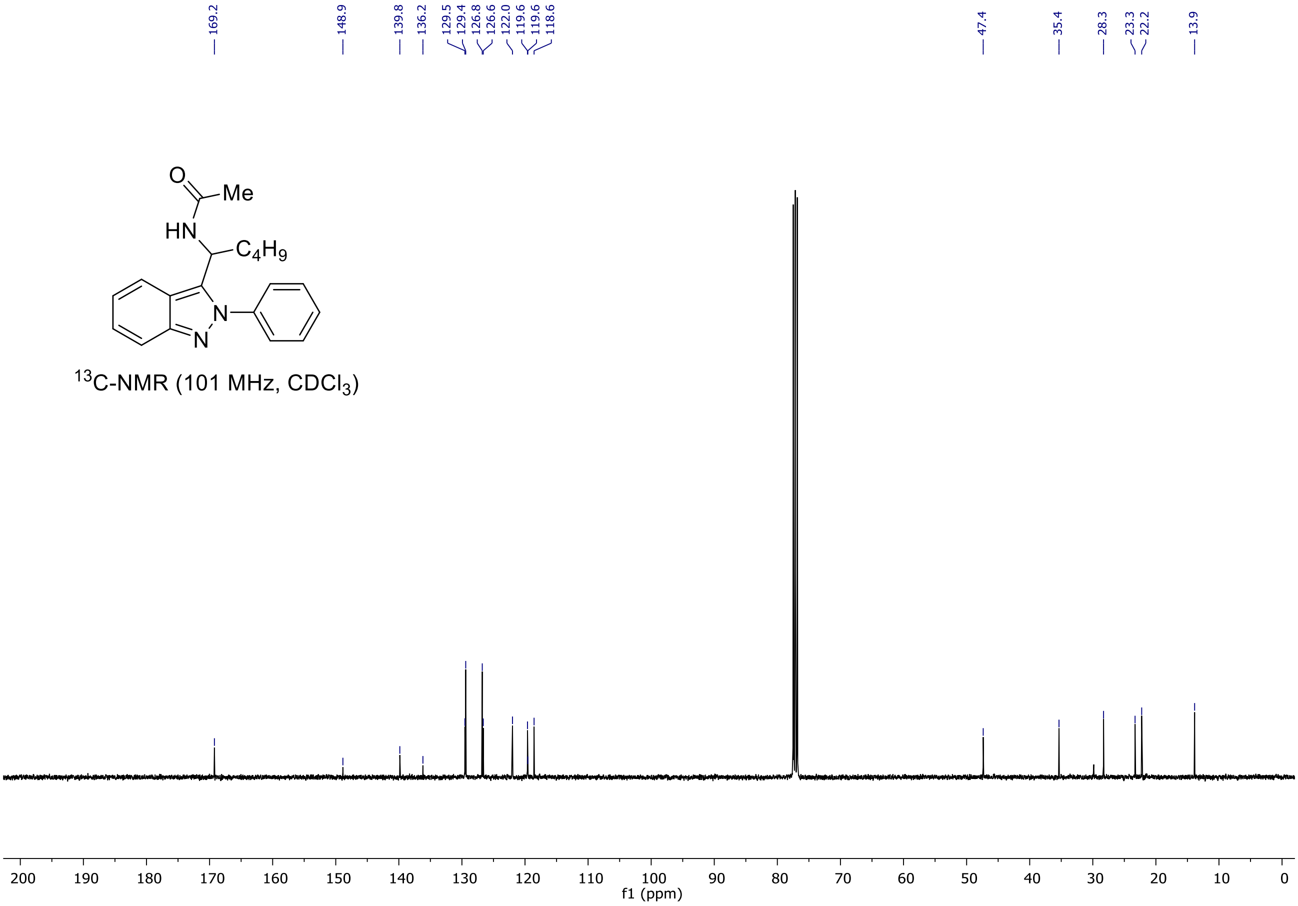

^{13}C-NMR (101 MHz, $CDCl_3$)

3-[1-(1H-Indol-1-yl)pentyl]-2-phenyl-2*H*-indazole (2az)

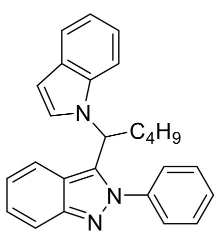

^{1}H-NMR (400 MHz, $CDCl_3$)

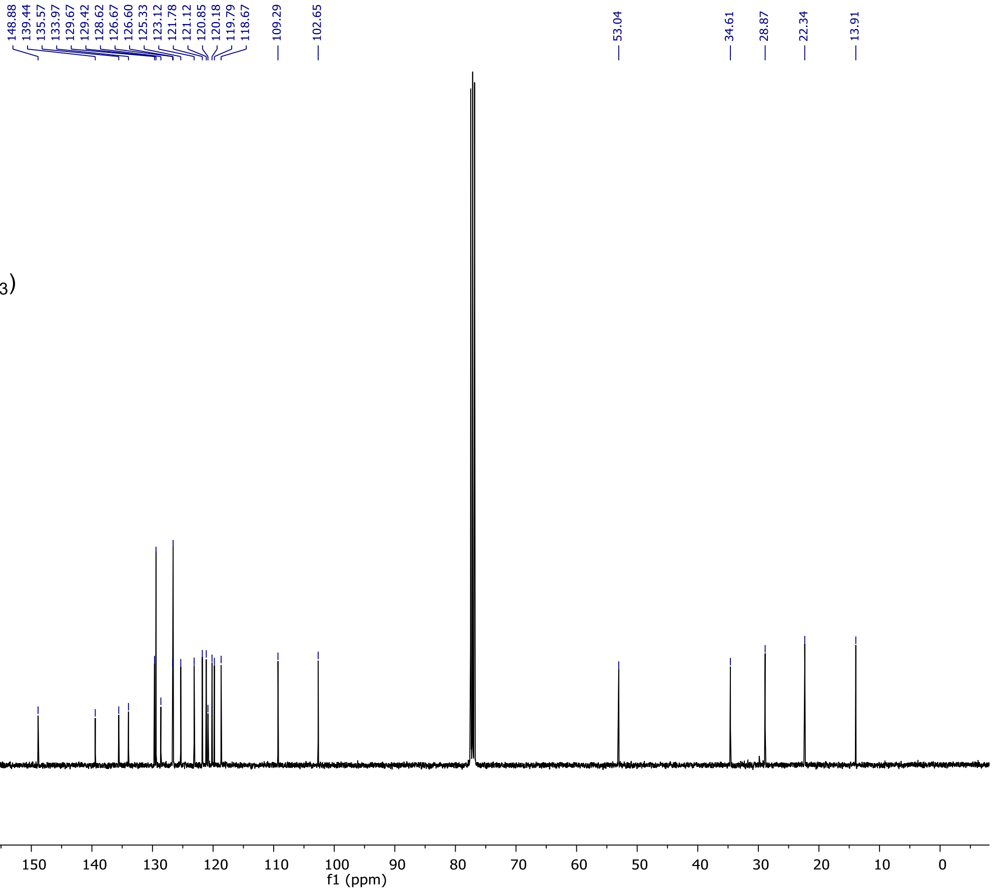

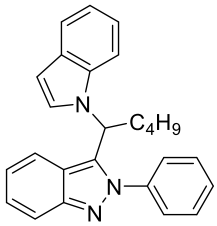

^{13}C-NMR (101 MHz, $CDCl_3$)

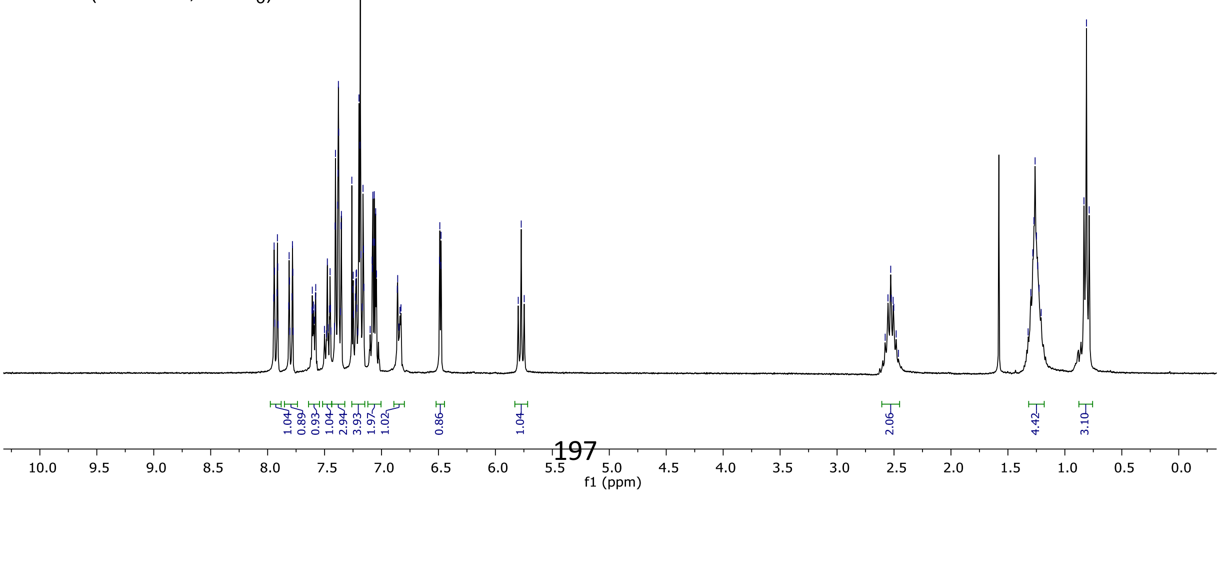

2-([1,1'-Biphenyl]-4-yl)-3-(1-methoxypentyl)-2*H*-indazole (2ba)

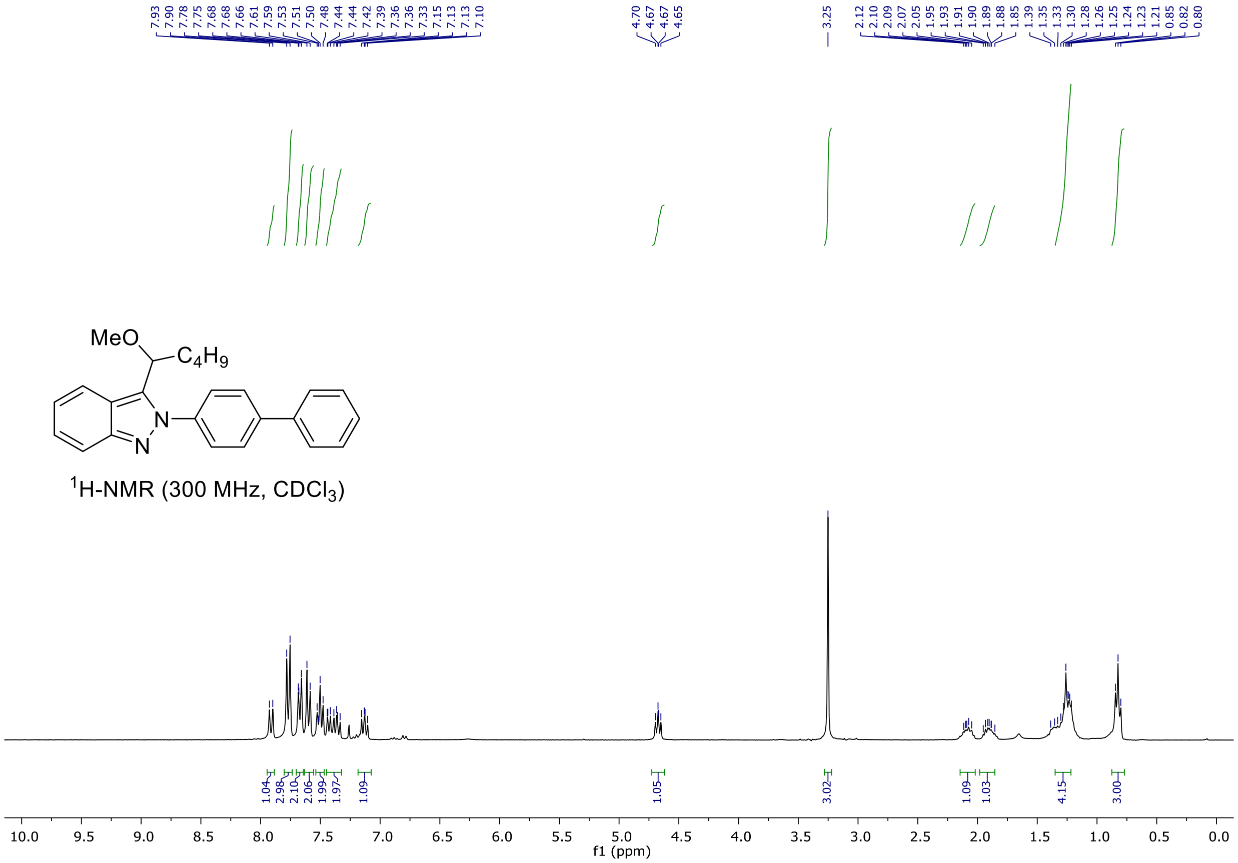

^{1}H-NMR (300 MHz, $CDCl_3$)

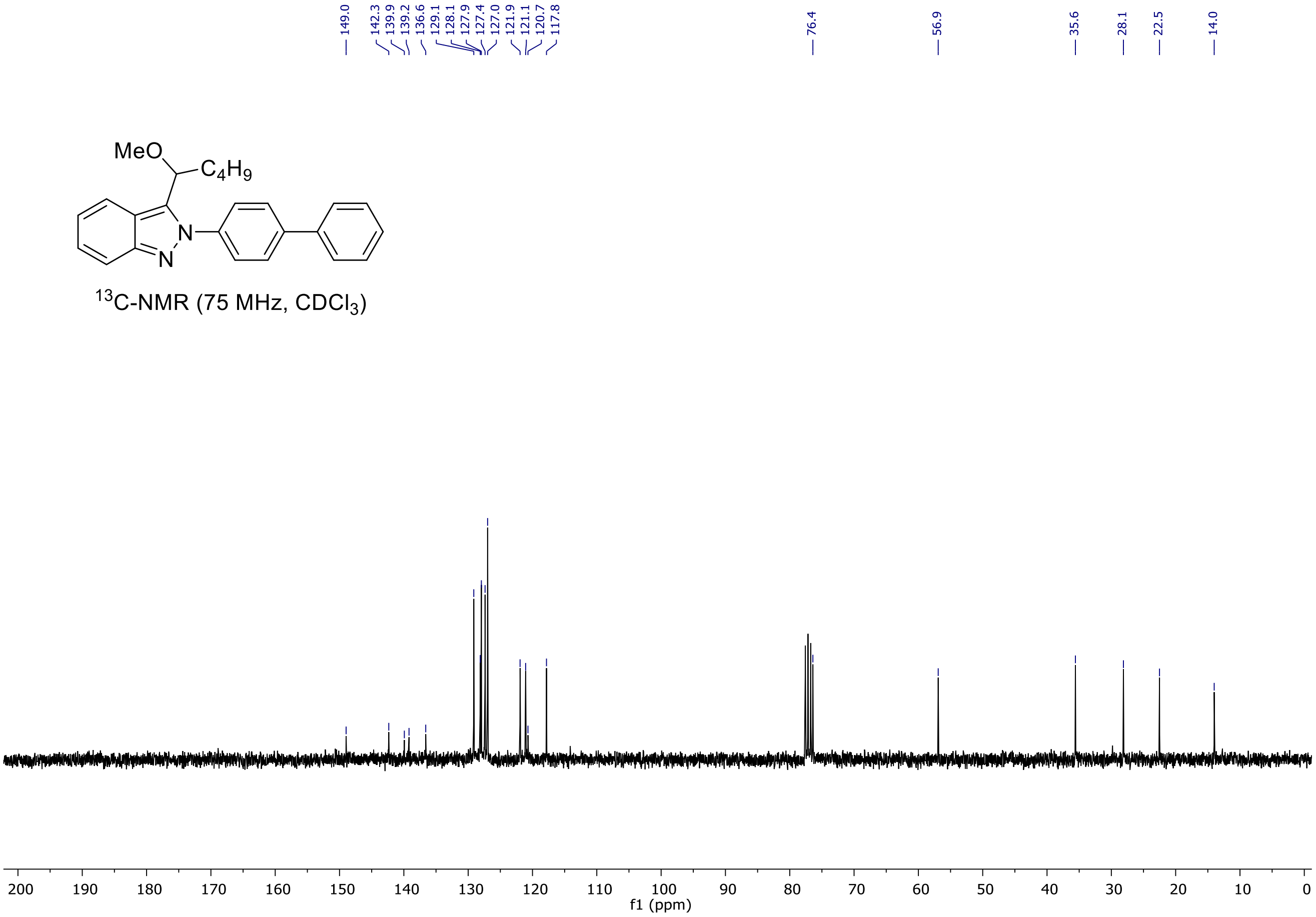

^{13}C-NMR (75 MHz, $CDCl_3$)

3-[3-(1-Methoxypentyl)-2*H*-indazol-2-yl]aniline (2bb)

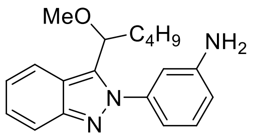

^{1}H-NMR (500 MHz, $CDCl_3$)

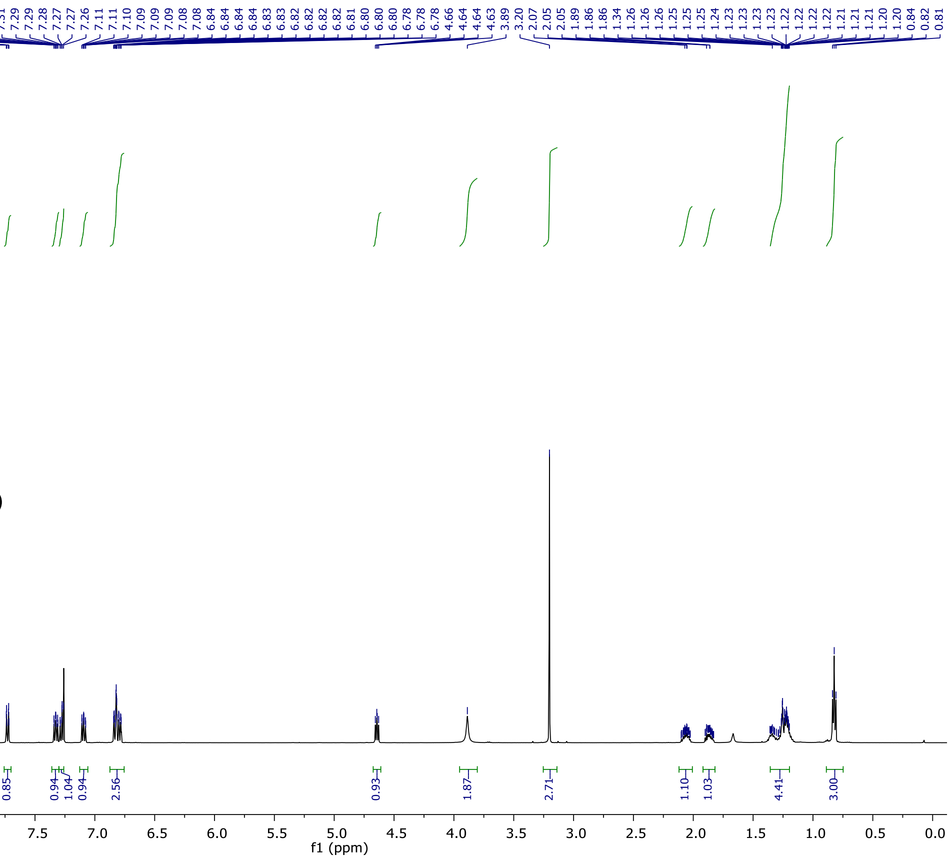

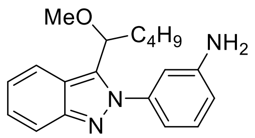

^{13}C-NMR (101 MHz, $CDCl_3$)

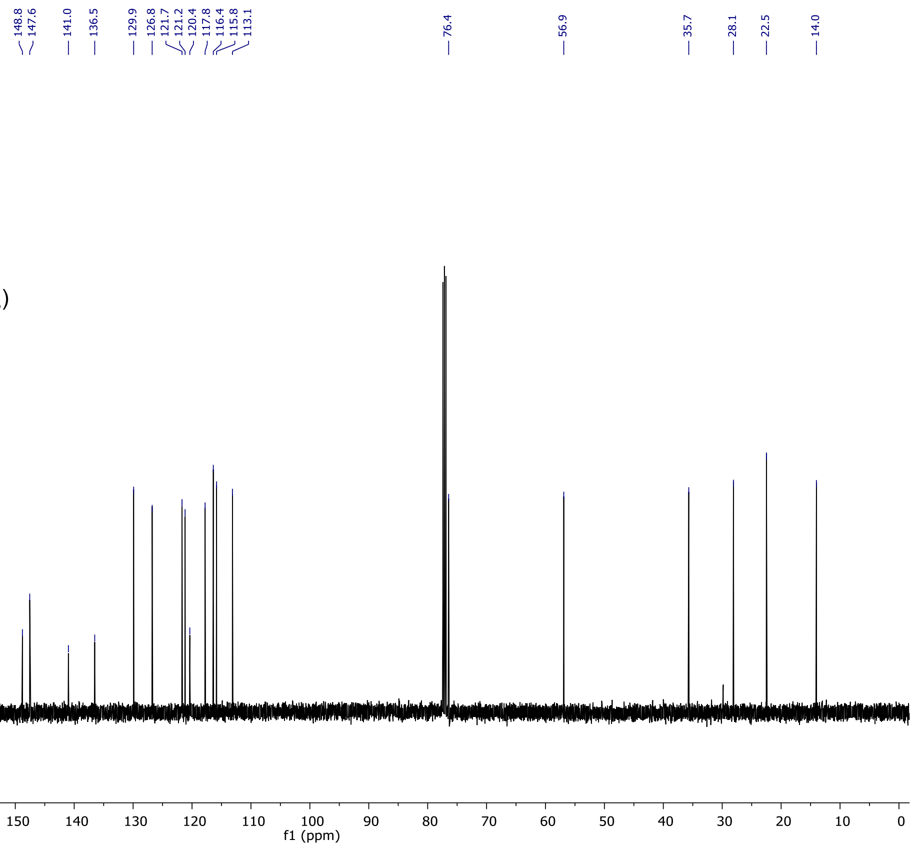

5-[3-(1-Methoxypentyl)-2*H*-indazol-2-yl]isophthalic acid (2bc)

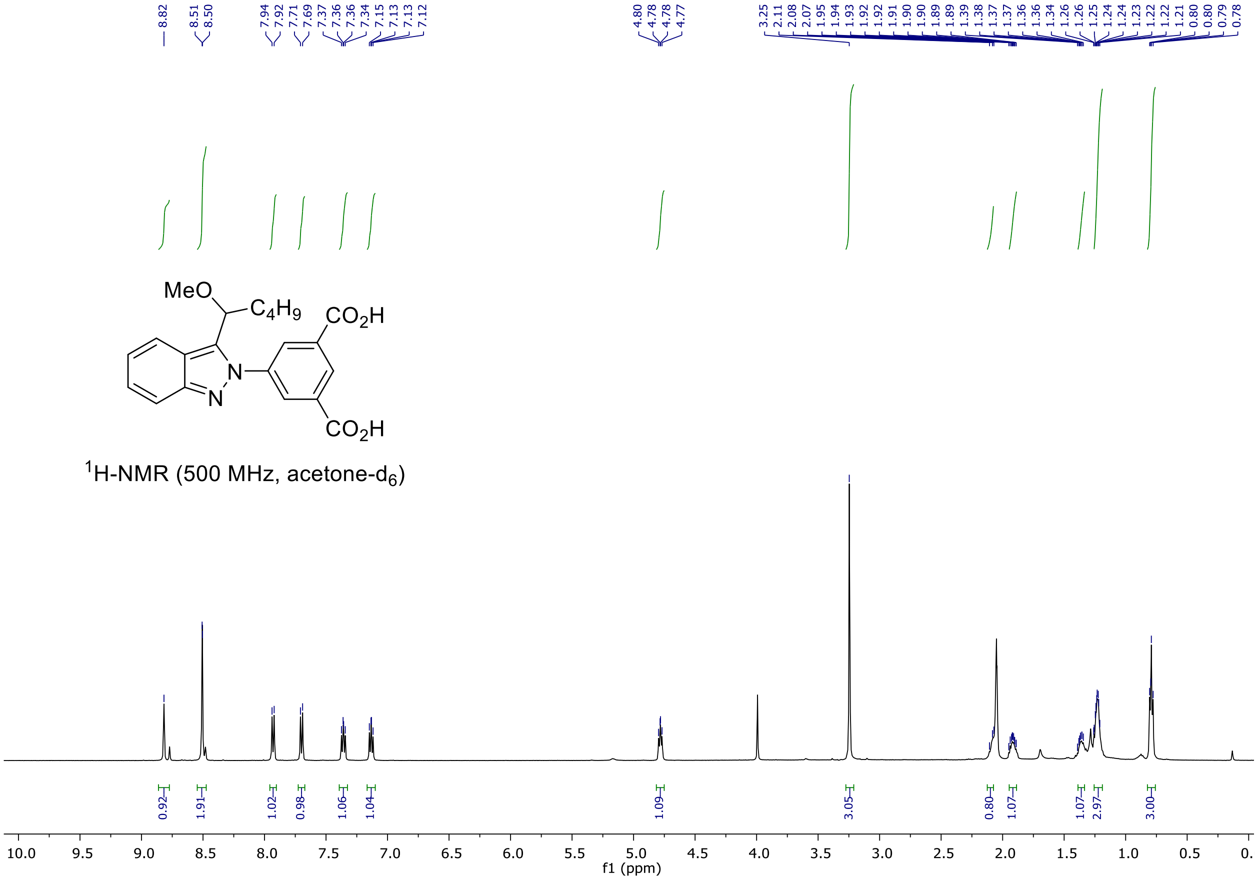

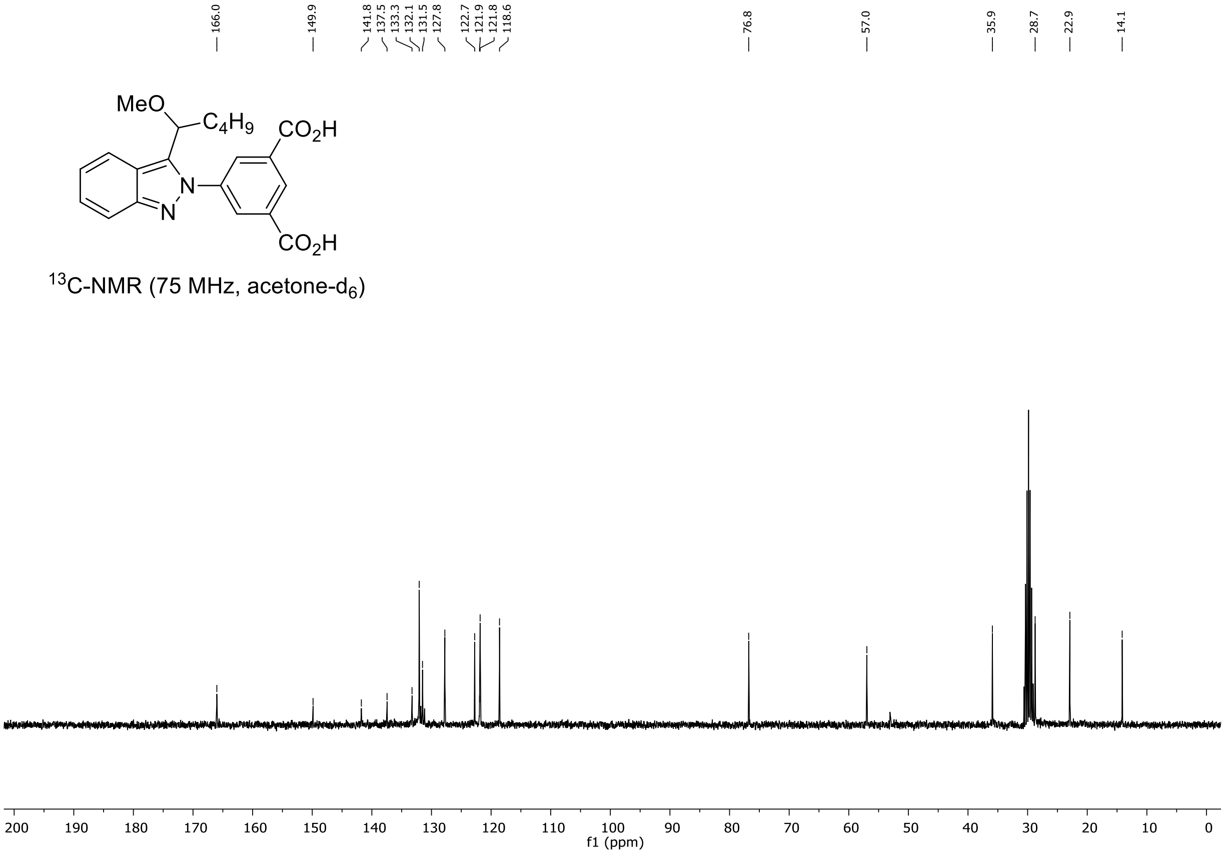

3-[1-(Ethylsulfonyl)pentyl]-2-phenyl-2*H*-indazole (2bd)

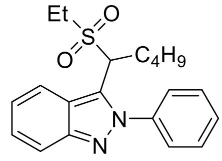

^{1}H-NMR (300 MHz, $CDCl_3$)

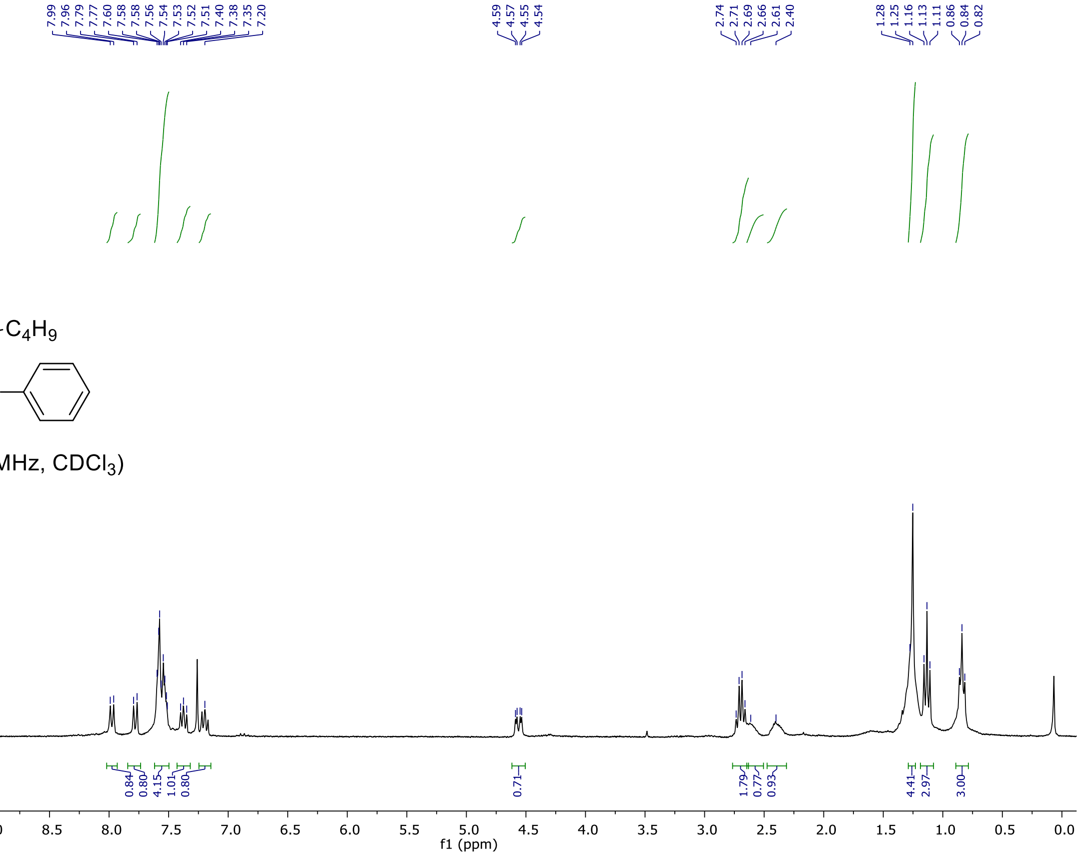

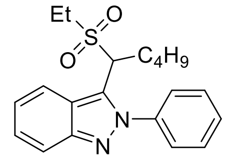

^{13}C-NMR (101 MHz, $CDCl_3$)

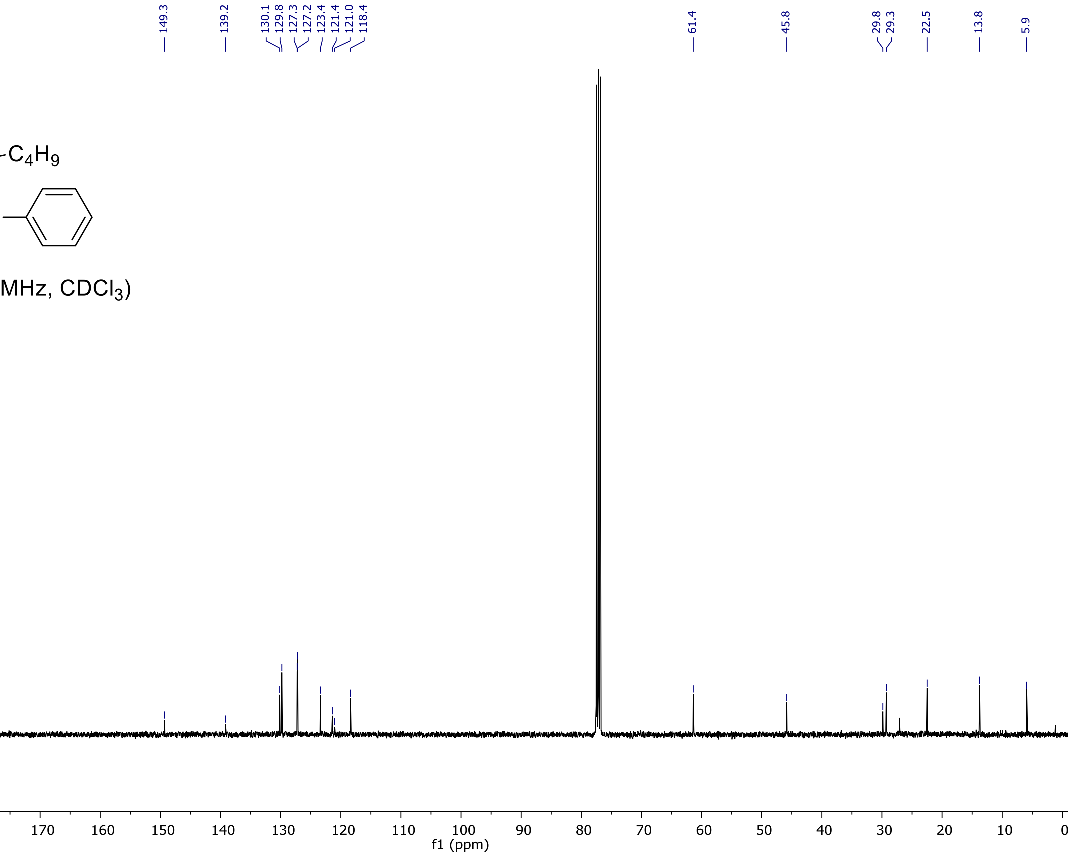

References

1. J. G. Ávila-Zárraga, R. Martínez, *Synth. Commun.* **2001**, *31*, 2177–2183.

2. C. Xu, Y. Feng, F. Li, J. Han, Y. M. He, Q. H. Fan, *Organometallics* **2019**, *38*, 3979–3990.

3. J. Guo, X. Wang, C. Ni, X. Wan, J. Hu, *Nat. Commun.* **2022**, *13*, 2752.

4. L. D. Shirtcliff, T. J. R. Weakley, M. M. Haley, F. Köhler, R. Herges, *J. Org. Chem.* **2004**, *69*, 6979–6985.

5. Gaussian 16, Revision C.01; Frisch, M. J. et al. Gaussian, Inc., Wallingford CT, 2016.

6. Lee, C.; Yang, W.; Parr, R. G. *Phys. Rev. B*, **1998**, *37*, 785-789; b) Becke, A. D. *Chem. Phys.* **1993**, *98*, 5648-5652; c) Kohn, W.; Becke, A. D.; Parr, R. G. *J. Phys. Chem.* **1996**, *100*, 12974-12980.
